# Multi-Platform Whole-Genome Microarray Analyses Refine the Epigenetic Signature of Breast Cancer Metastasis with Gene Expression and Copy Number

**DOI:** 10.1371/journal.pone.0008665

**Published:** 2010-01-13

**Authors:** Joseph Andrews, Wendy Kennette, Jenna Pilon, Alexandra Hodgson, Alan B. Tuck, Ann F. Chambers, David I. Rodenhiser

**Affiliations:** 1 London Regional Cancer Program, London Health Sciences Centre, London, Ontario, Canada; 2 Department of Biochemistry, The Schulich School of Medicine and Dentistry, University of Western Ontario, London, Ontario, Canada; 3 Department of Oncology, The Schulich School of Medicine and Dentistry, University of Western Ontario, London, Ontario, Canada; 4 Department of Paediatrics, The Schulich School of Medicine and Dentistry, University of Western Ontario, London, Ontario, Canada; 5 Department of Pathology, The Schulich School of Medicine and Dentistry, University of Western Ontario, London, Ontario, Canada; 6 EpiGenWestern Research Group at the Children's Health Research Institute, London, Ontario, Canada; Health Canada, Canada

## Abstract

**Background:**

We have previously identified genome-wide DNA methylation changes in a cell line model of breast cancer metastasis. These complex epigenetic changes that we observed, along with concurrent karyotype analyses, have led us to hypothesize that complex genomic alterations in cancer cells (deletions, translocations and ploidy) are superimposed over promoter-specific methylation events that are responsible for gene-specific expression changes observed in breast cancer metastasis.

**Methodology/Principal Findings:**

We undertook simultaneous high-resolution, whole-genome analyses of MDA-MB-468GFP and MDA-MB-468GFP-LN human breast cancer cell lines (an isogenic, paired lymphatic metastasis cell line model) using Affymetrix gene expression (U133), promoter (1.0R), and SNP/CNV (SNP 6.0) microarray platforms to correlate data from gene expression, epigenetic (DNA methylation), and combination copy number variant/single nucleotide polymorphism microarrays. Using Partek Software and Ingenuity Pathway Analysis we integrated datasets from these three platforms and detected multiple hypomethylation and hypermethylation events. Many of these epigenetic alterations correlated with gene expression changes. In addition, gene dosage events correlated with the karyotypic differences observed between the cell lines and were reflected in specific promoter methylation patterns. Gene subsets were identified that correlated hyper (and hypo) methylation with the loss (or gain) of gene expression and in parallel, with gene dosage losses and gains, respectively. Individual gene targets from these subsets were also validated for their methylation, expression and copy number status, and susceptible gene pathways were identified that may indicate how selective advantage drives the processes of tumourigenesis and metastasis.

**Conclusions/Significance:**

Our approach allows more precisely profiling of functionally relevant epigenetic signatures that are associated with cancer progression and metastasis.

## Introduction

A variety of whole-genome approaches have been used to identify the molecular profiles that contribute to and reflect cancer progression. To date, the scope of such whole genome profiling efforts has encompassed classical karyotypic analyses that identify chromosomal rearrangements [1], as well as gene expression profile [2] and epigenetic studies (including by our group; [3], [4]) that provide snap-shot signatures of gene expression and chromatin modification patterns, respectively. In the context of breast cancer, whole genome approaches have identified prognostic gene sets that predict a short interval to distant metastases (i.e. a poor prognosis signature; [5], [6], [7]) and described gene profiles that mediate metastasis to a secondary site [8], [9], [10]. However, few reports have identified epigenetic signatures of breast cancer, particularly in the context of epigenetic mechanisms of tumourigenesis that could be applied in cancer management. Such applications could provide diagnostic tests, prognostic factors and predictors of treatment response that would complement standard gene-expression based assays [11]. Furthermore, the integration of data sets from multiple whole genome platforms is complicated by the interrelated nature of the genetic and epigenetic signatures that characterize individual cells in both their normal or tumourigenic states [12]. For example, gene expression can be regulated at multiple levels that include epigenetic modulation by DNA methylation or through histone modifications that modify chromatin accessibility. In addition, recent reports show that gene copy number adds a further level of complexity in understanding the molecular context of health and disease states. This is because variations in gene dosage can complicate the interpretation of microarray data and may also differentially contribute to the developmental or tumour phenotype [13]. In only a few reports have such whole genome profiling efforts been integrated [14], [15], allowing a multi-dimensional characterization of biological systems [16]. In perhaps the most thorough report to date, integrative analysis of global cancer-related changes in DNA methylation, genomic imbalance, and gene expression provided evidence of the cumulative roles of epigenetic and genetic mechanisms in deregulation of gene expression networks in osteosarcoma [17], providing evidence for the combined contributions of genetic, epigenetic and chromosomal (cytogenetic) alterations to tumour progression.

Previously, we described the first use of a human gene promoter tiling microarray platform to identify genome-wide DNA methylation patterns in a human breast cancer cell line model of metastasis [4]. Gene networks and pathways were identified in MDA-MB-468GFP (468GFP) and MDA-MB-468LN (468LN) cells and selected target genes associated with epithelial–mesenchymal transition (EMT) were validated with respect to DNA methylation effects on gene expression. Although we found that many hypermethylation and hypomethylation events were interspersed across the genome, we also observed an apparent clustering of methylation events within identifiable chromosomal regions. For example, enriched regions of hypermethylation events were identified on chromosomes 6p, 7p, 11p/q, 18p and 19p/q, and similar clustering of hypomethylated events (on 1p, 3q, 7q and 20q) was also found [3], [4]. Analyses were subsequently undertaken that identified complex chromosomal rearrangements, including deletions, translocations and ploidy differences between these cell lines [1]. We also observed that a number of these chromosomal rearrangements included regions to which we had assigned clustered methylation signatures in our promoter methylation profiling study. Thus, these complementary data sets generated by our two studies have led us to hypothesize that complex genomic alterations in cancer cells (deletions, translocations and ploidy) are superimposed over promoter-specific methylation events responsible for gene-specific expression changes in breast cancer metastasis.

Here we tested this hypothesis using whole-genome platforms to cross reference gene expression, epigenetic and copy number data, in this human model of breast cancer metastasis [18]. Briefly, we undertook simultaneous high-resolution, whole-genome analyses using Affymetrix gene expression (U133) promoter (1.0R) and Copy Number Variation/Single Nucleotide Polymorphism (SNP 6.0) microarray platforms to correlate gene expression, epigenetic (DNA methylation), and gene copy number information. We observed widespread hypomethylation and hypermethylation differences within this cell line pair, many of which (∼650) correlate with gene expression changes. In addition, gene dosage events correlated with the profound karyotypic differences between the cell lines and were reflected in the methylation patterns that we observed. We used Partek Software analyses to integrate the three microarray platforms and to identify subsets of genes with methylation/expression patterns that were either dependent (or independent) of gene copy number. These included the SFN, TMEM16A, WNT5A, DLC1 and HOXD13 genes, the expression of which were validated by Quantitative Real Time PCR (qRT-PCR). Our integrated report thus provides an algorithm to logically assess prospective gene targets that are epigenetically regulated and those that are altered by copy number (but are not epigenetically modified) thus allowing us to refine the epigenetic signatures of breast cancer.

## Materials and Methods

### Cell Culture and Genomic DNA (gDNA)/Total RNA Extraction

MDA-MB-468GFP (468GFP) and MDA-MB-468GFP-LN (468LN) human breast cancer cell lines were isolated and characterised as described previously [18]. For gene expression studies, cells were grown from frozen stocks in αMEM medium (Invitrogen) Supplemented with 10% FCS (Wisent Inc.) for 4 passages, without the use of antibiotics. At the fifth passage, each cell line was split into parallel flasks (three each for expression and promoter analysis, and two for Copy Number Variation/Single Nucleotide Polymorphism (CNV/SNP) analysis, and grown to approximately 70% confluence. In some experiments, 468LN cells were cultured (with antibiotics) for 72 hours in the presence of 10 µM 5-aza-2′-deoxycytidine (5-azaC), with or without an additional 16 hour exposure to the histone deacetylase inhibitor Trichostatin A (TSA; 50 nM). A fourth treatment group with 5-azaC for 88 hours was also added. For gene expression microarrays, total RNA from each biological replicate was isolated using Trizol (Invitrogen) as per the manufacturer's instructions. For CNV/SNP arrays, genomic DNA (gDNA) was isolated from each flask separately using the GenElute Genomic DNA Miniprep kit (Sigma-Aldrich, St. Louis, MO, USA), as per the manufacturer's instructions. All microarray hybridizations, staining, washing, scanning, and data analyses were carried out at the London Regional Genomics Centre [47]. A complete list of PCR primers used in this study is presented in Table S1.

### Gene Expression Microarrays

Total RNA from each biological replicate was isolated using Trizol (Invitrogen) as per the manufacturer's instructions. 10 micrograms of RNA was used to produce Biotin-labeled cRNA, which was hybridized to Affymetrix HGU133_Plus_2 arrays. Array washing, scanning and probe quantification were carried out as per the manufacturer's instructions using GCOS software [48], except that the target intensity was set to 150. For each array, GCOS output was imported as. CEL files into Partek Genomic Suite software (Agilent), and data were normalized using the RMA (Robust Multichip Averaging) algorithm. ANOVA with nominal alpha value set to 0.05 was then used to determine those probe sets significantly different between the 468GFP and 468LN cell lines, followed by a Benjamini and Hochberg Multiple testing correction to reduce the false positive rate. These results were then separated by significant increasers or decreasers, and used in cross platform analysis.

### Human Promoter Microarrays

Analysis of hyper- and hypomethylated promoters in 468GFP vs 468LN was carried out using Affymetrix Human Promoter 1.0R arrays as described previously [4]. The annotations for the HGU133_Plus_2 array were used to determine which probe sets were associated with regions appearing to be significantly hyper- or hypomethylated in 468LN vs 468GFP cells, and these probe set IDs were used in cross platform analyses.

### CNV/SNP Microarray Analysis

To detect copy number variations in the 468LN vs the 468GFP cell lines, 4 µg of the same genomic DNA used in promoter analysis was labelled, fragmented, and hybridized to Affymetrix SNP 6.0 arrays. This array contains probes used in SNP analysis, as well as probes specific for CNV detection. CEL files produced by GCOS software for each array were then imported into Partek Genomic Suite and analyzed using the Copy Number Analysis workflow. All. CEL files were background corrected using RMA as above, and results were corrected for probe GC content and fragment length. 468LN cells were compared directly to 468GFP cells. Significantly different regions were determined using the Hidden Markov Model algorithm of the Partek Genomic Suite set to detect copy number (CN) states of 0.1, 1, 3, 4, 5 (a CN state of 2 was ignored), with the minimum number of probe sets contained in a region for it to be considered set to 3. Only regions of CNV showing up in two of two replicates were reported, and both SNP and CNV probes were used in the analysis. Regions identified were annotated with gene symbols by importing the annotation file from the UCSC genome browser (build hg18). Affymetrix Probe IDs from the HGU133_ Plus_2 array for the genes in all regions appearing either increased or decreased in copy number in the 468LN/468GFP direct comparison were obtained by submitting the gene symbols to Affymetrix through the Netaffx tool [49]; (http://www.affymetrix.com/analysis/index.affx). Alternatively, 468GFP or 468LN samples were compared to samples taken from normal females. As a normal reference population, we used a subset of 60 Yoruba (YRI; Ibidan, Nigeria) females of the 270 samples from the International HapMap project, which were run on the SNP 6.0 array (data available at Affymetrix). The microarray data discussed in this publication have been deposited in the National Center for Biotechnology Information's Gene Expression Omnibus (GEO) under accession GSE15619, along with detailed protocol notes.

### Cross Platform Venn Analysis

Probe set IDs from each of the three Microarray platforms were imported as separate lists into Genespring 7.3 GX (Agilent), and compared using the Venn Analysis tool. Probe sets appearing hypomethylated, increased in expression, and increased in copy number were compared in one analysis, and probe sets appearing hypermethylated, decreased in expression, and decreased in copy number in another. Lists of overlapping probe sets were then generated, and filtered in EXCEL to determine the number of unique genes represented. Proportionate Venn diagrams were then created based on these data sets.

### Bisulfite Genomic Sequencing

Hyper- or hypomethylation of regions predicted from the array analysis were confirmed using a variation of the bisulfite conversion method [19]. Genomic DNA (gDNA; 2 µg) was bisulfite treated using the Epitect DNA bisulfite treatment kit (Qiagen) as per the manufacturer's instructions. Primers specific to the converted gDNA were designed using the MethPrimer software [20] with the default parameters, except that amplicons were designed to be between 200 and 500 base pairs. For each cell line, 60 ng of each converted gDNA was subjected to PCR in 1X buffer, 200 uM dNTPs, 2.0–2.5 mM MgCl_2_, 400 nM forward and reverse primers (Sigma-Genosys), and 1 Unit *Taq* Polymerase (Invitrogen). The cycling conditions used were 1 cycle of 94°C for 5 minutes, followed by 5 cycles of 94°C for 1 minute, 55°C for 2 minutes, and 72°C for 2.5 minutes. This was followed by 35 cycles of 94°C for 1 minute, 55°C for 1 minute, and 72°C for 1.5 minutes. PCR products were visualized using agarose gel electrophoresis/ethidium bromide staining, and PCR products purified using the Qiaquick PCR purification kit. For each CpG island to be tested, 25 ng of purified PCR products from each cell line were then ligated into the T-vector PCR2.1 (Invitrogen) overnight at 14°C as per the manufacturer's instructions. Plasmids thus generated were transformed into TOP10 competent bacteria using the heat shock method and transformed bacteria were spread onto LB-Agar plates containing 100 µg/mL ampicillin, and 50 µL of 10 mg/mL 5-bromo-4-chloro-3-indolyl-beta-D-galactopyranoside (X-gal). Plates were incubated overnight at 37°C, and white colonies were picked and “patched” onto fresh LB-Agar plates. Potential clones were directly screened by PCR using gene specific primers, and clones showing the expected band size were inoculated into 2 mL of TB containing 100 µg/mL ampicillin and grown overnight at 37°C. Plasmid DNA was isolated using the Genelute Plasmid Miniprep kit (Sigma), and sequenced using the T7 promoter primer. Clone sequences thus obtained were compared to the expected sequence using the ClustalW alignment algorithm [21].

### Quantitative Real Time PCR (qRT-PCR)

qRT-PCR was used to confirm the effect of promoter methylation on gene expression. For each cell line, RNA used for expression microarray analysis was also used to synthesize cDNA with Superscript II (Invitrogen), as per the manufacturer's instructions. Real-time primers were then designed for each gene using Primerquest Software (Integrated DNA Technologies, [50]) with 18S chosen as the reference gene. Reactions (in triplicate for each biological replicate) used RT^2^ qPCR Mastermix (SABiosciences, Frederick, MD, USA), 200 nM forward and reverse primers, 200 uM dNTPs, and 1 uL cDNA diluted 1∶5 or 1∶10, using the Rotorgene RG-3000 thermocycler (Corbett Research, Kirkland, PQ, Canada). Standard curves were generated for each gene, using cDNA derived from a serial 3 fold dilution of cDNA derived from one of the biological replicates. cDNA from 468GFP cells was used for the standard curves, where the gene was expected to be increased in its expression in this line relative to 468LN; otherwise cDNA from the 468LN cell line was used. For each biological replicate, relative amounts of each gene were determined by comparison to the standard curve. The “unknown” samples were then normalized to 18S, and the expression level in the parental 468GFP cell line was set to 1. Results are presented as fold change relative to control. A similar method was used to examine the change in expression of SFN, TMEM16A and WNT5A in 468LN cells after treatment with 5-azaC, with or without TSA, except that GAPDH was used as the internal control gene.

qRT-PCR was also used to confirm copy number. Exonprimer software [51] was used to design primers that amplify across an exon for each gene of interest. The human mispriming library was used to decrease the probability of cross hybridization of primers. Standard curves for each primer set were generated using serial 5 fold dilutions of normal human female DNA (Novagen), starting at 30 ng/µL. qRT-PCR was carried out as above, except that 15 ng per reaction of the same genomic DNA used in the promoter array analysis was used. For each biological replicate, relative amounts of each gene were determined by comparison to the standard curve. The “unknown” samples were then normalized to β-globin, and the copy number in the parental 468GFP cell line was set to 1. Results are presented as fold change relative to control ± SEM. P-values were calculated by first log (base10) transforming the individual normalized ratios, and the normal distribution of each group (468GFP or 468LN) was checked using the Kolmogorov-Smirnov test. Equality of Variances was checked and confirmed using Levene's test, and p-values for copy number change in 468LN relative to 468GFP was calculated using the students t-test. Statistical Analyses were performed using SigmaStat software (Systat Software, Point Richmond, CA, USA).

### Ingenuity Pathway Analysis (IPA)

Gene networks and canonical pathways representing key genes were identified using the curated Ingenuity Pathways Analysis (IPA) database as previously described [4]. Briefly, the data set containing gene identifiers and corresponding fold changes was uploaded into the web-delivered application and each gene identifier was mapped to its corresponding gene object in the Ingenuity Pathways Knowledge Base (IPKB). The functional analysis identified the biological functions and/or diseases that were most significant to the data sets. Fisher's exact test was performed to calculate a p value determining the probability that each biological function and/or disease assigned to the data set was due to chance alone. The data set was mined for significant pathways with the IPA library of canonical pathways and networks were generated by using IPA as graphical representations of the molecular relationships between genes and gene products. The intensity of genes (node) colour in the networks indicates the degree of downregulation (green) or upregulation (red) of gene expression. Nodes are displayed using various shapes that represent the functional class of gene products.

## Results

### Copy Number Analyses Reveal Chromosomal Gains and Losses in These Breast Cancer Cell Lines

**Figure 1:**
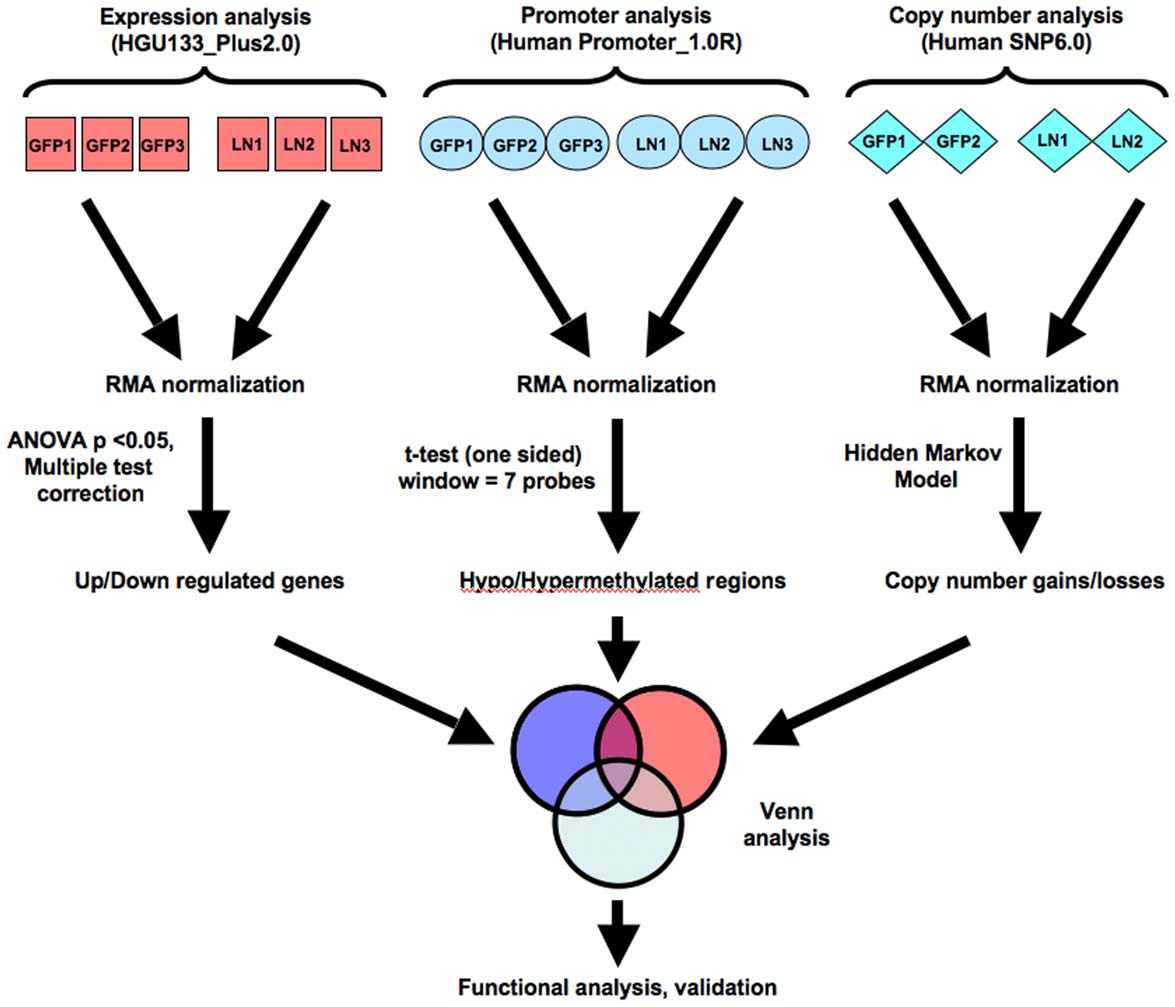
Experimental design for gene expression, promoter methylation and copy number analysis, and data integration. Individual microarrays in replicates (red, light blue or gray boxes for expression, promoter methylation, or copy number variation analysis respectively) were imported into Partek Genomics Suite (PGS) software and background corrected using the RMA algorithm. Genes significantly altered in expression, promoter methylation, or in copy number were then compared using Venn analysis in PGS, and further validated. GFP: MDA-MB-468GFP cells; LN: MDA-MB-468GFP-LN cells; RMA: Robust Multichip Averaging.

**Figure 2:**
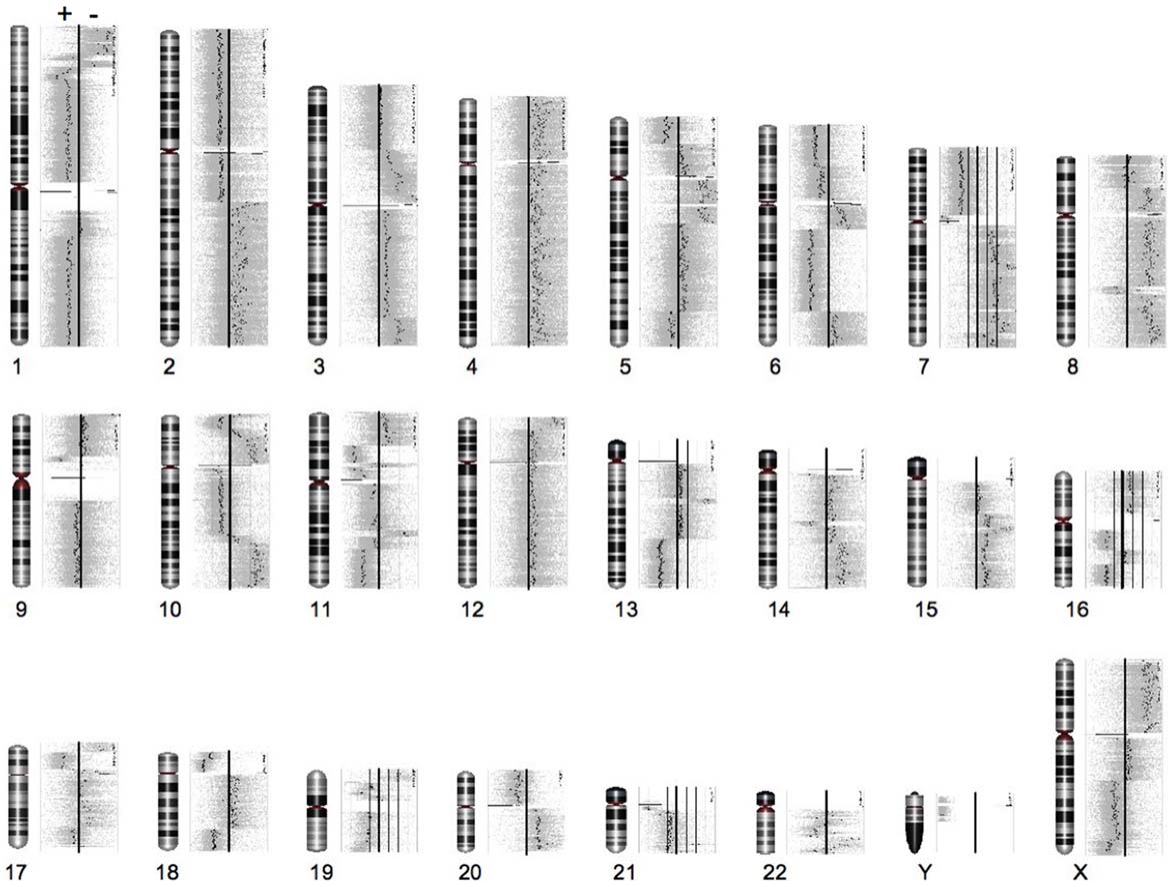
Mapping of genomic DNA copy number variation to individual chromosomes. The thicker vertical bar in the centre of each scan represent the normal diploid number, and the points represent smoothed averages of the probes on the array. Points falling to the right (or left) of the bar indicate regions of copy number gain (or loss, respectively) in 468LN vs 468GFP cells.

We undertook high-resolution whole genome profiling using several Affymetrix microarray platforms to cross reference gene dosage, expression and DNA methylation changes in an isogenic, paired cell lines based on the human MDA-MB-468 breast adenocarcinoma cell line [18], [22], [23]. The cell lines used in the present study include the GFP-transfected parental, poorly metastatic MDA-MB-468GFP (468GFP) cell line and a progeny MDA-MB-468GFP-LN (468LN) cell line variant that is highly tumourigenic and that also maintains a propensity to metastasize in a mouse xenograft models. Previously, we had used classical karyotype analyses to identify complex chromosomal rearrangements that were both shared and unique to each of the two cell lines [1]. Although karyotyping identifies genetically distinct cell clones within a cancer cell population, it is difficult to cross reference karyotype data with data from other whole genome platforms. Therefore, the Affymetrix genome-wide SNP array 6.0 platform was used to provide copy number estimates across the genomes of the 468GFP and 468LN cells. (All copy number microarray data are presented in Tables S2 and S3.) There are more than 1.8 million markers on a single 6.0 array, with half of these being non-polymorphic probes selected for their linear response to copy number and genomic position [24]. Two arrays were probed with duplicate DNA samples from the 468GFP and the 468LN cell lines, with the resultant data imported into and analyzed with the Partek Genomic Suite (Figure 1). When data were mapped to individual chromosomes, we were able to identify approximately 96 large regions of chromosome copy number differences between the 468LN and the 468GFP cells, spanning from 10–100 megabases (Figure 2). A number of these chromosomal changes appeared to reflect the common aberrations we had previously reported by karyotype analysis [1].

**Figure 3:**
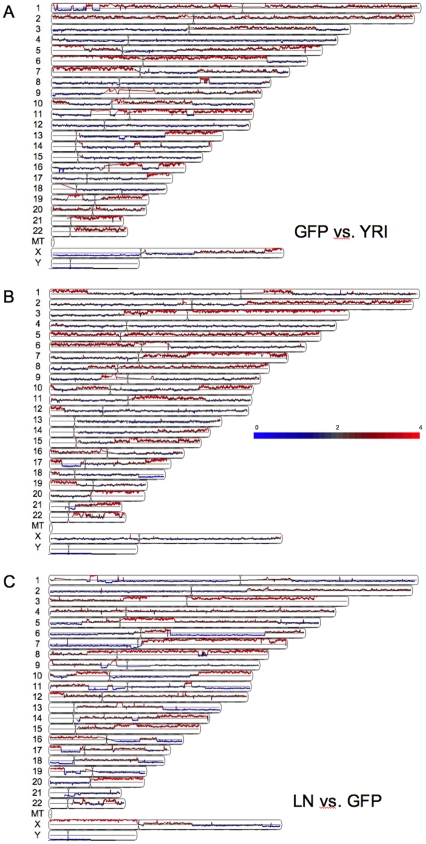
Chromosomal mapping of regions of copy number alteration. Regions appearing increased in copy number are shown in red, and those decreasing in copy number in blue. A: 468GFP samples vs reference Yoruba population (YRI), B: 468LN samples vs YRI reference population and C: 468LN vs 468GFP.

**Figure 4:**
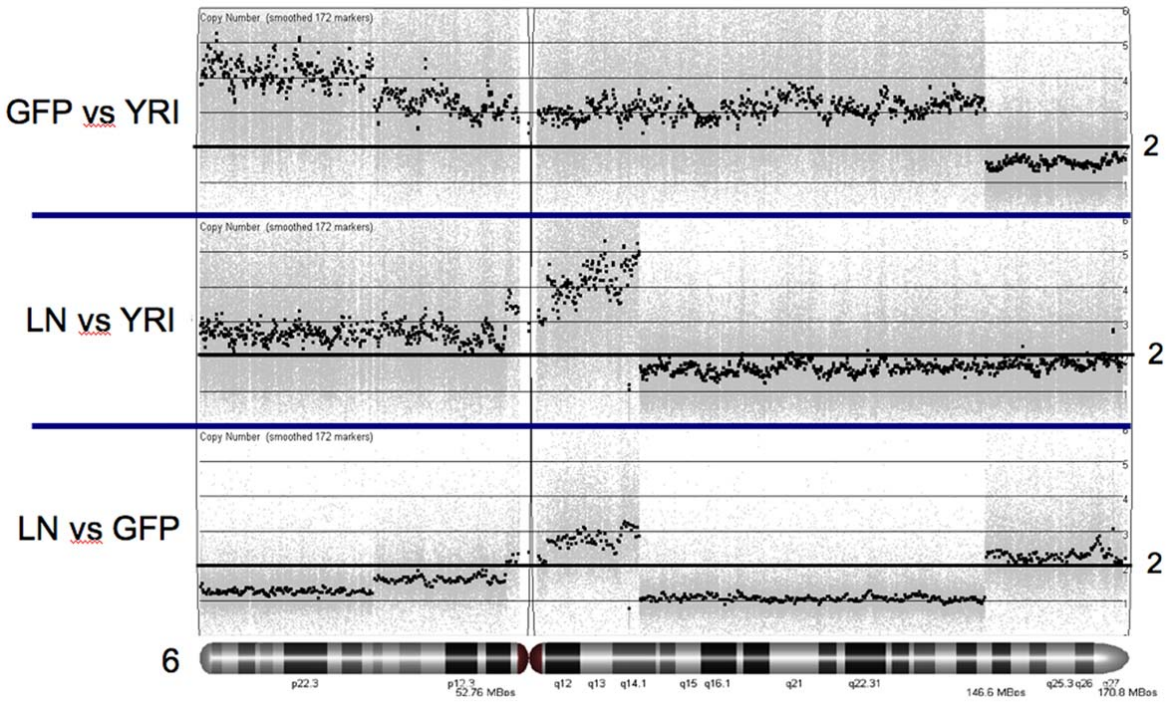
Chromosomal mapping of copy number variations detected in Human SNP 6.0 arrays. As a reference population, we used a subset (60 Yoruba females; YRI) of the 270 samples from the International HapMap Project [52] run on the Affymetrix Human SNP 6.0 array [48]. Copy numbers were normalized to 2 for ease of comparison. Chromosomal locations of regions of significant copy number alteration are shown: 468GFP vs the Yoruba reference population, 468LN vs Yoruba, and 468LN vs 468GFP.

These regional changes in chromosome copy number were particularly obvious when heat maps generated from 468GFP and 468LN DNAs were compared to a normal reference female Yoruba (YRI) population (Ibadan, Nigeria; [25]) from the International HapMap Project (Figure 3). This particular comparator population was chosen because the MDA-MB-468 cell line was originally isolated from the pleural effusion of a 51-year-old African-American female patient with metastatic adenocarcinoma of the breast [22]. As shown in Figure 3, comparisons of probe heat maps from these three genomes (468GFP, 468LN and YRI) showed complex differences among all three genomes. The 468LN genome possessed regions that appeared unchanged from the control population but also displayed regions that differed between 468GFP and 468LN cells. In comparing the 468LN vs 468GFP genomes (Table 1), the median copy number increase was 3.84N (range 2.11 – 47.50), with a median size of an amplified region being 666 kb (range 8 bp–36 Mb), while the median copy number decrease was 1.23N (range 0.07 – 1.92), with a median size of deleted region of 292 kb (range 6 bp–31 Mb). Copy number (averaged across the chromosomal regions; hence the fractional values) and fragment size data specific to each chromosome are also provided in Table 1. Specific examples of these regional changes in copy number can also be observed in the representative context of chromosome 6, by comparing the data across the three genomes (Figure 4). Genome comparisons between 468LN vs 468GFP revealed five defined regions on chromosome 6, ranging from 19.4 to 31.9 Mb, that varied between 1N and 3N in copy number. Comparisons between the cell lines and the control population showed additional variations in these patterns. For example, the 468GFP/YRI comparison showed an apparent proximal 6q (q12–q24) aneuploidy (3N) along with an apparent loss of one copy of 6q24-qter. A different profile was seen with the 468LN/YRI comparison of chromosome 6, where we observed increased ploidy (up to 5N) that was limited to the 6q centromeric region. Comparing these data with karyotype analysis from our previous paper showed that the 468LN line possesses a population of cells having chromosome 6 variants, including a common der(6;7)(q13;q22) translocation [1]. Thus, the 468LN vs 468GFP copy number profiles generated by our analyses represent the cumulative set of chromosomal losses and gains amassed during the evolution of these cell lines, with these changes reflecting the retention, the loss and/or the gain of specific cell clones within the cell line population.

**Table 1:** Association of significantly altered chromosomal regions with copy number variations.

	Copy Number Increase				Copy number decrease			
	region size (bases)		CNV (LN vs GFP)		region size (bases)		CNV (LN vs GFP)	
Chromosome	Median	range	Median	range	Median	range	Median	range
1	581135	1567–9179039	5.8	4.1–14.3	448766	178–4767825	1.4	0.1–1.4
2	19747	651–1754927	4.7	2.7–10.7	852822	142214–1328049	1.4	1.0–1.5
3	1714572	537–28619174	4.7	2.9–19.2	127	NA	0.7	NA
4	8158	366–870275	7.7	2.8–20.1	5060	115–54438	0.5	0.1–1.8
5	341544	382–12325442	3.8	3.2–45.9	820233	522–3962771	1.4	0.1–1.5
6	1345260	3648–4638195	2.9	2.4–3.3	3448507	582–31365262	1.2	0.3–1.4
7	1204223	357–10771567	3.8	2.1–47.5	120894	1198–13880419	0.5	0.1–1.8
8	523419	8–36364716	3.5	2.1–43.6	93012	927–416995	1.4	0.2–1.7
9	263469	41754–1489090	3.6	2.4–13.1	128738	6–20341142	1.0	0.2–1.4
10	1276318	565–18668517	3.5	3.0–6.5	179326	202–4999305	1.4	0.1–1.5
11	318665	4142–4710428	3.2	2.4–13.4	28250	13–15453067	0.7	0.2–1.5
12	257993	5211–4882198	3.6	3.3–19.4	15679	33–191249	0.5	0.1–0.9
13	24321	492–2742074	9.9	4.1–14.6	75230	362–18599472	1.1	0.2–1.3
14	158006	945–14212298	3.1	2.9–29.6	14272	210–3791211	0.3	0.1–1.2
15	1169715	382–20624312	3.8	3.1–39.1	139	NA	0.3	NA
16	433423	35–14073681	5.0	2.7–20.0	2976128	16026–15420894	1.2	0.6–1.3
17	496178	335–7515169	4.1	2.8–22.3	692738	820–11936223	1.2	0.2–1.3
18	7296	1105–47923	8.1	5.6–21.9	5199602	255–15390865	1.3	0.9–1.4
19	9639422	NA	3.2	NA	704502	4741–3695496	1.4	0.2–1.5
20	515523	1816–20739653	3.7	3.3–5.3	148965	40585–514274	1.3	1.0–1.9
21	201578	NA	3.3	NA	59842	11145–4587497	0.5	0.3–1.1
22	421267	6473–7039940	3.7	3.1–16.8	214143	7038–943379	1.3	1.1–1.5
X	1766175	413–14118291	4.0	3.0–9.9	123480	253–5781886	1.4	0.1–1.9

### Multi-Array Analyses Define Gene Subsets Related by Expression, Methylation and Copy Number

**Figure 5:**
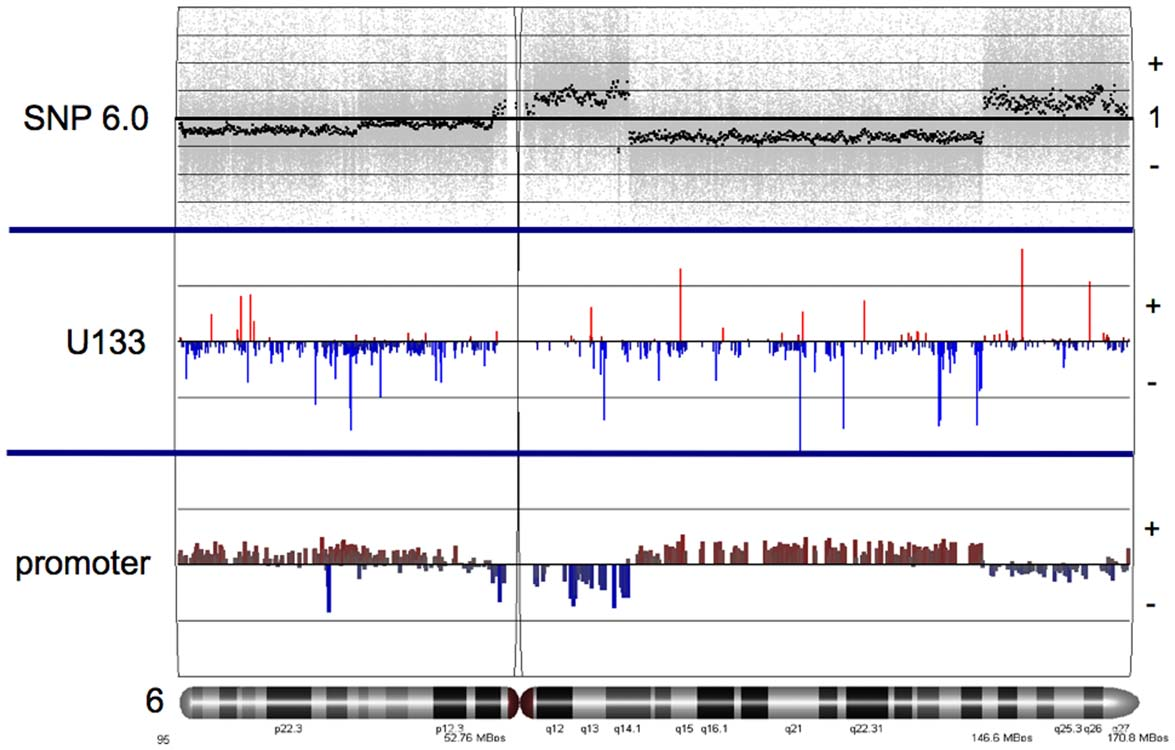
Representative multi-array alignment of data from the SNP/copy number variation (top), gene expression (middle) and promoter methylation (bottom) are shown for chromosome 6. In the upper panel 1 indicates relative copy number for 468GFP:468LN. For the U133 array data, plus represents significantly upregulated genes, and minus represents downregulated genes. For the promoter array data, plus represents significant regions of hypermethylation, and minus represents hypomethylated regions. Also, the promoter array data is presented on a log_2_ scale, while for expression data, the height of the bars representing individual genes is proportional to the expression fold change. Similar multi-array data specific for all other chromosomes are provided in Figure S1.

We next assessed the functional significance of these complex rearrangements and copy number changes in the context of gene expression and epigenetic (DNA methylation) profiles. Gene expression and promoter methylation patterns were determined using Affymetrix HGU133_Plus_2 arrays and Human Promoter 1.0R arrays respectively, and two approaches were taken to cross-reference these data sets. (Expression microarray data are presented in Tables S4 and S5; methylation microarray data are published in [4].) First, we used Partek Genomic Suite to directly align these three data sets in the context of each chromosome. Using chromosome 6 again as an example (Figure 5), we compared the 468LN and 468GFP profiles and observed direct correlations between the regional blocks related to copy number (Figure 4) and apparent ‘blocks’ of DNA methylation along the chromosome. Interestingly, we observed that a relative loss in copy number correlated with a gain in hypermethylation, while increases in copy number correlated with losses in DNA methylation (see 6q in Figure 5). Expression data for individual genes on the whole do not appear to consistently correlate with these apparent regional changes in copy number and DNA methylation. Similar multi-array alignment data specific for all other chromosomes are provided in Figure S1.

**Figure 6:**
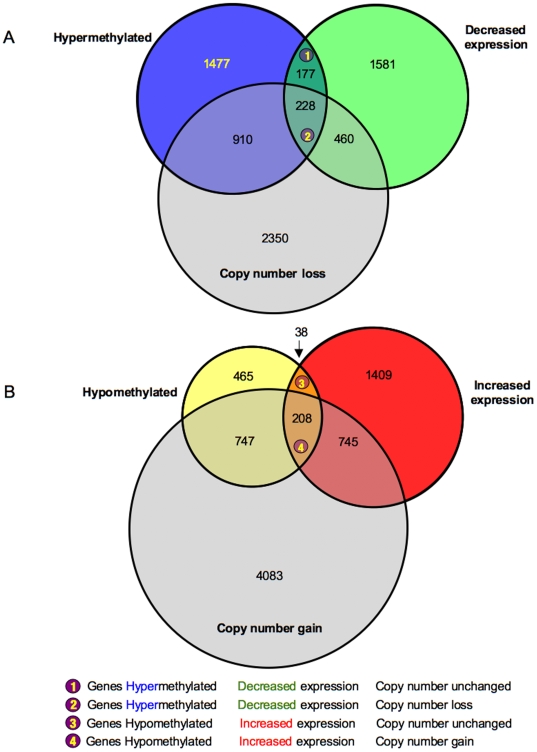
Proportional Venn analysis of significantly changed gene regions as determined by multiarray analyses. A: Venn analysis of genes predicted to be hypermethylated, decreased in expression, and showing a loss in copy number; specific regions of functional overlap are indicated (1 or 2). B: Venn analysis of genes predicted to be hypomethylated, increased in expression, and showing a gain in copy number; specific regions of functional overlap are indicated (3 or 4). The diameter of each circle is proportional to the number of genes identified by that specific array analysis.

Our second approach involved importing probe set IDs from each of the three microarray platforms and comparing these sets using the Genespring Venn Analysis tool. Figure 6 shows cross-referenced probe sets that were hypermethylated, decreased in expression, and decreased in copy number (Figure 6A) as well as probe sets that were hypomethylated, increased in expression and with a gain in copy number (Figure 6B). While platform-specific gene sets ranged from 1400 to 5700 genes, these Venn diagram analyses allowed us to identify specific gene subsets (38 to 228 genes; regions 1,2,3,4 in Figure 6) with specific relationships between expression, methylation status and copy number. As shown in Figure 6A, region (1) defined 177 genes with a functional relationship between hypermethylation and decreased expression that is independent of copy number, while region (2) defined 228 genes with a functional relationship between hypermethylation and decreased expression that is dependent of copy number. Similar gene sets identified in relation to gene expression and hypomethylation (regions 3,4) are shown in Figure 6b. (Complete gene lists for regions 1–4 are presented in Tables S6–S9.)

### Ingenuity Pathways Analysis (IPA) of Differentially Methylated/Expressed/Copy Number Variant Gene Targets

**Figure 7:**
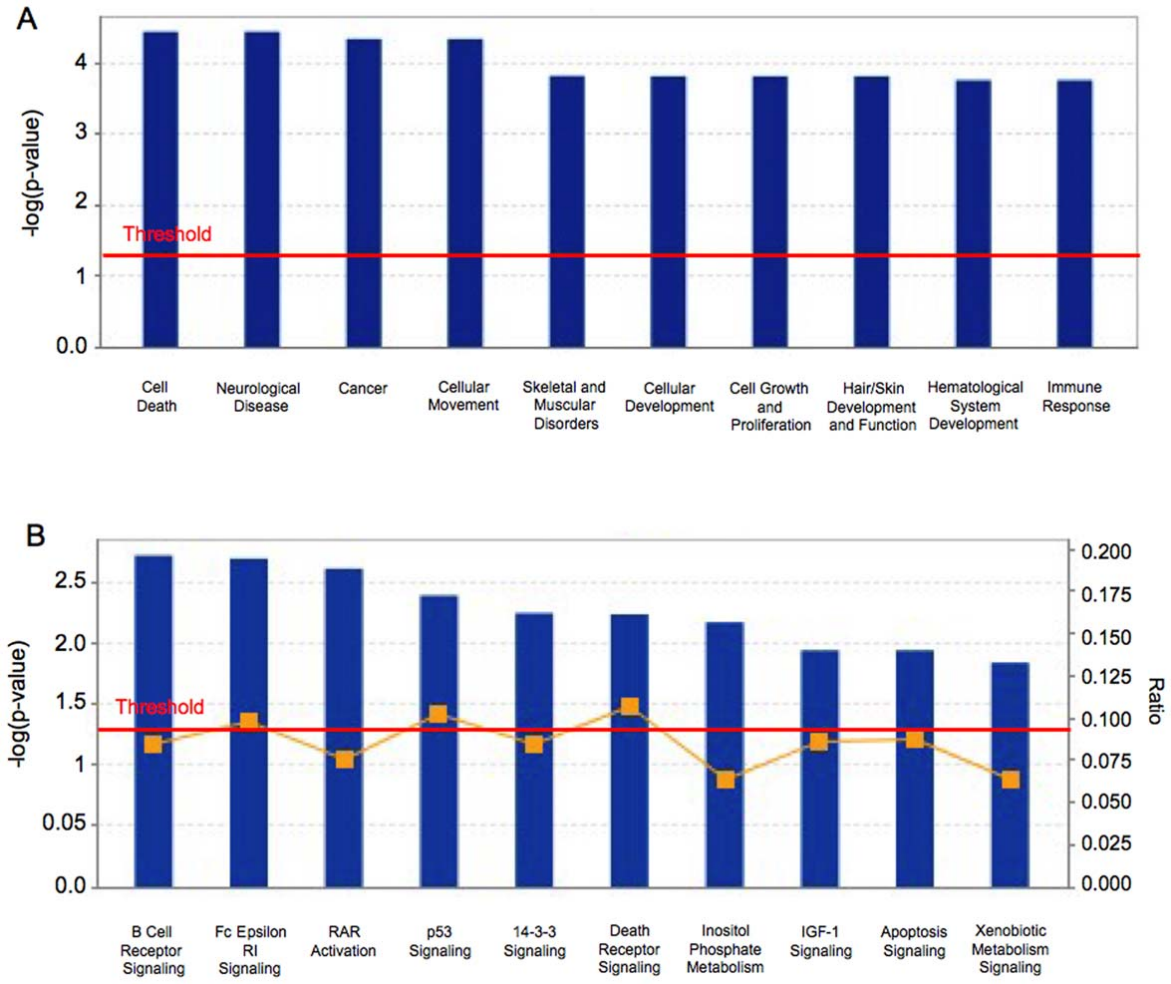
Ingenuity Pathway analyses. A: Top functional categories and B: canonical pathways from our data set based on significance.

**Figure 8:**
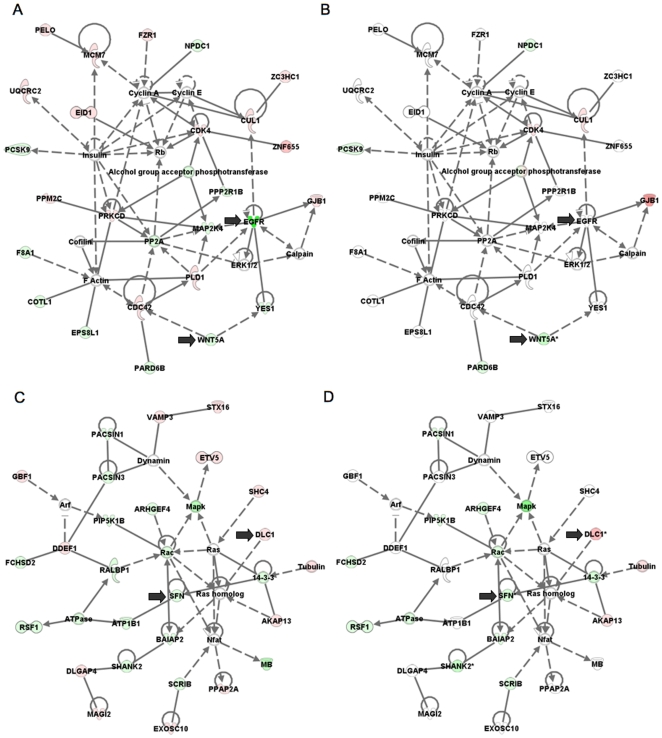
Network analysis was performed to provide a graphical representation of genes having known biological relationships. The EGFR and Mapk networks presented are shown in duplicate, with (A and C), displaying genes comprising the four insecting subregions shown in Figure 6 (regions 1,2,3 and 4) and (B and D) displaying genes comprising the two intersecting subregions (regions 1 and 3) that have a methylation/expression status that is independent of copy number. Green icons indicate downregulated genes and red indicates upregulated genes. The arrows indicate selected genes that have a variable methylation status that is dependent on copy number status.

Annotated gene lists were created with the 405 significantly hypermethylated (*P*<0.05; Tables S6, S7) and 246 hypomethylated (*P*<0.05; Tables S8, S9) gene targets identified within the four intersected regions that are shown in Figure 6. We used IPA to investigate the biological relevance of the observed genome-wide methylation changes by categorizing our data set into biological functions and/or diseases (Figure 7A). These broad categories each involved genes having roles in cell death, cell signalling, cellular movement, cancer and other functional categories. We also searched the gene lists and identified a number of significant canonical pathways from the IPA library, including pathways involved in B cell receptor, p53 and 14-3-3 signalling pathways (Figure 7B). Network analysis was also performed to provide a graphical representation of gene having known biological relationships. The top five networks were related to the EGFR, TGFβ1, NFkβ, ERK and the Mapk genes, with each network involving 30–40 hypermethylation/downregulation and hypomethylation/upregulation events (Figure 8; A,C). We also performed network analyses on the two intersecting regions (1 and 3) that displayed genes that were independent of copy number (Figure 8; B,D) and compared these results with the ‘complete’ networks derived from genes in all four regions. The level of expression of a number of genes in these networks (e.g the EGFR and Mapk networks; Figure 8, arrows), were predicted to be dependent on copy number as shown by the green (downregulated) and red (upregulated) shading of these gene icons in the networks.

### Confirmation of DNA Methylation, Expression and Copy Number Status

**Figure 9:**
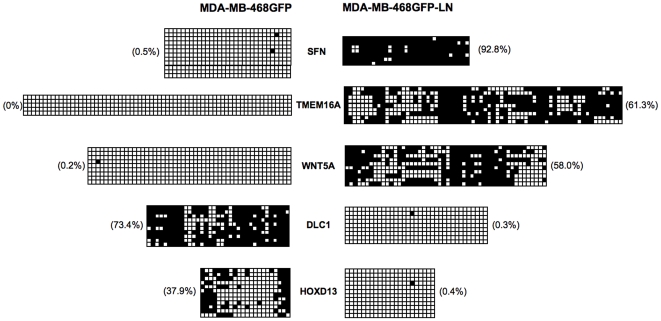
Sodium bisulfite sequencing, gene expression and copy number. Sodium bisulfite sequencing of representative genes detected with aberrant methylation with (or without) a concomitant change in copy number. Each square represents a CpG (open square: unmethylated; closed square: methylated). Each row of squares one cloned PCR sequence across the gene promoter (5–20 clones were sequenced per gene). Percentages indicate degree of methylation at each gene locus.

**Figure 10:**
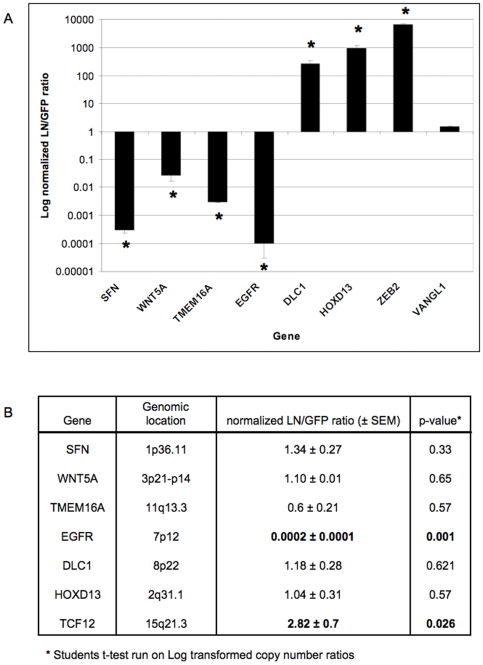
Expression and copy number analyses. A: Quantitative real time RT-PCR expression data for each of these genes, including EGFR. Scale of the y-axis is log_10_ of the fold change. B: Determination of copy number by quantitative real time PCR (qRT-PCR). Primers spanning an exon of the gene of interest were designed using ExonPrimer software, and qRT-PCR performed using 15 ng genomic DNA from 3 biological replicates each of LN and GFP DNA as template. Shown is the LN/GFP ratio, ± standard error of the mean (SEM). Data were normalized to β-globin as a reference gene. A p-value <0.05 indicates that mean normalized LN copy numbers in LN triplicates were significantly different from those in GFP.

We undertook sodium bisulfite analysis (Figure 9) to confirm the DNA methylation status at several gene loci putatively identified as being differentially methylated. Three of these genes (SFN, TMEM16A and WNT5A) showed dramatic hypermethylation in the 468LN cells (up to 92% CpG methylation), in contrast with <1% CpG methylation of the same promoter regions in the 468GFP cells, confirming the promoter microarray *in silico* analyses. In contrast, DLC1 and HOXD13 were markedly hypomethylated in the 468LN cells, in comparison with the 468GFP cells (<1% versus 38–73% methylation). Subsequent qRT-PCR experiments (Figure 10A) showed that expression of the hypermethylated SFN, TMEM16A and WNT5A genes was decreased in 468LN cells, whereas the hypomethylated DLC1 and HOXD13 genes were significantly increased relative to the 468GFP cells. VANGL1 expression did not change. In addition, we have previously shown that EGFR was significantly hypermethylated in 468LN cells (47% vs <1%) [1].

Copy number status for these genes as determined by SNP6.0 arrays was confirmed by qRT-PCR, using normal human genomic DNA as an external standard. After normalization to the internal standard (β-globin), a student's t-test was used to compare the copy number of each gene in 468LN cells vs that in 468GFP cells. The results are summarized in Figure 10B. These experiments showed that as predicted, copy number of SFN, WNT5A and TMEM16A in 468LN cells (subregion 1; Figure 6) did not significantly differ from that in 468GFP (p-value 0.33, 0.65, and 0.57, respectively). Similar results regarding similar copy number were confirmed for DLC1 and HOXD13 (subregion 3, Figure 6; p value = 0.66 and 0.57, respectively). TCF12 increased in copy number (2.8 fold; p<0.03). In contrast, EGFR (subregion 2, Figure 6) copy number was significantly decreased in LN vs GFP cells (p = 0.001).

### Reversal of DNA Methylation Status

**Figure 11:**
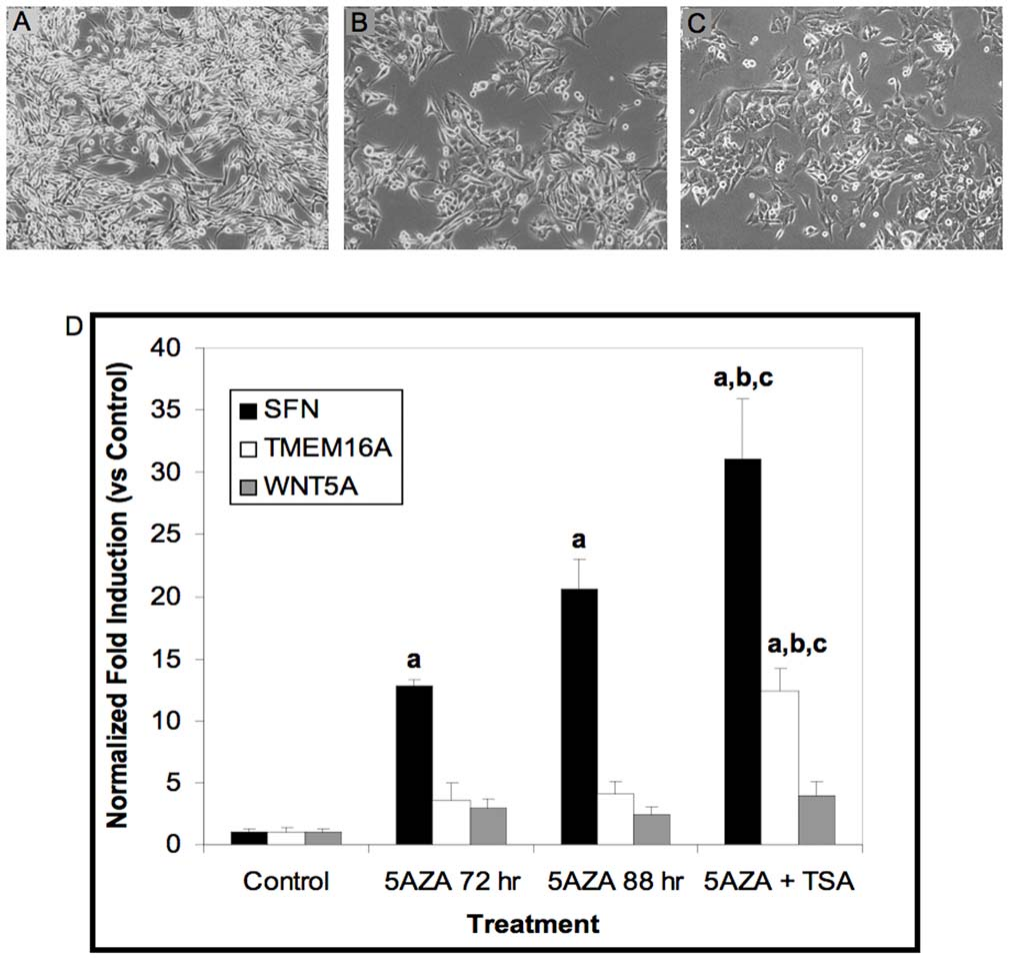
Exposure to 5-aza-2-deoxycytidine and Trichostatin A. A–C: 468LN cells were cultured for 7 days in A: the absence or B: the presence of 5-aza-2-deoxycytidine followed by C: an additional 16 h exposure to the histone deacetylase inhibitor Trichostatin A (TSA). D: Induction of epigenetically down-regulated genes. 468LN cells were cultured in the absence (Control) or presence of 5-aza-2-deoxycytidine (5AZA) for 72 hours (5AZA 72 hr), 88 hours (5AZA 88 hr), or for 72 hours followed by the addition of Trichostatin A (TSA) for 16 hours (5AZA+TSA). Total RNA was extracted and qRT-PCR performed as described in the text. Significant group differences were determined using ANOVA followed by the Student-Newman-Keuls multiple comparison procedure. **a**: significantly different (p<0.05) vs Control. **b**: significantly different vs 5AZA 72 hr. **c**: significantly different (p<0.05) vs 5AZA 88 hr.

Finally, we asked whether the altered methylation patterns seen in the 468LN cells could be reversed through the use of ‘epigenetic drugs’ that can restore the normal epigenetic patterns (and expression patterns) of these genes. 468LN cells were cultured for 72 hours in the presence of 10 µM 5-aza-2′-deoxycytidine (5-azaC) followed by an additional 16h exposure to the histone deacetylase inhibitor Trichostatin A (TSA; 50 nM). We observed phenotypic changes in the treated 468LN cells in that they changed from a mesenchymal to a more epithelial phenotype that was more similar to the parental 468GFP cell line ([18], Figure 11A–C). Furthermore, expression analysis using RT-PCR showed that 5-azaC in both the presence (or absence) of TSA could re-initiate expression of the stratifin (SFN) gene, which we had shown was down regulated epigenetically in the 468LN cells (Figures 9, 10A). As well, TMEM16A expression was re-initiated after exposure to 5-azaC+TSA while WNT5A trended toward re-expression relative to control cells. These results confirm that DNA methylation changes identified from multi-array technology may be used to identify genes that are potential targets for epigenetic therapy.

## Discussion

Whole genome scanning technologies are providing new opportunities to identify gene profiles related to breast cancer progression. While a number of studies have generated genetic signatures of metastasis risk [7], [26], recurrence and clinical outcome [5], [27] and have identified candidates for targeted therapy [9], [10], [28], the epigenetic profiles in normal or cancer cells and in tumours are less well characterized [4], [29]. Such epigenetic signatures encompass the heritable modifications that do not change the DNA sequence but rather provide ‘extra’ layers of control that regulate chromatin organization and gene expression [30], [31]. As well, new targeted epigenetic therapies can potentially be developed to identify and correct epigenetic alterations and restore normal gene expression patterns that are dependent on their epigenetic (i.e. DNA methylation) signature [32], [33].

The advent of these various whole genome platforms allows new opportunities to cross-reference these vast data sets to better understand tumour progression. Several groups have addressed allelic imbalance in tumourigenesis and undertaken comparative studies to integrate copy number analysis with gene expression patterns in the context of breast [34], lymphoma [35] and glioblastoma [36], [37]. Only a few reports have integrated global cancer-related changes in DNA methylation, genomic imbalance and gene expression, most notably in the context of osteosarcoma [17] and in the development of the SIGMA cancer genome database [38].

In this present report we address the relationship between multiple genomic parameters associated with breast cancer metastasis and progression, by hypothesizing that the complex chromosomal alterations in cancer cells may complicate the interpretation of the promoter-specific methylation events responsible for gene-specific expression changes. Our multi-platform microarray approach simultaneously cross-referenced data sets related to gene copy number, epigenetic (DNA methylation) and gene expression patterns in a paired set of MDA-MB-468 breast adenocarcinoma-derived cell lines that provided a model for tumour progression. While other reports have combined various platforms to integrate analysis of high-resolution microarray profiles [17], [38], we used platforms that share the common Affymetrix technology base. This permitted a robustness of design and economy that allowed standardized bioinformatic assessment and cross-referencing of the data sets, using a single software bioinformatics package (Partek Genomic Suite) to import, analyze and cross-reference raw data from the various microarray platforms.

Our initial copy number analyses revealed complex chromosomal rearrangements with approximately 96 large regions of chromosome copy number differences in the 468LN versus the 468GFP cells, ranging up to 100 megabases (Figure 2). While some of these chromosomal changes appeared to reflect the common aberrations we previously reported by karyotype analysis [1], direct comparisons cannot easily be made, since karyotype analyses are described as the frequency of chromosomal changes on a cell to cell basis, while microarray data are generated from the mixture of DNAs from the multiple cell clones that populate the cell line. Hence, validation of individual gene targets is essential to confirm their functional relevance to the cancer cell phenotype. Since normal cells were not available from the original host patient from whom the cell lines were derived, we performed in silico comparisons with a reference population generated by the International HapMap project (Figure 4). These analyses identified additional differences between the cell lines and the normal population and allowed us to better relate the significance of these chromosomal changes to variable DNA methylation patterns, as described below.

**Figure 12:**
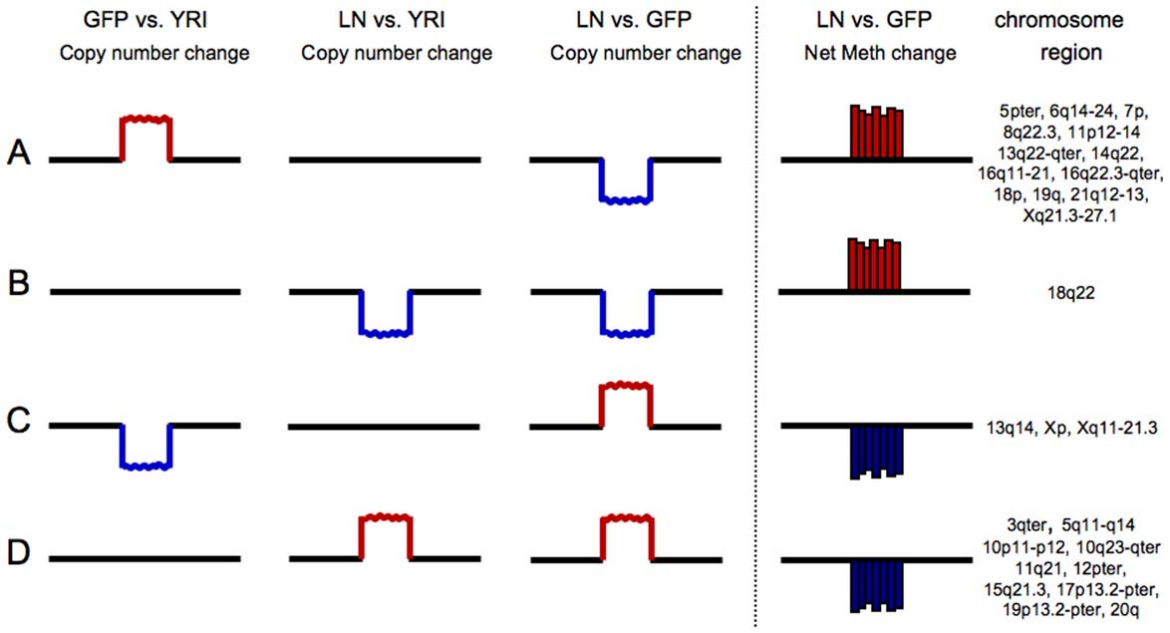
Four scenarios (A–D) are presented to explain the relationship between copy number and DNA methylation. Heat map analyses (Figure 3) revealed regional changes in copy number that reflected chromosomal aberrations in comparisons between the 468GFP and 468LN cells and the reference YRI population. These changes can be interpreted in the context of net gains (A,B) or losses (C,D) in DNA methylation. Specific chromosomal regions displaying these additive events are presented in the final column of this Figure.

The multi-array comparisons that were undertaken involved the complementary approaches of direct alignment of the data sets in the context of each chromosome (Figure 5A and Figure S1), as well as comparison of the data sets using the Genespring Venn Analysis tool (Figure 6). It was immediately apparent from our chromosome-based analyses that there were direct correlations between regional blocks related to copy number and apparent ‘blocks’ of DNA methylation along each of the chromosomes. Many of the relative losses in copy number correlated with an apparent gain in hypermethylation, while increases in copy number tended to correlate with losses in DNA methylation, again in the 468LN cells compared to the parental 468GFP line. These data are supportive of previous reports describing lower levels of DNA methylation in tumours relative to control DNA [39], with this loss of methylation mainly due to hypomethylation of repetitive DNA sequences and demethylation of coding regions and introns [40]. Furthermore, levels of genomic DNA hypomethylation increase as the cancer progresses from a benign proliferation of cells to an invasive cancer [41], which may explain some of the regional methylation differences we observe in the 468LN cells. Comparisons between cell line DNAs and the normal reference DNA provide further interpretation of the ‘additive’ nature of these copy number changes and their relationship to DNA methylation. As shown in Figure 12, the additive copy number differences we observed can be considered relative to specific changes in each of the cell lines relative to normal cells and points to the progressive nature of these genomic changes (Normal>468GFP>468LN), some of which contribute to gene expression losses and gains mediated through aberrant DNA methylation events (for example, the loss of a 7p isochromosome present in 468GFP cells and absent in the derived 478LN cells [1].

In addition to the chromosome-based analyses, Venn Analysis (Figure 6) provided the next level of analysis to sort and prioritize functionally relevant genes in this model system. Our integrative approach allowed us to refine the epigenetic signature that encompassed 2792 hyper- and 1478 hypomethylated events down to ∼650 variably-methylated gene promoter regions. Ingenuity Pathway Analysis further categorized our data set into functional categories and networks (Figure 7) from which high priority gene targets could be validated for their DNA methylation, expression and copy number status. The methylation and expression status of five of these targets is shown in Figures 9 and 10A, confirming the prediction of hypermethylation (and decreased expression) of SFN, TMEM16A and WNT5A and the hypomethylation (and increased expression) of DLC1 and HOXD13 in the 468LN cells. Furthermore, we used qRT-PCR to confirm that these correlations between the methylation and gene expression status of these genes was independent of copy number, as predicted by the assignment of these genes to Venn subregions 1 (SFN, TMEM16A, WNT5A) and 3 (DLC1, HOXD13) as shown in Figure 6.

Finally, we addressed the utility of microarray technologies to identify potential targets for epigenetic-based therapies to restore normal gene expression patterns. The stratifin gene (SFN), which has a hypermethylated promoter and displays minimal expression in the 468LN cells, was chosen as the target gene for these experiments using the demethylating agent 5-azaC and the histone deacetylase inhibitor TSA [42]. Stratifin (14-3-3σ) is a member of the 14-3-3 gene family that regulates numerous cell pathways relevant to breast and prostate cancer including cell cycle arrest, signal transduction, apoptosis and proliferation [43], [44], [45]. We identified stratifin in our experiments through its relationship with genes focussed in the ras/Mapk pathway identified by Ingenuity Pathway Analysis (Figure 8), and we subsequently confirmed its hypermethylated status and loss of expression in 468LN cells (Figure 9, 10A). We observed phenotypic changes in the 5-azaC/TSA treated 468LN cells in that they changed from a mesenchymal to a more epithelial phenotype that was more similar to the parental 468GFP cell line (Figure 11A–C). Furthermore, expression analysis using RT-PCR showed that 5-azaC, in both the presence and absence of TSA, could re-initiate expression of SFN while TMEM16a was re-expressed significantly only after the exposure to both drugs. Our data suggest that epigenetic changes cross-referenced with other multi-array whole genome technologies can identify genes as potential targets for epigenetic therapy in the context of multiple genomic characteristics (gene copy number, DNA methylation and expression), and would likely provide more accurate prognostic and predictive assessment than assessing single genes, or single platforms alone [46], particularly when these changes are cross-referenced with patient tumour material.

Our multi-platform approach refines the epigenetic signatures in breast cancer metastasis in the context of gene-specific functional changes in gene expression and copy number. As well, we show that this approach that cross-references multiple whole-genome data sets, also can isolate and identify targets that are altered by copy number (but are not epigenetically modified) and more precisely map functionally important epigenetic signatures associated with cancer progression. Our approach provides enhanced opportunities, particularly in the context of patient tumour material, to identify therapeutic targets for breast cancer treatment that are epigenetically regulated by alterations in DNA methylation.

## Supporting Information

Figure S1 Multi-platform integrative analysis of copy number, expression, and promoter array data.
(2.06 MB PDF)

Table S1 PCR Primer List.
(0.03 MB PDF)

Table S2 Regions showing an increase in copy number, (468LN relative to 468GFP). Included are associated genes contained within the regions.
(0.07 MB PDF)

Table S3 Regions showing a decrease in copy number, (468LN relative to 468GFP). Included are associated genes contained within the regions.
(0.05 MB PDF)

Table S4 Genes significantly increased in expression, 468LN vs 468GFP. Expression microarray (HGU133 Plus_2) data were prefiltered to remove genes changing less than 2 fold, and an ANOVA was run to determine significant (p<0.05) changers. A multiple testing correction using the algorithm of Benjamini and Hochberg was used to reduce the false discovery rate.
(0.46 MB PDF)

Table S5 Genes significantly decreased in expression, 468LN vs 468GFP. Expression microarray (HGU133 Plus_2) data were prefiltered to remove genes changing less than 2 fold, and an ANOVA was run to determine significant (p<0.05) changers. A multiple testing correction using the algorithm of Benjamini and Hochberg was used to reduce the false discovery rate.
(0.51 MB PDF)

Table S6 Genes Hypermethylated AND Decreased in expression, no change in copy number (Venn region 1).
(0.05 MB PDF)

Table S7 Genes Hypermethylated AND Decreased in expression, loss in copy number (Venn region 2).
(0.07 MB PDF)

Table S8 Genes Hypomethylated AND Increased in expression, no change in copy number (Venn region 3).
(0.06 MB PDF)

Table S9 Genes Hypomethylated AND Increased in expression, gain in copy number (Venn region 4).
(0.07 MB PDF)
