# Supplementary figures and images for: Multi-Platform Whole-Genome Microarray Analyses Refine the Epigenetic Signature of Breast Cancer Metastasis with Gene Expression and Copy Number
Source: PLoS One. 2010 Jan 13;5(1):e8665. doi: 10.1371/journal.pone.0008665 (PMC2801616; doi:10.1371/journal.pone.0008665)

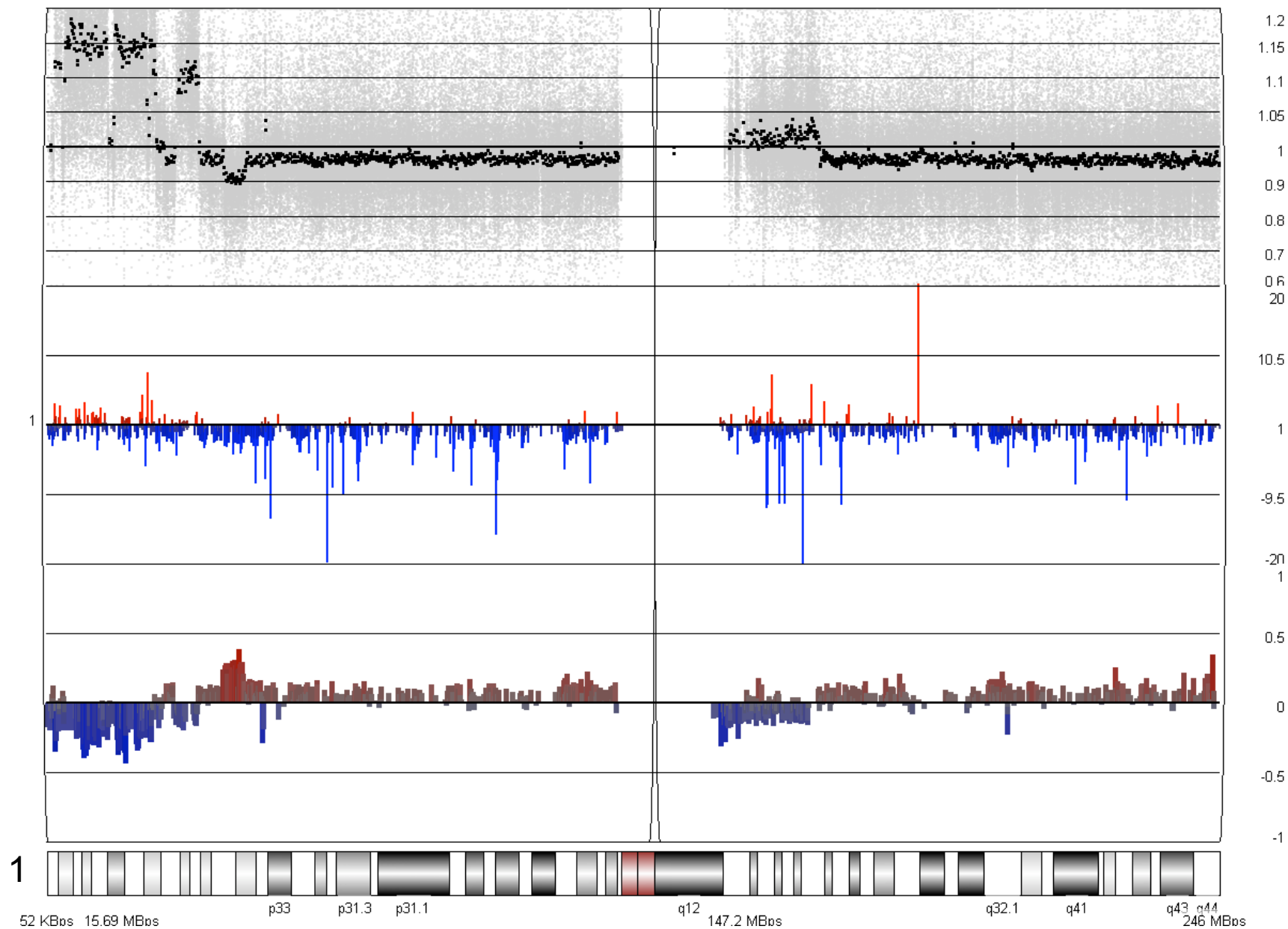

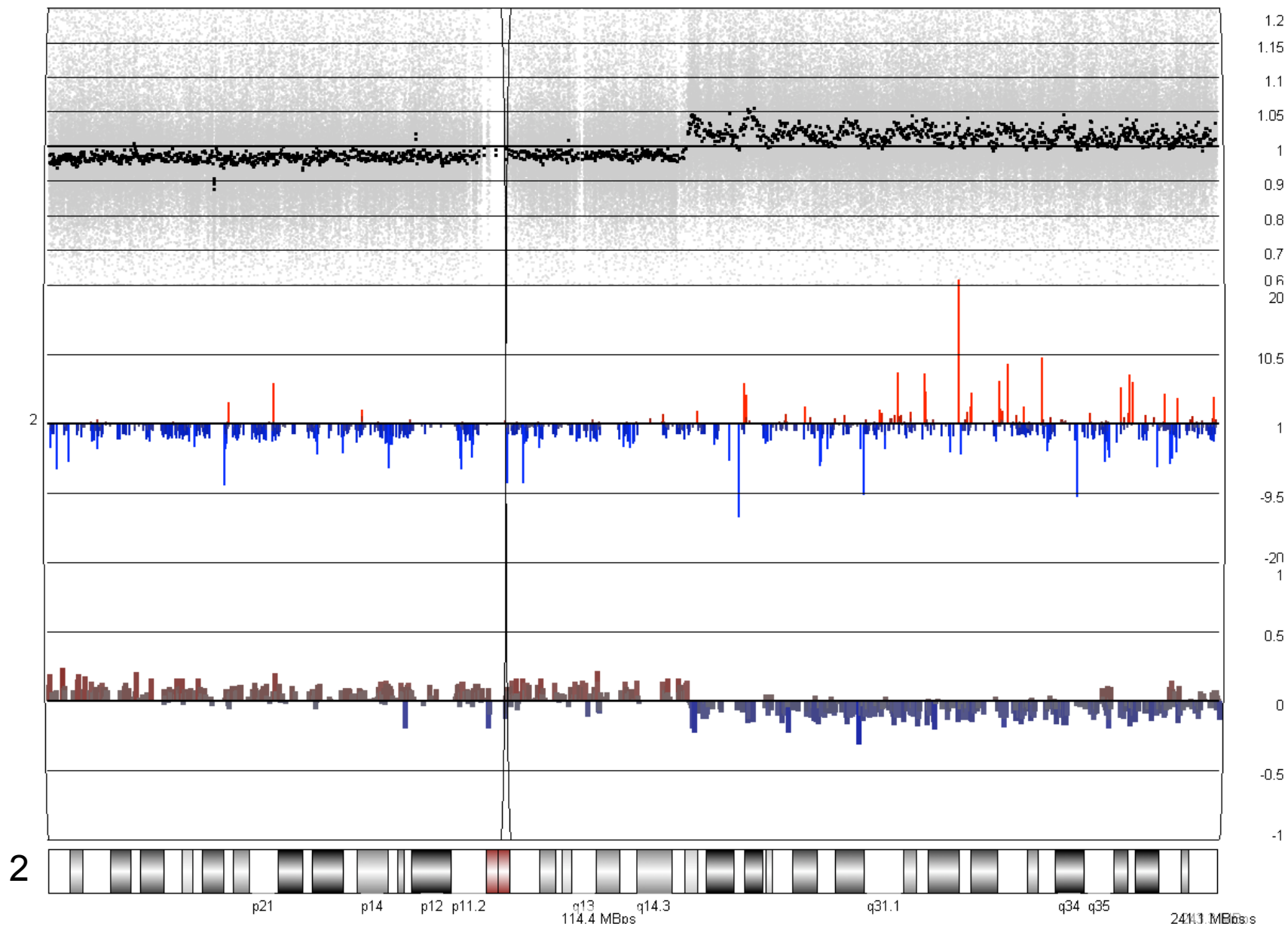

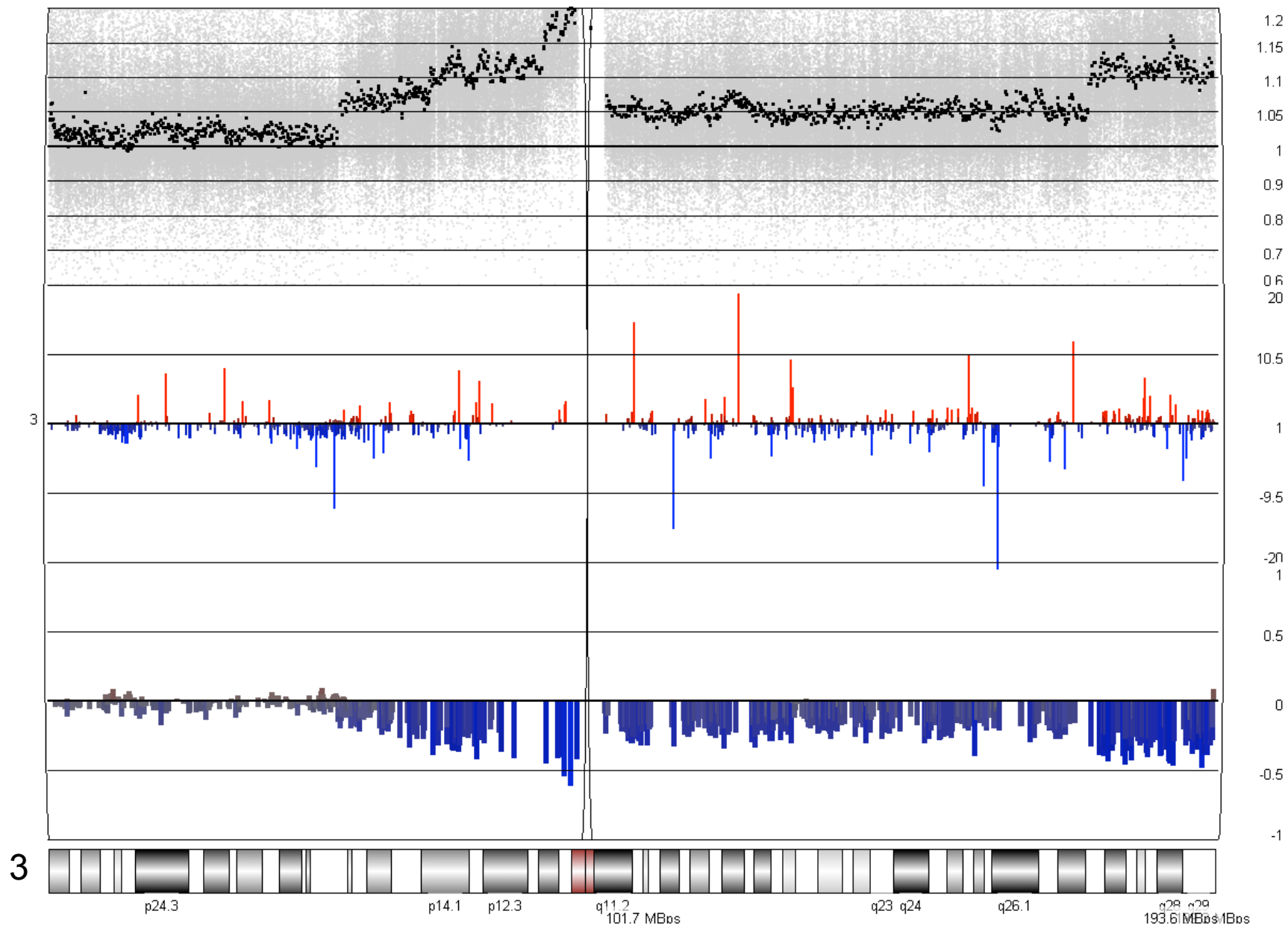

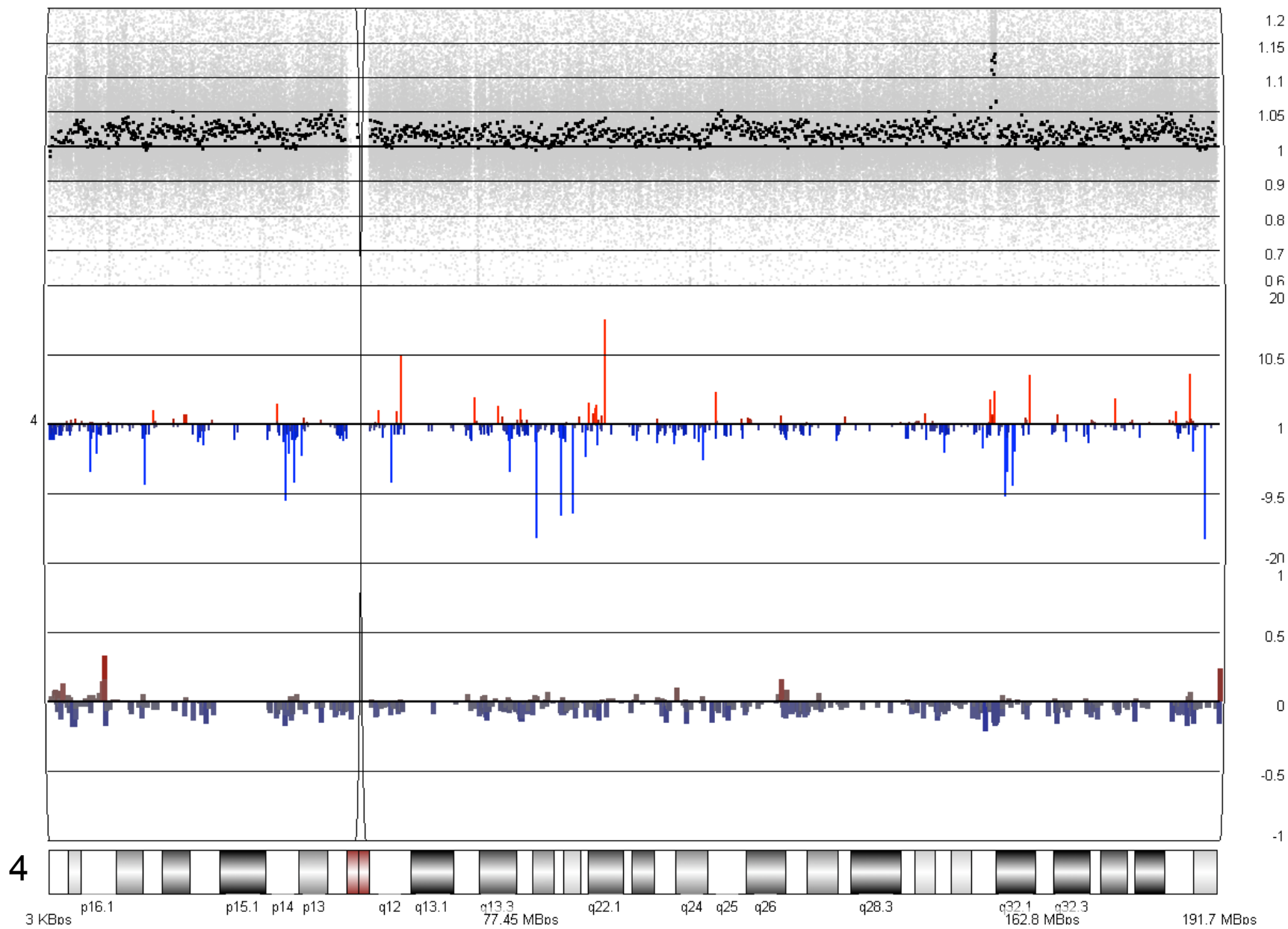

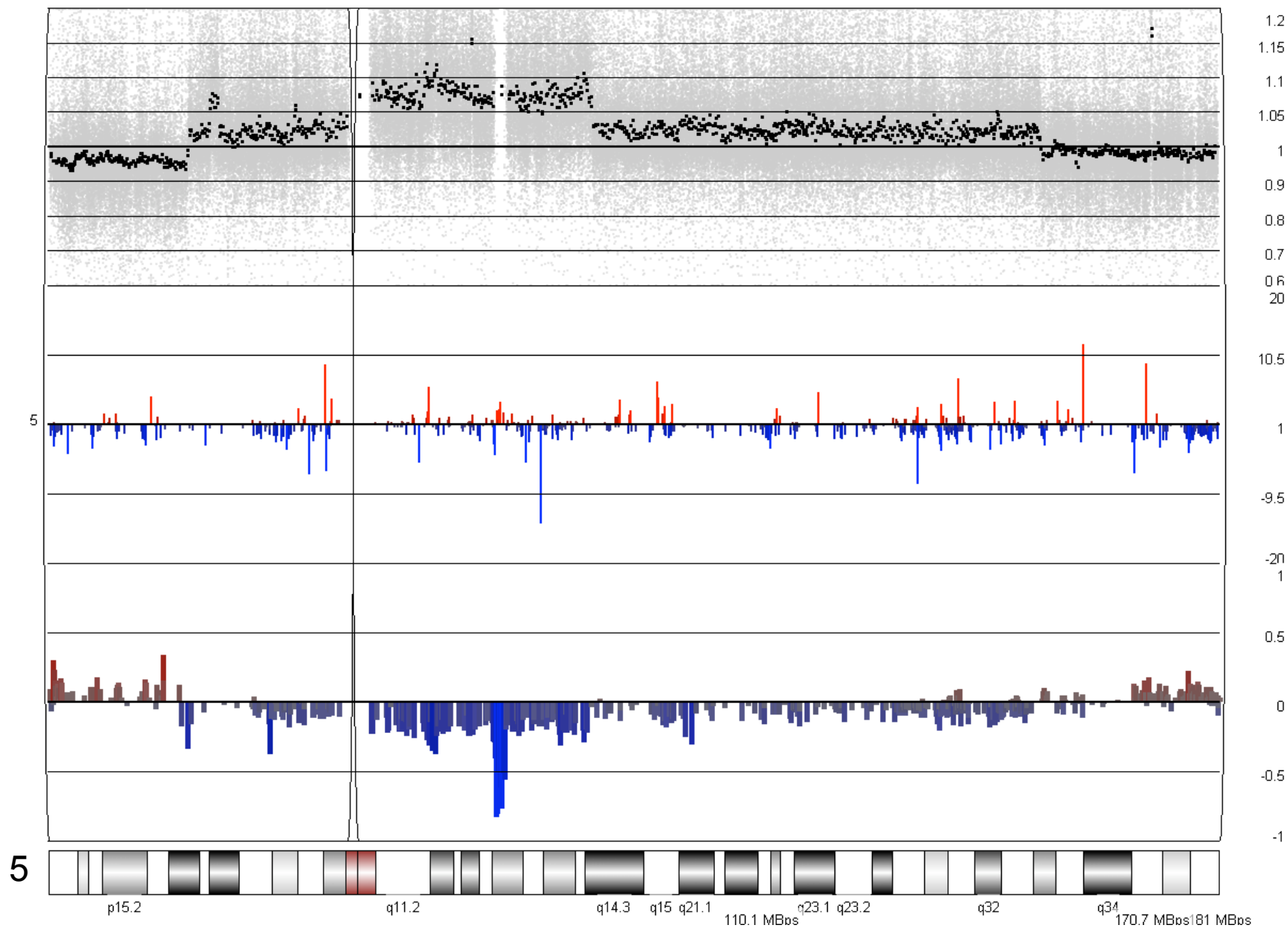

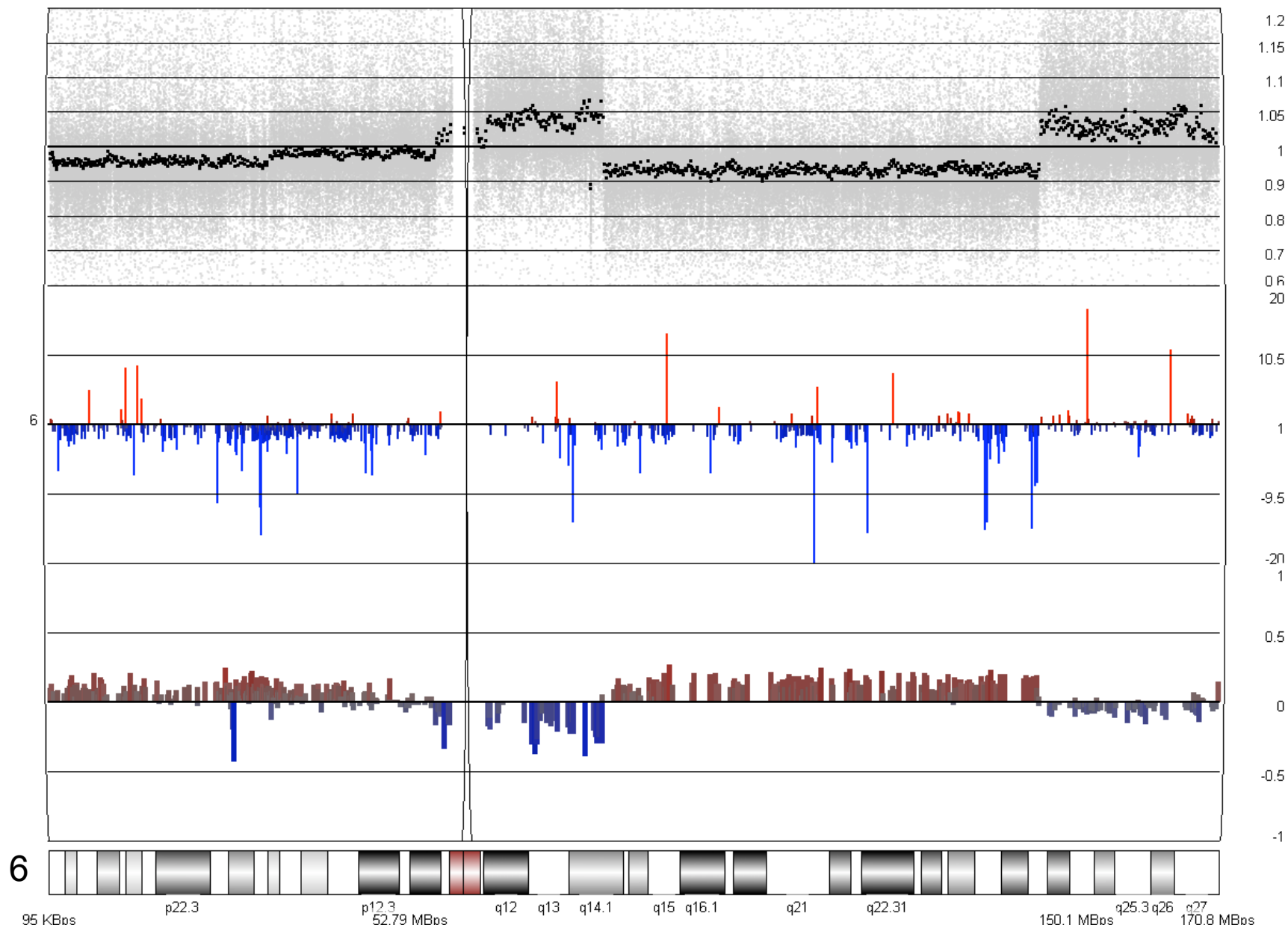

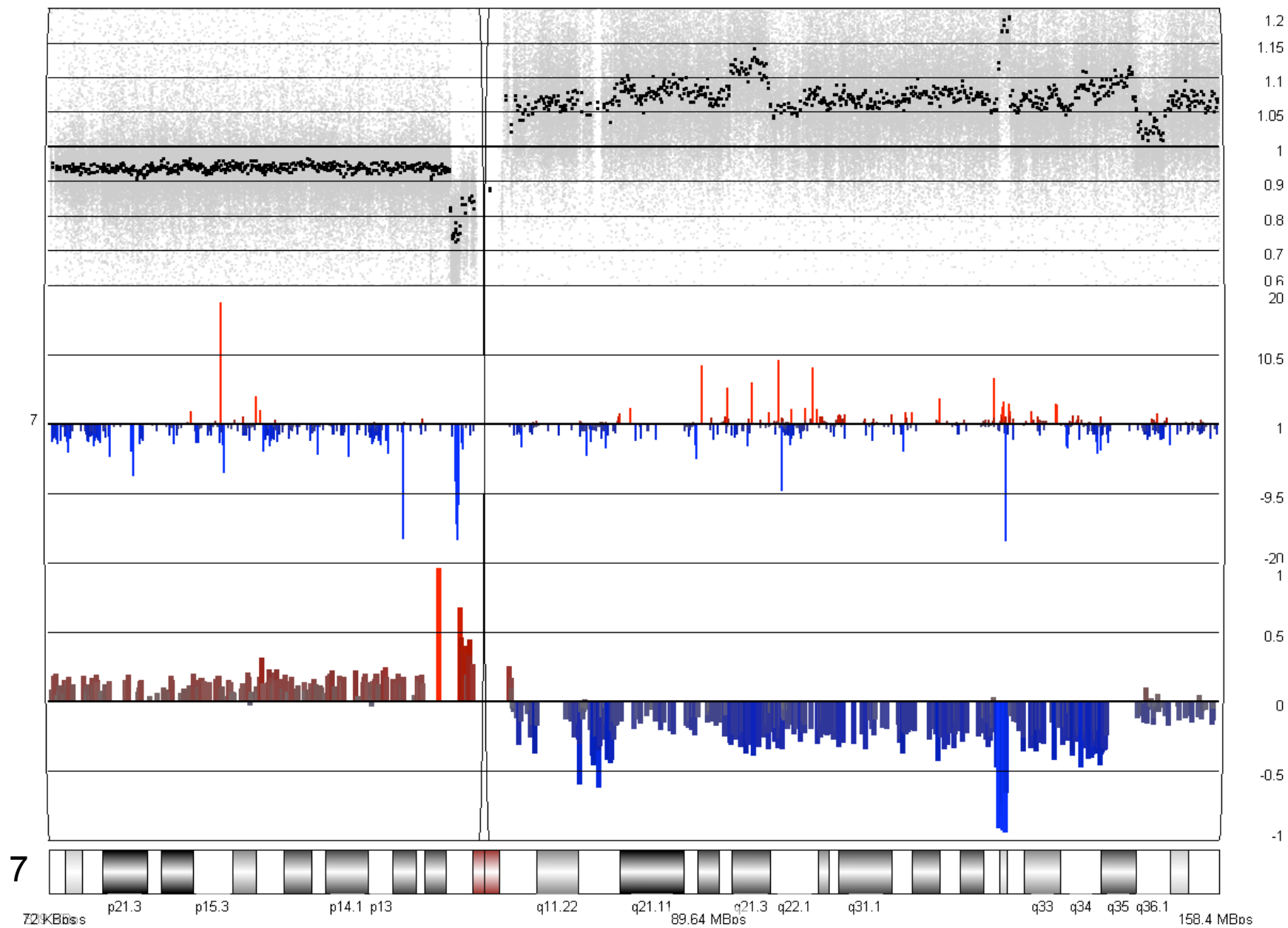

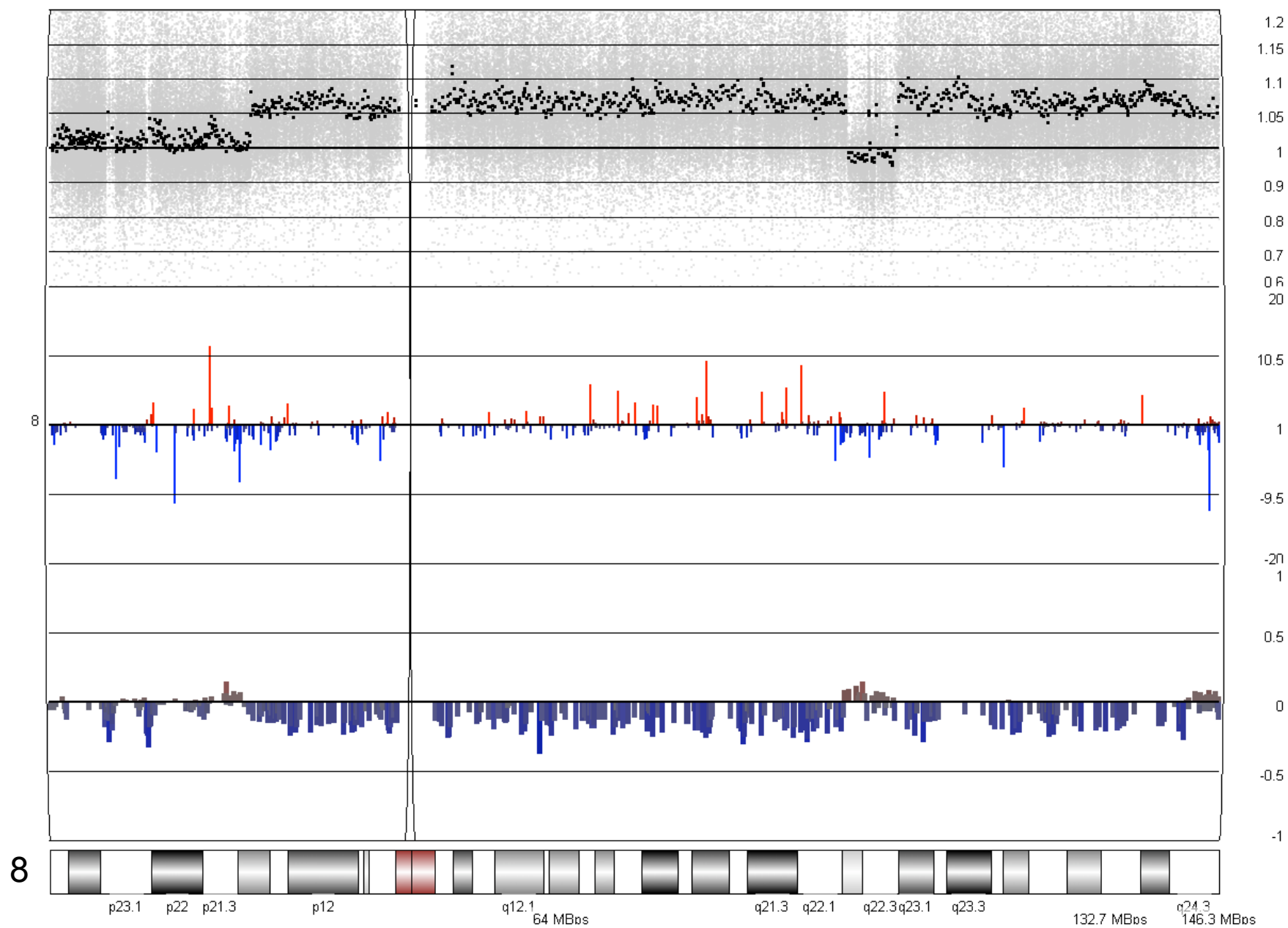

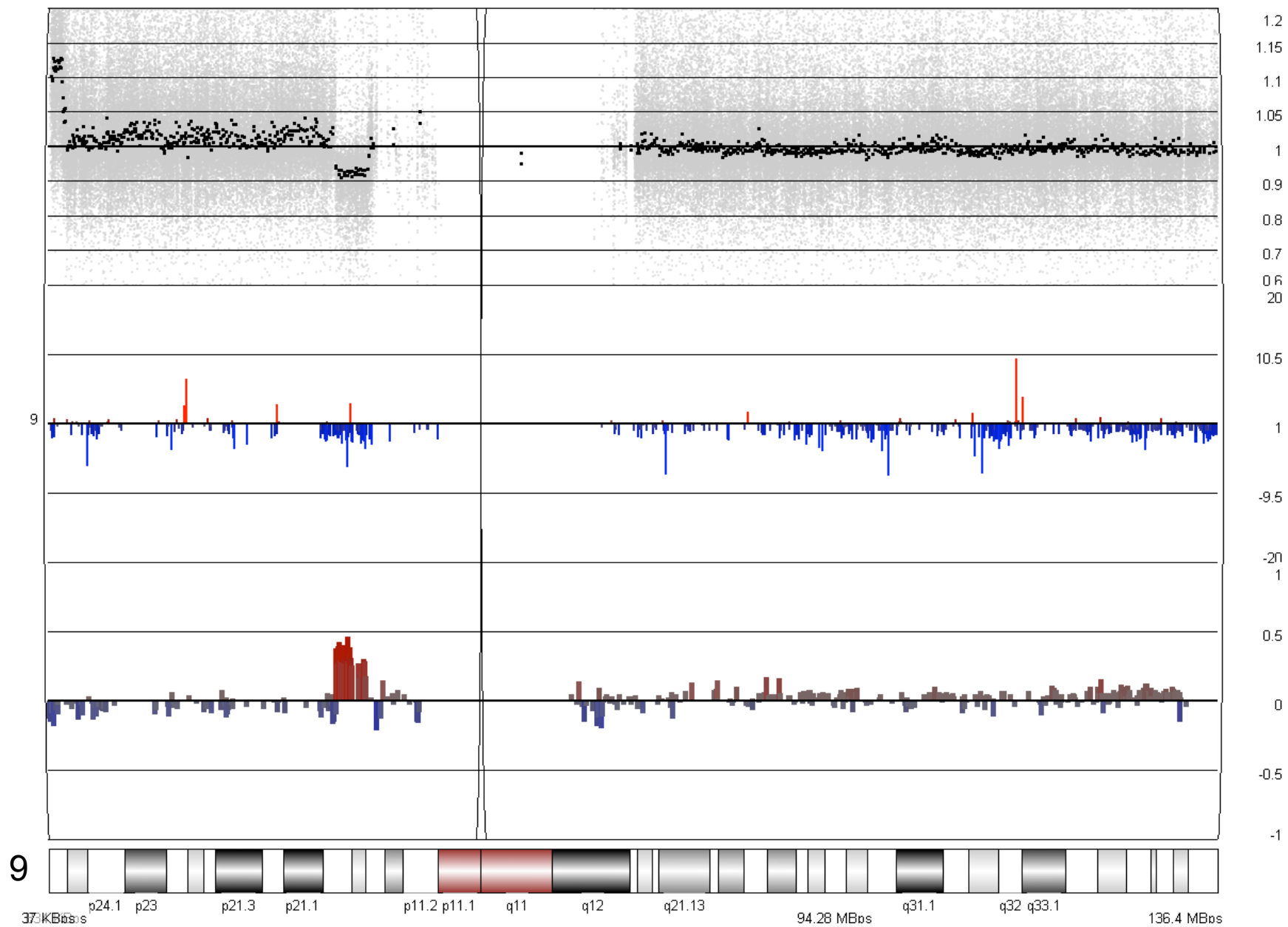

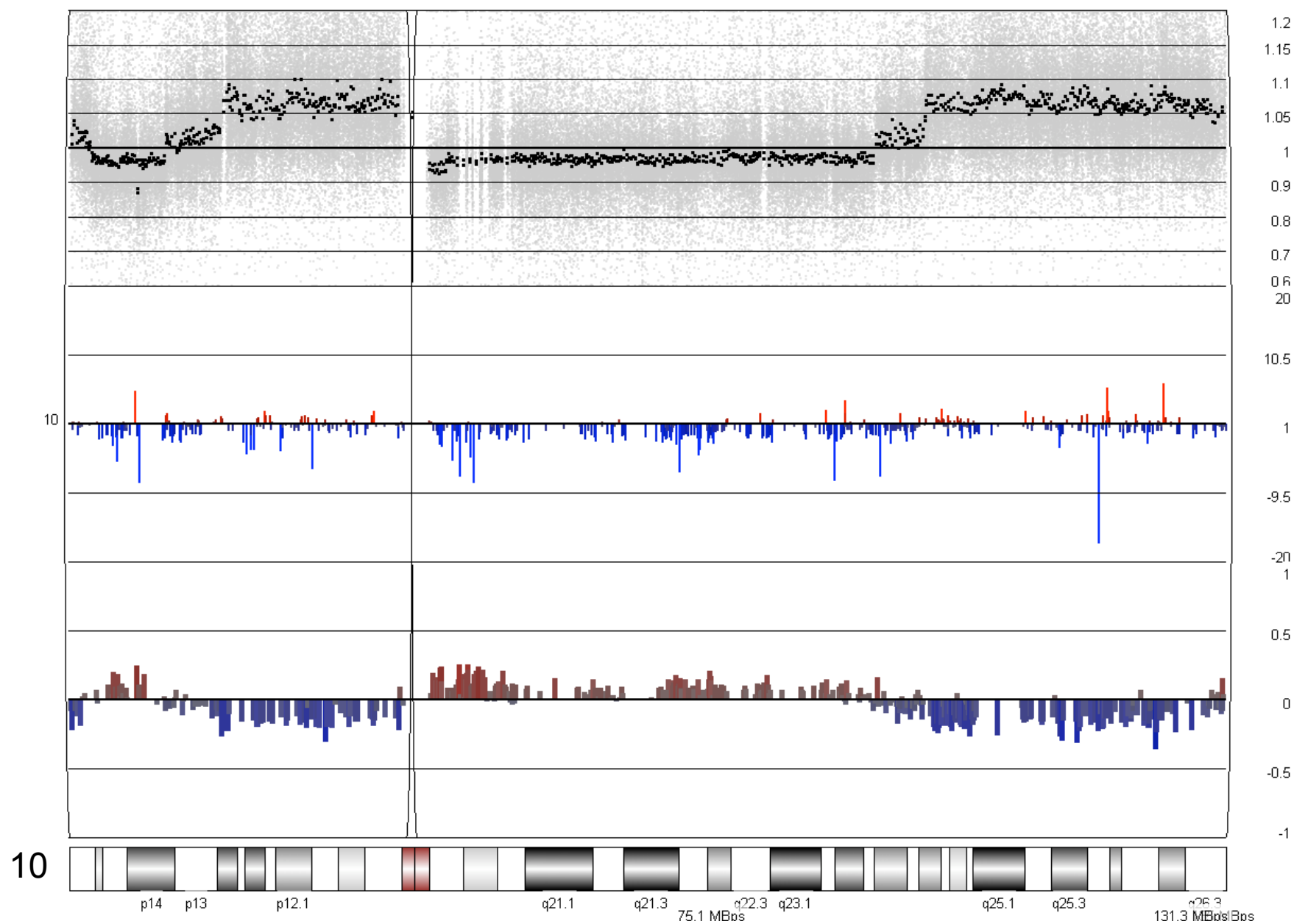

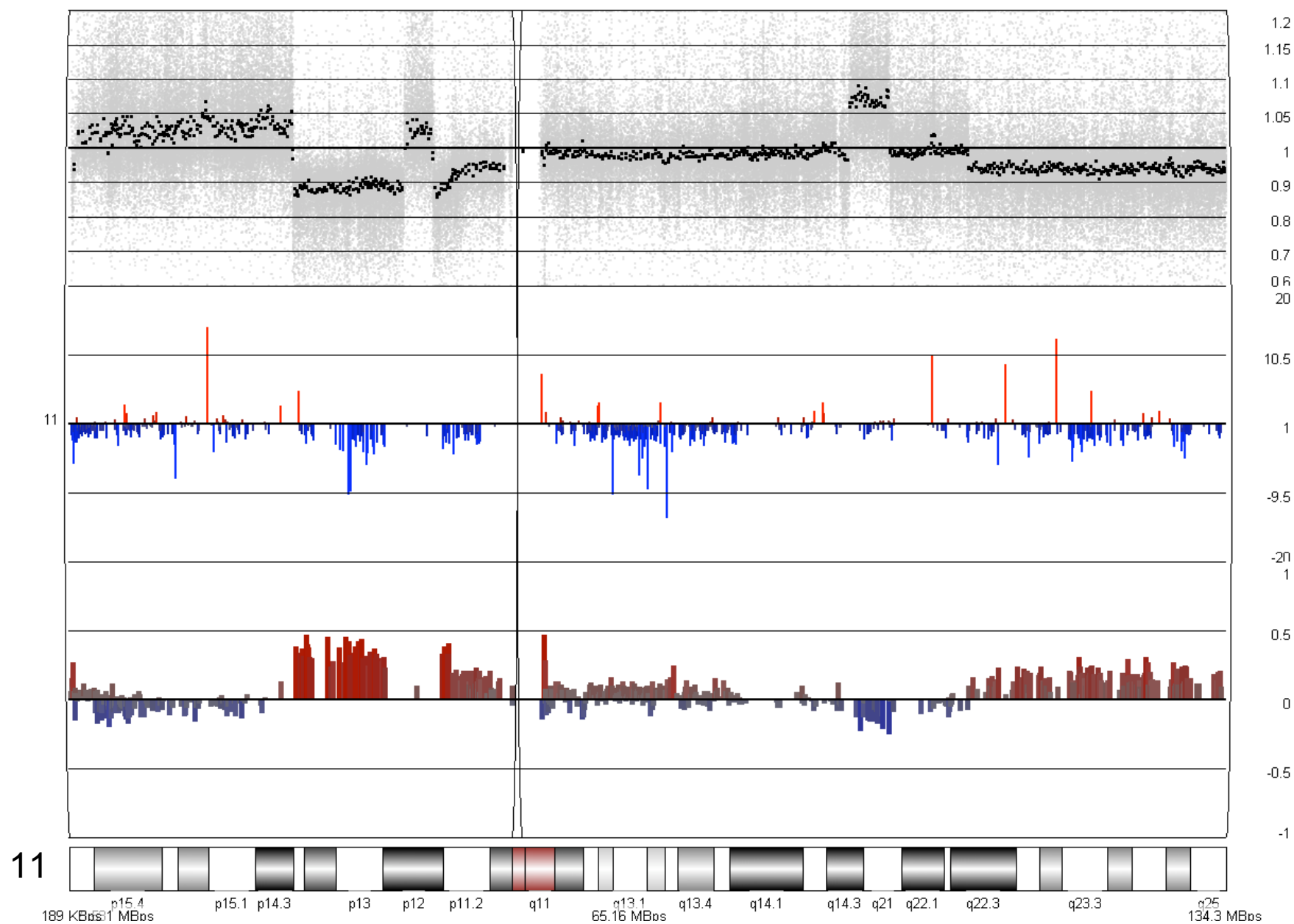

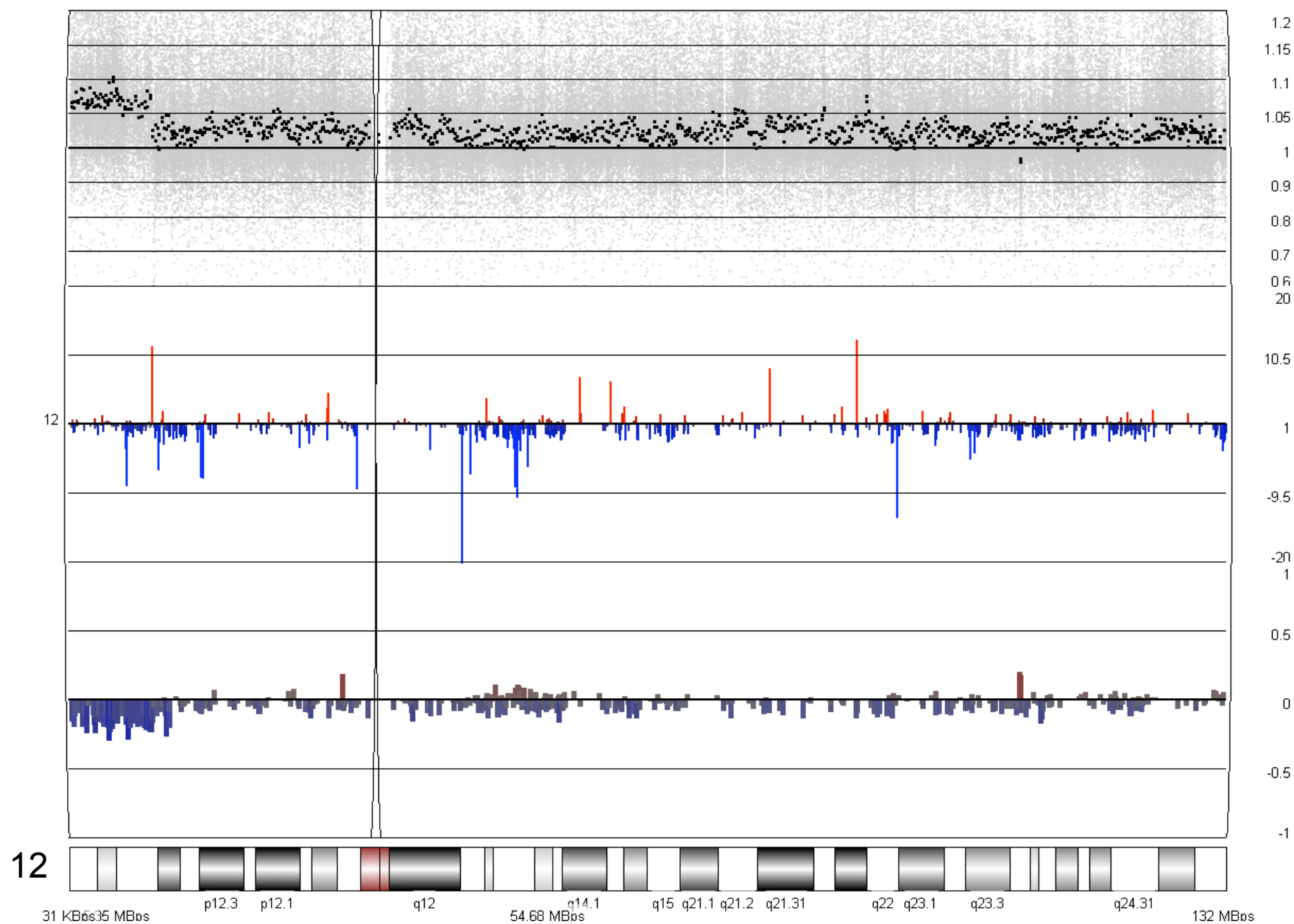



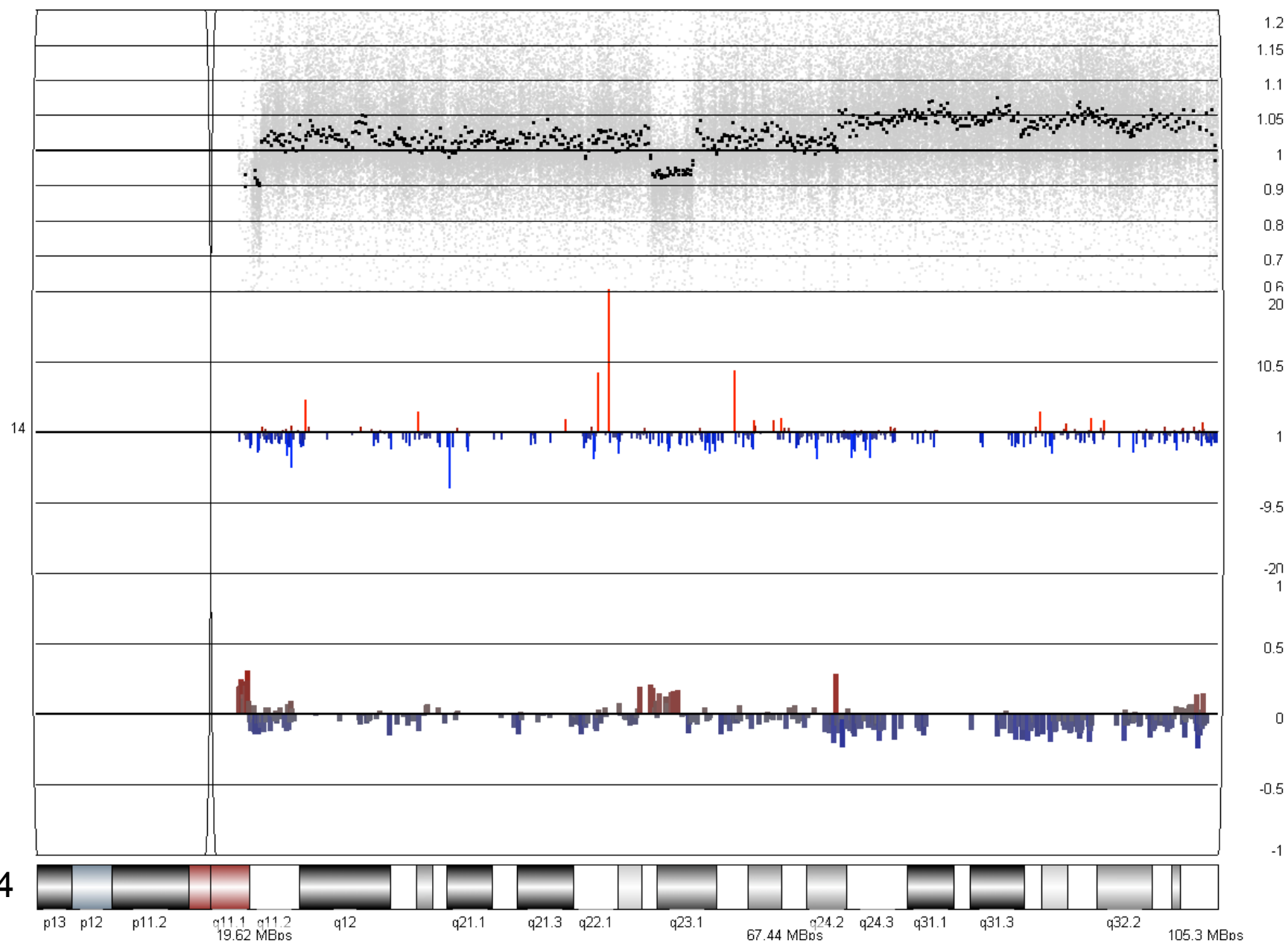

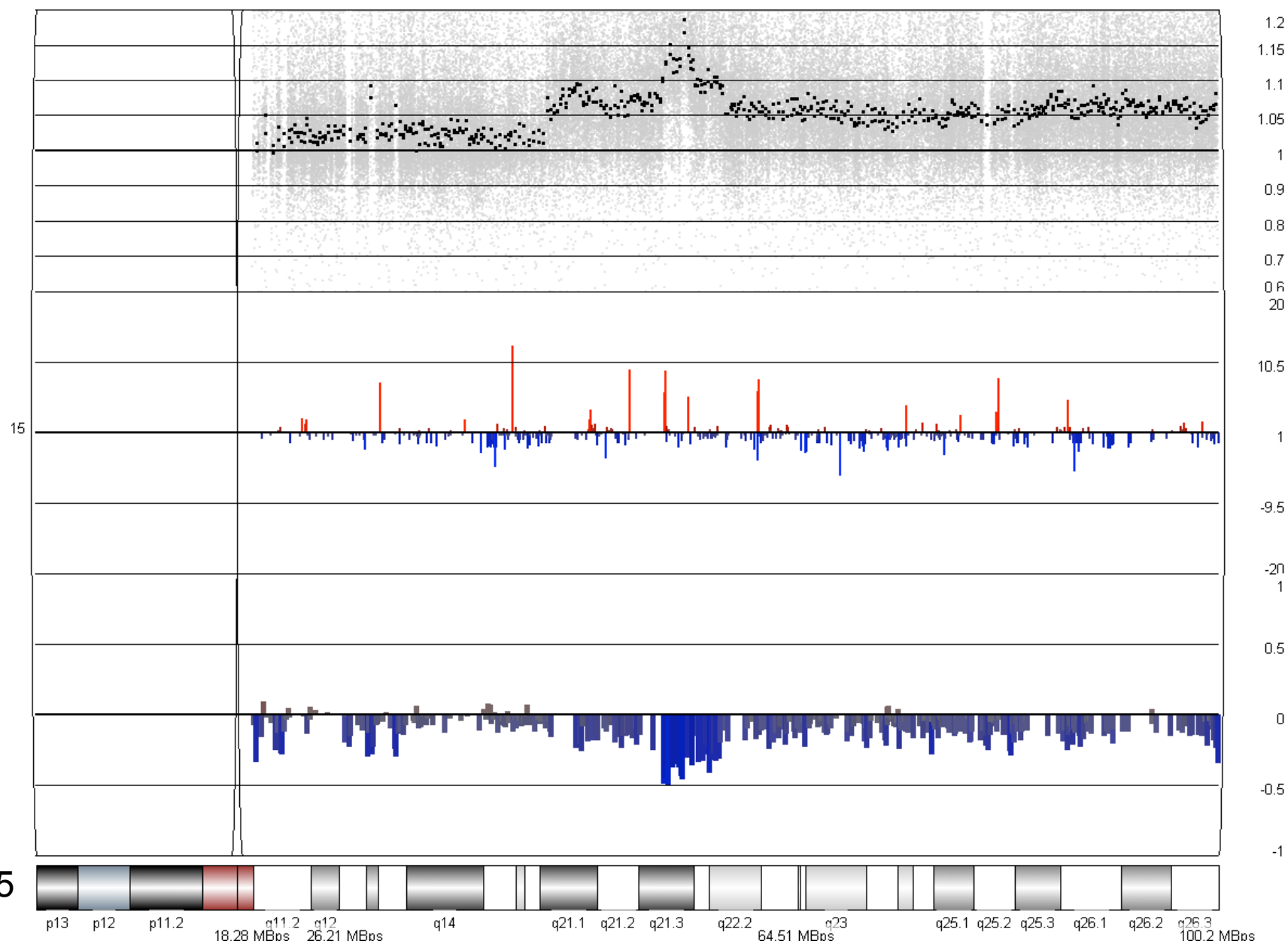

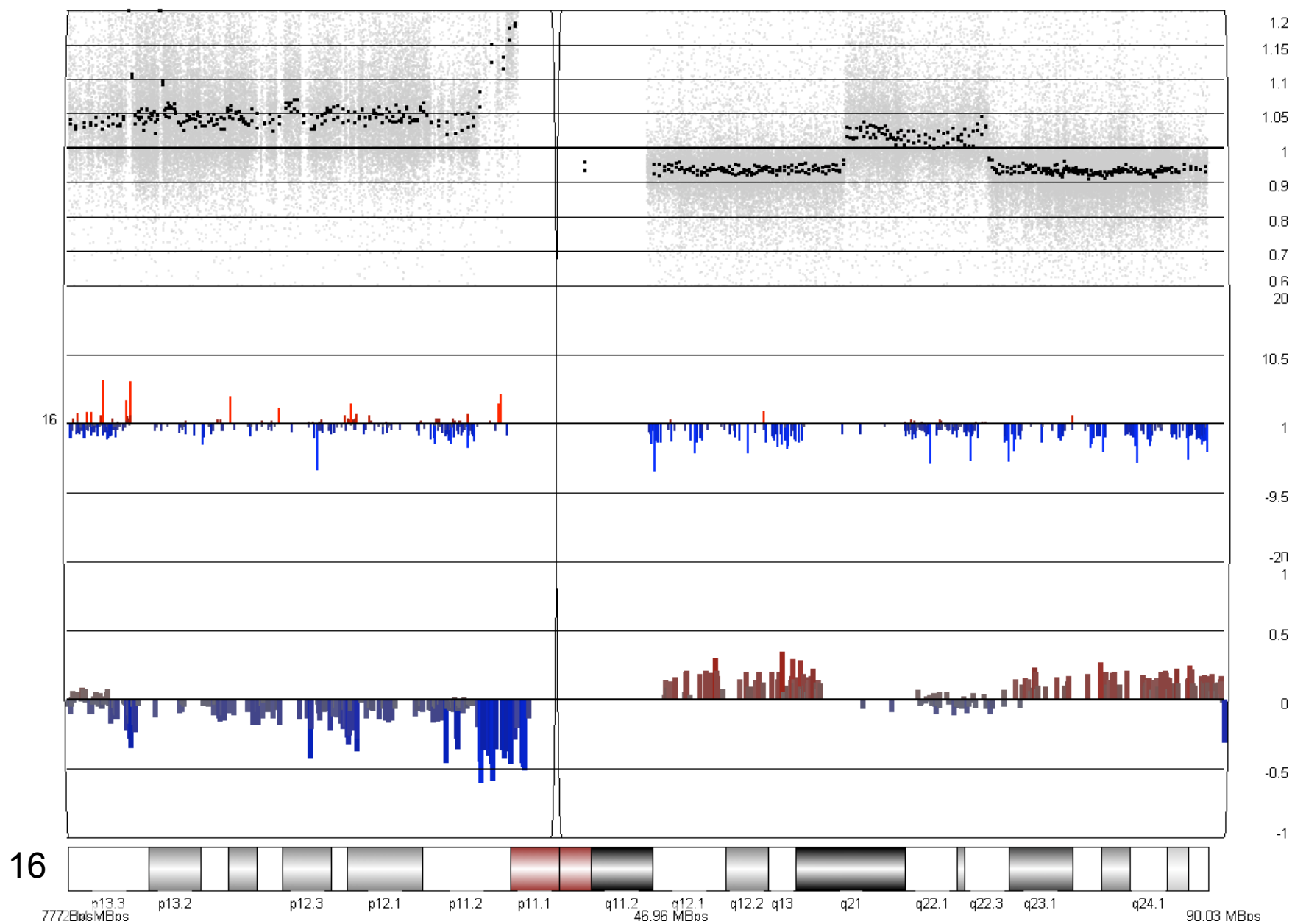

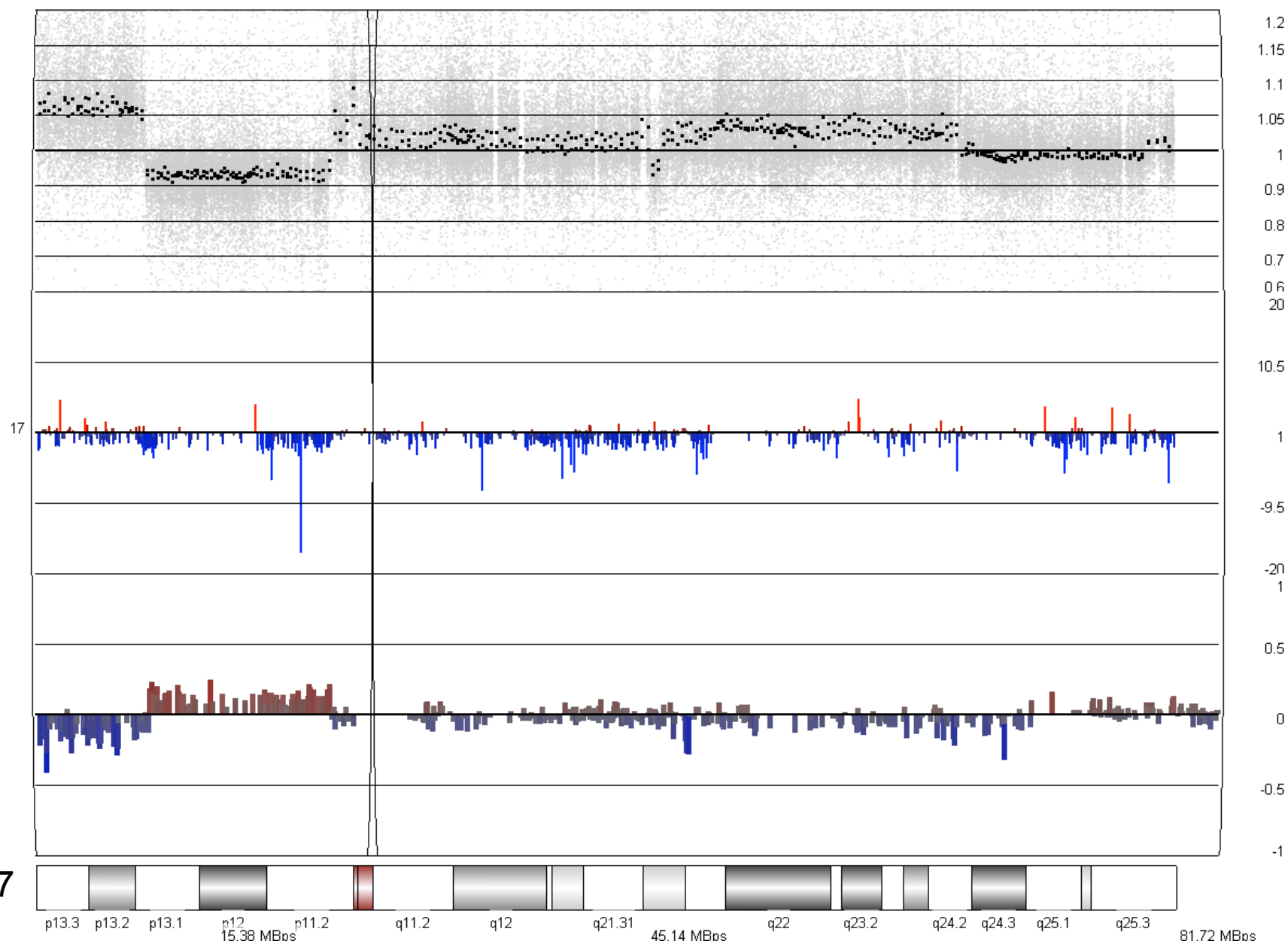

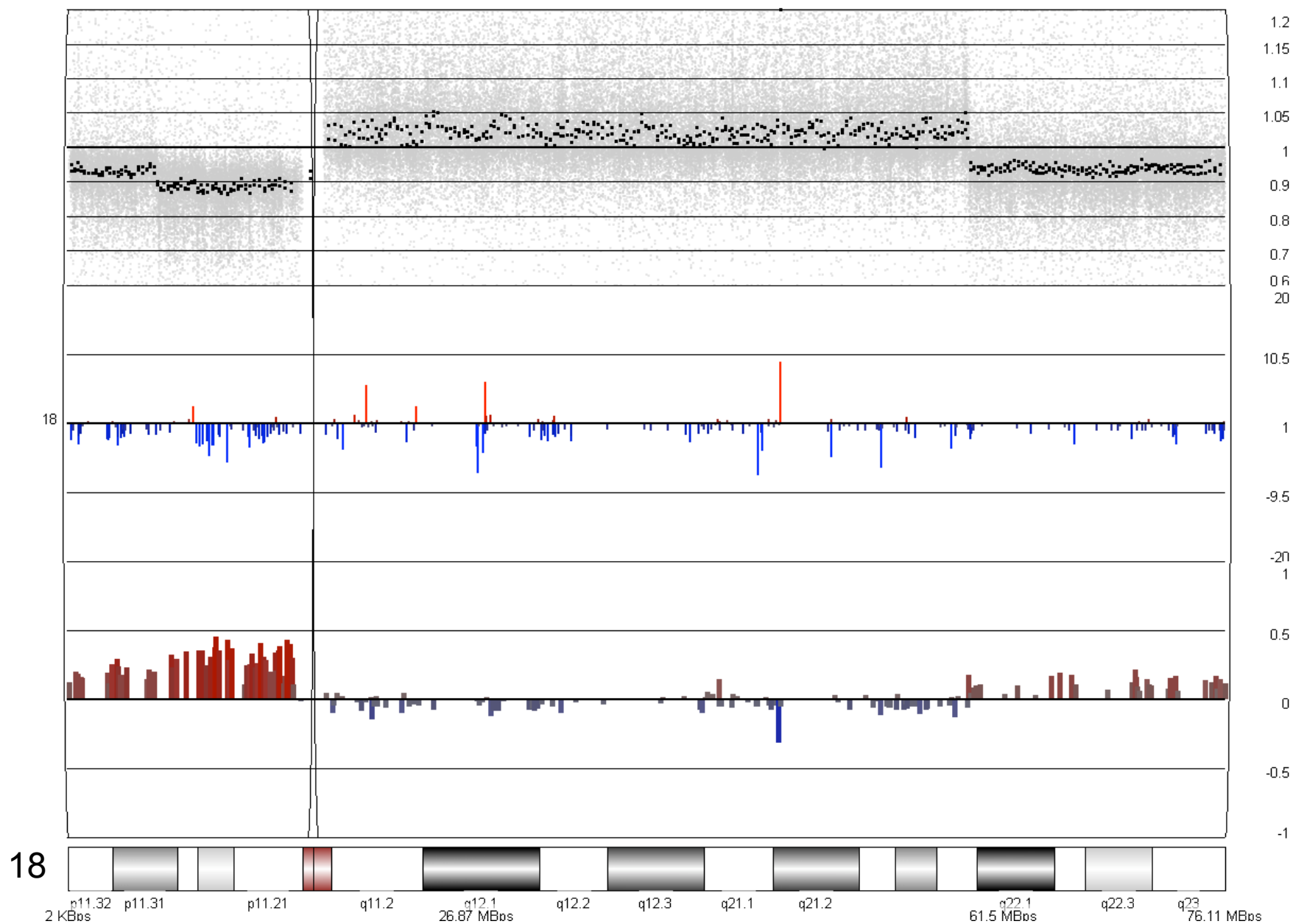

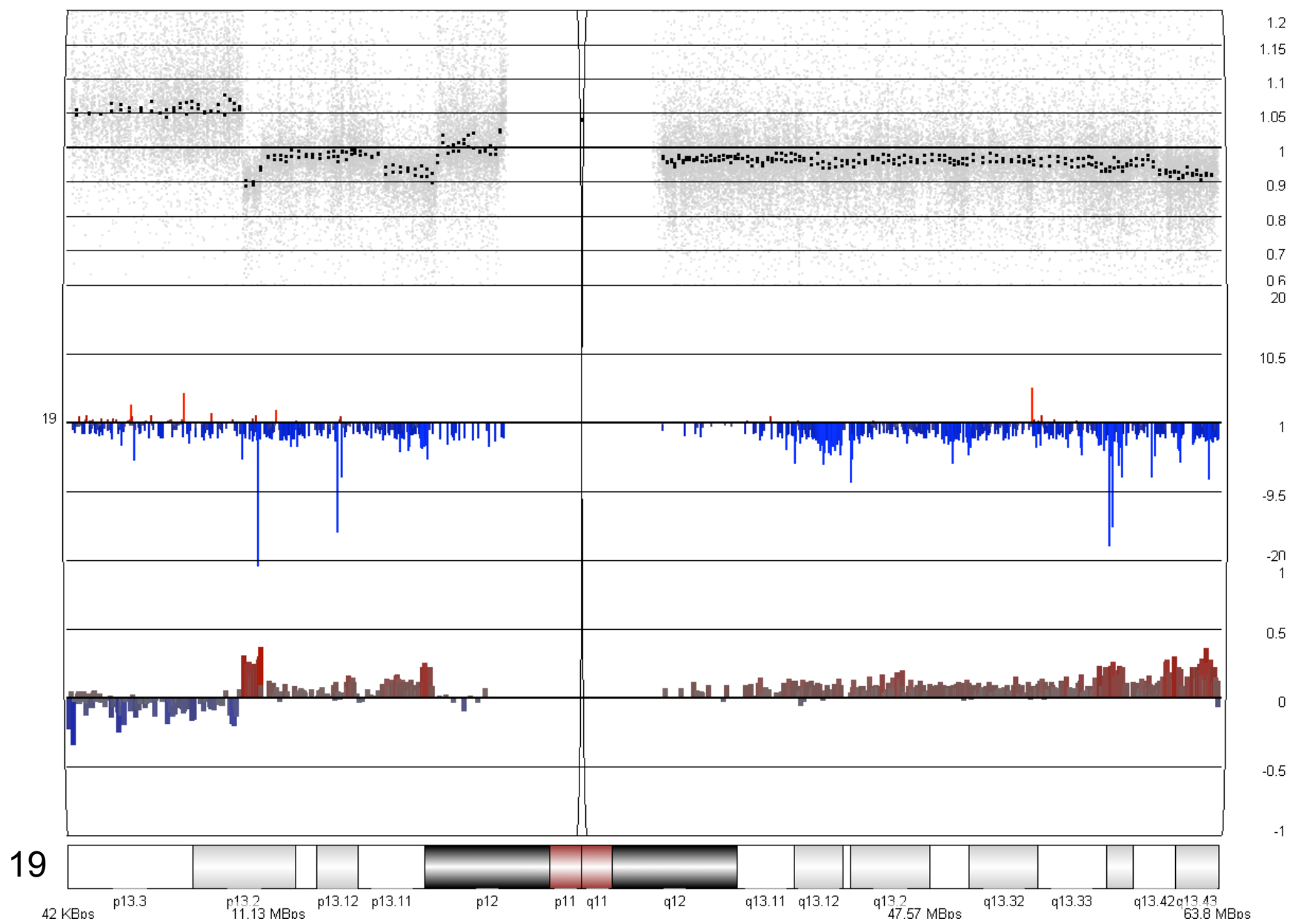

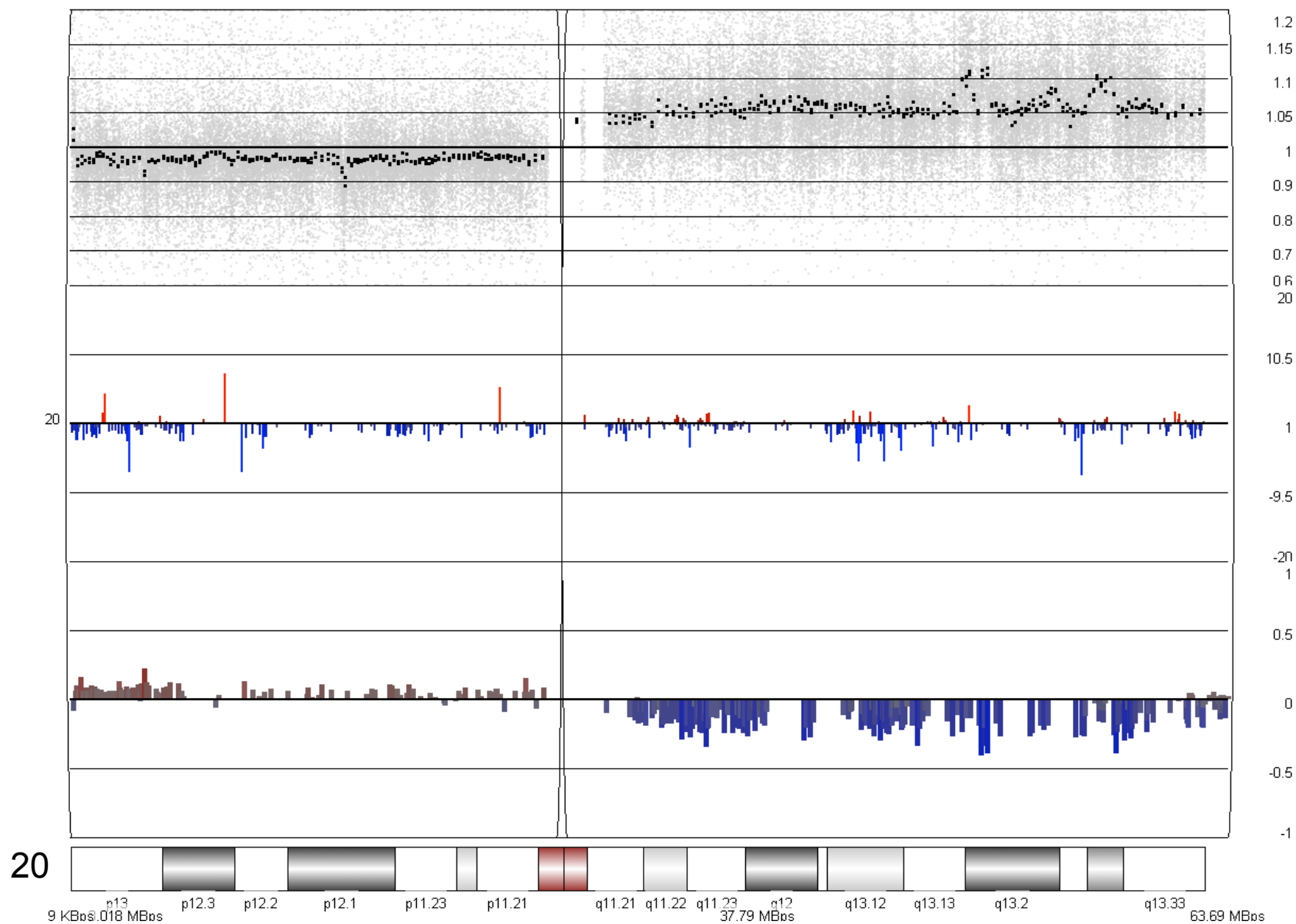

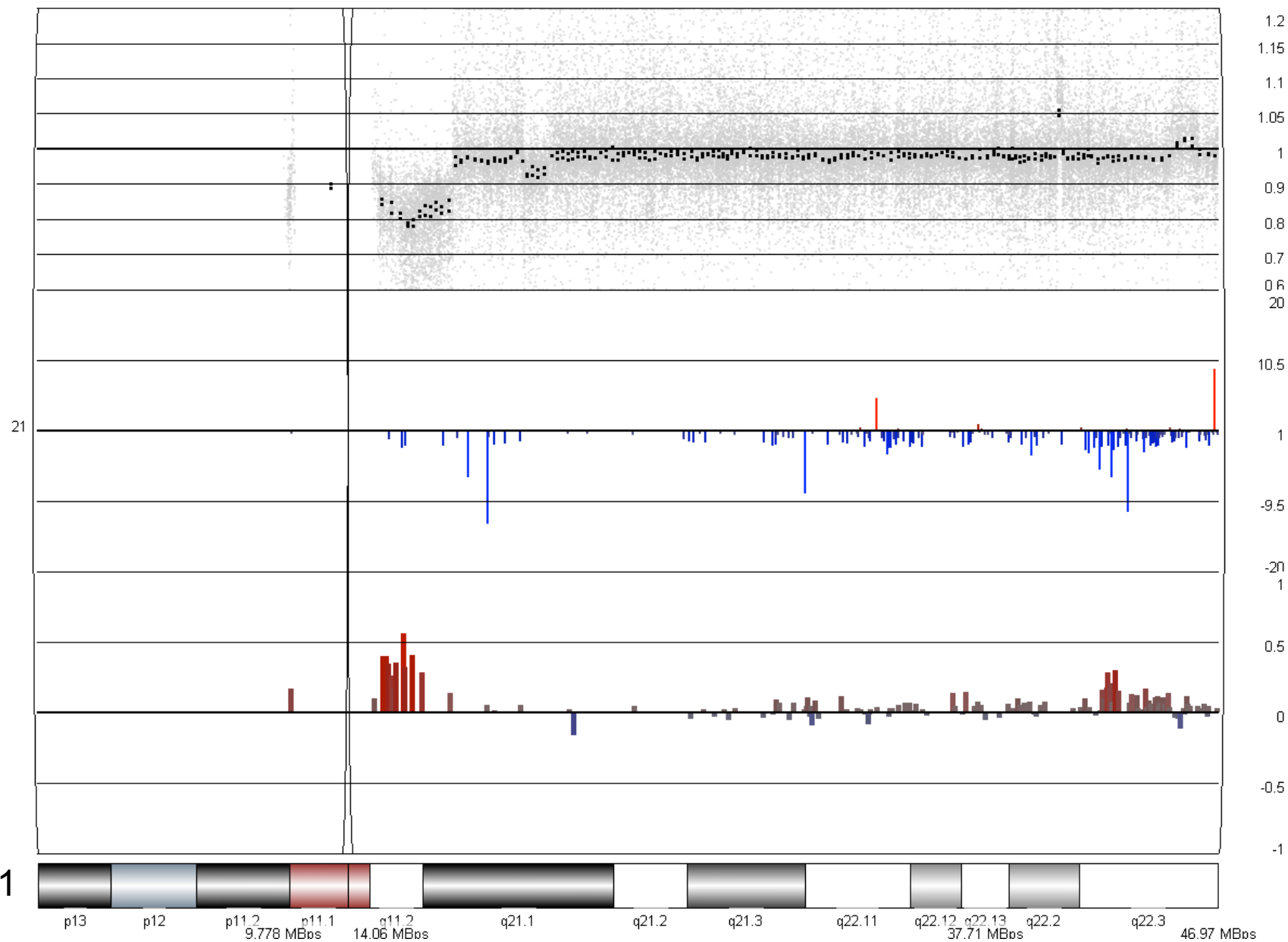

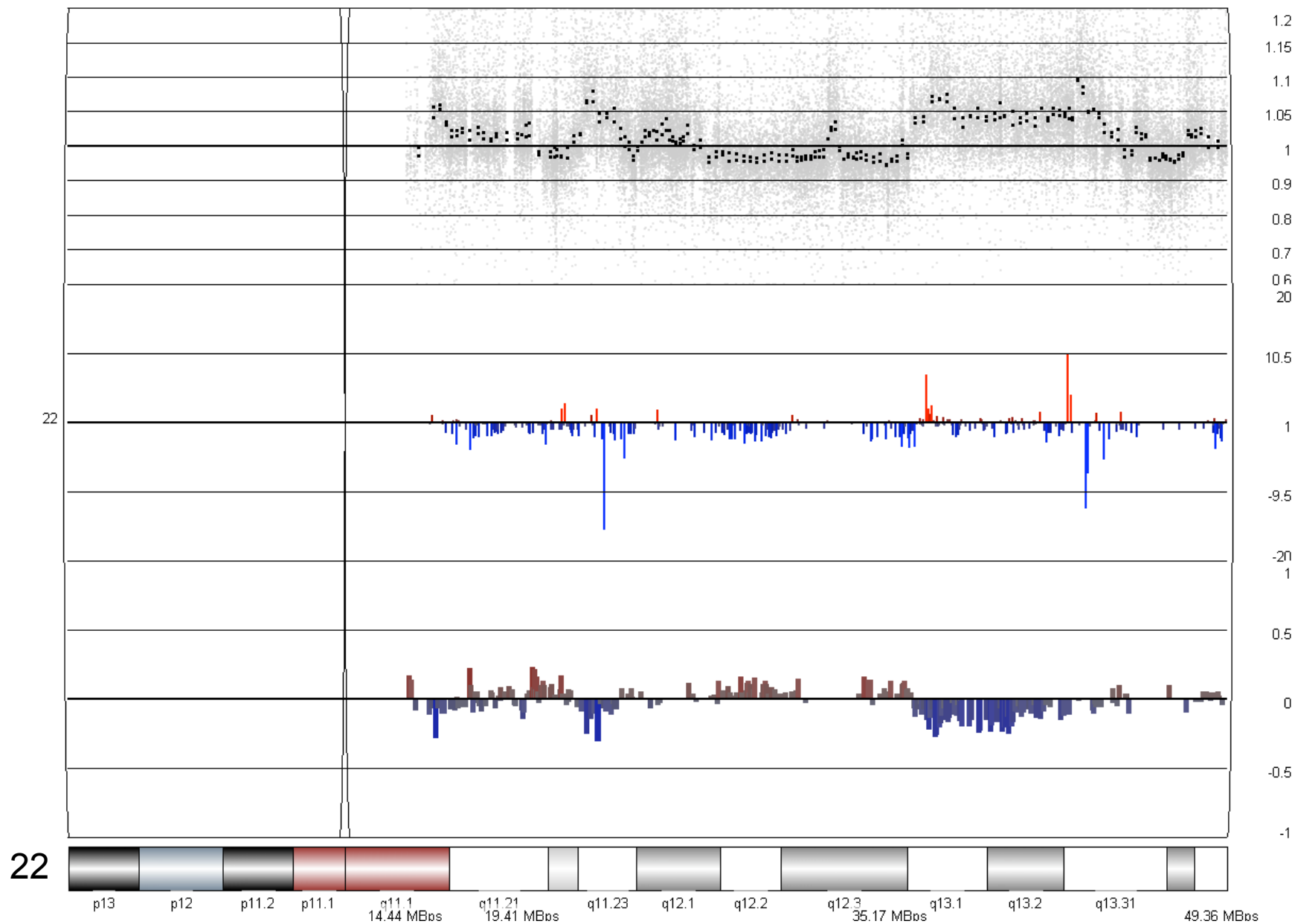

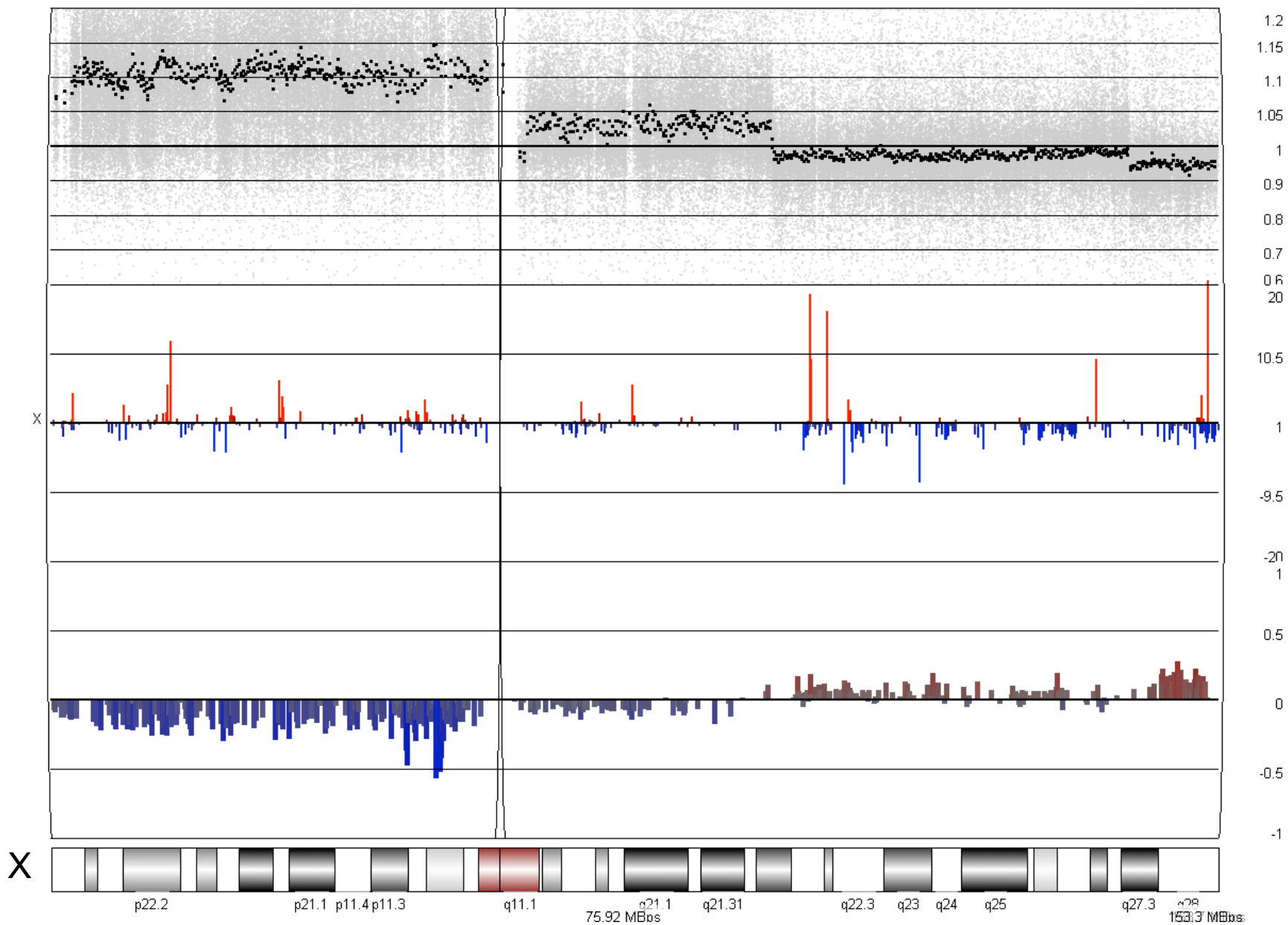

Supplement: Figure S1 — Multi-platform integrative analysis of copy number, expression, and promoter array data. (2.06 MB PDF) [file pone.0008665.s001.pdf]
