# Supplementary material for: Multi-Platform Whole-Genome Microarray Analyses Refine the Epigenetic Signature of Breast Cancer Metastasis with Gene Expression and Copy Number
Source: PLoS One. 2010 Jan 13;5(1):e8665. doi: 10.1371/journal.pone.0008665 (PMC2801616; doi:10.1371/journal.pone.0008665)
Supplement: Table S1 — PCR Primer List. (0.03 MB PDF) [file pone.0008665.s002.pdf]

Supplemental Table 1: Primer sets and conditions for PCR.

Primer set[1]	Forward (5'-3')	Reverse (5'-3')	Annealing Temp (degC)	Expected band size (bp)
**HOXD13-BGS**	AGTGGGTGGGTTTAGTTAGGTTTG	CTCTAACCCTCTCTCCCTCTATAA	55	245
**HOXD13-QRT**	CGTGGATGCCTTCATTTCTTGAGC	AGATGACCACAATCCCAGACCCAT	60	134
**HOXD13-CNV**	CCTCAGCTAGGTGCTCCG	TTGCCACCATTGGCAGG	62	424
**DLC1-BGS**	GTTTTTAGTTAGGATATG	ACTTCTTTCTACACTCAAAC	55	292
**DLC1-QRT**	AGTGTATGCGTACCTGTGTCGCTT	TGTGCCAGACACTGAATCCACCTT	60	193
**DLC1-CNV**	CGTCACCACACTGCAAATAG	GACGTGTTTGTTTAAGGCATTC	62	369
**WNT5A-BGS**	CCA CAT TTG GGG TTG GAA AGC CCT AAT TAC	AAA AAA CTC AAT TAA CTT CCA ACA AAA	55	474
**WNT5A-QRT**	GATCGTTAGCAGCATCAGTCCACA	CTGTGCCTTCGTGCCTATTTGCAT	60	114
**WNT5A-CNV**	GCAATAAACATCTGCTCCTTTC	GCATTAAATATTGCCGCATC	60	269
**TMEM16A-BGS**	TTT TTC TTC CTG TGT TTA AAT TTC TA	CAA ATA TTA ATA ATA TAA AC	55	468
**TMEM16A-QRT**	TTAAGCAGGAAACAGCACCCTTGG	TCTGTGGAAATGCTTCCGAGGTCA	60	170
**TMEM16A-CNV**	ATGTGAGCGCCTCCAAAG	AGAACGCCTCCAGGCTC	62	158
**SFN-BGS**	TTGGGTTTGGTTATTTAGTTAAAAGG	ACATCTCCTTCTTACTAATATCCAT	55	578
**SFN-QRT**	TGC TGC CTC TGA TCG TAG GAA TTG	TTC CCT CAA TCT CGG TCT TGC ACT	60	103
**SFN-CNV**	GCC TAT AAG AAC GTG GTG GG	AAT GAT GCG CTT CTT GTC G	62	294
**EGFR-BGS**	GATCGGGTTTAGAGGGGTAGTGTTGGAAA	ATCAATACTAAACRAAATCAAAAAACC	55	511
**EGFR-QRT**	GTGACCGTTTGGGAGTTGATGA	GGCTGAGGGAGGCGTTCTC	60	103
**EGFR-CNV**	CCTGGACCTTGAGGGATTG	CTTCAAGTGGAATTCTGCCC	62	313
**HBB (B-GLOBIN)-CNV**	GAGGGTTTGAAGTCCAACTCCTAA	CAGGGTGAGGTCTAAGTGATGACA	62	57
**GAPDH-QRT**	CATGTTCGTCATGGGTGTGAACCA	ATGGCATGGACTGTGGTCATGAGT	60	155

BGS: primers for Bisulfite genomic sequencing

QRT: primers used for quantitative real time PCR for mRNA abundance

CNV: primers used for quantitative real time PCR for copy number analysis
