# Supplementary material for: Multi-Platform Whole-Genome Microarray Analyses Refine the Epigenetic Signature of Breast Cancer Metastasis with Gene Expression and Copy Number
Source: PLoS One. 2010 Jan 13;5(1):e8665. doi: 10.1371/journal.pone.0008665 (PMC2801616; doi:10.1371/journal.pone.0008665)
Supplement: Table S2 — Regions showing an increase in copy number, (468LN relative to 468GFP). Included are associated genes contained within the regions. (0.07 MB PDF) [file pone.0008665.s003.pdf]

Supplemental Table 2: Regions showing an increase in copy number, (468GFP-LN relative to 468GFP)

#chromosome	region	length	**average Copy number**	# probesets
7	75704207-75709416	5209	**47.5**	3
15	54988480-55048156	59676	**39.1**	28
7	154024104-154030546	6442	**33.6**	4
17	36769083-36776021	6938	**22.2**	28
18	46765619-46813542	47923	**21.9**	32
16	4500302-4933725	433423	**20.0**	231
7	11674017-11687918	13901	**18.3**	10
16	7020605-7289951	269346	**16.9**	395
22	22680529-22726814	46285	**16.8**	19
14	23515474-23549549	34075	**16.6**	22
18	46823292-46858328	35036	**14.6**	104
16	32964249-33149454	185205	**14.6**	4
8	63380105-63386639	6534	**14.3**	7
1	22191588-22209830	18242	**14.3**	10
13	47796160-47854097	57937	**14.1**	102
8	102689782-102694888	5106	**13.8**	10
11	661522-682908	21386	**13.4**	16
8	2067801-2068760	959	**13.2**	6
5	138164484-138172714	8230	**12.9**	6
13	47882816-47957722	74906	**12.2**	79
7	130012614-130450545	437931	**10.8**	285
2	180128688-180130114	1426	**10.7**	14
13	47784709-47796149	11440	**10.5**	10
7	128899812-130010707	1110895	**9.9**	526
X	71941665-72116832	175167	**9.9**	11
X	104790819-104791232	413	**9.9**	5
7	27663117-27663474	357	**8.4**	4
18	46820307-46823139	2832	**8.1**	8
18	46815780-46819630	3850	**7.9**	13
3	86572955-90419180	3846225	**7.8**	2102
5	6665243-6665674	431	**7.8**	4
4	20977791-20985949	8158	**7.7**	16
15	55054108-55054490	382	**7.3**	2
3	84788974-86564578	1775604	**7.2**	1042
5	170225838-170512757	286919	**6.6**	123
16	32472250-32756399	284149	**6.6**	58
10	126434380-126591915	157535	**6.5**	69
5	65217734-65506940	289206	**6.4**	158
7	38450645-38455163	4518	**6.3**	6
15	28173703-28608929	435226	**6.1**	112
1	21409134-22097877	688743	**6.0**	401
1	3436673-12615712	9179039	**6.0**	5642
1	1286515-1867650	581135	**5.9**	148
1	13994326-16021930	2027604	**5.9**	1587
1	1878053-2930477	1052424	**5.8**	303
1	16038248-20970055	4931807	**5.8**	3227
3	191536487-193112515	1576028	**5.8**	1089
1	22210712-22743899	533187	**5.7**	363
15	55057549-55764951	707402	**5.7**	536
18	68669215-68670320	1105	**5.6**	7
15	53138984-54576939	1437955	**5.5**	819
15	54588915-54979351	390436	**5.5**	181
3	78799828-79607716	807888	**5.4**	409
3	187190536-187932763	742227	**5.3**	685
7	95137390-96450494	1313104	**5.3**	867
3	73452374-74570462	1118088	**5.3**	904
20	48853165-49615192	762027	**5.3**	578
3	68831448-70546020	1714572	**5.3**	1395
20	50017099-50466370	449271	**5.2**	299
3	182373513-184155953	1782440	**5.2**	986
16	7018114-7018149	35	**5.0**	2
3	74570835-75326887	756052	**4.9**	442
3	187933930-190453782	2519852	**4.9**	1902
20	48847293-48849109	1816	**4.9**	3
3	68171699-68807382	635683	**4.9**	469
3	193112683-196192769	3080086	**4.9**	2169
17	41750187-42107479	357292	**4.9**	52
3	179873655-182373376	2499721	**4.8**	1521
3	78733335-78798447	65112	**4.8**	48
3	197261608-199380515	2118907	**4.8**	1062
3	79610213-79835196	224983	**4.7**	145
3	191183987-191536470	352483	**4.7**	284

3	184156049-187189164	3033115	**4.7**	1631
7	96452073-96856682	404609	**4.7**	228
3	59885745-60087335	201590	**4.7**	302
4	154192260-155062535	870275	**4.7**	636
20	56057473-57423860	1366387	**4.7**	1026
3	79845163-84184912	4339749	**4.6**	2354
3	190465502-191181438	715936	**4.6**	565
3	75328509-78733250	3404741	**4.6**	1978
3	196196720-197260987	1064267	**4.6**	430
7	92092148-95107736	3015588	**4.5**	1960
22	43032379-43424682	392303	**4.5**	270
13	45042209-47784283	2742074	**4.5**	1924
13	47962809-48760145	797336	**4.5**	502
3	70680537-73222167	2541630	**4.5**	1841
7	138994319-141693868	2699549	**4.5**	1829
3	65043015-68171639	3128624	**4.5**	2182
7	96857573-97773607	916034	**4.5**	536
7	143682164-147108692	3426528	**4.4**	2265
3	177695202-179873152	2177950	**4.4**	1335
X	26727046-27957653	1230607	**4.3**	813
X	46178486-55533764	9355278	**4.3**	3983
9	30910-1520000	1489090	**4.2**	1659
X	24202309-26709573	2507264	**4.2**	1545
15	55769853-58299409	2529556	**4.2**	1892
7	141699450-142154515	455065	**4.2**	412
1	45726572-45976083	249511	**4.2**	97
X	9290675-17095515	7804840	**4.2**	5522
X	17097014-22481252	5384238	**4.2**	3018
7	142176272-143542557	1366285	**4.2**	868
15	30226573-30691968	465395	**4.2**	79
20	53805479-54321002	515523	**4.2**	427
13	47854202-47882733	28531	**4.1**	20
X	27959744-42078035	14118291	**4.1**	8994
5	56694759-57036303	341544	**4.1**	253
X	1917029-7543877	5626848	**4.1**	3911
1	26920933-31915725	4994792	**4.1**	2710
14	36306662-36352544	45882	**4.1**	56
5	58060726-61532033	3471307	**4.1**	2300
X	42089168-45408106	3318938	**4.0**	1805
8	132832538-133319203	486665	**4.0**	374
12	4911842-5395474	483632	**4.0**	491
5	82508048-83236914	728866	**4.0**	425
8	136613731-137615008	1001277	**4.0**	777
10	127884570-129160888	1276318	**3.9**	1023
X	23194174-24200150	1005976	**3.9**	678
22	21976553-22426783	450230	**3.9**	279
7	121318047-124783194	3465147	**3.9**	2057
10	127810968-127880313	69345	**3.8**	97
7	116190822-117809597	1618775	**3.8**	954
X	55534964-58319626	2784662	**3.8**	1369
7	106425024-108163417	1738393	**3.8**	1080
5	83237952-83304907	66955	**3.8**	54
10	108466837-109505540	1038703	**3.8**	843
7	83802374-85198094	1395720	**3.8**	818
8	136049738-136609910	560172	**3.8**	388
7	135872372-137076595	1204223	**3.8**	811
7	102139083-105364036	3224953	**3.8**	2069
12	91019006-91185828	166822	**3.7**	117
X	7544067-9290510	1746443	**3.7**	1358
7	91764443-92091908	327465	**3.7**	176
10	117702957-119000175	1297218	**3.7**	936
7	120013114-121317792	1304678	**3.7**	851
7	138974507-138993906	19399	**3.7**	15
X	108477-1894383	1785906	**3.7**	265
X	22492554-23193660	701106	**3.7**	618
12	5395521-5646508	250987	**3.7**	243
8	133321381-134650462	1329081	**3.6**	1183
10	129161079-129366465	205386	**3.6**	167
22	22426890-22660218	233328	**3.6**	154
7	147111995-147131735	19740	**3.6**	9
10	134570505-135356694	786189	**3.6**	336
5	61535707-63757272	2221565	**3.6**	1239
7	117810095-120012951	2202856	**3.6**	1214
7	108164312-109214872	1050560	**3.6**	680
7	124801133-128899323	4098190	**3.6**	2597
7	147132559-147649475	516916	**3.6**	434
7	97774291-97781659	7368	**3.6**	6

10	105742493-108466097	2723604	**3.5**	2069
12	20703-4902901	4882198	**3.5**	3489
5	77834914-82497833	4662919	**3.5**	2907
3	60087399-65041681	4954282	**3.5**	4406
7	130457966-135870490	5412524	**3.5**	3838
20	57492868-62384265	4891397	**3.5**	3128
7	75711830-83800312	8088482	**3.5**	5511
5	55467361-56694137	1226776	**3.5**	916
10	126599637-127808232	1208595	**3.5**	853
7	109241465-116190739	6949274	**3.5**	4318
8	138231733-146268959	8037226	**3.5**	4870
10	132136144-132877123	740979	**3.5**	703
5	54518564-55462146	943582	**3.5**	482
10	109507892-117700285	8192393	**3.5**	5746
8	134655412-136049429	1394017	**3.5**	1214
8	127956222-132625360	4669138	**3.5**	3405
7	154031950-158810207	4778257	**3.5**	3269
5	58005107-58060653	55546	**3.5**	37
3	49418085-59884355	10466270	**3.5**	6379
5	63760791-65215536	1454745	**3.5**	865
7	85199012-91764216	6565204	**3.5**	4086
7	105365127-106422804	1057677	**3.5**	773
5	49596050-54514773	4918723	**3.4**	3200
10	119003578-126426756	7423178	**3.4**	5768
10	100019596-105738745	5719149	**3.4**	3146
20	49616647-50015682	399035	**3.4**	295
10	129366515-132130439	2763924	**3.4**	2240
5	65508746-77834188	12325442	**3.4**	7107
20	28103258-48842911	20739653	**3.4**	12661
17	21506946-22142010	635064	**3.4**	228
15	51735154-53138983	1403829	**3.4**	1106
5	83305251-83971611	666360	**3.4**	342
20	54338133-55996422	1658289	**3.4**	1366
15	43147386-51731473	8584087	**3.4**	5561
11	90620872-95331300	4710428	**3.4**	3427
8	42979170-53812620	10833450	**3.4**	3987
7	97782124-102136421	4354297	**3.4**	1825
8	53815958-63375932	9559974	**3.4**	6463
7	151429049-154020828	2591779	**3.4**	1674
17	526-7515695	7515169	**3.4**	4323
20	50466864-51242945	776081	**3.4**	634
15	88850762-90257901	1407139	**3.3**	995
20	51245861-53689470	2443609	**3.3**	2034
7	137079626-138971429	1891803	**3.3**	1100
10	36350756-39114827	2764071	**3.3**	1566
12	5646821-9423684	3776863	**3.3**	2048
8	63391700-99756416	36364716	**3.3**	22034
10	17678316-36346833	18668517	**3.3**	13036
7	64930393-75701960	10771567	**3.3**	5060
8	106057243-115930962	9873719	**3.3**	5880
6	168078127-168338768	260641	**3.3**	180
3	154233326-159195650	4962324	**3.3**	2992
8	115937583-127953830	12016247	**3.3**	8334
3	164111308-171934281	7822973	**3.3**	4568
21	40543968-40745546	201578	**3.3**	171
8	32811648-40301274	7489626	**3.3**	4263
15	90261698-100286563	10024865	**3.2**	7927
3	147081068-154232327	7151259	**3.2**	4599
8	27315068-32798198	5483130	**3.2**	3751
3	95002168-102619163	7616995	**3.2**	4219
5	57036474-58001786	965312	**3.2**	765
22	15546276-16090860	544584	**3.2**	376
19	41910-9681332	9639422	**3.2**	3787
3	171940031-177688920	5748889	**3.2**	3899
15	78938864-79874464	935600	**3.2**	612
22	43436490-44281401	844911	**3.2**	643
10	132882533-134560418	1677885	**3.2**	1015
8	103199421-103470330	270909	**3.2**	127
8	40312067-42977456	2665389	**3.2**	1715
3	102624138-131243312	28619174	**3.2**	17648
6	80007463-80946088	938625	**3.2**	644
6	77044929-79019608	1974679	**3.2**	1331
15	79883549-88847503	8963954	**3.1**	5637
8	25047152-27313704	2266552	**3.1**	1769
8	103709666-103886282	176616	**3.1**	112
15	58310954-78935266	20624312	**3.1**	13045
22	35991234-43031174	7039940	**3.1**	4175

6	79095953-80001776	905823	**3.1**	535
7	62892165-64885600	1993435	**3.1**	946
3	131293903-147075133	15781230	**3.1**	9944
9	34041128-34355357	314229	**3.1**	144
22	22731529-23487311	755782	**3.1**	426
14	79187069-81567551	2380482	**3.1**	1554
14	72183786-73020004	836218	**3.1**	483
3	159201141-163353922	4152781	**3.0**	2527
3	393585-584719	191134	**3.0**	173
10	342358-549266	206908	**3.0**	138
8	102298539-102577101	278562	**3.0**	248
14	73703618-79182423	5478805	**3.0**	3752
14	81573462-95785760	14212298	**3.0**	9788
16	15061218-29134899	14073681	**3.0**	8039
6	68284933-72923128	4638195	**2.9**	3038
11	15364316-15980260	615944	**2.9**	487
3	35345-391943	356598	**2.9**	350
16	7290455-14897340	7606885	**2.9**	6445
14	95787550-105399314	9611764	**2.9**	5595
16	4934417-6727409	1792992	**2.9**	2004
6	72932043-76210170	3278127	**2.9**	2111
6	63941094-65224771	1283677	**2.9**	649
4	109933430-110445238	511808	**2.9**	324
6	164733175-166140017	1406842	**2.8**	1059
16	777-476632	475855	**2.8**	222
16	30786351-32014468	1228117	**2.8**	492
16	476687-4500058	4023371	**2.8**	1683
2	144865617-146580899	1715282	**2.8**	1017
17	46790798-47714172	923374	**2.8**	761
4	4361778-4795240	433462	**2.8**	333
2	133251996-135006923	1754927	**2.7**	1387
6	80946400-80950048	3648	**2.4**	2
7	130011376-130012574	1198	**1.8**	3
1	31915904-31916940	1036	**1.6**	2
1	246705249-246804070	98821	**1.6**	28
10	48167566-49146476	978910	**1.5**	209
1	170034692-170335752	301060	**1.5**	204
1	179718880-180579380	860500	**1.5**	649
1	51267389-53775502	2508113	**1.5**	1285
1	241731166-242192377	461211	**1.5**	343
19	53508644-54098319	589675	**1.5**	203
19	52005247-52736055	730808	**1.5**	286
22	28672191-29615570	943379	**1.5**	583
1	100144830-100626343	481513	**1.5**	260
2	44574966-45151080	576114	**1.5**	422
2	71683290-72963372	1280082	**1.5**	643
1	117437428-118814926	1377498	**1.5**	811
1	230529647-230689742	160095	**1.5**	176
1	244887695-245574531	686836	**1.5**	439
2	15948138-17075031	1126893	**1.5**	800
1	34380928-36661125	2280197	**1.5**	1211
X	103855195-104776367	921172	**1.5**	506
10	69123920-70080227	956307	**1.5**	485
2	41944415-42694094	749679	**1.5**	500
2	61543230-62329796	786566	**1.5**	366
1	165909616-166325398	415782	**1.5**	262
1	61551255-62551299	1000044	**1.5**	815
1	85121772-86552686	1430914	**1.5**	1019
1	239312051-240474093	1162042	**1.5**	932
19	48879346-49557541	678195	**1.5**	476
2	53301281-54244480	943199	**1.5**	665
10	89421040-89622383	201343	**1.5**	174
X	96495212-96823336	328124	**1.5**	155
2	130385289-131309515	924226	**1.5**	240
1	59350347-59955799	605452	**1.5**	418
8	102698572-103115567	416995	**1.5**	315
10	4836866-6131965	1295099	**1.5**	981
22	14432528-14805217	372689	**1.5**	71
1	89245350-89629944	384594	**1.5**	241
2	14611378-14921304	309926	**1.5**	237
2	30119449-31038526	919077	**1.5**	606
1	112782935-116515302	3732367	**1.5**	2366
5	15776346-15981658	205312	**1.5**	122
10	51119375-51716809	597434	**1.5**	157
1	225535628-226958854	1423226	**1.5**	710
1	94312915-95522944	1210029	**1.4**	949
2	27545538-28224796	679258	**1.4**	330

1	221467647-221696077	228430	**1.4**	158
10	6553345-6689941	136596	**1.4**	103
10	45926794-47118840	1192046	**1.4**	307
5	16719407-17206001	486594	**1.4**	287
1	64556203-65018484	462281	**1.4**	343
1	46922381-47399833	477452	**1.4**	242
1	93170859-93900356	729497	**1.4**	365
1	206721575-208032210	1310635	**1.4**	1082
1	31917610-33277595	1359985	**1.4**	547
1	179076604-179501358	424754	**1.4**	308
1	54603144-55255325	652181	**1.4**	452
1	197632736-198736692	1103956	**1.4**	787
1	24805320-26918842	2113522	**1.4**	1100
1	111120414-112114074	993660	**1.4**	817
1	110036555-110572117	535562	**1.4**	358
1	216629371-217228400	599029	**1.4**	423
2	84688184-85304325	616141	**1.4**	340
1	65199673-65648439	448766	**1.4**	294
18	46819769-46820024	255	**1.4**	4
1	167132622-167464968	332346	**1.4**	310
1	44925703-45718061	792358	**1.4**	384
19	41291642-44541942	3250300	**1.4**	1662
1	45979296-46459374	480078	**1.4**	243
1	242889399-243161992	272593	**1.4**	130
1	55906056-57822727	1916671	**1.4**	1573
2	5405970-6072427	666457	**1.4**	590
19	55285805-55939311	653506	**1.4**	253
10	44987988-45576787	588799	**1.4**	284
1	108884941-110035094	1150153	**1.4**	625
8	105066086-105417726	351640	**1.4**	213
19	56275428-59685525	3410097	**1.4**	2322
2	37949776-39045486	1095710	**1.4**	754
9	44994996-65336138	20341142	**1.4**	86
1	218694464-219016303	321839	**1.4**	186
10	61358959-61484423	125464	**1.4**	119
1	96267086-97044600	777514	**1.4**	471
2	51052476-51493426	440950	**1.4**	313
5	5917529-6664757	747228	**1.4**	651
1	42461553-44505044	2043491	**1.4**	1189
8	102587767-102687413	99646	**1.4**	90
1	177439579-177544139	104560	**1.4**	105
1	162796246-163095552	299306	**1.4**	246
22	34249392-34458396	209004	**1.4**	155
1	102144629-102362899	218270	**1.4**	172
5	6665906-7685035	1019129	**1.4**	839
1	230836901-231057128	220227	**1.4**	152
5	14372158-15384304	1012146	**1.4**	703
1	200114411-200476356	361945	**1.4**	216
2	2784-291380	288596	**1.4**	192
10	70547234-70669751	122517	**1.4**	76
10	47151388-48097459	946071	**1.4**	185
1	222307562-222689901	382339	**1.4**	238
5	10295162-11188399	893237	**1.4**	743
1	80226934-82673852	2446918	**1.4**	1711
6	14971309-26426882	11455573	**1.4**	8430
6	13136318-14822174	1685856	**1.4**	1302
6	26609876-29944615	3334739	**1.4**	1596
2	1006951-2335000	1328049	**1.4**	841
X	104799407-104880542	81135	**1.4**	47
1	171028464-171427555	399091	**1.4**	252
10	63944183-64054521	110338	**1.4**	72
1	172524588-172927174	402586	**1.4**	194
18	59272620-63443012	4170392	**1.4**	2954
1	97967505-98302175	334670	**1.4**	156
10	62057206-62214515	157309	**1.4**	113
5	647083-4609854	3962771	**1.4**	3186
6	30003952-31872878	1868926	**1.4**	950
10	81006701-81562784	556083	**1.4**	51
5	11237831-14122216	2884385	**1.4**	1882
10	59972521-60059007	86486	**1.4**	66
1	77144995-77335876	190881	**1.4**	108
6	733169-8551092	7817923	**1.3**	6550
8	99766247-100157433	391186	**1.3**	134
8	103895213-103981590	86377	**1.3**	47
18	68673407-76116029	7442622	**1.3**	5539
X	143031241-148451627	5420386	**1.3**	3835
10	7777857-9206351	1428494	**1.3**	1218

6	9571901-13134175	3562274	**1.3**	2777
18	63469463-68669065	5199602	**1.3**	3940
2	86621903-86943194	321291	**1.3**	130
13	75927360-77609997	1682637	**1.3**	1093
20	240235-401461	161226	**1.3**	101
11	188510-481334	292824	**1.3**	122
7	32994438-38445926	5451488	**1.3**	3938
5	17703686-21374946	3671260	**1.3**	2293
17	42395116-43073112	677996	**1.3**	292
17	7518132-19454355	11936223	**1.3**	7767
1	165509527-165621816	112289	**1.3**	48
X	148850985-154632871	5781886	**1.3**	3247
7	13782371-27662790	13880419	**1.3**	10933
16	71670952-73803543	2132591	**1.3**	1485
7	8420719-11673553	3252834	**1.3**	2681
20	14639122-15153396	514274	**1.2**	470
7	38455202-51761516	13306314	**1.2**	9068
16	73809807-77620657	3810850	**1.2**	3738
11	118996915-134449982	15453067	**1.2**	11840
7	27667007-32984213	5317206	**1.2**	3924
7	3167816-4471001	1303185	**1.2**	1152
11	104463376-118990424	14527048	**1.2**	9867
7	4478405-8420420	3942015	**1.2**	2409
7	51811236-54477377	2666141	**1.2**	2020
7	1738239-3163095	1424856	**1.2**	756
13	90498691-109098163	18599472	**1.2**	14084
X	148493841-148617321	123480	**1.2**	78
14	55285230-59076441	3791211	**1.2**	2755
17	19458875-20166355	707480	**1.2**	403
16	45021283-60442177	15420894	**1.2**	10420
10	39116227-44115532	4999305	**1.2**	1433
16	86673631-88815036	2141405	**1.2**	871
16	77722352-86667923	8945571	**1.2**	8874
1	101099302-101158767	59465	**1.2**	29
6	87475556-95340182	7864626	**1.2**	5268
22	35002522-35221804	219282	**1.2**	137
13	109101863-114126499	5024636	**1.2**	2976
13	77614025-84772150	7158125	**1.2**	4679
7	292305-1734526	1442221	**1.2**	388
6	80951883-87452286	6500403	**1.2**	4087
7	11691713-13734022	2042309	**1.2**	1645
6	95351785-126717047	31365262	**1.1**	19503
6	126721144-144586181	17865037	**1.1**	11716
13	84839694-90492717	5653023	**1.1**	3332
19	60094170-63789666	3695496	**1.1**	2315
19	17445908-20476510	3030602	**1.1**	1454
22	26644387-26712390	68003	**1.1**	37
14	18072124-20124317	2052193	**1.1**	694
9	98117119-98207740	90621	**1.1**	46
4	2281-56719	54438	**1.1**	63
9	35535830-38430946	2895116	**1.0**	1876
11	42208344-50359444	8151100	**1.0**	4681
