# Supplementary material for: Multi-Platform Whole-Genome Microarray Analyses Refine the Epigenetic Signature of Breast Cancer Metastasis with Gene Expression and Copy Number
Source: PLoS One. 2010 Jan 13;5(1):e8665. doi: 10.1371/journal.pone.0008665 (PMC2801616; doi:10.1371/journal.pone.0008665)
Supplement: Table S3 — Regions showing a decrease in copy number, (468LN relative to 468GFP). Included are associated genes contained within the regions. (0.05 MB PDF) [file pone.0008665.s004.pdf]

Supplemental Table 3: Regions showing an Decrease in copy number, (468GFP-LN relative to 468GFF

#chromosome	region	length	**average Copy number**	# probesets
1	31915904-31916940	1036	1.63	2
1	246705249-246804070	98821	1.55	28
10	48167566-49146476	978910	1.52	209
1	170034692-170335752	301060	1.50	204
1	179718880-180579380	860500	1.50	649
1	51267389-53775502	2508113	1.49	1285
1	241731166-242192377	461211	1.49	343
19	53508644-54098319	589675	1.49	203
19	52005247-52736055	730808	1.49	286
22	28672191-29615570	943379	1.49	583
1	100144830-100626343	481513	1.48	260
2	44574966-45151080	576114	1.48	422
2	71683290-72963372	1280082	1.48	643
1	117437428-118814926	1377498	1.48	811
1	230529647-230689742	160095	1.48	176
1	244887695-245574531	686836	1.48	439
2	15948138-17075031	1126893	1.47	800
1	34380928-36661125	2280197	1.47	1211
X	103855195-104776367	921172	1.47	506
10	69123920-70080227	956307	1.47	485
2	41944415-42694094	749679	1.47	500
2	61543230-62329796	786566	1.47	366
1	165909616-166325398	415782	1.47	262
1	61551255-62551299	1000044	1.47	815
1	85121772-86552686	1430914	1.47	1019
1	239312051-240474093	1162042	1.47	932
19	48879346-49557541	678195	1.47	476
2	53301281-54244480	943199	1.47	665
10	89421040-89622383	201343	1.46	174
X	96495212-96823336	328124	1.46	155
2	130385289-131309515	924226	1.46	240
1	59350347-59955799	605452	1.46	418
8	102698572-103115567	416995	1.46	315
10	4836866-6131965	1295099	1.46	981
22	14432528-14805217	372689	1.46	71
1	89245350-89629944	384594	1.46	241
2	14611378-14921304	309926	1.46	237
2	30119449-31038526	919077	1.45	606
1	112782935-116515302	3732367	1.45	2366
5	15776346-15981658	205312	1.45	122
10	51119375-51716809	597434	1.45	157
1	225535628-226958854	1423226	1.45	710
1	94312915-95522944	1210029	1.45	949
2	27545538-28224796	679258	1.45	330
1	221467647-221696077	228430	1.44	158
10	6553345-6689941	136596	1.44	103
10	45926794-47118840	1192046	1.44	307
5	16719407-17206001	486594	1.44	287
1	64556203-65018484	462281	1.44	343
1	46922381-47399833	477452	1.44	242
1	93170859-93900356	729497	1.44	365
1	206721575-208032210	1310635	1.44	1082
1	31917610-33277595	1359985	1.44	547
1	179076604-179501358	424754	1.44	308
1	54603144-55255325	652181	1.44	452
1	197632736-198736692	1103956	1.44	787
1	24805320-26918842	2113522	1.44	1100
1	111120414-112114074	993660	1.44	817
1	110036555-110572117	535562	1.43	358
1	216629371-217228400	599029	1.43	423
2	84688184-85304325	616141	1.43	340
1	65199673-65648439	448766	1.43	294
18	46819769-46820024	255	1.43	4
1	167132622-167464968	332346	1.43	310
1	44925703-45718061	792358	1.43	384
19	41291642-44541942	3250300	1.43	1662
1	45979296-46459374	480078	1.43	243
1	242889399-243161992	272593	1.43	130

1	55906056-57822727	1916671	1.42	1573
2	5405970-6072427	666457	1.42	590
19	55285805-55939311	653506	1.42	253
10	44987988-45576787	588799	1.42	284
1	108884941-110035094	1150153	1.42	625
8	105066086-105417726	351640	1.42	213
19	56275428-59685525	3410097	1.42	2322
2	37949776-39045486	1095710	1.42	754
9	44994996-65336138	20341142	1.42	86
1	218694464-219016303	321839	1.42	186
10	61358959-61484423	125464	1.41	119
1	96267086-97044600	777514	1.41	471
2	51052476-51493426	440950	1.41	313
5	5917529-6664757	747228	1.41	651
1	42461553-44505044	2043491	1.41	1189
8	102587767-102687413	99646	1.41	90
1	177439579-177544139	104560	1.41	105
1	162796246-163095552	299306	1.41	246
22	34249392-34458396	209004	1.41	155
1	102144629-102362899	218270	1.40	172
5	6665906-7685035	1019129	1.40	839
1	230836901-231057128	220227	1.40	152
5	14372158-15384304	1012146	1.40	703
1	200114411-200476356	361945	1.40	216
2	2784-291380	288596	1.40	192
10	70547234-70669751	122517	1.39	76
10	47151388-48097459	946071	1.39	185
1	222307562-222689901	382339	1.39	238
5	10295162-11188399	893237	1.39	743
1	80226934-82673852	2446918	1.38	1711
6	14971309-26426882	11455573	1.38	8430
6	13136318-14822174	1685856	1.38	1302
6	26609876-29944615	3334739	1.38	1596
2	1006951-2335000	1328049	1.38	841
X	104799407-104880542	81135	1.37	47
1	171028464-171427555	399091	1.37	252
10	63944183-64054521	110338	1.37	72
1	172524588-172927174	402586	1.37	194
18	59272620-63443012	4170392	1.37	2954
1	97967505-98302175	334670	1.37	156
10	62057206-62214515	157309	1.37	113
5	647083-4609854	3962771	1.37	3186
6	30003952-31872878	1868926	1.37	950
10	81006701-81562784	556083	1.37	51
5	11237831-14122216	2884385	1.35	1882
10	59972521-60059007	86486	1.35	66
1	77144995-77335876	190881	1.35	108
6	733169-8551092	7817923	1.35	6550
8	99766247-100157433	391186	1.34	134
8	103895213-103981590	86377	1.33	47
18	68673407-76116029	7442622	1.33	5539
X	143031241-148451627	5420386	1.33	3835
10	7777857-9206351	1428494	1.33	1218
6	9571901-13134175	3562274	1.33	2777
18	63469463-68669065	5199602	1.32	3940
2	86621903-86943194	321291	1.32	130
13	75927360-77609997	1682637	1.31	1093
20	240235-401461	161226	1.30	101
11	188510-481334	292824	1.30	122
7	32994438-38445926	5451488	1.30	3938
5	17703686-21374946	3671260	1.29	2293
17	42395116-43073112	677996	1.28	292
17	7518132-19454355	11936223	1.28	7767
1	165509527-165621816	112289	1.27	48
X	148850985-154632871	5781886	1.27	3247
7	13782371-27662790	13880419	1.26	10933
16	71670952-73803543	2132591	1.26	1485
7	8420719-11673553	3252834	1.26	2681
20	14639122-15153396	514274	1.23	470
7	38455202-51761516	13306314	1.23	9068
16	73809807-77620657	3810850	1.23	3738
11	118996915-134449982	15453067	1.23	11840

7	27667007-32984213	5317206	1.23	3924
7	3167816-4471001	1303185	1.23	1152
11	104463376-118990424	14527048	1.22	9867
7	4478405-8420420	3942015	1.22	2409
7	51811236-54477377	2666141	1.21	2020
7	1738239-3163095	1424856	1.21	756
13	90498691-109098163	18599472	1.21	14084
X	148493841-148617321	123480	1.20	78
14	55285230-59076441	3791211	1.20	2755
17	19458875-20166355	707480	1.20	403
16	45021283-60442177	15420894	1.20	10420
10	39116227-44115532	4999305	1.20	1433
16	86673631-88815036	2141405	1.19	871
16	77722352-86667923	8945571	1.19	8874
1	101099302-101158767	59465	1.18	29
6	87475556-95340182	7864626	1.18	5268
22	35002522-35221804	219282	1.17	137
13	109101863-114126499	5024636	1.17	2976
13	77614025-84772150	7158125	1.17	4679
7	292305-1734526	1442221	1.17	388
6	80951883-87452286	6500403	1.17	4087
7	11691713-13734022	2042309	1.15	1645
6	95351785-126717047	31365262	1.14	19503
6	126721144-144586181	17865037	1.14	11716
13	84839694-90492717	5653023	1.14	3332
19	60094170-63789666	3695496	1.13	2315
19	17445908-20476510	3030602	1.12	1454
22	26644387-26712390	68003	1.10	37
14	18072124-20124317	2052193	1.08	694
9	98117119-98207740	90621	1.07	46
4	2281-56719	54438	1.06	63
9	35535830-38430946	2895116	1.04	1876
11	42208344-50359444	8151100	1.03	4681
20	3926209-4062913	136704	0.96	113
11	55119916-55132856	12940	0.96	9
1	37192428-41960253	4767825	0.95	2763
9	34358097-35534057	1175960	0.92	622
12	108693615-108884864	191249	0.89	99
18	1543-15392408	15390865	0.88	10622
19	9681700-10738387	1056687	0.74	454
11	30136885-38817927	8681042	0.74	6290
4	70162241-70183466	21225	0.70	14
3	197261134-197261261	127	0.70	3
21	9758742-14346239	4587497	0.70	569
11	28816432-30128490	1312058	0.68	818
11	25919584-28807470	2887886	0.67	1916
7	56703960-57242577	538617	0.62	127
21	14995007-16486581	1491574	0.57	1063
4	70189134-70209004	19870	0.50	9
21	14457461-14687786	230325	0.50	162
7	55943236-56255432	312196	0.50	137
21	14380475-14413121	32646	0.46	20
21	14751128-14881879	130751	0.44	115
1	150838805-150852862	14057	0.42	16
21	14413165-14457314	44149	0.38	28
7	55800568-55942420	141852	0.35	66
Y	24578356-24697220	118864	0.34	30
Y	22191306-22529167	337861	0.33	96
Y	4920268-5117638	197370	0.33	45
Y	15417161-15642352	225191	0.32	120
Y	9916486-10061768	145282	0.32	56
Y	169542-2875303	2705761	0.32	94
Y	3503397-3606615	103218	0.32	19
Y	20276103-20575181	299078	0.31	137
Y	15192157-15351018	158861	0.31	98
Y	5555743-5886923	331180	0.31	77
Y	21233259-21424276	191017	0.30	113
Y	26136561-26502815	366254	0.30	154
Y	22752065-23922949	1170884	0.30	191
Y	18600026-18918077	318051	0.30	136
Y	18472916-18574836	101920	0.30	43
Y	12881420-13984995	1103575	0.30	557

Y	19261735-19779256	517521	0.30	263
Y	14091850-14851426	759576	0.30	372
Y	7162192-7503949	341757	0.29	173
Y	22057829-22189203	131374	0.29	57
Y	25367413-26133162	765749	0.29	137
Y	25078106-25358503	280397	0.28	41
Y	18247793-18472221	224428	0.28	104
Y	20182342-20275835	93493	0.28	40
Y	6791267-7155456	364189	0.28	190
Y	5898465-6232905	334440	0.28	127
Y	19112261-19261722	149461	0.27	84
Y	5119423-5551644	432221	0.27	86
Y	21119798-21232123	112325	0.27	39
Y	13985947-14089104	103157	0.26	55
Y	15094990-15190450	95460	0.26	40
Y	22587206-22751738	164532	0.25	45
Y	2878038-2931352	53314	0.25	20
Y	15353654-15414397	60743	0.24	34
1	246815817-246861087	45270	0.23	30
1	110035754-110036151	397	0.21	4
11	55159390-55187640	28250	0.21	19
10	72269865-72272401	2536	0.19	3
19	40541333-40548543	7210	0.18	11
7	32985593-32991713	6120	0.17	3
10	45613625-45905767	292142	0.13	3
7	54481855-55791075	1309220	0.11	1089
14	73076460-73090732	14272	0.11	8
14	71806722-71806932	210	0.11	5
4	70184239-70189096	4857	0.10	4
5	15773075-15773597	522	0.07	9
