# Supplementary material for: Multi-Platform Whole-Genome Microarray Analyses Refine the Epigenetic Signature of Breast Cancer Metastasis with Gene Expression and Copy Number
Source: PLoS One. 2010 Jan 13;5(1):e8665. doi: 10.1371/journal.pone.0008665 (PMC2801616; doi:10.1371/journal.pone.0008665)
Supplement: Table S4 — Genes significantly increased in expression, 468LN vs 468GFP. Expression microarray (HGU133 Plus_2) data were prefiltered to remove genes changing less than 2 fold, and an ANOVA was run to determine significant (p<0.05) changers. A multiple testing correction using the algorithm of Benjamini and Hochberg was used to reduce the false discovery rate. (0.46 MB PDF) [file pone.0008665.s005.pdf]

Supplemental Table 4: Genes significantly Increased in expression, LN vs GFP

Cell line ()*	468GFP				468GFP-LN					
Systematic	Ratio to GF	t-test P-value	Flags	Raw	**Ratio to GFP**	-test P-valu	Flags	Raw	Common	Map
210546_x_at	1.00	0.95	A	0.93	**2491.46**	0.00	P	2287.83	CTAG1	xq28
211674_x_at	0.98	0.91	A	1.80	**1495.97**	0.00	P	2651.93	CTAG1	xq28
214475_x_at	0.99	0.92	A	1.47	**1188.55**	0.00	P	1724.33	CAPN3	5q15.1-q21.1
215733_x_at	0.99	0.94	A	2.73	**1138.19**	0.00	P	3053.33	CTAG2	xq28
231307_at	0.92	0.80	A	1.13	**1074.00**	0.00	P	1186.33	MGC62094	xp11.22
1555504_at	0.97	0.87	A	0.57	**1039.49**	0.00	P	577.93	TYR	11q14-q21
224964_s_at	0.82	0.69	A	1.73	**617.13**	0.00	P	1063.23	GNG2	14q21
203603_s_at	0.83	0.71	A	1.23	**615.83**	0.00	P	752.87	ZFHX1B	2q22
209170_s_at	1.00	0.97	A	2.33	**595.83**	0.00	P	1367.93	GPM6B	xp22.2
200665_s_at	0.64	0.57	A	4.20	**554.03**	0.00	P	2317.60	SPARC	5q31.3-q32
205433_at	0.69	0.66	A	4.87	**498.35**	0.00	P	2372.63	BCHE	3q26.1-q26.2
211890_x_at	0.90	0.80	A	2.90	**492.96**	0.00	P	1428.30	CAPN3	5q15.1-q21.1
209686_at	0.96	0.86	A	3.93	**488.91**	0.00	P	1876.60	S100B	21q22.3
206332_s_at	0.99	0.94	A	4.17	**486.59**	0.00	P	1999.83	IFI16	1q22
206070_s_at	0.92	0.81	A	2.50	**471.97**	0.00	P	1154.53	EPHA3	3p11.2
206645_s_at	0.92	0.80	A	1.83	**468.42**	0.00	P	849.53	NR0B1	xp21.3-p21.2
228375_at	0.91	0.80	A	3.03	**465.21**	0.00	P	1403.20	IGSF11	3q13.33
205029_s_at	0.78	0.70	A	4.47	**461.89**	0.00	P	2006.57	FABP7	6q22-q23
235944_at	0.84	0.72	A	1.80	**457.63**	0.00	P	825.13	FIBL-6	1q25.3
201744_s_at	0.59	0.54	A	1.00	**457.00**	0.00	P	438.03	LUM	12q21.3-q22
47550_at	0.95	0.84	A	1.80	**418.23**	0.00	P	745.50	LZTS1	8p22
205862_at	0.66	0.57	A	2.37	**386.67**	0.00	P	877.73	GREB1	2p25.1
217339_x_at	1.00	0.97	A	5.23	**377.92**	0.00	P	1944.60	CTAG1	xq28
1555505_a_at	0.73	0.62	A	7.67	**377.78**	0.00	P	2884.43	TYR	11q14-q21
203132_at	0.56	0.55	A	1.50	**375.88**	0.00	P	542.33	RB1	13q14.2
1559827_at	0.99	0.92	A	0.83	**353.78**	0.00	P	290.37		3p14.1
219279_at	0.99	0.94	A	1.40	**353.37**	0.00	P	487.43	DOCK10	2q36.3
205694_at	0.99	0.94	P,A	13.97	**318.64**	0.00	P	4398.73	TYRP1	9p23
212526_at	0.91	0.79	A	2.73	**317.14**	0.00	P	862.00	SPG20	13q13.2
207397_s_at	0.97	0.87	A	1.40	**309.97**	0.00	P	423.47	HOXD13	2q31.1
232504_at	0.97	0.87	A	2.50	**298.52**	0.00	P	739.00	LOC285628	5q34
219911_s_at	0.98	0.90	A	6.30	**296.53**	0.00	P	1829.13	SLCO4A1	20q13.33
1569100_a_at	0.99	0.94	A	0.43	**294.59**	0.00	P	126.03		
208965_s_at	0.83	0.73	A	3.00	**285.51**	0.00	P	836.53	IFI16	1q22
204237_at	0.93	0.82	A	5.00	**275.46**	0.00	P	1346.33	GULP1	2q32.3-q33
209167_at	0.73	0.62	A	6.77	**270.99**	0.00	P	1824.07	GPM6B	Xp22.2
231899_at	0.83	0.72	A	1.87	**270.45**	0.00	P	493.90	KIAA1726	11q23.1
208986_at	0.97	0.87	A	7.77	**257.20**	0.00	P	1968.03	TCF12	15q21
206427_s_at	0.85	0.75	A	20.07	**255.21**	0.00	P	5109.23	MLANA	9p24.1
205893_at	0.74	0.68	A	1.43	**226.75**	0.00	P	319.37	NLGN1	3q26.32
209031_at	0.99	0.94	A	3.97	**224.11**	0.00	P	873.43	IGSF4	11q23.2
207337_at	0.88	0.75	A	6.17	**222.51**	0.00	P	1361.47	CTAG2	xq28
226333_at	0.98	0.89	A	2.47	**218.06**	0.00	P	529.63	IL6R	1q21
205337_at	1.00	0.99	A	19.90	**211.08**	0.00	P	4130.43	DCT	13q32
213139_at	0.91	0.80	A	8.97	**207.57**	0.00	P	1825.87	SNAI2	8q11
214321_at	0.79	0.71	M,A	12.33	**198.56**	0.00	P	2443.90	NOV	8q24.1
238041_at	0.68	0.60	A	1.47	**196.93**	0.00	P	286.60		
206696_at	0.96	0.86	A	7.73	**182.45**	0.00	P	1396.40	GPR143	xp22.3
218474_s_at	0.96	0.86	A	5.20	**181.35**	0.00	P	927.87	KCTD5	16p13.3
203440_at	0.85	0.74	A	3.30	**171.36**	0.00	P	549.63	CDH2	18q11.2
215913_s_at	0.99	0.93	A	1.70	**168.40**	0.00	P	287.50	GULP1	2q32.3-q33
209524_at	0.97	0.89	A	1.57	**165.45**	0.00	P	256.40	HDGFRP3	15q11.2
217963_s_at	0.89	0.76	A	20.07	**161.31**	0.00	P	3202.83	NGFRAP1	xq22.2
206426_at	0.77	0.70	P,A	13.83	**159.15**	0.00	P	2137.87	MLANA	9p24.1
204591_at	0.78	0.66	A	4.67	**158.91**	0.00	P	739.27	CHL1	3p26.1
220004_at	0.84	0.72	A	2.33	**157.08**	0.00	P	366.20	DDX43	6q12-q13
210198_s_at	0.75	0.64	A	19.93	**153.76**	0.00	P	2950.23	PLP1	xq22
201162_at	1.00	0.98	A	4.80	**153.43**	0.00	P	727.67	IGFBP7	4q12
219042_at	0.96	0.85	A	2.50	**151.23**	0.00	P	375.30	LZTS1	8p22
226785_at	0.72	0.62	A	3.73	**149.69**	0.00	P	554.43	ATP11C	xq27.1
227529_s_at	1.00	0.97	A	1.43	**148.82**	0.00	P	211.10	AKAP12	6q24-q25
209392_at	0.98	0.90	A	11.67	**148.48**	0.00	P	1707.80	ENPP2	8q24.1
221974_at	0.96	0.86	A	0.93	**144.90**	0.00	P	134.40		
216512_s_at	0.95	0.85	A	23.57	**142.47**	0.00	P	3327.97	DCT	13q32
208966_x_at	0.91	0.79	A	13.63	**138.51**	0.00	P	1835.63	IFI16	1q22
225626_at	0.97	0.88	A	1.70	**132.78**	0.00	P	220.77	PAG	8q21.12
205030_at	0.99	0.93	A	39.50	**127.68**	0.00	P	4961.20	FABP7	6q22-q23
221558_s_at	0.90	0.77	A	5.47	**123.96**	0.00	P	657.13	LEF1	4q23-q25
232010_at	0.83	0.73	A	3.57	**122.68**	0.00	P	430.83	FSTL5	4q32.3
210517_s_at	0.96	0.86	A	10.13	**121.95**	0.00	P	1222.80	AKAP12	6q24-q25
217975_at	0.98	0.91	P,M,A	16.87	**119.85**	0.00	P	1988.23	LOC51186	xq22.2
206030_at	0.99	0.95	A	2.00	**117.06**	0.00	P	230.83	ASPA	17pter-p13
214829_at	0.93	0.83	A	1.03	**112.29**	0.00	P	115.67	AASS	7q31.3
1555963_x_at	0.88	0.75	A	3.13	**110.96**	0.00	P	341.50	B3GNT7	2q37.1
241390_at	0.78	0.70	A	2.27	**104.05**	0.00	P	231.97		
201426_s_at	0.99	0.93	A	56.47	**103.67**	0.00	P	5798.10	VIM	10p13
232204_at	0.98	0.90	A	2.57	**100.98**	0.00	P	254.63	EBF	5q34
213638_at	0.96	0.86	A	13.40	**99.06**	0.00	P	1306.23	PHACTR1	6p23
223741_s_at	0.90	0.76	A	8.20	**98.81**	0.00	P	833.23	TTYH2	17q24
232136_s_at	0.90	0.77	A	0.73	**98.52**	0.00	P	71.33	CORTBP2	7q31
223629_at	0.85	0.73	A	3.80	**96.95**	0.00	P	368.33	PCDHB5	5q31
224397_s_at	0.98	0.90	A	2.50	**92.54**	0.00	P	229.03	ARG99	12p11.23
236681_at	0.99	0.95	A	8.00	**92.35**	0.00	P	724.53	HOXD13	2q31.1
217858_s_at	0.99	0.95	A	10.53	**90.11**	0.00	P	933.30	ARMCX3	q21.33-q22.2
223975_at	0.85	0.74	A	5.73	**90.08**	0.00	P	500.90	TRIM51	11q12.1
225566_at	0.97	0.88	A	6.33	**89.80**	0.00	P	555.93	NRP2	2q33.3
213624_at	0.92	0.80	A	6.20	**89.64**	0.00	P	551.27	SMPDL3A	6q22.32
230272_at	0.98	0.91	A	1.07	**86.66**	0.00	P	90.47		
209781_s_at	0.97	0.87	A	5.77	**84.17**	0.00	P	478.43	KHDRBS3	8q24.2
204955_at	0.98	0.90	P	25.40	**84.02**	0.00	P	2094.73	SRPX	xp21.1
213417_at	0.99	0.93	A	4.53	**82.02**	0.00	P	366.70	TBX2	17q23
213943_at	0.88	0.77	A	1.63	**81.50**	0.00	P	132.87	TWIST1	7p21.2
40560_at	1.00	0.96	A	7.93	**81.37**	0.00	P	633.47	TBX2	17q23
209843_s_at	0.99	0.95	A	11.43	**81.25**	0.00	P	919.83	SOX10	22q13.1
227522_at	0.71	0.60	A	4.87	**80.34**	0.00	P	392.80	LOC134147	5p15.31
224210_s_at	1.00	0.96	A	2.00	**78.25**	0.00	P	153.73	PXMP4	20q11.22
206071_s_at	0.84	0.71	A	3.20	**77.99**	0.00	P	247.53	EPHA3	3p11.2
202283_at	0.97	0.87	A	14.53	**77.97**	0.00	P	1106.50	SERPINF1	17p13.1
206630_at	0.98	0.90	A	60.70	**77.97**	0.00	P	4639.67	TYR	11q14-q21
237070_at	0.95	0.85	A	2.97	**76.62**	0.00	P	222.90	TRPM1	15q13-q14
206617_s_at	0.99	0.93	A	7.80	**76.47**	0.00	P	587.73	RENBP	xq28
225945_at	0.86	0.74	A	6.43	**76.33**	0.00	P	488.70	VIK	7q22.1
205945_at	0.77	0.67	A	5.80	**75.38**	0.00	P	437.57	IL6R	1q21
213262_at	0.97	0.88	P,A	8.23	**75.26**	0.00	P	612.67	SACS	13q12
225599_s_at	0.88	0.77	A	6.80	**72.16**	0.00	P	478.87	LOC286144	8q22.1
214434_at	0.95	0.84	A	5.37	**71.88**	0.00	P	375.90	HSPA12A	10q26.12
202219_at	0.95	0.85	A	27.83	**69.88**	0.00	P	1910.77	SLC6A8	xq28
219872_at	0.88	0.75	A	5.80	**69.25**	0.00	P	391.83	KFZp434L1	4q32.1
211844_s_at	0.95	0.83	A	2.57	**67.51**	0.00	P	169.57	NRP2	2q33.3
220534_at	1.00	0.96	A	20.60	**67.09**	0.00	P	1359.07	TRIM48	11q12.1
227297_at	0.85	0.72	A	2.77	**66.96**	0.00	P	185.17	ITGA9	3p21.3
237991_at	0.91	0.79	A	2.37	**65.21**	0.00	P	150.17		
209842_at	0.87	0.74	A	35.77	**64.70**	0.00	P	2245.87	SOX10	22q13.1
230047_at	0.78	0.66	A	4.73	**63.46**	0.00	P	299.33	FLJ32810	1q22.1-q22.2
207253_s_at	0.97	0.88	A	3.77	**62.87**	0.00	P	232.40	UBN1	16p13.3
223204_at	1.00	0.99	P	38.47	**61.87**	0.00	P	2342.10	KFZp434L1	4q32.1
212915_at	0.73	0.62	A	4.13	**61.87**	0.00	P	247.43	PDZRN3	3p14.1
220116_at	0.95	0.84	A	2.03	**60.48**	0.00	P	119.90	KCNN2	5q22.3
207336_at	0.99	0.92	A	1.37	**60.44**	0.00	P,A	83.47	SOX5	12p12.1
241457_at	0.81	0.68	A	2.60	**60.41**	0.00	P	158.87		
243000_at	0.97	0.89	A	2.67	**59.94**	0.00	P	156.77	CDK6	7q21-q22
228038_at	0.70	0.61	A	2.00	**59.74**	0.00	P	119.97	SOX2	3q26.3-q27
204271_s_at	0.97	0.89	P,M	21.87	**59.40**	0.00	P	1276.10	EDNRB	13q22
212636_at	0.80	0.72	A	8.40	**59.08**	0.00	P	486.00	QKI	6q26-27
213416_at	0.89	0.77	A	7.63	**58.69**	0.00	P	446.10	ITGA4	2q31-q32
222171_s_at	0.98	0.90	A	3.20	**58.05**	0.00	P	182.33	PKNOX2	11q24
1558066_s_at	0.96	0.86	A	11.10	**57.57**	0.00	P	628.83	TBC1D16	17q25.3
227503_at	0.87	0.74	A	1.37	**57.54**	0.00	P	78.30		

236038_at	0.97	0.87	A	0.63	**57.26**	0.00	P	35.50		
227655_at	0.86	0.74	A	3.07	**57.04**	0.00	P	169.60		
229430_at	0.77	0.66	A	3.93	**56.93**	0.00	P	224.57	MGC33510	8q12.3
204466_s_at	0.95	0.84	A	37.50	**56.84**	0.00	P	2105.37	SNCA	4q21
206233_at	0.78	0.66	A	6.10	**56.83**	0.00	P	345.00	B4GALT6	18q11
209859_at	0.96	0.87	A	10.83	**56.10**	0.00	P	604.30	TRIM9	14q22.1
205269_at	0.95	0.84	P,A	7.43	**55.65**	0.00	P	403.20	LCP2	5q33.1-qter
241789_at	0.77	0.65	A	1.13	**55.57**	0.00	P,A	63.50	RBMS3	3p24-p23
226771_at	0.79	0.66	A	6.37	**55.43**	0.00	P	341.80	ATP8B2	1q22
204627_s_at	0.90	0.78	A	2.70	**53.12**	0.00	P	142.93	ITGB3	17q21.32
226707_at	0.98	0.90	A	6.53	**52.98**	0.00	P	342.23	PP3856	8q24.3
204736_s_at	0.81	0.72	A	2.13	**52.78**	0.00	P	114.43	CSPG4	15q23
229222_at	0.97	0.88	A	7.50	**52.42**	0.00	P	387.40	FLJ21963	12q21.31
235127_at	0.95	0.84	A	2.20	**52.12**	0.00	P	114.20	PMP2	8q21.3-q22.1
211682_x_at	0.98	0.91	A	4.13	**51.18**	0.00	P,M,A	212.67	UGT2B28	4q13.3
231666_at	0.85	0.74	A	11.33	**51.10**	0.00	P	569.50	PAX3	2q35
228214_at	0.94	0.83	A	7.53	**50.45**	0.00	P	379.13		
1561789_at	0.79	0.71	A	1.90	**50.31**	0.00	P	95.37		
1554726_at	0.88	0.75	A	1.53	**50.22**	0.00	P	74.80	VIK	7q22.1
229225_at	0.81	0.68	A	11.40	**48.65**	0.00	P	536.13	NRP2	2q33.3
210944_s_at	1.00	0.98	P	46.60	**48.47**	0.00	P	2222.37	CAPN3	15q15.1-q21.1
226322_at	0.97	0.87	A	9.10	**48.46**	0.00	P	434.50	ARG99	12p11.23
215043_s_at	0.94	0.82	A	1.17	**48.23**	0.00	P	55.73	LOC153561	5q13.2
223595_at	0.93	0.83	A	8.40	**48.18**	0.00	P	402.33	AD031	11q22.2
205338_s_at	1.00	0.97	P,A	90.73	**48.15**	0.00	P	4303.33	DCT	13q32
1556331_a_at	0.79	0.71	A	1.87	**46.12**	0.00	P,M	85.13		
242690_at	0.91	0.78	A	1.53	**45.96**	0.00	P,A	69.50	ATP11C	xq27.1
243481_at	0.84	0.72	A	2.63	**45.93**	0.00	P	120.50	RHOJ	14q23.2
209199_s_at	0.81	0.68	A	6.97	**45.86**	0.00	P	318.00	MEF2C	5q14
231337_at	0.76	0.64	A	7.53	**45.67**	0.00	P	344.43		
205498_at	0.95	0.85	A	10.90	**45.22**	0.00	P	488.60	GHR	5p13-p12
1564198_a_at	0.80	0.70	A	6.60	**45.11**	0.00	P	293.50	C10orf90	10q26.2
203402_at	0.72	0.61	A	15.00	**44.59**	0.00	P	644.13	KCNAB2	1p36.3
227812_at	0.99	0.92	A	12.63	**44.06**	0.00	P	545.23	TNFRSF19	13q12.11-q12.3
221524_s_at	0.89	0.78	A	34.50	**43.61**	0.00	P	1472.93	RRAGD	6q15-q16
222444_at	0.96	0.86	A	9.33	**43.54**	0.00	P	401.47	ARMCX3	xq21.33-q22.2
203729_at	0.99	0.91	M,A	33.90	**43.36**	0.00	P	1440.00	EMP3	19q13.3
235593_at	0.79	0.67	A	5.77	**43.35**	0.00	P	248.67	ZFHX1B	2q22
235308_at	0.82	0.70	A	5.10	**43.28**	0.00	P	214.13	ZNF288	3q13.2
228245_s_at	0.99	0.94	P,M	85.33	**43.01**	0.00	P	3611.30	OVOS2	12p11.22
1554018_at	1.00	0.97	P	32.83	**43.00**	0.00	P	1391.00	GPNMB	7p15
207808_s_at	0.98	0.90	A	21.27	**42.96**	0.00	P	894.47	PROS1	3q11.2
244612_at	0.92	0.81	A	0.57	**42.45**	0.00	P	23.93		
243918_at	0.94	0.82	A	2.73	**42.43**	0.00	P	113.03		
228564_at	0.94	0.82	A	8.07	**41.08**	0.00	P	325.93	LOC375295	2q31.2
212624_s_at	0.98	0.89	A	7.00	**41.01**	0.00	P,A	284.77	CHN1	2q31-q32.1
201621_at	0.96	0.86	A	36.47	**40.85**	0.00	P	1491.83	NBL1	1p36.13-p36.11
209200_at	0.90	0.78	A	2.10	**40.75**	0.00	P,A	86.30	MEF2C	5q14
237631_at	0.95	0.83	A	11.93	**40.62**	0.00	P	477.63		
236972_at	0.84	0.71	A	3.87	**40.57**	0.00	P	152.20	RNF28	1p34-p33
218506_x_at	1.00	0.96	A	19.07	**40.53**	0.00	P	762.70	N-PAC	16p13.3
219594_at	0.96	0.86	A	2.67	**40.21**	0.00	P,A	106.63	NINJ2	12p13
202527_s_at	0.91	0.81	A	9.33	**40.09**	0.00	P	365.57	SMAD4	18q21.1
1561757_a_at	0.91	0.78	A	18.30	**39.85**	0.00	P	723.43	LOC283352	12q24.33
213712_at	0.98	0.89	M,A	30.20	**39.28**	0.00	P	1172.07	ELOVL2	6p24.1
202947_s_at	0.97	0.88	A	32.83	**38.67**	0.00	P	1246.27	GYPC	2q14-q21
231597_x_at	0.86	0.75	P,A	32.20	**38.62**	0.00	P	1215.10		
216813_at	0.97	0.87	A	1.93	**38.38**	0.00	P	76.17		
215241_at	0.89	0.77	A	5.90	**38.23**	0.00	P	219.83	TMEM16C	11p14.3
230560_at	0.90	0.79	A	5.43	**37.96**	0.00	P	201.23	STXBP6	14q11.2
223723_at	0.97	0.87	A	3.80	**37.47**	0.00	P	140.63	MFI2	3q28-q29
213435_at	0.93	0.81	P,A	18.07	**37.43**	0.00	P	658.20	SATB2	2q33
238447_at	0.92	0.81	A	8.23	**37.37**	0.00	P	307.67	RBMS3	3p24-p23
204759_at	0.71	0.62	A	4.10	**37.29**	0.00	P	150.77	CHC1L	13q14.3
228695_at	0.80	0.67	A	5.97	**37.19**	0.00	P	221.57	MGC33510	8q12.3
221523_s_at	1.00	0.97	P,A	18.23	**37.02**	0.00	P	663.73	RRAGD	6q15-q16
213222_at	0.95	0.85	A	18.77	**36.79**	0.00	P	678.57	PLCB1	20p12
235334_at	0.96	0.85	A	7.70	**36.06**	0.00	P	270.63	SIAT7C	1p31.1
236465_at	0.89	0.78	A	20.30	**35.80**	0.00	P	709.87	LOC285533	4q31.3
1558937_s_at	0.99	0.94	A	1.23	**35.49**	0.00	P	43.43		8
220425_x_at	0.97	0.87	A	22.77	**35.48**	0.00	P	789.30	ROPN1	3q21.2
234583_at	0.98	0.89	A	2.30	**35.44**	0.00	P	79.77		
224965_at	0.99	0.95	A	13.80	**35.36**	0.00	P	482.20	GNG2	14q21
224228_s_at	0.96	0.86	A	3.77	**35.33**	0.00	P	131.00	PRDM7	16q24.3
206232_s_at	0.97	0.88	A	4.10	**34.74**	0.00	P,A	141.93	B4GALT6	18q11
244387_at	0.95	0.84	A	8.33	**34.64**	0.00	P	286.57		
206560_s_at	0.89	0.78	A	10.17	**34.62**	0.00	P	345.57	MIA	19q13.32-q13.33
237069_s_at	0.85	0.73	A	2.77	**34.55**	0.00	P	95.07	TRPM1	15q13-q14
1555233_at	0.94	0.84	A	5.80	**34.38**	0.00	P	195.73		
226695_at	0.85	0.72	A	10.10	**34.35**	0.00	P	336.13	PRRX1	1q24
230264_s_at	0.99	0.93	P	96.07	**34.34**	0.00	P	3255.73	AP1S2	xp22.31
229001_at	0.96	0.86	A	4.00	**34.34**	0.00	P	137.03	PPP1R3E	14q11.2
218120_s_at	0.92	0.80	M,A	22.80	**33.98**	0.00	P	761.57	HMOX2	16p13.3
205954_at	0.99	0.92	A	27.10	**33.66**	0.00	P	900.13	RXRG	1q22-q23
213158_at	0.95	0.84	M,A	5.23	**33.66**	0.00	P	174.73		
201141_at	1.00	0.96	P	117.23	**33.45**	0.00	P	3864.70	GPNMB	7p15
205884_at	0.89	0.76	A	6.20	**33.40**	0.00	P	205.27	ITGA4	2q31-q32
210078_s_at	0.98	0.91	P,A	18.13	**33.26**	0.00	P	597.50	KCNAB1	3q26.1
230831_at	0.98	0.90	A	5.70	**33.11**	0.00	P	188.17		
226636_at	0.95	0.83	A	4.93	**32.98**	0.01	P,M,A	168.20	PLD1	3q26
201360_at	0.99	0.92	A	18.27	**32.93**	0.00	P	594.03	CST3	20p11.21
227354_at	0.78	0.66	A	5.93	**32.81**	0.00	P	193.73	PAG	8q21.12
228876_at	0.95	0.85	A	13.60	**32.69**	0.00	P	441.23	FLJ22582	22q13.1
240671_at	0.97	0.88	A	1.37	**32.65**	0.00	P	44.43		
204086_at	0.99	0.94	A	20.40	**32.45**	0.00	P	649.83	PRAME	22q11.22
206785_s_at	0.92	0.81	A	1.27	**32.29**	0.00	P,A	41.03	KLRC1	12p13
223501_at	0.96	0.86	P,A	29.03	**32.24**	0.00	P	914.27	TNFSF13B	13q32-34
1555962_at	0.74	0.64	A	10.13	**32.09**	0.00	P	336.60	B3GNT7	2q37.1
202986_at	0.93	0.82	A	10.87	**31.87**	0.00	P	340.63	ARNT2	15q24
207307_at	1.00	0.98	A	2.60	**31.81**	0.00	P	81.50	HTR2C	xq24
225603_s_at	0.97	0.87	A	13.10	**31.77**	0.00	P	409.40	LOC286144	8q22.1
219686_at	0.96	0.85	A	10.53	**31.75**	0.00	P,A	326.40	STK32B	4p16.2
220122_at	0.91	0.78	A	17.33	**31.74**	0.00	P	535.77	FLJ22344	5q15
210202_s_at	0.90	0.77	A	4.47	**31.67**	0.00	P	140.57	BIN1	2q14
213069_at	0.99	0.95	A	12.50	**31.42**	0.00	P	387.77	HEG	3q21.2
241698_at	0.98	0.90	A	3.97	**31.28**	0.00	P	122.63	C2orf11	2q33.1
225382_at	1.00	0.97	A	8.37	**31.26**	0.00	P,A	257.30	ZNF275	xq28
228170_at	0.99	0.95	P,A	16.00	**31.05**	0.00	P	488.60	OLIG1	21q22.11
211708_s_at	0.92	0.81	A	13.30	**30.76**	0.00	P	408.37	SCD	10q23-q24
232881_at	0.82	0.72	A	5.07	**30.60**	0.00	P	152.93	SANG	20q13.32
231067_s_at	0.78	0.66	A	3.37	**30.59**	0.00	P	99.60	AKAP12	6q24-q25
219263_at	0.85	0.74	A	7.23	**30.47**	0.00	P	216.70	RNF128	xq22.3
244163_at	0.90	0.79	A	0.90	**30.29**	0.00	P	27.57	SEMA3A	7p12.1
210839_s_at	0.98	0.89	P,A	24.07	**30.09**	0.00	P	716.17	ENPP2	8q24.1
205542_at	1.00	0.98	A	32.80	**29.90**	0.00	P	965.73	STEAP	7q21
1560813_at	0.96	0.86	A	2.90	**29.84**	0.00	P	85.40		
235740_at	0.96	0.86	A	12.03	**29.84**	0.00	P	355.13	FLJ22344	5q15
238852_at	0.87	0.77	A	3.87	**29.73**	0.00	P	113.87		
239182_at	1.00	0.95	A	1.63	**29.51**	0.00	P,M	47.47	LOC401022	2q31.2
207398_at	0.97	0.88	A	5.23	**29.49**	0.00	P	152.57	HOXD13	2q31.1
203815_at	1.00	0.99	A	6.20	**29.18**	0.00	P	179.53	GSTT1	22q11.23
222877_at	0.98	0.91	A	17.07	**28.80**	0.00	P	486.47	NRP2	2q33.3
203300_x_at	0.98	0.89	P	94.07	**28.79**	0.00	P	2687.10	AP1S2	xp22.31
219682_s_at	0.99	0.93	A	2.60	**28.73**	0.00	P,A	74.27	TBX3	12q24.1
220666_at	0.98	0.90	A	1.67	**28.63**	0.00	P	47.10		
222267_at	1.00	0.97	A	7.93	**28.53**	0.00	P	223.10	FLJ14803	7q32.3
243172_at	0.90	0.79	A	1.47	**28.51**	0.00	P,A	40.93		
216250_s_at	1.00	0.96	P	98.13	**28.48**	0.00	P	2764.57	LPXN	11q12.1
1569796_s_at	0.99	0.91	A	4.70	**28.32**	0.00	P,A	132.90	ATRNL1	10q26
230102_at	0.96	0.86	A	13.47	**28.29**	0.00	P	381.70	ETV5	3q28
239443_at	0.97	0.87	P,A	9.37	**28.18**	0.00	P	257.63	PCDHB6	5q31
1555766_a_at	0.84	0.71	A	6.90	**27.94**	0.00	P	186.40	GNG2	14q21
201163_s_at	0.97	0.89	P	43.57	**27.90**	0.00	P	1190.80	IGFBP7	4q12
209354_at	1.00	0.96	P	34.37	**27.79**	0.00	P	938.77	TNFRSF14	1p36.3-p36.2

220029_at	0.97	0.87	A	28.13	**27.74**	0.00	P	780.60	ELOVL2	6p24.1
227646_at	0.84	0.72	A	5.93	**27.74**	0.00	P	164.63	EBF	5q34
206155_at	0.93	0.80	A	11.33	**27.40**	0.00	P	307.70	ABCC2	10q24
205270_s_at	0.91	0.80	A	15.43	**27.39**	0.00	P	415.33	LCP2	5q33.1-qter
207144_s_at	0.96	0.86	A	7.53	**27.34**	0.00	P	205.23	CITED1	xq13.1
227498_at	0.99	0.94	A	28.53	**27.24**	0.00	P	764.47		
228333_at	0.97	0.87	A	15.40	**27.24**	0.00	P	415.60		
223502_s_at	0.99	0.93	A	31.50	**27.11**	0.00	P	842.00	TNFSF13B	13q32-34
203404_at	0.97	0.89	A	6.27	**26.95**	0.00	P	167.17	ARMCX2	q21.33-q22.2
213843_x_at	0.98	0.90	P,A	60.00	**26.84**	0.00	P	1601.47	SLC6A8	xq28
205334_at	0.99	0.93	A	51.17	**26.82**	0.00	P	1349.57	S100A1	1q21
227826_s_at	0.99	0.94	A	13.00	**26.71**	0.00	P	343.87		
229822_at	0.83	0.70	A	8.03	**26.68**	0.00	P	207.27	PARVG	22q13.2-q13
206042_x_at	0.99	0.92	A	12.90	**26.55**	0.00	P	337.00	SNRPN	15q12
1555527_at	0.96	0.87	A	2.43	**26.55**	0.00	P	64.10	COL9A1	6q12-q14
212190_at	1.00	0.97	P	184.77	**26.41**	0.00	P	4798.63	SERPINE2	2q33-q35
204491_at	0.93	0.82	A	10.40	**26.35**	0.00	P	272.70	PDE4D	5q12
204467_s_at	0.92	0.79	A	23.57	**26.30**	0.00	P	614.50	SNCA	4q21
209030_s_at	0.91	0.79	A	23.07	**26.29**	0.00	P	602.13	IGSF4	11q23.2
1561521_at	0.94	0.84	A	0.33	**26.26**	0.00	P,A	8.90	S100B	21q22.3
205226_at	0.95	0.84	A	2.60	**26.17**	0.00	P	66.60	PDGFRL	8p22-p21.3
227827_at	0.86	0.73	A	11.23	**25.84**	0.00	P	281.47		
216253_s_at	0.98	0.89	M,A	26.20	**25.70**	0.00	P	665.67	PARVB	2q13.2-q13.33
218625_at	1.00	0.96	A	45.20	**25.66**	0.00	P	1139.90	NRN1	6p25.1
205348_s_at	1.00	0.98	A	59.07	**25.66**	0.00	P	1490.67	DNCI1	7q21.3-q22.1
208885_at	0.87	0.76	A	63.87	**25.58**	0.00	P	1634.83	LCP1	13q14.3
210619_s_at	0.85	0.72	A	15.77	**25.54**	0.00	P	389.53	HYAL1	3p21.3-p21.2
220974_x_at	0.90	0.77	A	37.07	**25.38**	0.00	P	932.17	BA108L7.2	10q24.32
1556763_at	0.93	0.82	A	0.60	**25.36**	0.01	P,A	17.03		
238906_s_at	0.94	0.82	P,A	8.43	**25.33**	0.00	P	208.07	RHOJ	14q23.2
214632_at	1.00	0.97	P,A	19.43	**24.91**	0.00	P	476.77	NRP2	2q33.3
204860_s_at	0.99	0.94	A	2.77	**24.90**	0.00	P,A	68.73	BIRC1	5q13.1
231131_at	0.89	0.77	A	3.27	**24.82**	0.00	P	81.13	FLJ37659	xq21.33
217226_s_at	0.99	0.92	P,M	51.67	**24.74**	0.00	P	1264.97	PRRX1	1q24
226676_at	0.98	0.89	A	1.50	**24.46**	0.00	P,M,A	35.87	ZNF521	18q11.2
224191_x_at	0.87	0.76	A	23.37	**24.41**	0.00	P	559.13	ROPN1	3q21.2
238935_at	0.98	0.91	P,A	20.07	**24.33**	0.00	P	482.20	RPS27L	15q22.1
220298_s_at	0.98	0.90	A	2.83	**24.06**	0.00	P	66.63	SPATA6	1p33
1554443_s_at	0.86	0.74	A	15.90	**23.96**	0.00	P	370.47	VMD2	11q13
201137_s_at	0.95	0.84	A	6.50	**23.90**	0.00	P	153.80	HLA-DPB1	6p21.3
228601_at	0.99	0.94	A	11.23	**23.86**	0.00	P	265.27	LOC401022	2q31.2
208454_s_at	0.91	0.78	A	7.07	**23.58**	0.00	P	165.83	PGCP	8q22.2
202316_x_at	0.97	0.89	A	5.87	**23.57**	0.00	P	136.17	UBE4B	1p36.3
1560007_at	0.94	0.82	A	6.33	**23.56**	0.00	P	147.53		
219046_s_at	1.00	0.95	A	17.13	**23.53**	0.00	P	395.77	PKNOX2	11q24
218775_s_at	0.90	0.78	A	5.03	**23.44**	0.00	P	117.70	BOMB	4q35.1
229138_at	0.87	0.75	A	7.90	**23.26**	0.00	P	188.50	C12orf6	12p13.3
210807_s_at	0.86	0.73	A	7.43	**22.89**	0.00	P	168.53	SLC16A7	12q13
230413_s_at	1.00	0.98	P	32.63	**22.87**	0.00	P	735.47	AP1S2	Xp22.31
224477_s_at	0.99	0.92	A	4.23	**22.53**	0.00	P	95.03	SDOS	16p13.3
231361_at	0.97	0.87	A	12.97	**22.43**	0.00	P	284.03	NLGN1	3q26.32
212576_at	0.92	0.80	A	14.40	**22.39**	0.00	P	319.43	MGRN1	16p13.3
242137_at	0.97	0.88	A	1.57	**22.19**	0.00	P,M,A	34.40		
202897_at	0.92	0.80	A	20.07	**22.15**	0.00	P	433.37	PTPNS1	20p13
241386_at	0.94	0.83	A	7.53	**21.98**	0.00	P	163.00		
241612_at	0.84	0.71	A	5.83	**21.77**	0.00	P	125.77	FOXD3	1p32-p31
210869_s_at	1.00	0.96	P	44.07	**21.75**	0.00	P	943.73	MCAM	11q23.3
203068_at	0.88	0.77	A	46.17	**21.50**	0.00	P	972.53	KIAA0469	1p36.23
211546_x_at	0.99	0.94	P	31.20	**21.17**	0.00	P	651.30	SNCA	4q21
235359_at	0.86	0.73	A	19.53	**20.99**	0.00	P	397.90	UNQ3030	3q29
1553938_a_at	0.94	0.83	A	9.03	**20.80**	0.00	P	183.30	STK32A	5q32
213355_at	1.00	0.98	P,M,A	65.13	**20.75**	0.00	P	1332.20	SIAT10	3q12.2
209168_at	0.98	0.91	M,A	26.20	**20.65**	0.00	P	531.57	GPM6B	xp22.2
219102_at	0.93	0.81	A	8.70	**20.38**	0.00	P	176.83	RCN3	19q13.33
244463_at	0.90	0.78	A	2.17	**20.36**	0.00	P	44.00	ADAM23	2q33
205168_at	0.89	0.76	A	20.57	**20.05**	0.00	P	410.47	DDR2	1q12-q23
37965_at	0.96	0.87	P	78.67	**19.90**	0.00	P	1551.57	PARVB	2q13.2-q13.33
235147_at	0.85	0.72	A	7.00	**19.90**	0.00	P	138.03		
227617_at	0.94	0.83	A	53.60	**19.88**	0.00	P,M	1040.23		1p36.22
225681_at	1.00	0.97	P	34.80	**19.87**	0.00	P	681.17	CTHRC1	8q22.3
1553747_at	0.97	0.88	A	2.00	**19.37**	0.00	P	38.77	MGC16025	2q37.3
204780_s_at	0.92	0.81	P,A	17.70	**19.29**	0.00	P	334.93	TNFRSF6	10q24.1
210942_s_at	0.98	0.89	P,M	69.10	**19.26**	0.00	P	1316.80	SIAT10	3q12.2
226931_at	0.99	0.95	A	15.97	**19.25**	0.00	P	301.47	ARG99	12p11.23
203348_s_at	1.00	0.96	P,M	72.90	**19.19**	0.00	P	1378.33	ETV5	3q28
204629_at	1.00	0.95	A	67.03	**19.15**	0.00	P	1263.83	PARVB	2q13.2-q13.33
219855_at	1.00	0.95	A	8.37	**19.02**	0.00	P	156.93	NUDT11	xp11.23
1552658_a_at	0.88	0.77	A	6.17	**18.83**	0.00	P	114.07	NAV3	12q14.3
204214_s_at	0.94	0.82	A	60.30	**18.83**	0.00	P	1106.20	RAB32	6q24.2
227497_at	0.98	0.89	A	31.80	**18.82**	0.00	P	591.97		
1557218_s_at	0.98	0.91	A	4.10	**18.78**	0.00	P	75.93	FLJ34064	xp22.31
207827_x_at	1.00	0.96	P	42.53	**18.69**	0.00	P	785.23	SNCA	4q21
213808_at	0.98	0.91	A	2.27	**18.48**	0.00	P,M,A	41.20		
212667_at	0.97	0.88	A	12.80	**18.43**	0.00	P	232.40	SPARC	5q31.3-q32
207469_s_at	1.00	0.98	P	163.37	**18.43**	0.00	P	2959.47	PIR	xp22.31
227544_at	0.84	0.70	A	13.97	**18.37**	0.00	P	252.33	C14orf83	14q23.3
213010_at	0.99	0.93	A	29.53	**18.36**	0.00	P	534.97	PRKCDBP	11p15.4
203845_at	0.98	0.89	P	63.13	**18.28**	0.00	P	1138.60	PCAF	3p24
227530_at	0.94	0.83	A	24.83	**18.25**	0.00	P	448.23	AKAP12	6q24-q25
213325_at	0.87	0.74	A	9.87	**18.21**	0.00	P	178.53	PVRL3	3q13
204425_at	0.99	0.95	A	4.93	**18.19**	0.00	P	88.73	ARHGAP4	xq28
211162_x_at	0.96	0.86	A	20.90	**18.09**	0.00	P,M	373.80	SCD	10q23-q24
205407_at	1.00	0.96	P,M,A	15.67	**18.06**	0.00	P	278.13	RECK	9p13-p12
242770_at	0.97	0.87	A	5.47	**18.02**	0.00	P	96.20		
225544_at	0.99	0.94	A	3.13	**18.02**	0.00	P,A	55.67	TBX3	12q24.1
1555471_a_at	0.98	0.89	P,A	9.27	**17.90**	0.00	P	162.17	FMN2	1q43
232268_at	0.97	0.87	A	3.03	**17.82**	0.00	P	54.30		
215209_at	0.97	0.86	A	1.17	**17.81**	0.01	P,A	22.60	SEC24D	4q27
215591_at	0.89	0.78	A	4.67	**17.79**	0.00	P	82.43	SATB2	2q33
223079_s_at	0.91	0.80	P	55.97	**17.77**	0.00	P	989.60	GLS	2q32-q34
225728_at	0.95	0.84	A	21.70	**17.76**	0.00	P	382.40		
232122_s_at	0.99	0.92	A	28.40	**17.70**	0.00	P	493.80	FLJ12604	3q24-q25
205475_at	1.00	0.95	P,A	34.63	**17.66**	0.00	P	601.93	SCRG1	4q31-q32
209583_s_at	0.98	0.91	A	8.23	**17.52**	0.00	P	142.83	MOX2	3q12-q13
227952_at	0.97	0.88	A	14.33	**17.38**	0.00	P	244.07	ZNF595	4p16.3
215143_at	0.99	0.94	A	20.87	**17.38**	0.00	P	357.70	FLJ36166	7q22.1
203299_s_at	0.94	0.83	A	33.77	**17.36**	0.00	P	578.43	AP1S2	xp22.31
207671_s_at	0.90	0.77	A	21.10	**17.36**	0.00	P	356.50	VMD2	11q13
215719_x_at	0.92	0.80	P,A	11.33	**17.27**	0.00	P	195.33	TNFRSF6	10q24.1
207057_at	0.99	0.93	A	25.10	**17.26**	0.00	P	426.60	SLC16A7	12q13
225800_at	0.87	0.74	A	6.47	**17.18**	0.00	P,M,A	110.80	JAZF1	7p15.2-p15.1
1554954_at	0.96	0.86	A	5.37	**17.06**	0.00	P,M,A	91.03	C21orf90	21q22.3
210330_at	0.96	0.86	A	14.70	**17.02**	0.00	P	247.57	SGCD	5q33-q34
1565703_at	0.97	0.88	A	1.83	**16.92**	0.01	P,A	31.97	MADH4	18q21.1
1560240_at	0.92	0.80	A	2.87	**16.86**	0.00	P	48.40		
231688_at	0.85	0.73	A	2.00	**16.74**	0.01	P	35.10		
201654_s_at	0.91	0.79	A	2.00	**16.71**	0.01	P,A	34.27	HSPG2	1p36.1-p35
204638_at	1.00	0.96	P,A	76.77	**16.69**	0.00	P	1263.67	ACP5	9p13.3-p13.2
222846_at	0.92	0.81	P	24.27	**16.57**	0.00	P	400.30	RAB8B	15q22.1
228683_s_at	1.00	0.98	A	4.17	**16.22**	0.00	P	66.67	KCTD15	19q13.12
1569952_x_at	0.97	0.89	P,A	26.07	**16.18**	0.00	P	416.63		
231535_x_at	1.00	0.95	A	37.40	**16.03**	0.00	P	591.57	ROPN1	3q21.2
235061_at	0.88	0.75	A	10.20	**16.02**	0.00	P	158.47	KFZp761G0	4q22.1
1554302_s_at	0.97	0.87	A	23.23	**15.98**	0.00	P	368.43		
219064_at	0.98	0.90	A	21.80	**15.92**	0.00	P	343.87	ITIH5	10p15.1
214749_s_at	0.99	0.95	M,A	99.73	**15.88**	0.00	P	1557.67	FLJ20811	q21.33-q22.3
211431_s_at	0.94	0.83	A	8.90	**15.80**	0.00	P	139.03	TYRO3	5q15.1-q21.1
206644_at	0.95	0.85	P,M,A	8.77	**15.63**	0.00	P	138.63	NR0B1	p21.3-p21.2
1554508_at	0.92	0.81	A	8.07	**15.62**	0.00	P	126.67	PIK3AP1	10q24.2
205227_at	0.98	0.90	P,A	92.73	**15.46**	0.00	P	1407.73	IL1RAP	3q28
223302_s_at	0.99	0.94	A	31.63	**15.38**	0.00	P	479.10	VIK	7q22.1
201739_at	1.00	0.98	P	96.07	**15.32**	0.00	P	1445.83	SGK	6q23
210147_at	0.93	0.81	A	18.50	**15.29**	0.00	P	276.10	ART3	4p15.1-p14
223786_at	0.93	0.80	A	11.90	**15.27**	0.00	P,A	177.07	CHST6	16q22

230251_at	0.94	0.83	M,A	7.60	**15.26**	0.00	P	113.87	C6orf176	6q27
216254_at	0.89	0.76	A	2.07	**15.22**	0.00	P	31.30		
203650_at	1.00	0.95	P	25.20	**15.01**	0.00	P	373.47	PROCR	20q11.2
240772_at	0.90	0.79	A	2.07	**14.97**	0.01	P,A	31.37		
238067_at	0.87	0.73	A	5.20	**14.94**	0.00	P	77.60	FLJ20298	xq22.3
210762_s_at	0.98	0.91	A	29.20	**14.80**	0.00	P	424.23	DLC1	8p22
215960_at	1.00	0.96	A	25.27	**14.79**	0.00	P	368.73	SLC5A4	2q12.2-q12.3
219742_at	0.98	0.91	P,A	37.63	**14.73**	0.00	P	546.90	MGC10772	5q35.3
223170_at	0.95	0.84	A	62.60	**14.71**	0.00	P	899.40	KFZP564K19	17q12
231299_at	0.92	0.81	A	17.37	**14.60**	0.00	P	252.80	CENTG3	7q36.1
226066_at	0.99	0.95	P	251.90	**14.52**	0.00	P	3590.00	MITF	3p14.2-p14.1
223657_at	0.99	0.91	A	42.53	**14.50**	0.00	P	604.47	MGC10820	1p34.3
206806_at	0.96	0.85	A	14.20	**14.49**	0.00	P	200.67	DGKI	7q32.3-q33
223891_at	0.99	0.92	A	4.10	**14.47**	0.00	P,M,A	58.47	KCNQ5	6q14
201645_at	1.00	0.96	P	114.37	**14.29**	0.00	P	1608.83	TNC	9q33
220488_s_at	1.00	0.95	P,A	48.90	**14.28**	0.00	P	689.60	BCAS3	17q23
210471_s_at	0.98	0.90	A	20.80	**14.26**	0.00	P	293.50	KCNAB1	3q26.1
213249_at	0.98	0.90	P	41.70	**14.14**	0.00	P	577.10	FBXL7	5p15.1
226057_at	0.98	0.90	A	7.93	**14.11**	0.00	P	110.33	CDGAP	3q13.33
204527_at	1.00	0.98	P	104.40	**14.09**	0.00	P	1448.50	MYO5A	15q21
223618_at	0.96	0.85	A	20.40	**14.09**	0.00	P	280.67	FMN2	1q43
1554701_a_at	1.00	0.98	P	152.73	**14.04**	0.00	P	2114.43	TBC1D16	17q25.3
212664_at	1.00	0.97	A	72.90	**14.01**	0.00	P	1006.23	TUBB5	19p13.3
220484_at	0.91	0.80	A	51.73	**13.97**	0.00	P	706.80	MCOLN3	1p22.3
208978_at	0.98	0.90	A	50.50	**13.93**	0.00	P	698.13	CRIP2	14q32.3
220234_at	0.99	0.95	A	29.77	**13.91**	0.00	P	407.83	CA8	8q11-q12
227561_at	1.00	0.98	P,A	43.30	**13.88**	0.00	P	592.07	DDR2	1q12-q23
225442_at	0.97	0.87	A	33.77	**13.80**	0.00	P	460.40	DDR2	1q12-q23
235333_at	1.00	0.95	P	45.00	**13.70**	0.00	P	606.90	B4GALT6	18q11
204774_at	1.00	0.98	P	16.53	**13.65**	0.00	P	221.90	EVI2A	17q11.2
215617_at	0.96	0.85	P,A	9.43	**13.64**	0.00	P	126.57		
214449_s_at	0.92	0.81	P	48.17	**13.64**	0.00	P	652.70	RHOQ	2p21
205321_at	0.97	0.87	A	70.30	**13.63**	0.00	P	951.67	EIF2S3	xp22.2-p22.1
210854_x_at	1.00	0.99	P,M,A	119.43	**13.62**	0.00	P	1600.70	SLC6A8	xq28
216033_s_at	0.97	0.88	A	34.57	**13.62**	0.00	P	464.60	FYN	6q21
1555579_s_at	0.99	0.92	M,A	20.73	**13.55**	0.00	P	276.90	PTPRM	18p11.2
214297_at	1.00	0.95	A	17.73	**13.45**	0.00	P	236.97	CSPG4	15q23
223614_at	1.00	0.98	P,A	39.23	**13.39**	0.00	P	517.53	KFZp761D1	8q21.3
210135_s_at	1.00	0.95	A	17.97	**13.34**	0.00	P	236.10	SHOX2	3q25-q26.1
229942_at	0.96	0.87	A	22.23	**13.32**	0.00	P	295.80		
210139_s_at	0.99	0.92	P	117.33	**13.31**	0.00	P	1538.77	PMP22	17p12-p11.2
44783_s_at	1.00	0.96	P	66.23	**13.30**	0.00	P	865.30	HEY1	8q21
221667_s_at	0.88	0.76	A	8.50	**13.30**	0.00	P	111.70	HSPB8	12q24.23
244623_at	0.98	0.91	P	47.53	**13.23**	0.00	P	621.60		
227761_at	0.98	0.89	P	179.03	**13.06**	0.00	P	2311.47	MYO5A	15q21
1554758_a_at	0.99	0.94	A	4.00	**13.05**	0.00	P,M	51.87	CD99L2	xq28
205968_at	0.97	0.87	A	20.53	**12.99**	0.00	P	261.60	KCNS3	2p24
214040_s_at	0.93	0.82	A	23.43	**12.76**	0.00	P	295.30	GSN	9q33
202185_at	0.99	0.95	P	342.40	**12.68**	0.00	P	4257.70	PLOD3	7q22
1554522_at	0.98	0.90	P,A	7.27	**12.62**	0.00	P	90.60	CNNM2	10q24.33
220167_s_at	1.00	0.99	P	79.23	**12.46**	0.00	P	970.83	TP53TG3	16p13
200632_s_at	0.99	0.92	P	72.57	**12.44**	0.00	P	893.30	NDRG1	8q24.3
200756_x_at	0.99	0.93	M,A	142.30	**12.41**	0.00	P	1741.63	CALU	7q32
239331_at	1.00	0.98	A	52.30	**12.33**	0.00	P	635.60		
208893_s_at	0.95	0.85	A	27.43	**12.31**	0.00	P	340.27	DUSP6	12q22-q23
205439_at	0.90	0.77	A	24.13	**12.23**	0.00	P	292.33	GSTT2	22q11.23
220451_s_at	0.97	0.88	A	33.40	**12.20**	0.00	P	401.63	BIRC7	20q13.3
211925_s_at	0.98	0.90	A	4.80	**12.19**	0.00	P	57.80	PLCB1	20p12
239585_at	0.90	0.79	P,A	9.70	**12.16**	0.00	P	117.73	PCAF	3p24
228345_at	0.90	0.77	A	14.50	**12.10**	0.00	P	170.70		
204562_at	1.00	0.99	A	52.50	**12.07**	0.00	P	623.33	IRF4	6p25-p23
208167_s_at	0.97	0.87	A	9.77	**12.02**	0.00	P	116.70	MMP16	8q21
238622_at	0.97	0.88	P,A	18.57	**11.87**	0.00	P	218.43	RAP2B	3q25.2
230362_at	1.00	0.96	A	34.70	**11.87**	0.00	P	410.30	INPP5F	10q26.13
232173_at	0.97	0.88	A	29.57	**11.83**	0.00	P	343.33	LOC154790	7q34
209365_s_at	1.00	0.97	P,M	145.27	**11.79**	0.00	P	1691.90	ECM1	1q21
201718_s_at	0.95	0.85	A	14.87	**11.78**	0.00	P	174.57	EPB41L2	6q23
1557217_a_at	0.99	0.94	M,A	7.60	**11.73**	0.00	P	88.27	FLJ34064	xp22.31
203320_at	1.00	0.96	P	92.70	**11.63**	0.00	P	1060.77	LNK	12q24
209875_s_at	0.93	0.82	P,A	14.50	**11.58**	0.00	P	165.00	SPP1	4q21-q25
204781_s_at	0.90	0.78	A	16.33	**11.55**	0.00	P	186.43	TNFRSF6	10q24.1
202990_at	0.97	0.89	P	56.97	**11.51**	0.00	P	648.70	PYGL	14q21-q22
227657_at	0.98	0.89	A	5.33	**11.49**	0.00	P	60.17	RNF150	4q31.1
204469_at	0.91	0.80	P,A	9.63	**11.49**	0.00	P	108.20	PTPRZ1	7q31.3
211340_s_at	0.99	0.93	P	89.03	**11.31**	0.00	P	999.27	MCAM	11q23.3
225600_at	1.00	0.95	A	41.07	**11.30**	0.00	P	459.63	LOC286144	8q22.1
206653_at	0.99	0.93	P	73.30	**11.26**	0.00	P	816.90	POLR3G	5q14.3
214608_s_at	0.98	0.91	A	16.10	**11.24**	0.00	P	180.00	EYA1	8q13.3
238418_at	0.99	0.93	A	33.30	**11.24**	0.00	P	371.67	SLC35B4	7q33
209515_s_at	0.99	0.94	P	210.80	**11.23**	0.00	P	2335.17	RAB27A	15q15-q21.1
230249_at	0.90	0.77	A	3.87	**11.23**	0.00	P	42.57	KHDRBS3	8q24.2
209514_s_at	1.00	0.98	P	252.33	**11.21**	0.00	P	2781.03	RAB27A	15q15-q21.1
236591_at	1.00	0.97	A	8.37	**11.20**	0.00	P	93.70		
211737_x_at	0.94	0.83	A	24.33	**11.19**	0.00	P	265.93	PTN	7q33-q34
203167_at	0.97	0.87	A	102.70	**11.18**	0.00	P	1137.80	TIMP2	17q25
207233_s_at	1.00	0.96	P	215.00	**11.18**	0.00	P	2369.33	MITF	3p14.2-p14.1
226641_at	0.99	0.93	P	40.83	**11.17**	0.00	P	448.30	LOC91526	2q33.1
226225_at	0.99	0.95	P	52.40	**11.12**	0.00	P	575.17	MCC	5q21-q22
206529_x_at	0.95	0.84	P,M	15.87	**11.06**	0.00	P	173.77	SLC26A4	7q31
243348_at	0.95	0.84	A	2.50	**11.04**	0.00	P,A	27.63		
205541_s_at	0.90	0.78	A	8.53	**11.04**	0.00	P	92.33	GSPT2	p11.23-p11.21
236636_at	0.90	0.77	A	9.17	**10.99**	0.00	P	100.20		
219118_at	0.99	0.93	P	58.87	**10.98**	0.00	P	638.93	FKBP11	12q13.12
37966_at	0.99	0.91	A	72.57	**10.96**	0.00	P	778.80	PARVB	2q13.2-q13.33
214845_s_at	0.97	0.88	P	179.73	**10.94**	0.00	P	1943.43	CALU	7q32
1566989_at	0.92	0.79	A	20.50	**10.90**	0.00	P,A	228.47	ARID1B	6q25.1
220615_s_at	0.99	0.92	P,A	37.07	**10.89**	0.00	P	395.63	MLSTD1	12p11.23
201010_s_at	0.90	0.77	A	20.87	**10.89**	0.00	P	225.43	TXNIP	1q21.2
210233_at	0.98	0.89	P,A	41.23	**10.88**	0.00	P	444.60	IL1RAP	3q28
219563_at	1.00	0.98	P	77.53	**10.87**	0.00	P	831.10	C14orf139	14q32.2
230363_s_at	1.00	0.96	P	60.37	**10.84**	0.00	P	644.77	INPP5F	10q26.13
1555938_x_at	0.95	0.84	M,A	7.93	**10.81**	0.00	P	83.87	VIM	10p13
224178_s_at	0.98	0.90	A	4.17	**10.80**	0.01	P	45.93	SOX6	11p15.3
236154_at	0.97	0.88	A	4.37	**10.73**	0.00	P	45.97	QKI	6q26-27
240770_at	1.00	0.97	P	28.63	**10.72**	0.00	P	301.50	LOC134285	5q13.3
224822_at	0.99	0.92	A	22.83	**10.68**	0.00	P	240.80	DLC1	8p22
210951_x_at	0.97	0.88	P	293.23	**10.68**	0.00	P	3059.97	RAB27A	15q15-q21.1
220532_s_at	0.99	0.93	A	7.00	**10.59**	0.00	P,M	72.97	LR8	7q36.1
226338_at	1.00	0.96	A	38.00	**10.58**	0.00	P	395.47	KFZp762O0	8q21.3
63305_at	0.99	0.92	P	33.00	**10.58**	0.00	P	345.07	PKNOX2	11q24
202342_s_at	1.00	0.97	P	179.10	**10.55**	0.00	P	1863.10	TRIM2	4q31.3
225798_at	0.98	0.91	P,A	17.73	**10.55**	0.00	P	184.00	JAZF1	7p15.2-p15.1
235766_x_at	1.00	0.95	P	122.73	**10.43**	0.00	P	1265.40	RAB27A	15q15-q21.1
203349_s_at	1.00	0.99	P	132.87	**10.39**	0.00	P	1359.30	ETV5	3q28
37005_at	1.00	0.96	P	245.60	**10.38**	0.00	P	2511.33	NBL1	p36.13-p36.11
239296_at	0.96	0.86	A	14.33	**10.36**	0.01	P	148.37		
228266_s_at	1.00	0.96	A	12.03	**10.24**	0.00	P	121.33	HDGFRP3	15q11.2
1562730_a_at	0.98	0.90	A	15.37	**10.23**	0.00	P	154.77	SGCA	17q21
44790_s_at	0.97	0.88	A	10.73	**10.19**	0.00	P	107.70	C13orf18	13q14.11
220272_at	0.93	0.82	A	28.83	**10.15**	0.00	P	291.10	BNC2	9p22.2
1555355_a_at	0.94	0.83	P	34.63	**10.15**	0.00	P	343.00	ETS1	11q23.3
215695_s_at	1.00	0.96	P	82.33	**10.12**	0.00	P	818.37	GYG2	xp22.3
235158_at	0.99	0.94	P	144.73	**10.09**	0.00	P	1440.30	FLJ14803	7q32.3
242455_at	1.00	0.99	A	31.63	**10.07**	0.00	P	313.43	POU3F2	6q16
225647_s_at	0.98	0.91	P	190.50	**10.06**	0.00	P	1893.70	CTSC	1q14.1-q14.3
219450_at	0.99	0.93	P,A	24.27	**10.02**	0.00	P	240.23	FLJ11017	4p14
214913_at	0.99	0.94	P	25.70	**10.02**	0.00	P	253.60	ADAMTS3	4q21.1
208963_x_at	0.99	0.94	P	52.30	**10.01**	0.00	P	517.47	FADS1	1q12.2-q13.1
202501_at	0.99	0.92	P,A	50.17	**9.94**	0.00	P	491.13	MAPRE2	18q12.2
229812_at	0.98	0.89	P,A	35.63	**9.93**	0.00	P	349.43	USP48	1p36.12
243428_at	0.91	0.79	A	4.17	**9.92**	0.01	P,A	42.27	KCNQ1OT1	11p15
215185_at	0.91	0.79	A	8.27	**9.90**	0.00	P,A	81.57		9q34.2
222652_s_at	0.93	0.83	A	18.43	**9.89**	0.00	P	182.33	N-PAC	16p13.3
239252_at	0.96	0.85	P	23.43	**9.87**	0.00	P	225.87		

231789_at	0.94	0.83	A	6.97	**9.86**	0.00	P	67.17	PCDHB15	5q31
205383_s_at	1.00	0.98	A	28.33	**9.85**	0.00	P	275.03	ZNF288	3q13.2
240492_at	1.00	0.97	A	13.77	**9.84**	0.00	P	133.83		
231130_at	0.94	0.83	A	13.60	**9.82**	0.00	P	132.73	FKBP7	2q31.3
201655_s_at	0.98	0.90	M,A	15.73	**9.79**	0.00	P	152.53	HSPG2	1p36.1-p35
1555503_a_at	0.92	0.79	A	4.67	**9.78**	0.00	P	45.27	HUCEP11	1q32.1
228442_at	0.97	0.89	A	49.37	**9.78**	0.00	P	477.70	NFATC2	0q13.2-q13.3
203821_at	0.96	0.87	A	50.37	**9.74**	0.00	P	483.43	DTR	5q23
235777_at	0.90	0.79	A	14.70	**9.73**	0.00	P	140.63	LOC91526	2q33.1
241456_at	0.96	0.87	A	6.47	**9.72**	0.00	P,M	62.57		
238452_at	1.00	0.96	A	8.97	**9.69**	0.00	P	85.67	FLJ31052	1q23.1
229347_at	0.92	0.80	A	23.83	**9.67**	0.00	P	225.10		
203607_at	1.00	0.96	P	159.93	**9.63**	0.00	P	1517.87	INPP5F	10q26.13
212730_at	1.00	0.97	P	168.50	**9.62**	0.00	P	1592.23	DMN	15q26.3
225571_at	1.00	0.97	P	17.47	**9.61**	0.01	P	167.90	LIFR	5p13-p12
231490_at	0.97	0.89	A	5.93	**9.54**	0.00	P	56.20		
205673_s_at	0.94	0.84	P	31.93	**9.50**	0.00	P	301.10	ASB9	xp21.3
225622_at	1.00	0.96	P,A	28.47	**9.48**	0.00	P	265.00	PAG	8q21.12
221643_s_at	0.90	0.77	P,A	29.90	**9.48**	0.00	P	282.17	RERE	1p36.1-p36.2
212003_at	0.96	0.85	A	35.07	**9.44**	0.00	P	336.43	KFZp566C04	1p36.13
235104_at	0.94	0.84	A	15.30	**9.43**	0.00	P	143.90	LRAP	16
1558748_at	1.00	0.96	A	17.50	**9.41**	0.00	P	162.40		
240395_at	0.98	0.90	P,A	9.37	**9.38**	0.00	P	87.13		
219367_s_at	1.00	0.95	A	49.03	**9.37**	0.00	P	453.33	NRP2	2q33.3
232958_at	0.99	0.93	A	6.50	**9.36**	0.00	P	60.90	PYGL	14q21-q22
232682_at	0.95	0.84	P	88.53	**9.35**	0.00	P	821.20	FLJ10116	2q35
226587_at	0.99	0.92	A	10.77	**9.35**	0.00	P	98.77		
232773_at	0.99	0.93	P,A	21.73	**9.35**	0.00	P	201.27		
235371_at	0.99	0.93	P	73.57	**9.34**	0.00	P	677.47		3p14.2
213436_at	0.98	0.90	P,A	8.37	**9.33**	0.00	P	77.23	CNR1	6q14-q15
1557290_at	1.00	0.97	A	6.57	**9.33**	0.00	P,M,A	60.30		
209235_at	0.92	0.80	A	60.97	**9.31**	0.00	P	570.20	CLCN7	16p13
217989_at	1.00	0.96	P	95.53	**9.25**	0.00	P	868.67	DHRS8	4q22.1
218678_at	0.99	0.92	A	73.67	**9.24**	0.00	P	667.80	NES	1q23.1
1555434_a_at	0.86	0.73	A	29.70	**9.21**	0.01	P	274.23	SLC39A14	8p21.2
219257_s_at	1.00	0.95	P	146.67	**9.21**	0.00	P	1324.13	SPHK1	17q25.2
203928_x_at	1.00	0.96	A	18.57	**9.17**	0.00	P,A	167.27	MAPT	17q21.1
222851_at	0.98	0.90	P,A	50.70	**9.15**	0.00	P	459.20	FLJ10997	3p12.1
239135_at	0.93	0.80	A	14.00	**9.07**	0.00	P	126.00	FLJ11151	16p13.13
231972_at	0.99	0.91	A	40.80	**9.07**	0.00	P	364.30		
228714_at	0.92	0.81	A	15.57	**9.06**	0.00	P	140.77		
205110_s_at	0.98	0.91	A	24.70	**9.02**	0.00	P	218.40	FGF13	xq26.3
213371_at	0.99	0.94	A	26.57	**9.02**	0.00	P	237.10	LDB3	0q22.3-q23.2
1569969_a_at	0.92	0.79	A	1.93	**9.01**	0.00	P,A	17.27	UNC13C	15q21.1
203543_s_at	0.99	0.93	A	13.00	**9.00**	0.00	P	115.40	BTEB1	9q13
41113_at	0.95	0.84	A	7.97	**8.99**	0.00	P	71.00	ZNF500	16p13.3
225646_at	1.00	0.97	P	137.13	**8.99**	0.00	P	1213.67	CTSC	1q14.1-q14.3
206453_s_at	0.99	0.92	A	27.77	**8.98**	0.00	P,A	245.37	NDRG2	14q11.2
208670_s_at	0.98	0.91	M,A	86.50	**8.97**	0.00	P	768.20	CRI1	5q21.1-q21.2
229146_at	0.94	0.84	A	9.43	**8.95**	0.00	P	83.80	C7orf31	7p15.2
1556764_s_at	0.98	0.91	A	8.93	**8.93**	0.00	P,A	78.33		
216286_at	0.97	0.89	A	10.30	**8.92**	0.00	P	92.07		
201522_x_at	1.00	0.95	P	70.87	**8.91**	0.00	P	622.73	SNRPN	15q12
235675_at	0.97	0.88	P	10.50	**8.91**	0.01	P	95.37	LOC200895	3q11.2
231015_at	0.95	0.84	A	41.37	**8.91**	0.00	P	362.90	KLF15	3q13-q21
219412_at	0.99	0.93	P	238.13	**8.89**	0.00	P	2074.23	RAB38	11q14
209465_x_at	0.94	0.83	M,A	13.67	**8.83**	0.00	P	118.17	PTN	7q33-q34
206704_at	0.99	0.93	P	58.90	**8.82**	0.00	P	512.73	CLCN5	p11.23-p11.22
221601_s_at	1.00	0.96	A	55.80	**8.80**	0.00	P	482.40	TOSO	1q32.1
219117_s_at	0.99	0.93	P	161.17	**8.80**	0.00	P	1394.50	FKBP11	12q13.12
213543_at	0.98	0.89	A	12.80	**8.72**	0.00	P	109.73	SGCD	5q33-q34
230024_at	0.87	0.74	A	26.37	**8.72**	0.01	P	233.03	AARSL	6p21.1
244360_at	0.96	0.86	A	12.30	**8.70**	0.00	P	104.93	FBXO13	5q21.3
238478_at	0.98	0.89	A	30.10	**8.70**	0.00	P	258.97	BNC2	9p22.2
204944_at	0.99	0.92	M,A	34.97	**8.70**	0.00	P	300.37	PTPRG	3p21-p14
214492_at	0.99	0.92	A	29.83	**8.68**	0.00	P	255.60	SGCD	5q33-q34
228146_at	0.95	0.85	A	9.13	**8.67**	0.00	P,A	77.57	LOC339263	17p11.2
227176_at	0.97	0.88	A	29.27	**8.62**	0.00	P	249.73		
230538_at	0.96	0.87	P,A	16.93	**8.60**	0.00	P	142.87	RaLP	5q21.1-q21.2
202974_at	0.99	0.95	A	62.63	**8.58**	0.00	P	530.73	MPP1	xq28
205609_at	0.96	0.85	A	16.83	**8.57**	0.00	P	143.03	ANGPT1	8q22.3-q23
239353_at	0.95	0.85	A	12.73	**8.55**	0.00	P	106.33		
213096_at	0.99	0.93	A	38.40	**8.54**	0.00	P	324.53	HUCEP11	1q32.1
216493_s_at	0.88	0.74	A	6.70	**8.53**	0.00	P,A	55.97	IMP-3	7p11
224374_s_at	1.00	0.97	A	62.00	**8.53**	0.00	P	519.80	EMILIN2	18p11.3
1555403_a_at	0.92	0.80	A	2.40	**8.52**	0.03	P,A	22.70	CDH19	18q22-q23
1554874_at	0.99	0.94	A	8.57	**8.51**	0.00	P	72.23	MITF	3p14.2-p14.1
206401_s_at	0.99	0.92	A	14.87	**8.50**	0.00	P,A	124.33	MAPT	17q21.1
237803_x_at	0.99	0.95	A	3.37	**8.50**	0.00	P	28.20		
205880_at	0.95	0.84	A	16.83	**8.49**	0.00	P	141.67	PRKCM	14q11
212122_at	0.99	0.94	P	31.93	**8.48**	0.00	P	268.27	RHOQ	2p21
219743_at	0.94	0.82	A	9.73	**8.48**	0.00	P	81.73	HEY2	q22.2-q22.33
232530_at	0.98	0.89	A	27.23	**8.45**	0.00	P	227.87	PLD1	3q26
1552641_s_at	1.00	0.96	A	63.93	**8.44**	0.00	P	536.63	ATAD3B	1p36.33
244829_at	0.99	0.94	P,A	62.47	**8.44**	0.00	P	520.83	MGC40222	6p24.1
206826_at	1.00	0.95	P	16.90	**8.43**	0.00	P	140.93	PMP2	3q21.3-q22.1
222686_s_at	1.00	0.99	A	84.43	**8.39**	0.00	P	705.70	FLJ11151	16p13.13
207084_at	0.87	0.76	A	8.50	**8.25**	0.01	P	69.70	POU3F2	6q16
202920_at	0.96	0.85	P,A	13.53	**8.22**	0.00	P	110.10	ANK2	4q25-q27
235287_at	0.98	0.89	P,A	18.10	**8.21**	0.00	P	147.93	CDK6	7q21-q22
239034_at	0.98	0.91	P,M	24.37	**8.20**	0.00	P	197.60	LOC203414	xp11.3
214755_at	0.99	0.95	A	51.80	**8.18**	0.00	P	417.10	UAP1L1	9q34.3
237630_s_at	0.91	0.79	A	7.07	**8.16**	0.00	P,A	57.67		
217553_at	1.00	0.97	A	36.30	**8.14**	0.00	P	290.10	MGC87042	7p15.3
235931_at	0.98	0.89	P	52.80	**8.13**	0.00	P	423.60		
215977_x_at	0.92	0.82	A	19.20	**8.09**	0.00	P	154.43	GK	xp21.3
236610_at	0.94	0.83	A	6.43	**8.06**	0.01	P,A	53.00		
226269_at	0.99	0.92	P	23.53	**8.06**	0.00	P	187.67	GDAP1	8q13.3
227683_x_at	0.87	0.73	A	2.40	**8.04**	0.02	P,A	20.40	NUDT4	
236948_x_at	0.94	0.83	P,A	4.00	**8.04**	0.00	P	32.67	SFRS11	1p31
222294_s_at	1.00	0.98	P	122.43	**8.04**	0.00	P	971.30	RAB27A	15q15-q21.1
224560_at	0.93	0.81	P	303.17	**7.98**	0.00	P	2399.10	TIMP2	17q25
216874_at	1.00	0.96	A	15.13	**7.98**	0.00	P	119.23		
230645_at	0.94	0.83	A	13.87	**7.97**	0.00	P	109.20	FRMD3	9q21.33
212631_at	0.99	0.95	P	160.87	**7.96**	0.00	P	1262.77		
1569399_at	1.00	0.95	A	31.83	**7.95**	0.00	P	250.70		
216192_at	0.97	0.87	A	4.47	**7.95**	0.00	P,A	35.27	FABP7	6q22-q23
212545_s_at	0.94	0.82	A	3.67	**7.94**	0.02	P,A	31.37	ZHX3	20q12
204105_s_at	0.94	0.83	A	9.23	**7.94**	0.00	P	71.73	NRCAM	7q31.1-q31.2
205193_at	1.00	0.95	P	214.87	**7.94**	0.00	P	1682.40	MAFF	22q13.1
226516_at	1.00	0.96	A	176.37	**7.85**	0.00	P	1363.00	C19orf28	19p13.3
242116_x_at	0.94	0.83	M,A	13.73	**7.85**	0.00	P	107.27		
215812_s_at	1.00	0.97	A	86.30	**7.85**	0.00	P	666.57	SLC6A8	xq28
202827_s_at	0.97	0.89	A	11.17	**7.82**	0.01	P,M,A	90.17	MMP14	14q11-q12
211804_s_at	0.92	0.79	P,A	47.60	**7.81**	0.00	P	371.70	CDK2	12q13
201810_s_at	0.99	0.93	P	141.93	**7.78**	0.00	P	1092.60	SH3BP5	3p24.3
209032_s_at	0.90	0.79	P,M,A	35.60	**7.78**	0.00	P	270.37	IGSF4	11q23.2
235199_at	0.99	0.95	A	21.43	**7.77**	0.00	P,M	164.13		
1561691_at	0.95	0.85	P	31.13	**7.75**	0.00	P	236.93	LOC285735	6q23.1
235202_x_at	0.99	0.92	A	17.20	**7.73**	0.00	P	131.80	IKIP	12q23.1
243041_s_at	0.93	0.81	A	9.70	**7.69**	0.00	P	74.43	RBMS3	3p24-p23
235205_at	0.99	0.93	P,A	66.67	**7.67**	0.00	P	503.30		8q23.1
204344_s_at	0.91	0.80	A	20.77	**7.66**	0.00	P	159.83	SEC23A	14q13.3
242424_at	0.96	0.85	A	6.17	**7.65**	0.01	P,A	47.03	HCCR1	12q13.13
201516_at	1.00	0.98	P	324.20	**7.63**	0.00	P	2433.37	SRM	1p36-p22
201337_s_at	0.94	0.83	P,A	111.43	**7.61**	0.00	P	844.60	VAMP3	1p36.23
225337_at	0.98	0.89	A	56.33	**7.57**	0.00	P	419.87	ABHD2	15q26.1
218694_at	1.00	0.99	P	62.40	**7.57**	0.00	P	464.90	ARMCX1	q21.33-q22.2
1552256_a_at	1.00	0.95	P	135.97	**7.55**	0.00	P	1011.87	SCARB1	12q24.31
207793_s_at	0.99	0.94	A	2.67	**7.53**	0.00	P	20.13	EPB41	1p33-p32
201819_at	1.00	0.97	P	81.20	**7.53**	0.00	P	601.30	SCARB1	12q24.31
219282_s_at	0.98	0.91	P,A	38.90	**7.53**	0.00	P	289.40	TRPV2	17p11.2
224600_at	0.97	0.88	P	232.13	**7.51**	0.00	P	1725.77	CGGBP1	3p12-p11.1
205174_s_at	0.99	0.93	P	242.03	**7.50**	0.00	P	1792.10	QPCT	2p22.3

1554464_a_at	0.97	0.88	P	149.13	**7.47**	0.00	P	1103.13	CRTAP	3p22
210138_at	1.00	0.98	P,A	27.90	**7.46**	0.00	P	204.83	RGS20	8q12.1
1555542_at	0.99	0.92	A	12.20	**7.42**	0.00	P	89.30	FLJ36748	5q33.1
204701_s_at	0.99	0.93	A	17.53	**7.41**	0.01	P,A	130.47	STOML1	15q24-q25
216252_x_at	0.96	0.85	P,A	22.67	**7.39**	0.00	P	166.00	TNFRSF6	10q24.1
213241_at	0.99	0.92	A	26.20	**7.38**	0.00	P	190.10	PLXNC1	12q23.3
202468_s_at	1.00	0.98	P	112.77	**7.37**	0.00	P	816.93	CTNNAL1	9q31.2
243366_s_at	0.90	0.79	A	25.97	**7.33**	0.00	P	188.67	RP26	2q32.1
220178_at	1.00	1.00	P,A	152.90	**7.29**	0.00	P	1096.37	C19orf28	19p13.3
45288_at	1.00	0.96	P	57.93	**7.29**	0.00	P	416.23	ABHD6	3p21.2
227850_x_at	0.90	0.77	A	3.83	**7.28**	0.00	P,M	28.30	CDC42EP5	19q13.42
226252_at	0.99	0.93	P,A	21.33	**7.26**	0.00	P	153.57		
1554020_at	0.98	0.90	A	79.87	**7.25**	0.00	P	572.87	BICD1	2p11.2-p11.1
210964_s_at	1.00	1.00	A	126.43	**7.24**	0.00	P	900.47	GYG2	xp22.3
228728_at	0.95	0.83	A	13.23	**7.23**	0.00	P,M	93.40	FLJ21986	7q31.32
225925_s_at	0.99	0.92	P	109.33	**7.21**	0.00	P	774.20	USP48	1p36.12
232023_at	0.99	0.94	P	19.60	**7.20**	0.00	P	139.43	MGC26979	8q22.1
219260_s_at	0.96	0.86	P	135.73	**7.19**	0.00	P	966.67	DERP6	17p13.2
226152_at	0.97	0.88	P	55.63	**7.18**	0.00	P	397.43	TTC7L1	14q32.12
206896_s_at	0.95	0.84	P,A	43.30	**7.15**	0.00	P	303.03	GNG7	19p13.3
219616_at	0.98	0.91	A	58.37	**7.14**	0.00	P	409.47	FLJ21963	12q21.31
230372_at	0.95	0.84	A	27.60	**7.14**	0.00	P	192.10	HAS2	8q24.12
226075_at	1.00	0.98	P	69.10	**7.14**	0.00	P	485.40	SSB1	1p36.22
227231_at	0.99	0.92	P,M,A	10.27	**7.12**	0.00	P	72.10	KIAA1211	4q12
212486_s_at	0.97	0.87	M,A	17.67	**7.12**	0.00	P	123.50	FYN	6q21
201811_x_at	1.00	0.98	P	238.13	**7.12**	0.00	P	1665.90	SH3BP5	3p24.3
225540_at	1.00	0.98	P	114.27	**7.12**	0.00	P	799.40	MAP2	2q34-q35
203501_at	0.93	0.82	A	20.37	**7.11**	0.00	P	143.57	PGCP	8q22.2
1562475_at	0.97	0.88	A	11.77	**7.11**	0.00	P	82.77		2q23.1
238751_at	0.98	0.90	A	21.57	**7.10**	0.00	P	152.17		
230741_at	1.00	0.96	A	26.53	**7.10**	0.00	P	184.90		
223510_at	0.99	0.93	A	33.70	**7.10**	0.00	P	237.37	NRP2	2q33.3
207323_s_at	0.96	0.86	M,A	18.67	**7.07**	0.00	P	131.07	MBP	18q23
1555139_a_at	0.92	0.81	A	5.80	**7.07**	0.00	P,A	40.60	ZA20D1	1q21.3
217620_s_at	0.99	0.95	P	35.43	**7.07**	0.00	P	247.20	PIK3CB	3q22.3
202896_s_at	0.99	0.92	A	61.10	**7.06**	0.00	P	423.70	PTPNS1	20p13
1554332_a_at	0.99	0.95	P	92.80	**7.06**	0.00	P	645.97		
225881_at	1.00	0.95	P,A	171.40	**7.06**	0.00	P	1192.23	SLC35B4	7q33
242465_at	0.95	0.85	A	18.77	**7.04**	0.00	P	130.80		
202375_at	0.97	0.88	P	50.77	**7.04**	0.00	P	352.70	SEC24D	4q27
238459_x_at	0.95	0.84	A	8.37	**7.03**	0.00	P	57.37	SPATA6	1p33
226273_at	0.99	0.94	P	69.60	**7.01**	0.01	P	486.33	LOC158563	xp11.23
204633_s_at	1.00	0.99	P,A	121.80	**7.01**	0.00	P	839.43	RPS6KA5	14q31-q32.1
227230_s_at	0.92	0.81	A	20.43	**7.01**	0.00	P	143.00	KIAA1211	4q12
225727_at	1.00	0.96	P	49.80	**6.99**	0.00	P	344.00	PLEKHH1	14q24.1
203329_at	0.99	0.93	P,A	42.57	**6.95**	0.00	P	292.77	PTPRM	18p11.2
1567224_at	0.99	0.93	P,A	21.57	**6.92**	0.00	P	147.30		
205355_at	1.00	0.95	P	29.00	**6.92**	0.00	P	198.27	ACADSB	10q25-q26
203705_s_at	0.98	0.91	P	75.97	**6.90**	0.00	P	518.93	FZD7	2q33
1559634_at	0.94	0.84	A	2.50	**6.87**	0.02	P,A	17.70	CHRM3	1q41-q44
209123_at	1.00	0.98	P	289.10	**6.86**	0.00	P	1953.73	QDPR	4p15.31
225045_at	0.96	0.86	A	45.50	**6.82**	0.00	P	308.90	KIAA1212	2p16.3
1554717_a_at	0.98	0.89	A	12.00	**6.82**	0.00	P	80.93	PDE4D	5q12
205566_at	0.99	0.94	P	82.30	**6.81**	0.00	P	549.80	ABHD2	15q26.1
239640_at	1.00	0.97	A	2.80	**6.77**	0.01	P,A	19.40		7
209087_x_at	1.00	0.97	P	89.97	**6.77**	0.00	P	599.50	MCAM	11q23.3
222755_s_at	0.99	0.92	A	11.57	**6.76**	0.00	P,A	77.67	KIAA1416	8q12.1
239081_at	0.95	0.84	P,A	27.90	**6.74**	0.00	P	185.40	BIRC2	11q22
235570_at	0.99	0.92	A	23.77	**6.73**	0.00	P	157.43		
225793_at	1.00	0.97	P	132.13	**6.72**	0.00	P	874.70	MGC46719	1q21.2
1556216_s_at	0.99	0.94	M,A	8.03	**6.72**	0.01	P	53.93		
1553244_at	0.96	0.85	A	17.23	**6.71**	0.01	P	114.33	FLJ34064	xp22.31
202898_at	0.99	0.93	A	63.03	**6.71**	0.00	P	420.03	SDC3	1pter-p22.3
1552275_s_at	0.98	0.89	A	23.73	**6.69**	0.00	P	157.27	PXK	3p21.2
207012_at	0.98	0.89	A	29.83	**6.68**	0.00	P	195.47	MMP16	8q21
215728_s_at	0.95	0.85	P,A	66.47	**6.67**	0.00	P	446.23	BACH	p36.31-p36.11
227965_at	0.98	0.91	A	17.63	**6.67**	0.01	P	116.67	APC2	19p13.3
229363_at	1.00	0.99	M,A	32.83	**6.64**	0.00	P	214.30		
233406_at	0.97	0.87	A	2.73	**6.62**	0.00	P,A	17.97	KIAA0256	15q15.3
223837_at	1.00	0.95	A	37.37	**6.62**	0.00	P	243.77	GULP1	2q32.3-q33
224494_x_at	0.99	0.94	A	21.30	**6.61**	0.00	P	139.47	DHRS10	19q13.33
1558404_at	0.99	0.92	P	29.63	**6.59**	0.00	P	191.63		
233276_at	0.98	0.91	A	23.97	**6.59**	0.00	P	156.27		
219863_at	1.00	0.96	P,M,A	42.93	**6.59**	0.00	P	279.63	CEB1	4q22.1-q23
203518_at	0.99	0.94	P	105.63	**6.59**	0.00	P	688.03	CHS1	1q42.1-q42.2
229568_at	1.00	0.97	A	44.13	**6.58**	0.00	P	285.93	MOBKL2B	9p21.1
214336_s_at	0.93	0.80	P	41.83	**6.58**	0.01	P	283.10	COPA	1q23-q25
205991_s_at	1.00	0.98	P,M,A	31.47	**6.57**	0.00	P	204.87	PRRX1	1q24
238009_at	0.94	0.83	A	60.07	**6.54**	0.00	P	386.73		
216048_s_at	0.96	0.85	P	96.57	**6.54**	0.00	P	625.83	RHOBTB3	5q15
210105_s_at	0.99	0.92	P	159.10	**6.54**	0.00	P	1023.13	FYN	6q21
228158_at	0.99	0.92	A	55.40	**6.53**	0.00	P	356.20		
207425_s_at	0.98	0.89	P,A	33.90	**6.53**	0.00	P	219.37	MSF	17q25
203711_s_at	0.99	0.94	P	248.27	**6.51**	0.00	P	1599.53	HIBCH	2q32.3
205234_at	0.99	0.92	P,A	19.53	**6.51**	0.00	P	126.80	SLC16A4	1p13.2
230395_at	0.99	0.93	A	16.40	**6.51**	0.00	P,A	105.13		
226084_at	0.93	0.81	P,A	14.30	**6.50**	0.00	P	90.53	MAP1B	5q13
215945_s_at	0.98	0.89	P,M,A	46.37	**6.49**	0.00	P	298.43	TRIM2	4q31.3
225974_at	0.97	0.89	P	101.73	**6.49**	0.00	P	653.70	KFZp762C11	8q21.3
219372_at	1.00	0.96	P	161.90	**6.49**	0.00	P	1035.47	CDV-1	12q24.13
242836_at	1.00	0.98	P	22.53	**6.49**	0.00	P	143.90		
212098_at	0.99	0.94	P	261.23	**6.44**	0.00	P	1651.37	LOC151162	2q21.3
219451_at	0.95	0.85	A	47.63	**6.44**	0.00	P	302.33	MSRB	10p12
240120_at	0.98	0.90	A	11.73	**6.41**	0.01	P	76.00		
227221_at	0.96	0.85	P,A	41.73	**6.39**	0.00	P	263.97		
217781_s_at	0.98	0.90	P	434.60	**6.38**	0.00	P	2739.20	ZFP106	15q14
219759_at	0.99	0.95	A	25.73	**6.37**	0.00	P	161.60	LRAP	16
207060_at	1.00	0.97	A	17.20	**6.37**	0.00	P,M,A	107.97	EN2	7q36
205097_at	0.97	0.88	P	194.67	**6.34**	0.00	P	1220.20	SLC26A2	5q31-q34
224668_at	0.95	0.83	A	23.07	**6.31**	0.00	P,A	142.30	C20orf35	20q13.12
237817_at	0.98	0.90	P	24.80	**6.30**	0.00	P	154.70	SSR3	3q25.31
232127_at	0.98	0.91	P	44.70	**6.27**	0.01	P	280.50	CLCN5	p11.23-p11.22
211603_s_at	0.98	0.91	P,A	83.90	**6.27**	0.00	P	516.37	ETV4	17q21
210948_s_at	0.99	0.94	A	21.07	**6.26**	0.00	P	129.70	LEF1	4q23-q25
1554272_at	0.93	0.83	A	2.83	**6.26**	0.00	P,A	17.83	LRAP	16
238621_at	0.95	0.85	A	32.93	**6.24**	0.00	P	202.43	FMN	15q13.2
229253_at	0.97	0.87	P	146.77	**6.23**	0.00	P	895.27	CTMP	1q21
226059_at	0.97	0.87	A	33.87	**6.23**	0.00	P	208.63	FLJ12770	1q23.1
221953_s_at	0.99	0.94	P,A	83.83	**6.22**	0.00	P	511.50	MMP24	20q11.2
206661_at	0.92	0.79	A	11.63	**6.22**	0.00	P	72.20	DRF1	17q21.31
240264_at	0.96	0.87	A	9.40	**6.21**	0.00	P	57.13		
226281_at	0.98	0.91	P	56.27	**6.21**	0.00	P	341.80	DNER	2q37.1
241972_at	1.00	0.96	A	21.67	**6.18**	0.00	P	131.73	LOC401588	xp11.3
226022_at	1.00	0.96	P	259.60	**6.18**	0.00	P	1581.53	SASH1	6q24.3
208875_s_at	0.98	0.91	P,A	105.90	**6.18**	0.00	P	646.63	PAK2	3q29
220079_s_at	0.99	0.93	P	190.13	**6.18**	0.00	P	1154.93	USP48	1p36.12
200755_s_at	0.98	0.90	P	468.70	**6.17**	0.00	P	2855.27	CALU	7q32
200744_s_at	0.98	0.91	P	264.07	**6.16**	0.00	P	1611.53	GNB1	1p36.33
225575_at	0.99	0.93	A	51.50	**6.15**	0.00	P	313.03	LIFR	5p13-p12
1564479_a_at	1.00	0.99	A	59.87	**6.15**	0.00	P	363.67		
208892_s_at	0.98	0.91	P	193.13	**6.14**	0.00	P	1172.43	DUSP6	12q22-q23
1555240_s_at	0.98	0.91	P,A	50.33	**6.13**	0.00	P	307.20	GNG12	1p31.2
212793_at	0.98	0.89	P,A	66.17	**6.10**	0.00	P	398.97	DAAM2	6p21.1
226592_at	1.00	0.96	A	22.30	**6.10**	0.00	P,A	133.77		
217354_s_at	0.97	0.88	P,M,A	57.13	**6.09**	0.00	P	342.57	HPS1	0q23.1-q23.3
230575_at	0.92	0.81	A	15.17	**6.04**	0.00	P	90.93		
241348_at	0.99	0.94	P	85.70	**6.04**	0.00	P	510.90	FLJ10997	3p12.1
230177_at	0.99	0.95	P,M	63.27	**6.04**	0.00	P	376.20		
38069_at	1.00	0.96	P	198.47	**6.02**	0.00	P	1174.53	CLCN7	16p13
208669_s_at	0.97	0.88	P	429.30	**6.02**	0.00	P	2557.43	CRI1	5q21.1-q21.2
221679_s_at	0.97	0.88	P,A	36.50	**6.01**	0.00	P	217.37	ABHD6	3p21.2
202895_s_at	0.98	0.90	A	33.17	**6.01**	0.00	P	195.60	PTPNS1	20p13
222738_at	0.97	0.88	P,A	16.73	**6.00**	0.00	P	98.47	BOMB	4q35.1
204635_at	1.00	0.99	P	124.37	**6.00**	0.00	P	734.07	RPS6KA5	14q31-q32.1

226633_at	0.99	0.92	P	171.90	**5.99**	0.00	P	1008.67	RAB8B	15q22.1
218514_at	1.00	0.99	P	88.63	**5.99**	0.00	P	522.27	FLJ10587	17q23.2
236798_at	0.99	0.92	P	65.90	**5.97**	0.00	P	385.97		
235046_at	0.99	0.93	P	105.80	**5.96**	0.00	P	623.87		
214830_at	0.99	0.95	P	69.70	**5.95**	0.00	P	409.33	SLC38A6	14q23.1
212120_at	1.00	0.98	P	254.90	**5.94**	0.00	P	1490.63	PIGF	2p21-p16
223703_at	0.98	0.89	P,A	36.00	**5.93**	0.00	P	208.47	C10orf11	10q22.3
226038_at	1.00	0.98	P	75.23	**5.91**	0.00	P	439.80	FLJ23749	8p23.1
1564911_at	0.94	0.83	P,A	40.80	**5.90**	0.00	P	241.30	MATR3	5q31.3
1560011_at	0.94	0.81	A	16.03	**5.90**	0.00	P	93.67	PSCA	8q24.2
220205_at	0.99	0.93	P,A	11.03	**5.89**	0.00	P	64.33	TPTE	21p11
222407_s_at	0.98	0.91	P	345.23	**5.87**	0.00	P	2005.10	ZFP106	15q14
228490_at	0.99	0.94	P	75.27	**5.84**	0.00	P	433.07	ABHD2	15q26.1
208962_s_at	0.99	0.94	P	60.97	**5.84**	0.00	P	351.93	FADS1	1q12.2-q13.1
1562657_a_at	1.00	0.96	P,M,A	59.37	**5.83**	0.00	P	341.03	C10orf90	10q26.2
224045_x_at	0.96	0.87	A	7.13	**5.83**	0.00	P,A	40.77	C18orf2	18p11
204920_at	0.97	0.89	A	24.47	**5.80**	0.00	P	140.20	CPS1	2q35
1773_at	0.97	0.88	A	55.30	**5.79**	0.00	P	318.67	FNTB	14q23-q24
225717_at	0.99	0.94	P	77.17	**5.79**	0.00	P	440.93	KIAA1715	2q31
213005_s_at	1.00	0.98	P	252.33	**5.79**	0.00	P	1437.73	ANKRD15	9p24.3
229487_at	0.95	0.84	A	19.17	**5.78**	0.00	P	110.13	EBF	5q34
235733_at	0.98	0.92	M	82.70	**5.77**	0.00	P	472.17		
218610_s_at	0.98	0.89	P	23.80	**5.75**	0.00	P	135.87	FLJ11151	16p13.13
224776_at	0.99	0.94	P	255.03	**5.75**	0.00	P	1440.57	:FZp586M1{	8p11.21
201860_s_at	1.00	0.99	P	72.17	**5.73**	0.00	P	406.63	PLAT	8p12
233092_s_at	0.96	0.86	A	10.43	**5.73**	0.00	P	59.43	KFZP434B0	13
223266_at	0.99	0.95	P,A	184.83	**5.72**	0.00	P	1044.83	ALS2CR2	2q33-q34
209936_at	0.97	0.89	A	12.30	**5.72**	0.00	P,A	68.90	RBM5	3p21.3
1556410_a_at	0.98	0.90	A	13.07	**5.72**	0.00	P	73.87	KRTAP19-1	21q22.1
209610_s_at	1.00	0.96	P	374.27	**5.72**	0.00	P	2110.57	SLC1A4	2p15-p13
212909_at	0.98	0.91	A	35.50	**5.69**	0.00	P	199.63	MGC29643	2q21.2
232004_at	1.00	0.99	P	125.97	**5.68**	0.00	P	704.07	HNRPR	1p36.11
212110_at	0.99	0.93	P	390.87	**5.67**	0.00	P	2173.97	SLC39A14	8p21.2
242842_at	0.97	0.87	A	23.87	**5.65**	0.00	P	132.67	PARVG	22q13.2-q13
229024_at	0.99	0.91	A	19.70	**5.64**	0.00	P	109.63		
230466_s_at	0.98	0.91	P	192.23	**5.64**	0.00	P	1072.67		
225532_at	1.00	0.97	P	180.73	**5.63**	0.00	P	999.03	CABLES1	18q11.2
224851_at	0.98	0.90	P	183.33	**5.61**	0.00	P	1019.87	CDK6	7q21-q22
221552_at	0.97	0.87	A	79.60	**5.59**	0.00	P	438.53	ABHD6	3p21.2
235696_at	0.95	0.85	P,A	10.50	**5.59**	0.00	P	57.53		
212423_at	1.00	0.96	P,M,A	70.23	**5.59**	0.00	P	386.73	C10orf56	10q23.1
205501_at	0.98	0.91	M,A	6.00	**5.58**	0.00	P	32.87		
215415_s_at	0.99	0.94	A	18.90	**5.58**	0.00	P	105.57	CHS1	1q42.1-q42.2
235051_at	0.96	0.86	P	57.10	**5.57**	0.00	P	314.87	C3orf6	3q28
222378_at	0.98	0.89	A	12.83	**5.55**	0.01	P,M	71.17		
232269_x_at	1.00	0.98	P	147.80	**5.55**	0.00	P	808.50	C16orf23	16p13.3
238530_at	0.97	0.88	P	82.00	**5.54**	0.00	P	450.57	NNT	5p13.1-5cen
207325_x_at	0.99	0.95	P	315.67	**5.54**	0.00	P	1723.50	MAGEA1	xq28
1556283_s_at	0.99	0.94	A	23.27	**5.54**	0.00	P	126.87	FGFR1OP2	12p12.1
236220_at	0.99	0.93	A	10.73	**5.53**	0.00	P	58.23		
228602_at	0.99	0.92	P,A	41.47	**5.53**	0.00	P	227.03	SGCD	5q33-q34
243808_at	0.98	0.90	A	16.70	**5.52**	0.01	P	92.37		
202912_at	1.00	0.97	P,M,A	132.67	**5.51**	0.00	P	718.13	ADM	11p15.4
223253_at	0.97	0.87	P,A	34.77	**5.49**	0.00	P	188.30	UCC1	7p14.1
216887_s_at	0.96	0.86	A	28.80	**5.49**	0.00	P	156.90	LDB3	0q22.3-q23.2
224497_x_at	0.96	0.86	P,A	19.87	**5.48**	0.00	P	108.33	DHRS10	19q13.33
235719_at	0.92	0.81	A	12.90	**5.48**	0.00	P	70.77	CYP4V2	4q35.1
242932_at	1.00	0.97	P	54.70	**5.48**	0.01	P	297.97		
203457_at	0.99	0.94	P,A	65.63	**5.48**	0.00	P	355.10	STX7	6q23.1
228671_at	0.99	0.93	P,A	47.73	**5.47**	0.00	P	258.17		1p36.22
243048_at	1.00	0.98	A	71.53	**5.47**	0.00	P	384.30	CECR7	22q11.2
239598_s_at	1.00	0.95	P	129.07	**5.47**	0.00	P	696.27	FLJ20481	16q13
216316_x_at	0.97	0.89	A	24.53	**5.47**	0.00	P,A	133.57	GK	xp21.3
223461_at	1.00	0.99	P	516.83	**5.46**	0.00	P	2777.30	TBC1D7	6p23
233350_s_at	1.00	0.97	M,A	79.03	**5.46**	0.00	P	425.13	ZSIG11	3p21.31
219113_x_at	0.99	0.92	A	31.87	**5.45**	0.00	P	172.43	DHRS10	19q13.33
241813_at	1.00	0.97	P,A	26.97	**5.45**	0.00	P	144.77	MBD1	18q21
220703_at	0.99	0.92	P	55.77	**5.45**	0.00	P	298.97	C10orf110	10p15.3
219464_at	0.99	0.95	A	123.47	**5.45**	0.00	P	659.23	CA14	1q21
236600_at	0.98	0.91	P,A	19.43	**5.44**	0.00	P	104.07	SPG20	13q13.2
205197_s_at	0.98	0.91	P,M	113.20	**5.43**	0.00	P	607.93	ATP7A	xq13.2-q13.3
215330_at	0.99	0.92	A	14.63	**5.43**	0.02	P,M	80.73		
242857_at	0.97	0.87	P	153.33	**5.39**	0.00	P	818.03		
219815_at	0.99	0.93	A	70.80	**5.38**	0.00	P	376.70	GAL3ST4	7q22
221962_s_at	0.97	0.88	A	170.50	**5.38**	0.00	P	918.23	UBE2H	7q32
52837_at	0.98	0.91	A	32.20	**5.38**	0.00	P	169.50	KIAA1644	22q13
235911_at	1.00	0.95	P,M	92.00	**5.37**	0.01	P	490.00		
205919_at	0.97	0.88	P,A	61.67	**5.35**	0.00	P	326.50	HBE1	11p15.5
232523_at	0.98	0.90	P	94.23	**5.34**	0.00	P	493.13	MEGF10	5q33
230747_s_at	1.00	0.96	P	30.10	**5.34**	0.00	P	159.17	FLJ33761	18q11.2
218799_at	1.00	0.95	P	73.13	**5.33**	0.00	P	382.33	FLJ10349	1p35.3
203710_at	0.98	0.89	A	15.67	**5.33**	0.00	P	82.77	ITPR1	3p26-p25
201801_s_at	0.99	0.94	P	160.67	**5.30**	0.00	P	843.00	SLC29A1	6p21.1-p21.2
209000_s_at	0.99	0.92	A	96.30	**5.30**	0.00	P	504.37	8-Sep	5q31
235429_at	0.99	0.94	P	47.63	**5.30**	0.00	P	247.53	EIF3S6	8q22-q23
1553192_at	0.99	0.92	A	12.83	**5.29**	0.00	P,M	67.40	ZNF441	19p13.2
242854_x_at	0.97	0.87	P,A	6.77	**5.29**	0.01	P	35.73	DLEU2	13q14.3
242554_at	0.99	0.94	P	37.07	**5.29**	0.01	P	195.93		
241803_s_at	1.00	0.96	P	34.57	**5.28**	0.00	P	179.80		20q11.21
202664_at	0.97	0.87	A	10.00	**5.27**	0.01	P	53.03	WASPIP	2q31.2
242976_at	0.99	0.92	A	16.30	**5.26**	0.00	P	84.30		
211547_s_at	0.95	0.84	P	74.40	**5.26**	0.00	P	389.93	PAFAH1B1	17p13.3
208213_s_at	1.00	0.95	A	30.77	**5.25**	0.00	P	158.97	KCNAB1	3q26.1
204992_s_at	1.00	0.99	P	405.60	**5.25**	0.00	P	2097.70	PFN2	3q25.1-q25.2
214787_at	0.99	0.92	P,A	18.37	**5.24**	0.00	P	95.07	IRLB	15q22.2
210647_x_at	1.00	0.96	P	118.20	**5.23**	0.00	P	612.00	PLA2G6	22q13.1
241252_at	0.98	0.89	M,A	15.53	**5.23**	0.00	P	80.00	LOC15757(	8p21.1
217161_x_at	0.98	0.89	A	34.97	**5.22**	0.00	P,A	178.37	AGC1	15q26.1
212394_at	0.95	0.85	A	26.23	**5.22**	0.00	P	135.73	KIAA0090	1p36.13
214692_s_at	0.89	0.76	A	25.97	**5.20**	0.03	P,M,A	140.13	JRK	8q24.3
201848_s_at	0.99	0.92	P,A	110.67	**5.20**	0.00	P	569.70	BNIP3	10q26.3
227628_at	0.99	0.95	P	285.63	**5.20**	0.00	P	1462.73		
228011_at	0.98	0.91	P,A	65.20	**5.20**	0.01	P	338.07	LOC137392	8q22.1
221510_s_at	1.00	0.95	P	384.63	**5.18**	0.00	P	1965.77	GLS	2q32-q34
233546_at	0.99	0.91	A	15.87	**5.18**	0.00	P	81.20		
227771_at	1.00	0.99	A	26.27	**5.18**	0.00	P	134.50	LIFR	5p13-p12
223339_at	0.99	0.95	P	116.63	**5.16**	0.00	P	593.03	ATPIF1	1p35.3
242617_at	0.98	0.91	P	137.40	**5.16**	0.00	P	701.17	LOC283578	14q24.3
225295_at	0.99	0.92	P,A	92.90	**5.16**	0.00	P	475.93	SLC39A10	2q33.1
235194_at	1.00	0.96	A	98.77	**5.15**	0.00	P	501.67		
219082_at	0.99	0.92	M,A	50.30	**5.15**	0.00	P	256.00	CGI-14	16p13.3
218598_at	1.00	0.96	P	207.17	**5.14**	0.00	P	1051.17	RINT-1	7q22.2
228471_at	0.99	0.94	A	25.97	**5.14**	0.01	P	132.93	LOC91526	2q33.1
202341_s_at	0.98	0.89	A	53.77	**5.12**	0.00	P	270.63	TRIM2	4q31.3
236831_at	0.96	0.86	P	110.57	**5.12**	0.00	P	560.97	C3orf6	3q28
229251_s_at	0.99	0.93	P	102.03	**5.12**	0.00	P	513.63	TPCN2	1q13.1-q13.2
210007_s_at	0.97	0.89	P	73.83	**5.12**	0.00	P	376.13	GPD2	2q24.1
203632_s_at	1.00	0.96	P,M,A	194.70	**5.11**	0.00	P	979.43	GPRC5B	16p12
236852_at	0.97	0.87	A	24.73	**5.11**	0.00	P	124.93		8q22.3
200827_at	0.95	0.85	P	170.13	**5.10**	0.00	P	846.40	PLOD	1p36.3-p36.2
217489_s_at	0.95	0.85	A	17.13	**5.09**	0.00	P	85.33	IL6R	1q21
241864_x_at	0.99	0.92	P	29.33	**5.09**	0.01	P	149.30		
1554918_a_at	0.98	0.90	P,A	73.70	**5.09**	0.00	P	372.13	ABCC4	13q32
231906_at	0.97	0.87	M,A	54.90	**5.07**	0.00	P	273.23	HOXD8	2q31.1
227536_at	0.95	0.85	P	85.90	**5.07**	0.00	P	425.93	KIAA0853	13q14.11
212061_at	0.99	0.92	P	90.10	**5.07**	0.00	P	452.90	SR140	3q23
229797_at	1.00	0.96	P	188.50	**5.07**	0.00	P	942.07	MCOLN3	1p22.3
235215_at	0.98	0.89	P	31.90	**5.06**	0.00	P	160.33		
220062_s_at	0.97	0.88	A	34.60	**5.05**	0.00	P	172.83	MAGEE1	xq27
225484_at	1.00	0.96	A	91.13	**5.03**	0.00	P	452.13	TSGA14	7q32
211876_x_at	0.98	0.91	A	21.53	**5.02**	0.01	P	109.20	PCDHGA11	5q31
205489_at	0.99	0.94	A	47.93	**5.02**	0.00	P,M	237.20	CRYM	6p13.11-p12.3
230241_at	1.00	0.95	A	28.63	**5.01**	0.00	P	141.63		
210841_s_at	0.94	0.83	A	42.47	**5.00**	0.00	P	213.37	NRP2	2q33.3

224848_at	0.99	0.95	P	131.90	**4.99**	0.00	P	648.77	CDK6	7q21-q22
230730_at	0.95	0.85	A	26.10	**4.99**	0.00	P	127.53	SGCD	5q33-q34
205198_s_at	0.96	0.86	P	89.57	**4.98**	0.00	P	436.77	ATP7A	q13.2-q13.3
236728_at	0.95	0.84	A	17.03	**4.98**	0.01	P	84.40	FLJ39485	5q15
1554274_a_at	0.97	0.88	A	16.37	**4.98**	0.00	P	80.73	SSH1	12q24.12
208002_s_at	1.00	0.96	P	438.67	**4.97**	0.00	P	2151.23	BACH	p36.31-p36.11
206352_s_at	0.99	0.92	A	43.40	**4.96**	0.00	P	210.97	PEX10	1p36.32
240721_at	0.96	0.87	P,A	17.40	**4.96**	0.00	P	85.70	DBC-1	8p22
231847_at	1.00	0.96	P	323.00	**4.95**	0.00	P	1569.57	LOC12913	22q13.1
49679_s_at	0.99	0.95	P	110.47	**4.95**	0.00	P	538.63	MMP24	20q11.2
216272_x_at	1.00	0.97	P,M,A	72.47	**4.94**	0.00	P	353.00	7h3	19p13.13
224963_at	0.99	0.93	P	194.77	**4.94**	0.00	P	952.03	SLC26A2	5q31-q34
242794_at	0.98	0.89	P	15.60	**4.94**	0.00	P	76.37	MAML3	4q28
211651_s_at	0.96	0.87	P	124.07	**4.94**	0.00	P	607.20	LAMB1	7q22
230697_at	0.99	0.93	A	24.27	**4.94**	0.00	P	117.77	BBS5	2q31.1
215811_at	0.99	0.94	A	48.93	**4.93**	0.00	P	236.80		
203759_at	1.00	0.96	P	110.00	**4.93**	0.00	P	534.57	SIAT4C	11q23-q24
221561_at	0.99	0.92	P	159.43	**4.93**	0.00	P	775.97	SOAT1	1q25
222420_s_at	0.99	0.92	P	331.53	**4.93**	0.00	P	1602.20	UBE2H	7q32
226433_at	0.97	0.89	A	17.30	**4.92**	0.00	P	83.50	RNF157	17q25.3
216299_s_at	0.98	0.90	A	31.23	**4.92**	0.00	P	151.67	XRCC3	14q32.3
236517_at	0.99	0.92	P	37.53	**4.91**	0.00	P	182.30	MEGF10	5q33
242931_at	0.99	0.95	P	102.73	**4.90**	0.00	P	496.50		
239624_at	0.98	0.91	P,A	60.53	**4.89**	0.00	P	289.77		
228205_at	1.00	0.96	P	161.10	**4.89**	0.00	P	774.03	TKT	3p14.3
207091_at	0.99	0.95	A	17.47	**4.88**	0.00	P	84.30	P2RX7	12q24
237365_at	1.00	0.96	A	21.83	**4.87**	0.00	P	104.70		
236745_at	0.98	0.91	A	16.77	**4.86**	0.00	P	80.77	FLJ34512	16p13.3
225383_at	1.00	0.96	M,A	86.23	**4.85**	0.00	P	412.73	ZNF275	xq28
205453_at	0.99	0.91	A	35.83	**4.85**	0.00	P	170.50	HOXB2	17q21-q22
218404_at	0.99	0.94	P	346.57	**4.85**	0.00	P	1658.87	SNX10	7p15.2
227126_at	0.99	0.93	P	72.30	**4.85**	0.00	P	346.10		
242338_at	0.98	0.91	A	24.27	**4.85**	0.01	P	118.50	FZp762C1	8q21.3
236660_at	0.95	0.84	A	17.20	**4.85**	0.00	P	82.70		
222470_s_at	0.97	0.87	A	30.20	**4.85**	0.00	P	144.90	C20orf44	20q11.23
1563182_at	0.98	0.90	P	21.30	**4.84**	0.01	P	102.80		
1552472_a_at	0.99	0.92	P	78.43	**4.84**	0.00	P	373.83	CENTB2	3q29
226056_at	1.00	0.97	P	88.27	**4.82**	0.00	P	419.37	CDGAP	3q13.33
202609_at	0.99	0.95	P	242.70	**4.81**	0.00	P	1151.67	EPS8	12q23-q24
233903_s_at	0.99	0.94	P	35.10	**4.81**	0.00	P	167.20	SGEF	3q25.2
203706_s_at	0.99	0.94	P	108.47	**4.80**	0.00	P	514.10	FZD7	2q33
228415_at	1.00	0.96	P	361.00	**4.80**	0.00	P	1706.33	AP1S2	Xp22.31
200958_s_at	1.00	0.97	P	1114.57	**4.80**	0.00	P	5272.57	SDCBP	8q12
208891_at	1.00	0.97	P	259.27	**4.80**	0.00	P	1221.57	DUSP6	12q22-q23
215720_s_at	0.99	0.94	A	16.00	**4.80**	0.01	P,M,A	76.63	NFYA	6p21.3
228539_at	0.96	0.87	A	16.33	**4.79**	0.01	P	78.97		
229250_at	0.98	0.91	P	233.30	**4.79**	0.00	P	1093.60	TPCN2	1q13.1-q13.2
205376_at	0.99	0.93	P	168.27	**4.79**	0.00	P	789.87	INPP4B	4q31.1
209611_s_at	1.00	0.98	A	87.97	**4.78**	0.00	P	418.70	SLC1A4	2p15-p13
233539_at	0.99	0.95	P,A	37.37	**4.78**	0.00	P	177.00	NAPE-PLD	7q22.1
1554084_a_at	1.00	0.99	A	111.80	**4.78**	0.00	P	526.47	FLJ23323	1p36.23
223095_at	0.99	0.95	P,M	148.23	**4.77**	0.00	P	701.17	MRVLDC1	10q24.2
225630_at	0.96	0.86	A	12.17	**4.77**	0.01	P,A	58.17	KIAA1706	7p14
212746_s_at	0.99	0.93	P,A	129.70	**4.76**	0.00	P	607.57	KAB	1q44
244190_at	0.99	0.94	P,A	15.97	**4.75**	0.00	P	74.73	THAP5	7q22.3
1552927_at	0.99	0.92	P	12.83	**4.75**	0.00	P	59.80	TAB3	xp21.3
217540_at	0.96	0.86	P	22.67	**4.75**	0.00	P	106.73		
212005_at	1.00	0.95	A	78.03	**4.74**	0.00	P	369.93	FZp566C0	1p36.13
230364_at	0.98	0.91	P	35.33	**4.74**	0.00	P	164.30	CHPT1	12q
239901_at	0.98	0.91	A	48.07	**4.74**	0.00	P	225.27		
235339_at	0.98	0.90	A	48.80	**4.74**	0.00	P	229.20	SETDB2	13q14
230220_at	0.93	0.82	A	13.67	**4.74**	0.01	P	64.13		
223488_s_at	0.95	0.84	A	89.70	**4.73**	0.00	P	422.87	GNB4	3q27.1
230722_at	0.99	0.94	M,A	53.00	**4.73**	0.00	P	246.90	BNC2	9p22.2
1552485_at	0.98	0.91	P,M	89.00	**4.72**	0.00	P	416.67	LACTB	15q22.1
219471_at	0.99	0.92	A	37.93	**4.72**	0.00	P	176.97	C13orf18	13q14.11
227626_at	0.98	0.91	P	43.77	**4.72**	0.00	P	203.27	C6orf33	6p12.1
218788_s_at	0.98	0.90	A	50.70	**4.72**	0.00	P,M	236.90	SMYD3	1q44
225860_at	1.00	0.97	P,M,A	198.27	**4.72**	0.00	P	923.10	MMP24	20q11.2
226250_at	0.95	0.84	A	14.67	**4.72**	0.01	P,A	68.77		
200757_s_at	1.00	0.97	P	790.00	**4.71**	0.00	P	3658.30	CALU	7q32
209283_at	1.00	0.98	P,A	315.03	**4.71**	0.00	P	1466.40	CRYAB	1q22.3-q23.1
238624_at	0.98	0.90	P	19.93	**4.70**	0.00	P	91.87	NLK	17q11.2
203158_s_at	0.99	0.92	A	36.07	**4.70**	0.00	P	167.13	GLS	2q32-q34
1555014_x_at	0.98	0.90	P,A	13.27	**4.70**	0.00	P	61.13		
211479_s_at	0.97	0.89	P,A	9.90	**4.70**	0.01	P	46.73	HTR2C	xq24
225796_at	1.00	0.98	P	58.43	**4.69**	0.00	P	270.00	PXK	3p21.2
1556186_s_at	1.00	0.97	P	65.87	**4.69**	0.00	P	306.17	KIAA0090	1p36.13
202545_at	0.96	0.86	P	110.53	**4.69**	0.00	P	517.20	PRKCD	3p21.31
206752_s_at	0.98	0.91	P,A	28.60	**4.69**	0.01	P	134.30	DFFB	1p36.3
202459_s_at	0.99	0.92	P	51.57	**4.68**	0.00	P	238.73	LPIN2	18p11.31
217247_at	0.97	0.87	A	13.40	**4.68**	0.02	P,M,A	64.43	RBM3	xp11.2
202430_s_at	0.98	0.91	P	325.27	**4.67**	0.00	P	1502.00	PLSCR1	3q23
1569403_at	0.97	0.88	P,A	19.83	**4.67**	0.00	P	91.00		
227080_at	1.00	0.96	P	117.07	**4.66**	0.00	P	534.97	MGC45731	1p11.2
234987_at	1.00	0.98	P	88.43	**4.65**	0.00	P	405.70		20q11.23
232195_at	0.99	0.94	A	64.37	**4.65**	0.00	P	296.20	GPR158	10p12.31
238229_at	0.94	0.83	A	11.90	**4.65**	0.00	P	55.30	MGC26979	8q22.1
207968_s_at	0.98	0.89	A	21.67	**4.65**	0.00	P,M	100.00	MEF2C	5q14
235391_at	1.00	0.96	P	123.07	**4.65**	0.00	P	563.27	LOC13739	8q22.1
223158_s_at	0.99	0.93	P	113.30	**4.64**	0.00	P	520.17	NEK6	q33.3-q34.11
227462_at	0.99	0.92	P	72.97	**4.63**	0.00	P	334.00	LRAP	16
224759_s_at	1.00	0.99	P	192.77	**4.62**	0.00	P	877.77	MGC17943	12q24.11
201636_at	1.00	0.96	P	178.20	**4.62**	0.00	P	811.03	FXR1	3q28
202364_at	1.00	0.98	P	405.33	**4.62**	0.00	P	1842.87	MXI1	10q24-q25
227746_at	0.96	0.87	P,A	33.77	**4.62**	0.00	P	154.87	ELAVL1	19p13.2
232099_at	0.99	0.93	P	77.53	**4.62**	0.00	P	355.47	PCDHB16	5q31
226575_at	0.96	0.87	M,A	59.50	**4.62**	0.01	P	270.87	ZNF462	9q32
221676_s_at	0.99	0.92	P	340.40	**4.61**	0.00	P	1551.33	CORO1C	12q24.1
232701_at	0.96	0.87	A	31.57	**4.60**	0.00	P	142.23		
231579_s_at	1.00	0.97	P	507.47	**4.60**	0.00	P	2300.07	TIMP2	17q25
201849_at	1.00	0.97	P	250.97	**4.60**	0.00	P	1136.73	BNIP3	10q26.3
1552946_at	0.97	0.88	A	22.20	**4.59**	0.01	P	101.73	MGC17986	19q13.33
213022_s_at	0.98	0.90	P,A	44.10	**4.59**	0.00	P	200.73	UTRN	6q24
222699_s_at	0.99	0.93	P	149.03	**4.58**	0.00	P	678.07	PLEKHF2	8q22.1
1569323_at	0.93	0.83	A	15.13	**4.58**	0.01	P	69.57	PTPRG	3p21-p14
226738_at	0.98	0.90	A	63.50	**4.58**	0.00	P	288.00	FLJ33817	17p13.3
1557915_s_at	1.00	0.96	P	811.77	**4.57**	0.00	P	3659.83	GSTO1	10q25.1
226767_s_at	1.00	0.99	P	81.10	**4.57**	0.00	P	366.50	FZP566J2	16p13.3
210963_s_at	1.00	0.96	P,A	158.83	**4.57**	0.00	P	716.70	GYG2	xp22.3
1555781_at	1.00	0.97	A	57.93	**4.56**	0.00	P	260.03	PQLC2	1p36.13
54037_at	1.00	0.98	P	82.63	**4.56**	0.01	P	374.83	HPS4	22cen-q12.3
213361_at	1.00	0.99	P	179.40	**4.56**	0.00	P	804.57	TDRD7	9q22.33
1558693_s_at	1.00	0.97	P	139.30	**4.55**	0.00	P	624.30	MGC31963	1q23.1
226274_at	0.99	0.94	P,A	132.87	**4.54**	0.00	P	594.43	LOC15856	xp11.23
237305_at	0.98	0.91	A	10.70	**4.54**	0.00	P	47.90		
235443_at	1.00	0.97	P	34.20	**4.53**	0.00	P	153.03		
204735_at	1.00	0.98	P	51.37	**4.53**	0.00	P	228.50	PDE4A	19p13.2
205066_s_at	0.96	0.86	A	23.57	**4.52**	0.00	P	105.77	ENPP1	6q22-q23
1552705_at	0.95	0.85	P	12.00	**4.52**	0.01	P	53.53	DUSP19	2q32.1
206234_s_at	0.99	0.92	A	24.07	**4.52**	0.00	P	106.50	MMP17	12q24.3
1554576_a_at	0.97	0.89	P,A	146.53	**4.51**	0.00	P	653.77	ETV4	17q21
204764_at	0.98	0.89	A	92.50	**4.51**	0.00	P	414.93	FNTB	14q23-q24
1555234_a_at	0.94	0.84	A	29.30	**4.50**	0.01	P	130.83		
229493_at	0.98	0.90	A	40.00	**4.50**	0.00	P	176.63		
201487_at	0.99	0.92	P	544.77	**4.49**	0.00	P	2417.97	CTSC	1q14.1-q14.3
218773_s_at	1.00	0.96	P	388.07	**4.49**	0.00	P	1710.70	MSRB	10p12
1552928_s_at	1.00	0.95	P,A	25.47	**4.49**	0.00	P	113.00	TAB3	xp21.3
36711_at	1.00	0.96	P	894.93	**4.48**	0.00	P	3938.10	MAFF	22q13.1
243589_at	0.99	0.93	P	21.53	**4.48**	0.00	P	95.23	LOC28405	17q21.32
206046_at	0.97	0.89	A	44.47	**4.47**	0.00	P	195.47	ADAM23	2q33
219469_at	0.96	0.85	A	22.27	**4.47**	0.00	P	98.73	DNCH2	11q21-q22.1
226093_at	1.00	0.98	A	18.17	**4.47**	0.00	P	79.77	DCP1B	12p13.33
209014_at	1.00	0.97	P	497.83	**4.47**	0.00	P	2186.53	MAGED1	xp11.23

203641_s_at	0.98	0.90	A	48.40	**4.46**	0.00	P	212.80	COBLL1	2q24.3
225401_at	1.00	0.99	P	215.37	**4.46**	0.00	P	944.70	MGC31963	1q23.1
201998_at	1.00	0.97	P	360.60	**4.45**	0.00	P	1578.60	SIAT1	3q27-q28
238250_at	1.00	0.95	A	20.70	**4.45**	0.01	P,M,A	92.70		
209088_s_at	0.98	0.90	P	92.50	**4.44**	0.00	P	402.80	UBN1	16p13.3
231576_at	0.95	0.85	P,A	40.87	**4.44**	0.00	P	181.57		
239511_s_at	0.97	0.88	P,A	14.20	**4.44**	0.00	P	62.60		
202976_s_at	1.00	0.95	P	209.17	**4.44**	0.00	P	914.97	RHOBTB3	5q15
218987_at	0.97	0.88	P	45.00	**4.43**	0.00	P	195.13	ATF7IP	12p13.2
209361_s_at	1.00	0.96	P,M	167.73	**4.43**	0.00	P	731.67	PCBP4	3p21
238905_at	0.97	0.89	A	37.27	**4.43**	0.00	P,A	161.93	RHOJ	14q23.2
213236_at	0.99	0.92	A	140.27	**4.43**	0.00	P	607.77	SASH1	6q24.3
224461_s_at	0.96	0.86	P,M	129.17	**4.42**	0.00	P	559.57	AMID	10q22.2
217437_s_at	0.99	0.93	P,A	91.43	**4.42**	0.00	P	400.23	TACC1	8p11
224959_at	1.00	0.96	P	559.80	**4.41**	0.00	P	2435.93	SLC26A2	5q31-q34
205771_s_at	0.99	0.94	A	30.47	**4.41**	0.00	P	132.20	AKAP7	6q23
213112_s_at	0.97	0.88	A	18.80	**4.41**	0.00	P	81.67	SQSTM1	5q35
225652_at	1.00	0.99	P,A	278.03	**4.41**	0.00	P	1206.27		
204995_at	0.99	0.94	P	49.77	**4.40**	0.00	P	216.47	CDK5R1	17q12
239870_at	0.98	0.91	A	29.40	**4.40**	0.00	P	126.90	SPATS1	6p21.1
222385_x_at	0.95	0.84	P,A	363.87	**4.40**	0.00	P	1608.40	SEC61A1	3q21.3
242888_at	1.00	0.95	P	101.93	**4.40**	0.00	P	440.20		
243502_at	0.94	0.83	A	17.87	**4.40**	0.02	P,A	80.33		
244356_at	0.94	0.82	P,A	8.70	**4.39**	0.02	P	39.57		
230201_at	0.99	0.94	P,A	21.53	**4.39**	0.01	P	93.97	FXR1	3q28
202139_at	0.99	0.94	P	186.87	**4.39**	0.00	P	809.30	AKR7A2	p35.1-p36.23
224599_at	1.00	0.98	P	500.37	**4.39**	0.00	P	2155.33	CGGBP1	3p12-p11.1
203819_s_at	0.97	0.89	P	159.40	**4.38**	0.00	P	694.60	IMP-3	7p11
231855_at	0.99	0.92	P	118.83	**4.38**	0.00	P	515.60	KIAA1524	3q13.13
239697_x_at	0.99	0.92	P,M	21.90	**4.38**	0.01	P	95.43	FLJ42117	3p21.2
226400_at	0.98	0.89	P	287.83	**4.37**	0.00	P	1246.10	CDC42	1p36.1
244007_at	1.00	0.96	P	40.13	**4.37**	0.01	P	175.53		
209967_s_at	0.99	0.95	P,A	71.47	**4.37**	0.00	P	308.87	CREM	10p11.21
224847_at	1.00	0.95	P	204.07	**4.36**	0.00	P	877.13	CDK6	7q21-q22
225161_at	0.99	0.93	P	252.33	**4.35**	0.00	P	1082.73	EFG1	3q25.1-q26.2
213639_s_at	0.99	0.93	A	23.17	**4.34**	0.00	P,A	98.70	ZNF500	16p13.3
221773_at	0.99	0.95	P	137.73	**4.33**	0.00	P	588.87	ELK3	12q23
223163_s_at	1.00	0.97	P	372.83	**4.33**	0.00	P	1586.40	NIPA	7q32.3
223107_s_at	0.98	0.89	P	158.77	**4.33**	0.00	P	671.73	PS1D	1p35.1
1569788_at	1.00	0.95	P	16.50	**4.32**	0.01	P	70.93		
226487_at	1.00	0.99	P,A	47.07	**4.31**	0.00	P	199.63	FLJ14721	12q24.12
226229_s_at	0.99	0.93	A	42.50	**4.31**	0.01	P	180.77	HSPC182	1p36.33
209838_at	0.99	0.95	P	57.70	**4.30**	0.00	P	244.40	TRIP15	15q21.2
229017_s_at	0.98	0.91	P	122.57	**4.30**	0.00	P	521.97	DustyPK	1q32.1
243492_at	0.98	0.90	P,A	65.83	**4.30**	0.00	P	278.83	CTMP	1q21
241164_at	0.98	0.91	P,A	21.07	**4.30**	0.00	P	89.70		
1553679_s_at	0.97	0.88	P,A	26.30	**4.29**	0.00	P	112.17	VKORC1L1	7q11.21
203047_at	1.00	0.98	A	120.67	**4.29**	0.00	P	509.03	STK10	5q35.1
204691_x_at	1.00	0.96	P	118.80	**4.29**	0.00	P	504.63	PLA2G6	22q13.1
203723_at	0.99	0.93	A	67.93	**4.28**	0.00	P	285.57	ITPKB	1q42.13
223772_s_at	0.99	0.95	P	39.70	**4.28**	0.01	P	168.20	KFZP564G2(	15q14
202536_at	1.00	0.99	P	272.70	**4.27**	0.00	P	1148.10	KFZP564O1	3p12.1
1554168_a_at	0.99	0.93	P,A	248.50	**4.26**	0.00	P	1046.67	SH3KBP1	p22.1-p21.3
242665_at	1.00	0.97	P	184.27	**4.26**	0.01	P	787.13	FMNL2	2q24.1
1558458_at	1.00	0.96	A	9.23	**4.25**	0.00	P,A	38.63		7
213490_s_at	1.00	0.97	P,A	147.70	**4.24**	0.00	P	618.90	MAP2K2	19p13.3
218789_s_at	0.99	0.93	M,A	41.37	**4.23**	0.00	P	173.17	FLJ20010	1q14.2-q14.3
215533_s_at	1.00	0.98	P,A	53.57	**4.22**	0.00	P	222.83	UBE4B	1p36.3
200661_at	0.99	0.93	P,A	479.93	**4.22**	0.00	P	1986.73	PPGB	20q13.1
229236_s_at	0.99	0.93	P	161.87	**4.22**	0.00	P	672.97	SFXN4	10q26.13
1552274_at	1.00	0.97	A	60.87	**4.21**	0.00	P	252.50	PXK	3p21.2
1554251_at	1.00	0.97	A	70.70	**4.21**	0.00	P	293.23	HP1-BP74	1p36.12
218196_at	1.00	1.00	P	406.20	**4.21**	0.00	P	1680.50	OSTM1	6q21
227351_at	0.97	0.87	P	34.30	**4.21**	0.00	P	142.30	LOC146174	16p12.3
232263_at	1.00	0.96	A	15.20	**4.20**	0.00	P	63.33	SLC6A15	12q21.3
217960_s_at	0.97	0.89	P	336.80	**4.20**	0.00	P	1398.93	TOMM22	22q12-q13
212959_s_at	1.00	0.97	P	399.37	**4.20**	0.00	P	1647.57	MGC4170	12q23.3
213256_at	1.00	0.97	A	31.80	**4.20**	0.00	P	131.53	MGC48332	5q23.3
242017_at	0.99	0.93	A	20.97	**4.20**	0.00	P	86.33		
203441_s_at	0.99	0.93	A	40.50	**4.19**	0.00	P	167.70	CDH2	18q11.2
214070_s_at	0.99	0.92	A	65.73	**4.19**	0.00	P	273.83	ATP10B	5q34
222412_s_at	0.99	0.94	P	347.97	**4.19**	0.00	P	1439.67	SSR3	3q25.31
1552519_at	0.97	0.88	P	36.10	**4.19**	0.00	P	148.67	ACVR1C	2q24.2
212090_at	1.00	0.95	P	279.20	**4.19**	0.00	P	1151.20	GRINA	8q24.3
205219_s_at	0.98	0.89	P	77.80	**4.19**	0.00	P	322.17	GALK2	15q15.3
207088_s_at	0.98	0.91	P	278.83	**4.19**	0.00	P	1156.43	SLC25A11	17p13.3
1555705_a_at	1.00	0.98	A	69.77	**4.18**	0.00	P	287.43	CKLFSF3	16q22.1
1552789_at	0.98	0.90	P,A	10.60	**4.18**	0.03	P	46.63	FLJ32803	3q26.31
219677_at	0.98	0.91	A	52.67	**4.18**	0.00	P	217.80	SSB1	1p36.22
210757_x_at	0.98	0.91	A	122.33	**4.18**	0.00	P	505.93	DAB2	5p13
219703_at	0.99	0.94	P	85.87	**4.18**	0.00	P	355.63	MNS1	15q21.2
1569025_s_at	0.95	0.85	A	13.07	**4.18**	0.02	P	56.33		4q22.1
225157_at	1.00	0.97	P	166.73	**4.18**	0.00	P	684.80		
1570255_s_at	0.96	0.87	P,A	13.77	**4.17**	0.02	P	58.43	ANKRD20A	9p11.2
220078_at	1.00	0.96	P	33.40	**4.17**	0.01	P	138.23	USP48	1p36.12
229061_s_at	0.98	0.91	P	225.53	**4.17**	0.00	P	928.67	SLC25A13	7q21.3
217097_s_at	1.00	0.96	P	41.63	**4.16**	0.01	P	173.67	PHTF2	7q11.23-q21
223814_at	0.98	0.90	P,A	33.60	**4.16**	0.00	P	138.83	TRNT1	3p25.1
227960_s_at	1.00	0.98	P	321.03	**4.16**	0.00	P	1315.23	KFZP566J2(	16p13.3
219654_at	0.99	0.94	P	224.97	**4.16**	0.00	P	922.67	PTPLA	10p14-p13
1555039_a_at	0.99	0.94	P	46.90	**4.16**	0.00	P	193.67	ABCC4	13q32
232614_at	0.97	0.88	A	9.40	**4.16**	0.01	P,A	38.60		
244873_s_at	0.98	0.89	A	14.33	**4.15**	0.01	P	59.63		
208918_s_at	1.00	0.97	P	191.10	**4.15**	0.00	P	781.50	FLJ13052	p36.33-p36.21
216005_at	1.00	0.97	P	19.93	**4.15**	0.00	P	81.53	TNC	9q33
1557293_at	0.97	0.88	P	29.03	**4.15**	0.01	P	120.07		
216466_at	1.00	0.97	A	4.47	**4.14**	0.00	P	18.33	NAV3	
220952_s_at	1.00	0.98	P	208.60	**4.14**	0.00	P	848.77	PEPP2	12p12
235775_at	1.00	0.96	P	119.53	**4.14**	0.00	P	491.57	KFZp762A2	12q21.31
202825_at	1.00	0.97	P	230.63	**4.13**	0.00	P	938.57	SLC25A4	4q35
244251_at	0.99	0.93	A	14.70	**4.13**	0.00	P,M	60.13	LCP2	5q33.1-qter
219428_s_at	1.00	1.00	P	154.70	**4.13**	0.00	P	628.27	PXMP4	20q11.22
1557060_at	0.99	0.93	A	25.00	**4.13**	0.00	P	101.90	LOC148756	1q25.1
201635_s_at	0.97	0.88	P	324.70	**4.13**	0.00	P	1328.20	FXR1	3q28
1554311_a_at	0.98	0.90	P,A	13.90	**4.13**	0.01	P	58.50	SUPT6H	17q11.2
229100_s_at	0.99	0.94	A	31.33	**4.12**	0.01	P	127.97	TIMM22	17p13
1555216_a_at	0.95	0.84	A	34.03	**4.12**	0.00	P	136.97		
1554785_at	0.97	0.88	P,A	18.97	**4.12**	0.00	P	77.40	FLJ23518	11q21
226155_at	1.00	0.96	P	151.63	**4.12**	0.00	P	615.67	KIAA1600	10q26.11
232149_s_at	0.95	0.85	P	73.00	**4.12**	0.00	P	298.97	NSMAF	8q12-q13
219210_s_at	0.99	0.94	A	71.67	**4.11**	0.00	P	291.83	RAB8B	15q22.1
232568_at	1.00	0.97	P,A	32.17	**4.11**	0.02	P	133.37	MGC24103	9p22.2
228693_at	0.99	0.95	P	135.03	**4.11**	0.00	P	546.00	C3orf6	3q28
225726_s_at	0.99	0.92	P	176.33	**4.11**	0.00	P	710.37	PLEKHH1	14q24.1
202570_s_at	1.00	0.99	P	76.47	**4.11**	0.01	P	311.27	DLGAP4	20q11.23
210015_s_at	0.99	0.92	P,A	50.50	**4.10**	0.00	P	203.90	MAP2	2q34-q35
242251_at	0.98	0.90	P,A	60.20	**4.10**	0.01	P	245.47		
224663_s_at	1.00	0.96	P	187.57	**4.10**	0.00	P	757.33	CFL2	14q12
210493_s_at	0.99	0.94	P,M	26.53	**4.10**	0.00	P	107.27	KIAA0626	4q32.3
213397_x_at	0.98	0.89	P	27.60	**4.10**	0.01	P	111.93	RNASE4	14q11.1
1554082_a_at	1.00	0.98	P	130.43	**4.10**	0.00	P	526.57	FLJ23323	1p36.23
207541_s_at	1.00	0.99	P	116.23	**4.09**	0.00	P	467.83	PMSCL2	1p36.22
218548_x_at	1.00	0.97	P	128.00	**4.09**	0.00	P	516.17	ZSIG11	3p21.31
208732_at	0.99	0.94	P	177.40	**4.08**	0.00	P	714.70	RAB2	8q12.1
217632_at	0.98	0.91	P,A	19.67	**4.08**	0.00	P	79.73		
216205_s_at	0.98	0.90	P	101.03	**4.08**	0.00	P	411.10	MFN2	1p36.21
217935_s_at	1.00	0.97	P,A	165.93	**4.08**	0.00	P	666.93	C20orf44	20q11.23
200793_s_at	0.98	0.91	P	526.33	**4.07**	0.00	P	2120.93	ACO2	2q13.2-q13.31
1554864_a_at	0.96	0.86	A	18.13	**4.07**	0.00	P	73.97	SDC3	1pter-p22.3
242778_at	1.00	0.97	P	22.27	**4.06**	0.01	P	90.60	LPXN	11q12.1
214663_at	0.99	0.95	P	117.20	**4.06**	0.00	P	469.07	DustyPK	1q32.1
230280_at	0.97	0.88	P	42.33	**4.06**	0.00	P	168.77	TRIM9	14q22.1
215938_s_at	0.99	0.93	A	49.83	**4.06**	0.00	P	200.60	PLA2G6	22q13.1
213246_at	0.99	0.92	P	661.60	**4.06**	0.00	P	2654.27	C14orf109	14q32.13

243332_at	0.97	0.88	A	11.33	**4.06**	0.03	P	47.67		
235783_at	0.99	0.94	P,M	120.27	**4.06**	0.00	P	480.83		
205687_at	0.99	0.92	P	90.83	**4.06**	0.00	P	364.10	UBPH	16p12
243746_at	0.99	0.93	A	13.00	**4.05**	0.00	P	51.90	IGHMBP2	1q13.2-q13.4
218516_s_at	0.98	0.90	P	104.47	**4.05**	0.00	P	419.30	FLJ20421	8q11.23
226184_at	0.97	0.89	P	412.83	**4.04**	0.00	P	1651.60	FMNL2	2q24.1
1559052_s_at	0.96	0.86	P	88.93	**4.04**	0.00	P	360.20	PAK2	3q29
218981_at	0.98	0.90	P	142.70	**4.04**	0.00	P	572.67	ACN9	7q22.1
207387_s_at	0.99	0.95	M,A	42.00	**4.03**	0.00	P	167.00	GK	xp21.3
227889_at	0.99	0.93	P	302.27	**4.03**	0.00	P	1203.10	CAPNS2	16q13
214447_at	0.99	0.95	A	28.77	**4.03**	0.00	P	113.70	ETS1	11q23.3
238422_at	0.99	0.95	A	50.40	**4.03**	0.00	P	200.73	LOC151534	2p13.1
222288_at	1.00	0.96	P	121.90	**4.01**	0.01	P	485.20		
227554_at	0.98	0.89	P	96.47	**4.01**	0.00	P	378.37		7
1569495_at	1.00	0.99	A	31.77	**4.01**	0.00	P,A	125.57	FLJ30655	4q28.2
59705_at	1.00	0.99	P	40.47	**4.01**	0.00	P	159.93	SCLY	2q37.3
242138_at	0.99	0.94	P	94.80	**4.00**	0.00	P	374.40	DLX1	2q32
223234_at	1.00	0.96	P	186.80	**4.00**	0.00	P	738.03	MAD2L2	1p36
229371_at	1.00	0.96	P,A	54.60	**4.00**	0.00	P	215.73		
219312_s_at	0.99	0.94	P	49.30	**4.00**	0.00	P	194.57	ZBTB10	8q13-q21.1
201481_s_at	0.99	0.94	M,A	427.60	**4.00**	0.00	P	1686.13	PYGB	0p11.2-p11.1
1554026_a_at	0.99	0.92	A	49.67	**4.00**	0.01	P	196.50	MYO10	5p15.1-p14.3
224932_at	1.00	0.97	P	881.80	**3.99**	0.00	P	3462.43	LOC40091	22q11.23
213848_at	1.00	0.96	P	111.63	**3.99**	0.00	P	438.80	DUSP7	3p21
227293_at	1.00	0.95	P	43.80	**3.98**	0.00	P	171.17	LNX	4q12
222116_s_at	1.00	0.97	P	476.30	**3.98**	0.00	P	1864.63	TBC1D16	17q25.3
218007_s_at	1.00	0.99	P	1077.93	**3.98**	0.00	P	4216.90	RPS27L	15q22.1
229549_at	1.00	0.96	P	197.63	**3.98**	0.00	P	771.17	CALU	7q32
224002_s_at	0.99	0.92	P	39.03	**3.97**	0.00	P	153.47	FKBP7	2q31.3
244660_at	0.95	0.85	P,A	12.83	**3.97**	0.00	P	51.00	ELAVL1	19p13.2
207680_x_at	1.00	0.95	A	22.60	**3.96**	0.00	P	88.60	PAX3	2q35
221935_s_at	1.00	0.98	P	114.07	**3.96**	0.00	P	445.67	MGC34132	3p14.2
203542_s_at	1.00	0.97	A	33.93	**3.96**	0.00	P	132.10	BTEB1	9q13
213056_at	1.00	0.98	P	168.87	**3.95**	0.00	P	655.50	GRSP1	3p14.2
203269_at	0.99	0.92	P	267.43	**3.94**	0.00	P	1042.20	NSMAF	8q12-q13
203736_s_at	0.96	0.86	P,A	27.37	**3.94**	0.00	P	107.97	PPFIBP1	12p12.1
212117_at	0.99	0.93	P	189.37	**3.94**	0.00	P	736.83	RHOQ	2p21
205679_x_at	1.00	0.97	A	51.43	**3.94**	0.00	P	199.07	AGC1	15q26.1
205090_s_at	1.00	0.97	P	75.80	**3.93**	0.00	P	295.30	NAGPA	16p13.3
1555154_a_at	0.99	0.94	P,M	48.90	**3.93**	0.01	P	193.07	QKI	6q26-27
1566825_at	0.97	0.87	A	10.97	**3.92**	0.00	P,M	42.03		
1569701_at	1.00	0.95	P	21.10	**3.92**	0.00	P	82.00	PER3	1p36.23
1558801_at	0.98	0.91	P	135.60	**3.92**	0.00	P	525.60		
211515_s_at	0.96	0.86	P	64.43	**3.92**	0.00	P	252.50	DustyPK	1q32.1
224352_s_at	1.00	0.96	P	102.43	**3.92**	0.00	P	395.17	CFL2	14q12
213549_at	0.99	0.95	P	85.60	**3.91**	0.00	P	331.03	SLC18A2	10q25
203157_s_at	0.99	0.92	A	54.03	**3.91**	0.00	P	208.57	GLS	2q32-q34
1552660_a_at	0.98	0.91	P	116.23	**3.90**	0.00	P	450.87	FLJ11193	5p13.3
219033_at	0.96	0.86	A	41.43	**3.90**	0.00	P	158.10	FLJ21308	5q11.2
201089_at	1.00	0.95	P	630.03	**3.90**	0.00	P	2426.07	ATP6V1B2	8p22-p21
1558815_at	0.99	0.92	A	15.20	**3.89**	0.01	P	59.57	ARGBP2	4q35.1
222121_at	0.98	0.90	P	29.33	**3.89**	0.00	P	112.73	SGEF	3q25.2
1556704_s_at	1.00	0.96	A	8.17	**3.89**	0.01	P	31.50	LOC286297	9q12
1560265_at	0.97	0.88	P,A	16.27	**3.88**	0.01	P	63.30		
215079_at	0.99	0.94	A	22.33	**3.88**	0.00	P,M	85.97		7
236219_at	0.99	0.95	P	34.93	**3.88**	0.00	P	133.10	TMEM20	10q23.33
242635_s_at	0.98	0.90	P	29.57	**3.87**	0.00	P	113.07	NAPE-PLD	7q22.1
225158_at	0.98	0.90	P	282.07	**3.87**	0.00	P	1081.97	EFG1	3q25.1-q26.2
214279_s_at	0.97	0.89	A	32.37	**3.87**	0.01	P	126.10	NDRG2	14q11.2
226773_at	1.00	0.98	P,A	94.83	**3.87**	0.00	P	360.90		
223376_s_at	0.99	0.94	P	810.00	**3.86**	0.00	P	3071.67	BRI3	7q22.1
212811_x_at	1.00	0.96	P	395.13	**3.86**	0.00	P	1504.90	SLC1A4	2p15-p13
235197_s_at	1.00	0.98	P	67.83	**3.86**	0.00	P	258.27		
1558766_at	0.96	0.86	A	17.00	**3.86**	0.00	P,A	65.07		
227335_at	0.99	0.93	P	141.17	**3.86**	0.00	P	537.23	C20orf158	20q13.33
226844_at	0.99	0.94	P,M	55.10	**3.85**	0.00	P	209.80	MOBKL2B	9p21.1
235709_at	0.99	0.94	A	186.00	**3.85**	0.00	P	707.63	LOC283431	12q23.3
207836_s_at	1.00	0.97	P	56.47	**3.84**	0.00	P	214.03	RBPMS	8p12-p11
229246_at	0.99	0.94	P,A	25.77	**3.84**	0.01	P,M	99.23	SFRS1	17q21.3-q22
229869_at	0.99	0.94	A	26.80	**3.84**	0.01	P	102.47		
227580_s_at	0.99	0.92	P	146.30	**3.84**	0.00	P	551.60	FZP434B0	7q22.1
203217_s_at	0.99	0.93	P,A	229.53	**3.84**	0.00	P	864.80	SIAT9	2p11.2
211698_at	1.00	0.97	P	536.50	**3.83**	0.00	P	2027.30	CRI1	5q21.1-q21.2
204210_s_at	0.99	0.93	A	33.03	**3.83**	0.00	P	124.70	PCYT1A	3q29
225202_at	0.98	0.91	P	482.53	**3.83**	0.00	P	1827.03	RHOBTB3	5q15
239208_s_at	0.98	0.90	P,A	55.00	**3.83**	0.00	P	208.57		
223173_at	1.00	0.95	P	207.23	**3.83**	0.00	P	783.90	SPINL	16p12.1
220482_s_at	1.00	0.95	P,A	31.90	**3.83**	0.01	P	122.53	DELGEF	11p14.3
238000_at	1.00	0.99	P	32.93	**3.83**	0.00	P	124.20		
212658_at	1.00	0.98	P	207.23	**3.83**	0.00	P	780.37	LHFPL2	5q14.1
221591_s_at	1.00	0.99	P	381.27	**3.83**	0.00	P	1435.60	FLJ10156	17p13.2
221489_s_at	1.00	0.97	P	279.23	**3.83**	0.00	P	1051.03	SPRY4	5q31.3
1554690_a_at	1.00	0.97	P,A	55.23	**3.82**	0.01	P	210.30	TACC1	8p11
206907_at	0.99	0.92	P	43.60	**3.82**	0.00	P	163.67	TNFSF9	19p13.3
202675_at	1.00	0.96	P	319.93	**3.82**	0.00	P	1201.73	SDHB	1p36.1-p35
202158_s_at	1.00	0.97	P	237.33	**3.82**	0.00	P	893.53	CUGBP2	10p13
226713_at	0.99	0.93	P	169.83	**3.81**	0.00	P	638.93	C3orf6	3q28
202975_s_at	0.99	0.93	P	445.30	**3.81**	0.00	P	1673.87	RHOBTB3	5q15
207938_at	0.97	0.89	A	25.80	**3.80**	0.00	P	97.70	PI15	8q13.3
226532_at	1.00	0.96	P	112.50	**3.80**	0.00	P	421.83		
240926_at	0.97	0.88	P,A	21.77	**3.80**	0.00	P	82.53	MGC4126	3q29
212119_at	1.00	0.98	P,M	408.67	**3.80**	0.00	P	1525.07	RHOQ	2p21
204624_at	0.98	0.91	P	62.23	**3.80**	0.00	P	233.20	ATP7B	13q14.2-q21
209900_s_at	1.00	0.98	P	84.20	**3.79**	0.00	P	314.90	SLC16A1	1p12
230426_at	1.00	0.95	P	128.07	**3.79**	0.00	P	479.13	DLD	7q31-q32
212632_at	0.99	0.95	P	271.80	**3.79**	0.00	P	1012.63		
202446_s_at	0.99	0.94	P	790.80	**3.79**	0.00	P	2961.43	PLSCR1	3q23
235759_at	0.98	0.90	P,A	16.10	**3.78**	0.01	P	60.10	EFCBP1	8q21.3
202132_at	1.00	0.97	P	136.07	**3.78**	0.00	P	507.27	TAZ	3q23-q24
224833_at	1.00	0.96	P	359.70	**3.78**	0.00	P	1336.93	ETS1	11q23.3
1554691_a_at	0.98	0.91	P	310.17	**3.78**	0.00	P	1170.33	PACSIN2	2q13.2-13.33
243829_at	0.99	0.93	P	26.57	**3.77**	0.00	P	99.37	BRAF	7q34
217997_at	1.00	0.99	P	127.80	**3.77**	0.00	P	474.33	PHLDA1	12q15
243591_at	0.97	0.87	A	44.20	**3.77**	0.01	P	167.00		
243846_x_at	0.97	0.88	P,A	23.33	**3.77**	0.01	P	87.13		
226794_at	1.00	0.98	P	43.17	**3.76**	0.00	P	160.30	STXBP5	6q24.3
209234_at	0.98	0.91	P,M,A	89.50	**3.76**	0.00	P	332.93	KIF1B	1p36.2
214776_x_at	0.98	0.91	P,M,A	7.00	**3.76**	0.00	P	25.90	XYLB	3p22-p21.3
205084_at	0.99	0.94	P	131.73	**3.76**	0.00	P	490.30	BCAP29	7q22-q31
241542_at	0.98	0.89	A	23.67	**3.76**	0.01	P	88.23		
221986_s_at	0.98	0.90	P	34.17	**3.75**	0.00	P	126.67	DRE1	3q27.3
209504_s_at	0.99	0.95	A	82.27	**3.74**	0.00	P	303.80	PLEKHB1	1q13.5-q14.1
1559307_s_at	0.97	0.88	P	17.63	**3.74**	0.00	P	65.33	RBL1	20q11.2
200982_s_at	1.00	0.96	P,A	331.87	**3.74**	0.00	P	1220.27	ANXA6	5q32-q34
203304_at	0.99	0.95	P	428.73	**3.74**	0.00	P	1572.93	BAMBI	0p12.3-p11.2
230652_at	0.95	0.84	P,A	18.80	**3.74**	0.01	P	70.07	TIMP1	p11.3-p11.23
205296_at	0.99	0.94	P	107.57	**3.74**	0.00	P	397.07	RBL1	20q11.2
242769_at	0.99	0.95	P	20.93	**3.74**	0.00	P	77.10		
215968_at	0.97	0.88	P,A	20.67	**3.74**	0.00	P	76.57		
235174_s_at	1.00	0.97	P	116.37	**3.72**	0.00	P	428.73		
243945_at	0.98	0.91	A	8.67	**3.72**	0.00	P	31.90	TRIM2	4q31.3
212402_at	0.99	0.94	P	161.53	**3.72**	0.00	P	593.80	KIAA0853	13q14.11
222798_at	0.99	0.94	P	108.73	**3.72**	0.01	P	401.73	PTER	10p12
201066_at	0.99	0.94	P	882.63	**3.71**	0.00	P	3239.27	CYC1	8q24.3
204017_at	1.00	0.98	P	251.97	**3.71**	0.00	P	919.67	KDELR3	22q13.1
201802_at	0.99	0.95	P	162.43	**3.71**	0.00	P	594.70	SLC29A1	p21.1-p21.2
203820_s_at	0.99	0.94	P	237.77	**3.71**	0.00	P	871.40	IMP-3	7p11
212476_at	1.00	0.97	P	374.07	**3.71**	0.01	P	1371.60	CENTB2	3q29
218847_at	1.00	0.96	P	214.17	**3.71**	0.00	P	780.90	IMP-2	3q28
218413_s_at	0.96	0.85	A	10.73	**3.70**	0.03	P,A	40.33	ANC_2H01	3q27.1
243189_at	0.99	0.94	M,A	25.60	**3.70**	0.00	P,A	92.97	NRF1	7q32
217983_s_at	1.00	0.97	P	194.27	**3.69**	0.00	P	705.93	RNASET2	6q27
218176_at	0.99	0.93	P	250.73	**3.68**	0.00	P	913.20	MAGEF1	3q13
206917_at	0.99	0.94	A	41.80	**3.68**	0.00	P,A	153.27	GNA13	17q24.3

227167_s_at	0.99	0.94	P	329.90	**3.68**	0.00	P	1199.20		
213107_at	0.99	0.94	P	67.97	**3.68**	0.00	P	245.53	TNIK	3q26.31
238756_at	1.00	0.97	P	414.67	**3.68**	0.00	P	1502.37	LOC283431	12q23.3
229342_at	0.99	0.93	P,M	39.27	**3.67**	0.00	P	142.07		
40420_at	0.99	0.93	P	159.20	**3.67**	0.00	P	573.27	STK10	5q35.1
212419_at	0.99	0.92	A	71.77	**3.67**	0.00	P	260.03	C10orf56	10q23.1
209307_at	0.97	0.88	P	286.23	**3.67**	0.00	P	1040.10	SWAP70	11p15
203223_at	1.00	0.96	P,A	31.33	**3.67**	0.02	P	115.87	RABEP1	17p13.3
241534_at	0.99	0.94	P,A	12.13	**3.67**	0.01	P	44.27	ATP8B1	18q21
227408_s_at	0.99	0.95	P	69.90	**3.67**	0.00	P	251.80	SNX25	4q35.1
65585_at	1.00	0.95	P	145.73	**3.66**	0.01	P	534.07	MGC16279	8p23.1
242844_at	0.98	0.91	P	98.53	**3.66**	0.00	P	356.30	PGGT1B	5q23.1
202581_at	1.00	0.97	P	589.83	**3.66**	0.00	P	2122.77	HSPA1B	6p21.3
205142_x_at	1.00	0.98	P,A	126.87	**3.66**	0.00	P	457.83	ABCD1	xq28
212459_x_at	1.00	0.97	P	412.83	**3.66**	0.00	P	1484.53	SUCLG2	3p14.3
201506_at	1.00	0.99	P	288.30	**3.65**	0.00	P	1036.13	TGFBI	5q31
223220_s_at	0.99	0.92	P	174.03	**3.65**	0.00	P	627.43	BAL	3q13-q21
205492_s_at	0.96	0.85	A	16.10	**3.65**	0.00	P,A	58.33	DPYSL4	10q26
38037_at	0.96	0.86	A	44.70	**3.65**	0.00	P	159.40	DTR	5q23
242474_s_at	1.00	0.96	A	24.87	**3.65**	0.01	P	90.57	LOC203547	xq28
234975_at	1.00	0.97	P	44.27	**3.65**	0.00	P	159.17	GSPT1	16p13.1
222206_s_at	1.00	0.98	P	112.47	**3.65**	0.00	P	404.87	LOC56926	19p13.3
242450_at	0.99	0.92	A	10.70	**3.64**	0.00	P	38.30	RGMB	5q21.1
242308_at	0.99	0.93	A	68.17	**3.64**	0.01	P	248.70	MCOLN3	1p22.3
227700_x_at	1.00	1.00	P	208.87	**3.64**	0.00	P	750.33	ATAD3A	1p36.33
208964_s_at	1.00	0.97	P	226.93	**3.64**	0.00	P	810.10	FADS1	1q12.2-q13.1
219648_at	1.00	0.97	P	518.97	**3.63**	0.00	P	1856.63	FLJ10116	2q35
225171_at	1.00	0.95	P	186.07	**3.63**	0.00	P	665.60	ARHGAP18	6q23.1
225331_at	1.00	0.96	P	580.87	**3.63**	0.00	P	2081.83	C3orf6	3q28
215772_x_at	1.00	0.96	P	421.03	**3.62**	0.00	P	1502.90	SUCLG2	3p14.3
235875_at	1.00	0.98	P,A	62.17	**3.62**	0.01	P	224.10		
240466_at	0.96	0.87	A	13.30	**3.62**	0.00	P	47.87		
200765_x_at	1.00	0.96	P	349.87	**3.62**	0.00	P	1248.27	CTNNA1	5q31
200824_at	1.00	0.97	P	862.10	**3.62**	0.00	P	3075.30	GSTP1	11q13
233899_x_at	1.00	0.96	P	65.87	**3.62**	0.00	P	235.43	ZBTB10	8q13-q21.1
218330_s_at	1.00	0.97	P	270.83	**3.62**	0.01	P	967.20	NAV2	11p15.1
213109_at	0.97	0.87	P	60.50	**3.62**	0.00	P	214.10	TNIK	3q26.31
221638_s_at	0.99	0.94	A	28.60	**3.62**	0.00	P	102.43	STX16	20q13.32
1569133_x_at	0.97	0.88	P,A	18.80	**3.61**	0.00	P	67.27	KFZp313G17	5q15
201470_at	0.99	0.94	P	1264.87	**3.61**	0.00	P	4477.57	GSTO1	10q25.1
208460_at	1.00	0.98	M,A	63.00	**3.61**	0.01	P	227.37	GJA7	17q21.31
225882_at	0.98	0.90	P	77.07	**3.61**	0.00	P	275.13	SLC35B4	7q33
239258_at	0.98	0.89	P	20.67	**3.60**	0.02	P	74.40	RHOQ	2p21
209625_at	1.00	0.96	P,A	96.97	**3.60**	0.00	P	343.27	PIGH	14q11-q24
235227_at	0.97	0.88	A	29.73	**3.60**	0.00	P	104.60		
206308_at	0.95	0.84	P,A	35.67	**3.60**	0.01	P	128.57	DNMT2	10p15.1
238444_at	0.98	0.90	A	10.93	**3.59**	0.02	P,M	39.60	ZNF618	9q33.1
223700_at	0.99	0.94	P	119.00	**3.59**	0.01	P	424.57	GAJ	4q31.3
219051_x_at	1.00	0.96	P,M	156.83	**3.59**	0.02	P	566.73	C16orf23	16p13.3
229327_s_at	0.97	0.88	A	4.47	**3.59**	0.00	P	15.97	MAF	16q22-q23
239614_x_at	0.96	0.86	P,M	20.63	**3.59**	0.00	P	73.63		
63825_at	0.99	0.94	P	167.60	**3.59**	0.00	P	594.67	ABHD2	15q26.1
225531_at	1.00	0.97	A	177.97	**3.59**	0.01	P	634.53	CABLES1	18q11.2
232983_s_at	1.00	0.98	P	117.00	**3.59**	0.00	P	413.80	DELGEF	11p14.3
217992_s_at	0.99	0.95	P	191.53	**3.58**	0.00	P	677.33	EFHD2	1p36.13
202032_s_at	0.98	0.91	P	274.53	**3.58**	0.00	P	961.67	MAN2A2	15q26.1
217118_s_at	1.00	0.99	P	237.93	**3.58**	0.00	P	837.67	KIAA0930	22q13.31
218402_s_at	1.00	0.97	A	125.30	**3.58**	0.00	P	446.33	HPS4	22cen-q12.3
221540_x_at	1.00	0.97	P	196.90	**3.57**	0.00	P	694.93	GTF2H2	5q12.2-q13.3
210976_s_at	1.00	0.98	P	351.80	**3.57**	0.00	P	1240.17	PFKM	12q13.3
218501_at	1.00	0.98	P	193.70	**3.57**	0.00	P	678.30	ARHGEF3	3p21-p13
201505_at	1.00	0.97	P	194.40	**3.56**	0.01	P	684.73	LAMB1	7q22
212826_s_at	1.00	0.96	P	1007.53	**3.56**	0.00	P	3539.17	SLC25A6	p22.32 and yp
210837_s_at	1.00	0.96	P,M,A	11.73	**3.56**	0.01	P	42.10	PDE4D	5q12
236249_at	0.99	0.92	P	57.30	**3.55**	0.01	P	202.03	IKIP	12q23.1
209147_s_at	0.99	0.92	P,A	87.07	**3.55**	0.00	P	306.03	PPAP2A	5q11
227615_at	1.00	0.95	P	88.33	**3.55**	0.00	P	308.07	TFIP11	22q12.1
227295_at	0.99	0.93	P	59.03	**3.54**	0.00	P	206.53	IKIP	12q23.1
243904_at	0.99	0.95	P	47.83	**3.54**	0.00	P	166.93		
243816_at	0.99	0.95	P	65.17	**3.54**	0.00	P	227.57		
221815_at	0.98	0.91	P,A	63.43	**3.54**	0.01	P	224.97	ABHD2	15q26.1
221571_at	1.00	0.98	P,M,A	95.90	**3.54**	0.00	P	334.70	TRAF3	14q32.33
218056_at	1.00	0.97	P	348.00	**3.53**	0.00	P	1213.70	BFAR	16p13.13
200742_s_at	0.99	0.91	P	194.37	**3.53**	0.01	P	687.47	CLN2	11p15
213906_at	0.97	0.88	P	112.93	**3.53**	0.00	P	394.73	MYBL1	8q22
219952_s_at	1.00	0.98	A	172.43	**3.53**	0.00	P	601.83	MCOLN1	9p13.3-p13.2
225485_at	1.00	0.97	P	242.57	**3.52**	0.00	P	843.73	TSGA14	7q32
209737_at	1.00	0.97	P,A	20.03	**3.52**	0.01	P	69.97	AIP1	7q21
228366_at	0.98	0.91	A	70.53	**3.52**	0.00	P	243.83	PPA2	4q25
1555468_at	1.00	0.97	P	51.07	**3.52**	0.01	P	178.33	NRP2	2q33.3
225810_at	1.00	0.99	P	94.40	**3.52**	0.00	P	327.43	FLJ20313	15q13.1
220444_at	0.99	0.92	A	29.10	**3.52**	0.01	P	101.83	ZNF557	19p13.3
228702_at	1.00	0.97	P,M,A	88.20	**3.52**	0.00	P	305.17	FLJ43663	7q32.3
243006_at	0.98	0.91	P,A	12.43	**3.52**	0.02	P	44.30		
204288_s_at	0.98	0.89	A	68.23	**3.52**	0.00	P	236.80	ARGBP2	4q35.1
203827_at	0.99	0.94	P	215.77	**3.52**	0.00	P	748.90	FLJ10055	17q24.3
1554887_at	0.99	0.94	P,A	23.17	**3.52**	0.01	P	81.53		
243740_at	0.99	0.93	A	28.00	**3.51**	0.00	P	97.17		
203343_at	1.00	0.99	P	394.70	**3.51**	0.00	P	1363.20	UGDH	4p15.1
203631_s_at	0.97	0.87	A	45.23	**3.51**	0.00	P	157.70	GPRC5B	16p12
231840_x_at	1.00	0.99	P	133.67	**3.51**	0.00	P	461.77	LOC90624	5q31.1
225851_at	0.99	0.92	P,A	211.03	**3.51**	0.00	P	729.80	FNTB	14q23-q24
202069_s_at	0.98	0.90	P	478.83	**3.50**	0.00	P	1660.53	IDH3A	5q25.1-q25.2
215485_s_at	0.98	0.89	A	68.83	**3.50**	0.00	P	238.93	ICAM1	9p13.3-p13.2
235204_at	1.00	0.96	P	59.23	**3.50**	0.00	P	204.17	ENTPD7	
226030_at	1.00	0.97	P	82.80	**3.49**	0.00	P	284.70	ACADSB	10q25-q26
224860_at	1.00	0.97	P,A	31.27	**3.49**	0.01	P	108.03	C9orf123	9p24.1
200770_s_at	1.00	0.97	P,M	166.90	**3.49**	0.00	P	574.40	LAMC1	1q31
218121_at	0.98	0.89	A	53.47	**3.49**	0.01	P	186.77	HMOX2	16p13.3
225420_at	0.97	0.88	P	117.40	**3.49**	0.00	P	404.70	GPAM	10q25.3
239735_at	1.00	0.96	P	77.13	**3.49**	0.02	P	272.20		
229833_at	1.00	0.97	A	84.83	**3.48**	0.01	P	295.70		
226955_at	0.99	0.92	P	121.13	**3.48**	0.00	P	416.40	FLJ36748	5q33.1
201525_at	1.00	0.97	A	126.53	**3.48**	0.00	P	433.63	APOD	3q26.2-qter
238619_at	0.96	0.86	P	134.87	**3.48**	0.01	P	459.60		
226684_at	1.00	0.96	P	84.27	**3.48**	0.00	P	288.17	C14orf103	14q32.31
1556744_a_at	0.98	0.90	P	42.57	**3.47**	0.00	P	145.20		
244106_at	0.99	0.95	A	16.90	**3.47**	0.00	P	57.73		
214508_x_at	0.99	0.94	A	51.10	**3.47**	0.00	P	174.20	CREM	10p11.21
235296_at	1.00	0.97	P	124.23	**3.47**	0.00	P	423.83	EIF5A2	3q26.2
233115_at	0.97	0.87	P	32.97	**3.47**	0.00	P	113.17		
242149_at	0.97	0.87	P	153.83	**3.47**	0.00	P	528.60		
230296_at	1.00	0.97	P	206.90	**3.46**	0.00	P	704.13	LOC146174	16p12.3
227197_at	1.00	0.97	P	122.07	**3.46**	0.00	P	416.53	SGEF	3q25.2
205356_at	1.00	0.95	P	175.83	**3.46**	0.00	P	597.10	USP13	3q26.2-q26.3
225910_at	1.00	0.98	P	157.87	**3.46**	0.00	P	538.80	LOC284019	17q24.3
204511_at	0.98	0.91	A	70.40	**3.46**	0.01	P	241.27	FARP2	2q37.3
1556064_at	0.99	0.94	M,A	42.03	**3.46**	0.00	P	143.73	LOC284926	22q13.31
203642_s_at	1.00	0.97	P	152.17	**3.46**	0.00	P	516.53	COBLL1	2q24.3
229883_at	1.00	0.99	P,M	60.03	**3.45**	0.00	P	205.90	GRIN2D	19q13.1-qter
1564906_at	0.96	0.86	A	25.40	**3.45**	0.00	P,M	87.43	MATR3	5q31.3
213140_s_at	1.00	0.96	P	360.00	**3.45**	0.00	P	1222.53	SS18L1	20q13.3
1558028_x_at	0.99	0.93	P,M	60.03	**3.44**	0.00	P	204.03		
224867_at	1.00	0.99	P	955.27	**3.44**	0.00	P	3231.37	CG012	13q12-q13
226365_at	0.99	0.95	A	58.00	**3.43**	0.00	P	196.63		8
1554424_at	0.99	0.92	P	24.67	**3.43**	0.01	P	83.70	FIP1L1	4q12
218568_at	0.99	0.93	P	197.23	**3.43**	0.00	P	667.70	FLJ10842	7q34
202679_at	1.00	0.96	P	145.53	**3.43**	0.00	P	490.50	NPC1	18q11-q12
222132_s_at	1.00	0.99	P	279.90	**3.43**	0.00	P	948.27	FLJ10842	7q34
203544_s_at	1.00	0.98	P	328.17	**3.42**	0.00	P	1105.07	STAM	10p14-p13
212450_at	1.00	0.96	P	165.00	**3.42**	0.00	P	556.20	KIAA0256	15q15.3
217635_s_at	0.96	0.86	P,M,A	47.43	**3.42**	0.01	P	162.70	POLG	15q25
210447_at	0.97	0.87	P,M	27.33	**3.42**	0.00	P	92.60	GLUD1	10q23.3
219430_at	0.97	0.88	A	65.30	**3.42**	0.01	P,M,A	225.83	C11ORF4	11cen-q22.3

225949_at	0.97	0.89	P,A	145.63	**3.42**	0.00	P	494.10	LOC340371	8q24.3
203799_at	0.99	0.95	P	68.33	**3.42**	0.00	P	230.20	DCL-1	2q24.2
226077_at	1.00	0.96	P	527.60	**3.42**	0.00	P	1776.27	FLJ31951	5q33.3
229113_s_at	1.00	0.95	P,A	161.33	**3.42**	0.00	P	540.80	FLJ31031	1p36.32
243586_at	0.96	0.86	P,A	12.87	**3.41**	0.02	P	44.40		
203159_at	1.00	0.97	P	134.73	**3.41**	0.00	P	451.57	GLS	2q32-q34
235964_x_at	0.99	0.94	P	52.93	**3.41**	0.00	P	177.07	FLJ46365	8q11.21
216483_s_at	1.00	0.96	P	284.30	**3.41**	0.00	P	956.03	C19orf10	19p13.3
221187_s_at	0.95	0.85	A	30.83	**3.41**	0.02	P	106.30	FLJ22688	19q13.33
227121_at	0.99	0.92	A	41.77	**3.41**	0.00	P	139.47		
217309_s_at	0.98	0.91	P,A	98.07	**3.41**	0.00	P	331.43	DSCR3	21q22.2
235389_at	0.99	0.92	P,A	34.83	**3.41**	0.00	P	116.43	C20orf104	q11.22-q11.23
212563_at	1.00	0.99	P	219.77	**3.40**	0.00	P	738.27	BOP1	8q24.3
202637_s_at	0.99	0.94	P	285.77	**3.40**	0.00	P	962.63	ICAM1	9p13.3-p13.2
221675_s_at	1.00	0.99	P	425.83	**3.40**	0.00	P	1424.70	CHPT1	12q
212829_at	0.98	0.91	P	170.27	**3.40**	0.01	P	569.03		
226782_at	1.00	1.00	P	83.37	**3.39**	0.00	P	278.47	SLC25A30	13q14.11
219068_x_at	0.99	0.92	P	160.93	**3.39**	0.00	P	534.73	ATAD3A	1p36.33
202464_s_at	1.00	0.98	P	177.63	**3.39**	0.00	P	592.47	PFKFB3	10p14-p15
215329_s_at	0.96	0.86	A	81.27	**3.39**	0.01	P,A	273.20	CDC2L1	1p36
221985_at	1.00	0.99	P	64.17	**3.39**	0.00	P	214.07	DRE1	3q27.3
202297_s_at	1.00	0.96	P	527.23	**3.39**	0.00	P	1759.73	RER1	1pter-q24
211414_at	1.00	0.97	A	15.43	**3.39**	0.01	P	51.60	GLS	2q32-q34
212233_at	1.00	0.98	P	48.00	**3.39**	0.00	P	160.07	MAP1B	5q13
218549_s_at	1.00	0.97	P	247.07	**3.38**	0.00	P	821.77	CGI-90	8q21.2
226902_at	1.00	0.99	P	97.97	**3.38**	0.00	P	326.07	USP13	3q26.2-q26.3
201396_s_at	1.00	0.97	P	135.90	**3.38**	0.00	P	451.07	SGTA	19p13
242494_at	1.00	0.98	M,A	21.00	**3.38**	0.01	P	70.50		
225955_at	1.00	0.98	P	135.17	**3.38**	0.00	P	449.93	LOC284207	17q25.3
224744_at	0.99	0.92	P	125.60	**3.38**	0.00	P	418.73	FLJ20421	8q11.23
200745_s_at	0.99	0.93	P	726.90	**3.37**	0.00	P	2423.47	GNB1	1p36.33
212739_s_at	1.00	0.98	P	400.13	**3.37**	0.00	P	1327.20	NME4	16p13.3
239960_x_at	1.00	0.95	P	51.43	**3.37**	0.00	P	171.17	LOC90624	5q31.1
225116_at	1.00	1.00	P	222.60	**3.37**	0.00	P	737.53	HIPK2	7q32-q34
235532_at	0.98	0.91	A	43.20	**3.37**	0.00	P	143.20		
226980_at	0.99	0.94	P	317.73	**3.37**	0.00	P	1054.73	XTP1	5q12.1
208682_s_at	1.00	0.98	P	239.70	**3.37**	0.00	P	793.77	MAGED2	xp11.2
227593_at	1.00	0.98	P	77.33	**3.37**	0.00	P	256.87		
225564_at	0.99	0.92	P	122.67	**3.37**	0.01	P	409.30	SPATA13	13q12.13
225545_at	0.99	0.92	P,A	247.47	**3.36**	0.00	P	814.53	EEF2K	16p12.3
229512_at	1.00	0.99	P,M	75.73	**3.36**	0.01	P	252.40		
223335_at	1.00	0.97	P	248.80	**3.36**	0.00	P	820.80	LOC51249	1p34.1
218202_x_at	0.98	0.91	P	276.67	**3.36**	0.01	P	930.00	MRPL44	2q36.3
219234_x_at	0.97	0.88	P,A	22.40	**3.35**	0.00	P	74.53	SCRN3	2q31.1
225677_at	1.00	0.98	P	215.03	**3.35**	0.00	P	710.03	BCAP29	7q22-q31
224913_s_at	0.99	0.95	P	669.70	**3.35**	0.00	P	2211.97	TIMM50	19q13.2
200764_s_at	0.99	0.93	P	248.03	**3.35**	0.00	P	819.17	CTNNA1	5q31
210943_s_at	1.00	0.98	P	175.37	**3.35**	0.01	P	578.70	CHS1	1q42.1-q42.2
238465_at	1.00	0.96	P	315.90	**3.34**	0.00	P	1042.97	MGC33648	5q11.2
210685_s_at	0.96	0.85	P	80.27	**3.34**	0.00	P	265.77	UBE4B	1p36.3
202156_s_at	1.00	0.98	P,A	148.87	**3.34**	0.01	P	491.87	CUGBP2	10p13
210290_at	0.99	0.94	P	37.60	**3.34**	0.01	P	124.90	ZNF174	16p13.3
230768_at	1.00	0.96	P,A	32.20	**3.34**	0.00	P	106.07		
209233_at	0.99	0.92	P	412.27	**3.33**	0.00	P	1360.00	C2F	12p13
220038_at	0.99	0.94	P	248.87	**3.33**	0.00	P	819.53	SGKL	q12.3-8q13.1
223539_s_at	1.00	0.98	P,M,A	122.50	**3.33**	0.00	P	402.00	SERF1A	5q12.2-q13.3
219915_s_at	0.99	0.95	A	40.13	**3.33**	0.00	P	131.30	SLC16A10	6q21-q22
202009_at	1.00	0.98	P	102.80	**3.33**	0.00	P	336.80	PTK9L	3p21.1
1552663_a_at	0.99	0.93	A	103.53	**3.32**	0.00	P	338.43	ELKS	12p13.3
202797_at	1.00	0.97	P	226.17	**3.32**	0.00	P	740.03	SACM1L	3p21.3
204544_at	0.98	0.90	P	348.40	**3.32**	0.01	P	1153.67	HPS5	11p14
201859_at	0.97	0.89	P,A	32.43	**3.32**	0.00	P	106.07	PRG1	10q22.1
1554283_at	1.00	0.96	P,A	27.97	**3.32**	0.01	P	92.00	CCRN4L	4q28.3
225093_at	0.99	0.94	P	137.70	**3.32**	0.01	P	453.60	UTRN	6q24
215599_at	0.99	0.94	P	108.70	**3.31**	0.03	P	366.13	SMA4	5q13
212810_s_at	0.99	0.94	P	180.43	**3.31**	0.01	P	587.67	SLC1A4	2p15-p13
225447_at	1.00	0.96	P	567.73	**3.31**	0.00	P	1852.67	GPD2	2q24.1
222664_at	0.99	0.95	P	97.37	**3.31**	0.00	P	317.13	KCTD15	19q13.12
227699_at	0.99	0.95	P,A	117.07	**3.30**	0.00	P	381.27	FLJ25436	14q23.1
1564175_at	0.98	0.91	A	16.70	**3.29**	0.00	P	54.27		
202944_at	0.98	0.90	P,A	86.50	**3.29**	0.00	P	281.97	NAGA	22q11
209208_at	0.99	0.94	A	225.23	**3.29**	0.00	P	734.50	MPDU1	17p13.1-p12
202472_at	1.00	0.97	A	54.27	**3.29**	0.00	P	176.10	MPI	15q22-qter
1559221_at	0.98	0.91	A	13.07	**3.29**	0.00	P,M	42.13		
226440_at	0.99	0.94	P	240.00	**3.29**	0.00	P	778.43	DUSP22	6p25.3
226854_at	1.00	0.97	A	35.13	**3.29**	0.00	P,M,A	114.70	GTPBP5	20q13.33
205308_at	1.00	0.99	A	38.37	**3.29**	0.00	P	124.40	CGI-62	8q21.11
1554442_at	0.99	0.94	A	347.33	**3.29**	0.00	P,M	1123.73	VMD2	11q13
236634_at	1.00	0.95	A	15.57	**3.29**	0.02	P	51.77	FLJ25402	8p22
214488_at	0.97	0.89	A	9.50	**3.29**	0.01	P	31.30	RAP2B	3q25.2
239780_at	1.00	0.97	P,A	17.20	**3.28**	0.02	P	56.73		
200868_s_at	0.99	0.94	P	341.00	**3.28**	0.00	P	1105.30	ZNF313	20q13.13
241838_at	1.00	0.95	P	58.63	**3.27**	0.00	P	188.43		
241435_at	0.99	0.93	P	32.57	**3.27**	0.01	P	105.93		
202914_s_at	1.00	0.96	P,A	59.07	**3.27**	0.00	P	191.60	ARHGEF11	1q21
223518_at	1.00	0.96	P	204.77	**3.27**	0.00	P	658.23	DFFA	1p36.3-p36.2
235554_x_at	0.99	0.92	P	70.17	**3.27**	0.00	P	226.50	MGC29898	4p15.32
215073_s_at	0.98	0.90	P	58.30	**3.27**	0.00	P	188.30	NR2F2	15q26
202089_s_at	0.98	0.90	P	360.57	**3.26**	0.00	P	1165.00	SLC39A6	18q12.2
201226_at	1.00	0.97	P	522.50	**3.26**	0.00	P	1676.27	NDUFB8	0q23.2-q23.33
219325_s_at	0.96	0.85	A	7.53	**3.26**	0.03	P	25.47	ELAC1	18q21
219131_at	0.99	0.93	P,A	69.20	**3.26**	0.00	P	221.73	TERE1	1pter
229111_at	1.00	0.96	A	37.33	**3.26**	0.00	P	119.53	MASP2	1p36.3-p36.2
211613_s_at	1.00	0.95	A	32.70	**3.26**	0.00	P	105.50	GPD2	2q24.1
226775_at	0.99	0.95	P	60.33	**3.26**	0.00	P	193.67	e(y)2	8q23.2
241804_at	1.00	0.96	P	21.37	**3.26**	0.01	P	69.33		
201719_s_at	1.00	0.97	P,M	88.90	**3.25**	0.00	P	285.17	EPB41L2	6q23
244181_at	0.97	0.88	P,M	14.90	**3.25**	0.02	P	48.70		
228007_at	0.99	0.94	P	86.13	**3.25**	0.01	P	276.07	C6orf204	6q22
208319_s_at	0.99	0.94	P	808.43	**3.25**	0.00	P	2592.53	RBM3	xp11.2
1553299_at	0.99	0.94	P,A	9.87	**3.24**	0.00	P	31.47		
200615_s_at	1.00	0.98	A	119.83	**3.24**	0.00	P	383.60	AP2B1	17q11.2-q12
203410_at	1.00	0.96	A	195.97	**3.24**	0.00	P	627.17	AP3M2	8p11.2
238427_at	0.99	0.93	M,A	29.80	**3.24**	0.00	P	96.13	GRPEL2	5q33.1
1554670_at	0.99	0.93	P	97.33	**3.24**	0.01	P	314.20	GGA1	22q13.31
217790_s_at	1.00	0.99	P	157.20	**3.23**	0.00	P	500.73	SSR3	3q25.31
204209_at	1.00	0.97	P,A	103.80	**3.23**	0.00	P	331.83		
221042_s_at	0.99	0.94	P	89.60	**3.23**	0.00	P	285.67	CLMN	14q32.2
234431_at	0.99	0.93	A	42.53	**3.23**	0.01	P,M	136.07	GSN	9q33
223785_at	1.00	0.98	P	164.13	**3.23**	0.00	P	523.27	FLJ10719	15q25-q26
210844_x_at	1.00	0.98	P	432.07	**3.22**	0.00	P	1369.13	CTNNA1	5q31
238811_at	0.97	0.88	A	15.73	**3.22**	0.00	P	50.03	ATP11B	3q27
216683_at	0.98	0.90	P,A	20.00	**3.21**	0.00	P	63.13		
41644_at	0.98	0.90	P	287.87	**3.21**	0.00	P	904.80	SASH1	6q24.3
1558305_at	0.97	0.87	P,A	27.73	**3.21**	0.02	P	89.60	TNRC15	2q37.1
225479_at	1.00	0.96	P	663.67	**3.21**	0.00	P	2100.00	LOC116064	3q13.33
226795_at	0.99	0.92	A	53.43	**3.21**	0.01	P	169.33	CHDC1	13q14.11
201278_at	1.00	0.97	P	74.53	**3.21**	0.00	P	235.43	DAB2	5p13
227131_at	0.99	0.94	P	89.43	**3.21**	0.00	P	282.17	MAP3K3	17q24.2
239108_at	0.98	0.90	P	73.80	**3.20**	0.01	P	231.83	MLSTD1	12p11.23
212142_at	0.98	0.91	P	80.87	**3.20**	0.00	P	257.23	MCM4	8q11.2
1567081_x_at	0.99	0.94	P	147.73	**3.20**	0.01	P	472.77	CLN6	15q22.31
223249_at	1.00	0.97	P	185.60	**3.20**	0.00	P	585.60	CLDN12	7q21
224462_s_at	0.99	0.94	P	195.43	**3.20**	0.00	P	614.50	CHCHD6	3q21.3
223159_s_at	0.99	0.94	P	69.77	**3.20**	0.00	P	219.53	NEK6	q33.3-q34.11
200980_s_at	1.00	0.98	P	901.27	**3.19**	0.00	P	2846.13	PDHA1	xp22.2-p22.1
223964_x_at	1.00	0.96	P	126.80	**3.19**	0.00	P	399.50	ZSIG11	3p21.31
223854_at	0.99	0.92	P	73.43	**3.19**	0.00	P	231.43	PCDHB10	5q31
228757_at	0.95	0.83	A	46.20	**3.19**	0.02	P	148.23	SCDR10	19p13.3
238908_at	0.98	0.89	P	49.27	**3.19**	0.01	P	154.40	CALU	7q32
241696_at	0.98	0.91	P,M	25.27	**3.19**	0.02	P	81.30	C9orf39	9p22.2-p22.1
63009_at	1.00	0.97	P	187.57	**3.19**	0.00	P	589.37	FLJ10539	3p14.2
201563_at	1.00	0.97	P	364.90	**3.19**	0.00	P	1144.87	SORD	15q15.3
238646_at	0.99	0.95	A	15.17	**3.18**	0.01	P	47.77		

207147_at	0.99	0.94	A	77.00	**3.18**	0.00	P	241.70	DLX2	2q32
223927_at	0.97	0.88	A	13.87	**3.18**	0.01	P,A	43.87	PCDHB9	5q31
1556361_s_at	1.00	0.98	P,M	21.00	**3.18**	0.00	P	65.73	(FZP566D1:	1p32.3-p31.3
208624_s_at	0.98	0.91	P	224.63	**3.18**	0.00	P	706.37	EIF4G1	3q27-qter
212085_at	1.00	0.95	P	1164.13	**3.18**	0.00	P	3650.63	SLC25A6	p22.32 and yp
1561017_at	0.99	0.94	P	38.40	**3.17**	0.00	P	120.03		
218258_at	1.00	0.99	P	707.70	**3.17**	0.00	P	2207.80	POLR1D	13q12.2
227563_at	1.00	0.95	P	46.10	**3.17**	0.01	P	144.83	MGC42630	9q21.12
229826_at	1.00	0.97	P	65.97	**3.17**	0.01	P	207.03		
226362_at	0.98	0.91	A	56.70	**3.17**	0.00	P,M,A	177.53		5q13.1
208361_s_at	1.00	0.95	M,A	67.37	**3.17**	0.00	P	210.30	POLR3D	8q21
242188_at	0.99	0.95	P	34.17	**3.16**	0.01	P	107.67		
1557197_a_at	0.98	0.90	P	43.83	**3.16**	0.04	P	141.30	LGALS3	14q21-q22
235126_at	0.99	0.93	A	53.80	**3.16**	0.00	P	167.33		
221056_x_at	0.99	0.91	A	77.27	**3.16**	0.00	P	240.87	EPS15L1	19p13.12
1553322_s_at	0.97	0.89	P,A	20.43	**3.16**	0.01	P	65.07	TEAD1	11p15.4
1555388_s_at	0.99	0.94	M,A	24.00	**3.16**	0.00	P	74.30	SNX25	4q35.1
239405_at	0.98	0.90	A	55.57	**3.16**	0.00	P	173.80		
227283_at	1.00	0.96	A	43.40	**3.16**	0.01	P,A	138.20		
218298_s_at	1.00	0.98	P,M,A	63.60	**3.15**	0.00	P	197.70	C14orf159	14q32.12
201392_s_at	0.99	0.94	P	204.07	**3.15**	0.00	P	631.00	IGF2R	6q26
219099_at	1.00	0.95	P	414.87	**3.15**	0.00	P	1288.53	C12orf5	12p13.3
222701_s_at	1.00	0.98	P	129.93	**3.14**	0.00	P	401.47	CHCHD7	8q11.23
242260_at	1.00	0.96	P	303.10	**3.14**	0.00	P	939.27	MATR3	5q31.3
213900_at	0.98	0.89	P,M,A	64.00	**3.14**	0.00	P	198.70	C9orf61	9q13-q21
223304_at	1.00	0.96	P	221.50	**3.14**	0.00	P	686.47	SLC37A3	7q34
212345_s_at	1.00	0.99	P	487.57	**3.14**	0.00	P	1507.83	CREB3L2	7q34
210605_s_at	0.99	0.94	M,A	153.23	**3.14**	0.02	P	483.10	MFGE8	15q25
244487_at	0.98	0.90	P	37.07	**3.14**	0.00	P	114.53		
242652_at	1.00	0.99	A	32.30	**3.14**	0.01	P	100.40		
226460_at	0.99	0.94	P	190.63	**3.14**	0.00	P	588.67	KIAA1450	4q32.1
242390_at	0.96	0.86	P,A	47.80	**3.14**	0.02	P	149.07		
1554835_a_at	0.99	0.93	A	27.37	**3.13**	0.00	P,M	84.97	B3GNT5	3q28
217859_s_at	0.97	0.89	P	34.83	**3.13**	0.00	P	108.27	SLC39A9	14q24.1
235198_at	1.00	0.96	P	259.50	**3.13**	0.00	P	800.97		
223707_at	1.00	0.96	P	79.40	**3.13**	0.00	P	244.40	MGC10850	11
243529_at	0.99	0.92	P	95.83	**3.13**	0.00	P	298.67	MetRS	2q33.1
203432_at	0.99	0.92	P	515.07	**3.13**	0.00	P	1593.97	TMPO	12q22
201040_at	1.00	0.99	P,A	139.27	**3.13**	0.00	P	431.47	GNAI2	3p21
208358_s_at	0.98	0.89	P,A	60.37	**3.13**	0.00	P	186.97	UGT8	4q26
229483_at	0.99	0.95	P	41.30	**3.13**	0.01	P	128.13		
206256_at	1.00	0.95	P,A	52.90	**3.12**	0.00	P	162.43	CPN1	10q24.31
202566_s_at	0.98	0.91	P,A	97.17	**3.12**	0.00	P	300.37	SVIL	10p11.2
232197_x_at	0.99	0.94	P	57.93	**3.12**	0.01	P	179.33	ARSB	5p11-q13
213627_at	0.98	0.91	A	167.37	**3.12**	0.00	P	515.17	MAGED2	xp11.2
221834_at	1.00	0.97	P	108.97	**3.12**	0.01	P	335.27	LONP	16q12.1
1554310_a_at	0.98	0.89	P,A	19.83	**3.12**	0.00	P	60.67	EIF4G3	1p36.12
218640_s_at	0.99	0.92	P	278.10	**3.11**	0.00	P	854.40	PLEKHF2	8q22.1
218156_s_at	0.99	0.92	P	512.70	**3.11**	0.00	P	1575.03	FLJ10534	17p13.3
225674_at	0.97	0.89	P	417.63	**3.11**	0.00	P	1285.30	BCAP29	7q22-q31
201666_at	1.00	0.98	P	530.40	**3.11**	0.01	P	1642.03	TIMP1	p11.3-p11.23
238227_at	0.98	0.90	P,A	46.47	**3.11**	0.00	P	142.27		
203005_at	0.98	0.90	P	180.17	**3.11**	0.00	P	551.03	LTBR	12p13
225329_at	0.99	0.93	P,A	111.60	**3.11**	0.01	P	341.83	LOC348262	17
207624_s_at	1.00	0.97	P,M	36.97	**3.10**	0.00	P	112.93	RPGR	xp11.4
227161_at	0.99	0.92	P	96.43	**3.10**	0.00	P	295.97	C7orf3	7q36.3
225028_at	0.99	0.94	P	33.00	**3.10**	0.00	P	101.30		Xp11.22
208185_x_at	0.99	0.94	A	11.53	**3.10**	0.02	P,A	35.57		
240759_at	0.99	0.95	P,A	18.20	**3.10**	0.02	P	56.30		
206473_at	0.99	0.92	A	48.77	**3.10**	0.00	P	150.47	MBTPS2	(p22.1-p22.2
235144_at	0.99	0.94	P	371.57	**3.10**	0.00	P	1138.93	FLJ31614	9q21.33
201393_s_at	0.99	0.93	P	420.33	**3.09**	0.00	P	1285.00	IGF2R	6q26
240757_at	0.98	0.90	P	9.60	**3.09**	0.01	P	29.60	CLASP1	2q14.2-q14.3
212719_at	1.00	0.96	P	108.57	**3.09**	0.00	P	330.90	PLEKHE1	18q21.33
243287_s_at	1.00	0.97	P,M	55.70	**3.09**	0.00	P	169.83		
214552_s_at	0.97	0.88	P	50.77	**3.09**	0.00	P	155.37	RABEP1	17p13.3
215286_s_at	1.00	0.97	M,A	45.63	**3.09**	0.01	P	140.67	PHTF2	7q11.23-q21
214850_at	1.00	0.98	P,A	35.97	**3.09**	0.00	P	109.47	SMA5	5q13
221536_s_at	1.00	1.00	P	201.50	**3.09**	0.00	P	611.97	FLJ11301	3q29
238654_at	0.99	0.93	A	62.13	**3.08**	0.00	P	189.57	LOC147645	19q13.41
207543_s_at	0.98	0.90	P,A	180.20	**3.08**	0.00	P	543.93	P4HA1	0q21.3-q23.1
226974_at	1.00	0.98	P	62.03	**3.08**	0.00	P	188.57		
214681_at	0.99	0.93	A	50.07	**3.08**	0.02	P	154.30	GK	xp21.3
225917_at	1.00	0.97	P	52.37	**3.08**	0.02	P	160.90	ATF7IP	12p13.2
200696_s_at	0.99	0.92	P	711.77	**3.08**	0.00	P	2165.27	GSN	9q33
226776_at	0.98	0.90	P,A	20.77	**3.08**	0.00	P	62.77	e(y)2	8q23.2
243487_at	0.99	0.92	P,A	39.83	**3.08**	0.01	P	122.23	AF5Q31	5q31
1556588_at	1.00	0.98	P	116.87	**3.08**	0.00	P	354.47	LOC283687	15q24.3
235967_at	0.99	0.92	A	38.07	**3.08**	0.00	P	115.70		
224702_at	1.00	0.95	P	472.83	**3.08**	0.00	P	1437.00	MGC23909	5q14.2
231234_at	0.99	0.93	P	32.97	**3.08**	0.01	P	100.07	CTSC	1q14.1-q14.3
200903_s_at	0.99	0.94	P	992.70	**3.08**	0.00	P	3022.53	AHCY	20cen-q13.1
227871_at	1.00	0.97	P	226.47	**3.07**	0.00	P	685.97	FLJ38564	xq21.2
233518_at	0.98	0.89	P	30.97	**3.07**	0.00	P	93.73		
215483_at	1.00	0.95	P	31.70	**3.07**	0.02	P	97.07	AKAP9	7q21-q22
207630_s_at	1.00	0.98	P	94.20	**3.07**	0.00	P	284.90	CREM	10p11.21
218741_at	0.99	0.93	P	284.70	**3.07**	0.00	P	856.00	C22orf18	22q13.31
244523_at	1.00	0.97	M,A	48.23	**3.07**	0.00	P	145.97	MMD	17q
212250_at	0.99	0.94	P	638.87	**3.07**	0.00	P	1934.00	LYRIC	8q22.1
205281_s_at	1.00	0.96	P	139.37	**3.07**	0.00	P	421.37	PIGA	xp22.1
206566_at	0.99	0.92	M,A	25.27	**3.07**	0.02	P	78.80	SLC7A1	13q12-q14
244008_at	0.97	0.89	A	29.13	**3.07**	0.00	P,M	87.67		
201096_s_at	0.99	0.92	P	912.93	**3.07**	0.00	P	2766.30	ARF4	3p21.2-p21.1
210980_s_at	0.98	0.91	P	737.67	**3.07**	0.00	P	2235.07	ASAH1	8p22-p21.3
211960_s_at	1.00	0.95	P	855.27	**3.07**	0.00	P	2584.87	RAB7	3q21.3
243361_at	0.97	0.88	A	31.40	**3.06**	0.02	P,M	97.10		
1563614_at	0.99	0.93	A	14.30	**3.06**	0.00	P	43.10	MTBP	8q24.12
228563_at	0.98	0.90	A	90.03	**3.06**	0.00	P	272.33	GJA7	17q21.31
219083_at	1.00	0.97	M,A	333.27	**3.06**	0.00	P	1004.07	FLJ10539	3p14.2
201009_s_at	1.00	0.99	A	81.33	**3.06**	0.00	P,A	244.27	TXNIP	1q21.2
237664_at	0.98	0.90	P	25.83	**3.05**	0.00	P	77.47		
204330_s_at	0.98	0.91	P	41.60	**3.05**	0.01	P	126.13	MRPS12	9q13.1-q13.2
213018_at	0.98	0.91	P	87.40	**3.05**	0.00	P	263.53	ODAG	7q21-q22
217749_at	1.00	0.95	P	212.60	**3.05**	0.00	P	638.53	COPG	3q21.3
226837_at	1.00	0.95	P	173.30	**3.05**	0.00	P	521.47	SPRED1	15q13.3
242577_at	0.99	0.92	P,A	11.47	**3.05**	0.00	P	34.67		
238069_at	1.00	0.96	P,M	56.07	**3.04**	0.00	P	168.47	USP45	6q16.3
230071_at	0.99	0.94	A	33.57	**3.04**	0.00	P	100.97	FLJ10849	4q21.22
224733_at	1.00	0.99	P	102.53	**3.04**	0.00	P	306.50	CKLFSF3	16q22.1
215308_at	0.99	0.94	A	24.83	**3.04**	0.00	P	73.90	G22P1	2q13.2-q13.31
211373_s_at	0.98	0.91	A	52.17	**3.04**	0.00	P	155.33	PSEN2	1q31-q42
203011_at	0.99	0.93	P	420.80	**3.04**	0.00	P	1260.83	IMPA1	q21.13-q21.3
1562955_at	0.99	0.92	P,A	32.43	**3.03**	0.00	P	97.27		
1559060_a_at	0.99	0.92	P,A	27.47	**3.03**	0.01	P	82.30	RAPGEF6	5q31.1
229354_at	1.00	0.96	P	56.17	**3.03**	0.00	P	167.60	AHRR	5p15.3
217043_s_at	0.99	0.94	P	225.20	**3.03**	0.00	P	672.23	MFN1	3q27.1
1552695_a_at	0.99	0.92	A	43.47	**3.03**	0.00	P,M	129.60	SLC2A13	12q12
236982_at	0.99	0.93	A	27.00	**3.02**	0.00	P,M	80.17		
209236_at	0.98	0.91	A	94.53	**3.02**	0.00	P	282.53	SLC23A2	20p13
1555910_at	0.99	0.93	P	88.53	**3.02**	0.00	P	263.73	PTCD2	5q13.2
236953_s_at	0.99	0.93	P	55.30	**3.02**	0.01	P	166.40	LOC283506	13q13.3
203182_s_at	0.99	0.94	P	313.90	**3.02**	0.00	P	935.70	SRPK2	7q22-q31.1
221786_at	0.99	0.94	P	160.10	**3.02**	0.00	P	476.23	PHF10	6q27
1559117_at	0.99	0.95	A	53.43	**3.01**	0.00	P,A	158.40		
202538_s_at	0.99	0.95	P,A	307.80	**3.01**	0.00	P	914.50	(FZP564O1	3p12.1
227560_at	0.98	0.90	P	146.30	**3.01**	0.00	P	435.60	SFXN2	10q24.33
238218_at	0.98	0.89	A	39.27	**3.01**	0.00	P	116.63	DDX43	6q12-q13
201439_at	1.00	0.96	P	138.43	**3.01**	0.00	P	411.27	GBF1	10q24
235396_at	1.00	0.95	A	146.43	**3.01**	0.00	P	433.30	(FZp761P11	22q11.21
205586_x_at	0.98	0.91	P,A	53.97	**3.01**	0.00	P	160.37	VGF	7q22
1556224_a_at	1.00	0.97	A	11.73	**3.01**	0.01	P,A	35.03	LOC155060	7q36.1
210658_s_at	0.97	0.88	P	115.20	**3.01**	0.00	P	342.87	GGA2	16p12
235167_at	0.99	0.93	P,A	48.97	**3.01**	0.00	P	145.63		
206861_s_at	1.00	0.99	P	539.77	**3.01**	0.00	P	1596.40	CGGBP1	3p12-p11.1

221264_s_at	1.00	0.97	P	126.37	**3.00**	0.01	P	375.40	TARDBP	1p36.22
201028_s_at	0.99	0.93	P	307.73	**3.00**	0.00	P	912.43	CD99	>22.32; yp11.3
221911_at	1.00	0.98	P	83.40	**3.00**	0.00	P	246.40	LOC221810	7p21.3
225875_s_at	0.99	0.93	P	104.00	**3.00**	0.01	P	310.30	DJ462O23.2	p36.12-p35.1
215792_s_at	0.99	0.95	P	244.90	**3.00**	0.00	P	721.00	FLJ10737	1p36.23
221539_at	0.98	0.91	P	611.73	**3.00**	0.00	P	1815.47	EIF4EBP1	8p12
216933_x_at	1.00	0.96	P,A	28.53	**2.99**	0.01	P	85.17	APC	5q21-q22
227627_at	1.00	0.95	P	186.63	**2.99**	0.00	P	551.60	SGKL	q12.3-8q13.1
235295_at	0.99	0.92	P	138.37	**2.99**	0.00	P	408.57	PANX1	11q21
227237_x_at	0.98	0.90	P	144.07	**2.99**	0.00	P	421.70	ATAD3A	1p36.33
1569669_at	0.98	0.91	P,A	12.67	**2.99**	0.01	P	37.70	MGC21658	xp11.22
237116_at	0.97	0.88	P,A	18.10	**2.99**	0.01	P	53.97	PFN2	3q25.1-q25.2
212561_at	1.00	0.98	P	163.60	**2.99**	0.00	P	481.20	RAB6IP1	11p15.3
209883_at	0.99	0.93	P,A	48.57	**2.99**	0.00	P	143.10	C1orf17	1q25
212178_s_at	0.98	0.89	P	414.53	**2.99**	0.00	P	1223.93	POM121	7q11.23
225343_at	1.00	0.96	P	419.27	**2.98**	0.00	P	1227.93	LOC283578	14q24.3
200831_s_at	0.99	0.92	P,A	294.73	**2.98**	0.00	P	871.10	SCD	10q23-q24
241893_at	0.99	0.94	M,A	11.03	**2.98**	0.01	P	32.97		
204807_at	0.99	0.95	P	110.43	**2.97**	0.00	P	324.57	TMEM5	12q14.1
235213_at	0.99	0.91	A	29.53	**2.97**	0.02	P	87.33	ITPKB	1q42.13
204261_s_at	1.00	0.96	A	16.30	**2.97**	0.00	P	47.80	PSEN2	1q31-q42
222654_at	0.99	0.95	P	301.40	**2.97**	0.00	P	883.73	FLJ20421	8q11.23
222834_s_at	0.97	0.88	P	261.37	**2.97**	0.00	P	759.80	GNG12	1p31.2
219865_at	1.00	0.98	A	59.83	**2.97**	0.01	P	175.57	HSPC157	1p36.12
228076_s_at	0.99	0.92	P,A	34.07	**2.97**	0.02	P	103.43	NOL6	9p13.3
221563_at	0.99	0.92	P	89.07	**2.97**	0.00	P	259.57	DUSP10	1q41
200604_s_at	0.97	0.89	P	263.83	**2.97**	0.00	P	778.50	PRKAR1A	17q23-q24
203282_at	0.99	0.94	P	617.93	**2.97**	0.00	P	1810.13	GBE1	3p12.3
228717_at	0.98	0.90	P	47.40	**2.97**	0.01	P	139.93		
212904_at	0.99	0.93	P	597.83	**2.96**	0.00	P	1736.73	KIAA1185	1p36.32
223457_at	1.00	0.97	P	91.40	**2.96**	0.00	P	266.30	COPG2	7q32
209414_at	0.96	0.86	A	20.27	**2.96**	0.02	P	60.53	FZR1	19p13.3
202134_s_at	1.00	1.00	A	294.77	**2.96**	0.01	P	872.90	TAZ	3q23-q24
201541_s_at	0.99	0.93	P	235.67	**2.95**	0.00	P	683.33	ZNHIT1	7q22.1
221961_at	0.99	0.94	A	26.87	**2.95**	0.01	P	78.97	CLCN7	16p13
223282_at	1.00	0.96	M,A	60.40	**2.95**	0.00	P	174.90	SDCCAG33	18q22.3
201380_at	1.00	0.97	P	301.07	**2.95**	0.00	P	874.17	CRTAP	3p22
202519_at	1.00	0.95	P	159.47	**2.95**	0.01	P	463.43	MONDOA	12q21.31
202422_s_at	0.99	0.94	P	179.87	**2.95**	0.00	P	523.03	ACSL4	xq22.3-q23
223319_at	1.00	0.97	A	102.13	**2.95**	0.00	P	295.87	GPHN	4q23.3-q24.1
212835_at	0.99	0.94	P,A	105.70	**2.95**	0.00	P	307.50	KIAA0157	10q26.2
1565681_s_at	0.99	0.94	P,A	81.63	**2.95**	0.00	P	237.10	KIAA0934	10p15.3
215698_at	0.99	0.92	P	86.50	**2.95**	0.00	P	251.67	JARID1A	12p11
222402_at	0.99	0.94	P	168.63	**2.94**	0.00	P	490.17	C13orf12	13q12.3
202638_s_at	0.99	0.94	P	217.47	**2.94**	0.00	P	631.07	ICAM1	9p13.3-p13.2
236526_x_at	0.99	0.94	P	60.33	**2.94**	0.01	P	176.20	FLJ13611	5q12.3
230972_at	1.00	0.99	P	220.90	**2.94**	0.00	P	639.37	ANKRD9	14q32.33
242996_at	1.00	0.97	A	33.10	**2.94**	0.00	P	96.30	MTRF1	3q14.1-q14.3
203335_at	1.00	1.00	P	210.90	**2.94**	0.00	P	609.13	PHYH	10pter-p11.2
210788_s_at	1.00	0.95	P	293.60	**2.94**	0.00	P	849.97	DHRS7	14q23.1
229338_at	0.99	0.94	P,A	44.73	**2.93**	0.01	P	130.67	SR140	3q23
203627_at	1.00	0.98	P	107.13	**2.93**	0.00	P	309.53	IGF1R	15q26.3
231277_x_at	0.99	0.94	A	80.30	**2.93**	0.00	P	231.83	FLJ33977	5q23.1
241733_at	0.99	0.92	P,A	23.70	**2.93**	0.00	P	67.97	MGC33382	18q21.2
240558_at	0.99	0.94	A	22.73	**2.93**	0.02	P,A	66.37		
202929_s_at	1.00	0.96	P	546.20	**2.93**	0.00	P	1575.00	DDT	22q11.23
241836_x_at	0.99	0.94	P,A	70.20	**2.93**	0.00	P	201.53	FLJ23047	3q12.3
229474_at	1.00	0.98	P,A	42.63	**2.92**	0.00	P	122.67	MICAL3	22q11.21
205868_s_at	1.00	0.98	A	22.30	**2.92**	0.01	P	65.23	PTPN11	12q24
217949_s_at	1.00	0.99	P	531.33	**2.92**	0.00	P	1526.00	VKORC1	16p11.2
209582_s_at	0.99	0.93	A	23.73	**2.92**	0.00	P,A	68.17	MOX2	3q12-q13
235407_at	1.00	0.96	P,A	65.90	**2.92**	0.00	P	188.90	RABGEF1	7q11.21
208915_s_at	0.99	0.93	M,A	145.37	**2.92**	0.01	P	427.53	GGA2	16p12
230663_at	0.98	0.90	M,A	29.27	**2.92**	0.02	P	85.90	FMNL2	2q24.1
1555783_x_at	1.00	0.96	A	76.17	**2.92**	0.00	P	219.53	PQLC2	1p36.13
232674_at	0.98	0.89	A	17.03	**2.92**	0.00	P,M,A	48.77	UCN2	3p21.3
214543_x_at	0.99	0.94	P,A	135.57	**2.91**	0.00	P	391.37	QKI	6q26-27
236503_at	0.98	0.90	P,A	30.40	**2.91**	0.01	P	86.93		
228841_at	1.00	0.97	P	268.03	**2.91**	0.01	P	770.67	LOC90624	5q31.1
235114_x_at	0.99	0.94	P,M	77.00	**2.91**	0.01	P	223.17	HOOK3	8p11.21
222841_s_at	0.99	0.92	P	163.53	**2.91**	0.00	P	468.97	TIMM22	17p13
234926_s_at	1.00	0.96	P	834.97	**2.91**	0.00	P	2388.17	C20orf43	20q13.31
1553118_at	0.99	0.94	A	187.10	**2.91**	0.00	P	533.23	CTMP	1q21
1554266_at	1.00	0.99	P,A	15.73	**2.91**	0.01	P	45.67	TRIP11	14q31-q32
33736_at	0.99	0.93	P	81.40	**2.91**	0.00	P	233.37	STOML1	15q24-q25
218971_s_at	1.00	0.95	P,A	78.13	**2.91**	0.00	P	223.10	HSPC049	7q33
217737_x_at	0.99	0.94	P	860.17	**2.91**	0.00	P	2467.40	C20orf43	20q13.31
201521_s_at	1.00	0.98	P	281.43	**2.90**	0.00	P	805.17	NCBP2	3q29
218592_s_at	1.00	0.99	P	504.47	**2.90**	0.00	P	1444.67	CECR5	22q11.2
1559103_s_at	0.97	0.87	P	10.13	**2.90**	0.00	P	28.80		
225511_at	1.00	0.96	M,A	91.50	**2.90**	0.00	P	262.20	GPRC5B	16p12
1568877_a_at	1.00	0.96	P	78.50	**2.90**	0.01	P	227.10	ACBD5	10p12.1
1557292_a_at	0.99	0.93	P,A	44.70	**2.90**	0.01	P	127.37	MCOLN3	1p22.3
228522_at	0.99	0.92	P	67.77	**2.89**	0.00	P	192.17		
35160_at	0.98	0.91	P	88.13	**2.89**	0.00	P	250.03	LDB1	10q24-q25
232579_at	1.00	0.95	P,A	33.13	**2.89**	0.01	P	95.00		
213220_at	1.00	0.97	P	177.93	**2.89**	0.00	P	506.67	LOC92482	10q25.3
201809_s_at	1.00	0.95	A	56.37	**2.89**	0.00	P,M,A	160.80	ENG	9q33-q34.1
229265_at	1.00	0.98	P	354.63	**2.89**	0.00	P	1008.40	SKI	1q22-q24
215629_s_at	1.00	0.96	P	116.13	**2.89**	0.00	P	330.70	DLEU2	13q14.3
202288_at	0.99	0.93	P	49.07	**2.89**	0.01	P	140.40	FRAP1	1p36.2
204957_at	1.00	0.97	P	255.57	**2.89**	0.00	P	725.47	ORC5L	7q22.1
226625_at	1.00	0.98	P	131.27	**2.89**	0.00	P	373.27	TGFBR3	1p33-p32
208820_at	0.99	0.92	P	286.23	**2.88**	0.00	P	814.43	PTK2	8q24-qter
227539_at	0.99	0.92	P,A	64.63	**2.88**	0.00	P	183.57	GNA13	17q24.3
235277_at	0.99	0.95	A	15.10	**2.88**	0.00	P,A	43.07	AMOTL1	11q14.3
225549_at	0.97	0.87	P	113.77	**2.88**	0.02	P	328.57	DDX6	11q23.3
202124_s_at	0.99	0.93	P	499.83	**2.88**	0.00	P	1420.77	ALS2CR3	2q33
206662_at	0.99	0.94	P	269.80	**2.88**	0.00	P	767.20	GLRX	5q14
238002_at	0.98	0.91	P	252.93	**2.88**	0.01	P	727.27	GOLPH4	3q26.2
204502_at	1.00	0.95	P,A	63.77	**2.88**	0.01	P	183.60	SAMHD1	20pter-q12
225153_at	0.99	0.95	P	536.17	**2.88**	0.00	P	1513.93	EFG1	3q25.1-q26.2
218087_s_at	1.00	0.99	A	15.43	**2.88**	0.01	P	43.80	SORBS1	0q23.3-q24.1
208727_s_at	1.00	0.98	P	240.37	**2.87**	0.00	P	681.17	CDC42	1p36.1
206918_s_at	1.00	0.96	P	404.47	**2.87**	0.00	P	1143.93	CPNE1	20q11.23
1554149_at	1.00	0.97	P	443.30	**2.87**	0.00	P	1253.60	C3orf4	3p11-q11
212652_s_at	0.99	0.94	P	331.33	**2.87**	0.00	P	938.03	SNX4	3q21.2
203054_s_at	1.00	0.95	P	228.03	**2.87**	0.00	P	648.83	TCTA	3p21
200901_s_at	1.00	0.98	P	648.67	**2.87**	0.00	P	1831.40	M6PR	12p13
227012_at	1.00	0.96	P	315.17	**2.87**	0.00	P	892.30	MCFP	7q21.13
205010_at	1.00	0.95	P	177.73	**2.87**	0.01	P	501.83	FLJ10613	xp11.22
218440_at	1.00	0.97	P	180.10	**2.86**	0.00	P	508.07	MCCC1	3q27
200746_s_at	1.00	0.98	P	742.17	**2.86**	0.00	P	2092.20	GNB1	1p36.33
212349_at	1.00	0.97	P,A	67.27	**2.86**	0.00	P	189.87	POFUT1	20q11
226034_at	1.00	0.95	P	425.40	**2.86**	0.00	P	1203.13		
225878_at	1.00	0.97	P	220.83	**2.86**	0.00	P	621.63	KIF1B	1p36.2
225115_at	0.99	0.92	P	117.47	**2.86**	0.01	P	331.73	HIPK2	7q32-q34
227277_at	1.00	0.98	P	161.43	**2.86**	0.00	P	454.53	LYRIC	8q22.1
226146_at	1.00	0.97	P,M,A	139.93	**2.86**	0.00	P	392.63		
214531_s_at	1.00	0.96	P,A	223.77	**2.86**	0.00	P	631.23	SNX1	15q22.1
1554948_at	0.99	0.92	P	55.20	**2.86**	0.01	P	157.03	C22orf1	22q13.31
203387_s_at	1.00	0.99	P,M	220.17	**2.86**	0.00	P	620.07	TBC1D4	13q21.33
209286_at	1.00	0.99	P	201.33	**2.86**	0.01	P	567.97	CDC42EP3	2p21
208674_x_at	1.00	0.97	P	915.27	**2.85**	0.00	P	2579.33	DDOST	1p36.1
221802_s_at	1.00	0.99	P	434.57	**2.85**	0.00	P	1220.00	KIAA1598	10q26.12
211595_s_at	0.99	0.94	P	324.57	**2.85**	0.00	P	909.70	MRPS11	15q25
224669_at	0.99	0.95	P	127.57	**2.85**	0.00	P	358.53	C20orf35	20q13.12
217915_s_at	0.98	0.90	P	866.53	**2.85**	0.00	P	2438.27	C15orf15	15q21
235338_s_at	0.99	0.95	P,A	52.20	**2.85**	0.02	P	148.67	SETDB2	13q14
244841_at	0.99	0.93	P	41.13	**2.85**	0.02	P	116.57	SEC24A	5q31.2
223613_at	0.99	0.94	A	79.80	**2.85**	0.01	P	225.40	UQCR	19p13.3
218602_s_at	1.00	0.99	P	118.53	**2.85**	0.01	P	333.37	FAM29A	9p22.1
221739_at	1.00	0.99	P	506.37	**2.84**	0.00	P	1416.40	C19orf10	19p13.3
220865_s_at	1.00	0.97	P	279.70	**2.84**	0.00	P	783.63	TPRT	10p12.2

230706_s_at	1.00	0.95	P	97.00	**2.84**	0.00	P	272.63	MGC2408	3q27.3
202276_at	1.00	0.98	P	859.83	**2.84**	0.01	P	2412.33	SHFM1	7q21.3-q22.1
227756_at	1.00	0.96	M,A	38.40	**2.84**	0.00	P	107.20	MGC26690	15q21.3
222209_s_at	0.99	0.94	P	247.83	**2.84**	0.00	P	694.63	FLJ22104	11q14.1
208442_s_at	0.98	0.91	P	87.40	**2.84**	0.01	P	245.80	ATM	11q22-q23
1553750_a_at	0.97	0.89	P,M	20.57	**2.84**	0.00	P	57.90	MGC33371	11q21
226556_at	1.00	0.96	P	419.00	**2.84**	0.00	P	1171.17		
213763_at	0.99	0.93	A	79.47	**2.84**	0.00	P	222.47	HIPK2	7q32-q34
203695_s_at	1.00	0.97	A	58.27	**2.84**	0.00	P	163.23	DFNA5	7p15
231976_at	0.98	0.90	P	40.97	**2.84**	0.01	P	116.07	WINS1	15q26.3
235206_at	0.99	0.92	A	42.27	**2.84**	0.00	P,M	118.50	SCAND1	0q11.1-q11.23
228013_at	1.00	0.95	P	98.93	**2.84**	0.00	P	275.60	PPP2R2A	8p21.1
221882_s_at	1.00	0.96	P	190.43	**2.83**	0.00	P	535.77	TMEM8	16p13.3
202652_at	0.99	0.93	A	33.33	**2.83**	0.00	P,M,A	92.90	APBB1	11p15
1555882_at	0.99	0.93	P,A	70.83	**2.83**	0.00	P	198.17	LOC169981	xp11.22
242664_at	1.00	0.95	P,A	8.87	**2.83**	0.02	P	25.17		
215159_s_at	0.97	0.87	A	31.63	**2.83**	0.00	P	88.70	FLJ13052	36.33-p36.21
204388_s_at	0.99	0.93	A	20.63	**2.83**	0.01	P	58.67	MAOA	xp11.4-p11.3
233061_at	0.98	0.90	P	53.50	**2.83**	0.01	P	149.17	C20orf142	20q13.12
230416_at	0.99	0.93	P	126.67	**2.83**	0.00	P	353.77	SLC18A2	10q25
203196_at	1.00	0.97	P	180.80	**2.83**	0.00	P	504.03	ABCC4	13q32
225384_at	1.00	0.97	P	278.77	**2.83**	0.00	P	776.53	DOCK7	1p32.1
1552893_at	0.97	0.88	P	56.80	**2.83**	0.01	P	159.00	CAM-KIIN	3q27.3
214931_s_at	1.00	0.98	P	85.17	**2.83**	0.00	P	237.83	SRPK2	7q22-q31.1
228315_at	0.99	0.94	P	252.00	**2.83**	0.01	P	706.53		
201538_s_at	0.99	0.95	P,A	53.70	**2.83**	0.00	P	149.87	DUSP3	17q21
235219_at	0.99	0.94	P,A	20.63	**2.83**	0.00	P	57.43	LOC116349	5p15.33
222409_at	1.00	0.98	P	566.63	**2.82**	0.00	P	1571.97	CORO1C	12q24.1
210006_at	1.00	0.97	P	111.23	**2.82**	0.00	P	308.40	KFZP564O2	3p21.1
1558742_at	1.00	0.96	P	40.57	**2.82**	0.00	P	112.57	DEXI	16p13.2
203206_at	1.00	0.96	P,M,A	145.13	**2.82**	0.00	P	404.27	KIAA0140	10q26.2
213017_at	1.00	0.96	P	339.63	**2.82**	0.01	P	945.57	ABHD3	18q11.2
202234_s_at	1.00	0.98	P	59.73	**2.82**	0.01	P	165.80	SLC16A1	1p12
214835_s_at	1.00	0.97	P	640.93	**2.82**	0.01	P	1788.40	SUCLG2	3p14.3
200600_at	1.00	0.96	P	1055.00	**2.81**	0.00	P	2924.70	MSN	xq11.2-q12
233842_x_at	1.00	0.96	P	766.10	**2.81**	0.00	P	2122.80	C20orf43	20q13.31
227204_at	0.98	0.90	A	89.03	**2.81**	0.00	P	247.47	PARD6G	18q23
241453_at	1.00	0.96	P	37.20	**2.81**	0.00	P	102.40	PTK2	8q24-qter
233561_at	0.98	0.91	A	7.33	**2.81**	0.02	P,A	20.67		
224966_s_at	1.00	0.99	P,A	81.47	**2.81**	0.00	P	224.63	LOC56931	19p13.3
222129_at	1.00	0.99	P	180.83	**2.81**	0.01	P	500.30	C2orf17	2q36.1
225915_at	0.99	0.93	A	40.90	**2.80**	0.00	P	113.13	CAB39L	13q14.12
212820_at	1.00	0.99	P	161.43	**2.80**	0.00	P	444.63	RC3	15q15.3
200940_s_at	1.00	0.98	P	245.90	**2.80**	0.00	P	675.87	RERE	1p36.1-p36.2
203852_s_at	0.99	0.94	P	316.67	**2.80**	0.01	P	881.33	SMN1	5q13
214259_s_at	1.00	0.95	P	303.47	**2.80**	0.01	P	837.70	AKR7A2	p35.1-p36.23
242134_at	0.98	0.91	P,A	20.47	**2.80**	0.02	P	57.80		
212249_at	0.98	0.90	P	60.87	**2.80**	0.02	P	169.40	PIK3R1	5q13.1
1557567_a_at	1.00	0.95	M	22.67	**2.80**	0.01	P	62.90	LOC148987	1p32.3
205442_at	1.00	0.98	P	163.40	**2.80**	0.01	P	452.50	KIAA0626	4q32.3
230739_at	1.00	1.00	P	364.13	**2.80**	0.01	P	1004.60		
204408_at	1.00	0.96	P	240.43	**2.80**	0.00	P	664.10	APEX2	xp11.22
201823_s_at	1.00	0.97	P	454.67	**2.80**	0.00	P	1251.00	RNF14	5q23.3-q31.1
220735_s_at	0.98	0.91	A	12.67	**2.79**	0.00	P	35.00	SENP7	3q12
201752_s_at	0.98	0.90	P	227.43	**2.79**	0.00	P	627.03	ADD3	0q24.2-q24.3
218724_s_at	0.97	0.88	P,M,A	23.00	**2.79**	0.01	P	63.13	TGIF2	20q11.2-q12
226763_at	0.98	0.91	P	154.33	**2.79**	0.01	P	426.27	SESTD1	2q31.3
204371_s_at	1.00	0.97	A	44.33	**2.79**	0.00	P	121.80	KHSRP	19p13.3
205748_s_at	0.99	0.95	P	278.30	**2.78**	0.00	P	760.17	RNF126	19p13.3
202157_s_at	0.99	0.94	P	545.20	**2.78**	0.00	P	1496.00	CUGBP2	10p13
227696_at	1.00	0.96	P	255.97	**2.78**	0.00	P	700.90	EXOSC6	16q22.1
202369_s_at	0.98	0.91	P	340.83	**2.78**	0.00	P	939.73	TRAM2	6p21.1-p12
229173_at	0.99	0.93	P	168.60	**2.78**	0.00	P	463.87	KIAA1715	2q31
201734_at	1.00	0.96	P	462.93	**2.78**	0.00	P	1268.93	CLCN3	4q33
236417_at	0.99	0.92	P	39.17	**2.78**	0.01	P	108.13		
222572_at	0.99	0.93	P	311.77	**2.78**	0.00	P	856.07	PPM2C	8q22.1
219868_s_at	0.99	0.92	P,A	112.17	**2.78**	0.00	P	308.43	ANKFY1	17p13.3
223465_at	0.99	0.91	P	118.37	**2.78**	0.01	P	327.47	COL4A3BP	5q13.3
222416_at	0.99	0.94	P	445.30	**2.78**	0.02	P	1231.37	PYCS	10q24.3
1554447_at	1.00	0.95	P	24.10	**2.78**	0.01	P	66.03		
224359_s_at	0.99	0.92	A	28.40	**2.78**	0.00	P,A	77.93	HOOK3	8p11.21
242580_at	0.99	0.91	P,A	25.43	**2.78**	0.00	P	69.73		
201716_at	1.00	0.99	P,M	342.50	**2.77**	0.01	P	945.27	SNX1	15q22.1
239481_at	0.98	0.91	P,A	49.10	**2.77**	0.01	P	134.40	FLJ37659	xq21.33
235006_at	0.99	0.92	P	85.00	**2.77**	0.00	P	232.80	MGC13017	5q31.2
203092_at	0.99	0.93	P	93.90	**2.77**	0.01	P	258.23	TIMM44	9p13.3-p13.2
212665_at	0.99	0.94	P	266.37	**2.77**	0.00	P	726.33	TIPARP	3q25.31
203386_at	0.99	0.92	P	224.07	**2.77**	0.01	P	613.40	TBC1D4	13q21.33
1570315_at	0.98	0.91	P	73.93	**2.77**	0.00	P	200.27		16q22.3
221706_s_at	1.00	0.97	P,A	69.83	**2.77**	0.01	P	191.53	MDS032	19p13.12
227328_at	0.99	0.95	M,A	64.60	**2.77**	0.01	P	176.00	CAMTA1	1p36.23
200822_x_at	1.00	0.99	P	1722.70	**2.77**	0.00	P	4699.43	TPI1	12p13
235573_at	0.99	0.94	P	42.87	**2.76**	0.00	P	116.13		
222833_at	1.00	0.96	P	258.53	**2.76**	0.01	P	705.67	CAPNS2	16q13
204108_at	0.99	0.94	P,A	139.83	**2.76**	0.00	P	381.10	NFYA	6p21.3
215501_s_at	0.98	0.89	P,A	45.50	**2.76**	0.00	P	124.93	DUSP10	1q41
228390_at	1.00	0.95	P,A	29.00	**2.76**	0.02	P	79.90		
37433_at	0.99	0.94	P	34.10	**2.76**	0.00	P	92.23	MIZ1	18q21.1
227101_at	0.99	0.93	P	63.07	**2.76**	0.01	P	172.60	LOC168850	7q32.2
208453_s_at	0.99	0.95	P	187.27	**2.76**	0.00	P	512.23	XPNPEP1	10q25.3
241913_at	0.97	0.87	P	14.87	**2.76**	0.01	P	40.20		
52159_at	0.99	0.93	P	107.27	**2.76**	0.00	P	290.00	HEMK	3p21.3
226116_at	1.00	0.97	P	304.30	**2.76**	0.00	P	826.10		
225468_at	0.99	0.95	P,M	236.83	**2.75**	0.01	P	651.40	FLJ36874	11q12.2
217787_s_at	1.00	0.95	P	121.77	**2.75**	0.00	P	330.33	GALNT2	1q41-q42
235694_at	1.00	0.97	P	121.70	**2.75**	0.00	P	328.90	TCFL5	20q13.3-qter
1558329_at	1.00	0.98	A	59.73	**2.75**	0.00	P,M	161.37	NFKBIL2	8q24.3
239066_at	0.98	0.89	A	29.00	**2.75**	0.02	P,M	79.50		
207231_at	0.98	0.91	P	26.90	**2.74**	0.00	P	73.07	DZIP3	3q13.13
36566_at	1.00	0.98	P	121.57	**2.74**	0.00	P	328.17	CTNS	17p13
203253_s_at	1.00	0.96	P	374.07	**2.74**	0.00	P	1011.30	KIAA0433	5q21.2
203674_at	1.00	0.99	P	184.30	**2.74**	0.00	P	496.53	HELZ	17q24.3
238573_at	0.99	0.92	M,A	70.10	**2.74**	0.01	P	190.23		
242051_at	0.99	0.93	A	16.23	**2.74**	0.01	P	44.30		
204440_at	0.98	0.91	P	130.33	**2.74**	0.00	P	352.60	CD83	6p23
244726_at	0.99	0.93	P,A	67.73	**2.74**	0.00	P	182.97		
235484_at	0.97	0.88	P	117.93	**2.74**	0.01	P	320.67	PTAR1	9q21.13
239329_at	0.99	0.93	P	38.63	**2.74**	0.00	P	104.47	RAB21	12q15
200911_s_at	1.00	0.98	P	524.03	**2.73**	0.00	P	1411.17	TACC1	8p11
241584_at	0.98	0.91	P,A	34.53	**2.73**	0.00	P	93.40		
1552302_at	0.99	0.93	P	21.00	**2.73**	0.01	P	56.80	MGC20235	17q21.31
211801_x_at	1.00	0.96	P,A	118.40	**2.73**	0.00	P	319.73	MFN1	3q27.1
1556865_at	0.99	0.92	A	21.83	**2.73**	0.01	P	59.20		
226208_at	1.00	0.95	P	167.00	**2.73**	0.00	P	449.53	ZSWIM6	5q12.1
203212_s_at	0.99	0.95	P,A	191.10	**2.73**	0.00	P	514.40	MTMR2	11q22
1555889_a_at	1.00	0.97	P	981.10	**2.73**	0.00	P	2640.20	CRTAP	3p22
208728_s_at	0.98	0.90	P	155.23	**2.73**	0.01	P	422.27	CDC42	1p36.1
229940_at	0.99	0.95	P,M	27.33	**2.73**	0.01	P	74.20	FLJ23027	14q32.32
236192_at	1.00	0.99	P	71.73	**2.73**	0.00	P	192.73		
238435_at	0.98	0.89	P	67.80	**2.73**	0.01	P	182.67	LOC340591	Xp22.31
226711_at	1.00	0.99	P	352.07	**2.73**	0.00	P	943.33	HTLF	2p22-p16
1558021_at	0.98	0.91	P,A	26.90	**2.72**	0.00	P	72.33	RAB9P40	9q34.11
234311_s_at	0.99	0.92	P	144.63	**2.72**	0.00	P	389.37	LOC85865	7q21.2
226087_at	0.99	0.93	P	79.17	**2.72**	0.01	P	212.03	LZIC	1p36.22
218374_s_at	0.98	0.91	P	123.53	**2.72**	0.00	P	333.03	C12orf4	12p13.3
220688_s_at	1.00	0.98	P	290.27	**2.72**	0.01	P	783.80	C1orf33	1p36.13
214553_s_at	0.99	0.94	P	130.17	**2.72**	0.00	P	349.57	ARPP-19	15q21.1
204146_at	0.99	0.95	P	369.83	**2.72**	0.00	P	993.20	PIR51	2p13.2-p13.1
204521_at	0.99	0.92	P	88.23	**2.72**	0.01	P	237.20	HSU79274	12q24.13
201824_at	1.00	0.99	P	421.57	**2.72**	0.00	P	1128.27	RNF14	5q23.3-q31.1
1557363_a_at	0.99	0.94	P,A	8.27	**2.72**	0.00	P	22.20	PHIP	6q14
214657_s_at	0.99	0.93	P	65.97	**2.72**	0.01	P	177.57		
226923_at	1.00	0.95	P	321.97	**2.71**	0.00	P	858.17	SCFD2	4q12
229735_s_at	0.99	0.94	P	54.50	**2.71**	0.00	P	147.07	DJ462O23.2	p36.12-p35.1

242239_at	0.98	0.90	P	34.50	**2.71**	0.02	P	92.87	NOPD1	10p13
225946_at	0.99	0.92	P	92.47	**2.71**	0.01	P	249.47	C12orf2	12p12.3
231920_s_at	0.98	0.90	P	98.30	**2.71**	0.02	P	268.60	CSNK1G1	5q22.1-q22.31
205957_at	0.98	0.91	P	111.17	**2.71**	0.00	P	297.43	PLXNB3	xq28
202320_at	0.99	0.93	A	160.00	**2.71**	0.00	P	429.00	GTF3C1	16p12
213816_s_at	0.99	0.94	P,A	64.60	**2.71**	0.00	P	173.33	MET	7q31
1569024_at	0.98	0.91	P	38.03	**2.71**	0.00	P	102.63		4q22.1
209086_x_at	1.00	0.99	A	102.00	**2.71**	0.00	P	272.03	MCAM	11q23.3
238511_at	0.98	0.91	A	22.63	**2.71**	0.01	P,A	61.50		
227068_at	0.99	0.93	P	813.33	**2.71**	0.00	P	2174.07	PGK1	xq13
200743_s_at	0.99	0.94	P	388.07	**2.71**	0.00	P	1028.97	CLN2	11p15
201985_at	0.98	0.92	P	253.60	**2.71**	0.01	P	682.67	KIAA0196	8p22
213224_s_at	1.00	0.96	P	73.50	**2.70**	0.00	P	195.33	LOC92482	10q25.3
211213_at	1.00	0.96	P,M	11.67	**2.70**	0.01	P	31.20	ORC5L	7q22.1
202296_s_at	1.00	0.98	P	704.30	**2.70**	0.00	P	1873.73	RER1	1pter-q24
202913_at	1.00	0.97	M,A	138.70	**2.70**	0.00	P	368.87	ARHGEF11	1q21
214155_s_at	0.99	0.93	P	151.57	**2.70**	0.00	P	404.13	LOC113251	12q13.12
232048_at	0.99	0.92	P	33.70	**2.70**	0.00	P	89.77	MGC33371	11q21
1556081_at	0.98	0.89	P,A	19.57	**2.70**	0.03	P	53.90		
226859_at	1.00	0.99	P	90.13	**2.69**	0.01	P	240.10	GNG10	9q32
213093_at	1.00	0.97	P	85.00	**2.69**	0.00	P	224.77	PRKCA	17q22-q23.2
235030_at	1.00	0.96	P,A	25.47	**2.69**	0.02	P	68.67	MGC15606	3q12.3
217599_s_at	0.99	0.92	P	141.40	**2.69**	0.01	P	378.47	HIC	7q31.2
225213_at	0.99	0.94	P	329.60	**2.69**	0.00	P	875.17	TA-PP2C	12q24.13
243636_s_at	0.97	0.88	P,A	14.80	**2.69**	0.02	P	39.93		
212239_at	0.99	0.94	P	123.93	**2.69**	0.01	P	329.60	PIK3R1	5q13.1
232643_at	0.99	0.94	A	27.20	**2.69**	0.02	P,A	72.83		
218152_at	1.00	0.97	P	251.80	**2.69**	0.00	P	667.77	HMG20A	15q24
1564381_s_at	0.99	0.92	P	39.03	**2.69**	0.01	P	103.83		
239989_at	1.00	0.95	P	31.87	**2.69**	0.00	P	84.37		
218529_at	0.98	0.90	P	185.50	**2.69**	0.00	P	488.83	8D6A	9p13.3-p13.2
234157_at	1.00	0.98	P,A	49.07	**2.69**	0.00	P,M	129.43		
224058_s_at	0.99	0.93	P,M,A	51.67	**2.68**	0.00	P	136.67	LOC158160	10p11.21
234106_s_at	0.98	0.90	A	51.23	**2.68**	0.00	P	135.97	KFZp761A1	16p13.3
227337_at	0.99	0.92	A	46.23	**2.68**	0.00	P	122.43	Lrp2bp	4q35.1
225823_at	0.99	0.92	P	573.40	**2.68**	0.00	P	1507.27	QIL1	19p13.3
231835_at	0.99	0.94	P,A	114.10	**2.68**	0.00	P	300.50	MGC26818	1p36.32
241730_at	1.00	0.98	P	24.63	**2.68**	0.01	P	65.13	MYNN	3q26.31
228831_s_at	1.00	0.99	P	53.73	**2.68**	0.00	P	141.57	GNG7	19p13.3
243345_at	0.99	0.92	P	29.50	**2.68**	0.03	P	78.90	RNF14	5q23.3-q31.1
226389_s_at	1.00	0.96	A	71.30	**2.68**	0.00	P	188.63	RAPGEF1	9q34.3
1570552_at	1.00	0.96	P	38.47	**2.68**	0.01	P	101.67	FLJ33761	18q11.2
1567997_x_at	1.00	0.97	A	15.20	**2.68**	0.00	P,A	40.03		
208917_x_at	1.00	0.99	P,A	51.50	**2.68**	0.01	P	136.23	FLJ13052	p36.33-p36.21
224821_at	1.00	0.96	P	492.57	**2.67**	0.00	P	1296.37	MGC15429	3p21.31
230123_at	0.98	0.91	A	55.03	**2.67**	0.00	P	145.97	FLJ10420	1p36.13
225056_at	0.99	0.94	P	202.70	**2.67**	0.02	P	538.17	SIPA1L2	1q42.2
225424_at	1.00	0.97	P	87.77	**2.67**	0.01	P	231.00	GPAM	10q25.3
219973_at	1.00	0.96	P	75.67	**2.67**	0.00	P	199.50	FLJ23548	4q26
215694_at	0.98	0.89	P,A	17.87	**2.67**	0.01	P	47.50	SPATA5L1	15q15.1
204819_at	0.99	0.94	P	125.97	**2.67**	0.00	P	330.20	FGD1	xp11.21
208675_s_at	1.00	0.98	P	925.43	**2.67**	0.00	P	2432.73	DDOST	1p36.1
206092_x_at	1.00	0.95	P,A	62.00	**2.67**	0.00	P	163.17	C20orf41	20q13.3
209553_at	0.98	0.89	P	118.17	**2.67**	0.00	P	311.30	KIAA0804	3q28
207030_s_at	0.99	0.93	P	97.23	**2.67**	0.00	P	254.83	CSRP2	12q21.1
224800_at	1.00	0.99	P	731.00	**2.66**	0.00	P	1916.30	WDFY1	2q36.3
225789_at	0.98	0.91	M,A	172.03	**2.66**	0.01	P	449.33	CENTG3	7q36.1
211572_s_at	0.99	0.94	P	57.47	**2.66**	0.00	P	150.30	SLC23A2	20p13
238563_at	0.99	0.92	P	60.10	**2.66**	0.00	P	158.23	ABI1	10p11.2
228856_at	0.98	0.91	P,A	29.50	**2.66**	0.00	P	77.60	MGC2474	16p11.2
202043_s_at	1.00	0.99	P	951.30	**2.66**	0.00	P	2492.43	SMS	xp22.1
238174_at	0.97	0.88	P	14.00	**2.66**	0.01	P	36.73		
202424_at	1.00	0.96	P	957.53	**2.66**	0.00	P	2497.60	MAP2K2	19p13.3
228043_at	1.00	0.98	P	50.10	**2.66**	0.01	P	132.50	FLJ12787	5q13.3
239466_at	1.00	0.96	P	46.90	**2.66**	0.01	P	123.20		3q13.12
204317_at	0.98	0.91	P	76.83	**2.66**	0.01	P	202.23	GTSE1	2q13.2-q13.3
224670_at	0.99	0.92	P	102.50	**2.66**	0.00	P	268.87	C20orf35	20q13.12
223443_s_at	0.99	0.93	P	100.63	**2.65**	0.01	P	265.27	FLJ32065	17q24.2
219148_at	1.00	0.97	P	867.73	**2.65**	0.00	P	2274.10	TOPK	8p21.2
229348_at	0.99	0.92	P,M	134.53	**2.65**	0.01	P	352.40	TERE1	1pter
226341_at	0.99	0.92	P	93.57	**2.65**	0.01	P	246.57		
225623_at	1.00	0.98	P	171.53	**2.65**	0.00	P	448.60	KIAA1737	14q24.3
228228_at	1.00	0.98	P	85.63	**2.65**	0.00	P	224.53	MGC15476	19q13.33
212747_at	1.00	0.95	P	755.50	**2.65**	0.00	P	1975.57	ANKS1	6p21.31
240113_at	0.98	0.89	P,M	42.77	**2.65**	0.02	P	112.40		
225760_at	0.98	0.91	P	236.87	**2.65**	0.00	P	621.17	KIAA1915	1p32.1
235263_at	0.99	0.93	P	121.43	**2.65**	0.01	P	318.93	KFZP434A01	q11.23-q21.1
208883_at	0.99	0.93	P	53.13	**2.65**	0.02	P	139.83	DD5	8q22
229819_at	0.98	0.90	A	25.50	**2.65**	0.02	P	68.50	A1BG	19q13.4
223108_s_at	0.99	0.95	P	255.87	**2.65**	0.00	P	667.90	PS1D	1p35.1
207769_s_at	1.00	0.98	P	275.97	**2.65**	0.01	P	721.67	PQBP1	xp11.23
229994_at	0.98	0.90	P,A	13.30	**2.65**	0.03	P	35.93		
212841_s_at	0.99	0.94	A	54.67	**2.65**	0.01	P	142.67	PPFIBP2	11p15.4
212144_at	1.00	0.98	P	252.23	**2.65**	0.00	P	656.33	UNC84B	22q13.1
225352_at	1.00	0.98	P	215.63	**2.65**	0.00	P	561.43	TLOC1	3q26.2-q27
1555989_at	0.98	0.91	P	102.20	**2.64**	0.00	P	267.87	DAAM1	14q23.1
225143_at	0.99	0.93	P	335.60	**2.64**	0.00	P	869.53	SFXN4	10q26.13
225240_s_at	1.00	0.97	P	194.43	**2.64**	0.01	P	508.23	MSI2	17q23.2
221531_at	0.99	0.92	P	601.87	**2.64**	0.00	P	1571.63	REC14	15q24.1
1558409_at	1.00	0.96	A	16.17	**2.64**	0.01	P	42.20		
226972_s_at	1.00	0.98	M,A	43.03	**2.64**	0.00	P	112.07	KFZP434G1	7q33
213216_at	0.99	0.92	P	61.83	**2.64**	0.01	P	161.67	KIAA0459	1p36.13
202946_s_at	1.00	0.97	P	359.93	**2.63**	0.00	P	934.23	BTBD3	20p12.1
228776_at	0.99	0.94	P	389.47	**2.63**	0.00	P	1013.33		
209853_s_at	0.99	0.95	P	253.13	**2.63**	0.01	P	662.73	PSME3	17q21
203010_at	0.99	0.95	P,A	160.27	**2.63**	0.01	P	414.20	STAT5A	17q11.2
204394_at	1.00	0.96	P,A	83.57	**2.63**	0.01	P	216.97	SLC43A1	1p11.2-p11.1
232113_at	0.98	0.89	A	12.50	**2.63**	0.02	P	32.57		
211994_at	0.98	0.90	P	282.57	**2.63**	0.00	P	734.47	PRKWNK1	12p13.3
211126_s_at	1.00	0.98	P	77.90	**2.63**	0.00	P	201.50	CSRP2	12q21.1
218867_s_at	0.99	0.95	P,A	139.90	**2.63**	0.00	P	362.47	FLJ21415	12q24.22
238831_at	1.00	0.98	P	50.60	**2.62**	0.01	P	131.67	FLJ10525	4p14
231858_x_at	0.98	0.90	M,A	34.77	**2.62**	0.00	P	90.27	KFZp761E1	11q13.1
223413_s_at	1.00	0.98	P	294.93	**2.62**	0.00	P	761.90	LYAR	4p16.2
226279_at	1.00	0.96	P	248.10	**2.62**	0.00	P	642.30	SPUVE	11q14.1
236832_at	0.97	0.88	A	30.00	**2.62**	0.02	P	77.70	LOC221442	6p21.1
222421_at	1.00	0.95	P	391.77	**2.62**	0.00	P	1012.87	UBE2H	7q32
223286_at	0.99	0.93	P	239.57	**2.62**	0.01	P	626.13	DERP6	17p13.2
230561_s_at	0.98	0.90	P	23.20	**2.62**	0.00	P	60.17	FLJ23861	2q34
241865_at	0.98	0.89	P	39.63	**2.62**	0.01	P	102.20		
236381_s_at	0.98	0.90	P	100.60	**2.62**	0.00	P	259.87	WDR8	1p36.3
242918_at	0.98	0.90	A	26.57	**2.62**	0.02	P,M	69.83	NASP	1p34.1
228168_at	0.98	0.90	P,A	52.93	**2.62**	0.01	P	136.93	ATP5G3	2q31.2
204104_at	1.00	0.96	P,A	78.17	**2.62**	0.02	P	205.07	SNAPC2	9p13.3-p13.2
213011_s_at	1.00	0.99	P	1637.73	**2.62**	0.00	P	4220.33	TPI1	12p13
212911_at	1.00	0.97	P	49.07	**2.62**	0.00	P	126.40	KIAA0962	1p36.1
221958_s_at	0.99	0.91	P	137.70	**2.61**	0.00	P	355.80	FLJ23091	1p31.2
213008_at	0.98	0.91	P	303.37	**2.61**	0.00	P	783.13	FLJ10719	15q25-q26
239035_at	0.99	0.92	P,A	44.30	**2.61**	0.01	P	115.07	MTHFR	1p36.3
202537_s_at	0.99	0.93	P	465.10	**2.61**	0.01	P	1202.93	KFZP564O1	3p12.1
223053_x_at	1.00	0.95	P	292.77	**2.61**	0.00	P	754.57	HSPC182	1p36.33
228830_s_at	0.98	0.91	P	68.13	**2.61**	0.00	P	175.80	ATF7	12q13
214527_s_at	1.00	0.95	P	350.17	**2.61**	0.00	P	900.77	PQBP1	xp11.23
244802_at	0.99	0.95	P,A	27.83	**2.61**	0.01	P	71.93		
225173_at	1.00	0.97	P	120.60	**2.61**	0.02	P	312.63	ARHGAP18	6q23.1
201304_at	1.00	0.95	P	691.83	**2.61**	0.00	P	1778.47	NDUFA5	7q32
207098_s_at	1.00	0.96	P	315.53	**2.61**	0.00	P	810.73	MFN1	3q27.1
1558220_at	0.99	0.92	P	61.33	**2.61**	0.01	P	158.93	LOC255812	3q29
1555247_a_at	0.99	0.92	P,A	33.87	**2.61**	0.00	P	87.30	RAPGEF6	5q31.1
204967_at	0.99	0.92	P,A	111.10	**2.60**	0.00	P	285.33	APXL	xp22.3
208876_s_at	0.98	0.90	P	124.53	**2.60**	0.01	P	322.03	PAK2	3q29
242336_at	0.98	0.91	P,A	21.97	**2.60**	0.00	P	56.63	GSK3B	3q13.3
222999_s_at	1.00	0.97	P	167.77	**2.60**	0.01	P	431.10	CCNL2	1p36.33
204172_at	1.00	0.96	P	617.67	**2.60**	0.00	P	1583.27	CPOX	3q12

1554148_a_at	0.98	0.91	P	94.70	**2.60**	0.00	P	243.43	SLC33A1	3q25.31
213607_x_at	1.00	0.95	M,A	92.63	**2.60**	0.02	P	238.47	FLJ13052	36.33-p36.21
225924_at	1.00	0.96	P	148.53	**2.60**	0.00	P	379.20	KIAA1450	4q32.1
202382_s_at	1.00	0.96	P	482.13	**2.59**	0.00	P	1231.33	GNPDA1	5q21
222623_s_at	0.98	0.91	P	345.47	**2.59**	0.00	P	883.93	ANC_2H01	3q27.1
238609_at	1.00	0.99	P	47.83	**2.59**	0.01	P	122.17		
238557_at	0.99	0.91	A	29.93	**2.59**	0.02	P	76.70		
1556082_a_at	0.99	0.94	A	15.27	**2.59**	0.00	P,M,A	39.00		
209350_s_at	1.00	0.99	P	324.20	**2.59**	0.00	P	825.23	GPS2	17p13
221535_at	1.00	0.99	P	249.60	**2.59**	0.00	P	634.93	FLJ11301	3q29
200961_at	1.00	0.98	P	519.33	**2.59**	0.00	P	1322.23	SEPHS2	16p12.1
227562_at	1.00	0.97	P	56.63	**2.59**	0.01	P	144.63	MAP2K1IP1	4q23
223296_at	1.00	0.98	P	263.00	**2.59**	0.00	P	668.43	MGC4399	1p36.22
236619_at	1.00	0.95	P	36.40	**2.59**	0.03	P	94.17		
226276_at	0.99	0.93	P	1116.30	**2.58**	0.00	P	2851.00	MGC23909	5q14.2
1555870_at	1.00	0.96	P	123.87	**2.58**	0.00	P	315.43	FLJ32096	1p36.23
201881_s_at	0.98	0.91	P	117.50	**2.58**	0.00	P	301.07	ARIH1	15q24
219143_s_at	1.00	0.96	P,A	191.80	**2.58**	0.00	P	489.17	RPP25	15q23
213374_x_at	0.99	0.93	P	919.60	**2.58**	0.00	P	2342.73	HIBCH	2q32.3
227188_at	0.99	0.93	P	55.23	**2.58**	0.02	P	140.93	C21orf63	21q22.11
223538_at	1.00	0.97	P	308.43	**2.58**	0.00	P	783.17	SERF1A	5q12.2-q13.3
213447_at	0.99	0.92	P	68.13	**2.58**	0.02	P	176.30	IPW	15q11-q12
203628_at	0.99	0.94	M,A	105.97	**2.58**	0.01	P	269.90	IGF1R	15q26.3
202526_at	0.98	0.90	A	12.97	**2.58**	0.00	P	33.07	SMAD4	18q21.1
40020_at	0.99	0.92	P	66.53	**2.58**	0.01	P	169.67	CELSR3	3p24.1-p21.2
203150_at	1.00	0.99	P	242.93	**2.58**	0.00	P	615.97	RAB9P40	9q34.11
210983_s_at	1.00	0.98	P,A	854.97	**2.58**	0.00	P	2180.80	MCM7	7q21.3-q22.1
222447_at	1.00	0.99	P	309.10	**2.58**	0.00	P	783.80	DREV1	16p13-p12
227936_at	0.99	0.95	P	168.60	**2.57**	0.00	P	428.07	FLJ32370	8q11.23
204929_s_at	1.00	0.98	P,A	48.67	**2.57**	0.01	P	123.73	VAMP5	2p11.2
214564_s_at	1.00	0.97	A	71.17	**2.57**	0.01	P	182.07	PCDHGC3	5q31
208700_s_at	1.00	0.97	P	1518.67	**2.57**	0.00	P	3849.47	TKT	3p14.3
203201_at	0.99	0.93	P,A	80.60	**2.57**	0.00	P	203.17	PMM2	6p13.3-p13.2
219239_s_at	1.00	0.98	A	48.50	**2.57**	0.00	P,A	122.97	FLJ10997	3p12.1
221504_s_at	1.00	0.97	P	291.83	**2.57**	0.02	P	746.17	ATP6V1H	8p22-q22.3
202111_at	0.98	0.91	P	301.73	**2.57**	0.00	P	766.67	SLC4A2	7q35-q36
238960_s_at	0.98	0.90	P,A	25.47	**2.57**	0.01	P	65.13	LOC113251	12q13.12
218108_at	1.00	0.95	P	344.70	**2.57**	0.00	P	872.70	C14orf130	14q32.13
241495_at	1.00	0.96	P,M	16.40	**2.57**	0.00	P	41.53	CCNL1	3q25.32
226805_at	1.00	0.96	P	210.33	**2.57**	0.00	P	532.43	C20orf142	20q13.12
224521_s_at	0.99	0.95	P	66.07	**2.56**	0.00	P	166.50	MGC13183	12p13.33
203635_at	0.99	0.93	P	361.37	**2.56**	0.00	P	914.37	DSCR3	21q22.2
202313_at	0.99	0.92	P	407.07	**2.56**	0.00	P	1029.53	PPP2R2A	8p21.1
201622_at	0.99	0.94	P	360.33	**2.56**	0.00	P	904.77	SND1	7q31.3
206501_x_at	1.00	0.98	P,A	53.93	**2.56**	0.00	P	135.93	ETV1	7p22
230521_at	0.99	0.95	P	173.83	**2.56**	0.01	P	443.20	C9orf100	9p13.2
1557352_at	1.00	0.96	P,A	13.07	**2.56**	0.00	P	33.07	SQLE	8q24.1
218897_at	1.00	0.96	P	144.40	**2.56**	0.00	P	364.17	MGC10993	2q14.2
202611_s_at	1.00	0.96	P	154.00	**2.56**	0.01	P	388.80	CRSP2	xp11.4-p11.2
1559759_at	1.00	0.97	A	68.53	**2.56**	0.02	P	173.70	KIFC3	16q13-q21
212685_s_at	1.00	0.95	P	374.70	**2.56**	0.00	P	940.67	TBL2	7q11.23
33760_at	1.00	0.97	P	144.20	**2.56**	0.00	P	362.07	PEX14	1p36.22
208101_s_at	1.00	0.96	P	281.17	**2.56**	0.00	P	710.17	C9orf74	9q34.13
1554456_a_at	0.98	0.90	P,A	87.80	**2.55**	0.00	P	221.80	WINS1	15q26.3
225876_at	0.99	0.94	P	217.73	**2.55**	0.01	P	547.50	DJ462O23.2	p36.12-p35.1
223039_at	1.00	0.97	P	569.00	**2.55**	0.00	P	1430.90	MGC1842	22q11.2
202241_at	0.99	0.94	P	347.40	**2.55**	0.01	P	872.23	TRIB1	8q24.13
210109_at	0.97	0.89	A	58.53	**2.55**	0.01	P,A	148.47	NSG-X	7q31
201173_x_at	0.99	0.93	P	611.80	**2.55**	0.00	P	1529.20	NUDC	1p35-p34
231845_at	1.00	0.97	P	209.10	**2.55**	0.01	P	525.73	AARSL	6p21.1
216397_s_at	0.98	0.91	P,A	108.97	**2.55**	0.00	P	274.50	BOP1	8q24.3
200979_at	1.00	0.96	P	318.77	**2.55**	0.00	P	798.80	PDHA1	Xp22.2-p22.1
242981_at	0.99	0.92	P	54.50	**2.55**	0.00	P	137.30		
222547_at	0.99	0.92	P	60.07	**2.55**	0.00	P	151.20	MAP4K4	2q11.2-q12
1555943_at	0.99	0.93	A	70.07	**2.54**	0.00	P	176.17	MGC5352	12q24.33
1556321_a_at	1.00	0.98	P,M	68.57	**2.54**	0.01	P	171.80		
235123_at	1.00	0.98	P	94.77	**2.54**	0.01	P	238.37		
237464_at	0.99	0.94	P	44.63	**2.54**	0.00	P	111.60	IMAA	16p12
222912_at	0.99	0.93	P	92.40	**2.54**	0.01	P	230.50	ARRB1	11q13
200866_s_at	0.99	0.93	P	817.93	**2.54**	0.00	P	2051.20	PSAP	10q21-q22
215905_s_at	0.98	0.90	P,A	151.47	**2.54**	0.00	P	380.83	HPRP8BP	1p35.1
218273_s_at	1.00	0.96	P	154.10	**2.54**	0.01	P	387.90	PPM2C	8q22.1
219137_s_at	1.00	0.95	P	409.57	**2.54**	0.00	P	1026.83	GL004	2q36.3
203451_at	0.99	0.94	A	75.10	**2.54**	0.00	P,M,A	187.50	LDB1	10q24-q25
226968_at	1.00	0.96	P	96.23	**2.54**	0.01	P	241.90	KIF1B	1p36.2
219112_at	0.99	0.93	P,A	44.43	**2.53**	0.01	P	110.73	RAPGEF6	5q31.1
209237_s_at	0.98	0.90	A	48.93	**2.53**	0.01	P,A	123.07	SLC23A2	20p13
242008_at	0.99	0.93	P	25.47	**2.53**	0.01	P	63.73		
203775_at	1.00	0.98	P	493.70	**2.53**	0.00	P	1230.20	SLC25A13	7q21.3
209811_at	0.99	0.93	P,A	71.20	**2.53**	0.00	P	178.73	CASP2	7q34-q35
236292_at	0.99	0.94	P	77.40	**2.53**	0.00	P	192.13		
221482_s_at	1.00	0.96	P	478.53	**2.53**	0.00	P	1195.37	ARPP-19	15q21.1
209420_s_at	1.00	0.96	M,A	146.80	**2.53**	0.00	P	365.07	SMPD1	1p15.4-p15.1
225837_at	1.00	0.99	P	173.57	**2.53**	0.00	P	431.60	MGC13204	12p13.33
239288_at	0.99	0.94	A	23.33	**2.53**	0.00	P	57.87	TNIK	3q26.31
238320_at	0.98	0.89	P,A	69.27	**2.53**	0.01	P	174.83	TncRNA	11q13.1
229712_at	0.99	0.92	P,A	29.90	**2.53**	0.00	P	74.73	SNAPC3	9p22.2
239439_at	0.98	0.90	P,A	43.90	**2.53**	0.03	P	112.33	AF5Q31	5q31
226656_at	0.99	0.95	P	281.47	**2.53**	0.01	P	701.83		
208878_s_at	1.00	0.98	P	231.83	**2.52**	0.00	P	574.90	PAK2	3q29
244519_at	1.00	0.98	P,M	158.00	**2.52**	0.00	P	392.00	ASXL1	20q11.1
220032_at	1.00	0.95	P	75.80	**2.52**	0.00	P	188.03	FLJ21986	7q31.32
203883_s_at	1.00	0.99	P	160.50	**2.52**	0.01	P	398.93	Rab11-FIP2	10q26.12
203452_at	0.98	0.89	P,A	142.93	**2.52**	0.01	P	356.63	B3GAT3	11q12.3
235234_at	0.98	0.91	P,M,A	80.13	**2.52**	0.00	P	200.17	FLJ36874	11q12.2
204015_s_at	0.99	0.93	P	347.90	**2.52**	0.00	P	864.37	DUSP4	8p12-p11
240146_at	0.97	0.89	P	27.03	**2.52**	0.01	P	67.47	CAPZA2	7q31.2-q31.3
207265_s_at	0.99	0.93	P	224.37	**2.52**	0.01	P	561.90	KDELR3	22q13.1
200972_at	0.99	0.92	P	805.27	**2.52**	0.00	P	2001.87	TM4SF8	15q24.1
218300_at	0.99	0.92	P,A	162.17	**2.52**	0.01	P	402.50	MGC4606	16p12.1
204761_at	0.99	0.92	P	107.10	**2.52**	0.00	P	266.23	USP6NL	10p13
204849_at	1.00	0.99	P	227.37	**2.52**	0.00	P	563.03	TCFL5	20q13.3-qter
230077_at	0.98	0.91	P	37.10	**2.51**	0.01	P	92.73	LOC255812	3q29
224859_at	1.00	0.96	P	176.87	**2.51**	0.01	P	442.07	B7H3	15q23-q24
235949_at	1.00	0.98	P	38.37	**2.51**	0.01	P	95.43		
216088_s_at	1.00	0.99	P	493.00	**2.51**	0.01	P	1223.00	PSMA7	20q13.33
200987_x_at	0.99	0.93	P,A	424.67	**2.51**	0.00	P	1056.17	PSME3	17q21
211961_s_at	1.00	1.00	P	975.47	**2.51**	0.00	P	2406.50	RAB7	3q21.3
202661_at	0.99	0.94	P,A	25.30	**2.51**	0.00	P	62.27	ITPR2	12p11
218456_at	0.99	0.94	P	117.03	**2.51**	0.00	P	289.60	C1QDC1	12p11
214074_s_at	0.99	0.95	P	175.03	**2.51**	0.00	P	433.83	EMS1	11q13
208626_s_at	1.00	0.95	P	1138.50	**2.51**	0.00	P	2805.13	VAT1	17q21
203685_at	0.99	0.94	P,A	64.33	**2.50**	0.00	P	159.10	BCL2	18q21.3
204014_at	1.00	0.99	P,M	193.10	**2.50**	0.01	P	477.70	DUSP4	8p12-p11
209568_s_at	1.00	0.96	P	111.17	**2.50**	0.00	P	272.83	RGL1	1q25.2
222045_s_at	0.98	0.90	P,A	52.93	**2.50**	0.01	P	132.63	C20orf67	20q13.12
228889_at	0.99	0.92	A	46.10	**2.50**	0.00	P,A	113.17	C14orf128	14q13.1
216222_s_at	0.98	0.90	P	160.67	**2.50**	0.02	P	402.63	MYO10	5p15.1-p14.3
228171_s_at	1.00	0.98	P	217.57	**2.50**	0.00	P	534.47	KFZP434I21	16q22.1
202070_s_at	1.00	0.97	P	317.87	**2.50**	0.00	P	781.70	IDH3A	5q25.1-q25.2
236122_at	1.00	0.97	P	93.30	**2.50**	0.00	P	229.33		13q13.3
212240_s_at	1.00	0.96	P	141.30	**2.50**	0.00	P	347.77	PIK3R1	5q13.1
215866_at	1.00	0.96	A	34.33	**2.50**	0.00	P,A	84.50		
208657_s_at	0.99	0.95	P	238.10	**2.50**	0.01	P	588.07	MSF	17q25
236565_s_at	1.00	0.97	P	144.90	**2.50**	0.01	P	358.40	FLJ11196	15q22.32
231954_at	0.99	0.95	P	57.50	**2.50**	0.01	P	142.67	KFZP434I07	4q31.23
239313_at	0.99	0.95	A	59.87	**2.49**	0.00	P,A	147.10		7
218109_s_at	1.00	0.98	P	665.93	**2.49**	0.00	P	1631.07	FLJ14153	3q25.32
236311_at	0.98	0.89	A	24.20	**2.49**	0.02	P	60.10		
203950_s_at	0.99	0.94	M,A	39.63	**2.49**	0.01	P,M,A	97.17	CLCN6	1p36
215931_s_at	1.00	0.96	P,A	64.70	**2.49**	0.00	P	159.37	ARFGEF2	20q13.13
210115_at	1.00	0.98	P	274.27	**2.49**	0.00	P	672.93	RPL39L	3q27
203884_s_at	1.00	0.99	P	118.00	**2.49**	0.00	P	289.57	Rab11-FIP2	10q26.12
236955_at	0.98	0.92	P,A	16.83	**2.49**	0.01	P	41.47		

203636_at	1.00	0.99	P	228.57	**2.49**	0.00	P	559.73	MID1	xp22
243613_at	0.98	0.91	P	12.80	**2.49**	0.01	P	31.43	MGC24039	12p11.22
228562_at	1.00	0.96	P	157.10	**2.49**	0.01	P	386.20	ZBTB10	8q13-q21.1
202528_at	0.99	0.95	P	284.83	**2.49**	0.00	P	695.93	GALE	1p36-p35
209780_at	1.00	0.97	P	383.23	**2.49**	0.01	P	941.03	PHTF2	7q11.23-q21
220123_at	0.98	0.90	A	17.37	**2.49**	0.02	P,A	43.13	SLC35F5	2q14.1
211051_s_at	0.99	0.93	M,A	55.03	**2.49**	0.01	P	136.50	EXTL3	8p21
224740_at	1.00	0.96	P	812.70	**2.49**	0.00	P	1991.53		
237145_at	0.99	0.91	P,A	28.57	**2.49**	0.01	P	69.67	EIF2AK4	15q14
232415_at	1.00	0.97	A	10.43	**2.49**	0.00	P,A	25.57	PCDHB13	5q31
1553193_at	1.00	0.95	P,A	19.53	**2.49**	0.02	P	48.70	ZNF441	19p13.2
230369_at	1.00	0.96	P	52.70	**2.49**	0.00	P	129.73	GPR161	1q23.3
207439_s_at	0.98	0.91	P	93.33	**2.49**	0.00	P	229.63	SLC35A2	p11.23-p11.22
212839_s_at	0.98	0.89	P	38.40	**2.48**	0.01	P	94.67	SSA2	1q31
223954_x_at	0.98	0.90	P,A	146.50	**2.48**	0.00	P	360.50	APBA2BP	20q11.22
206132_at	1.00	0.98	P,A	136.93	**2.48**	0.01	P	338.43	MCC	5q21-q22
213391_at	0.99	0.92	P	136.23	**2.48**	0.00	P	334.13	LOC286148	8q22.1
204352_at	1.00	0.95	P	85.30	**2.48**	0.01	P	208.67	TRAF5	1q32
1555864_s_at	1.00	0.99	P	1005.97	**2.48**	0.00	P	2456.00	PDHA1	xp22.2-p22.1
243372_at	1.00	0.95	A	58.53	**2.48**	0.00	P,M	142.47	HSPD1	2q33.1
214670_at	1.00	0.96	P	246.00	**2.48**	0.01	P	602.30	ZNF36	7q21.3-q22.1
204841_s_at	0.99	0.94	P	35.40	**2.48**	0.00	P	86.97	EEA1	12q22
222668_at	1.00	0.96	P	142.13	**2.48**	0.00	P	347.67	KCTD15	19q13.12
213836_s_at	0.99	0.94	P	328.30	**2.48**	0.00	P	797.70	FLJ10055	17q24.3
226268_at	0.99	0.92	P,A	63.70	**2.48**	0.00	P	155.13	RAB21	12q15
209003_at	1.00	0.99	P	574.87	**2.48**	0.00	P	1402.33	SLC25A11	17p13.3
212721_at	1.00	0.95	P	317.80	**2.48**	0.01	P	778.00	SFRS12	5q12.3
210868_s_at	0.98	0.91	P	85.23	**2.48**	0.01	P	209.57	ELOVL6	4q25
209466_x_at	0.98	0.89	A	144.20	**2.48**	0.00	P	349.50	PTN	7q33-q34
212196_at	1.00	0.95	P	123.90	**2.48**	0.00	P	302.60		
232592_at	1.00	0.95	A	31.27	**2.47**	0.01	P	76.47		
241478_at	1.00	0.96	P,A	26.73	**2.47**	0.01	P	65.60	FLJ23471	7p22.3
1555736_a_at	1.00	0.96	P	145.43	**2.47**	0.00	P	354.80	AGTRAP	1p36.21
209404_s_at	0.98	0.89	P	309.60	**2.47**	0.01	P	758.60	CGI-109	5q23.1
1554352_s_at	1.00	0.96	A	63.17	**2.47**	0.00	P	154.03	IRLB	15q22.2
226710_at	1.00	0.98	P	325.93	**2.47**	0.00	P	791.93		
222474_s_at	0.99	0.94	P	830.23	**2.47**	0.00	P	2025.60	TOMM22	22q12-q13
224935_at	1.00	0.95	P	1016.80	**2.47**	0.00	P	2467.87	EIF2S3	xp22.2-p22.1
223976_at	1.00	0.95	P,M,A	26.60	**2.47**	0.01	P	65.03	FUT10	8p12
219281_at	1.00	0.98	P	185.53	**2.47**	0.00	P	450.83	MSRA	8p23.1
216381_x_at	1.00	0.97	P,M	153.80	**2.47**	0.00	P	374.80	AKR7A2	p35.1-p36.23
235926_at	0.98	0.90	P	94.43	**2.47**	0.02	P	230.40	ANAPC5	12q24.31
225069_at	0.99	0.93	P	190.17	**2.47**	0.01	P	461.30		
219164_s_at	1.00	0.96	P	56.53	**2.47**	0.00	P	137.07	C14orf103	14q32.31
235725_at	0.99	0.95	P,M	33.57	**2.46**	0.01	P	81.70		
239170_at	0.99	0.92	P	62.07	**2.46**	0.00	P	151.50		
240118_at	0.98	0.91	A	11.80	**2.46**	0.02	P,A	29.07		
225972_at	1.00	0.98	P	54.77	**2.46**	0.00	P	132.33	KFZp762C11	8q21.3
227357_at	1.00	0.96	P	340.13	**2.46**	0.00	P	824.43	TAB3	Xp21.3
230737_s_at	0.99	0.94	P	30.87	**2.46**	0.01	P	74.53	SVIL	10p11.2
203867_s_at	1.00	0.97	A	166.93	**2.46**	0.00	P,M	403.33	FLJ10458	17q21.1
203843_at	1.00	0.95	P,M	185.17	**2.46**	0.00	P	447.23	RPS6KA3	xp22.2-p22.1
241936_x_at	1.00	0.95	A	24.40	**2.46**	0.01	P	59.13		
227636_at	1.00	0.98	P	264.23	**2.46**	0.02	P	642.67	THAP5	7q22.3
227356_at	1.00	0.98	P	94.10	**2.45**	0.00	P	226.97	KIAA0779	3q21.3
211713_x_at	1.00	0.97	P	462.93	**2.45**	0.01	P	1122.50	KIAA0101	15q22.1
228049_x_at	0.99	0.92	P	901.73	**2.45**	0.00	P	2167.80		
1556180_at	1.00	0.95	P	79.20	**2.45**	0.01	P	193.40	LOC255458	5q35.3
223917_s_at	0.99	0.93	P,A	398.23	**2.45**	0.00	P	957.33	SLC39A3	19p13.3
213623_at	0.99	0.92	P	158.53	**2.45**	0.00	P	384.20	KIF3A	5q31
212439_at	1.00	0.98	P,M	172.83	**2.45**	0.00	P	417.10	IHPK1	3p21.31
222685_at	1.00	0.98	P	294.73	**2.45**	0.01	P	713.27	FAM29A	9p22.1
218695_at	0.99	0.93	P	350.87	**2.45**	0.01	P	845.23	EXOSC4	8q24.3
235289_at	0.98	0.91	P,M,A	48.50	**2.45**	0.02	P	117.80	EIF5A2	3q26.2
243452_at	1.00	0.96	A	50.10	**2.45**	0.00	P	120.57	B4GALT6	18q11
225127_at	1.00	0.98	P	298.83	**2.45**	0.01	P	724.50	KIAA1423	6q25.3
213194_at	1.00	0.98	P	377.27	**2.45**	0.00	P	907.63	ROBO1	3p12
1554062_at	0.99	0.94	P,A	18.93	**2.45**	0.02	P	46.03	XG	xp22.33
1552631_a_at	1.00	0.99	A	32.10	**2.45**	0.01	P	77.43	MAP3K6	1p35.3
226524_at	0.99	0.93	P,A	99.73	**2.45**	0.00	P	240.80	MGC26717	3p12.1
219637_at	1.00	0.98	P	92.80	**2.45**	0.01	P	224.20	FLJ12584	2q37.1
225204_at	1.00	0.97	P	346.97	**2.44**	0.00	P	834.33	TA-PP2C	12q24.13
203093_s_at	0.99	0.92	P	230.47	**2.44**	0.00	P	556.33	TIMM44	9p13.3-p13.2
221007_s_at	1.00	0.96	P	146.20	**2.44**	0.01	P	352.90	FIP1L1	4q12
202317_s_at	0.99	0.95	P	202.60	**2.44**	0.01	P	486.93	UBE4B	1p36.3
1558733_at	0.98	0.89	P,M	97.77	**2.44**	0.00	P	236.67	FLJ35036	3q23
209300_s_at	0.99	0.94	P	247.23	**2.44**	0.00	P	594.13	KFZP566B1	12p13.31
202610_s_at	0.98	0.90	P	234.83	**2.44**	0.00	P	566.77	CRSP2	xp11.4-p11.2
239106_at	1.00	0.96	P	170.27	**2.44**	0.01	P	410.13	LOC340591	Xp22.31
236259_at	1.00	0.99	P	201.73	**2.44**	0.01	P	485.90	STK4	20q11.2-q13.2
233946_at	0.99	0.94	P,A	29.00	**2.44**	0.00	P	69.70	SMU-1	9p12
215920_s_at	0.97	0.88	M,A	29.73	**2.43**	0.02	P,A	72.70	LOC283970	16q22.1
204835_at	1.00	0.97	P	228.87	**2.43**	0.00	P	549.50	POLA	xp22.1-p21.3
242838_at	0.99	0.93	P	44.20	**2.43**	0.01	P	105.87	FLJ12748	3q27.3
210574_s_at	1.00	0.98	P	518.40	**2.43**	0.00	P	1240.53	NUDC	1p35-p34
224707_at	1.00	0.95	P	537.63	**2.43**	0.00	P	1289.23	ORF1-FL49	5q31.3
205452_at	0.99	0.94	P	315.10	**2.43**	0.00	P	758.03	PIGB	15q21-q22
236105_at	1.00	0.98	P,M	16.90	**2.43**	0.00	P	40.43		
202943_s_at	0.99	0.93	P	171.07	**2.43**	0.00	P	407.43	NAGA	22q11
233405_at	0.99	0.95	P	20.23	**2.43**	0.03	P	48.90		
215210_s_at	1.00	0.97	P	249.60	**2.43**	0.00	P	598.63	DLST	14q24.3
244498_x_at	0.99	0.92	A	38.17	**2.43**	0.00	P	91.37	FLJ43276	15q25.2
201203_s_at	0.98	0.90	P,M,A	104.33	**2.43**	0.00	P	250.57	RRBP1	20p12
203637_s_at	0.99	0.94	P	285.27	**2.43**	0.00	P	680.57	MID1	xp22
221041_s_at	0.99	0.94	P	284.63	**2.42**	0.00	P	679.23	SLC17A5	6q14-q15
201618_x_at	1.00	0.99	P,A	347.73	**2.42**	0.01	P	829.90	GPAA1	8q24.3
201061_s_at	1.00	0.96	P	363.63	**2.42**	0.00	P	868.40	STOM	9q34.1
242787_at	1.00	0.97	P	146.67	**2.42**	0.00	P	351.00	INCENP	11q12-q13
214695_at	1.00	0.96	P	85.40	**2.42**	0.00	P	203.33	NICE-4	1q22
236700_at	1.00	0.97	P	41.70	**2.42**	0.02	P	100.13	EIF3S8	16p11.2
56919_at	0.99	0.93	P	53.23	**2.42**	0.01	P	127.07	GORASP1	3p22-p21.33
227452_at	1.00	0.97	P	270.33	**2.42**	0.00	P	641.87		17q25.1
1569190_at	1.00	0.97	P,A	53.17	**2.42**	0.00	P	126.63	FLJ30655	4q28.2
212887_at	1.00	0.96	P	415.13	**2.42**	0.01	P	991.97	SEC23A	14q13.3
218465_at	1.00	0.99	P	348.97	**2.42**	0.00	P	828.90	FLJ10525	4p14
210813_s_at	0.99	0.94	P	90.80	**2.41**	0.01	P	216.57	XRCC4	5q13-q14
240344_x_at	1.00	0.99	P	121.73	**2.41**	0.01	P	289.60	LOC90624	5q31.1
241595_at	0.99	0.93	A	26.17	**2.41**	0.01	P,M,A	62.03		
201954_at	1.00	0.97	P	1566.53	**2.41**	0.00	P	3707.50	ARPC1B	7q22.1
218852_at	1.00	1.00	P	205.10	**2.41**	0.00	P	486.23	C14orf10	14q13.2
222979_s_at	0.98	0.91	P	699.83	**2.41**	0.00	P	1671.30	SURF4	9q34.2
201664_at	0.99	0.95	P	927.13	**2.41**	0.00	P	2202.43	SMC4L1	3q26.1
32502_at	0.99	0.94	P	130.87	**2.41**	0.01	P	310.03	PP1665	11q13.3
222625_s_at	0.98	0.90	P	55.23	**2.41**	0.01	P	131.53	NDE1	16p13.11
244661_at	0.99	0.95	P	56.70	**2.41**	0.02	P	135.30		
214989_x_at	0.98	0.91	P	183.97	**2.41**	0.02	P	438.93	PEPP2	12p12
202030_at	1.00	0.97	P	176.77	**2.40**	0.00	P	417.63	BCKDK	16p11.2
229346_at	0.99	0.94	A	14.83	**2.40**	0.03	P,A	35.77	NES	1q23.1
1554915_a_at	0.98	0.90	P	94.50	**2.40**	0.01	P	225.33	KFZp667B12	3p21.2
222679_s_at	0.99	0.93	P	396.73	**2.40**	0.02	P	947.83	RP42	3q26.3
1555465_at	0.99	0.92	P,A	81.13	**2.40**	0.00	P	193.33	MCOLN2	1p22
226893_at	0.99	0.92	P	121.07	**2.40**	0.00	P	287.97	ABL2	1q24-q25
213449_at	1.00	0.97	P,A	84.50	**2.40**	0.00	P	199.57	POP1	8q22.1
216267_s_at	1.00	0.97	P	151.70	**2.40**	0.01	P	362.43	PL6	3p21.3
225780_at	0.99	0.93	P	158.23	**2.40**	0.00	P	374.80	RSC1A1	1p36.1
218399_s_at	1.00	0.98	P	471.03	**2.40**	0.00	P	1110.97	CDCA4	14q32.33
240486_at	0.99	0.92	P,A	41.00	**2.40**	0.03	P	97.57	HELZ	17q24.3
223203_at	0.99	0.93	P	294.13	**2.40**	0.01	P	694.73	PRO0659	xp11.23
210156_s_at	0.99	0.92	P	832.53	**2.40**	0.00	P	1968.97	PCMT1	6q24-q25
228003_at	1.00	0.96	P,A	50.37	**2.39**	0.01	P	119.37	RAB30	11q12-q14
244020_at	1.00	0.98	P,A	24.07	**2.39**	0.02	P	57.17		
1568699_at	0.98	0.90	P,M,A	48.17	**2.39**	0.01	P	114.07	MGC16028	14q24.3
222327_x_at	0.99	0.93	P,A	83.93	**2.39**	0.00	P	196.93	LOC283491	13q21.2
212421_at	1.00	0.96	P	91.47	**2.39**	0.01	P	216.97	KIAA0930	22q13.31

202993_at	1.00	0.98	P	500.67	**2.39**	0.00	P	1178.17	ILVBL	19p13.1
1570566_at	0.99	0.91	P,A	22.13	**2.39**	0.01	P	52.23		
241396_at	1.00	0.99	A	87.33	**2.39**	0.01	P	206.63	NEDD4L	18q21
226939_at	0.99	0.92	P	142.47	**2.39**	0.00	P	336.37	CPEB2	4p15.33
223487_x_at	0.99	0.93	P	172.90	**2.39**	0.01	P	410.33	GNB4	3q27.1
225685_at	1.00	0.96	P	596.90	**2.38**	0.01	P	1401.23		
225738_at	1.00	0.96	P	164.67	**2.38**	0.00	P	388.90	RAPGEF1	9q34.3
201030_x_at	1.00	0.97	P	2376.17	**2.38**	0.00	P	5611.20	LDHB	2p12.2-p12.1
235780_at	1.00	0.98	A	34.80	**2.38**	0.00	P,M	81.67	PRKACB	1p36.1
203621_at	1.00	0.97	P	1192.90	**2.38**	0.00	P	2802.13	NDUFB5	3q27.1
231094_s_at	1.00	0.95	P,A	68.23	**2.38**	0.02	P	161.30	FTHFSDC1	6q25.1
209342_s_at	0.99	0.93	P,A	47.37	**2.38**	0.00	P	111.53	IKBKB	8p11.2
1556597_a_at	1.00	0.95	A	24.70	**2.38**	0.02	P	58.27	LOC28451	1p36.13
239144_at	0.98	0.90	A	15.37	**2.38**	0.01	P,A	36.30	B3GAT2	6q13
241763_s_at	1.00	0.96	P	40.13	**2.38**	0.01	P	94.33	FBXO32	8q24.13
212214_at	0.99	0.94	P,A	270.93	**2.38**	0.00	P	637.37	OPA1	3q28-q29
239842_x_at	0.99	0.94	P,A	31.13	**2.38**	0.00	P	72.70		
212682_s_at	0.98	0.91	A	170.20	**2.38**	0.02	P	405.63	BC002942	22q13.33
201512_s_at	0.99	0.94	P	514.07	**2.38**	0.00	P	1207.13	TOMM70A	3q12.3
203974_at	0.99	0.91	P	173.60	**2.38**	0.00	P	407.47	FAM16AX	xp22.32
224149_x_at	0.98	0.90	P	82.13	**2.38**	0.01	P	194.43	SLMAP	3p21.2-p14.3
224151_s_at	0.99	0.93	P	126.03	**2.38**	0.00	P	295.70	AK3L1	9p24.1-p24.3
202550_s_at	1.00	0.95	P	436.33	**2.38**	0.00	P	1021.77	VAPB	20q13
1554334_a_at	0.99	0.94	P,A	17.57	**2.38**	0.01	P	41.17	DNAJA4	15q24.1
218866_s_at	0.99	0.93	P	453.93	**2.38**	0.00	P	1064.13	POLR3K	16p13.3
226190_at	1.00	0.98	P	823.20	**2.38**	0.00	P	1926.73		
223290_at	0.99	0.95	P,M	282.97	**2.38**	0.00	P	663.17	PDXP	22cen-q12.3
200883_at	0.99	0.92	P	776.43	**2.37**	0.00	P	1822.43	UQCRC2	16p12
217595_at	1.00	0.97	P,A	31.53	**2.37**	0.01	P	74.73	GSPT1	16p13.1
238519_at	1.00	0.96	P	84.90	**2.37**	0.00	P	198.47		
210793_s_at	1.00	0.97	P	105.73	**2.37**	0.00	P	246.70	NUP98	11p15.5
234132_at	0.99	0.94	A	35.70	**2.36**	0.00	P,A	83.40		
228638_at	0.99	0.93	P	107.30	**2.36**	0.00	P	250.67	MGC34648	1p35.3
224693_at	0.99	0.93	P	325.40	**2.36**	0.00	P	757.90	C20orf108	20q13.31
231793_s_at	0.99	0.94	P	65.50	**2.36**	0.00	P	152.53	CAMK2D	4q26
221550_at	0.99	0.95	P	99.00	**2.36**	0.01	P	232.13	COX15	10q24
1555041_a_at	0.99	0.95	A	234.50	**2.36**	0.02	P	549.50	NAGA	22q11
202394_s_at	1.00	0.98	P	129.83	**2.36**	0.00	P	301.23	ABCF3	3q27.3
201976_s_at	1.00	0.96	P	781.17	**2.36**	0.00	P	1816.97	MYO10	5p15.1-p14.3
221895_at	0.98	0.90	A	103.67	**2.36**	0.01	P,M,A	242.90	MOSPD2	xp22.31
239300_at	0.99	0.94	P	17.70	**2.35**	0.01	P	41.23		
227138_at	1.00	0.98	P	112.97	**2.35**	0.00	P	261.90	LOC253263	3p22.3
244350_at	1.00	0.96	P	58.97	**2.35**	0.02	P	137.60		
228713_s_at	0.99	0.95	P,A	57.67	**2.35**	0.01	P	134.30	DHRS10	19q13.33
226888_at	0.99	0.94	P	85.50	**2.35**	0.01	P	197.63	CSNK1G1	5q22.1-q22.31
224622_at	1.00	0.97	P	500.10	**2.35**	0.00	P	1156.30	TBC1D14	4p16.1
200737_at	0.99	0.93	P	1257.53	**2.35**	0.00	P	2912.50	PGK1	xq13
225341_at	0.99	0.91	A	43.13	**2.35**	0.00	P	100.40	LOC80298	12q24.1
238879_at	1.00	0.96	P	82.80	**2.35**	0.00	P	191.37	RP42	3q26.3
225614_at	1.00	0.98	P	536.03	**2.34**	0.00	P	1237.27	LOC113174	11p15.1
1568596_a_at	1.00	0.95	A	143.90	**2.34**	0.01	P	333.57	TROAP	12q13.12
230061_at	0.99	0.91	A	4.83	**2.34**	0.01	P,A	11.33	LOC116441	3q24
236834_at	1.00	0.97	P	95.80	**2.34**	0.00	P	220.70	SCFD2	4q12
216870_x_at	0.98	0.91	P	142.67	**2.34**	0.00	P	330.37	DLEU2	13q14.3
57703_at	0.98	0.90	P	58.43	**2.34**	0.01	P	135.40	SENP5	3q29
225663_at	1.00	0.97	P	405.40	**2.34**	0.00	P	934.53	ACBD5	10p12.1
204593_s_at	0.99	0.92	P	421.87	**2.34**	0.00	P	977.87	FLJ20232	22q13
225710_at	1.00	0.99	P	781.90	**2.34**	0.00	P	1799.57		
227847_at	1.00	0.99	P	183.80	**2.34**	0.00	P	423.93	EPM2AIP1	3p22.1
201637_s_at	1.00	0.99	P	534.93	**2.34**	0.00	P	1229.83	FXR1	3q28
210042_s_at	0.99	0.95	P	72.97	**2.34**	0.01	P	169.10	CTSZ	20q13
229115_at	1.00	0.99	A	239.10	**2.34**	0.01	P,M,A	552.23	DNCH1	14q32
207495_at	1.00	0.95	P,A	29.30	**2.34**	0.01	P	67.87	RAB28	4p16.1
201155_s_at	0.99	0.93	P	402.93	**2.34**	0.00	P	923.03	MFN2	1p36.21
202168_at	0.99	0.94	P	647.93	**2.34**	0.00	P	1483.77	TAF9	5q11.2-q13.1
209231_s_at	0.98	0.91	P	156.73	**2.34**	0.01	P	366.77	MGC3248	16p12.3
203171_s_at	0.99	0.92	P,A	102.13	**2.34**	0.00	P	235.63	KIAA0409	11p15.4
210754_s_at	0.99	0.93	P	90.50	**2.34**	0.00	P	208.97	LYN	8q13
238731_at	1.00	0.97	P,A	16.90	**2.33**	0.02	P	39.17	SETDB2	13q14
218574_s_at	1.00	0.98	P	69.90	**2.33**	0.00	P	160.47	LMCD1	3p26-p24
216170_at	0.99	0.94	P	27.47	**2.33**	0.03	P	64.10		
212895_s_at	0.99	0.95	P	279.60	**2.33**	0.00	P	640.73	ABR	17p13.3
202235_at	0.98	0.89	A	25.50	**2.33**	0.01	P,A	59.17	SLC16A1	1p12
201336_at	1.00	1.00	P	627.07	**2.33**	0.00	P	1439.37	VAMP3	1p36.23
200929_at	0.99	0.92	P	820.43	**2.33**	0.00	P	1894.67	TMP21	14q24.3
201378_s_at	1.00	0.99	P	487.10	**2.33**	0.00	P	1121.97	NICE-4	1q22
228433_at	1.00	0.96	P	95.10	**2.33**	0.00	P	218.60	FLJ11236	
219361_s_at	0.99	0.92	P,M,A	109.07	**2.33**	0.02	P	251.63	FLJ12484	15q25.3
227862_at	0.99	0.93	P	58.53	**2.33**	0.01	P	134.83		1p35.3
218493_at	1.00	0.98	P	555.00	**2.33**	0.00	P	1272.67	C16orf33	16p13.3
218389_s_at	0.98	0.91	P	425.90	**2.33**	0.01	P	984.50	APH-1A	1p36.13-q31.3
223963_s_at	1.00	0.97	A	53.17	**2.33**	0.00	P	121.93	IMP-2	3q28
218962_s_at	1.00	0.95	P	264.07	**2.33**	0.00	P	606.37	FLJ13576	7q31.32
202344_at	0.99	0.95	P,A	133.40	**2.33**	0.00	P	305.93	HSF1	8q24.3
202246_s_at	1.00	0.98	P	1042.00	**2.33**	0.00	P	2392.57	CDK4	12q14
243509_at	1.00	0.98	P,M,A	45.17	**2.33**	0.01	P	103.70		
217367_s_at	1.00	0.97	M,A	54.47	**2.33**	0.01	P	125.07	ZHX3	20q12
223517_at	0.99	0.93	A	43.53	**2.33**	0.01	P	99.43	FBX30	1p36.21
227954_at	0.99	0.94	P	105.57	**2.33**	0.01	P	243.73	LOC162073	16p13.11
201280_s_at	0.99	0.95	P	129.13	**2.33**	0.01	P	295.07	DAB2	5p13
225756_at	0.99	0.94	P	287.07	**2.32**	0.00	P	658.17	CSNK1E	22q13.1
226484_at	1.00	0.99	P,A	70.77	**2.32**	0.00	P	161.60	KIAA1190	3p21.33
218430_s_at	0.98	0.90	P	42.80	**2.32**	0.01	P	98.40	FLJ12994	15q21.2
213263_s_at	1.00	0.98	A	94.83	**2.32**	0.00	P	216.53	PCBP2	12q13.12-q13.13
222477_s_at	1.00	0.97	P	458.97	**2.32**	0.00	P	1048.23	TM7SF3	12q11-q12
222113_s_at	0.99	0.93	P	131.83	**2.32**	0.00	P	300.33	EPS15L1	19p13.12
225922_at	0.99	0.94	P	242.80	**2.32**	0.00	P	555.77	KIAA1450	4q32.1
202279_at	1.00	0.96	P	795.67	**2.32**	0.00	P	1810.20	C14orf2	14q32.33
214260_at	1.00	0.95	P	102.00	**2.32**	0.00	P	233.10	COPS8	2q37.3
222555_s_at	0.98	0.91	P	376.17	**2.32**	0.00	P	865.90	MRPL44	2q36.3
237622_at	1.00	0.98	A	38.93	**2.32**	0.00	P	89.03		
235588_at	0.99	0.93	P,M,A	87.00	**2.32**	0.01	P	197.90	LOC157570	8p21.1
214306_at	1.00	0.95	P	133.60	**2.31**	0.01	P	304.60	OPA1	3q28-q29
226901_at	0.99	0.94	P	220.50	**2.31**	0.01	P	503.80	LOC284018	17q24.3
207029_at	0.98	0.91	P,A	16.47	**2.31**	0.02	P	38.40	KITLG	12q22
1559942_at	0.99	0.92	P	152.83	**2.31**	0.01	P	346.87	HIC	7q31.2
230653_at	1.00	0.97	P	76.77	**2.31**	0.02	P	176.37		
212248_at	1.00	0.97	P	349.30	**2.31**	0.01	P	797.97	LYRIC	8q22.1
219982_s_at	1.00	0.97	P,A	30.47	**2.31**	0.01	P,A	69.47	SERF1A	5q12.2-q13.3
209093_s_at	1.00	0.96	P	232.63	**2.31**	0.00	P	529.60	GBA	1q21
211930_at	0.99	0.93	P	298.87	**2.31**	0.02	P	685.37	hnRNPA3	2q31.2
241687_at	1.00	0.97	P	63.63	**2.31**	0.01	P	144.77		
218188_s_at	1.00	0.98	P	578.47	**2.31**	0.01	P	1312.50	TIMM13	19p13.3
1557593_at	0.99	0.94	P,A	12.13	**2.31**	0.00	P	27.53	PF6	1p12
211060_x_at	1.00	0.98	P	392.53	**2.31**	0.00	P	889.73	GPAA1	8q24.3
238781_at	0.98	0.90	P,M	54.50	**2.31**	0.01	P	124.40	SFRS12	5q12.3
221436_s_at	1.00	0.95	P	362.93	**2.30**	0.02	P	833.47	CDCA3	12p13
221004_s_at	1.00	0.97	P,M	233.50	**2.30**	0.00	P	529.87	ITM2C	2q37
202047_s_at	0.99	0.95	P,A	373.37	**2.30**	0.00	P	844.20	CBX6	22q13.1
235653_s_at	0.99	0.93	P,A	35.73	**2.30**	0.00	P	81.17	THAP6	4q21.21
1557113_at	1.00	1.00	M,A	22.80	**2.30**	0.02	P,M	52.07	LOC283588	14q32.12
228956_at	0.99	0.93	P	188.07	**2.30**	0.00	P	427.03	UGT8	4q26
211865_s_at	1.00	0.97	P,A	54.33	**2.30**	0.00	P	123.00	FZR1	19p13.3
242705_x_at	0.98	0.91	P	130.03	**2.30**	0.01	P	294.50	LRPAP1	4p16.3
203767_s_at	1.00	0.98	P	78.80	**2.30**	0.02	P	179.77	STS	xp22.32
218858_at	1.00	0.95	P	85.67	**2.30**	0.00	P	194.10	DEPDC6	8q24.12
225538_at	0.99	0.92	P	332.13	**2.30**	0.00	P	757.43	ZCCHC9	5q14.1
230098_at	0.99	0.95	P	118.27	**2.30**	0.00	P	267.30		
1569253_at	0.99	0.92	P	34.60	**2.30**	0.01	P	78.83	MGC16733	11q13.4
234985_at	1.00	0.96	P	533.57	**2.30**	0.01	P	1210.87	LOC143458	11p13
221771_s_at	1.00	0.96	P	164.73	**2.30**	0.00	P	373.30	HSMPP8	13q12.11
222518_at	1.00	0.96	P	254.73	**2.30**	0.00	P	577.10	ARFGEF2	20q13.13
214631_at	1.00	0.95	A	11.37	**2.30**	0.02	P	26.03	ZBTB33	xq23
201018_at	0.99	0.94	P	388.60	**2.30**	0.00	P	880.87	EIF1AX	xp22.13

203230_at	1.00	0.95	P	136.43	**2.30**	0.00	P	308.53	DVL1	1p36
218264_at	0.98	0.90	P	169.53	**2.30**	0.01	P	387.43	BCCIP	10q26.1
242486_at	0.99	0.93	P	90.17	**2.29**	0.00	P	203.33		
207234_at	0.99	0.95	P	22.43	**2.29**	0.01	P	51.13	RFX3	9p24.2
237400_at	1.00	0.98	P	34.40	**2.29**	0.01	P	78.30	ATP5S	14q22.1
1557429_s_at	0.98	0.91	A	5.23	**2.29**	0.03	P,A	12.07	FLJ32800	15q15.3
241418_at	1.00	0.99	M,A	41.80	**2.29**	0.01	P	95.27		
210720_s_at	1.00	0.97	P	200.93	**2.29**	0.00	P	453.03	APBA2BP	20q11.22
225657_at	1.00	0.99	P	375.30	**2.29**	0.00	P	845.50	LOC152217	3q29
225612_s_at	0.99	0.95	P	217.13	**2.29**	0.01	P	490.87	B3GNT5	3q28
244674_at	0.99	0.94	A	42.47	**2.29**	0.01	P	95.77		
200782_at	0.99	0.93	P	2074.07	**2.29**	0.00	P	4688.57	ANXA5	4q28-q32
216352_x_at	0.99	0.92	A	39.77	**2.29**	0.01	P	89.87	PCDHGA3	5q31
226637_at	0.99	0.94	P,A	35.27	**2.29**	0.01	P	79.93	UBE2H	7q32
226249_at	1.00	0.98	P	221.07	**2.29**	0.01	P	498.03		9q33.1
204956_at	1.00	0.96	P	78.40	**2.28**	0.02	P	177.20	MTAP	9p21
226432_at	0.99	0.95	P	129.03	**2.28**	0.00	P	290.40		
203897_at	1.00	0.96	P	115.90	**2.28**	0.02	P	263.07	LOC57149	16p11.2
210792_x_at	0.98	0.90	P	450.67	**2.28**	0.00	P	1015.30	SIVA	14q32.33
232652_x_at	0.99	0.95	P	305.27	**2.28**	0.01	P	683.77	SCAND1	20q11.1-q11.23
212431_at	1.00	0.97	P,A	148.83	**2.28**	0.00	P	335.43	KIAA0194	5q33.1
242407_at	0.99	0.94	P,M,A	33.63	**2.28**	0.02	P	76.20		
223360_at	1.00	0.96	P	105.83	**2.28**	0.00	P	237.50	C21orf56	21q22.3
219972_s_at	0.98	0.91	P	111.30	**2.28**	0.01	P	250.80	C14orf135	14q23.1
238822_at	0.99	0.93	P	58.30	**2.28**	0.01	P	130.83		
235007_at	1.00	0.95	P	42.90	**2.28**	0.02	P	96.60	BBS7	4q27
219736_at	0.99	0.92	P	63.17	**2.28**	0.02	P	142.87	TRIM36	5q23.1
203340_s_at	0.99	0.93	P	211.23	**2.28**	0.00	P	475.00	SLC25A12	2q24
223332_x_at	0.99	0.95	P	138.07	**2.27**	0.01	P	310.27	RNF126	19p13.3
227178_at	1.00	0.97	P,A	74.07	**2.27**	0.00	P	166.07	CUGBP2	10p13
219348_at	1.00	0.96	P	83.03	**2.27**	0.00	P	186.10	MDS032	19p13.12
209120_at	1.00	0.96	P	196.03	**2.27**	0.02	P	443.23	NR2F2	15q26
224605_at	0.98	0.90	P	169.77	**2.27**	0.01	P	380.03		4q27
206302_s_at	1.00	0.96	P	261.90	**2.27**	0.00	P	586.63	NUDT4	12q21
228304_at	0.99	0.94	P,A	34.13	**2.27**	0.01	P	76.43		
225276_at	1.00	0.98	P,A	198.97	**2.27**	0.00	P	444.30	GSPT1	16p13.1
212263_at	1.00	0.98	P	302.53	**2.27**	0.01	P	676.43	QKI	6q26-27
1553559_at	0.99	0.93	A	60.03	**2.27**	0.01	P	136.20	LOC134285	5q13.3
224875_at	0.99	0.92	P	331.37	**2.27**	0.02	P	750.43	FLJ37562	5q31.2
204074_s_at	0.99	0.94	P,A	90.20	**2.27**	0.00	P	201.37	KIAA0562	1p36.32
205545_x_at	1.00	0.97	P	232.67	**2.27**	0.00	P	518.07	DNAJC8	1p35.2
222624_s_at	1.00	0.96	P	100.50	**2.27**	0.01	P	225.63	ANC_2H01	3q27.1
221537_at	1.00	0.95	P	128.47	**2.27**	0.01	P	288.03	KFZp564A1	3q21.3
209528_s_at	1.00	0.95	P	67.30	**2.27**	0.01	P	150.50	KIAA0683	16p13.3
242984_at	0.98	0.90	P,M,A	28.23	**2.27**	0.02	P	64.60	MKLN1	7q32
221787_at	1.00	0.96	P	112.67	**2.27**	0.01	P	253.07	PHF10	6q27
212241_at	0.99	0.94	P	159.70	**2.27**	0.00	P	357.20	GRINL1A	15q22.1
201611_s_at	1.00	0.96	P	167.27	**2.26**	0.00	P	373.50	ICMT	1p36.21
239719_at	0.99	0.94	P	154.97	**2.26**	0.00	P	344.50	CD109	6q14.1
208877_at	1.00	0.96	P	476.90	**2.26**	0.00	P	1066.37	PAK2	3q29
52164_at	1.00	0.95	P	736.47	**2.26**	0.01	P	1656.27	C11orf24	11q13
219819_s_at	0.99	0.94	P	473.40	**2.26**	0.00	P	1056.30	MRPS28	8q21.1-q21.2
201661_s_at	0.99	0.95	P	379.47	**2.26**	0.00	P	846.30	ACSL3	2q34-q35
226478_at	0.98	0.91	P	80.63	**2.26**	0.01	P	180.77		
1558797_at	1.00	0.97	P,A	12.63	**2.26**	0.01	P	28.27		
223355_at	0.98	0.89	M,A	97.10	**2.26**	0.02	P	218.57	ALG1	16p13.3
229876_at	0.99	0.93	A	48.37	**2.26**	0.00	P,A	107.67	PHKA1	Xq12-q13
223138_s_at	0.99	0.94	P	352.53	**2.26**	0.02	P	791.23	DHX36	3p13-q23
205298_s_at	1.00	0.96	P	230.53	**2.26**	0.01	P	514.17	BTN2A2	6p22.1
219628_at	0.98	0.91	P	163.87	**2.26**	0.00	P	362.47	WIG1	3q26.3-q27
212294_at	1.00	0.95	P	1089.80	**2.26**	0.00	P	2429.43	GNG12	1p31.2
210465_s_at	0.99	0.94	P,A	127.53	**2.26**	0.00	P	285.43	SNAPC3	9p22.2
218195_at	0.99	0.94	P	438.90	**2.26**	0.00	P	978.00	C6orf211	6q25.1
228990_at	1.00	0.96	P	112.57	**2.26**	0.01	P	250.23	LOC85028	1p35.2
211067_s_at	1.00	0.98	M,A	149.23	**2.26**	0.00	P	332.00	GAS7	17p13.1
215690_x_at	0.99	0.95	P,A	432.47	**2.26**	0.01	P	964.47	GPAA1	8q24.3
222649_at	0.99	0.95	P	158.70	**2.26**	0.00	P	352.20	XPO4	13q11
227063_at	1.00	0.98	P	335.63	**2.26**	0.00	P	744.40	MGC40107	17p13.2
214241_at	1.00	0.99	P	76.10	**2.26**	0.01	P	169.23	NDUFB8	10q23.2-q23.33
219265_at	0.98	0.90	A	101.57	**2.25**	0.01	P	226.87	MOBKL2B	9p21.1
1554780_a_at	1.00	0.96	P	100.20	**2.25**	0.00	P	222.80	PHTF2	7q11.23-q21
218817_at	0.99	0.95	P	68.67	**2.25**	0.00	P	153.23	FLJ22649	4q34.2
212525_s_at	0.99	0.94	P,M	191.60	**2.25**	0.01	P	429.47	H2AFX	11q23.2-q23.3
1564064_a_at	1.00	0.99	P,A	125.20	**2.25**	0.00	P	277.67	ATP11B	3q27
242057_at	0.99	0.92	P	18.27	**2.25**	0.02	P	40.80		
213861_s_at	0.99	0.93	P	132.00	**2.25**	0.00	P	294.47	KFZP586D0	12q13.2
229742_at	1.00	0.98	P	444.27	**2.25**	0.00	P	984.30		15q22.31
226358_at	1.00	0.97	A	39.53	**2.25**	0.00	P	87.80	LOC145842	15q22.1
204141_at	1.00	0.97	P	971.70	**2.25**	0.00	P	2146.73	TUBB	6p25
242470_at	0.99	0.94	P	26.67	**2.25**	0.02	P	59.47	FLJ38944	19q13.2
226041_at	1.00	0.98	P	123.47	**2.25**	0.00	P	273.53	NAPE-PLD	7q22.1
221808_at	1.00	0.97	P	449.27	**2.25**	0.00	P	995.13	RAB9A	xp22.2
207719_x_at	1.00	0.98	P	512.07	**2.25**	0.00	P	1133.40	KAB	1q44
228710_at	1.00	0.99	P	159.73	**2.25**	0.01	P	354.03		
222977_at	1.00	0.97	P	602.37	**2.25**	0.00	P	1335.27	SURF4	9q34.2
1552303_a_at	1.00	0.96	P	32.40	**2.25**	0.00	P	71.47	MGC20235	17q21.31
203482_at	1.00	0.99	P	174.50	**2.25**	0.01	P	386.73	C10orf6	10q24.32
238034_at	1.00	0.95	P	153.60	**2.25**	0.01	P	339.67	CANX	5q35
226234_at	0.99	0.92	P,M	62.87	**2.25**	0.01	P	138.67	GDF11	12q13.13
227406_at	0.99	0.93	P	180.93	**2.25**	0.00	P	398.27	GABPB2	15q21.2
230758_at	0.99	0.94	P	34.20	**2.24**	0.01	P	75.70		
239721_at	1.00	0.96	P	76.20	**2.24**	0.03	P	170.17		
209306_s_at	1.00	0.97	P	626.53	**2.24**	0.00	P	1384.37	SWAP70	11p15
229072_at	1.00	0.97	A	32.17	**2.24**	0.00	P	71.03		
201248_s_at	0.99	0.95	P	283.73	**2.24**	0.00	P	627.97	SREBF2	22q13
230435_at	0.99	0.94	A	38.70	**2.24**	0.01	P,M,A	85.63	FLJ30851	2p24.1
205362_s_at	0.99	0.93	P	71.70	**2.24**	0.01	P	159.37	PFDN4	20q13
221045_s_at	0.99	0.93	P	230.50	**2.24**	0.00	P	509.80	PER3	1p36.23
222672_at	0.99	0.92	P,A	49.30	**2.24**	0.02	P	109.50	C6orf149	6p25.1
201918_at	0.99	0.92	P	386.33	**2.24**	0.00	P	853.73	FLJ10618	3q23
227787_s_at	1.00	0.96	P	136.87	**2.24**	0.00	P	302.10	THRAP6	8q24.11
1562648_at	0.99	0.94	P,A	20.93	**2.24**	0.02	P	46.33	LOC55580	2p16.3
242755_at	1.00	0.95	P,A	10.23	**2.24**	0.00	P	22.57		
223396_at	1.00	0.99	P	397.40	**2.24**	0.00	P	874.57	C7orf35	7q21.11
218147_s_at	1.00	0.96	P	247.17	**2.24**	0.01	P	546.40	AD-017	3p21.31
229329_s_at	0.98	0.90	P,A	27.77	**2.23**	0.03	P,A	62.23	HSPC182	1p36.33
224890_s_at	1.00	0.95	P	376.43	**2.23**	0.01	P	829.00		7
222465_at	1.00	0.99	P	1015.23	**2.23**	0.01	P	2234.10	C15orf15	15q21
227005_at	1.00	0.95	P	245.43	**2.23**	0.00	P	540.27	RPP14	3p21.2
227108_at	0.99	0.94	P	20.57	**2.23**	0.02	P	45.40	STARD9	15q14
219204_s_at	0.99	0.93	P	45.50	**2.23**	0.02	P	100.90	SRR	17p13
217974_at	0.98	0.89	P	69.77	**2.23**	0.01	P	152.93	TM7SF3	12q11-q12
218701_at	0.99	0.95	P	154.67	**2.23**	0.00	P	340.00	LACTB2	8p22-q22.3
226354_at	0.99	0.94	P	226.13	**2.23**	0.01	P	496.10	LACTB	15q22.1
230032_at	1.00	0.98	P	170.00	**2.23**	0.00	P	371.77	OSGEPL1	2q32.3
40273_at	0.99	0.93	P,M,A	152.87	**2.23**	0.00	P	334.30	SPHK2	19q13.2
201836_s_at	0.99	0.93	P,A	53.50	**2.22**	0.01	P	117.63	TAF65(gamm	2pter-p25.1
223015_at	1.00	0.98	P	884.73	**2.22**	0.00	P	1938.13	eIF2A	3q25.1
227861_at	1.00	0.97	P,A	576.27	**2.22**	0.00	P	1262.33	MGC33214	5q14.3
203746_s_at	1.00	0.97	P	444.53	**2.22**	0.00	P	973.80	HCCS	xp22.3
210062_s_at	0.99	0.94	P,A	61.23	**2.22**	0.00	P	134.47	ZNF589	3p21
218299_at	1.00	0.97	P,A	286.50	**2.22**	0.00	P	628.50	C11orf24	11q13
228972_at	1.00	0.99	P	153.70	**2.22**	0.01	P	336.27		
210710_at	0.99	0.93	P,A	39.10	**2.22**	0.01	P	85.70	VG5Q	5q14.1
217122_s_at	1.00	0.96	P	168.00	**2.22**	0.00	P	367.80	SLC35E2	1p36.33
1570061_at	0.99	0.94	P	37.07	**2.22**	0.00	P	81.13		
204262_s_at	1.00	0.97	P	87.87	**2.22**	0.01	P	192.60	PSEN2	1q31-q42
241879_at	1.00	0.95	P	311.37	**2.22**	0.01	P	683.83		
221030_s_at	1.00	0.95	P,A	33.27	**2.22**	0.03	P	74.30	ARHGAP24	4q22.1
225088_at	0.99	0.91	P	117.13	**2.22**	0.01	P	257.63	FLJ31153	p13.13-p13.12
227481_at	1.00	0.97	P	73.00	**2.22**	0.02	P	161.17	MAGI1	6q25.2
208625_s_at	0.99	0.95	P	1122.57	**2.22**	0.00	P	2457.40	EIF4G1	3q27-qter
48031_r_at	1.00	0.96	M,A	34.33	**2.22**	0.02	P	75.90	C5orf4	5q31-q32
211137_s_at	1.00	0.95	P	204.50	**2.22**	0.01	P	446.13	ATP2C1	3q22.1

215411_s_at	0.98	0.90	P	291.77	**2.22**	0.00	P	633.47	C6orf4	6q21
230265_at	0.98	0.91	P	62.10	**2.22**	0.00	P	136.57	SEL1L	14q24.3-q31
218771_at	0.99	0.94	P	166.77	**2.22**	0.00	P	364.83	PANK4	1p36.32
225063_at	0.99	0.94	P	284.97	**2.22**	0.01	P	629.00	BMSC-UbP	15q23
206194_at	0.99	0.93	P	117.77	**2.22**	0.00	P	257.50	HOXC4	12q13.3
212445_s_at	1.00	0.98	P	203.03	**2.22**	0.00	P	441.97	NEDD4L	18q21
235706_at	0.98	0.90	P	91.17	**2.22**	0.03	P	202.43	CPM	12q14.3
205659_at	1.00	0.95	P	132.00	**2.22**	0.00	P	288.43	HDAC9	7p21.1
203774_at	1.00	0.96	P	230.37	**2.22**	0.01	P	504.83	MTR	1q43
231953_at	0.99	0.92	P	47.20	**2.21**	0.00	P	102.50	FALZ	17q24.3
222845_x_at	1.00	0.97	P	1053.40	**2.21**	0.00	P	2298.30	CGI-119	12q14.1-q15
229333_at	1.00	0.99	P	167.77	**2.21**	0.00	P	365.67		
1554057_at	1.00	0.98	P	121.93	**2.21**	0.01	P	267.10		
218593_at	1.00	0.98	P	430.07	**2.21**	0.00	P	935.93	FLJ10377	7q32.2
221014_s_at	0.99	0.95	P	49.63	**2.21**	0.01	P	108.50	RAB33B	4q28
226003_at	1.00	0.99	P	223.87	**2.21**	0.01	P	488.43	KIF21A	12q12
224591_at	1.00	0.98	P	747.47	**2.21**	0.01	P	1626.90	HP1-BP74	1p36.12
223991_s_at	0.99	0.94	P	104.00	**2.21**	0.00	P	226.80	GALNT2	1q41-q42
217902_s_at	1.00	0.99	P	137.47	**2.21**	0.00	P	299.10	HERC2	15q13
201727_s_at	1.00	0.96	P,A	256.87	**2.21**	0.00	P	562.77	ELAVL1	19p13.2
225099_at	1.00	0.97	P	379.77	**2.21**	0.01	P	827.67	LOC200933	3q29
212491_s_at	0.99	0.94	P,M	127.87	**2.21**	0.00	P	278.80	DNAJC8	1p35.2
221512_at	1.00	1.00	A	185.20	**2.21**	0.00	P	402.47	DKFZP564D0	1p35.3
204137_at	0.99	0.93	P,M	129.30	**2.21**	0.00	P	281.87	TM7SF1	1q42-q43
217716_s_at	1.00	0.95	P	1074.57	**2.21**	0.00	P	2338.20	SEC61A1	3q21.3
229450_at	0.99	0.95	P	106.47	**2.21**	0.00	P	231.70	IFIT4	10q24
215096_s_at	1.00	0.97	P	615.93	**2.20**	0.01	P	1335.20	ESD	3q14.1-q14.2
201733_at	0.99	0.94	P	141.63	**2.20**	0.02	P	308.30	CLCN3	4q33
203730_s_at	0.99	0.93	P	108.13	**2.20**	0.00	P	235.63	ZFP95	7q22
227445_at	0.99	0.92	P,A	90.27	**2.20**	0.00	P	196.83	LOC115509	16p11.2
212172_at	0.98	0.91	P	50.07	**2.20**	0.02	P	108.67	AK2	1p34
212723_at	0.99	0.93	P	342.70	**2.20**	0.00	P	741.37	PTDSR	17q25
207606_s_at	1.00	0.97	P	152.63	**2.20**	0.01	P	331.43	ARHGAP12	10q11.1
229511_at	0.98	0.91	P	56.37	**2.20**	0.02	P	124.00	SMARCE1	17q21.2
200734_s_at	1.00	0.96	P,A	373.83	**2.20**	0.00	P	811.87	ARF3	12q13
219206_x_at	1.00	0.99	P	652.83	**2.20**	0.00	P	1415.23	CGI-119	12q14.1-q15
222411_s_at	1.00	0.98	P	1232.00	**2.20**	0.00	P	2670.67	SSR3	3q25.31
202930_s_at	0.99	0.93	P	362.13	**2.20**	0.00	P	786.40	SUCLA2	3q12.2-q13.3
218754_at	1.00	0.98	P	237.57	**2.20**	0.01	P	515.50	FLJ23323	1p36.23
220750_s_at	1.00	0.97	P	130.77	**2.20**	0.00	P	283.90	LEPRE1	1p34.1
64883_at	0.99	0.94	P	130.93	**2.20**	0.00	P	282.97	MOSPD2	xp22.31
243367_at	0.98	0.90	P,A	37.30	**2.20**	0.02	P	81.67		
209009_at	1.00	0.98	P	750.70	**2.20**	0.00	P	1624.17	ESD	3q14.1-q14.2
209288_s_at	0.99	0.94	P	417.63	**2.20**	0.01	P	906.07	CDC42EP3	2p21
208734_x_at	0.99	0.93	P	671.23	**2.19**	0.01	P	1454.93	RAB2	8q12.1
209172_s_at	1.00	0.99	P	261.17	**2.19**	0.02	P	567.17	CENPF	1q32-q41
222705_s_at	0.99	0.93	A	66.57	**2.19**	0.01	P	144.20	SLC25A15	13q14
244193_at	1.00	0.98	P	78.17	**2.19**	0.00	P	169.07	FLJ13236	12q13.12
212002_at	1.00	0.96	P,M	220.73	**2.19**	0.00	P	476.63	DKFZp566C0	1p36.13
203449_s_at	1.00	1.00	P	180.03	**2.19**	0.01	P	388.90	TERF1	8q13
201581_at	1.00	0.96	P	524.23	**2.19**	0.00	P	1132.30	DJ971N18.2	20p12
225293_at	0.99	0.92	A	53.27	**2.19**	0.01	P	114.50	COL27A1	9q33.1
225074_at	0.99	0.92	P	165.67	**2.19**	0.00	P	359.27	RAB2B	14q11.2
225162_at	0.99	0.94	P	321.03	**2.19**	0.00	P	694.93	DKFZp434D02	4q31.23
1565939_at	0.99	0.91	P,A	19.73	**2.19**	0.00	P	42.73	FLJ11193	5p13.3
223892_s_at	1.00	0.97	P	989.47	**2.19**	0.00	P	2135.67	CGI-119	12q14.1-q15
213554_s_at	0.99	0.95	P	248.27	**2.19**	0.00	P	535.73	H41	3q22.1
212251_at	0.99	0.94	P	625.43	**2.19**	0.01	P	1348.50	LYRIC	8q22.1
213394_at	0.98	0.90	A	157.33	**2.19**	0.03	P,M,A	340.73	KLHL3	5q31
209239_at	1.00	0.99	P	303.13	**2.19**	0.00	P	653.87	NFKB1	4q24
242928_at	0.99	0.94	P	20.47	**2.19**	0.02	P	44.37		
232565_at	0.98	0.90	A	29.47	**2.19**	0.01	P	63.67		
1555024_at	1.00	0.97	P,A	6.37	**2.18**	0.01	P,A	13.77	ADAM22	7q21
1559268_at	0.99	0.94	A	38.83	**2.18**	0.01	P	84.60	ADAM23	2q33
207428_x_at	1.00	0.95	P,A	118.50	**2.18**	0.01	P	256.70	CDC2L1	1p36
225024_at	0.99	0.95	P	285.70	**2.18**	0.00	P	615.17	C20orf77	20q11.21-q12
235260_s_at	0.99	0.93	P	150.00	**2.18**	0.00	P	323.17	MGC29898	4p15.32
1557062_at	0.98	0.91	P	27.23	**2.18**	0.02	P	58.37		
201001_s_at	0.99	0.94	P	502.30	**2.18**	0.00	P	1079.27	UBE2V1	20q13.2
239835_at	1.00	0.96	P	42.33	**2.18**	0.01	P	90.60	TA-KRP	3p14
218093_s_at	0.99	0.93	P	173.17	**2.18**	0.00	P	372.40	ANKRD10	13q34
225692_at	1.00	0.97	P	606.20	**2.18**	0.00	P	1296.03	CAMTA1	1p36.23
218376_s_at	0.99	0.92	P	159.90	**2.18**	0.03	P	345.23	NICAL	6q21
204335_at	1.00	0.97	P	62.67	**2.18**	0.01	P	134.70	FLJ10374	19p13.3
34764_at	1.00	0.95	P	112.97	**2.18**	0.01	P	241.53	LARS2	3p21.3
224573_at	0.99	0.95	P	1975.23	**2.17**	0.00	P	4237.67	MGC49942	17p13.2
242384_at	1.00	0.96	P,A	29.33	**2.17**	0.02	P	63.23		
204040_at	1.00	0.97	P	190.93	**2.17**	0.01	P	407.47	RNF144	2p25.2
35671_at	1.00	0.97	P	252.17	**2.17**	0.00	P	537.90	GTF3C1	16p12
217852_s_at	1.00	0.96	P	547.17	**2.17**	0.00	P	1169.97	ARL10C	3p26.1
241464_s_at	1.00	0.97	A	75.53	**2.17**	0.00	P	161.63		
222430_s_at	0.99	0.94	P	382.10	**2.17**	0.01	P	818.10	HGRG8	1p35
228092_at	0.99	0.94	P	176.03	**2.17**	0.00	P	376.60	CREM	10p11.21
234949_at	0.98	0.91	P	44.50	**2.17**	0.03	P	96.43	MGC72104	20q11.21
224626_at	1.00	0.96	P	496.83	**2.17**	0.00	P	1064.17	SLC35A4	5q31.3
209665_at	1.00	0.96	P	253.27	**2.17**	0.01	P	543.37	CYB561D2	3p21.3
223398_at	0.99	0.94	P	164.73	**2.17**	0.00	P	351.80	C9orf89	9q22.32
208660_at	0.99	0.93	P	1183.03	**2.17**	0.00	P	2530.13	CS	12q13.2-q13.3
225820_at	1.00	0.99	P	59.03	**2.17**	0.00	P	125.73	PHF17	4q26-q27
224783_at	1.00	0.98	P	150.43	**2.16**	0.01	P	322.63	MGC29814	17q25.3
203733_at	0.99	0.92	P	278.90	**2.16**	0.00	P	592.17	DEXI	16p13.2
229930_at	0.99	0.92	A	89.40	**2.16**	0.00	P	189.57		22q13.31
204618_s_at	0.98	0.91	P	137.83	**2.16**	0.01	P	295.40	GABPB2	15q21.2
220753_s_at	1.00	0.96	P,M,A	106.37	**2.16**	0.00	P	226.40	CRYL1	13q12.11
202141_s_at	1.00	0.99	P	1104.33	**2.16**	0.00	P	2345.67	COPS8	2q37.3
225322_s_at	0.99	0.93	A	45.17	**2.16**	0.00	P,M,A	96.37	FLJ22175	17q25.3
217865_at	1.00	0.95	P	629.53	**2.16**	0.00	P	1338.90	RNF130	5q35.3
209416_s_at	1.00	0.96	P	146.17	**2.16**	0.02	P	316.33	FZR1	19p13.3
231656_x_at	0.99	0.91	A	45.17	**2.16**	0.01	P	96.83	OSBPL10	3p22.3
209029_at	1.00	0.99	P	451.87	**2.15**	0.00	P	957.20	COPS7A	12p13.31
202568_s_at	0.99	0.94	P	90.67	**2.15**	0.02	P	195.97	MARK3	14q32.3
242621_at	1.00	0.95	P,M,A	93.13	**2.15**	0.00	P	198.63	ZNF498	7q22.1
212908_at	1.00	0.99	P	102.33	**2.15**	0.00	P	216.63	KIAA0962	1p36.1
201029_s_at	1.00	0.98	P	942.37	**2.15**	0.00	P	1992.57	CD99	xp22.32; yp11.3
1558692_at	0.99	0.94	P	117.63	**2.15**	0.02	P	250.20	MGC31963	1q23.1
205379_at	1.00	0.98	P	640.20	**2.15**	0.00	P	1351.90	CBR3	21q22.2
204808_s_at	0.99	0.94	P	623.87	**2.15**	0.00	P	1316.43	TMEM5	12q14.1
218793_s_at	0.99	0.93	P	151.97	**2.15**	0.00	P	322.57	SCML1	xp22.2-p22.1
204030_s_at	1.00	0.98	P	292.00	**2.15**	0.00	P	617.33	SCHIP1	3q25.33
1559485_at	0.99	0.92	A	28.53	**2.15**	0.02	P,A	60.40		
237157_at	1.00	0.96	A	45.47	**2.15**	0.03	P,A	98.03		
241706_at	1.00	0.95	P,M	220.17	**2.15**	0.00	P	465.63	CPNE8	12q12
209285_s_at	0.98	0.90	P	109.03	**2.15**	0.02	P	233.10	RAP140	3p21.31
219342_at	1.00	0.96	P	87.97	**2.15**	0.00	P	185.67	CAS1	7q21.3
217631_at	0.99	0.92	P	64.97	**2.15**	0.03	P	139.13	GTPBP4	10p15-p14
238785_at	0.98	0.91	P	49.53	**2.15**	0.02	P	105.73		
235676_at	0.99	0.91	P	51.67	**2.15**	0.01	P	109.63	LOC90701	18q21.32
202000_at	1.00	0.98	P	256.97	**2.15**	0.00	P	542.47	NDUFA6	22q13.2-q13.31
1568592_at	1.00	0.96	P,M,A	113.70	**2.14**	0.00	P	239.40		15q15.1
219284_at	1.00	0.99	P	128.20	**2.14**	0.01	P	270.80	HSPBAP1	3q21.1
233707_at	0.99	0.94	A	48.10	**2.14**	0.02	P	103.63		
222681_at	1.00	0.97	P	135.67	**2.14**	0.01	P	287.10	MDS010	3q13.33
201191_at	0.99	0.93	P	172.13	**2.14**	0.00	P	364.37	PITPN	17p13.3
223307_at	0.99	0.95	P	536.10	**2.14**	0.00	P	1126.50	CDCA3	12p13
228820_at	1.00	0.97	P,A	96.17	**2.14**	0.01	P	203.50	LOC63929	q13.31-q13.33
205259_at	0.99	0.93	A	17.93	**2.14**	0.01	P	37.97	NR3C2	4q31.1
223626_x_at	0.99	0.94	P,M	64.03	**2.14**	0.03	P	135.93	FAM14A	14q32.2
213760_s_at	0.99	0.94	A	115.80	**2.14**	0.00	P	245.13	ZNF330	4q31.1-q31.2
206469_x_at	0.99	0.94	M,A	135.77	**2.14**	0.00	P	285.30	AKR7A2	1p35.1-p36.23
208926_at	0.99	0.94	P	416.27	**2.14**	0.00	P	874.43	NEU1	6p21.3
229743_at	0.99	0.92	A	99.70	**2.14**	0.02	P,M,A	212.13	LOC220929	10p11.23
222554_s_at	0.99	0.94	A	154.47	**2.14**	0.00	P	326.47	NOL6	9p13.3
231823_s_at	1.00	0.98	P	399.67	**2.14**	0.00	P	844.73	KIAA1295	5q35.2
1558714_at	0.99	0.94	P,M,A	33.67	**2.14**	0.03	P	71.53		

212502_at	0.99	0.93	P	391.30	**2.14**	0.00	P	825.63	C10orf22	10q21.3
223082_at	0.99	0.93	P	549.63	**2.14**	0.00	P	1152.27	SH3KBP1	xp22.1-p21.3
225178_at	0.99	0.93	P,M,A	163.43	**2.14**	0.01	P	346.47	TTC14	3q27.2
218884_s_at	0.99	0.93	P	85.10	**2.14**	0.01	P	179.57	FLJ13220	4p13
241362_at	0.99	0.94	A	33.57	**2.14**	0.00	P,A	70.50	LOC28474(	20q11.23
235300_x_at	1.00	0.97	P	40.50	**2.14**	0.00	P	85.33	RCHY1	4q21.21
215031_x_at	1.00	0.96	P	118.10	**2.14**	0.00	P	248.83	RNF126	19p13.3
201168_x_at	1.00	0.98	P	1076.83	**2.14**	0.01	P	2278.87	ARHGDIA	17q25.3
203686_at	0.99	0.94	P	259.93	**2.13**	0.00	P	547.57	MPG	16p13.3
242636_at	0.98	0.91	P,A	22.87	**2.13**	0.01	P	48.50	PRCP	11q14
235918_x_at	1.00	0.97	P	270.03	**2.13**	0.00	P	566.00	FLJ23047	3q12.3
225744_at	0.99	0.94	P	233.27	**2.13**	0.01	P	490.17	ZDHHC8	22q11.21
221964_at	1.00	0.96	P,M	82.80	**2.13**	0.00	P	173.43	TULP3	12p13.3
203578_s_at	0.99	0.93	P	34.97	**2.13**	0.00	P	73.67	SLC7A6	16q22.1
208733_at	0.99	0.95	P	31.97	**2.13**	0.01	P	67.03	RAB2	8q12.1
177_at	0.99	0.94	P	30.47	**2.13**	0.01	P	64.47	PLD1	3q26
212470_at	0.99	0.93	P	308.83	**2.13**	0.01	P	650.03	SPAG9	17q21.33
202384_s_at	1.00	0.98	P	112.00	**2.13**	0.01	P	235.20	TCOF1	5q32-q33.1
209213_at	0.99	0.94	P	608.60	**2.13**	0.00	P	1281.47	CBR1	21q22.13
233364_s_at	1.00	0.97	A	14.90	**2.13**	0.02	P	31.47		
201988_s_at	1.00	0.97	P	167.77	**2.13**	0.00	P	352.63	CREBL2	12p13
212018_s_at	0.99	0.92	P	589.03	**2.13**	0.01	P	1243.03	KFZP564M1	16p13.13
201034_at	1.00	0.99	P	430.03	**2.13**	0.00	P	900.13	HADHSC	4q22-q26
200090_at	1.00	0.99	P	609.23	**2.13**	0.00	P	1277.17	FNTA	8p22-q11
227249_at	1.00	0.99	P	246.73	**2.13**	0.01	P	516.50	NDE1	16p13.11
225653_at	0.98	0.90	A	112.87	**2.13**	0.01	P	239.37	TGFBRAP1	2q12.2
225097_at	1.00	0.96	P,A	160.17	**2.13**	0.01	P	335.70	HIPK2	7q32-q34
244631_at	1.00	0.96	P,M	45.27	**2.13**	0.00	P	94.40		
212494_at	1.00	0.97	P	69.60	**2.13**	0.00	P	145.27	TENC1	12q13.13
218164_at	0.99	0.93	P	273.77	**2.12**	0.00	P	569.93	FLJ21347	17q21.33
202572_s_at	1.00	0.96	P	188.33	**2.12**	0.00	P	394.53	DLGAP4	20q11.23
225578_at	1.00	0.97	P	629.57	**2.12**	0.00	P	1317.37		
236042_at	0.99	0.93	P,A	32.07	**2.12**	0.00	P	66.90		12p13.33
238026_at	1.00	0.96	P	124.67	**2.12**	0.02	P	262.97	RPL35A	3q29-qter
203885_at	0.99	0.95	P	195.97	**2.12**	0.00	P	410.57	RAB21	12q15
231894_at	0.99	0.95	P	59.73	**2.12**	0.02	P	126.40		
1553217_s_at	1.00	0.96	M,A	20.37	**2.12**	0.01	P	42.77	ZNF41	xp11.23
207826_s_at	0.99	0.92	M,A	155.07	**2.12**	0.01	P	323.33	ID3	p36.13-p36.12
205080_at	1.00	0.96	A	68.00	**2.12**	0.00	P	141.83	RARB	3p24
234584_s_at	0.99	0.95	P	72.83	**2.12**	0.02	P	153.40	ATE1	10q26.13
205705_at	1.00	0.96	P,A	48.27	**2.12**	0.01	P	101.43	ANKRD26	10pter-q22.1
200950_at	0.99	0.92	P	1237.00	**2.12**	0.00	P	2596.53	ARPC1A	7q22.1
222015_at	1.00	0.97	A	126.17	**2.12**	0.01	P	263.90	CSNK1E	22q13.1
204169_at	1.00	0.96	P	248.10	**2.12**	0.01	P	520.80	IMPDH1	7q31.3-q32
202626_s_at	1.00	0.98	P	57.60	**2.12**	0.01	P	121.00	LYN	8q13
222138_s_at	0.99	0.93	P	262.80	**2.12**	0.00	P	545.33	WDR13	xp11.23
229412_at	0.99	0.94	P,A	91.80	**2.12**	0.00	P	192.07	TBN	6p21.1
205510_s_at	1.00	0.96	P	54.07	**2.12**	0.02	P	113.00	FLJ10038	15q15.3
34689_at	1.00	0.98	A	147.03	**2.12**	0.01	P,A	307.27	TREX1	3p21.3-p21.2
45714_at	0.99	0.92	P	116.77	**2.12**	0.00	P	242.40	HCFC1R1	16p13.3
208949_s_at	1.00	0.99	P	2424.83	**2.12**	0.00	P	5065.07	LGALS3	14q21-q22
215706_x_at	0.99	0.94	P,A	377.63	**2.12**	0.01	P	787.73	ZYX	7q32
1555384_a_at	0.99	0.93	P,A	99.43	**2.12**	0.00	P	208.00	LOC113251	12q13.12
215134_at	1.00	0.97	P,A	46.70	**2.12**	0.00	P	97.53	PI4KII	10q24
222270_at	0.99	0.92	P	49.53	**2.11**	0.03	P	104.57	KIAA1387	2p16.2
204628_s_at	1.00	0.95	A	51.97	**2.11**	0.00	P	108.43	ITGB3	17q21.32
231784_s_at	1.00	0.95	P	295.70	**2.11**	0.01	P	616.50	FZP564O0	8q22.3
243751_at	0.99	0.91	P	26.03	**2.11**	0.01	P	54.33		
223617_x_at	1.00	0.98	P	212.33	**2.11**	0.00	P	442.10	ATAD3A	1p36.33
201186_at	1.00	0.97	P	302.87	**2.11**	0.01	P	628.07	LRPAP1	4p16.3
218984_at	1.00	0.97	P	461.23	**2.11**	0.00	P	957.87	FLJ20485	7q22.2
229697_at	1.00	0.96	A	44.03	**2.11**	0.00	P,A	91.50	HIRIP3	16p12.1
1554638_at	1.00	0.97	P	63.67	**2.11**	0.02	P	132.47	ZFYVE16	5p15.2-q14.3
222277_at	1.00	0.97	A	92.70	**2.11**	0.01	P	194.07		
201805_at	1.00	1.00	P	352.37	**2.11**	0.00	P	731.80	PRKAG1	12q12-q14
209760_at	0.99	0.94	P,M	92.30	**2.11**	0.00	P	190.60	KIAA0922	4q31.3
222886_at	1.00	0.95	P	38.93	**2.11**	0.02	P	81.30	FLJ22609	3q11.2
214718_at	1.00	0.96	P	266.53	**2.11**	0.03	P	557.73	ODAG	7q21-q22
226831_at	1.00	0.96	P	244.33	**2.10**	0.01	P	506.33	LOC91137	5q22.2
224811_at	1.00	0.95	P	457.43	**2.10**	0.00	P	948.13		
239292_at	1.00	0.97	P,A	24.53	**2.10**	0.02	P	51.30		
204778_x_at	0.99	0.94	A	54.53	**2.10**	0.01	P	113.30	HOXB7	17q21.3
225426_at	1.00	0.95	P	168.43	**2.10**	0.00	P	350.07	PPP6C	9q34.11
58780_s_at	1.00	0.96	P,A	46.80	**2.10**	0.01	P	96.77	FLJ10357	14q11.2
221203_s_at	1.00	0.95	P	131.93	**2.10**	0.01	P	273.63	FLJ10201	3q27.3
208722_s_at	1.00	0.96	P	500.03	**2.10**	0.00	P	1034.70	ANAPC5	12q24.31
230970_at	0.98	0.91	P,A	25.93	**2.10**	0.03	P	54.30		
225221_at	1.00	0.98	P	530.73	**2.10**	0.00	P	1095.27		
218898_at	1.00	0.99	P	221.53	**2.10**	0.00	P	457.57	CT120	17p13.3
218212_s_at	0.99	0.93	P	318.50	**2.10**	0.00	P	659.83	MOCS2	5q11
204252_at	1.00	0.96	P	505.83	**2.10**	0.00	P	1042.67	CDK2	12q13
239742_at	0.98	0.91	P	144.23	**2.10**	0.03	P	301.67	TULP4	6q25-q26
201889_at	1.00	0.95	P	880.13	**2.10**	0.00	P	1818.00	FAM3C	7q22.1-q31.1
214037_s_at	0.99	0.93	P	40.10	**2.09**	0.01	P	83.27	JM1	xp11.23
203367_at	1.00	0.97	P	285.47	**2.09**	0.00	P	588.47	DUSP14	17q12
224869_s_at	0.99	0.92	P	381.87	**2.09**	0.01	P	790.87	MRPS25	3p25
235079_at	0.99	0.95	P	172.30	**2.09**	0.02	P	357.10		
243815_at	0.99	0.92	M,A	46.50	**2.09**	0.02	P	96.77	PGBD4	15q13.2
227917_at	1.00	0.98	P	72.03	**2.09**	0.00	P	148.43		
201657_at	1.00	0.97	P	140.80	**2.09**	0.00	P	290.07	ARL1	12q23.3
203834_s_at	1.00	0.97	P,A	176.17	**2.09**	0.00	P	363.77	TGOLN2	2p11.2
201206_s_at	1.00	1.00	P	159.73	**2.09**	0.01	P	328.97	RRBP1	20p12
201114_x_at	1.00	0.98	P	1322.83	**2.09**	0.00	P	2720.63	PSMA7	20q13.33
203183_s_at	1.00	0.96	P	120.87	**2.09**	0.01	P	250.93	SMARCD1	12q13-q14
1559820_at	1.00	0.95	A	40.97	**2.09**	0.02	P,A	85.27	APG10L	5q14.1
212276_at	1.00	0.96	P	157.97	**2.09**	0.00	P	325.10	LPIN1	2p25.1
1566739_at	0.99	0.93	A	21.23	**2.09**	0.01	P,A	43.97	PLCE1	10q23
219049_at	1.00	0.96	P	189.43	**2.09**	0.00	P	388.20	ChGn	8p21.3
219262_at	0.99	0.91	P	40.80	**2.09**	0.02	P	84.90	SUV39H2	10p13
201949_x_at	0.99	0.93	P	709.93	**2.09**	0.01	P	1452.67	CAPZB	1p36.1
201580_s_at	1.00	0.97	P	450.07	**2.08**	0.00	P	922.77	DJ971N18.2	20p12
207396_s_at	0.99	0.95	P	513.47	**2.08**	0.00	P	1059.37	ALG3	3q27.3
237176_at	0.99	0.94	P,A	64.87	**2.08**	0.00	P	133.53		
1556925_at	0.99	0.93	P	19.73	**2.08**	0.01	P	40.70	CSPG6	10q25
228499_at	0.99	0.95	A	88.97	**2.08**	0.02	P	182.90	PFKFB4	3p21-p22
205570_at	1.00	0.96	P,A	62.07	**2.08**	0.00	P	127.30	PIP5K2A	10p12.32
229744_at	0.99	0.93	P	195.23	**2.08**	0.01	P	404.80	SSFA2	2q32.1
218158_s_at	0.99	0.93	P	157.47	**2.08**	0.00	P	323.40	APPL	3p21.1-p14.3
213923_at	1.00	0.97	P	1052.17	**2.08**	0.00	P	2153.63	RAP2B	3q25.2
31837_at	1.00	0.97	P,M	442.63	**2.08**	0.00	P	906.93	BC002942	22q13.33
226780_s_at	1.00	0.96	P	510.53	**2.08**	0.00	P	1041.93	HSPC268	7q34
218472_s_at	1.00	0.98	P	235.60	**2.08**	0.00	P	481.63	PELO	5q11.2
209264_s_at	1.00	0.96	P,A	259.17	**2.08**	0.01	P	531.67	TM4SF7	11p15.5
224660_at	1.00	0.98	P	1321.67	**2.08**	0.00	P	2702.80	MGC14156	4q22.1
223299_at	0.99	0.93	P	595.33	**2.08**	0.01	P	1224.40	LOC90701	18q21.32
212722_s_at	1.00	0.96	P	148.97	**2.08**	0.01	P	304.83	PTDSR	17q25
227611_at	0.99	0.94	P	36.70	**2.07**	0.00	P	75.07	FLJ25005	15q26.3
233015_at	1.00	0.96	A	27.63	**2.07**	0.00	P,M	56.37	MBNL1	3q25
235714_at	0.99	0.93	P,M	50.10	**2.07**	0.01	P	101.83	GANC	15q15.2
222044_at	1.00	0.95	A	153.47	**2.07**	0.00	P,M	312.03	C20orf67	20q13.12
205088_at	1.00	0.96	P	210.27	**2.07**	0.00	P	429.60	CXorf6	xq28
201753_s_at	1.00	0.98	P	412.77	**2.07**	0.00	P	840.53	ADD3	0q24.2-q24.3
226579_at	0.99	0.93	P	180.90	**2.07**	0.00	P	367.30	XRCC3	14q32.3
223004_s_at	0.99	0.95	P	1249.70	**2.07**	0.00	P	2552.70	C3orf1	3q13.33
224706_at	1.00	0.96	P	319.90	**2.07**	0.00	P	651.90	MGC33867	1p36.21
219217_at	0.99	0.93	P	456.77	**2.07**	0.00	P	935.23	FLJ23441	11q13.4
204715_at	1.00	0.97	P	158.83	**2.07**	0.00	P	324.50	PANX1	11q21
201332_s_at	0.99	0.93	A	60.60	**2.07**	0.01	P	124.50	STAT6	12q13
224604_at	1.00	0.98	P	436.17	**2.07**	0.01	P	886.33		4q27
1558166_at	0.99	0.92	P	36.90	**2.07**	0.00	P	75.67	MGC16275	17q25.2
218161_s_at	1.00	0.96	P,A	113.43	**2.07**	0.00	P	230.20	CLN6	15q22.31
1566284_at	1.00	0.96	P,A	52.20	**2.07**	0.00	P	106.30		
226347_at	1.00	0.97	P	320.73	**2.07**	0.01	P	653.03	FUT11	10q22.3
238011_at	0.99	0.92	P	45.07	**2.06**	0.01	P	92.20	PRO0149	16p13.2

1559399_s_at	1.00	0.96	P	105.33	**2.06**	0.03	P	216.23	ZCCHC10	5q31.1
241953_at	1.00	0.97	P,M,A	11.07	**2.06**	0.00	P,M	22.53	FLJ25694	13q21.2
201105_at	1.00	0.96	P	2764.97	**2.06**	0.00	P	5637.70	LGALS1	22q13.1
201765_s_at	1.00	0.96	P	348.63	**2.06**	0.01	P	709.27	HEXA	15q23-q24
212244_at	1.00	0.97	P	299.50	**2.06**	0.00	P	606.77	GRINL1A	15q22.1
226531_at	1.00	0.96	M,A	194.83	**2.06**	0.00	P	394.37	FLJ14466	12q24.31
1567080_s_at	1.00	0.99	P	248.70	**2.06**	0.01	P	507.47	CLN6	15q22.31
217751_at	1.00	0.96	P	866.83	**2.06**	0.00	P	1757.40	GSTK1	7q35
205601_s_at	0.99	0.94	P,A	48.17	**2.06**	0.01	P	97.40	HOXB5	17q21.3
228088_at	0.99	0.93	P	58.07	**2.06**	0.00	P	117.40		
201663_s_at	0.99	0.94	P	868.53	**2.06**	0.00	P	1768.70	SMC4L1	3q26.1
226199_at	0.99	0.94	P	77.07	**2.06**	0.00	P	156.33	MGC23937	xq13.2
203525_s_at	0.99	0.93	P	276.33	**2.06**	0.01	P	562.33	APC	5q21-q22
236667_at	0.99	0.94	P	85.07	**2.06**	0.02	P	174.10		12q24.31
212672_at	0.98	0.91	P	161.20	**2.06**	0.03	P	330.70	ATM	11q22-q23
217911_s_at	0.99	0.93	P	944.97	**2.06**	0.00	P	1918.17	BAG3	0q25.2-q26.2
205608_s_at	1.00	0.97	P,A	44.63	**2.06**	0.00	P	90.13	ANGPT1	8q22.3-q23
238045_at	1.00	0.98	P	57.43	**2.06**	0.00	P	116.37	LOC157378	8q24.13
230259_at	0.99	0.93	P	88.80	**2.06**	0.00	P	178.87	C10orf125	10q26.3
227330_x_at	1.00	0.96	P,A	51.80	**2.05**	0.03	P	106.63	LOC285458	4
203211_s_at	1.00	0.95	P	767.23	**2.05**	0.00	P	1553.73	MTMR2	11q22
232216_at	1.00	0.98	P	112.00	**2.05**	0.01	P	226.63	YME1L1	10p14
202114_at	1.00	0.97	P	252.37	**2.05**	0.00	P	509.20	SNX2	5q23
218620_s_at	1.00	0.96	A	106.07	**2.05**	0.00	P	214.60	HEMK	3p21.3
202454_s_at	1.00	0.97	P	350.33	**2.05**	0.00	P	707.10	ERBB3	12q13
206583_at	0.99	0.94	P,A	85.43	**2.05**	0.01	P	172.67	FLJ20344	xp11.3
217918_at	1.00	0.98	P	1262.63	**2.05**	0.00	P	2547.90	DNCL2A	20q11.21
208946_s_at	1.00	0.96	P	465.80	**2.05**	0.00	P	940.70	BECN1	17q21
241384_x_at	0.99	0.95	P	43.87	**2.05**	0.02	P	89.40	MGC40579	3q23
223388_s_at	1.00	0.99	A	63.70	**2.05**	0.00	P	128.30	ZFYVE1	14q22-q24
1554557_at	1.00	0.96	P	45.93	**2.05**	0.02	P	93.30	ATP11B	3q27
1555131_a_at	1.00	0.96	A	77.47	**2.05**	0.01	P	157.33	PER3	1p36.23
207661_s_at	0.98	0.91	A	26.40	**2.04**	0.01	P	53.60	SH3MD1	10q25.1
227693_at	1.00	0.99	P	87.20	**2.04**	0.00	P	175.27	WDR20	14q32.33
205411_at	0.98	0.91	P,A	54.70	**2.04**	0.03	P	113.13	STK4	0q11.2-q13.2
235320_at	1.00	0.96	P	85.57	**2.04**	0.01	P	172.10	ARL6	3q12.1
212522_at	0.99	0.93	P	313.50	**2.04**	0.01	P	632.77	PDE8A	15q25.2
219999_at	0.99	0.91	P	44.43	**2.04**	0.01	P	89.13	MAN2A2	15q26.1
230048_at	1.00	0.96	P	56.73	**2.04**	0.01	P	114.07		
227107_at	1.00	0.96	P	363.17	**2.04**	0.01	P	728.00	PANX1	11q21
201835_s_at	0.99	0.94	A	61.40	**2.04**	0.00	P	124.03	PRKAB1	12q24.1
209028_s_at	1.00	0.97	P	292.83	**2.04**	0.02	P	590.33	ABI1	10p11.2
224825_at	1.00	0.96	P,M,A	166.80	**2.04**	0.00	P	336.40	DNTTIP1	20q13.12
238056_at	0.99	0.93	P	132.80	**2.04**	0.00	P	267.87		1q23.1
1555501_s_at	0.99	0.94	P	233.40	**2.04**	0.00	P	466.30	MGC12197	3q25.32
235987_at	0.99	0.93	M,A	14.43	**2.04**	0.02	P	29.17	PRKXP1	15q26
226569_s_at	0.99	0.93	P	108.33	**2.04**	0.01	P	217.17	CHTF18	16p13.3
224332_s_at	0.99	0.94	P	423.13	**2.04**	0.00	P	844.80	MRPL43	10q24.31
219184_x_at	0.99	0.93	A	99.70	**2.04**	0.01	P	199.47	TIMM22	17p13
228685_at	1.00	0.96	P	38.80	**2.04**	0.01	P,A	77.80		
236402_at	1.00	0.98	P,M,A	47.40	**2.04**	0.00	P	95.00	BRAF	7q34
233995_at	0.98	0.91	P	70.57	**2.03**	0.03	P	142.47		
238691_at	1.00	0.96	A	25.70	**2.03**	0.00	P	51.60	LOC283596	14q32.2
232475_at	1.00	0.96	A	79.70	**2.03**	0.00	P	160.07	MGC45866	15q26.1
213113_s_at	1.00	0.96	P	935.47	**2.03**	0.00	P	1865.33	SLC43A3	11q11
212020_s_at	1.00	0.97	P	299.57	**2.03**	0.01	P	607.10	MKI67	10q25-qter
238015_at	1.00	0.95	P	143.87	**2.03**	0.00	P	288.33	LOC201725	4q32.1
202733_at	1.00	0.98	P	190.37	**2.03**	0.00	P	380.53	P4HA2	5q31
217917_s_at	1.00	0.98	P	800.13	**2.03**	0.00	P	1595.60	DNCL2A	20q11.21
241938_at	0.99	0.95	P,M	49.30	**2.03**	0.01	P	98.83	QKI	6q26-27
222233_s_at	0.99	0.94	P	126.57	**2.03**	0.00	P	253.50	DCLRE1C	10p13
201271_s_at	1.00	0.98	P	428.90	**2.03**	0.00	P	856.17	RALY	q11.21-q11.23
241924_at	0.99	0.93	P	132.87	**2.03**	0.01	P	266.63		
1552364_s_at	0.99	0.92	P	51.97	**2.03**	0.01	P	103.93	MSI2	17q23.2
201682_at	1.00	0.96	P	548.30	**2.03**	0.02	P	1100.07	PMPCB	7q22-q32
222767_s_at	0.99	0.95	P	90.57	**2.03**	0.00	P	180.73	FLJ21415	12q24.22
224931_at	1.00	0.95	M,A	168.40	**2.03**	0.00	P	335.77	SLC41A3	3q21.2
232889_at	0.99	0.93	P	248.57	**2.03**	0.01	P	494.23	LOC153561	5q13.2
216913_s_at	1.00	0.96	P,M,A	135.40	**2.03**	0.00	P	270.13	KIAA0690	10q24.2
223186_at	0.99	0.95	P	276.40	**2.03**	0.00	P	552.47	Kua	20q13.2
222598_s_at	0.99	0.93	A	51.70	**2.03**	0.01	P	103.07	NAV2	11p15.1
201377_at	1.00	0.98	P	434.50	**2.02**	0.00	P	865.43	NICE-4	1q22
203510_at	1.00	0.95	P	714.60	**2.02**	0.01	P	1427.57	MET	7q31
202260_s_at	1.00	0.97	P	318.47	**2.02**	0.02	P	643.13	STXBP1	9q34.1
212282_at	1.00	0.96	P	499.67	**2.02**	0.00	P	996.40	MAC30	17q11.2
210771_at	0.99	0.91	P	17.17	**2.02**	0.01	P	34.37	PPARA	22q13.31
212067_s_at	0.99	0.93	A	20.23	**2.02**	0.01	P	40.37	C1R	12p13
200622_x_at	1.00	0.97	P	355.53	**2.02**	0.00	P	708.47	CALM3	9q13.2-q13.3
203094_at	1.00	0.96	P	405.93	**2.02**	0.00	P	809.23	MAD2L1BP	6p21.1
226924_at	0.99	0.95	P	94.93	**2.02**	0.01	P	189.27		18q22.3
221912_s_at	0.99	0.95	A	50.23	**2.02**	0.03	P	100.57	MGC1203	p36.11-p34.2
201016_at	0.99	0.94	P	883.77	**2.02**	0.00	P	1760.93	EIF1AX	xp22.13
203314_at	1.00	0.95	P	129.57	**2.02**	0.01	P	256.90	PGPL	22.33; yp11.32
201281_at	1.00	0.98	P	1111.27	**2.02**	0.00	P	2208.03	ADRM1	20q13.33
206289_at	0.99	0.95	P	105.53	**2.02**	0.02	P	210.77	HOXA4	7p15-p14
206412_at	1.00	0.97	A	67.63	**2.02**	0.02	P,M	135.20	FER	5q21
208914_at	1.00	0.96	M,A	155.87	**2.02**	0.00	P	309.13	GGA2	16p12
203832_at	1.00	0.99	P	527.20	**2.02**	0.00	P	1044.43	SNRPF	12q23.1
225104_at	1.00	0.96	P	164.70	**2.01**	0.01	P	328.30	ZNF598	16p13.3
202243_s_at	1.00	0.99	P	1967.63	**2.01**	0.00	P	3900.77	PSMB4	1q21
36994_at	0.99	0.94	P	1376.80	**2.01**	0.00	P	2743.23	ATP6V0C	16p13.3
221764_at	0.99	0.94	P,A	208.17	**2.01**	0.00	P	415.43	C19orf22	19p13.3
218495_at	1.00	0.98	P	899.20	**2.01**	0.00	P	1780.80	UXT	p11.23-p11.22
212373_at	0.99	0.93	P	118.07	**2.01**	0.01	P	235.07	FEM1B	15q22
226395_at	0.99	0.93	P	161.03	**2.01**	0.03	P	322.60	LOC286170	8p11.21
238841_at	1.00	1.00	P	63.80	**2.01**	0.01	P	126.63	PTPDC1	9q22.32
224936_at	1.00	0.98	P	886.73	**2.01**	0.00	P	1753.37	EIF2S3	xp22.2-p22.1
1558459_s_at	0.99	0.94	A	94.03	**2.01**	0.00	P	185.73		7
212792_at	0.99	0.92	P	175.77	**2.01**	0.00	P	348.97	KIAA0877	7p14.3-p14.2
226413_at	1.00	1.00	P	160.30	**2.01**	0.01	P	317.47		12q13.11
229835_s_at	1.00	0.96	P	182.57	**2.01**	0.00	P	361.43	C20orf45	20q13.32
235472_at	0.99	0.92	P	51.57	**2.01**	0.01	P	103.00	FUT10	8p12
241727_x_at	1.00	0.97	A	87.20	**2.01**	0.00	P	172.13	LOC200895	3q11.2
1552278_a_at	1.00	0.98	A	31.23	**2.01**	0.02	P	62.13	MGC9564	17q11.2
202405_at	0.99	0.95	P	80.80	**2.01**	0.01	P	159.23		
201231_s_at	1.00	0.99	P	2900.57	**2.01**	0.00	P	5730.03	ENO1	1p36.3-p36.2
218926_at	1.00	0.98	P	88.50	**2.01**	0.00	P	174.73	MYNN	3q26.31
229647_at	1.00	0.97	P	204.20	**2.00**	0.01	P	403.27		
234594_at	1.00	0.96	P	58.83	**2.00**	0.01	P	116.23	C14orf85	14
223387_at	1.00	0.98	P,M	199.07	**2.00**	0.01	P	392.00	ZFYVE1	14q22-q24
227841_at	0.99	0.93	P,A	133.27	**2.00**	0.01	P,M,A	261.90		
223346_at	1.00	0.97	P	151.93	**2.00**	0.00	P	299.37	VPS18	15q14-q15
202142_at	1.00	0.98	P	787.93	**2.00**	0.00	P	1552.20	COPS8	2q37.3
