# Supplementary material for: Multi-Platform Whole-Genome Microarray Analyses Refine the Epigenetic Signature of Breast Cancer Metastasis with Gene Expression and Copy Number
Source: PLoS One. 2010 Jan 13;5(1):e8665. doi: 10.1371/journal.pone.0008665 (PMC2801616; doi:10.1371/journal.pone.0008665)
Supplement: Table S5 — Genes significantly decreased in expression, 468LN vs 468GFP. Expression microarray (HGU133 Plus_2) data were prefiltered to remove genes changing less than 2 fold, and an ANOVA was run to determine significant (p<0.05) changers. A multiple testing correction using the algorithm of Benjamini and Hochberg was used to reduce the false discovery rate. (0.51 MB PDF) [file pone.0008665.s006.pdf]

Supplemental Table 5: Genes significantly Decreased in expression, LN vs GFP

Cell line ()*	468GFP				468GFP-LN					
Systematic	Ratio to 468GFP	t-test P-value	Flags	Raw	Ratio to 468GFP	t-test P-value	Flags	Raw	Common	Map
205709_s_at	1.00	0.98	P	539.87	0.01	1.00	A	1.10	CDS1	4q21.23
205916_at	0.98	0.91	P	696.33	0.01	1.00	A	3.43	S100A7	1q21
201839_s_at	1.00	0.97	P	3576.00	0.01	1.00	A	7.87	TACSTD1	2p21
201951_at	0.95	0.85	P	503.97	0.01	1.00	A	2.60	ALCAM	3q13.1
202286_s_at	1.00	0.98	P	3076.10	0.01	1.00	A	7.53	TACSTD2	1p32-p31
202437_s_at	0.99	0.95	P	4115.27	0.01	1.00	A	5.17	CYP1B1	2p21
201983_s_at	0.99	0.93	P	5577.07	0.01	1.00	A	14.07	EGFR	7p12
205668_at	1.00	0.97	P	279.00	0.01	1.00	A	1.83	LY75	2q24
202546_at	1.00	0.99	P	1207.63	0.01	1.00	A	3.63	VAMP8	2p12-p11.2
200606_at	1.00	0.96	P	3626.30	0.01	1.00	P,A	21.07	DSP	6p24
206023_at	1.00	0.98	P	959.43	0.01	1.00	A	1.77	NMU	4q12
206378_at	1.00	0.97	P	3826.73	0.01	1.00	A	11.23	SCGB2A2	11q13
65517_at	1.00	1.00	P	1369.27	0.01	1.00	A	10.20	AP1M2	19p13.2
205980_s_at	0.99	0.93	P	897.83	0.01	1.00	A	3.87	ARHGAP8	22q13.31
205928_at	1.00	0.96	P	450.50	0.01	1.00	A	2.67	ZNF443	19p13.2
201427_s_at	1.00	0.95	P	441.03	0.01	1.00	A	1.53	SEPP1	5q31
201015_s_at	1.00	0.96	P	1639.27	0.01	1.00	A	5.27	JUP	17q21
201131_s_at	1.00	1.00	P	1571.40	0.01	1.00	A	4.63	CDH1	16q22.1
201650_at	1.00	1.00	P	5467.43	0.01	1.00	A	0.83	KRT19	17q21.2
204750_s_at	1.00	0.97	P	261.37	0.01	1.00	A	2.30	DSC2	18q12.1
203608_at	1.00	0.98	P	554.13	0.01	1.00	A	1.27	ALDH5A1	6p22.2-p22.3
203215_s_at	0.99	0.95	P	366.60	0.01	1.00	A	1.70	MYO6	6q13
203571_s_at	1.00	0.97	P	198.23	0.01	1.00	A	1.43	C10orf116	10q23.31
203917_at	1.00	0.96	P	1045.50	0.01	1.00	A	8.53	CXADR	21q21.1
204070_at	1.00	0.96	P	1944.63	0.01	1.00	A	11.77	RARRES3	11q23
204351_at	1.00	0.95	P	1378.43	0.01	1.00	A	4.50	S100P	4p16
203953_s_at	0.98	0.91	P	313.83	0.01	1.00	A	1.07	CLDN3	7q11.23
204664_at	0.99	0.95	P,A	364.83	0.01	1.00	A	1.80	ALPP	2q37
266_s_at	1.00	0.97	P	2977.27	0.01	1.00	A	2.67	CD24	6q21
202669_s_at	0.99	0.93	P	522.23	0.01	1.00	A	3.13	EFNB2	13q33
202599_s_at	1.00	0.95	P	1603.40	0.01	1.00	A	9.07	NRIP1	21q11.2
202600_s_at	0.99	0.92	P	1401.20	0.01	1.00	A	3.87	NRIP1	21q11.2
202890_at	1.00	0.98	P	802.87	0.01	1.00	A	0.43	MAP7	6q23.2
203180_at	0.99	0.94	P	3928.07	0.01	1.00	A	7.20	ALDH1A3	15q26.3
205157_s_at	1.00	0.97	P	2251.60	0.01	1.00	A	8.30	KRT17	17q12-q21
205422_s_at	0.99	0.94	P	272.33	0.01	1.00	A	0.77	ITGBL1	13q33
203028_s_at	1.00	0.98	P,A	1742.70	0.01	1.00	A	9.30	CYBA	16q24
204400_at	1.00	0.99	P	243.23	0.01	1.00	A	1.67	EFS	14q11.2-q12
241950_at	1.00	0.95	P	579.20	0.01	1.00	A	4.20		
223631_s_at	0.97	0.88	P	497.10	0.01	1.00	A	2.47	C19orf33	19q13.13
222450_at	1.00	0.95	P,A	1001.20	0.01	1.00	A	1.77	TMEPAI	20q13.31-q13.33
223136_at	1.00	0.98	P	664.90	0.01	1.00	A	3.07	AIG1	6q24.1
224609_at	0.99	0.93	P	2518.37	0.01	1.00	A	5.83	CTL2	19p13.1
225911_at	0.99	0.94	P	551.93	0.01	1.00	A	3.97	LOC255743	4q25
226482_s_at	1.00	0.97	P	2449.33	0.01	1.00	A	5.47	F11R	1q21.2-q21.3
225175_s_at	1.00	0.98	P	2733.47	0.01	1.00	A	2.07	CTL2	19p13.1
225645_at	1.00	0.98	P	2438.20	0.01	1.00	A	4.63	EHF	11p12
219850_s_at	1.00	0.96	P	369.87	0.01	1.00	A	1.57	EHF	11p12
220145_at	1.00	0.99	P	96.13	0.01	1.00	A	0.50	FLJ21159	4q31.3
218885_s_at	1.00	0.98	P	320.53	0.01	1.00	A	2.40	GALNT12	9q31.1
218963_s_at	1.00	0.95	P	1048.90	0.01	1.00	A	6.07	KRT23	17q21.2
220520_s_at	1.00	0.97	P	124.83	0.01	1.00	A	0.77	FLJ20130	xq22.3
221872_at	1.00	0.96	P	4082.57	0.01	1.00	A	10.73	RARRES1	3q25.32
222062_at	1.00	0.97	P	258.27	0.01	1.00	A	1.43	IL27RA	19p13.11
221024_s_at	1.00	0.97	P	342.20	0.01	1.00	A	2.97	SLC2A10	20q13.1
221609_s_at	1.00	0.96	P	1285.07	0.01	1.00	A	5.00	WNT6	2q35
226535_at	1.00	0.99	P	808.43	0.01	1.00	A	2.77	ITGB6	2q24.3
231618_s_at	1.00	0.96	P	944.37	0.01	1.00	A	2.73	MGC33329	7p12.3
231856_at	0.98	0.89	P	170.63	0.01	1.00	A	1.00	KIAA1244	6q23.3
230641_at	0.98	0.91	P	234.17	0.01	1.00	A	1.30		
230875_s_at	1.00	0.96	P	776.23	0.01	1.00	A	1.87	ATP11A	13q34
232165_at	1.00	0.98	P	302.30	0.01	1.00	A	1.50	EPPK1	8q24.3
234310_s_at	0.96	0.86	P	738.43	0.01	1.00	A	4.10	SUSD2	22q11-q12
235626_at	1.00	0.99	P	448.80	0.01	1.00	A	3.43	CAMK1D	10p14
232541_at	1.00	0.99	P	3491.80	0.01	1.00	A	5.70		
232925_at	1.00	0.99	P	982.70	0.01	1.00	A	4.53		
227240_at	1.00	0.98	P	504.50	0.01	1.00	A	2.83	NGEF	2q37
227556_at	1.00	0.98	P	1680.43	0.01	1.00	A	8.30	NME7	1q24
226722_at	1.00	0.96	P	262.63	0.01	1.00	A	2.00	FAM20C	7p22.3
227235_at	1.00	0.97	P	218.90	0.01	1.00	A	1.50		
1553423_a_at	0.99	0.94	P	134.33	0.01	1.00	A	0.57	FLJ31952	17q21.1
229377_at	1.00	0.98	P	625.40	0.01	1.00	A	3.47	GRTP1	13q34
1553132_a_at	0.98	0.91	P	155.23	0.01	1.00	A	0.30	MTAC2D1	14q32.12
227947_at	1.00	0.96	P	715.20	0.01	1.00	A	1.83	C6orf56	6q24.1
228072_at	1.00	0.99	P	1195.93	0.01	1.00	A	5.70	SYT12	11q13.1
209581_at	1.00	0.96	P	2317.50	0.01	1.00	A	6.23	HRASLS3	11q13.1
209587_at	0.99	0.94	P	311.57	0.01	1.00	A	1.00	PITX1	5q31
209260_at	1.00	0.98	P	904.43	0.01	1.00	A	7.00	SFN	1p35.3
209351_at	1.00	0.98	P	2758.37	0.01	1.00	A	3.50	KRT14	17q12-q21
209602_s_at	0.99	0.94	P	353.70	0.01	1.00	A	1.43	GATA3	10p15
209772_s_at	0.95	0.84	P	1214.10	0.01	1.00	A	3.03	CD24	6q21
209792_s_at	0.99	0.94	P	1164.60	0.01	1.00	A	6.73	KLK10	19q13.3-q13.4
209605_at	1.00	0.97	P	1103.03	0.01	1.00	A	9.77	TST	22q13.1
209771_x_at	1.00	0.97	P	4909.50	0.01	1.00	A	19.77	CD24	6q21
206546_at	0.96	0.87	P	103.67	0.01	1.00	A	0.53	SYCP2	20q13.33
206683_at	1.00	0.95	P	281.10	0.01	1.00	A	1.63	ZNF165	6p21.3
206391_at	0.99	0.92	P	1577.87	0.01	1.00	A	2.37	RARRES1	3q25.32

206392_s_at	0.98	0.91	P	3872.73	0.01	1.00	A	3.90	RARRES1	3q25.32
207169_x_at	1.00	0.99	P	2721.47	0.01	1.00	A	14.83	DDR1	6p21.3
208650_s_at	0.99	0.94	P	3063.90	0.01	1.00	A	6.30	CD24	6q21
209016_s_at	1.00	0.98	P	4535.60	0.01	1.00	A	1.87	KRT7	12q12-q13
208091_s_at	1.00	0.99	P	5033.30	0.01	1.00	A	14.90	DKFZP564K0822	7p11.2
208502_s_at	0.99	0.93	P	377.77	0.01	1.00	A	1.40	PITX1	5q31
209885_at	0.99	0.92	P	546.60	0.01	1.00	A	2.90	RHOD	11q14.3
216438_s_at	1.00	0.97	P	5250.40	0.01	1.00	M,A	11.47	TMSB4X	xq21.3-q22
217762_s_at	1.00	0.98	P	2044.03	0.01	1.00	A	9.37	RAB31	18p11.3
215172_at	0.98	0.91	P	325.47	0.01	1.00	A	1.37	DKFZP566K0524	10q11.22
215729_s_at	1.00	0.96	P	2166.23	0.01	1.00	A	2.73	VGLL1	xq26.3
217763_s_at	1.00	0.99	P	1166.10	0.01	1.00	A	5.37	RAB31	18p11.3
218729_at	0.99	0.93	P	1157.30	0.01	1.00	A	4.07	LXN	3q25.32
218804_at	1.00	0.95	P	747.20	0.01	1.00	A	1.83	TMEM16A	11q13.2
217764_s_at	1.00	0.98	P	2323.80	0.01	1.00	A	14.57	RAB31	18p11.3
218312_s_at	0.99	0.93	P	993.13	0.01	1.00	A	7.17	FLJ12895	19q13.43
211379_x_at	0.99	0.92	P	407.27	0.01	1.00	A	1.90	B3GALT3	3q25
212148_at	1.00	0.96	P	981.40	0.01	1.00	A	6.43	PBX1	1q23
210095_s_at	1.00	0.96	P	1715.87	0.01	1.00	A	6.23	IGFBP3	7p13-p12
210715_s_at	1.00	0.99	P	2457.40	0.01	1.00	A	10.30	SPINT2	19q13.1
212768_s_at	1.00	0.97	P	187.57	0.01	1.00	A	0.70	GW112	13q14.2
213711_at	0.99	0.94	P	2111.43	0.01	1.00	A	8.33	KRTHB1	12q13
214404_x_at	0.95	0.84	P	1346.00	0.01	1.00	A	10.70	SPDEF	6p21.3
213506_at	1.00	0.95	P	1105.20	0.01	1.00	A	1.23	F2RL1	5q13
213693_s_at	1.00	0.97	P	528.70	0.01	1.00	A	2.47	MUC1	1q21
236489_at	0.99	0.92	P	230.77	0.01	1.00	A	1.57		
204734_at	1.00	0.97	P	244.13	0.01	0.00	A	2.00	KRT15	17q21.2
202790_at	0.86	0.75	P	1495.10	0.01	0.00	A	9.77	CLDN7	17p13
231766_s_at	0.99	0.93	P	177.37	0.01	0.00	A	1.17	COL12A1	6q12-q13
221690_s_at	1.00	0.98	P	1093.43	0.01	0.00	A	8.10	NALP2	19q13.42
201820_at	1.00	0.99	P	3294.47	0.01	0.00	A	19.97	KRT5	12q12-q13
227702_at	0.98	0.90	P	584.30	0.01	0.00	A	4.63	CYP4X1	1p33
209369_at	0.99	0.94	P	1353.70	0.01	0.00	A	9.33	ANXA3	4q13-q22
1560562_a_at	1.00	0.99	P	312.80	0.01	0.00	A	2.13	MGC48625	19q13.42
217388_s_at	0.99	0.94	P	1995.87	0.01	0.00	M,A	19.43	KYNU	2q22.3
238029_s_at	1.00	0.98	P	332.10	0.01	0.00	A	2.87	SLC16A14	2q37.1
226039_at	1.00	0.97	P	487.47	0.01	0.00	A	4.27	MGAT4A	2q12
226374_at	1.00	0.97	P	1111.03	0.01	0.00	A	8.40		
227574_at	1.00	0.97	P	1128.77	0.01	0.00	A	10.03	KIAA0657	2q36.1
206067_s_at	1.00	0.96	P	504.27	0.01	0.00	A	2.90	WT1	11p13
208651_x_at	0.99	0.94	P	2632.30	0.01	0.00	A	26.50	CD24	6q21
202826_at	1.00	0.99	P	706.17	0.01	0.00	A	5.10	SPINT1	15q14
227480_at	1.00	0.99	P	3149.47	0.01	0.00	A	20.57	SUSD2	22q11-q12
228707_at	1.00	0.97	P	230.87	0.01	0.00	A	2.03	CLDN23	8p23.1
203836_s_at	0.99	0.92	P	555.97	0.01	0.00	A	4.17	MAP3K5	6q22.33
218546_at	0.99	0.93	P	490.00	0.01	0.00	A	4.20	FLJ14146	1q42.11
216905_s_at	1.00	0.97	P	529.57	0.01	0.00	A	4.53	ST14	11q24-q25
238604_at	1.00	0.97	P	1592.30	0.01	0.00	P	14.30		
203535_at	1.00	0.97	P	823.60	0.01	0.00	A	6.67	S100A9	1q21
223349_s_at	1.00	0.96	P	420.17	0.01	0.00	A	3.23	BOK	2q37.3
202489_s_at	1.00	0.97	P	2715.33	0.01	0.00	A	26.00	FXYD3	19q13.13
209008_x_at	1.00	0.97	P	3567.67	0.01	0.00	M,A	35.27	KRT8	12q13
242277_at	0.78	0.70	P	135.80	0.01	0.00	A	1.07	C6orf56	6q24.1
202718_at	0.99	0.94	P	324.30	0.01	0.00	A	2.90	IGFBP2	2q33-q34
224650_at	1.00	0.96	P	2044.17	0.01	0.00	A	17.10	MAL2	8q23
235698_at	0.99	0.94	P	201.47	0.01	0.00	A	2.10	ZFP90	16q22.1
202435_s_at	0.95	0.85	P	2117.97	0.01	0.00	M,A	21.17	CYP1B1	2p21
227642_at	1.00	0.98	P	703.43	0.01	0.00	A	7.27	TFCP2L1	2q14
231849_at	1.00	0.96	P	396.67	0.01	0.00	A	4.00	LOC144501	12q13.13
236562_at	0.98	0.89	P	109.63	0.01	0.00	A	1.13	ZNF439	19p13.2
202888_s_at	1.00	0.99	P	524.07	0.01	0.00	A	4.30	ANPEP	15q25-q26
208161_s_at	1.00	0.98	P	601.87	0.01	0.00	A	6.07	ABCC3	17q22
227758_at	1.00	0.95	P	760.67	0.01	0.00	A	7.40	RERG	12p13.1
205266_at	1.00	0.96	P	663.90	0.01	0.00	A	7.03	LIF	22q12.2
203060_s_at	1.00	0.99	P	814.10	0.01	0.00	A	8.80	PAPSS2	10q23-q24
208607_s_at	0.99	0.92	P	125.10	0.01	0.00	A	1.43	SAA2	11p15.1-p14
203238_s_at	0.98	0.91	P	1342.23	0.01	0.00	A	10.30	NOTCH3	19p13.2-p13.1
201787_at	1.00	0.97	P	727.37	0.01	0.00	A	6.13	FBLN1	22q13.31
202436_s_at	0.99	0.94	P	4243.40	0.01	0.00	M,A	48.73	CYP1B1	2p21
204942_s_at	0.98	0.91	P	1856.80	0.01	0.00	A	17.67	ALDH3B2	11q13
218261_at	1.00	0.99	P	1495.70	0.01	0.00	A	16.13	AP1M2	19p13.2
239148_at	1.00	0.99	P	372.87	0.01	0.00	A	3.30		
221645_s_at	1.00	0.95	P	424.87	0.01	0.00	A	3.20	ZNF83	19q13.3
239153_at	1.00	0.98	P	398.23	0.01	0.00	A	4.10		12q13.13
224579_at	1.00	0.96	P	1396.00	0.01	0.00	P	15.83	SLC38A1	12q13.11
1557014_a_at	1.00	0.97	P	130.63	0.01	0.00	A	1.47	C9orf122	9p12
220638_s_at	1.00	0.97	P,A	192.60	0.01	0.00	A	1.77	CBLC	19q13.2
201825_s_at	0.99	0.93	P	449.73	0.01	0.00	A	5.07	CGI-49	1q44
228067_at	1.00	0.99	P	213.70	0.01	0.00	A	2.17	MGC42367	2q11.2
221760_at	1.00	0.96	P	681.47	0.01	0.00	A	5.20	MAN1A1	6q22
227567_at	1.00	0.95	P	320.30	0.01	0.00	A	3.60		17
1559960_x_at	0.97	0.87	P,A	149.47	0.01	0.00	A	1.57		16q23.1
227875_at	1.00	0.96	P	251.13	0.01	0.00	A	2.73	KLHL13	xq23-q24
212143_s_at	0.95	0.85	P	695.60	0.01	0.00	A	7.47	IGFBP3	7p13-p12
202575_at	0.99	0.92	P	3350.03	0.01	0.00	A	38.13	CRABP2	1q21.3
37117_at	1.00	0.98	P	1063.93	0.01	0.00	A	11.03	ARHGAP8	22q13.31
210571_s_at	0.90	0.79	P	533.13	0.01	0.00	A	5.60	CMAH	6p21.32
229614_at	1.00	0.99	P	277.87	0.01	0.00	M,A	2.17	LOC162967	19q13.42
219995_s_at	1.00	0.99	P	258.13	0.01	0.00	A	2.73	FLJ13841	17q25.3
201508_at	0.99	0.94	P	414.77	0.01	0.00	A	4.87	IGFBP4	17q12-q21.1
216379_x_at	1.00	0.97	P	5013.30	0.01	0.00	P,M	60.20		
205487_s_at	0.99	0.92	P	465.40	0.01	0.00	A	4.20	VGLL1	xq26.3
222453_at	0.98	0.91	P	127.43	0.01	0.00	A	1.37	CYBRD1	2q31.1
228706_s_at	0.98	0.91	P	79.67	0.01	0.00	A	0.93	CLDN23	8p23.1
243818_at	1.00	0.97	P	211.47	0.01	0.00	A	2.53		
204363_at	0.97	0.88	P	1364.67	0.01	0.00	A	14.40	F3	1p22-p21

212151_at	1.00	0.97	P,A	877.27	0.01	0.00	A	10.33	PBX1	1q23
208791_at	1.00	0.98	P,A	198.77	0.01	0.00	A	2.50	CLU	8p21-p12
220192_x_at	1.00	0.95	P	1669.60	0.01	0.00	A	21.33	SPDEF	6p21.3
227971_at	0.99	0.94	P	580.37	0.01	0.00	P,A	6.83	NRK	xq22.3
1552797_s_at	1.00	0.97	P	1996.70	0.01	0.00	P,A	24.40	PROM2	2q11.2
201428_at	0.99	0.94	P	197.70	0.01	0.00	A	2.57	CLDN4	7q11.23
222242_s_at	0.99	0.95	P	3604.37	0.01	0.00	M,A	47.27	KLK5	19q13.3-q13.4
238441_at	0.99	0.94	P	107.67	0.01	0.00	A	1.23		
207419_s_at	0.94	0.83	P,A	192.67	0.01	0.00	A	2.63	RAC2	22q13.1
220289_s_at	0.99	0.92	P	120.73	0.01	0.00	A	1.60	AIM1L	1p35.3
238689_at	0.95	0.85	P	469.67	0.01	0.01	A	5.47	GPR110	6p21.1
224999_at	0.99	0.95	P	4971.30	0.01	0.00	A	67.27		
204733_at	0.99	0.94	P	2433.73	0.01	0.00	A	31.97	KLK6	19q13.3
1554195_a_at	1.00	0.98	P	394.60	0.01	0.00	A	5.57	MGC23985	5q32
231929_at	1.00	0.96	P,A	89.97	0.01	0.01	A	1.30		
206400_at	0.99	0.93	P	296.70	0.01	0.00	A	4.00	LGALS7	19q13.2
204049_s_at	0.98	0.89	P	686.23	0.01	0.00	A	8.77	PHACTR2	6q24.1
218186_at	1.00	0.97	P	1579.67	0.01	0.00	A	21.40	RAB25	1q22
240304_s_at	1.00	0.96	P	321.77	0.01	0.00	A	4.37		16p13.11
228523_at	1.00	0.99	P	1767.57	0.01	0.00	P,A	24.13	NANOS1	10q26.13
214598_at	1.00	1.00	P	1016.13	0.01	0.00	A	14.23	CLDN8	21q22.11
225792_at	1.00	0.97	P	524.63	0.01	0.01	A	7.70		
214920_at	1.00	0.99	P	265.77	0.01	0.01	A	3.20	LOC221981	7p21.3
204818_at	0.99	0.95	P	326.03	0.01	0.00	A	4.53	HSD17B2	16q24.1-q24.2
239350_at	0.98	0.90	P,A	141.20	0.01	0.00	A	2.13	MRVLDC3	16q22.2
232164_s_at	1.00	0.97	P	1051.33	0.01	0.00	A	14.67	EPPK1	8q24.3
227803_at	1.00	0.98	P	221.63	0.01	0.00	A	2.53	ENPP5	6p21.1-p11.2
211372_s_at	0.99	0.95	P,A	263.13	0.01	0.00	A	3.80	IL1R2	2q12-q22
228974_at	1.00	0.98	P	446.93	0.01	0.00	M,A	6.33	MGC48625	19q13.42
212446_s_at	1.00	0.97	P	530.03	0.01	0.01	P,A	7.73	LASS6	2q31.1
243161_x_at	1.00	0.99	P	1364.10	0.01	0.01	A	21.80	ZFP42	4q35.2
208146_s_at	0.99	0.92	P	503.57	0.01	0.00	A	7.53	CPVL	7p15-p14
205518_s_at	0.98	0.90	P	1138.63	0.01	0.00	A	15.83	CMAH	6p21.32
242463_x_at	1.00	0.96	P	325.83	0.01	0.00	A	4.50	ZNF611	19q13.42
229623_at	1.00	0.99	P	54.70	0.01	0.01	A	0.90		4q21.3
212442_s_at	1.00	0.96	P	1052.23	0.01	0.00	A	15.70	LASS6	2q31.1
222896_at	1.00	0.95	P	339.93	0.01	0.01	A	5.23	MGC3169	19p13.12
225355_at	1.00	0.99	P	763.70	0.01	0.00	A	11.50	DKFZP761M1511	5q35.2
203910_at	1.00	0.96	P	2269.37	0.01	0.00	P	33.87	PARG1	1p22.1
226869_at	0.99	0.95	P,M,A	153.07	0.01	0.01	A	2.60		
207002_s_at	1.00	0.97	P	194.80	0.01	0.01	A	3.20	PLAGL1	6q24-q25
238778_at	1.00	0.99	P	197.83	0.01	0.00	A	2.77	MPP7	10p12.1
207540_s_at	0.99	0.94	P,M	219.57	0.02	0.00	A	3.27	SYK	9q22
207069_s_at	0.99	0.92	P	301.67	0.02	0.00	A	4.70	SMAD6	15q21-q22
201288_at	1.00	0.96	P	1862.63	0.02	0.00	A	28.80	ARHGDIB	12p12.3
220414_at	0.98	0.90	P	640.70	0.02	0.00	A	9.83	CALML5	10p15.1
237061_at	0.98	0.91	P	65.63	0.02	0.00	A	0.97	ZNF347	19q13.42
210059_s_at	1.00	0.97	P	905.43	0.02	0.01	A	14.50	MAPK13	6p21.31
218995_s_at	1.00	0.96	P	801.37	0.02	0.00	A	12.17	EDN1	6p24.1
214614_at	0.95	0.85	P	191.67	0.02	0.00	A	2.97	HLXB9	7q36
222449_at	1.00	0.96	P	522.20	0.02	0.01	A	8.67	TMEPAI	20q13.31-q13.33
205403_at	1.00	0.99	P	553.90	0.02	0.01	A	9.37	IL1R2	2q12-q22
1556211_a_at	0.99	0.94	P	68.83	0.02	0.01	A	1.13		
222895_s_at	1.00	0.98	P	352.33	0.02	0.01	A	5.63	BCL11B	14q32.31
1556499_s_at	1.00	0.97	P	357.07	0.02	0.01	A	6.37	COL1A1	17q21.3-q22.1
203904_x_at	1.00	0.97	P	1292.70	0.02	0.00	A	20.37	KAI1	11p11.2
208779_x_at	1.00	1.00	P	3186.00	0.02	0.00	P	53.60	DDR1	6p21.3
211343_s_at	1.00	0.95	P	472.20	0.02	0.00	A	7.17	COL13A1	10q22
223843_at	0.99	0.94	P	88.43	0.02	0.00	A	1.40	SCARA3	8p21
219532_at	0.98	0.90	P	118.10	0.02	0.00	A	1.97	ELOVL4	6q14
232202_at	1.00	0.95	P	475.07	0.02	0.01	A	7.83		
224724_at	1.00	0.95	P	517.10	0.02	0.01	A	10.03	SULF2	20q12-q13.2
219109_at	1.00	0.98	P	725.07	0.02	0.00	A	10.83	PF20	2q35
218162_at	1.00	0.96	P	2272.13	0.02	0.00	A	37.50	OLFML3	1p13.1
213174_at	0.98	0.91	P	205.33	0.02	0.00	A	3.40	TTC9	14q24.1
217901_at	0.98	0.90	P	383.00	0.02	0.01	A	7.60	DSG2	18q12.1
209366_x_at	0.99	0.92	P	1255.77	0.02	0.01	A	22.67	CYB5	18q23
214203_s_at	0.99	0.93	P	893.97	0.02	0.00	A	15.03	PRODH	22q11.21
223374_s_at	1.00	0.96	P	419.27	0.02	0.00	A	6.90	B3GALT3	3q25
207717_s_at	0.98	0.90	P	427.17	0.02	0.01	A	6.73	PKP2	12p11
222168_at	1.00	0.97	P	489.43	0.02	0.00	A	7.47		
202917_s_at	1.00	0.96	P	1230.07	0.02	0.00	A	21.33	S100A8	1q21
1556300_s_at	1.00	0.97	P	123.03	0.02	0.01	A	2.20		
201425_at	1.00	0.98	P	362.53	0.02	0.01	A	6.33	ALDH2	12q24.2
212236_x_at	1.00	0.99	P	2776.80	0.02	0.00	A	45.77	KRT17	17q12-q21
216568_x_at	0.96	0.87	P	177.70	0.02	0.00	A	3.00	KRT8	12q13
230552_at	0.98	0.89	P	98.03	0.02	0.02	A	2.07	LOC284412	19q13.13
206295_at	1.00	0.96	P	252.60	0.02	0.01	A	3.97	IL18	11q22.2-q22.3
221666_s_at	1.00	0.98	P	521.23	0.02	0.02	A	10.53	ASC	16p12-p11.2
223315_at	0.99	0.93	P	1018.57	0.02	0.00	A	17.63	NTN4	12q22-q23
244741_s_at	0.98	0.90	P	413.90	0.02	0.01	A	7.80	MGC9913	19q13.43
208165_s_at	1.00	0.96	P	333.63	0.02	0.00	A	5.77	PRSS16	6p21
242354_at	0.99	0.94	P	672.83	0.02	0.00	A	11.67		
228600_x_at	0.99	0.93	P	290.20	0.02	0.00	A	5.13	MGC72075	7p15.3
203837_at	1.00	0.96	P	692.40	0.02	0.00	P,A	11.87	MAP3K5	6q22.33
231993_at	0.99	0.93	P	87.13	0.02	0.01	A	1.67		
209211_at	1.00	0.96	P	522.33	0.02	0.00	P,A	9.33	KLF5	13q21.33
208680_at	0.99	0.94	P	2247.03	0.02	0.00	A	38.57	PRDX1	1p34.1
223423_at	0.98	0.90	P	394.00	0.02	0.02	A	8.43	GPR160	3q26.2-q27
1553102_a_at	0.96	0.86	P	70.17	0.02	0.01	A	1.33	DKFZP434C171	5q33.1
203581_at	1.00	0.96	P	381.43	0.02	0.01	A	7.80	RAB4A	1q42-q43
1554438_at	0.98	0.90	P	66.23	0.02	0.00	A	1.20	KIAA1217	10p12.31
201850_at	1.00	0.98	P	1062.00	0.02	0.00	A	19.30	CAPG	2cen-q24
237493_at	0.97	0.89	P	96.67	0.02	0.00	A	1.73	IL22RA2	6q25.1
202016_at	1.00	0.96	P	1646.77	0.02	0.00	P,M	28.80	MEST	7q32
222847_s_at	0.99	0.94	P	174.67	0.02	0.00	A	3.13	EGLN3	14q13.1

218677_at	1.00	0.99	P	2084.53	0.02	0.00	M,A	36.33	S100A14	1q21.1
223748_at	0.99	0.94	P	400.97	0.02	0.02	A	8.30	SLC4A11	20p12
207076_s_at	1.00	0.99	P	3030.03	0.02	0.00	A	52.27	ASS	9q34.1
226961_at	1.00	0.97	P	253.93	0.02	0.00	A	4.50	LOC222171	7p15.1
1553696_s_at	0.99	0.95	P	135.67	0.02	0.00	A	2.43	ZNF569	19q13.13
225016_at	0.98	0.89	P	226.73	0.02	0.01	A	4.83	APCDD1	18p11.21
1553972_a_at	1.00	0.99	P	1087.57	0.02	0.00	A	20.50	CBS	21q22.3
233555_s_at	0.94	0.83	P	124.33	0.02	0.01	A	2.57	SULF2	20q12-q13.2
235779_at	1.00	0.98	P	85.63	0.02	0.01	A	1.67		
1557285_at	0.97	0.87	P	26.90	0.02	0.02	A	0.53	AREG	4q13-q21
210984_x_at	0.91	0.79	P	554.73	0.02	0.02	A	13.43	EGFR	7p12
208510_s_at	0.99	0.93	P	185.07	0.02	0.00	A	3.50	PPARG	3p25
226834_at	1.00	1.00	P	136.10	0.02	0.01	A	2.60	ASAM	11q24.1
202948_at	1.00	0.99	P,A	212.53	0.02	0.01	A	3.70	IL1R1	2q12
213085_s_at	1.00	0.96	P	1247.30	0.02	0.01	A	24.43	KIBRA	5q35.1
213423_x_at	0.98	0.90	P	925.17	0.02	0.01	A	17.60	TUSC3	8p22
206142_at	0.97	0.88	P,M	120.20	0.02	0.00	A	2.20	ZNF135	19q13.4
222862_s_at	1.00	0.96	P	225.93	0.02	0.00	A	4.17	AK5	1p31
236058_at	1.00	0.96	P,M	66.93	0.02	0.00	A	1.23	FLJ34633	1p35.3
220066_at	1.00	0.97	P	90.03	0.02	0.00	A	1.63	CARD15	16p12-q21
226610_at	1.00	0.98	P	220.10	0.02	0.00	A	4.03	p30	17p11.2
220030_at	0.95	0.85	P	48.27	0.02	0.00	A	0.93	STYK1	12p13.31
1552716_at	0.98	0.91	P	86.90	0.02	0.00	A	1.70	FLJ23577	5p13.2
204503_at	1.00	0.96	P	302.17	0.02	0.00	A	5.57	EVPL	17q25
239043_at	1.00	0.99	P	178.70	0.02	0.01	A	3.23	ZNF404	19q13.32
218445_at	0.99	0.93	P	359.80	0.02	0.00	A	7.10	H2AFY2	10q22
1562226_at	1.00	0.99	P,M,A	47.00	0.02	0.00	A	0.90	FLJ14712	7p21.3
224476_s_at	1.00	0.98	P,M	128.90	0.02	0.01	A	3.03	MESP1	15q26.1
230986_at	0.98	0.91	P	69.27	0.02	0.00	A	1.33		
226267_at	0.99	0.92	P	507.87	0.02	0.01	A	9.60	JDP2	14q24.3
229116_at	0.99	0.92	P	163.53	0.02	0.01	A	3.53	CNK2	Xp22.13
235651_at	1.00	0.97	P	234.10	0.02	0.00	A	4.47		
206827_s_at	1.00	0.98	P	168.37	0.02	0.00	A	3.17	TRPV6	7q33-q34
238718_at	1.00	0.95	P	353.10	0.02	0.01	A	7.13		
228303_at	0.98	0.91	P	144.30	0.02	0.00	A	2.77		
209173_at	1.00	0.96	P	193.47	0.02	0.00	A	3.80	AGR2	7p21.3
1553151_at	0.94	0.83	P	118.10	0.02	0.00	A	2.30	ATP6V0D2	8q21.13
202687_s_at	0.99	0.94	P	111.17	0.02	0.00	A	2.27	TNFSF10	3q26
212013_at	1.00	0.98	P	285.30	0.02	0.01	A	6.00	D2S448	2p25
222108_at	1.00	0.97	P	1134.13	0.02	0.00	A	21.67	AMIGO2	12q13.11
219587_at	1.00	0.97	P	110.70	0.02	0.00	A	2.13	TTC12	11q23.2
1560201_at	1.00	0.97	P	159.07	0.02	0.01	A	3.30	FLJ39963	7p11.2
228708_at	1.00	0.99	P	1239.40	0.02	0.00	A	25.60	RAB27B	18q21.2
216074_x_at	0.99	0.93	P,A	245.57	0.02	0.01	A	5.27	KIBRA	5q35.1
207011_s_at	0.99	0.92	P	509.77	0.02	0.00	A	10.37	PTK7	6p21.1-p12.2
215726_s_at	1.00	0.99	P	1108.37	0.02	0.00	A	21.40	CYB5	18q23
205728_at	1.00	0.97	P	154.63	0.02	0.01	A	3.47	SH2D1A	Xq25-q26
205768_s_at	1.00	0.98	P	173.60	0.02	0.00	A	3.50	SLC27A2	15q21.2
212012_at	0.98	0.90	P	1061.37	0.02	0.00	A	21.43	D2S448	2p25
235564_at	0.91	0.79	P	30.47	0.02	0.01	A	0.67	H-plk	7q11.21
229105_at	0.99	0.94	P	118.63	0.02	0.01	A	2.53	GPR39	2q21-q22
243198_at	0.99	0.94	P	105.07	0.02	0.01	A	2.37	LOC161577	15q21.2
221816_s_at	1.00	0.99	P	450.43	0.02	0.01	A	9.63	PHF11	13q14.12
1557094_at	0.99	0.94	P	331.27	0.02	0.01	A	6.53	ANXA8	10q11.2
228737_at	0.99	0.94	P,A	48.17	0.02	0.00	A	0.97	C20orf100	20q13.12
244740_at	1.00	0.96	P	571.77	0.02	0.00	A	11.77	MGC9913	19q13.43
202177_at	1.00	0.96	P	199.60	0.02	0.00	A	4.13	GAS6	13q34
222773_s_at	0.97	0.87	P	183.40	0.02	0.01	A	4.03	GALNT12	9q31.1
204964_s_at	0.97	0.88	P,A	209.17	0.02	0.01	A	4.93	SSPN	12p11.2
223435_s_at	1.00	0.95	P	104.17	0.02	0.00	A	2.13	PCDHA6	5q31
237923_at	1.00	0.97	P	58.27	0.02	0.00	A	1.20		
227070_at	1.00	0.96	P	190.40	0.02	0.01	A	4.40	LOC83468	12q
219429_at	0.96	0.87	P	724.13	0.02	0.00	P,A	15.07	FA2H	16q23
1556210_at	0.96	0.86	P	32.33	0.02	0.01	A	0.77		
203434_s_at	0.99	0.93	P,A	88.90	0.02	0.00	A	1.87	MME	3q25.1-q25.2
204990_s_at	0.99	0.92	P	314.30	0.02	0.00	A	6.57	ITGB4	17q11-qter
204730_at	1.00	0.98	P	330.00	0.02	0.00	A	7.17	RIMS3	1pter-p22.2
229390_at	1.00	0.96	P,A	68.80	0.02	0.00	A	1.43		
205093_at	1.00	1.00	P,A	179.93	0.02	0.00	A	3.83	PEPP3	1q32.1
201984_s_at	0.99	0.94	P	3189.83	0.02	0.00	A	67.40	EGFR	7p12
212816_s_at	0.99	0.94	P	597.37	0.02	0.01	A	14.27	CBS	21q22.3
205547_s_at	0.99	0.95	P	367.70	0.02	0.00	A	8.33	TAGLN	11q23.2
205979_at	0.99	0.94	P,M	50.30	0.02	0.00	A	1.17	SCGB2A1	11q13
1007_s_at	1.00	0.97	P	2839.73	0.02	0.00	A	60.83	DDR1	6p21.3
236255_at	1.00	0.98	P	100.60	0.02	0.00	A	2.17	KIAA1909	5p15.33
204679_at	1.00	0.99	P	558.97	0.02	0.01	A	13.57	KCNK1	1q42-q43
213240_s_at	1.00	0.97	P	450.43	0.02	0.00	A	9.77	KRT4	12q12-q13
203216_s_at	1.00	0.98	P	976.40	0.02	0.00	M,A	21.20	MYO6	6q13
229634_at	1.00	0.98	P	198.10	0.02	0.00	A	4.57	FLJ90586	7q35
205239_at	0.99	0.93	P	433.67	0.02	0.00	P,A	9.50	AREG	4q13-q21
226302_at	1.00	0.97	P	347.87	0.02	0.01	A	8.00	ATP8B1	18q21-q22
236448_at	1.00	0.98	P	442.60	0.02	0.00	A	9.87	UNC5A	5q35.3
1560779_a_at	1.00	0.99	P	98.87	0.02	0.01	A	2.33	LOC126167	19q13.13
201340_s_at	0.99	0.92	P	643.03	0.02	0.00	A	14.60	ENC1	5q12-q13.3
207802_at	1.00	0.96	P,A	55.53	0.02	0.00	A	1.27	CRISP3	6p12.3
204915_s_at	0.99	0.93	P	165.87	0.02	0.00	A	3.90	SOX11	2p25
211341_at	0.99	0.94	P	85.10	0.02	0.00	A	1.90	POU4F1	13q21.1-q22
1554897_s_at	0.99	0.94	P,A	145.87	0.02	0.00	A	3.27	RHBDL2	1p34.2
235027_at	0.99	0.94	P	103.73	0.02	0.00	A	2.40		
35148_at	1.00	0.97	P	290.73	0.02	0.00	A	6.57	TJP3	19p13.3
235955_at	1.00	0.98	P	47.90	0.02	0.02	A	1.33	MRVLDC2	5q13.1
215537_x_at	1.00	0.97	P	633.17	0.02	0.00	A	14.43	DDAH2	6p21.3
244224_x_at	0.94	0.84	P	252.80	0.02	0.02	A	6.77	LYNX1	8q24.3
218035_s_at	0.99	0.92	P	843.53	0.02	0.01	A	22.17	FLJ20273	4p13-p12
224189_x_at	0.99	0.92	P	195.30	0.02	0.01	A	4.77	EHF	11p12
204914_s_at	1.00	0.98	P	224.03	0.02	0.01	A	5.47	SOX11	2p25

205767_at	0.99	0.93	P	143.37	0.02	0.01	A	3.60	EREG	4q21.21
230746_s_at	1.00	0.97	P	530.37	0.02	0.00	M,A	12.63	STC1	8p21-p11.2
203186_s_at	0.99	0.94	P	1901.40	0.02	0.00	A	44.37	S100A4	1q21
1558217_at	1.00	0.96	P	418.00	0.02	0.01	P,A	10.97	FLJ31952	17q21.1
204885_s_at	0.99	0.94	P	61.03	0.02	0.01	A	1.53	MSLN	16p13.3
205328_at	1.00	0.96	P	1924.50	0.02	0.00	P	45.20	CLDN10	13q31-q34
1554593_s_at	0.98	0.90	P	629.83	0.02	0.00	A	14.67	SLC1A6	19p13.13
205016_at	1.00	0.98	P	619.97	0.02	0.01	A	16.70	TGFA	2p13
220169_at	1.00	0.96	P	177.47	0.02	0.02	A	5.27	FLJ23235	4p14
212886_at	0.99	0.92	P	45.00	0.02	0.00	A	1.10	DKFZP434C171	5q33.1
211368_s_at	1.00	0.98	P	128.90	0.02	0.00	A	3.00	CASP1	11q23
229245_at	1.00	0.96	P	215.83	0.02	0.01	A	6.03	PEPP3	1q32.1
227801_at	0.99	0.92	P	378.53	0.02	0.00	P,A	9.30	TSBF1	3q26.1
226863_at	1.00	0.97	P	447.33	0.02	0.01	A	12.07		
211689_s_at	0.94	0.83	P,M,A	165.43	0.02	0.01	A	4.83	TMPRSS2	21q22.3
242181_at	1.00	0.98	P	617.90	0.02	0.02	A	16.47		
244745_at	0.85	0.74	P	183.33	0.02	0.03	A	6.60	RERG	12p13.1
227088_at	0.99	0.93	P	131.60	0.02	0.01	A	3.43		4q27
235550_at	1.00	0.95	P	192.70	0.02	0.00	A	4.70		
225011_at	1.00	0.98	P	993.57	0.02	0.00	M,A	23.87	PRKAR2A	3p21.3-p21.2
239954_at	1.00	0.98	P	56.40	0.02	0.00	A	1.37	ZNF160	19q13.42
217875_s_at	1.00	0.98	P,A	142.90	0.02	0.00	A	3.50	TMEPAI	20q13.31-q13.33
228423_at	1.00	0.98	P	281.40	0.02	0.00	A	7.10		
207843_x_at	1.00	0.96	P	1270.80	0.02	0.00	M,A	31.40	CYB5	18q23
225525_at	1.00	0.97	P	776.20	0.02	0.02	A	23.00	KIAA1671	22q12
221885_at	1.00	0.96	P,M	79.83	0.02	0.00	A	1.93	KIAA1277	7q34
211607_x_at	0.85	0.73	P	411.50	0.02	0.00	A	10.37	EGFR	7p12
203423_at	0.99	0.93	P	673.07	0.02	0.01	A	18.47	RBP1	3q23
229159_at	1.00	0.95	P	108.73	0.02	0.02	A	2.90	KIAA0960	7p21.3
209459_s_at	0.96	0.87	P	34.97	0.02	0.01	A	0.97	ABAT	16p13.2
218960_at	1.00	0.99	P	120.13	0.02	0.00	A	3.00	TMPRSS4	11q23.3
207430_s_at	0.99	0.94	P,M,A	21.10	0.02	0.00	A	0.53	MSMB	10q11.2
219983_at	1.00	0.97	P	359.63	0.02	0.01	A	9.43	HRASLS	3q29
213172_at	1.00	0.96	P	172.67	0.03	0.02	A	5.03	TTC9	14q24.1
206658_at	1.00	0.99	P	183.03	0.03	0.00	A	4.57	UPK3B	7q11.2
236656_s_at	1.00	0.96	P	543.20	0.03	0.01	A	14.73		
222829_s_at	0.99	0.94	P,A	157.97	0.03	0.00	A	3.93	IL20RA	6q22.33-q23.1
204347_at	0.99	0.92	P	415.90	0.03	0.01	A	11.97	AK3	1p31.3
207090_x_at	1.00	0.98	P	201.97	0.03	0.02	A	5.97	ZFP30	19q13.13
238599_at	0.99	0.95	P	91.37	0.03	0.02	A	2.83	IRAK1BP1	6q14-q15
228910_at	1.00	0.95	P	656.93	0.03	0.00	A	16.90	KAI1	11p11.2
203954_x_at	1.00	0.97	P	412.37	0.03	0.01	A	12.07	CLDN3	7q11.23
214751_at	0.99	0.95	P	871.07	0.03	0.00	P,M	22.57	LOC90333	19q13.42
202005_at	1.00	0.99	P	771.57	0.03	0.01	A	20.73	ST14	11q24-q25
234970_at	0.96	0.87	P	82.73	0.03	0.00	A	2.10	MTAC2D1	14q32.12
205593_s_at	0.94	0.83	P	121.73	0.03	0.00	A	3.20	PDE9A	21q22.3
210749_x_at	1.00	0.98	P	2530.63	0.03	0.00	M,A	64.30	DDR1	6p21.3
204880_at	1.00	0.98	P	149.40	0.03	0.00	A	4.00	MGMT	10q26
243386_at	1.00	0.97	P	184.67	0.03	0.02	A	5.47		
217621_at	0.99	0.93	P	46.80	0.03	0.00	A	1.27		
1556097_at	1.00	0.97	P	36.37	0.03	0.00	A	0.97		
227341_at	1.00	0.99	P	91.70	0.03	0.00	A	2.47	C10orf30	10p14
204855_at	0.99	0.94	P	271.50	0.03	0.00	A	7.27	SERPINB5	18q21.3
238451_at	0.90	0.79	P	36.50	0.03	0.01	A	1.10	MPP7	10p12.1
213582_at	0.99	0.95	P	123.23	0.03	0.02	A	4.03	ATP11A	13q34
227252_at	1.00	0.96	P	342.57	0.03	0.00	A	9.27		
1553589_a_at	0.99	0.93	P	97.40	0.03	0.01	A	3.10	MAP17	1p33
1555724_s_at	0.99	0.94	P,A	360.43	0.03	0.00	A	9.80	TAGLN	11q23.2
210663_s_at	0.98	0.90	P	940.03	0.03	0.00	A	25.83	KYNU	2q22.3
230233_at	0.94	0.84	P	66.53	0.03	0.00	A	1.83	RASGEF1B	4q21.3
236429_at	0.99	0.94	P	124.67	0.03	0.01	A	3.73		
227177_at	0.99	0.94	P,A	190.53	0.03	0.00	A	5.37	CORO2A	9q22.3
224901_at	0.99	0.92	P	661.03	0.03	0.00	A	18.47	SCD4	4q21.3
226553_at	1.00	0.99	P,A	383.60	0.03	0.00	A	10.60	TMPRSS2	21q22.3
209493_at	1.00	0.95	P	306.50	0.03	0.01	A	9.80	PDZK3	5p13.3
212976_at	1.00	0.95	P	66.30	0.03	0.01	A	1.97	TA-LRRP	1p22.2
205490_x_at	1.00	0.98	P,A	313.93	0.03	0.00	A	9.30	GJB3	1p34
213441_x_at	0.99	0.92	P	1738.53	0.03	0.01	A	53.73	SPDEF	6p21.3
219768_at	1.00	0.98	P	578.83	0.03	0.00	A	16.93	B7-H4	1p12
235795_at	0.99	0.94	P	120.20	0.03	0.02	A	4.13	PAX6	11p13
203997_at	0.99	0.94	P	505.57	0.03	0.02	A	16.77	PTPN3	9q31
212978_at	1.00	0.97	P	503.83	0.03	0.01	A	16.67	TA-LRRP	1p22.2
202668_at	1.00	0.99	P	882.67	0.03	0.00	A	26.20	EFNB2	13q33
204200_s_at	0.97	0.88	P	182.10	0.03	0.01	A	5.97	PDGFB	22q13.1
209692_at	0.99	0.94	P	154.63	0.03	0.00	A	4.53	EYA2	20q13.1
216641_s_at	0.93	0.82	P,A	254.23	0.03	0.00	A	7.30	LAD1	1q25.1-q32.3
223681_s_at	1.00	0.97	P	671.17	0.03	0.00	P	19.40	INADL	1p32.1
203421_at	0.99	0.93	P,A	106.43	0.03	0.00	A	3.07	TP53I11	11p11.2
219395_at	1.00	0.97	P	363.93	0.03	0.01	A	11.10	FLJ21918	16q22.1
210511_s_at	0.99	0.92	P,M,A	90.87	0.03	0.00	A	2.77	INHBA	7p15-p13
209270_at	0.99	0.94	P	201.90	0.03	0.01	A	6.33	LAMB3	1q32
209228_x_at	0.99	0.92	P	606.90	0.03	0.02	A	22.03	TUSC3	8p22
225664_at	1.00	0.97	P	395.73	0.03	0.00	A	11.57	COL12A1	6q12-q13
203058_s_at	1.00	0.96	P	306.43	0.03	0.01	A	10.07	PAPSS2	10q23-q24
228302_x_at	0.99	0.94	P	111.40	0.03	0.00	A	3.37	CaMKIINalpha	1p36.13
241420_at	0.98	0.91	P	291.17	0.03	0.00	A	8.87		
205829_at	0.95	0.85	P	407.87	0.03	0.01	A	13.70	HSD17B1	17q11-q21
204160_s_at	0.97	0.89	P	258.77	0.03	0.02	A	9.13	ENPP4	6p21.1
214667_s_at	0.98	0.90	P	255.03	0.03	0.01	A	8.00	TP53I11	11p11.2
228411_at	1.00	0.98	P	241.10	0.03	0.01	A	7.57	ALS2CR19	2q33.3
204913_s_at	1.00	0.97	P	231.37	0.03	0.01	A	7.47	SOX11	2p25
1557389_at	0.97	0.89	P,A	189.37	0.03	0.01	A	6.13		
203896_s_at	0.97	0.87	P	86.47	0.03	0.01	A	2.83	PLCB4	20p12
233044_at	0.99	0.93	P,A	366.67	0.03	0.01	A	12.93		
215189_at	0.99	0.94	P	1582.03	0.03	0.00	P,A	48.53	KRTHB6	12q13
229901_at	1.00	0.99	P,M	179.03	0.03	0.00	A	5.70	ZNF488	10q11.22

205019_s_at	0.99	0.93	P	772.67	0.03	0.01	A	27.57	VIPR1	3p22
219181_at	0.99	0.92	P	706.40	0.03	0.00	A	22.43	LIPG	18q21.1
221087_s_at	1.00	1.00	P	147.13	0.03	0.00	A	4.70	APOL3	22q13.1
205309_at	0.98	0.91	P,A	49.80	0.03	0.00	A	1.57	SMPDL3B	1p35.2
207120_at	0.99	0.92	P	69.63	0.03	0.01	A	2.47	FLJ14011	19q13.43
230464_at	1.00	0.97	P	132.03	0.03	0.00	A	4.17	EDG8	19p13.2
201952_at	1.00	0.97	P	1105.60	0.03	0.00	P,A	35.03	ALCAM	3q13.1
209683_at	1.00	0.99	P	48.77	0.03	0.00	A	1.57	DKFZP566A1524	2p24.3
240041_at	0.99	0.92	P,A	67.00	0.03	0.02	A	2.60		
223500_at	0.99	0.95	P,A	48.37	0.03	0.00	A	1.57	CPLX1	4p16.3
211002_s_at	0.98	0.91	P	178.57	0.03	0.01	A	6.37	TRIM29	11q22-q23
209309_at	1.00	0.99	P	3334.17	0.03	0.00	P	106.83	AZGP1	7q22.1
209126_x_at	0.98	0.90	P	426.63	0.03	0.01	A	15.20	KRT6A	12q12-q13
204083_s_at	0.99	0.94	P	1835.97	0.03	0.00	P	60.33	TPM2	9p13.2-p13.1
222802_at	1.00	0.97	P	1171.97	0.03	0.00	A	39.17	EDN1	6p24.1
229341_at	0.92	0.80	P,A	121.27	0.03	0.00	A	4.03	TFCP2L1	2q14
219529_at	0.99	0.94	P,A	143.53	0.03	0.01	A	5.23	CLIC3	9q34.3
210150_s_at	0.98	0.90	P	501.10	0.03	0.00	A	17.07	LAMA5	20q13.2-q13.3
227475_at	1.00	0.96	P	1477.13	0.03	0.00	P,A	48.07	FOXQ1	6p25
238481_at	0.95	0.85	P	142.07	0.03	0.02	A	5.63	MGP	12p13.1-p12.3
227892_at	1.00	0.97	P	137.97	0.03	0.00	A	4.60		
1562235_s_at	1.00	0.96	P	55.23	0.03	0.01	A	2.10		
219905_at	0.99	0.94	P,A	90.00	0.03	0.00	A	3.17	ERMAP	1p34.1
206059_at	0.96	0.87	P	580.50	0.03	0.00	A	19.47	ZNF91	19p13.1-p12
210086_at	0.98	0.90	P	283.47	0.03	0.00	A	9.67	HR	8p21.2
230323_s_at	0.99	0.94	P	256.43	0.03	0.01	A	9.13	LOC120224	11q24.3
209691_s_at	1.00	0.96	P,A	83.20	0.03	0.01	A	2.93	DOK4	16q13
229964_at	1.00	0.98	P	689.57	0.03	0.00	P	23.17	C9orf152	9q32
1553883_at	0.89	0.77	P,A	11.50	0.03	0.02	A	0.47	C19orf9	19p13.11
203780_at	1.00	0.96	P	201.73	0.03	0.00	P,A	6.83	EVA1	11q24
203074_at	1.00	0.99	P	3264.13	0.03	0.00	A	111.70	ANXA8	10q11.2
218006_s_at	0.98	0.90	P	158.93	0.03	0.01	A	6.20	ZNF22	10q11
226959_at	1.00	0.95	P	174.83	0.03	0.00	A	5.97		
205048_s_at	1.00	0.96	P	4389.53	0.03	0.00	P	148.17	PSPH	7p15.2-p15.1
1554776_at	0.85	0.74	P,A	91.73	0.03	0.00	A	3.30	ZFP42	4q35.2
206276_at	0.98	0.89	P	167.10	0.03	0.00	A	5.70	LY6D	8q24-qter
232315_at	0.99	0.95	P	45.83	0.03	0.00	A	1.60	LOC400713	19q13.41
203895_at	1.00	0.97	P	268.47	0.03	0.00	A	9.43	PLCB4	20p12
240068_at	1.00	0.96	P,A	37.53	0.03	0.01	A	1.47	LOC284835	21q22.3
236279_at	0.99	0.92	P,M	108.60	0.03	0.00	A	3.73	FAM31C	19p13.3
227806_at	0.99	0.94	P	170.63	0.03	0.00	A	5.97		
227478_at	0.99	0.92	P	138.27	0.03	0.00	A	4.87	LOC284262	18q21.1
242070_at	0.99	0.91	P	182.87	0.04	0.00	A	6.57		19q13.13
227449_at	0.99	0.92	P	285.53	0.04	0.00	A	10.17	EPHA4	2q36.1
53991_at	1.00	0.97	P	239.33	0.04	0.00	A	8.57	KIAA1277	7q34
218718_at	1.00	0.99	P	549.80	0.04	0.01	A	20.57	PDGFC	4q32
204179_at	1.00	0.96	P	1256.37	0.04	0.00	P,A	45.77	MB	22q13.1
209373_at	1.00	0.99	P,M	465.13	0.04	0.01	A	19.30	BENE	2q13
202889_x_at	0.96	0.87	P,A	216.67	0.04	0.00	A	7.70	MAP7	6q23.2
218321_x_at	0.99	0.93	P	482.33	0.04	0.00	A	17.03	MK-STYX	7q11.23
209558_s_at	0.91	0.79	P,M,A	231.83	0.04	0.01	A	8.80	HIP1R	12q24
204678_s_at	0.99	0.94	P	120.13	0.04	0.00	A	4.30	KCNK1	1q42-q43
218451_at	0.97	0.89	P,A	298.80	0.04	0.00	A	10.50	CDCP1	3p21.32
232078_at	0.92	0.82	P,A	265.47	0.04	0.00	A	9.47	PVRL2	19q13.2-q13.4
212473_s_at	1.00	0.95	P	732.63	0.04	0.00	M,A	25.97	MICAL2	11p15.3
214823_at	0.99	0.94	P	208.60	0.04	0.00	A	7.70	ZNF204	6p21.3
222463_s_at	1.00	0.97	P	284.67	0.04	0.01	A	11.57	BACE1	11q23.2-q23.3
213929_at	1.00	0.97	P	454.60	0.04	0.00	P	17.13		
202504_at	1.00	0.95	P	1118.77	0.04	0.00	A	41.57	TRIM29	11q22-q23
209083_at	1.00	0.98	P	143.53	0.04	0.01	A	5.70	CORO1A	16p12.1
1554246_at	1.00	0.99	P	223.70	0.04	0.01	A	9.17	MGC52423	1p34.1
226988_s_at	0.99	0.93	P	299.00	0.04	0.02	A	14.00	MYH14	19q13.33
206122_at	1.00	0.99	P,A	130.67	0.04	0.00	A	4.80	SOX15	17p13
205155_s_at	1.00	0.96	P	133.10	0.04	0.00	A	4.83	SPTBN2	11q13
222173_s_at	1.00	0.96	P	139.03	0.04	0.01	A	5.57	TBC1D2	9q31.1
225536_at	1.00	0.99	P	797.03	0.04	0.00	A	29.07	BCLP	1p35-p34
1554592_a_at	0.94	0.83	P	335.10	0.04	0.00	A	12.60	SLC1A6	19p13.13
218922_s_at	0.94	0.82	P,M,A	51.73	0.04	0.00	A	1.93	LASS4	19p13.3
225987_at	1.00	0.99	P	1496.57	0.04	0.00	A	55.73	FLJ23153	7q21.13
235048_at	1.00	0.96	P,M	124.37	0.04	0.00	A	4.67	KIAA0888	5q13.3
227140_at	1.00	0.98	P	215.53	0.04	0.00	M,A	8.20		
244261_at	0.99	0.94	P,M,A	75.63	0.04	0.00	A	2.83	IL28RA	1p36.11
203453_at	1.00	0.96	P	835.67	0.04	0.00	A	31.27	SCNN1A	12p13
218086_at	0.99	0.93	P	320.53	0.04	0.00	A	12.30	NPDC1	9q34.3
213664_at	0.99	0.93	P	384.13	0.04	0.01	P,A	15.37	SLC1A1	9p24
206832_s_at	0.98	0.91	P,A	87.33	0.04	0.00	A	3.30	SEMA3F	3p21.3
222222_s_at	1.00	0.99	P,M,A	338.10	0.04	0.01	A	13.90	HOMER3	19p13.11
203438_at	0.99	0.92	P	178.63	0.04	0.00	A	6.80	STC2	5q35.2
235599_at	1.00	0.96	P,A	45.03	0.04	0.00	A	1.73	LOC339535	1q43
230578_at	0.98	0.90	P,A	42.53	0.04	0.00	A	1.63	ZNF471	19q13.43
228948_at	0.97	0.87	P,A	121.57	0.04	0.00	A	4.97	EPHA4	2q36.1
206043_s_at	0.99	0.95	P,A	218.70	0.04	0.00	A	8.77	KIAA0703	16q24.1
218180_s_at	1.00	0.97	P	234.50	0.04	0.01	A	10.07	EPS8L2	11p15.5
209269_s_at	0.95	0.85	P	67.13	0.04	0.00	A	2.77	SYK	9q22
225962_at	0.97	0.89	P,M	57.47	0.04	0.01	A	2.63	ZNRF1	16q22.3
212875_s_at	1.00	0.99	P	873.47	0.04	0.00	A	34.67	C21orf25	21q22.3
206048_at	1.00	0.98	P	69.00	0.04	0.00	A	2.77	ZNF339	20pter-q11.23
1554314_at	0.98	0.90	P	98.77	0.04	0.00	A	4.00	C6orf141	6p12.3
203582_s_at	0.97	0.88	P	263.73	0.04	0.00	A	10.57	RAB4A	1q42-q43
220979_s_at	0.99	0.92	P	573.87	0.04	0.00	P,A	22.90	SIAT7E	1p31.1
208116_s_at	0.89	0.78	P	144.37	0.04	0.01	A	6.30	MAN1A1	6q22
219874_at	0.99	0.94	P	87.77	0.04	0.01	A	3.80	SLC12A8	3q21.2
207109_at	0.99	0.93	P	47.77	0.04	0.01	A	2.13	POU2F3	11q23.3
202994_s_at	0.96	0.85	P,A	115.23	0.04	0.01	A	5.07	FBLN1	22q13.31
212531_at	0.99	0.95	P	637.97	0.04	0.00	A	25.60	LCN2	9q34
238513_at	0.98	0.91	P	939.97	0.04	0.00	P	37.53	TMG4	11p13

222240_s_at	1.00	0.98	P	662.73	0.04	0.00	A	27.33	ISYNA1	19p13.11
214046_at	1.00	0.99	P	362.77	0.04	0.00	A	15.17		
226706_at	0.97	0.87	P	237.67	0.04	0.02	P,A	10.93	FLJ23867	1q25.1
233025_at	0.98	0.89	P,A	56.40	0.04	0.01	A	2.50	PDZK3	5p13.3
236725_at	1.00	0.98	P	295.20	0.04	0.01	A	13.57	KIBRA	5q35.1
236657_at	0.98	0.90	P	207.67	0.04	0.02	A	9.97		
220318_at	0.99	0.93	P,A	124.83	0.04	0.00	A	5.13	EPN3	17q21.33
226586_at	0.96	0.86	P	331.67	0.04	0.00	A	13.93	SAMD6	9q31.1
209343_at	1.00	0.98	P	1680.70	0.04	0.00	A	70.47	EFHD1	2q37.1
219634_at	0.98	0.91	P	230.30	0.04	0.01	A	10.37	CHST11	12q
223895_s_at	1.00	0.98	P	252.63	0.04	0.01	A	11.73	EPN3	17q21.33
202800_at	1.00	0.99	P	619.30	0.04	0.00	A	25.80	SLC1A3	5p13
206382_s_at	0.99	0.95	P,A	90.10	0.04	0.02	A	4.57	BDNF	11p13
211812_s_at	0.96	0.87	P	114.50	0.04	0.01	A	5.27	B3GALT3	3q25
210480_s_at	0.97	0.87	P	142.73	0.04	0.00	A	6.17	MYO6	6q13
233982_x_at	0.98	0.91	P	397.43	0.04	0.01	A	18.43	MK-STYX	7q11.23
225373_at	0.99	0.94	P	201.37	0.04	0.01	A	9.23	PP2135	10q22.3
209679_s_at	1.00	0.96	P	497.20	0.04	0.02	A	24.17	LOC57228	12q13.13
213947_s_at	1.00	0.98	P,A	207.07	0.04	0.00	A	8.87	NUP210	3p25.2-p25.1
223779_at	1.00	0.95	P	876.10	0.04	0.00	P	37.90	MGC10981	4p16.1
213680_at	1.00	0.95	P	1259.47	0.04	0.00	A	54.70	KRT6B	12q12-q13
242054_s_at	0.99	0.91	P	30.53	0.04	0.00	A	1.37	SIX3	2p16-p21
213400_s_at	0.98	0.89	P,A	127.80	0.04	0.01	A	5.83	TBL1X	xp22.3
232370_at	0.96	0.87	P,A	70.43	0.04	0.00	A	3.10	LOC254057	3q21.3
232120_at	0.99	0.95	P	311.23	0.04	0.00	A	13.43		
237086_at	0.99	0.92	P	187.87	0.04	0.00	A	8.37	FOXA1	14q12-q13
236337_at	0.99	0.92	P	137.17	0.04	0.01	A	6.33	LOC221711	6p24.1
1559258_a_at	1.00	0.99	P	559.00	0.04	0.00	P	24.20		Xq24
222762_x_at	0.99	0.92	P	474.93	0.04	0.00	P,A	20.97	LIMD1	3p21.3
242915_at	1.00	0.96	P	158.83	0.04	0.00	A	6.93	LOC91120	19p13.11
206631_at	0.85	0.72	P,A	32.30	0.04	0.01	A	1.57	PTGER2	14q22
225841_at	1.00	0.98	P	632.60	0.04	0.00	P,A	28.17	FLJ30525	1p13.3
204856_at	1.00	0.97	P	325.17	0.04	0.01	A	14.93	B3GNT3	19p13.1
201510_at	0.98	0.90	P	254.13	0.04	0.01	A	12.43	ELF3	1q32.2
205258_at	0.95	0.84	P	625.33	0.04	0.00	A	28.10	INHBB	2cen-q13
208933_s_at	0.94	0.83	P	129.60	0.04	0.01	A	6.17	LGALS8	1q42-q43
209603_at	1.00	0.97	P,M,A	110.40	0.04	0.00	A	4.87	GATA3	10p15
208622_s_at	0.95	0.85	P	767.53	0.04	0.01	A	38.83	VIL2	6q25.2-q26
236302_at	1.00	0.97	P	251.07	0.04	0.01	A	11.83	PPM1E	17q23.2
227388_at	1.00	0.98	P	602.60	0.04	0.00	P,A	27.30	LOC389708	9p21.1
204048_s_at	0.98	0.90	P	587.53	0.05	0.00	A	26.67	PHACTR2	6q24.1
203021_at	1.00	0.97	P	1405.10	0.05	0.00	P,A	64.47	SLPI	20q12
200637_s_at	0.95	0.84	P	516.63	0.05	0.00	A	23.27	PTPRF	1p34
235924_at	1.00	0.95	P	243.17	0.05	0.01	A	12.20		
208373_s_at	1.00	0.97	P	348.57	0.05	0.01	A	16.27	P2RY6	11q13.5
200636_s_at	1.00	0.99	P	2002.50	0.05	0.00	A	92.40	PTPRF	1p34
224209_s_at	0.99	0.93	P	875.17	0.05	0.00	P,A	39.50	GDA	9q21.11-21.33
209604_s_at	1.00	0.99	P	1442.73	0.05	0.00	A	65.87	GATA3	10p15
238673_at	0.99	0.92	P	285.57	0.05	0.00	P,A	13.20		
202295_s_at	1.00	0.98	P	906.30	0.05	0.00	P,A	41.90	CTSH	15q24-q25
203528_at	1.00	0.97	P	148.90	0.05	0.01	A	7.37	SEMA4D	9q22-q31
226494_at	0.95	0.85	P,A	78.33	0.05	0.00	A	3.70	KIAA1543	19p13.3-p13.2
1554006_a_at	1.00	0.97	P	308.43	0.05	0.01	A	15.93	LLGL2	17q24-q25
219360_s_at	0.95	0.85	P	64.40	0.05	0.01	A	3.27	TRPM4	19q13.33
206140_at	0.99	0.95	P	56.73	0.05	0.00	A	2.63	LHX2	9q33-q34.1
1558280_s_at	1.00	0.98	P	239.67	0.05	0.00	A	11.47	PARG1	1p22.1
232417_x_at	1.00	0.96	P	93.27	0.05	0.01	A	4.60	ZDHHC11	5p15.33
200635_s_at	1.00	0.95	P	1001.37	0.05	0.00	A	48.83	PTPRF	1p34
213618_at	1.00	0.97	P	313.33	0.05	0.01	A	15.83	CENTD1	4p15.1
218921_at	0.96	0.86	P	150.20	0.05	0.02	A	8.60	SIGIRR	11p15.5
209318_x_at	1.00	0.97	P	206.10	0.05	0.01	A	10.77	PLAGL1	6q24-q25
209909_s_at	1.00	1.00	P	562.07	0.05	0.00	P	26.93	TGFB2	1q41
39248_at	1.00	0.99	P	330.33	0.05	0.00	P,A	16.17	AQP3	9p13
217014_s_at	1.00	0.97	P	1315.77	0.05	0.00	P	62.80	GJE1	7q22.1
239370_at	0.99	0.94	P	292.17	0.05	0.00	A	13.97		
210880_s_at	0.99	0.92	P,A	115.23	0.05	0.00	A	5.63	EFS	14q11.2-q12
231807_at	1.00	0.95	P	153.87	0.05	0.00	A	7.43	KIAA1217	10p12.31
202688_at	1.00	0.97	P	245.67	0.05	0.00	A	11.87	TNFSF10	3q26
231283_at	0.99	0.93	P	57.30	0.05	0.01	A	3.07	MGAT4A	2q12
204718_at	1.00	0.99	P	699.33	0.05	0.00	P,A	33.93	EPHB6	7q33-q35
230405_at	1.00	0.97	P,A	89.20	0.05	0.01	A	4.73		5q31.1
225688_s_at	1.00	0.98	P	427.67	0.05	0.00	P	20.87	PHLDB2	3q13.13
234722_x_at	0.99	0.94	P	160.13	0.05	0.01	A	8.90	OBP2B	9q34
204751_x_at	0.98	0.90	P	243.90	0.05	0.01	A	13.20	DSC2	18q12.1
238805_at	0.99	0.94	P	149.13	0.05	0.02	A	8.13	MGC14839	11q23.2
218810_at	0.99	0.95	P	359.60	0.05	0.01	A	18.40	FLJ23231	1p34.2
203713_s_at	1.00	0.97	P	382.27	0.05	0.00	A	18.87	LLGL2	17q24-q25
204971_at	1.00	1.00	P	439.97	0.05	0.00	P,A	21.83	CSTA	3q21
201842_s_at	1.00	0.99	P	345.37	0.05	0.00	A	17.13	EFEMP1	2p16
211719_x_at	0.99	0.92	P,A	102.73	0.05	0.00	A	5.20	FN1	2q34
1555862_s_at	0.96	0.87	P	118.73	0.05	0.02	A	6.70	FLJ23471	7p22.3
227598_at	1.00	0.97	P	96.83	0.05	0.00	A	4.87	C7orf29	7q36.1
218237_s_at	0.99	0.94	P	872.90	0.05	0.00	A	43.73	SLC38A1	12q13.11
226129_at	1.00	0.99	P	927.40	0.05	0.00	A	46.30	FLJ46072	8q24.3
208890_s_at	1.00	0.98	P	1613.50	0.05	0.00	A	80.37	PLXNB2	22q13.33
1552727_s_at	0.99	0.95	P	291.67	0.05	0.00	P,A	14.63		
226907_at	0.99	0.95	P,A	233.03	0.05	0.02	A	13.90	PPP1R14C	6q24.3-q25.3
204201_s_at	1.00	0.99	P	150.60	0.05	0.00	M,A	7.67	PTPN13	4q21.3
1566229_a_at	0.90	0.79	P,A	36.30	0.05	0.03	A	2.53		
209791_at	1.00	0.97	P	867.67	0.05	0.01	A	47.10	PADI2	1p35.2-p35.1
226548_at	0.99	0.93	P,A	64.37	0.05	0.01	A	3.60	LOC112868	16p12.1
205014_at	1.00	0.95	P	896.97	0.05	0.01	A	49.33	HBP17	4p16-p15
201117_s_at	0.99	0.95	P	48.23	0.05	0.01	A	2.67	CPE	4q32.3
218644_at	1.00	0.95	P	333.77	0.05	0.02	A	19.50	PLEK2	14q24.1
219522_at	0.99	0.93	P	818.50	0.05	0.00	A	41.67	FJX1	11p13
223624_at	0.98	0.90	P,A	45.60	0.05	0.00	A	2.43	ANUBL1	10q11.22

229975_at	0.99	0.94	P	99.53	0.05	0.01	A	5.53		
213122_at	1.00	0.98	P	397.70	0.05	0.01	A	22.00	TSPYL5	8q22.1
228716_at	0.99	0.95	P	145.90	0.05	0.01	A	8.30	THRB	3p24.3
222561_at	1.00	0.99	P	2576.67	0.05	0.00	P	133.30	LANCL2	7q31.1-q31.33
206799_at	0.98	0.89	P	91.93	0.05	0.00	A	4.73	SCGB1D2	11q13
226701_at	1.00	0.98	P	232.33	0.05	0.01	P,A	12.97	GJA5	1q21.1
208190_s_at	0.99	0.92	P	1192.20	0.05	0.00	P	61.93	LISCH7	19q13.13
242098_at	0.93	0.81	P	20.23	0.05	0.02	A	1.27	LOC202451	6q23.3
236646_at	0.99	0.94	P	162.27	0.05	0.00	A	8.53	FLJ31166	12p13.31
221011_s_at	0.98	0.91	P	280.13	0.05	0.02	A	18.13	LBH	2p23.3
205613_at	1.00	0.97	P	150.30	0.05	0.00	A	8.20	LOC51760	16p13.11
206277_at	0.95	0.84	P,A	68.97	0.05	0.00	A	3.70	P2RY2	11q13.5-q14.1
225618_at	1.00	1.00	P	99.90	0.05	0.01	A	5.63	LOC201176	17q21.31
220027_s_at	1.00	0.96	P	343.20	0.05	0.01	A	19.73	RASIP1	19q13.33
1555812_a_at	0.96	0.87	P	965.53	0.05	0.00	M,A	51.23	ARHGDIB	12p12.3
210004_at	0.96	0.86	P	226.80	0.05	0.00	P	12.70	OLR1	12p13.2-p12.3
222892_s_at	0.99	0.95	P	132.17	0.05	0.00	A	7.27	FLJ11036	3p25.2
223000_s_at	1.00	0.97	P	2249.20	0.05	0.00	P	119.97	F11R	1q21.2-q21.3
1569157_s_at	1.00	0.96	P	403.53	0.05	0.01	P,A	22.47	LOC162993	19p13.2
204381_at	0.97	0.88	P	115.00	0.05	0.02	A	7.20	LRP3	19q13.12
211434_s_at	1.00	0.97	P	124.30	0.05	0.01	A	7.17	CCRL2	3p21
205265_s_at	0.99	0.93	P	205.97	0.05	0.02	A	12.43	APEG1	2q36.1
209873_s_at	1.00	0.98	P	601.60	0.05	0.01	A	33.87	PKP3	11p15
230276_at	1.00	0.96	P,A	32.53	0.05	0.01	A	1.90	DKFZP566A1524	2p24.3
210367_s_at	0.99	0.94	P	674.73	0.05	0.00	P,A	36.33	PTGES	9q34.3
222496_s_at	0.96	0.87	P	327.53	0.05	0.01	A	18.77	FLJ20273	4p13-p12
227769_at	1.00	0.97	P	516.37	0.05	0.00	P,A	27.80	GPR27	3p21-p14
1556185_a_at	0.99	0.94	P	169.20	0.05	0.01	P,A	10.07		
203469_s_at	0.99	0.95	P	172.50	0.05	0.02	A	10.50	CDK10	16q24
219867_at	1.00	0.96	P	304.37	0.06	0.01	A	18.70	CHODL	21q11.2
234653_at	0.91	0.79	P	279.73	0.06	0.00	A	15.10		
204693_at	1.00	0.97	P	303.23	0.06	0.01	A	18.47	CDC42EP1	22q13.1
204268_at	0.99	0.95	P	873.57	0.06	0.00	A	47.63	S100A2	1q21
220646_s_at	0.99	0.93	P	145.90	0.06	0.00	A	8.13	KLRF1	12p12.3-13.2
217681_at	1.00	0.96	P	81.43	0.06	0.01	A	4.97	WNT7B	22q13
213285_at	0.99	0.92	P	356.23	0.06	0.00	A	19.90	LOC161291	14q23.1
231311_at	1.00	0.96	P	256.73	0.06	0.01	A	14.97		
201939_at	1.00	0.98	P	2445.73	0.06	0.00	P	136.10	PLK2	5q12.1-q13.2
228051_at	1.00	0.96	P	446.33	0.06	0.01	A	28.33	LOC202451	6q23.3
230630_at	1.00	0.98	P	631.20	0.06	0.00	A	35.77	AK3	9pter-p13
229530_at	0.99	0.94	P	185.90	0.06	0.00	P,A	10.77		
226299_at	1.00	0.99	P	131.33	0.06	0.00	A	7.40	PKN3	9q34.13
202995_s_at	0.96	0.86	P	289.27	0.06	0.02	P,A	19.00	FBLN1	22q13.31
225301_s_at	1.00	0.96	P	659.30	0.06	0.00	A	37.20	MYO5B	18q21
228346_at	0.99	0.94	P	191.73	0.06	0.00	A	10.83		
203256_at	0.98	0.91	P,A	217.17	0.06	0.00	A	12.50	CDH3	16q22.1
226611_s_at	1.00	0.97	P	400.83	0.06	0.00	A	22.87	p30	17p11.2
206595_at	1.00	0.97	P	360.90	0.06	0.00	A	20.67	CST6	11q13
203939_at	1.00	0.96	P	513.87	0.06	0.01	A	30.60	NT5E	6q14-q21
228846_at	0.99	0.94	P	389.13	0.06	0.00	A	22.23	MAD	2p13-p12
227963_at	1.00	0.97	P	48.67	0.06	0.01	A	3.03		
212654_at	1.00	0.99	P	49.63	0.06	0.00	A	2.97	TPM2	9p13.2-p13.1
204667_at	1.00	0.96	P	360.93	0.06	0.00	A	20.90	FOXA1	14q12-q13
227752_at	0.99	0.94	P,A	44.77	0.06	0.00	A	2.57		
206953_s_at	0.99	0.92	P	206.97	0.06	0.01	A	12.97	LPHN2	1p31.1
231775_at	1.00	0.97	P	255.33	0.06	0.02	A	16.43	TNFRSF10A	8p21
212472_at	1.00	0.96	P	419.33	0.06	0.00	A	25.13	MICAL2	11p15.3
225342_at	1.00	0.98	P	1840.57	0.06	0.00	P	105.87	AK3	9pter-p13
239623_at	1.00	0.99	P,A	40.43	0.06	0.00	A	2.37		5q23.3
211864_s_at	0.94	0.84	P	1479.10	0.06	0.00	P	86.20	FER1L3	10q24
241844_x_at	0.97	0.88	P	50.83	0.06	0.02	A	3.27	FLJ23235	4p14
240382_at	1.00	0.97	P	84.87	0.06	0.01	A	5.23		
204019_s_at	0.98	0.90	P	1271.07	0.06	0.00	P	75.57	SH3YL1	2p25.3
205466_s_at	0.93	0.82	P,A	27.60	0.06	0.00	A	1.63	HS3ST1	4p16
220187_at	0.95	0.84	P	69.20	0.06	0.02	A	4.80	FLJ23153	7q21.13
1555340_x_at	0.98	0.90	P	5523.40	0.06	0.00	P	327.30	RAP1A	1p13.3
204385_at	0.97	0.87	P	902.77	0.06	0.00	A	54.70	KYNU	2q22.3
227565_at	0.99	0.94	P	430.93	0.06	0.00	P	25.80		
210058_at	1.00	0.99	P	1168.40	0.06	0.00	P	70.23	MAPK13	6p21.31
201341_at	1.00	0.97	P	955.40	0.06	0.00	A	57.63	ENC1	5q12-q13.3
208119_s_at	1.00	0.97	P	334.90	0.06	0.00	A	20.33	ZNF505	19p13.11
226926_at	1.00	0.96	P	469.90	0.06	0.00	P	28.60	ZD52F10	19q13.13
209894_at	1.00	0.98	P	224.83	0.06	0.01	P	14.30	LEPR	1p31
218309_at	1.00	0.98	P	1013.90	0.06	0.00	P	61.70	CaMKIINalpha	1p36.13
206723_s_at	1.00	0.95	P	393.13	0.06	0.01	A	25.50	EDG4	19p12
242006_at	0.98	0.91	P,A	38.93	0.06	0.00	A	2.40	C6orf152	6q14.3
232117_at	0.90	0.79	P,A	18.27	0.06	0.02	A	1.37	ZNF471	19q13.43
204963_at	1.00	0.97	P,M,A	117.93	0.06	0.00	A	7.40	SSPN	12p11.2
220907_at	1.00	0.95	P	28.40	0.06	0.01	A	1.83	FLJ22684	6p21.1
232080_at	1.00	0.96	P	370.20	0.06	0.00	P,A	22.60	NEDL2	2q33.1
218219_s_at	1.00	0.97	P	5070.60	0.06	0.00	P	311.10	LANCL2	7q31.1-q31.33
232038_at	1.00	0.98	P	61.67	0.06	0.01	A	3.93	C6orf170	6q22.32
227753_at	1.00	0.96	P	591.53	0.06	0.00	A	37.33	FLJ90586	7q35
208009_s_at	0.99	0.94	P	305.50	0.06	0.01	A	20.07	ARHGEF16	1p36.3
210694_s_at	0.98	0.89	P	107.87	0.06	0.00	A	6.80	MID1	Xp22
209454_s_at	1.00	0.95	P	359.70	0.06	0.00	P	22.97	TEAD3	6p21.2
227370_at	1.00	0.97	P	555.03	0.06	0.00	P	34.90	KIAA1946	2q32.2
231577_s_at	1.00	0.98	P	635.47	0.06	0.00	P	40.60	GBP1	1p22.2
33323_r_at	0.99	0.95	P	3412.73	0.06	0.00	P	213.93	SFN	1p35.3
227345_at	1.00	0.96	P	105.57	0.06	0.02	A	7.37	TNFRSF10D	8p21
225000_at	1.00	0.97	P	917.00	0.06	0.00	A	60.27	PRKAR2A	3p21.3-p21.2
205253_at	1.00	0.97	P,M	57.70	0.06	0.00	A	3.67	PBX1	1q23
204348_s_at	1.00	0.97	P	1619.37	0.06	0.00	P,M	102.27	AK3	1p31.3
220149_at	1.00	0.99	P	653.33	0.06	0.00	P	41.63	FLJ22671	2q37.3
204287_at	0.97	0.87	P	197.00	0.06	0.01	A	13.00	SYNGR1	22q13.1
1555339_at	0.99	0.93	P	4658.70	0.06	0.00	P	300.83	RAP1A	1p13.3

219580_s_at	1.00	0.96	P	160.13	0.06	0.01	P,A	10.63	TMC5	16p13.11
207291_at	1.00	0.99	P	565.90	0.07	0.00	A	36.43	TMG4	11p13
201613_s_at	1.00	0.95	P	165.93	0.07	0.01	A	11.83	RUVBL1	3q21
204072_s_at	1.00	0.95	P	123.83	0.07	0.00	A	8.20	13CDNA73	13q12.3
214433_s_at	1.00	0.98	P	138.63	0.07	0.00	A	9.07	SELENBP1	1q21-q22
226382_at	1.00	0.99	P	295.67	0.07	0.00	A	19.67	LOC283070	10p14
235988_at	0.99	0.93	P	184.53	0.07	0.01	A	12.70	GPR110	6p21.1
212062_at	1.00	0.97	P	310.13	0.07	0.01	A	21.73	ATP9A	20q13.11-q13.2
211653_x_at	1.00	0.95	P	265.83	0.07	0.01	A	19.03	AKR1C1	10p15-p14
242919_at	0.99	0.93	P	209.27	0.07	0.01	M,A	14.60	LOC114977	19p13.11
201474_s_at	1.00	0.96	P	303.07	0.07	0.01	A	21.90	ITGA3	17q21.33
243585_at	1.00	0.99	P,A	112.90	0.07	0.01	A	7.83		
222890_at	1.00	0.96	P	101.83	0.07	0.01	P,A	7.07	HSPC065	16q13
229842_at	0.99	0.94	P	165.27	0.07	0.02	A	12.17	ELF3	1q32.2
227997_at	1.00	0.97	P	511.47	0.07	0.00	P,A	33.93		
223687_s_at	1.00	0.96	P	200.50	0.07	0.01	A	13.80	LY6K	8q24.3
202350_s_at	1.00	0.97	P	138.03	0.07	0.00	P,M	9.27	MATN2	8q22
232116_at	0.99	0.92	P,A	145.87	0.07	0.00	A	9.77	SOM	1p36.11
205990_s_at	1.00	0.96	P	267.47	0.07	0.00	A	18.37	WNT5A	3p21-p14
210697_at	0.99	0.92	P,M,A	53.93	0.07	0.01	A	3.90	ZNF257	19q13
1554777_at	0.92	0.81	P	133.77	0.07	0.00	A	9.13	ZFP42	4q35.2
226248_s_at	1.00	0.97	P	206.47	0.07	0.00	A	13.93	KIAA1324	1p13.3-p13.2
205470_s_at	0.99	0.94	P,A	59.83	0.07	0.01	A	4.47	KLK11	19q13.3-q13.4
218094_s_at	1.00	0.99	P	755.53	0.07	0.01	P,A	52.53	C20orf35	20q13.12
227629_at	1.00	0.97	P	221.77	0.07	0.01	P	16.47	PRLR	5p14-p13
209730_at	1.00	0.96	P	79.20	0.07	0.01	A	5.57	SEMA3F	3p21.3
214909_s_at	1.00	0.99	P	519.97	0.07	0.00	P,M	35.43	DDAH2	6p21.3
219388_at	0.98	0.90	P	943.30	0.07	0.01	P,M,A	67.80	TFCP2L3	8q22.3
204198_s_at	1.00	0.96	P	105.03	0.07	0.01	A	7.67	RUNX3	1p36
219630_at	0.98	0.91	P,A	128.17	0.07	0.01	A	9.43	MAP17	1p33
60474_at	0.99	0.94	P	212.13	0.07	0.01	A	15.13	C20orf42	20p12.3
230440_at	0.99	0.93	P	322.83	0.07	0.00	A	22.47	ZNF469	16q24
226185_at	1.00	0.97	P	529.33	0.07	0.00	P	37.90	CDS1	4q21.23
204304_s_at	1.00	0.98	P	564.13	0.07	0.00	P	39.00	PROM1	4p15.33
221709_s_at	0.99	0.94	P,A	67.23	0.07	0.00	A	4.67	C14orf131	14q32.33
52940_at	1.00	0.98	P	440.23	0.07	0.00	A	30.63	SIGIRR	11p15.5
222549_at	0.99	0.94	P	617.90	0.07	0.00	A	43.07	CLDN1	3q28-q29
226803_at	0.99	0.91	P	312.77	0.07	0.00	A	22.30	Shax3	8q21.13
223739_at	1.00	0.96	P	78.80	0.07	0.01	A	5.77	PADI1	1p36.13
215125_s_at	0.99	0.93	P	252.77	0.07	0.01	A	18.53	UGT1A10	2q37
227347_x_at	1.00	0.98	P	1136.83	0.07	0.00	P,A	79.43	Hes4	1p36.33
204343_at	0.99	0.94	P	77.10	0.07	0.02	A	5.87	ABCA3	16p13.3
214329_x_at	1.00	0.96	P	203.43	0.07	0.00	A	14.50	TNFSF10	3q26
227721_at	0.98	0.91	P	206.60	0.07	0.01	A	15.53	VIP	19p13.12
238017_at	0.99	0.94	P	114.80	0.07	0.00	A	8.10	RDH-E2	8q12.1
239657_x_at	0.99	0.93	P	375.40	0.07	0.00	A	26.67	FOXO6	1p34.1
232960_at	0.97	0.89	P,A	8.87	0.07	0.01	A	0.67	FLJ20130	Xq22.3
208006_at	0.99	0.94	P	493.77	0.07	0.00	A	35.93	FOXI1	5q34
212611_at	0.99	0.95	P	1507.10	0.07	0.00	P,A	108.30	DTX4	11q12.2
52975_at	1.00	0.95	P,A	159.57	0.07	0.00	A	11.67	C9orf28	9q34.12
239903_at	0.99	0.93	P	82.43	0.07	0.02	A	6.50		
227053_at	1.00	0.98	P	146.80	0.07	0.00	A	10.63	PACSIN1	6p21.3
230482_at	1.00	0.99	P	209.57	0.07	0.00	A	15.30	SIAT7E	1p31.1
228124_at	0.99	0.93	P	154.20	0.07	0.01	P,A	11.57	C20orf22	20p11.21
203725_at	0.99	0.92	P	692.80	0.07	0.01	P,A	51.70	GADD45A	1p31.2-p31.1
226817_at	1.00	0.99	P	775.47	0.07	0.00	P,M	56.67	DSC2	18q12.1
226226_at	1.00	0.97	P	347.83	0.07	0.01	A	27.03	LOC120224	11q24.3
205775_at	1.00	0.99	P	517.10	0.07	0.00	A	37.93	D6S2654E	6p25-pter
214858_at	1.00	0.95	P	63.73	0.07	0.02	A	5.13		
209696_at	1.00	0.97	P	363.97	0.07	0.00	A	26.87	FBP1	9q22.3
215842_s_at	0.97	0.87	P,A	70.17	0.07	0.02	A	5.70	ATP11A	13q34
201631_s_at	1.00	0.96	P	5885.63	0.07	0.00	P	433.00	IER3	6p21.3
222774_s_at	1.00	0.99	P	1000.47	0.07	0.00	P	73.57	NETO2	16q11
228360_at	1.00	0.97	P	286.93	0.07	0.01	A	21.63	LOC130576	2q23.3
213293_s_at	0.99	0.95	P	145.37	0.07	0.02	A	11.87	TRIM22	11p15
40093_at	0.96	0.87	P	197.33	0.07	0.00	A	14.53	LU	19q13.2
238493_at	1.00	0.98	P	137.60	0.07	0.00	P,A	10.17	ZNF506	19p13.11
205514_at	1.00	0.97	P	180.23	0.07	0.01	A	14.30	ZNF415	19q13.42
235304_at	0.99	0.92	P	60.00	0.07	0.01	A	4.73		
239853_at	0.99	0.93	P	130.33	0.08	0.02	A	10.80	KLC2L	19q13
225354_s_at	0.98	0.91	P	170.83	0.08	0.01	A	13.57	SH3BGRL2	6q13-15
221541_at	1.00	0.99	P	760.73	0.08	0.01	A	58.73	DKFZP434B044	16q24.1
219797_at	1.00	0.97	P	127.90	0.08	0.02	A	10.47	MGAT4A	2q12
1569241_a_at	0.98	0.90	P	45.20	0.08	0.01	A	3.57	ZNF505	19p13.11
223427_s_at	0.94	0.83	P	189.70	0.08	0.00	P	14.43	EPB41L4B	9q31-q32
235343_at	1.00	0.97	P	72.57	0.08	0.00	A	5.40	FLJ12505	1q32.3
228320_x_at	0.99	0.93	P,A	159.77	0.08	0.01	A	12.77	LOC92558	12q24.31
225258_at	1.00	0.95	P	763.87	0.08	0.01	A	61.10	FBLP-1	1p36.13
212339_at	0.99	0.92	P,A	134.97	0.08	0.02	A	11.73	EPB41L1	20q11.2-q12
231215_at	1.00	0.97	P	64.63	0.08	0.01	P,A	5.13		
220161_s_at	0.99	0.93	P	731.23	0.08	0.00	M,A	56.07	EPB41L4B	9q31-q32
239744_at	0.98	0.91	P	255.47	0.08	0.00	A	19.37		
204401_at	0.99	0.93	P	1332.23	0.08	0.00	A	103.30	KCNN4	19q13.2
226932_at	0.99	0.93	P	439.50	0.08	0.01	A	35.57	SSPN	12p11.2
221646_s_at	0.99	0.92	P	219.13	0.08	0.00	P,A	16.80		
226001_at	0.99	0.94	P	1500.07	0.08	0.00	P	114.90	KLHL5	4p14
218834_s_at	1.00	0.98	P	269.83	0.08	0.01	A	21.70	HSPA5BP1	11q12.2
209035_at	0.98	0.91	P	237.20	0.08	0.00	A	18.27	MDK	11p11.2
203687_at	0.94	0.82	P	275.70	0.08	0.02	A	23.63	CX3CL1	16q13
236616_at	0.99	0.94	P	205.40	0.08	0.00	A	15.97		
220177_s_at	1.00	0.99	P	230.43	0.08	0.01	A	18.60	TMPRSS3	21q22.3
211712_s_at	0.99	0.93	P,M	61.47	0.08	0.00	A	4.83	ANXA9	1q21
238983_at	0.99	0.94	P	63.93	0.08	0.01	A	5.23	FLJ14001	4p14
205759_s_at	1.00	0.98	P	378.97	0.08	0.00	A	29.23	SULT2B1	19q13.3
201578_at	1.00	0.96	P	310.03	0.08	0.00	A	24.30	PODXL	7q32-q33
241400_at	0.99	0.91	P	242.80	0.08	0.00	A	18.80		

229796_at	1.00	0.98	P,A	66.40	0.08	0.00	A	5.30		
240432_x_at	0.98	0.90	P	308.97	0.08	0.01	A	25.57		
205891_at	1.00	0.97	P	605.30	0.08	0.00	A	48.97	ADORA2B	17p12-p11.2
219411_at	0.99	0.94	P	240.60	0.08	0.01	A	20.57	ELMO3	16q22.1
225314_at	1.00	0.97	P	737.37	0.08	0.00	A	58.33	MGC45416	4p12
239250_at	1.00	0.99	P	245.27	0.08	0.01	P,A	20.13	ZNF542	19q13.43
222746_s_at	1.00	0.97	P	422.93	0.08	0.00	P,M	34.17	BSPRY	9q33.1
243998_at	0.99	0.92	P,A	10.87	0.08	0.01	A	0.90	MGC45562	17q21.2
232297_at	0.98	0.91	P	367.63	0.08	0.00	P	29.27	KLHL5	4p14
206722_s_at	0.99	0.92	P,A	292.80	0.08	0.02	A	25.73	EDG4	19p12
228121_at	0.99	0.93	P	1217.07	0.08	0.00	P	98.13	TGFB2	1q41
220197_at	1.00	0.97	P	317.77	0.08	0.00	A	26.30	ATP6V0A4	7q33-q34
218888_s_at	0.97	0.89	P	958.77	0.08	0.01	P	79.17	NETO2	16q11
1556090_at	1.00	0.98	P,A	32.27	0.08	0.01	A	2.73	HSMPP8	13q12.11
210827_s_at	1.00	0.96	P	522.87	0.08	0.00	A	42.93	ELF3	1q32.2
203126_at	0.99	0.94	P	2292.27	0.08	0.00	P	184.17	IMPA2	18p11.2
204720_s_at	1.00	0.98	P	528.37	0.08	0.00	P	42.63	DNAJC6	1pter-q31.3
237031_at	0.99	0.94	P,M,A	101.00	0.08	0.00	A	8.23		11q13.3
217593_at	1.00	0.99	P	156.07	0.08	0.01	A	13.03	FLJ12895	19q13.43
1567032_s_at	0.93	0.82	P,A	29.40	0.08	0.02	A	2.63	ZNF160	19q13.42
210026_s_at	1.00	0.99	P,M	289.33	0.08	0.01	A	24.50	CARD10	22q13.1
228221_at	1.00	0.96	P	387.57	0.08	0.00	A	31.70	MGC45474	1p22.1
218764_at	1.00	0.98	P	205.70	0.08	0.00	A	16.90	PRKCH	14q22-q23
203287_at	1.00	0.96	P	799.27	0.08	0.00	A	66.63	LAD1	1q25.1-q32.3
1555904_at	0.98	0.90	P,A	23.43	0.08	0.02	A	2.10	13CDNA73	13q12.3
229616_s_at	1.00	0.96	P	636.57	0.08	0.00	A	52.40	LOC196996	15q22.33
215471_s_at	0.97	0.87	P,A	247.23	0.08	0.02	A	23.37	MAP7	6q23.2
227492_at	0.98	0.90	P	579.87	0.08	0.00	P	48.57	OCLN	5q13.1
201242_s_at	1.00	0.96	P	2376.23	0.08	0.00	P	197.90	ATP1B1	1q24
230370_x_at	0.99	0.94	P	491.93	0.08	0.00	P,A	41.23	MK-STYX	7q11.23
214022_s_at	0.99	0.94	P	399.83	0.08	0.00	P	33.17	IFITM1	11p15.5
228153_at	0.99	0.95	P	1793.47	0.08	0.00	P	150.13	IBRDC2	6p22.3
235293_at	0.99	0.95	P	138.27	0.09	0.01	A	12.03		
217904_s_at	0.99	0.94	P	280.63	0.09	0.01	A	25.10	BACE1	11q23.2-q23.3
203365_s_at	0.99	0.93	P	151.87	0.09	0.00	A	12.83	MMP15	16q13-q21
235687_at	0.97	0.88	P,A	68.07	0.09	0.00	A	5.73	ZNF626	19p13.11
229638_at	1.00	0.98	P	2392.40	0.09	0.00	P	201.60	IRX3	16q12.2
213429_at	1.00	0.95	P,M	127.63	0.09	0.01	A	11.53		
207943_x_at	0.99	0.95	P	181.27	0.09	0.01	A	15.83	PLAGL1	6q24-q25
218005_at	1.00	0.98	P	481.80	0.09	0.00	A	40.77	ZNF22	10q11
226372_at	1.00	0.97	P	133.70	0.09	0.01	A	11.97	CHST11	12q
225822_at	1.00	0.98	P	681.87	0.09	0.00	A	59.20	MGC17299	1p34.1
228042_at	1.00	0.96	P,A	34.80	0.09	0.00	A	2.97	ADPRH	3q13.31-q13.33
1555173_at	0.98	0.89	P	193.83	0.09	0.01	A	16.87		
213424_at	0.99	0.92	P	213.40	0.09	0.00	P	18.40	KIAA0895	7p14.1
201798_s_at	1.00	0.96	P	2554.30	0.09	0.00	P	217.83	FER1L3	10q24
205769_at	1.00	0.97	P	183.13	0.09	0.00	A	16.03	SLC27A2	15q21.2
238570_at	0.99	0.95	P	114.00	0.09	0.01	A	10.57	ZNF83	19q13.3
244297_at	0.97	0.88	P	99.27	0.09	0.03	A	9.83	FLJ35740	9p12
208596_s_at	0.95	0.85	P	332.90	0.09	0.01	A	29.90	UGT1A10	2q37
231175_at	1.00	0.97	P	71.70	0.09	0.00	A	6.13	C6orf65	6p12.1
204952_at	0.99	0.93	P	709.80	0.09	0.00	A	62.83	C4.4A	19q13.32
219528_s_at	1.00	0.98	P	188.30	0.09	0.01	P,A	17.00	BCL11B	14q32.31
226069_at	1.00	0.97	P	85.20	0.09	0.02	A	8.20	PRICKLE1	12q12
205926_at	0.99	0.94	P	367.10	0.09	0.00	A	32.30	IL27RA	19p13.11
206712_at	0.99	0.92	P,A	41.43	0.09	0.00	A	3.60	GRTP1	13q34
206314_at	0.96	0.86	P,A	23.83	0.09	0.01	A	2.17	ZNF167	3p22.3-p21.1
58916_at	1.00	0.98	P	249.90	0.09	0.00	P	21.77	KCTD14	11q13.4
204597_x_at	1.00	0.99	P	204.77	0.09	0.01	A	18.70	STC1	8p21-p11.2
206272_at	1.00	0.97	P	189.77	0.09	0.00	P,A	17.00	SPHAR	1q42.11-q42.3
228391_at	1.00	0.95	P	214.70	0.09	0.00	P,A	18.83	CYP4V2	4q35.1
238827_at	0.96	0.86	P,A	170.53	0.09	0.00	A	15.00		
210002_at	1.00	0.99	P	149.93	0.09	0.01	A	13.70	GATA6	18q11.1-q11.2
243788_at	0.98	0.91	P,A	17.53	0.09	0.01	A	1.60		
203920_at	1.00	0.98	P	453.80	0.09	0.00	A	39.93	NR1H3	11p11.2
209546_s_at	0.99	0.93	P,A	70.17	0.09	0.01	A	6.47	APOL1	22q13.1
1557283_a_at	1.00	0.97	P	68.20	0.09	0.00	A	6.07	ZNF519	18p11.21
212859_x_at	1.00	0.99	P	1973.20	0.09	0.00	P	175.17	MT1E	16q13
232079_s_at	0.95	0.84	P	300.90	0.09	0.01	A	28.13	PVRL2	19q13.2-q13.4
226068_at	1.00	0.97	P	296.73	0.09	0.01	A	27.60	SYK	9q22
243582_at	1.00	0.97	P	265.13	0.09	0.00	A	23.67	SH3RF2	5q32
240457_at	1.00	0.96	P	35.33	0.09	0.02	A	3.43		
202552_s_at	1.00	0.98	P	606.40	0.09	0.00	P	55.00	CRIM1	2p21
241990_at	1.00	0.99	P	138.90	0.09	0.00	A	12.83	RHOV	15q13.3
232609_at	0.98	0.91	P	234.37	0.09	0.01	A	22.10	CRB3	19p13.3
1558888_x_at	0.95	0.83	P,A	24.40	0.09	0.00	A	2.20	MGC35402	19q13.42
212706_at	1.00	0.96	P	68.53	0.09	0.01	A	6.63	RASA4	7q22-q31.1
202768_at	1.00	0.99	P,A	82.93	0.09	0.00	A	7.47	FOSB	19q13.32
210261_at	0.93	0.82	P	106.67	0.09	0.02	A	10.40	KCNK2	1q41
227674_at	1.00	0.95	P	100.60	0.09	0.01	A	9.33	ZNF585A	19q13.13
1552648_a_at	1.00	0.97	P	102.70	0.09	0.00	A	9.30	TNFRSF10A	8p21
235648_at	0.99	0.94	P	261.57	0.09	0.00	P,A	24.40	ZNF567	19q13.13
225651_at	1.00	0.96	P	200.80	0.09	0.00	M,A	18.30	UBE2E2	3p24.2
223232_s_at	0.99	0.94	P,M,A	133.43	0.09	0.01	A	12.93	CGN	1q21
226755_at	0.97	0.88	P	134.87	0.09	0.02	A	13.47		1q32.2
229276_at	0.97	0.89	P,A	91.53	0.09	0.00	A	8.60	IGSF9	1q22-q23
1568838_at	1.00	0.98	P	102.50	0.09	0.01	P,A	10.30		
209212_s_at	0.99	0.93	P	348.60	0.09	0.00	A	33.10	KLF5	13q21.33
225001_at	0.99	0.95	P	1505.07	0.09	0.00	P,A	138.83	RAB3D	19p13.2
235463_s_at	1.00	0.99	P	194.43	0.09	0.00	P	18.00	LASS6	2q31.1
227834_at	0.99	0.92	P,A	13.67	0.09	0.01	A	1.30	C6orf198	6q23.3
242979_at	1.00	0.98	P	100.20	0.09	0.01	A	10.03		
204470_at	1.00	0.96	P	117.30	0.09	0.00	P,M	10.90	CXCL2	4q21
203817_at	1.00	0.96	P	326.00	0.09	0.01	A	32.27	GUCY1B3	4q31.3-q33
218611_at	1.00	0.99	P	2797.23	0.09	0.00	P	258.50	IER5	1q25.1
201462_at	1.00	0.97	P	1671.03	0.09	0.00	P	154.60	SCRN1	7p14.3-p14.1

223616_at	0.93	0.82	P,A	73.80	0.09	0.01	A	7.13	FLJ12644	19q13.41
230356_at	0.99	0.94	P	168.43	0.10	0.00	A	16.20		
57588_at	1.00	0.97	P	111.07	0.10	0.02	A	11.70	SLC24A3	20p13
207705_s_at	0.99	0.94	P	342.87	0.10	0.00	P,A	32.23	KIAA0980	20p11.22-p11.1
227998_at	1.00	0.96	P	4515.20	0.10	0.00	P	424.93	S100A16	
228082_at	0.88	0.75	P,A	42.90	0.10	0.01	A	4.37	ASAM	11q24.1
229657_at	1.00	0.98	P	186.50	0.10	0.00	A	17.80	THRB	3p24.3
222582_at	0.99	0.95	P	170.67	0.10	0.00	P	16.20	PRKAG2	7q35-q36
39966_at	1.00	1.00	P	235.83	0.10	0.00	P	22.70	CSPG5	3p21.3
203756_at	1.00	0.97	P,A	226.60	0.10	0.01	A	22.20	ARHGEF17	11q13.3
230774_at	1.00	0.98	P	151.43	0.10	0.00	P	14.83	ZADH1	14q24.2
218803_at	1.00	0.99	P	487.57	0.10	0.00	P	46.40	CHFR	12q24.33
226865_at	1.00	0.97	P	213.93	0.10	0.01	A	21.27		
236089_at	0.99	0.92	P	49.93	0.10	0.02	A	5.33		
223948_s_at	0.96	0.86	P,A	125.23	0.10	0.00	A	12.10	TMPRSS3	21q22.3
228256_s_at	1.00	0.98	P	224.93	0.10	0.00	A	21.87	TIGA1	5q21-q22
204151_x_at	1.00	0.98	P	447.50	0.10	0.01	P,A	45.27	AKR1C1	10p15-p14
221729_at	1.00	0.95	P	930.03	0.10	0.00	P	89.27	COL5A2	2q14-q32
213415_at	1.00	0.95	P,A	15.87	0.10	0.01	A	1.67	CLIC2	xq28
228625_at	1.00	0.96	P	422.13	0.10	0.00	A	40.87	CITED4	1p34.1
203962_s_at	0.99	0.92	P	195.47	0.10	0.01	A	19.47	NEBL	10p12
243539_at	0.99	0.91	P	317.00	0.10	0.00	P	30.33	KIAA1841	2q14
224746_at	1.00	0.98	P	827.47	0.10	0.00	A	80.47	KIAA1522	1p34.3
212560_at	1.00	0.99	P	1387.13	0.10	0.00	P	133.73	SORL1	11q23.2-q24.2
205933_at	1.00	0.97	P	121.63	0.10	0.01	A	12.00	SETBP1	18q21.1
202551_s_at	1.00	0.95	P	823.23	0.10	0.00	P	80.57	CRIM1	2p21
207949_s_at	1.00	0.96	P	250.30	0.10	0.01	A	25.87	ICA1	7p22
204484_at	0.99	0.93	P	567.90	0.10	0.01	A	60.30	PIK3C2B	1q32
223642_at	0.99	0.93	P	242.03	0.10	0.01	A	25.70	ZIC2	13q32
223709_s_at	0.99	0.94	P	389.13	0.10	0.00	P,A	38.20	WNT10A	2q35
230076_at	1.00	0.96	P	259.20	0.10	0.00	A	25.37	FLJ10156	17p13.2
219489_s_at	0.99	0.94	P	432.37	0.10	0.01	P	45.13	RHBDL2	1p34.2
202269_x_at	1.00	0.98	P	404.93	0.10	0.01	P	40.63	GBP1	1p22.2
227404_s_at	0.99	0.94	P	2528.97	0.10	0.00	P	250.33	EGR1	5q31.1
1560776_at	0.97	0.87	P	68.83	0.10	0.01	A	6.97		
221810_at	0.99	0.92	P	234.10	0.10	0.01	A	23.93	RAB15	14q23.3
221655_x_at	1.00	0.96	P,A	508.53	0.10	0.00	A	50.60	EPS8L1	19q13.42
209114_at	0.99	0.94	P,M,A	198.13	0.10	0.01	A	21.10	TSPAN-1	1p34.1
238846_at	1.00	0.97	P	228.53	0.10	0.00	P,M	23.00	TNFRSF11A	18q22.1
235176_at	1.00	0.96	P	134.80	0.10	0.01	A	13.80	ZNF545	19q13.13
228340_at	1.00	0.96	P	427.37	0.10	0.00	A	43.00	TLE3	15q22
227180_at	0.97	0.87	P	75.67	0.10	0.01	A	8.13	ELOVL7	5q12.1
228260_at	1.00	0.96	P	269.50	0.10	0.00	P,A	27.63	ELAVL2	9p21
227429_at	1.00	0.96	P	282.80	0.10	0.01	A	30.20	MGC45840	11p15.5
222136_x_at	1.00	0.97	P,M	163.07	0.10	0.01	A	17.47	ZNF43	19p13.1-p12
217025_s_at	0.99	0.92	P	380.17	0.10	0.01	P	39.27	DBN1	5q35.3
203317_at	0.99	0.95	P	120.30	0.10	0.02	A	13.53	TIC	2q13
214734_at	1.00	0.96	P	461.10	0.10	0.00	A	46.73	SLAC2-B	11q23.1
238688_at	0.99	0.93	P	380.63	0.10	0.02	A	41.43	TPM1	15q22.1
204112_s_at	0.99	0.94	P	972.70	0.10	0.00	P	98.97	HNMT	2q22.1
212299_at	0.98	0.90	P	216.33	0.10	0.02	A	23.27	NEK9	14q24.2
227342_s_at	1.00	0.98	P	508.03	0.10	0.00	A	51.73	MYEOV	11q13
1560570_a_at	0.94	0.83	P,A	4.37	0.10	0.01	A	0.47		
1563498_s_at	0.97	0.87	P	78.97	0.10	0.03	P,A	8.97	LOC283130	11q13.1
1552848_a_at	1.00	0.98	P	295.00	0.10	0.00	A	30.43	FLJ30296	xp22.13
1553995_a_at	0.98	0.89	P	149.77	0.10	0.00	A	15.60	NT5E	6q14-q21
206746_at	0.99	0.92	P	98.87	0.10	0.01	A	10.27	BFSP1	20p11.23-p12.1
217585_at	0.97	0.87	P	50.00	0.10	0.02	A	5.60	NEBL	10p12
223535_at	1.00	0.97	P	209.93	0.10	0.00	P	21.60	NUDT12	5q21.3
204044_at	1.00	0.97	P	381.00	0.10	0.01	A	39.97	QPRT	16p12.1
208623_s_at	1.00	0.99	P	2414.27	0.10	0.00	P	249.53	VIL2	6q25.2-q26
207574_s_at	0.99	0.95	P	508.87	0.10	0.00	P,A	52.23	GADD45B	19p13.3
1552477_a_at	0.99	0.93	P	113.73	0.10	0.00	A	11.73	IRF6	1q32.3-q41
218454_at	1.00	0.97	P	374.50	0.10	0.00	A	39.17	FLJ22662	12p13.2
1558508_a_at	1.00	0.95	P	299.40	0.11	0.00	P,A	31.30		1q31.3
230460_at	0.99	0.92	P,A	103.83	0.11	0.01	A	11.00		
219416_at	1.00	0.97	P,A	99.43	0.11	0.00	A	10.70	SCARA3	8p21
203476_at	0.99	0.95	P	2684.23	0.11	0.00	P	281.17	TPBG	6q14-q15
1568617_a_at	0.99	0.92	P	157.20	0.11	0.01	A	17.33	KIAA1543	19p13.3-p13.2
227692_at	0.98	0.90	P	52.57	0.11	0.02	A	6.07	GNAI1	7q21
219513_s_at	0.99	0.93	P	116.93	0.11	0.00	A	12.50	SH2D3A	19p13.3
202267_at	1.00	0.99	P	173.10	0.11	0.01	A	19.00	LAMC2	1q25-q31
206758_at	0.98	0.91	P,A	100.57	0.11	0.01	A	11.43	EDN2	1p34
203918_at	1.00	0.96	P	208.07	0.11	0.02	A	23.37	PCDH1	5q32-q33
227228_s_at	0.99	0.94	P	123.27	0.11	0.01	A	14.10	KIAA1509	14q32.12
233026_s_at	0.97	0.87	P,A	18.10	0.11	0.01	A	2.03	PDZK3	5p13.3
202270_at	1.00	0.97	P	440.97	0.11	0.00	P	46.73	GBP1	1p22.2
218552_at	1.00	0.98	P	331.77	0.11	0.00	A	35.40	FLJ10948	1p32.3
242350_s_at	1.00	0.96	P	280.30	0.11	0.01	M,A	32.00		
223432_at	0.99	0.93	P	202.77	0.11	0.01	A	22.37	OSBP2	22q12.2
219922_s_at	1.00	0.99	P	471.93	0.11	0.00	P,A	51.37	LTBP3	11q12
212314_at	1.00	0.96	P	191.00	0.11	0.01	A	21.60	KIAA0746	4p15.31
207126_x_at	1.00	0.97	P	458.80	0.11	0.01	P	51.33	UGT1A10	2q37
202525_at	1.00	0.98	P	332.03	0.11	0.00	A	36.23	PRSS8	16p11.2
235085_at	0.99	0.92	P	601.57	0.11	0.00	M,A	65.63	DKFZp761P0423	8p23.1
229053_at	1.00	0.97	P	71.47	0.11	0.02	A	8.33	LOC51760	16p13.11
211535_s_at	1.00	0.96	P	244.13	0.11	0.01	A	27.50	FGFR1	8p11.2-p11.1
1560285_at	0.98	0.90	P,A	40.70	0.11	0.01	A	4.77		
1552691_at	1.00	0.96	P	61.77	0.11	0.01	P,A	7.03	ARL11	13q14.12
208131_s_at	0.99	0.92	P	332.13	0.11	0.01	A	38.33	PTGIS	20q13.11-q13.13
236635_at	1.00	0.95	P	222.40	0.11	0.01	M,A	25.33	FLJ14011	19q13.43
205481_at	1.00	0.96	P	353.87	0.11	0.00	P,A	39.20	ADORA1	1q32.1
210397_at	0.98	0.89	P	170.10	0.11	0.01	A	19.97	DEFB1	8p23.2-p23.1
207722_s_at	0.99	0.92	P	165.73	0.11	0.01	A	18.77	BTBD2	19p13.3
218692_at	1.00	0.96	P	321.63	0.11	0.00	P,A	36.70	FLJ20366	8q23.2
226405_s_at	1.00	0.98	P	469.53	0.11	0.00	P	52.50	ARRDC1	9q34.3

213894_at	0.99	0.94	P	178.67	0.11	0.01	A	20.97	KIAA0960	7p21.3
220035_at	0.99	0.94	P	209.07	0.11	0.01	A	24.80	NUP210	3p25.2-p25.1
225418_at	1.00	0.97	P	533.80	0.11	0.00	M,A	60.27	PVRL2	19q13.2-q13.4
1559450_at	0.99	0.93	P,A	17.67	0.11	0.01	A	2.13		
91826_at	0.97	0.88	P	665.60	0.11	0.00	A	74.27	EPS8L1	19q13.42
228606_at	1.00	0.98	P	749.80	0.11	0.00	P	84.37	MGC33212	3q29
202262_x_at	0.99	0.95	P	595.47	0.11	0.00	A	67.60	DDAH2	6p21.3
204602_at	1.00	0.96	P	1138.87	0.11	0.00	P	130.00	DKK1	10q11.2
201286_at	1.00	0.97	P	606.37	0.11	0.00	P	68.83	SDC1	2p24.1
225129_at	1.00	0.97	P	434.27	0.11	0.00	P	49.27	CPNE2	16q13
202812_at	1.00	0.99	P	679.33	0.12	0.00	P	76.90	GAA	17q25.2-q25.3
203741_s_at	1.00	0.97	P	149.97	0.12	0.00	A	17.13	ADCY7	16q12-q13
1553155_x_at	0.98	0.91	P	390.07	0.12	0.01	P,A	45.70	ATP6V0D2	8q21.13
229534_at	0.99	0.95	P	218.40	0.12	0.00	M,A	25.23	PTE2B	14q24.2
1552849_at	0.99	0.94	P,A	62.73	0.12	0.01	A	7.70	LOC130951	2p13.1
243174_at	0.99	0.95	P	212.40	0.12	0.01	P	26.17		
228494_at	1.00	0.96	P	124.00	0.12	0.00	A	14.43		
207018_s_at	0.97	0.88	P	68.90	0.12	0.00	A	7.97	RAB27B	18q21.2
230534_at	0.97	0.88	P	82.83	0.12	0.02	A	10.03	MGC15634	1q42.13
228249_at	1.00	0.98	P	565.77	0.12	0.00	M,A	65.93	LOC119710	11p13
41469_at	1.00	0.98	P	283.63	0.12	0.00	A	33.07	PI3	20q12-q13
55583_at	1.00	0.95	P	233.50	0.12	0.00	A	27.40	DOCK6	19p13.2
1557458_s_at	1.00	0.97	P	641.03	0.12	0.00	P,A	74.40	SHB	9p12-p11
219517_at	1.00	0.97	P	511.67	0.12	0.01	P	60.60	ELL3	15q15.1
228062_at	0.99	0.95	P	82.13	0.12	0.01	A	10.00	NAP1L5	4q22.1
210973_s_at	0.93	0.82	P,M,A	170.40	0.12	0.02	A	21.50	FGFR1	8p11.2-p11.1
220296_at	0.94	0.82	P,A	167.47	0.12	0.02	A	20.57	GALNT10	5q33.2
227134_at	0.99	0.92	P	293.00	0.12	0.00	A	34.60	SYTL1	1p35.3
205428_s_at	1.00	0.97	P	346.93	0.12	0.01	A	42.00	CALB2	16q22.2
201596_x_at	1.00	0.98	P	6538.07	0.12	0.00	P	769.10	KRT18	12q13
233329_s_at	1.00	0.96	P	562.77	0.12	0.00	P	66.57	LOC51315	2p11.2
201243_s_at	1.00	0.99	P	1784.20	0.12	0.00	P	209.97	ATP1B1	1q24
218779_x_at	1.00	0.99	P,M	778.47	0.12	0.00	A	91.63	EPS8L1	19q13.42
238819_at	0.99	0.92	P	159.17	0.12	0.00	M,A	19.10	ZNF347	19q13.42
210674_s_at	1.00	0.96	P	60.40	0.12	0.02	A	7.50	PCDHAC2	5q31
204908_s_at	0.99	0.92	P	348.93	0.12	0.00	P,A	42.47	BCL3	19q13.1-q13.2
239847_at	1.00	0.98	P	256.40	0.12	0.00	P,M	30.30		
1560512_at	1.00	0.98	P	115.70	0.12	0.01	A	14.10		
244704_at	0.99	0.94	P	121.13	0.12	0.01	P,M,A	15.10	NFYB	12q22-q23
223194_s_at	1.00	0.97	P	420.10	0.12	0.01	A	51.43	C6orf85	6p25.2
201079_at	1.00	0.98	P	2432.40	0.12	0.00	P	289.97	SYNGR2	17q25.3
204202_at	0.99	0.94	P	374.03	0.12	0.01	P	45.67	KIAA1023	7p22.3
235141_at	0.98	0.89	P	379.27	0.12	0.00	A	45.10	MRVLDC2	5q13.1
242346_x_at	1.00	0.97	P	324.40	0.12	0.00	A	39.50		
202806_at	0.99	0.94	P	659.40	0.12	0.00	A	79.13	DBN1	5q35.3
212538_at	0.99	0.95	P	1225.03	0.12	0.00	P	146.80	DOCK9	13q32.3
217551_at	1.00	0.96	P	360.87	0.12	0.00	P,M	43.33		
212336_at	1.00	0.96	P	138.53	0.12	0.00	A	16.83	EPB41L1	20q11.2-q12
203085_s_at	1.00	0.97	P	164.37	0.12	0.00	A	19.93	TGFB1	19q13.1
219520_s_at	1.00	0.98	P	320.47	0.12	0.01	A	40.83	KIAA1280	xp22.32
35666_at	1.00	0.96	P	1180.47	0.12	0.00	P	141.83	SEMA3F	3p21.3
213996_at	1.00	0.96	P,M,A	94.13	0.12	0.01	A	11.60	YPEL1	22q11.2
205349_at	0.97	0.88	P,M	153.97	0.12	0.01	A	18.83	GNA15	19p13.3
221009_s_at	0.97	0.88	P,M,A	54.27	0.12	0.02	A	7.27	ANGPTL4	19p13.3
203468_at	0.99	0.92	P	129.00	0.12	0.02	P,A	16.57	CDK10	16q24
209357_at	0.99	0.94	P	1279.67	0.12	0.00	P	155.13	CITED2	6q23.3
203585_at	1.00	1.00	P	1128.57	0.12	0.01	P	140.77	ZNF185	xq28
223382_s_at	1.00	0.98	P	1345.07	0.12	0.00	P	162.63	ZNRF1	16q22.3
203020_at	1.00	0.96	P	561.73	0.12	0.00	P	68.07	HHL	1q24
206125_s_at	1.00	0.97	P	376.43	0.12	0.00	A	46.30	KLK8	19q13.3-q13.4
209163_at	1.00	0.98	P	889.53	0.12	0.00	A	108.20	CYB561	17q11-qter
1553994_at	1.00	0.98	P	170.27	0.12	0.01	M,A	21.70	NT5E	6q14-q21
205485_at	0.98	0.91	P	81.67	0.12	0.01	A	10.33	RYR1	19q13.1
213110_s_at	1.00	0.96	P	1003.20	0.12	0.00	P	122.97	COL4A5	xq22
232322_x_at	0.99	0.95	P	925.03	0.12	0.00	P,M	112.97	STARD10	11q13
202887_s_at	0.99	0.93	P	935.00	0.12	0.00	A	114.10	DDIT4	10pter-q26.12
236297_at	1.00	0.98	P	71.70	0.12	0.02	M,A	9.47	PLXDC2	10p12.33
210547_x_at	0.98	0.90	P	372.20	0.12	0.00	A	46.30	ICA1	7p22
209872_s_at	0.95	0.85	P,A	156.37	0.12	0.01	A	19.83	PKP3	11p15
221730_at	0.98	0.91	P	345.90	0.13	0.00	P,M,A	42.90	COL5A2	2q14-q32
222227_at	1.00	0.99	P	2210.77	0.13	0.00	P	276.20	ZNF236	18q22-q23
230610_at	0.99	0.93	P,A	55.67	0.13	0.00	A	6.93	MCLC	1p13.3
241726_at	0.98	0.89	P	99.57	0.13	0.00	P,M	12.50	HLCS	21q22.1
212444_at	1.00	0.95	P	675.20	0.13	0.01	P,A	85.50	RAI3	12p13-p12.3
210387_at	1.00	0.96	P	188.47	0.13	0.01	A	24.13	HIST1H2BG	6p21.3
222830_at	0.99	0.93	P	660.97	0.13	0.00	P	82.77	TFCP2L2	2p25.1
238466_at	0.99	0.94	P,A	15.73	0.13	0.00	A	1.97		
205150_s_at	1.00	0.95	P	161.30	0.13	0.00	P,A	20.33	KIAA0644	7p15.1
221942_s_at	1.00	0.96	P	408.63	0.13	0.00	A	51.23	GUCY1A3	4q31.1-q31.2
236769_at	0.97	0.88	P	42.23	0.13	0.02	A	5.80	LOC158402	9q32
1568720_at	0.98	0.91	P	166.23	0.13	0.00	P,M,A	21.07	ZNF506	19p13.11
241359_at	1.00	0.97	P	160.57	0.13	0.00	A	20.20		
204199_at	0.99	0.94	P	118.87	0.13	0.01	A	15.23	RALGPS1	9q34.13
236346_at	0.99	0.94	P	234.60	0.13	0.00	A	29.70		
221665_s_at	1.00	0.99	P	523.77	0.13	0.00	A	65.83	EPS8L1	19q13.42
226650_at	1.00	0.99	P	712.77	0.13	0.00	P	89.67	LOC90637	7p22.3
213307_at	0.99	0.95	P	259.50	0.13	0.00	P	33.20	SHANK2	11q13.2
225165_at	0.99	0.95	P	188.37	0.13	0.00	A	24.23	PPP1R1B	17q21.2
233035_at	1.00	0.96	P,A	31.27	0.13	0.00	A	3.97		
203477_at	0.98	0.91	P	154.70	0.13	0.00	A	19.93	COL15A1	9q21-q22
1555935_s_at	1.00	0.99	P	116.77	0.13	0.01	A	15.17	HUNK	21q22.1
214133_at	1.00	0.97	P	62.40	0.13	0.02	A	8.40	MUC6	11p15.5-p15.4
231778_at	1.00	0.96	P,A	117.37	0.13	0.00	A	15.27	DLX3	17q21
201709_s_at	1.00	0.97	P	1497.00	0.13	0.00	M,A	190.57	NIPSNAP1	22q12.2
220163_s_at	0.95	0.85	P	124.50	0.13	0.01	P,A	16.67	HR	8p21.2
226368_at	1.00	0.97	P	174.73	0.13	0.00	A	22.47	CHST11	12q

207980_s_at	0.96	0.87	P	621.20	0.13	0.00	P	80.33	CITED2	6q23.3
204656_at	1.00	0.99	P	390.80	0.13	0.00	A	50.10	MCART1	9p13.3-p12
210314_x_at	1.00	0.99	P	374.77	0.13	0.01	P	49.83	TNFSF13	17p13.1
222312_s_at	0.99	0.94	P	343.60	0.13	0.00	A	44.23		
221664_s_at	1.00	0.97	P	680.13	0.13	0.00	A	87.97	F11R	1q21.2-q21.3
1553105_s_at	0.93	0.82	P,A	153.67	0.13	0.02	A	21.63	DSG2	18q12.1
226145_s_at	1.00	0.96	P	160.10	0.13	0.00	P,M,A	20.73	FRAS1	4q21.21
1552546_a_at	0.98	0.90	P	137.93	0.13	0.01	A	18.67	FLJ25409	8p11.22
218373_at	1.00	0.96	P	837.83	0.13	0.01	P	111.80	FTS	16q12.2
242903_at	1.00	0.97	P	167.63	0.13	0.01	P,A	22.30	IFNGR1	6q23-q24
209125_at	0.98	0.90	P	113.83	0.13	0.00	A	15.03	KRT6A	12q12-q13
218844_at	1.00	0.96	P,M	131.70	0.13	0.01	A	17.87	FLJ20920	17q21.33
219308_s_at	1.00	0.95	P	193.20	0.13	0.00	A	25.67	AK5	1p31
205455_at	1.00	0.97	P	237.33	0.13	0.00	P,A	31.67	MST1R	3p21.3
226187_at	0.98	0.91	P,M	235.13	0.13	0.01	A	32.10	CDS1	4q21.23
33322_i_at	1.00	0.97	P	4120.80	0.13	0.00	P	544.97	SFN	1p35.3
203323_at	0.99	0.92	P	1753.70	0.13	0.00	P	233.97	CAV2	7q31.1
223471_at	1.00	0.96	P	322.00	0.14	0.01	P	43.90	RAB3IP	12q14.3
221698_s_at	0.95	0.84	P	108.83	0.14	0.02	A	16.00	CLECSF12	12p13.2-p12.3
203108_at	1.00	0.96	P	717.07	0.14	0.00	A	95.73	RAI3	12p13-p12.3
243410_at	0.99	0.93	P	83.30	0.14	0.01	A	11.47		
205194_at	1.00	0.99	P	2194.50	0.14	0.00	P	294.20	PSPH	7p15.2-p15.1
212769_at	0.99	0.92	P	312.97	0.14	0.00	P,A	41.87	TLE3	15q22
1569107_s_at	0.99	0.95	P	91.70	0.14	0.02	A	13.00	FLJ16030	1p34.2
206472_s_at	1.00	0.98	P	531.63	0.14	0.00	P	72.27	TLE3	15q22
227985_at	1.00	0.96	P	223.57	0.14	0.00	A	30.80		
235515_at	0.99	0.93	P	307.03	0.14	0.01	A	43.47	FLJ36445	19q13.13
220959_s_at	1.00	0.95	P	85.67	0.14	0.02	A	12.37	OBP2A	9q34
226275_at	1.00	0.97	P	404.73	0.14	0.00	P,M,A	54.97	MAD	2p13-p12
203498_at	0.98	0.90	P	186.07	0.14	0.00	P	25.33	DSCR1L1	6p21.1
204647_at	1.00	0.97	P	2333.70	0.14	0.00	P	315.70	HOMER3	19p13.11
235121_at	0.99	0.92	P	237.13	0.14	0.00	P	32.83	ZNF542	19q13.43
235247_at	1.00	0.97	P	152.73	0.14	0.00	A	20.87		
224694_at	0.99	0.94	P	440.97	0.14	0.00	P	60.77	ANTXR1	2p13.1
207394_at	1.00	0.96	P	109.80	0.14	0.01	A	15.77	ZNF137	19q13.4
212647_at	0.98	0.91	P,A	84.33	0.14	0.00	A	11.60	RRAS	19q13.3-qter
201289_at	1.00	0.97	P	2212.00	0.14	0.00	P	301.53	CYR61	1p31-p22
202510_s_at	1.00	0.96	P	424.43	0.14	0.00	M,A	57.90	TNFAIP2	14q32
210764_s_at	0.97	0.88	P	876.13	0.14	0.00	A	121.97	CYR61	1p31-p22
205749_at	0.98	0.90	P	194.87	0.14	0.02	A	28.40	CYP1A1	15q22-q24
205640_at	0.99	0.93	P,A	51.90	0.14	0.01	A	7.37	ALDH3B1	11q13
217066_s_at	0.98	0.89	P,A	75.47	0.14	0.01	A	10.77	DMPK	19q13.3
204985_s_at	0.99	0.95	P	230.37	0.14	0.00	A	31.80	MGC2650	19q13.32
230273_at	0.94	0.84	P,A	31.43	0.14	0.02	A	4.70	C6orf165	6q15
224837_at	0.99	0.91	P	429.23	0.14	0.00	P,A	59.37	FOXP1	3p14.1
230766_at	1.00	0.97	P	230.57	0.14	0.01	P	33.27		
206094_x_at	1.00	0.97	P,M	332.37	0.14	0.00	P,A	46.97	UGT1A6	2q37
202035_s_at	0.99	0.94	P	79.97	0.14	0.01	P,A	11.47	SFRP1	8p12-p11.1
238168_at	0.98	0.90	P	54.83	0.14	0.03	A	8.43	TM4SF1	3q21-q25
1554640_at	0.98	0.89	P	82.10	0.14	0.02	A	12.20	PALM2	9q31-q33
215532_x_at	0.99	0.94	P	70.20	0.14	0.00	P,M	10.03	ZNF492	19p13.11
1556212_x_at	0.99	0.92	P	38.93	0.14	0.01	A	5.60		
242417_at	1.00	0.95	P	243.07	0.14	0.00	A	34.47	LOC283278	11p15.2
203027_s_at	0.99	0.94	P	238.93	0.14	0.00	P,A	34.03	MVD	16q24.3
232353_s_at	0.98	0.90	P	630.40	0.14	0.01	M,A	92.73	MK-STYX	7q11.23
226285_at	0.99	0.94	P	793.80	0.14	0.00	P	111.50	M11S1	11p13
204341_at	0.99	0.93	P	608.50	0.14	0.00	A	85.33	TRIM16	17p11.2
210986_s_at	1.00	0.99	P	4539.73	0.14	0.00	P	634.17	TPM1	15q22.1
214721_x_at	1.00	0.99	P	772.53	0.14	0.00	P	109.13	CDC42EP4	17q24-q25
206561_s_at	1.00	0.97	P	355.63	0.14	0.00	A	50.20	AKR1B10	7q33
201466_s_at	0.99	0.91	P	231.87	0.14	0.00	P	32.57	JUN	1p32-p31
206346_at	0.99	0.92	P	48.87	0.14	0.02	P,A	7.40	PRLR	5p14-p13
40837_at	0.99	0.94	P	535.20	0.14	0.02	A	79.53	TLE2	19p13.3
243885_x_at	0.98	0.90	P	45.60	0.14	0.00	P,A	6.37	EFG1	3q25.1-q26.2
216061_x_at	0.98	0.91	P	260.13	0.14	0.01	M,A	38.37	PDGFB	22q13.1
1552319_a_at	0.95	0.85	P	57.87	0.14	0.01	A	8.40	KLK8	19q13.3-q13.4
204681_s_at	0.99	0.94	P	188.37	0.14	0.00	P,A	26.87	RAPGEF5	7p15.3
219749_at	1.00	0.97	P	232.67	0.14	0.01	A	33.30	SH2D4A	8p21.2
227052_at	1.00	0.99	P	580.83	0.14	0.00	P	82.47		
35147_at	0.98	0.90	P	657.67	0.14	0.01	A	94.07	MCF2L	13q34
228596_at	0.98	0.91	P	267.43	0.14	0.00	A	37.77	ARHGEF5	7q33-q35
218982_s_at	1.00	0.99	P	3768.53	0.14	0.00	P	533.10	MRPS17	7p11
31845_at	1.00	0.95	P	482.10	0.14	0.00	P	68.43	ELF4	xq26
244780_at	0.99	0.94	P	90.57	0.14	0.02	A	14.03	SGPP2	2q36.1
207847_s_at	0.99	0.92	P	638.27	0.14	0.01	P,M,A	94.07	MUC1	1q21
229801_at	1.00	0.98	P	296.83	0.14	0.01	A	42.73	C10orf47	10p14
221794_at	1.00	0.97	P	233.40	0.14	0.00	A	33.47	DOCK6	19p13.2
235957_at	0.99	0.94	P	71.53	0.14	0.02	A	10.77		
216268_s_at	1.00	0.97	P	433.97	0.15	0.00	P	62.03	JAG1	20p12.1-p11.23
205289_at	0.97	0.89	P	106.90	0.15	0.00	A	15.37	BMP2	20p12
219501_at	0.99	0.95	P	140.33	0.15	0.01	A	20.50	FLJ10094	13q14.11
214807_at	1.00	0.96	P	106.47	0.15	0.00	A	15.43		
213780_at	0.99	0.94	P	82.67	0.15	0.01	A	12.50		
235953_at	1.00	0.95	P	116.67	0.15	0.02	P,M,A	18.23	ZNF610	19q13.41
228496_s_at	0.98	0.89	P	706.23	0.15	0.00	P	102.33	CRIM1	2p21
201668_x_at	0.95	0.85	P	824.30	0.15	0.00	P	119.47	MARCKS	6q22.2
218128_at	0.99	0.95	P	418.70	0.15	0.00	P	60.83	NFYB	12q22-q23
237489_at	1.00	0.99	P	35.10	0.15	0.01	A	5.27		
226425_at	1.00	0.95	P	148.23	0.15	0.01	A	22.07	FLJ21069	2p23.3
208711_s_at	1.00	0.98	P	962.20	0.15	0.00	P	139.20	CCND1	11q13
214912_at	0.98	0.89	P	67.77	0.15	0.02	A	10.63		
212327_at	0.98	0.91	P	632.00	0.15	0.00	P	92.27	KIAA1102	4p14
242045_at	1.00	0.96	P	88.80	0.15	0.01	A	13.40	LOC348840	3q29
221614_s_at	1.00	0.97	P	145.17	0.15	0.00	P	21.07	RPH3AL	17p13.3
202704_at	1.00	0.97	P	1944.90	0.15	0.00	P	284.50	TOB1	17q21
233687_s_at	1.00	0.97	P	103.83	0.15	0.01	A	15.30	KLK9	19q13.41

1553684_at	0.99	0.92	P	69.00	0.15	0.00	A	10.20	PPIL6	6q21
223665_at	1.00	0.98	P	44.70	0.15	0.01	A	6.70	ARPM1	3q26.31
228975_at	0.99	0.93	P	288.33	0.15	0.01	P,A	44.27	SP6	17q21.32
1560792_at	1.00	0.95	P	164.03	0.15	0.00	P,A	24.40	IER5	1q25.1
205743_at	1.00	0.97	P	531.27	0.15	0.00	A	78.27	STAC	3p22.3
225406_at	0.99	0.94	P	1311.47	0.15	0.00	P	193.60	TWSG1	18p11.3
200878_at	0.99	0.93	P	899.97	0.15	0.00	P	131.93	EPAS1	2p21-p16
206029_at	0.99	0.92	P,M,A	45.90	0.15	0.02	A	7.17	ANKRD1	10q23.33
1563022_at	0.99	0.92	P	101.30	0.15	0.00	A	15.27		Xq26.3
217080_s_at	0.99	0.93	P	156.33	0.15	0.00	A	23.43	HOMER2	15q24.3
227417_at	1.00	0.96	P	207.97	0.15	0.00	A	31.43	FLJ20605	1q42.11
225897_at	0.99	0.93	P	1566.27	0.15	0.00	P	232.47	MARCKS	6q22.2
226875_at	1.00	0.95	P	125.23	0.15	0.01	A	19.33	DOCK11	xq24
239231_at	1.00	0.99	P	758.77	0.15	0.00	P	112.37		
226176_s_at	1.00	0.97	P	182.30	0.15	0.00	P,A	27.10	USP42	7p22.2
219615_s_at	0.98	0.91	P	181.30	0.15	0.00	P,A	27.20	KCNK5	6p21
218303_x_at	1.00	0.98	P	1103.70	0.15	0.00	P,M	164.33	LOC51315	2p11.2
219765_at	1.00	0.97	P	338.80	0.15	0.00	P,A	51.03	FLJ12586	19q13.43
203149_at	1.00	0.96	P	452.87	0.15	0.00	A	67.93	PVRL2	19q13.2-q13.4
210136_at	0.98	0.89	P	346.97	0.15	0.00	P,A	52.27		18q23
210096_at	1.00	0.97	P	101.20	0.15	0.00	A	15.40	CYP4B1	1p34-p12
228640_at	0.99	0.93	P	57.43	0.15	0.02	A	9.27	PCDH7	4p15
229145_at	1.00	0.98	P	764.87	0.15	0.00	P	115.43	C10orf104	10q22.2
226799_at	0.98	0.92	P,A	81.70	0.15	0.01	A	12.70	FGD6	12q23.1
212328_at	1.00	0.95	P	420.10	0.15	0.00	P	63.83	KIAA1102	4p14
220387_s_at	1.00	0.99	P	203.30	0.15	0.00	A	30.83	HHLA3	1p31.2
202291_s_at	0.99	0.94	P	4768.87	0.15	0.00	P	726.63	MGP	12p13.1-p12.3
232244_at	1.00	0.96	P	192.30	0.15	0.01	P,A	30.20	KIAA1161	9p13.2
1554741_s_at	0.98	0.91	P	152.37	0.15	0.00	P	23.43	FLJ30435	9q21.12
201670_s_at	0.99	0.94	P	2029.40	0.15	0.00	P	308.83	MARCKS	6q22.2
225207_at	0.98	0.89	P	55.33	0.15	0.01	A	8.73	PDK4	7q21.3-q22.1
215489_x_at	0.99	0.92	P	1168.83	0.15	0.00	P	178.47	HOMER3	19p13.11
219010_at	0.99	0.94	P	640.10	0.15	0.00	A	99.57	FLJ10901	1q32.1
203717_at	0.97	0.88	P	153.07	0.15	0.01	A	24.00	DPP4	2q24.3
241895_at	0.97	0.87	P,A	17.67	0.15	0.01	A	2.83		2q21.2
238702_at	1.00	0.95	P	76.27	0.15	0.00	P,A	11.80	ADMP	3q26.1
219280_at	0.99	0.94	P	153.43	0.15	0.02	M,A	24.63	C21orf107	21q22.2
239995_at	1.00	0.97	P	213.17	0.15	0.00	P,M,A	32.70		19q13.13
229240_at	1.00	0.96	P	180.77	0.15	0.01	P	28.37	ZDHHC21	9p22.3
1559072_a_at	1.00	0.97	P	228.23	0.16	0.01	A	36.00	KIAA1904	22q13.1
231836_at	0.99	0.93	P	277.97	0.16	0.01	P	44.63	HKR1	19q13.13
215146_s_at	0.99	0.93	P	42.23	0.16	0.02	M,A	6.97	KIAA1043	22q12.1
221088_s_at	1.00	0.98	P	90.23	0.16	0.00	A	13.93	PPP1R9A	7q21.3
235736_at	0.99	0.95	P	127.30	0.16	0.01	P,A	19.90		
228726_at	0.99	0.92	P	497.07	0.16	0.00	P,M	76.57	SERPINB1	6p25
205246_at	0.99	0.92	P	304.00	0.16	0.00	P,A	47.17	PEX13	2p14-p16
241247_at	1.00	0.95	P	106.03	0.16	0.00	A	16.40		3q25.1
242907_at	0.99	0.95	P	176.07	0.16	0.00	P	27.53	GBP2	1p22.2
238315_s_at	0.98	0.91	P	65.50	0.16	0.02	A	11.07	ZNF567	19q13.13
238952_x_at	1.00	0.96	P	88.23	0.16	0.01	P,A	14.10	DKFZp779O175	19q13.13
1562484_at	1.00	0.97	P	45.37	0.16	0.02	A	7.47	FLJ35848	17q21.31
221016_s_at	1.00	0.99	P	318.50	0.16	0.00	M,A	50.40	TCF7L1	2p11.2
232151_at	0.97	0.88	P	107.40	0.16	0.00	A	17.00		
208228_s_at	0.99	0.93	P	129.47	0.16	0.01	A	20.70	FGFR2	10q26
242245_at	1.00	0.99	P	299.70	0.16	0.00	P	46.90		
219976_at	0.99	0.93	P	53.50	0.16	0.01	A	8.50	HOOK1	1p32.1
215506_s_at	0.98	0.89	P	103.20	0.16	0.02	P,A	17.27	ARHI	1p31
229310_at	1.00	0.99	P	150.83	0.16	0.01	A	24.57	KBTBD9	2p24.1
210239_at	0.97	0.88	P	594.43	0.16	0.00	P	94.20	IRX5	16q11.2-q13
1559020_a_at	0.99	0.94	P	23.60	0.16	0.02	A	4.00		
214240_at	0.99	0.93	P	145.17	0.16	0.01	A	23.60	GAL	11q13.1
221824_s_at	1.00	0.96	P	178.27	0.16	0.00	A	28.17	MIR	10q11.22
224989_at	0.99	0.93	P	394.07	0.16	0.01	P	62.93		
214958_s_at	0.97	0.89	P	361.83	0.16	0.00	A	57.93	EVER1	17q25.3
204928_s_at	1.00	0.95	P	970.70	0.16	0.00	P	154.67	SLC10A3	xq28
210220_at	0.98	0.90	P	200.10	0.16	0.01	A	32.40	FZD2	17q21.1
226550_at	0.99	0.93	P	279.10	0.16	0.00	P	44.30		
221841_s_at	0.99	0.94	P	255.30	0.16	0.00	P,A	41.50	KLF4	9q31
224990_at	1.00	0.95	P	876.93	0.16	0.00	P	140.67	LOC201895	4p14
227077_at	1.00	0.99	P	523.27	0.16	0.00	P,A	83.50	ZNF286	17p11.2
226297_at	1.00	0.97	P	2889.33	0.16	0.00	P	461.80	HIPK3	11p13
238937_at	0.98	0.89	P	107.17	0.16	0.02	P,A	18.20	FLJ32191	19q13.13
241355_at	0.99	0.93	P	640.63	0.16	0.00	A	102.50	HR	8p21.2
201556_s_at	0.98	0.90	P	291.13	0.16	0.00	P	46.47	VAMP2	17p13.1
225224_at	0.99	0.93	P	209.40	0.16	0.00	A	34.43	C20orf112	20q11.1-q11.23
204589_at	0.99	0.92	P	165.70	0.16	0.00	P,A	26.77	ARK5	12q24.11
235275_at	1.00	0.96	P	113.30	0.16	0.01	A	19.03	BMP8B	1p35-p32
227944_at	1.00	0.97	P	100.53	0.16	0.01	M,A	16.63		
217967_s_at	1.00	0.98	P	185.47	0.16	0.01	P,A	30.83	C1orf24	1q25
233273_at	0.98	0.91	P	66.60	0.16	0.02	P,A	11.70		
201693_s_at	0.99	0.92	P	390.13	0.16	0.00	A	63.60	EGR1	5q31.1
205020_s_at	1.00	0.99	P	554.50	0.16	0.00	P,A	89.57	ARL4A	7p21-p15.3
1560556_a_at	1.00	0.97	P	79.83	0.16	0.00	P,A	13.10		
1568854_at	1.00	0.96	P	68.40	0.16	0.00	A	11.30		
208789_at	1.00	0.98	P,M	842.63	0.16	0.00	A	136.63	PTRF	17q21.31
214927_at	0.98	0.91	P	210.93	0.16	0.00	A	34.50	ITGBL1	13q33
218849_s_at	1.00	0.98	P	724.57	0.16	0.00	A	117.50	RAI	19q13.32
218693_at	0.97	0.89	P	241.00	0.17	0.01	P	40.47	NET-7	10q22.1
201906_s_at	1.00	0.98	P	940.17	0.17	0.00	P	153.00	CTDSPL	3p21.3
232977_x_at	0.98	0.90	P,M	328.27	0.17	0.01	A	55.63	MYH14	19q13.33
1555310_a_at	1.00	0.96	P	336.20	0.17	0.00	A	55.20	PAK6	15q14
204284_at	1.00	0.97	P	264.07	0.17	0.00	A	43.60	PPP1R3C	10q23-q24
208937_s_at	1.00	0.96	P	769.27	0.17	0.00	P	127.27	ID1	20q11
209502_s_at	0.98	0.90	P	176.43	0.17	0.02	A	30.67	BAIAP2	17q25
212770_at	0.99	0.95	P,M	423.77	0.17	0.00	A	69.90	TLE3	15q22
232567_at	0.99	0.93	P,M	156.37	0.17	0.01	A	27.07	ARHGAP8	22q13.31

216488_s_at	0.92	0.81	P,A	94.47	0.17	0.01	A	16.20	ATP11A	13q34
205793_x_at	0.94	0.83	P,A	242.40	0.17	0.02	A	42.03	TNK1	17p13.1
208712_at	1.00	0.98	P	1011.47	0.17	0.00	P	169.50	CCND1	11q13
222065_s_at	0.97	0.87	P	860.40	0.17	0.01	P	144.63	FLII	17p11.2
228619_x_at	1.00	0.95	P	702.40	0.17	0.00	P	116.33	MGC3794	1q23.2
226736_at	1.00	0.98	P	701.33	0.17	0.01	A	117.93	C14orf52	14q23.3
227284_at	1.00	0.98	P	379.03	0.17	0.00	P	62.93	LOC90321	19q13.41
209191_at	1.00	0.95	P	1638.97	0.17	0.00	P	273.73	MGC4083	18p11.21
212056_at	1.00	0.96	P	505.57	0.17	0.00	A	84.17	KIAA0182	16q24.1
244640_at	1.00	0.98	P	215.77	0.17	0.01	P,A	36.47		
218175_at	0.99	0.94	P,A	545.10	0.17	0.00	A	91.03	FLJ22471	12q24.31
213340_s_at	1.00	0.98	P	186.17	0.17	0.01	P,M,A	31.77	KIAA0495	1p36.32
232060_at	1.00	0.99	P	187.33	0.17	0.00	P	31.37		
201694_s_at	0.99	0.93	P	1126.90	0.17	0.00	P	187.67	EGR1	5q31.1
206170_at	1.00	0.97	P	267.47	0.17	0.01	A	46.13	ADRB2	5q31-q32
204717_s_at	0.99	0.93	P	274.10	0.17	0.01	A	46.90	SLC29A2	11q13
228772_at	1.00	0.98	P	80.93	0.17	0.01	P,A	14.20	HNMT	2q22.1
222354_at	1.00	0.97	P	81.63	0.17	0.00	A	13.80	F11R	1q21.2-q21.3
216594_x_at	0.99	0.95	P	415.97	0.17	0.00	P	71.20	AKR1C1	10p15-p14
1560296_at	0.97	0.88	P	452.73	0.17	0.00	P	76.00		
241356_at	0.97	0.88	P	89.07	0.17	0.01	A	15.57		
201601_x_at	1.00	0.98	P	293.40	0.17	0.00	A	49.80	IFITM1	11p15.5
223434_at	1.00	0.99	P	315.20	0.17	0.00	P	53.33	GBP3	1p22.2
214319_at	0.99	0.93	P	83.33	0.17	0.02	A	15.43	13CDNA73	13q12.3
1568658_at	1.00	0.99	P	406.47	0.17	0.00	P	69.13		2p16.1
223103_at	1.00	0.97	P	1660.93	0.17	0.00	P	281.40	STARD10	11q13
1559026_at	1.00	0.95	P	46.43	0.17	0.01	A	8.17		
1566901_at	1.00	0.98	P	371.37	0.17	0.00	P	63.33		
218284_at	0.99	0.94	P	290.87	0.17	0.01	A	51.10	DKFZP586N0721	15q22.31
223581_at	1.00	0.97	P,A	71.37	0.17	0.00	A	12.17	ZNF577	19q13.41
204663_at	1.00	0.95	P	213.17	0.17	0.00	A	36.73	ME3	11cen-q22.3
211725_s_at	1.00	0.97	P	1016.10	0.17	0.00	P	173.80	BID	22q11.1
227045_at	1.00	0.96	P	294.03	0.17	0.00	P,A	50.53	ZNF614	19q13.41
225273_at	1.00	0.95	P,A	229.40	0.17	0.02	A	41.23	KIAA1280	xp22.32
1566558_x_at	0.98	0.91	P	104.03	0.17	0.01	A	18.37		
220407_s_at	0.96	0.86	P	335.57	0.18	0.00	P,A	58.50	TGFB2	1q41
212057_at	0.99	0.95	P	1255.63	0.18	0.00	P	217.67	KIAA0182	16q24.1
225667_s_at	1.00	0.98	P	152.73	0.18	0.01	A	27.07	NSE1	2p25.1
203232_s_at	1.00	0.97	P	1039.17	0.18	0.01	P	183.10	SCA1	6p23
208156_x_at	0.98	0.91	P	285.07	0.18	0.01	A	51.60		
59697_at	1.00	0.95	P	478.30	0.18	0.00	M,A	83.37	RAB15	14q23.3
203359_s_at	1.00	0.99	P	1370.43	0.18	0.00	P	237.87	MYCBP	1p33-p32.2
222859_s_at	0.98	0.91	P	119.23	0.18	0.02	A	22.70	DAPP1	4q25-q27
1553611_s_at	1.00	0.96	P	80.47	0.18	0.00	A	14.03	FLJ33790	11q13.3
201826_s_at	0.97	0.88	P,M	195.57	0.18	0.00	A	33.90	CGI-49	1q44
202729_s_at	1.00	0.98	P	276.70	0.18	0.00	A	48.17	LTBP1	2p22-p21
228156_at	0.99	0.93	P	305.67	0.18	0.00	P,A	53.13		
227703_s_at	1.00	0.98	P	199.30	0.18	0.00	A	34.70	SYTL4	xq21.33
227603_at	0.99	0.94	P	336.10	0.18	0.00	P	58.73		
222904_s_at	0.99	0.93	P	100.50	0.18	0.00	P,A	17.97	TMC5	16p13.11
205330_at	0.99	0.93	P	105.97	0.18	0.02	A	19.87	MN1	22q12.1
227509_x_at	1.00	0.96	P	155.70	0.18	0.01	P,M	27.50		
238752_at	0.99	0.95	P,A	59.00	0.18	0.00	A	10.33	MRS2L	6p22.3-p22.1
204106_at	1.00	0.99	P	702.57	0.18	0.00	P	123.13	TESK1	9p13
202743_at	1.00	0.96	P	879.13	0.18	0.00	P	154.30	PIK3R3	1p34.1
1554112_a_at	1.00	0.96	P	78.97	0.18	0.00	A	14.00	ULK2	17p11.2
201464_x_at	0.97	0.87	P	623.37	0.18	0.00	A	109.13	JUN	1p32-p31
213052_at	1.00	0.98	P	700.87	0.18	0.00	A	123.83	PRKAR2A	3p21.3-p21.2
211056_s_at	0.99	0.93	P	616.27	0.18	0.00	P	109.53	SRD5A1	5p15
219461_at	1.00	0.96	P	182.73	0.18	0.01	A	34.07	PAK6	15q14
210538_s_at	1.00	0.98	P	92.83	0.18	0.00	P,M	16.47	BIRC3	11q22
203829_at	1.00	0.98	P	474.43	0.18	0.00	M,A	84.73	ELP4	11p13
213478_at	0.99	0.95	P,A	152.70	0.18	0.01	A	27.50	KIAA1026	1p36.13
211799_x_at	0.99	0.94	P	649.87	0.18	0.01	P	117.60	HLA-C	6p21.3
1569076_a_at	0.99	0.95	P	111.47	0.18	0.00	A	19.77		19q13.41
208790_s_at	0.99	0.92	P	118.10	0.18	0.02	P,A	22.20	PTRF	17q21.31
219518_s_at	0.99	0.94	P	185.47	0.18	0.00	P	33.40	ELL3	15q15.1
218145_at	1.00	0.97	P	2859.30	0.18	0.00	P	511.60	TRIB3	20p13-p12.2
224435_at	1.00	0.98	P	1300.87	0.18	0.00	P	232.20	C10orf58	10q23.1
222881_at	0.99	0.94	P	87.60	0.18	0.00	A	15.80	HPSE	4q21.3
1557667_at	1.00	0.97	P	112.87	0.18	0.00	P	20.27		
219622_at	0.98	0.90	P	520.77	0.18	0.00	A	93.00	RAB20	13q34
225263_at	1.00	0.97	P	606.00	0.18	0.00	P	108.40	HS6ST1	2q21
224792_at	1.00	0.97	P	392.10	0.18	0.00	P	70.77	TNKS1BP1	11q12.1
212559_at	0.99	0.93	P	227.73	0.18	0.00	P	40.83	PRKAR1B	7pter-p22
201594_s_at	1.00	0.98	P	2733.47	0.18	0.00	P	490.90	PPP4R1	18p11.22
218019_s_at	1.00	0.98	P	1853.10	0.18	0.00	P	335.90	C21orf97	21q22.3
209227_at	0.97	0.89	P	86.13	0.18	0.02	A	16.80	TUSC3	8p22
205420_at	1.00	0.97	P	350.03	0.18	0.00	P	63.57	PEX7	6q21-q22.2
210987_x_at	0.99	0.92	P	4123.40	0.18	0.00	P	745.90	TPM1	15q22.1
223931_s_at	0.98	0.91	P	144.13	0.18	0.00	A	26.17	CHFR	12q24.33
202037_s_at	0.99	0.95	P	309.63	0.18	0.00	A	56.53	SFRP1	8p12-p11.1
225020_at	0.99	0.95	P	402.60	0.18	0.01	A	74.13	DAB2IP	9q33.1-q33.3
203556_at	0.98	0.90	P	225.47	0.18	0.01	P,A	41.80	ZHX2	8q24.13
209451_at	1.00	0.98	P	902.70	0.18	0.00	P	164.63	TANK	2q24-q31
200965_s_at	1.00	0.97	P	1143.93	0.18	0.00	P	208.77	ABLIM1	10q25
215860_at	0.99	0.92	P	60.93	0.19	0.00	A	11.17	SYT12	11q13.1
203139_at	0.99	0.94	P	258.07	0.19	0.00	A	47.63	DAPK1	9q34.1
223383_at	1.00	0.96	P	671.70	0.19	0.00	P,A	123.30	ZNRF1	16q22.3
243209_at	1.00	0.96	P,A	38.43	0.19	0.02	A	7.37	KCNQ4	1p34
1559503_a_at	0.94	0.83	P,A	7.40	0.19	0.03	A	1.50		10q11.21
234290_x_at	0.98	0.89	P	469.17	0.19	0.00	A	86.43	MYH14	19q13.33
219946_x_at	0.99	0.92	P	460.63	0.19	0.02	A	88.40	MYH14	19q13.33
233550_s_at	0.94	0.83	P,A	128.73	0.19	0.03	A	26.53	SLC4A11	20p12
221843_s_at	0.99	0.92	P	209.17	0.19	0.00	A	38.50	KIAA1609	16q24.1
203961_at	1.00	0.98	P	156.30	0.19	0.01	P,A	29.70	NEBL	10p12

226124_at	1.00	0.99	P	246.60	0.19	0.00	P	45.40	ZFP90	16q22.1
243835_at	0.98	0.90	P	148.37	0.19	0.01	P,A	27.90	ZDHHC21	9p22.3
1558942_at	1.00	0.96	P	488.47	0.19	0.00	P	89.70	LOC91661	19q13.42
228482_at	0.99	0.93	P,A	133.47	0.19	0.01	A	25.70	FLJ36674	17p11.2
1554922_at	0.98	0.91	P,A	8.77	0.19	0.01	A	1.67	MGC42493	1q42.13
226745_at	0.99	0.92	P	212.73	0.19	0.00	P	39.70	CYP4V2	4q35.1
206572_x_at	0.99	0.94	P	309.57	0.19	0.01	A	58.97	ZNF85	19p13.1-p12
242722_at	0.99	0.92	P,A	347.50	0.19	0.01	A	64.83	LMO7	13q21.33
215066_at	0.98	0.91	P,A	85.87	0.19	0.03	A	17.53	PTPRF	1p34
230063_at	0.99	0.94	P	136.03	0.19	0.01	A	26.00	ZNF264	19q13.4
241431_at	1.00	0.97	P	251.23	0.19	0.00	M,A	46.90		
204867_at	0.99	0.94	P	177.60	0.19	0.00	P,A	33.03	GCHFR	15q15
204029_at	0.99	0.95	P	414.73	0.19	0.00	P	77.70	CELSR2	1p21
202727_s_at	1.00	0.96	P	1958.17	0.19	0.00	P	367.20	IFNGR1	6q23-q24
220419_s_at	1.00	0.96	P	707.23	0.19	0.00	P	132.23	USP25	21q11.2
229256_at	0.99	0.94	P	172.33	0.19	0.00	P,M,A	32.73	PGM2L1	11q13.3
224097_s_at	1.00	0.95	P	322.50	0.19	0.00	P	60.87	F11R	1q21.2-q21.3
217979_at	1.00	0.96	P	3846.50	0.19	0.00	P	722.43	TM4SF13	7p21.2
216550_x_at	1.00	0.96	P	360.43	0.19	0.01	P	69.23	ANKRD12	18p11.22
237439_at	0.98	0.90	P	101.90	0.19	0.00	M,A	19.03	USP43	17p13.1
210761_s_at	0.99	0.94	P,A	191.17	0.19	0.00	A	36.17	GRB7	17q21.2
228549_at	1.00	0.96	P	115.53	0.19	0.01	P,A	22.20	KIAA0792	1q42.13
204446_s_at	1.00	0.97	P	72.23	0.19	0.01	A	14.20	ALOX5	10q11.2
227038_at	1.00	0.96	P	287.03	0.19	0.00	P	54.50	MGC26963	4q25
231697_s_at	0.99	0.92	P	142.13	0.19	0.01	P,A	27.37	VMP1	17q23.2
223468_s_at	0.96	0.87	P	357.87	0.19	0.00	P,M,A	67.73	RGMA	15q26.1
219255_x_at	1.00	0.99	P,M	130.40	0.19	0.02	A	26.07	IL17RB	3p21.1
243327_at	0.99	0.92	P	93.77	0.19	0.02	A	18.93		
230394_at	0.98	0.91	P,M	38.27	0.19	0.02	A	7.53	TCP10L	21q22.11
221732_at	1.00	0.97	P	783.03	0.19	0.01	A	154.17	ENTPD8	17q25.3
227500_at	1.00	0.96	P,M	201.57	0.19	0.00	A	38.67		7p22.2
218245_at	0.99	0.93	P	288.20	0.19	0.02	A	57.93	E2IG4	11q13.3
218966_at	0.99	0.95	P	614.17	0.19	0.00	P	118.10	MYO5C	15q21
213403_at	0.97	0.88	P	47.03	0.19	0.01	A	9.27		
221044_s_at	1.00	0.96	P	199.90	0.19	0.01	M,A	38.53	TRIM34	11p15
204131_s_at	0.99	0.95	P	642.97	0.19	0.00	P	122.77	FOXO3A	6q21
229553_at	0.99	0.92	P	267.73	0.19	0.01	P	52.30	PGM2L1	11q13.3
217966_s_at	0.99	0.94	P	98.43	0.19	0.01	A	19.03	C1orf24	1q25
242579_at	1.00	0.96	P	112.20	0.19	0.01	A	21.83		
231861_at	1.00	0.97	P	126.83	0.19	0.00	P,A	24.47	LRP10	14q11.2
211538_s_at	1.00	0.97	P	342.93	0.19	0.00	P	66.07		
223464_at	1.00	0.95	P	246.63	0.19	0.01	A	48.23	OSBPL5	11p15.4
242123_at	0.98	0.90	P	102.73	0.20	0.00	P	19.67	MPRA	1p35.3
223233_s_at	1.00	0.99	P,A	210.17	0.20	0.00	A	40.90	CGN	1q21
234665_x_at	1.00	0.97	P	438.47	0.20	0.00	P,M	84.60	HHLA3	1p31.2
243666_at	0.99	0.95	P	47.13	0.20	0.01	A	9.37	BRUNOL4	18q12
242868_at	0.99	0.93	P,M	64.07	0.20	0.00	A	12.33		
204058_at	0.99	0.92	P	681.00	0.20	0.00	P	131.77	ME1	6q12
221750_at	1.00	0.96	P	1073.07	0.20	0.01	P	208.73	HMGCS1	5p14-p13
203564_at	1.00	0.98	P	1863.00	0.20	0.00	P	359.77	FANCG	9p13
209598_at	0.99	0.94	P	215.80	0.20	0.02	A	44.07	PNMA2	8p21.1
228696_at	1.00	0.96	P	563.80	0.20	0.01	P	113.30	Prostein	1q32.1
220144_s_at	1.00	0.99	P	94.60	0.20	0.00	A	18.43	ANKRD5	20pter-q11.23
206032_at	0.99	0.94	P	119.37	0.20	0.02	P,A	25.07	DSC3	18q12.1
226811_at	1.00	0.95	P	75.40	0.20	0.00	A	14.87	FLJ20202	1p12
242539_at	1.00	0.98	P	120.87	0.20	0.00	P	23.60		
226245_at	1.00	1.00	P	1125.37	0.20	0.00	P	221.30	KCTD1	18q12.1
232051_at	1.00	0.96	P,A	108.33	0.20	0.00	A	21.33	MGC10992	16q13
205427_at	0.98	0.91	P	339.27	0.20	0.00	M,A	66.60	ZNF354A	5q35.3
226807_at	1.00	0.97	P	328.70	0.20	0.00	P,A	64.60	ZFP1	16q22.3
201473_at	0.99	0.94	P	785.77	0.20	0.00	P	156.07	JUNB	19p13.2
202023_at	0.99	0.94	P	292.83	0.20	0.00	A	57.47	EFNA1	1q21-q22
233198_at	0.99	0.92	P	235.57	0.20	0.01	P,A	47.03	LOC92497	12q23.3
210495_x_at	1.00	0.98	P	154.73	0.20	0.00	A	30.63	FN1	2q34
230748_at	1.00	0.95	P	60.80	0.20	0.00	P	11.90	SLC16A6	17q24.3
215093_at	1.00	0.96	P	1078.47	0.20	0.01	P	213.33	NSDHL	xq28
38340_at	0.99	0.94	P	653.30	0.20	0.00	A	127.90	HIP1R	12q24
240690_at	1.00	0.97	P	617.23	0.20	0.00	P	121.67		
204773_at	1.00	0.98	P	198.57	0.20	0.01	P,A	40.13	IL11RA	9p13
1555486_a_at	0.99	0.93	P	447.57	0.20	0.00	A	87.93	FLJ14213	11p13
36499_at	1.00	0.97	P	461.87	0.20	0.00	P	91.23	CELSR2	1p21
204604_at	1.00	0.97	P	317.30	0.20	0.00	P	63.40	PFTK1	7q21-q22
228341_at	1.00	0.98	P	185.73	0.20	0.00	P	36.80	FLJ31265	3q22.1
216867_s_at	1.00	0.95	P,A	44.77	0.20	0.01	A	9.03	PDGFA	7p22
237680_at	0.98	0.91	P,M	62.40	0.20	0.01	A	12.83		
209164_s_at	0.99	0.94	P	619.60	0.20	0.00	A	123.00	CYB561	17q11-qter
219201_s_at	0.99	0.92	P	147.70	0.20	0.01	A	30.30	TWSG1	18p11.3
228393_s_at	1.00	0.96	P	227.27	0.20	0.00	P	45.43	ZNF302	19q13.12
203231_s_at	0.99	0.92	P	477.43	0.20	0.00	P,A	95.47	SCA1	6p23
219232_s_at	1.00	0.98	P	167.27	0.20	0.02	P	35.17	EGLN3	14q13.1
218062_x_at	1.00	0.99	P	1175.30	0.20	0.00	P	236.17	CDC42EP4	17q24-q25
229292_at	1.00	0.99	P	251.23	0.20	0.02	A	52.17	EPB41L5	2q14.2
223540_at	0.98	0.91	P	199.53	0.20	0.01	A	40.57	PVRL4	1q22-q23.2
1557302_at	0.99	0.92	P	178.37	0.20	0.01	P,A	36.67		
218129_s_at	0.97	0.88	P	386.77	0.20	0.00	P	78.03	NFYB	12q22-q23
242462_at	0.99	0.92	P	262.83	0.20	0.00	P,A	52.83		
205977_s_at	1.00	0.98	P	317.93	0.20	0.00	A	64.70	EPHA1	7q34
228608_at	0.99	0.94	P	49.97	0.20	0.01	A	10.53	VGCNL1	13q32.3
229515_at	1.00	0.99	P	134.93	0.20	0.00	P	27.17	PAWR	12q21
236543_at	1.00	0.97	P,A	50.37	0.20	0.02	A	10.63		
228937_at	1.00	0.96	P	281.93	0.20	0.00	P	57.17	FLJ38725	13q14.11
220261_s_at	0.97	0.88	P	112.03	0.20	0.01	A	23.17	ZDHHC4	7p22.2
220088_at	1.00	0.96	P,M,A	60.50	0.20	0.01	A	12.37	C5R1	19q13.3-q13.4
1559171_at	0.98	0.89	P,A	31.70	0.21	0.03	A	6.87		
212509_s_at	1.00	0.97	P	504.80	0.21	0.00	P,A	102.43	FLJ46603	17q25.3
204532_x_at	1.00	0.96	P	446.97	0.21	0.02	P,A	95.90	UGT1A6	2q37

1569136_at	1.00	0.98	P	23.77	0.21	0.01	P,A	5.00	MGAT4A	2q12
226885_at	1.00	0.98	P	343.80	0.21	0.00	P	69.63		
238632_at	0.99	0.93	P	71.20	0.21	0.03	A	15.23		
218531_at	1.00	0.98	P	693.97	0.21	0.00	P	141.73	FLJ21749	11q13.1
209500_x_at	1.00	0.96	P	484.40	0.21	0.00	P	98.43	TNFSF13	17p13.1
219959_at	0.99	0.95	P	382.23	0.21	0.00	P	78.43	MOCOS	18q12
227798_at	1.00	0.96	P	650.80	0.21	0.00	P	132.90	MADH1	4q31
213358_at	0.99	0.93	P	831.77	0.21	0.00	P	170.70	KIAA0802	18p11.22
204328_at	0.99	0.94	P	260.80	0.21	0.00	A	53.50	EVER1	17q25.3
219901_at	0.97	0.88	P	150.93	0.21	0.01	P	31.27	FGD6	12q23.1
37512_at	1.00	0.99	P	86.63	0.21	0.02	P	18.23	RODH	12q13
209386_at	1.00	0.99	P	3857.93	0.21	0.00	P	788.17	TM4SF1	3q21-q25
210542_s_at	1.00	0.97	P	165.47	0.21	0.00	A	34.27	SLCO3A1	15q26
209099_x_at	0.99	0.94	P	522.10	0.21	0.00	P	107.00	JAG1	20p12.1-p11.23
210993_s_at	0.99	0.93	P	470.30	0.21	0.00	P	97.50	SMAD1	4q31
236313_at	0.99	0.94	P	141.40	0.21	0.00	P,A	29.33	CDKN2B	9p21
201432_at	1.00	0.96	P	3366.50	0.21	0.00	P	691.33	CAT	11p13
210845_s_at	1.00	0.97	P	479.30	0.21	0.00	P	98.50	PLAUR	19q13
204243_at	1.00	0.98	P	607.17	0.21	0.00	P	124.70	RLF	1p32
227811_at	0.99	0.92	P	118.27	0.21	0.01	A	25.03	FGD3	9q22
210130_s_at	0.99	0.95	P	129.63	0.21	0.02	A	27.73	TM7SF2	11q13
235591_at	0.97	0.87	P	23.90	0.21	0.02	A	5.23	SSTR1	14q13
212590_at	0.99	0.93	P	427.97	0.21	0.00	P	88.67	RRAS2	11p15.2
229666_s_at	1.00	0.97	P	1693.57	0.21	0.00	P	350.07	CSTF3	11p13
239210_at	0.99	0.92	P	28.47	0.21	0.00	A	5.87		
233955_x_at	0.99	0.94	P	1047.70	0.21	0.00	P	216.67	CXXC5	5q31.3
234491_s_at	1.00	0.98	P	925.13	0.21	0.01	P	192.93	SAV1	14q13-q23
205596_s_at	1.00	0.98	P	734.33	0.21	0.00	P	151.77	SMURF2	17q22-q23
229223_at	0.99	0.94	P	183.63	0.21	0.00	A	37.83		
229613_at	1.00	0.98	P	152.13	0.21	0.00	A	31.37		
210767_at	0.99	0.93	P	68.20	0.21	0.00	A	14.13	NF2	22q12.2
204675_at	1.00	0.98	P	1387.50	0.21	0.00	P	286.37	SRD5A1	5p15
230281_at	1.00	0.96	P	430.43	0.21	0.01	P	89.30	FLJ32702	16q23.2
213540_at	1.00	0.97	P	262.30	0.21	0.00	A	54.77	HSD17B8	6p21.3
238944_at	1.00	0.98	P	312.40	0.21	0.02	A	68.00		
1557129_a_at	1.00	0.99	P	394.43	0.21	0.02	P	84.43	CANP	11q12.2
224516_s_at	1.00	0.99	P	930.53	0.21	0.00	P	194.00	CXXC5	5q31.3
225981_at	0.99	0.93	P	244.77	0.21	0.00	M,A	51.37	C17orf28	17q25.2
216336_x_at	0.98	0.90	P	1377.57	0.21	0.00	P	287.73	MT1E	16q13
214088_s_at	1.00	0.99	P	154.43	0.21	0.00	A	32.13	FUT3	19p13.3
236528_at	0.99	0.92	P	156.57	0.21	0.01	P,A	33.43	UBE2J1	6q16.1
217523_at	0.98	0.91	P	989.60	0.21	0.00	P	207.77	CD44	11p13
227150_at	0.99	0.94	P,M	401.77	0.21	0.00	A	84.03		
214866_at	1.00	1.00	P	172.43	0.21	0.00	A	36.00	PLAUR	19q13
233748_x_at	0.98	0.91	P,A	181.30	0.21	0.00	A	38.23	PRKAG2	7q35-q36
229578_at	1.00	0.98	P	357.60	0.21	0.00	A	75.10	JPH2	20q13.12
240655_at	0.99	0.93	P,A	56.90	0.21	0.01	A	12.10		
226455_at	0.99	0.95	P	785.93	0.21	0.00	P	163.87	CREB3L4	1q22
207675_x_at	0.99	0.94	P,M	281.20	0.21	0.00	A	59.23	ARTN	1p33-p32
224518_s_at	0.99	0.94	P,A	87.57	0.21	0.01	A	18.70	ZNF559	19p13.2
219077_s_at	1.00	0.96	P	353.13	0.21	0.00	M,A	74.23	WWOX	16q23.3-q24.1
203919_at	0.94	0.83	P,M	44.87	0.21	0.03	A	9.83	TCEA2	20q13.33
237064_x_at	0.99	0.93	P	162.07	0.21	0.01	P	34.77		
230487_at	0.98	0.90	P,A	51.80	0.21	0.01	A	11.27		6q25.3
230518_at	1.00	0.96	P	331.30	0.22	0.00	P	70.13	EVA1	11q24
244467_at	0.99	0.94	P	111.43	0.22	0.00	A	23.70		
227280_s_at	1.00	0.98	P	732.03	0.22	0.00	P	156.07	FLJ40432	2q34
227143_s_at	1.00	0.97	P	461.63	0.22	0.01	P	102.00	BID	22q11.1
232124_at	0.99	0.92	P,A	14.57	0.22	0.00	A	3.10	DKFZP434B172	3p21.33
238030_at	1.00	0.97	P	168.57	0.22	0.00	P	35.83		
241704_x_at	1.00	0.96	P	349.73	0.22	0.01	P	76.07	LOC162967	19q13.42
224925_at	0.97	0.88	P,A	42.50	0.22	0.02	A	9.30	PREX1	20q13.13
204584_at	1.00	0.97	P	202.13	0.22	0.00	A	43.20	L1CAM	xq28
218432_at	0.99	0.93	P	1186.87	0.22	0.00	P	253.90	FBXO3	11p13
222996_s_at	1.00	0.98	P	637.77	0.22	0.00	P	137.43	CXXC5	5q31.3
230404_at	1.00	0.95	P	59.50	0.22	0.00	A	12.77		5q23.3
216442_x_at	0.99	0.94	P	125.97	0.22	0.01	A	27.53	FN1	2q34
201685_s_at	0.99	0.94	P	115.87	0.22	0.00	A	24.70	C14orf92	14q11.2
205479_s_at	0.99	0.94	P	105.67	0.22	0.01	A	22.90	PLAU	10q24
1552283_s_at	1.00	0.99	P	97.50	0.22	0.01	A	21.07	ZDHHC11	5p15.33
203509_at	1.00	0.98	P	863.50	0.22	0.00	P	185.33	SORL1	11q23.2-q24.2
823_at	0.99	0.92	P	396.63	0.22	0.00	P	84.87	CX3CL1	16q13
213713_s_at	0.99	0.93	P,M	278.37	0.22	0.01	A	61.10	LOC89944	11q25
202844_s_at	0.99	0.94	P	739.70	0.22	0.01	P	160.87	RALBP1	18p11.3
203964_at	1.00	0.98	P	1282.37	0.22	0.00	P	276.07	NMI	2p24.3-q21.3
201855_s_at	0.99	0.94	P	909.67	0.22	0.01	P	197.83	KIAA0431	16q23.2
208456_s_at	0.97	0.88	P	156.33	0.22	0.01	P,A	34.13	RRAS2	11p15.2
202081_at	1.00	0.98	P	4101.93	0.22	0.00	P	884.80	IER2	19p13.13
202322_s_at	1.00	0.96	P	896.17	0.22	0.00	P	193.70	GGPS1	1q43
204447_at	1.00	0.95	P,A	291.67	0.22	0.00	A	62.93	ProSAPiP1	20p13
213804_at	1.00	0.97	P	126.70	0.22	0.02	P,A	28.13	INPP5B	1p34
228865_at	1.00	0.96	P	163.93	0.22	0.01	A	36.77	SARG	1q32.1
205583_s_at	1.00	0.96	P	570.57	0.22	0.01	P	124.73	FLJ23018	xq23
234300_s_at	0.98	0.90	P	69.37	0.22	0.01	M,A	15.37	ZFP28	19q13.43
203484_at	1.00	0.98	P	6663.57	0.22	0.00	P	1450.47	SEC61G	7p11.2
211031_s_at	0.99	0.92	P	120.47	0.22	0.01	A	26.47	CYLN2	7q11.23
57540_at	1.00	0.98	P	132.27	0.22	0.00	P,A	28.77	RBKS	2p23.3
223842_s_at	0.99	0.94	P	72.00	0.22	0.00	A	15.70	SCARA3	8p21
244050_at	0.99	0.95	P	91.57	0.22	0.01	A	20.67	LOC401494	9p21.3
205832_at	1.00	0.95	P	455.53	0.22	0.00	A	100.90	CPA4	7q32
217759_at	1.00	0.98	P	1504.10	0.22	0.00	P	330.23	TRIM44	11p13
210069_at	0.99	0.93	P	193.07	0.22	0.01	P	42.70	CPT1B	22q13.33
219371_s_at	0.99	0.95	P	360.87	0.22	0.01	P,A	79.73	KLF2	19p13.13-p13.11
228461_at	1.00	0.98	P	119.20	0.22	0.01	A	26.60	SH3MD4	2q13
215034_s_at	0.98	0.89	P	1168.97	0.22	0.00	P	258.37	TM4SF1	3q21-q25
206825_at	1.00	0.97	P	164.27	0.22	0.00	A	36.13	OXTR	3p25

210652_s_at	1.00	0.95	P	214.03	0.22	0.00	M,A	47.23	C1orf34	1p32.3
225671_at	0.99	0.94	P,A	386.57	0.22	0.01	A	86.57	LOC124976	17p13.3
228670_at	0.99	0.94	P	302.73	0.22	0.01	P	67.23	TEP1	14q11.2
228065_at	0.99	0.94	P	654.87	0.22	0.00	P	144.57	BCL9L	11q23.3
206557_at	1.00	0.95	P	92.17	0.22	0.01	A	20.57	FLJ12985	19q13.42
241455_at	0.99	0.94	P,M,A	59.70	0.22	0.01	A	13.43		
239492_at	0.99	0.92	P	117.67	0.22	0.01	P,A	26.10	SEC14L4	22q12.2
218490_s_at	1.00	0.99	P	271.30	0.22	0.01	P	60.47	ZNF302	19q13.12
227264_at	0.97	0.88	P	295.13	0.22	0.01	A	65.50	TRAF6	11p13
232771_at	0.98	0.90	P,A	29.80	0.23	0.02	A	6.90	NRK	xq22.3
212024_x_at	0.98	0.89	P,M	693.30	0.23	0.00	P,A	153.63	FLII	17p11.2
243188_at	1.00	0.97	P	137.10	0.23	0.01	P	30.67	ZNF283	19q13.32
213659_at	1.00	0.96	P	117.83	0.23	0.02	P,A	26.77	ZNF75	xq26.3
204254_s_at	1.00	0.97	P	538.73	0.23	0.00	P	119.77	VDR	12q12-q14
201681_s_at	1.00	0.99	P	872.77	0.23	0.00	P	194.53	DLG5	10q23
206958_s_at	1.00	0.96	P	1341.70	0.23	0.00	P	299.43	UPF3A	13q34
239007_at	1.00	0.97	P	73.80	0.23	0.00	P	16.47	ZNF616	19q13.41
239236_at	0.99	0.95	P	161.93	0.23	0.01	P,M	36.67		
221867_at	1.00	0.97	P	131.87	0.23	0.00	A	29.33	FLJ31821	16q12.1
239528_at	0.98	0.89	P	210.37	0.23	0.00	A	47.03	PROM2	2q11.2
219676_at	0.99	0.93	P	236.07	0.23	0.00	P,A	52.77	ZNF435	6p21.33
239768_x_at	0.99	0.92	P	89.27	0.23	0.01	A	20.50		
206698_at	0.99	0.94	P	95.63	0.23	0.01	A	21.87	XK	xp21.1
1558854_a_at	0.98	0.89	P	21.50	0.23	0.03	A	5.00	FLJ40342	17q21.32
204595_s_at	0.96	0.85	P	183.63	0.23	0.01	A	41.87	STC1	8p21-p11.2
1558943_x_at	1.00	0.95	P	466.97	0.23	0.01	P	105.63	LOC91661	19q13.42
203026_at	1.00	0.97	P	998.43	0.23	0.00	P	222.87	ZBTB5	9p13.1
1557145_at	1.00	0.97	P	139.73	0.23	0.02	P	32.20	STK38	6p21
205130_at	1.00	0.99	P	1773.27	0.23	0.00	P	397.43	RAGE	14q32
228999_at	1.00	0.97	P	123.23	0.23	0.00	P	27.60	CHD2	15q26
208729_x_at	0.99	0.92	P	1012.73	0.23	0.00	P	229.20	HLA-B	6p21.3
217165_x_at	1.00	0.98	P	1297.90	0.23	0.02	P,M	299.60	MT2A	16q13
208763_s_at	1.00	0.98	P	842.17	0.23	0.00	P,M	189.23	DSIPI	xq22.3
215033_at	0.98	0.91	P,A	81.20	0.23	0.01	A	18.63	TM4SF1	3q21-q25
235095_at	0.96	0.86	P	106.60	0.23	0.01	A	24.23	LOC146439	16p13.3
219151_s_at	1.00	0.99	P	234.13	0.23	0.00	P	52.70	RABL2B	22q13.33
229665_at	1.00	0.98	P	849.33	0.23	0.01	P	192.53	CSTF3	11p13
230441_at	0.96	0.86	P	73.17	0.23	0.01	P,A	16.87	KIAA1909	5p15.33
1558105_a_at	0.99	0.92	P	147.27	0.23	0.00	P	33.30		
214820_at	0.99	0.95	P	189.93	0.23	0.01	P	43.10	C21orf107	21q22.2
210524_x_at	0.98	0.90	P	973.93	0.23	0.00	P	219.13	MT1F	16q13
205190_at	0.99	0.95	P	750.53	0.23	0.00	P	169.63	PLS1	3q23
230088_at	0.98	0.91	P,A	44.97	0.23	0.01	A	10.23		
219101_x_at	0.99	0.95	P,M	160.77	0.23	0.00	A	36.47	ABHD8	19p13.12
227613_at	0.98	0.89	P	92.23	0.23	0.00	A	20.97		
228904_at	1.00	0.97	P	420.03	0.23	0.00	P	94.90		
228126_x_at	0.99	0.92	P,A	93.90	0.23	0.00	A	21.60	CTXN1	19
204657_s_at	1.00	0.98	P	534.90	0.23	0.00	A	121.03	SHB	9p12-p11
225010_at	0.99	0.95	P	2110.83	0.23	0.00	P	479.93	CCDC6	10q21
205756_s_at	1.00	0.99	P	377.97	0.23	0.00	P	85.60	F8	xq28
1569208_a_at	0.98	0.89	P	32.87	0.23	0.03	A	7.87		
243594_x_at	0.99	0.93	P	186.70	0.23	0.00	A	42.30	SPIRE2	16q24
1555259_at	0.98	0.89	P	245.73	0.23	0.00	P,M	56.27	ZAK	2q24.2
209367_at	1.00	0.97	P	381.23	0.23	0.01	P,M	87.67	STXBP2	19p13.3-p13.2
205027_s_at	0.98	0.90	P	32.33	0.23	0.01	A	7.50	MAP3K8	10p12.1
213629_x_at	0.98	0.89	P	1080.43	0.23	0.01	P	250.07	MT1F	16q13
1557222_at	0.98	0.91	P	41.50	0.23	0.00	A	9.57		
232028_at	1.00	0.98	P	297.07	0.23	0.00	P	67.80		
226982_at	1.00	0.98	P	310.90	0.23	0.00	P	70.80	ELL2	5q15
1569723_a_at	1.00	0.98	P,A	265.67	0.23	0.01	M,A	61.37	SPIRE2	16q24
226028_at	1.00	0.95	P,A	23.30	0.23	0.01	A	5.47	ROBO4	11q24.2
209566_at	0.98	0.91	P	292.47	0.23	0.01	P	68.33	INSIG2	2q14.2
1555842_at	0.99	0.93	P	237.10	0.23	0.02	P,M,A	56.17	LOC284356	19q13.33
218127_at	0.99	0.93	P	605.77	0.23	0.00	P	140.20	NFYB	12q22-q23
242414_at	1.00	0.97	P	659.47	0.23	0.00	A	153.93	QPRT	16p12.1
209633_at	1.00	0.99	P	451.23	0.23	0.01	P	104.80	PPP2R3A	3q22.1
223592_s_at	1.00	0.96	P	425.20	0.23	0.00	P	98.10	RNF135	17q11.2
1558487_a_at	1.00	0.97	P	1171.30	0.23	0.00	P	270.83	HNLF	7p13
204148_s_at	0.99	0.95	P	342.23	0.24	0.00	A	78.93	POMZP3	7q11.23
1555894_s_at	0.99	0.92	P	379.20	0.24	0.00	A	87.90	LOC92154	16q22.1
235498_at	1.00	0.96	P	75.67	0.24	0.00	P,A	17.70	MGC22773	1p31.1
212256_at	0.98	0.90	P	749.83	0.24	0.00	P	176.73	GALNT10	5q33.2
225806_at	1.00	0.97	P	478.70	0.24	0.00	P	111.10	JUB	14q11.2
1553764_a_at	0.97	0.88	P	309.67	0.24	0.00	P	72.23	JUB	14q11.2
218292_s_at	0.99	0.94	P,A	133.80	0.24	0.02	A	32.47	PRKAG2	7q35-q36
219496_at	1.00	0.96	P	358.70	0.24	0.00	P	83.30	C2orf26	2q13
238787_at	1.00	0.95	P	193.57	0.24	0.01	P	45.53		
228567_at	1.00	0.97	P	399.50	0.24	0.00	P	93.30		
218856_at	1.00	0.98	P	1018.20	0.24	0.00	M,A	237.13	TNFRSF21	6p21.1-12.2
222462_s_at	0.99	0.92	P	284.87	0.24	0.02	P,A	69.37	BACE1	11q23.2-q23.3
219032_x_at	1.00	0.98	P	277.33	0.24	0.00	P	64.53	OPN3	1q43
204062_s_at	1.00	0.98	P	103.27	0.24	0.02	A	25.03	ULK2	17p11.2
1556009_at	1.00	0.96	P	118.60	0.24	0.00	P	27.97	PEX13	2p14-p16
234963_s_at	0.94	0.84	P,A	113.13	0.24	0.01	A	27.50	FA2H	16q23
219228_at	0.99	0.94	P	220.17	0.24	0.01	P,M	52.27	ZNF331	19q13.3-q13.4
206747_at	0.99	0.94	P	254.73	0.24	0.00	A	59.50	KIAA0514	10q11.22
213959_s_at	0.99	0.94	P	162.30	0.24	0.01	P	38.63	KIAA1005	16q12.2
201669_s_at	1.00	0.96	P	4007.30	0.24	0.00	P	939.50	MARCKS	6q22.2
212443_at	1.00	0.96	P,A	148.67	0.24	0.02	A	36.40	KIAA0540	3p21.31
213002_at	1.00	0.95	P	496.27	0.24	0.00	P	116.93	MARCKS	6q22.2
224665_at	0.99	0.92	P	1062.97	0.24	0.00	P	249.93	C10orf104	10q22.2
219270_at	0.98	0.89	P,A	188.20	0.24	0.00	A	43.90	MGC4504	15q14
204842_x_at	0.98	0.91	P	708.93	0.24	0.02	P,A	170.50	PRKAR2A	3p21.3-p21.2
204702_s_at	1.00	0.95	P	312.13	0.24	0.01	P	74.03	NFE2L3	7p15-p14
211911_x_at	1.00	0.95	P	1825.97	0.24	0.00	P	430.87	HLA-C	6p21.3
218854_at	0.99	0.92	P	96.20	0.24	0.00	M,A	22.77	SART2	6q22

200884_at	1.00	0.99	P	313.20	0.24	0.00	P,M,A	74.13	CKB	14q32
50221_at	0.96	0.85	P	177.23	0.24	0.00	A	41.83	TFEB	6p21
205225_at	1.00	0.95	P,M	179.43	0.24	0.00	A	42.67	ESR1	6q25.1
1556425_a_at	1.00	0.98	P	252.40	0.24	0.00	P	59.63	LOC284219	18p11.22
235739_at	1.00	0.96	P	160.40	0.24	0.00	A	38.03		
226584_s_at	1.00	0.99	P	303.83	0.24	0.00	P	72.27	C20orf55	20p13
205992_s_at	1.00	0.97	P	56.43	0.24	0.02	M,A	13.73	IL15	4q31
212774_at	1.00	0.99	P	976.60	0.24	0.00	P	231.03	ZNF238	1q44-qter
232281_at	1.00	0.96	P	134.80	0.24	0.00	P,A	32.03		
209684_at	1.00	0.96	P	313.77	0.24	0.00	P	74.67	RIN2	20p11.22
204059_s_at	1.00	0.96	P	1260.77	0.24	0.00	P	298.87	ME1	6q12
213568_at	0.99	0.94	P	179.47	0.24	0.00	P	42.63	OSR2	8q22.2
1552634_a_at	0.99	0.93	P	174.83	0.24	0.02	P,A	42.23	ZNF101	19p13.11
209140_x_at	1.00	1.00	P	2277.20	0.24	0.00	P	539.70	HLA-C	6p21.3
223179_at	1.00	0.95	P	312.20	0.24	0.00	A	74.33	MGC10500	16p12.1
241002_at	1.00	0.96	P	46.60	0.24	0.02	A	11.57		
212775_at	1.00	0.98	P	746.23	0.24	0.00	P	177.30	KIAA0657	2q36.1
223094_s_at	0.96	0.86	P,A	122.07	0.24	0.00	A	29.43	ANKH	5p15.1
224664_at	1.00	0.98	P	1932.20	0.24	0.00	P	461.40	C10orf104	10q22.2
221904_at	0.99	0.95	P	100.67	0.24	0.01	P,A	24.73	MGC21688	3q27.3
1555874_x_at	0.99	0.92	P	228.13	0.24	0.01	A	54.70	MGC21881	9q21.11
236073_at	0.99	0.93	P,A	56.57	0.24	0.01	A	13.90		
230406_at	0.99	0.93	P,M,A	114.40	0.24	0.01	A	27.70		
224496_s_at	1.00	0.97	P	125.77	0.24	0.01	P,A	30.60	MGC10744	17p13.1
225746_at	0.98	0.91	P	46.77	0.24	0.02	A	11.63	RAB11-FIP4	17q11.2
225509_at	1.00	0.97	P	493.40	0.24	0.00	P	118.20	LOC56757	5q31-q32
235093_at	1.00	0.97	P	587.83	0.24	0.00	P	140.80		
238853_at	1.00	0.95	P,A	186.47	0.24	0.02	A	46.27		
224838_at	0.99	0.95	P	181.13	0.24	0.00	A	43.43	FOXP1	3p14.1
34408_at	0.99	0.94	P	223.80	0.24	0.00	P	53.60	RTN2	19q13.32
213351_s_at	0.99	0.94	P,A	107.93	0.24	0.03	A	26.97	KIAA0779	3q21.3
244579_at	0.99	0.94	P,A	124.97	0.24	0.02	A	31.20		
212325_at	0.97	0.89	P	291.20	0.24	0.01	P	70.73	KIAA1102	4p14
203773_x_at	0.99	0.92	P	1224.63	0.24	0.00	P	293.63	BLVRA	7p14-cen
37996_s_at	1.00	1.00	P	285.07	0.24	0.00	P	68.60	DMPK	19q13.3
229160_at	1.00	0.96	P	50.47	0.24	0.00	A	12.17	FLJ33516	xq22.3
221696_s_at	1.00	0.99	P	85.30	0.24	0.00	P	20.80	STYK1	12p13.31
240176_at	1.00	0.96	P	99.13	0.25	0.02	A	24.47		
235142_at	0.99	0.92	P	484.67	0.25	0.00	P	117.53	ZBTB8	1p34.3
229025_s_at	1.00	0.99	P	1516.57	0.25	0.00	P	366.17	FLJ25059	11p14.1
225432_s_at	1.00	0.95	P	270.03	0.25	0.00	P,M,A	65.57	CSRP2BP	20p11.23
227450_at	1.00	0.98	P	359.37	0.25	0.00	P	88.07	FLJ32115	12p13.1
224617_at	1.00	0.98	P	2163.37	0.25	0.01	P	526.47	ROD1	9q33.1
235685_at	1.00	0.97	P	174.43	0.25	0.00	P	42.23		
213668_s_at	0.99	0.92	P	236.23	0.25	0.02	P,A	58.83	SOX4	6p22.3
231986_at	1.00	0.95	P	61.70	0.25	0.00	P,A	14.97	RIMS1	6q12-q13
212662_at	1.00	0.97	P	283.00	0.25	0.01	P,M	69.47	PVR	19q13.2
226809_at	1.00	0.96	P	110.00	0.25	0.01	P,M,A	26.83		
231231_at	0.95	0.85	P,M,A	45.53	0.25	0.01	A	11.13		
201349_at	0.99	0.92	P	1238.20	0.25	0.00	P	304.43	SLC9A3R1	17q25.2
222392_x_at	1.00	0.97	P	3680.07	0.25	0.00	P	895.20	PERP	6q24
206954_at	0.95	0.84	P	154.70	0.25	0.02	A	39.37	WIT-1	11p13
1557236_at	1.00	0.96	P	120.43	0.25	0.01	P	30.17		
226411_at	0.97	0.88	P,M,A	53.63	0.25	0.03	A	14.00	LOC115704	19p13.3
205467_at	1.00	0.96	P,A	98.67	0.25	0.02	A	24.93	CASP10	2q33-q34
209522_s_at	1.00	0.97	P	266.10	0.25	0.00	P	65.07	CRAT	9q34.1
222573_s_at	0.97	0.89	P	536.97	0.25	0.00	P	131.93	SAV1	14q13-q23
227215_at	1.00	0.96	P	275.77	0.25	0.00	P,M,A	68.07	GOPC	6q21
204493_at	0.99	0.93	P	358.47	0.25	0.00	P	88.17	BID	22q11.1
227116_at	1.00	0.99	P	425.00	0.25	0.00	P	104.43	HSRG1	16q23.1
213427_at	1.00	0.97	P	1792.00	0.25	0.00	P	438.83	RPP40	6p25.1
1554179_s_at	0.99	0.92	P,A	495.60	0.25	0.01	A	124.50	LYNX1	8q24.3
229318_at	1.00	0.97	P	162.10	0.25	0.00	P,M	40.00		
219718_at	0.99	0.92	P	141.70	0.25	0.01	A	34.90	FLJ10986	1p32.1
205584_at	0.99	0.95	P	300.70	0.25	0.01	P	74.70	FLJ23018	xq23
225022_at	1.00	0.97	P	1040.87	0.25	0.00	P	255.80	GOPC	6q21
238028_at	0.99	0.94	P	88.87	0.25	0.00	A	21.83		6p21.1
203906_at	0.99	0.91	P	147.57	0.25	0.01	A	36.83	KIAA0763	3p25.2
211926_s_at	0.97	0.89	P	1075.73	0.25	0.00	P	267.50	MYH9	22q13.1
1562307_at	0.97	0.88	P	27.13	0.25	0.00	A	6.67		
222858_s_at	1.00	0.97	P	123.53	0.25	0.02	A	31.70	DAPP1	4q25-q27
237435_at	0.99	0.94	P	22.30	0.25	0.01	P,A	5.57		
241459_at	1.00	0.98	P,A	51.10	0.25	0.00	A	12.60		
208436_s_at	1.00	0.96	P	259.17	0.25	0.00	P,M	64.30	IRF7	11p15.5
240081_at	1.00	0.97	P	37.17	0.25	0.00	A	9.23		
229221_at	1.00	0.99	P	686.53	0.25	0.00	P	169.97	CD44	11p13
210910_s_at	1.00	1.00	P	336.33	0.25	0.00	P	83.40	POMZP3	7q11.23
219290_x_at	0.97	0.87	P	272.13	0.25	0.01	P	67.43	DAPP1	4q25-q27
228032_s_at	1.00	0.98	P	312.03	0.25	0.00	P	77.27		
217783_s_at	1.00	0.99	P	707.40	0.25	0.00	P	175.37	CGI-127	2p23.3
228242_at	1.00	0.98	P	110.10	0.25	0.02	P,A	27.90		
230679_at	1.00	0.97	P	180.57	0.25	0.01	P	45.07		
224929_at	1.00	0.97	P	231.40	0.25	0.00	A	57.43	LOC340061	5q31.3
227670_at	1.00	0.97	P	223.40	0.25	0.01	P,A	55.73	ZNF75A	16p13.11
225299_at	0.99	0.94	P	84.53	0.25	0.02	A	21.70	MYO5B	18q21
224891_at	1.00	0.99	P	1530.63	0.25	0.00	P	379.67	FOXO3A	6q21
219298_at	1.00	0.98	P,M	115.40	0.25	0.00	A	28.93	ECHDC3	10p14
232150_at	0.99	0.93	P	212.40	0.25	0.01	P,A	53.30		
220432_s_at	1.00	0.96	P	129.87	0.25	0.01	P	32.70	CYP39A1	6p21.1-p11.2
219410_at	1.00	0.96	P	1559.60	0.25	0.00	P	389.57	FLJ10134	3q12.3
205042_at	1.00	0.97	P	1669.93	0.25	0.00	P	419.07	GNE	9p13.1
204197_s_at	0.99	0.94	P	144.47	0.25	0.01	P,A	36.83	RUNX3	1p36
235798_at	0.99	0.92	P	69.13	0.25	0.01	P,M	17.50		
213075_at	0.99	0.93	P,A	63.80	0.25	0.01	A	16.27	OLFML2A	9q34.11
225548_at	0.99	0.95	P	759.50	0.25	0.00	P	191.37	ShrmL	4q21.22
215299_x_at	1.00	0.96	P	577.77	0.25	0.00	P	144.80	SULT1A1	16p12.1

206940_s_at	0.98	0.91	P	135.47	0.26	0.00	P,A	34.07	POU4F1	13q21.1-q22
228400_at	1.00	0.96	P	248.40	0.26	0.01	P	63.53		
238768_at	1.00	0.98	P	464.37	0.26	0.00	P	117.27		2p11.2
202951_at	1.00	0.99	P	1056.30	0.26	0.01	P	266.90	STK38	6p21
209457_at	0.98	0.90	P	537.63	0.26	0.00	P	136.07	DUSP5	10q25
231940_at	0.99	0.95	P	320.07	0.26	0.00	P	80.87	ZNF529	19q13.13
243201_at	1.00	0.99	P	45.23	0.26	0.01	A	11.67	HNRPH2	Xq22
223576_at	1.00	0.97	P	555.23	0.26	0.01	P	141.47	C6orf203	6q21
218603_at	1.00	0.95	P	618.20	0.26	0.00	P	156.30	HECA	6q23-q24
209387_s_at	0.97	0.87	P	1619.00	0.26	0.00	P	412.50	TM4SF1	3q21-q25
208998_at	1.00	1.00	P	3219.63	0.26	0.00	P	814.40	UCP2	11q13
212543_at	1.00	0.97	P	2321.37	0.26	0.00	P	588.57	AIM1	6q21
229030_at	0.97	0.87	P	71.10	0.26	0.01	P,A	18.10		
222217_s_at	1.00	0.99	P	387.13	0.26	0.00	P	98.23	SLC27A3	1q22
225411_at	0.99	0.93	P	460.97	0.26	0.00	P	117.30	FLJ14681	2q13
209279_s_at	1.00	0.96	P	1073.77	0.26	0.00	P	271.77	NSDHL	xq28
1556284_at	0.99	0.93	P	153.93	0.26	0.02	P	40.33	PPA2	4q25
228452_at	0.98	0.90	P	121.63	0.26	0.00	P	30.83	C17orf39	17p11.2
202557_at	1.00	0.95	P	497.43	0.26	0.00	P	126.43	STCH	21q11
203360_s_at	1.00	0.99	P	960.43	0.26	0.00	P	243.47	MYCBP	1p33-p32.2
211676_s_at	0.97	0.89	P	766.30	0.26	0.00	P	195.63	IFNGR1	6q23-q24
218983_at	0.97	0.87	P	156.27	0.26	0.00	P	40.03	C1RL	12p13.31
217665_at	1.00	0.96	P	68.93	0.26	0.02	P	18.13		
235729_at	1.00	0.99	P	137.43	0.26	0.01	P	35.90	ZNF514	2q11.2
214415_at	1.00	0.97	P	27.43	0.26	0.01	A	7.10		
218744_s_at	0.98	0.91	P	357.80	0.26	0.01	P	91.73	PACSIN3	11p12-p11.12
221589_s_at	1.00	0.98	P	199.50	0.26	0.00	P	51.03	ALDH6A1	14q24.3
228857_at	1.00	0.96	P,M	224.10	0.26	0.00	P,M,A	57.17	LOC285831	6p21.32
208370_s_at	1.00	0.98	P	2796.23	0.26	0.00	P	715.87	DSCR1	21q22.12
203268_s_at	0.97	0.88	P	279.47	0.26	0.00	A	71.23	DRG2	17p11.2
215930_s_at	1.00	0.95	P	216.53	0.26	0.01	P	55.60	MGEA6	14q13.3
212989_at	1.00	0.97	P	436.67	0.26	0.00	P	112.43	TMEM23	10q11.2
219704_at	1.00	0.97	P	216.33	0.26	0.02	A	57.83	YBX2	17p11.2-p13.1
217760_at	1.00	0.99	P	976.63	0.26	0.00	P	249.93	TRIM44	11p13
230944_at	1.00	0.98	P,A	95.53	0.26	0.00	A	24.57	MGC45491	6p21.1
224335_s_at	0.99	0.93	P	264.20	0.26	0.00	A	68.37	BACE1	11q23.2-q23.3
201708_s_at	1.00	0.97	P	530.13	0.26	0.01	P,A	139.30	NIPSNAP1	22q12.2
225959_s_at	1.00	0.98	P	498.20	0.26	0.00	P,A	128.73	ZNRF1	16q22.3
226504_at	0.98	0.90	P,M,A	147.30	0.26	0.02	A	39.47	LOC150368	22q13.31
204346_s_at	1.00	0.95	P	362.97	0.26	0.00	P,M,A	93.60	RASSF1	3p21.3
209703_x_at	1.00	0.98	P	97.03	0.26	0.01	P	25.30	DKFZP586A0522	12q13.13
232481_s_at	0.99	0.94	P	105.13	0.26	0.01	A	27.57	SLITRK6	13q31.1
203313_s_at	0.99	0.93	P	1057.83	0.26	0.00	P	274.07	TGIF	18p11.3
212311_at	0.98	0.90	P	113.20	0.26	0.02	M,A	30.80	KIAA0746	4p15.31
226529_at	0.99	0.95	P	731.97	0.26	0.00	P	189.50	FLJ11273	7p21.3
209834_at	1.00	0.97	P,M,A	235.83	0.26	0.01	A	61.87	CHST3	10q22.2
227432_s_at	0.98	0.91	P	248.77	0.26	0.01	P	65.60	INSR	19p13.3-p13.2
206774_at	1.00	0.99	P,A	84.67	0.26	0.01	A	22.27	FRMPD1	9p13.1
229810_at	1.00	0.99	P	211.43	0.26	0.00	P	54.80		
213030_s_at	1.00	0.99	P	178.80	0.26	0.01	P,A	46.70	PLXNA2	1q32.2
227196_at	1.00	0.98	P	974.23	0.26	0.01	P	254.57	RHPN2	19q13.12
225886_at	1.00	0.96	P	351.87	0.26	0.01	P	92.50	DDX5	17q21
226666_at	0.98	0.91	P	327.00	0.26	0.00	P	85.53	DAAM1	14q23.1
212268_at	0.99	0.94	P	726.60	0.26	0.00	P	189.97	SERPINB1	6p25
209282_at	0.99	0.93	P,M,A	252.43	0.26	0.00	A	66.20	PRKD2	19q13.3
227421_at	1.00	0.97	P	634.47	0.27	0.00	P	165.80	C21orf57	21q22.3
225665_at	1.00	0.96	P	2090.23	0.27	0.00	P	547.90	ZAK	2q24.2
228381_at	1.00	0.98	P,M	164.23	0.27	0.00	A	43.43	ATF7IP2	16p13.2
216028_at	1.00	0.95	P	464.30	0.27	0.00	P	121.27	DKFZP564C152	11
229506_at	0.97	0.88	P	70.43	0.27	0.00	A	18.57		
228531_at	1.00	0.97	P	71.43	0.27	0.00	P,M,A	18.77	FLJ20073	7q21.3
1569443_s_at	0.97	0.88	P,A	59.47	0.27	0.00	A	15.50		
218388_at	1.00	0.96	P	1377.03	0.27	0.00	P	361.73	PGLS	19p13.2
228864_at	0.98	0.89	P	130.43	0.27	0.00	P,A	34.57	LOC115950	19p13.2
225264_at	1.00	0.98	P	1461.47	0.27	0.00	P	383.37	RARSL	6q16.1
218780_at	1.00	0.99	P	500.33	0.27	0.00	P,M	131.97	HOOK2	19p13.2
220325_at	0.99	0.95	P,A	84.50	0.27	0.01	A	22.40	TAF7L	xq22.1
220669_at	1.00	0.97	P	66.63	0.27	0.01	P	17.73	HSHIN1	4q31.21
212757_s_at	1.00	0.98	P	606.03	0.27	0.00	P	160.87	CAMK2G	10q22
210355_at	0.99	0.93	P,A	21.53	0.27	0.01	A	5.77	PTHLH	12p12.1-p11.2
201110_s_at	1.00	0.95	P	1784.40	0.27	0.01	P	474.10	THBS1	15q15
214323_s_at	1.00	0.99	P	2496.80	0.27	0.00	P	660.70	UPF3A	13q34
224580_at	1.00	0.98	P	129.87	0.27	0.01	P,A	34.70	SLC38A1	12q13.11
208812_x_at	1.00	1.00	P	3027.57	0.27	0.00	P	799.70	HLA-C	6p21.3
227046_at	1.00	0.97	P	355.20	0.27	0.00	P	94.60	SLC39A11	17q25.1
217149_x_at	1.00	0.95	P	379.77	0.27	0.00	P	100.50	TNK1	17p13.1
235604_x_at	0.99	0.92	P	102.77	0.27	0.00	A	27.57	ZNF493	19p13.11
203324_s_at	0.99	0.93	P	2038.10	0.27	0.00	P	541.57	CAV2	7q31.1
202031_s_at	1.00	0.95	P	978.30	0.27	0.00	P	258.97	DKFZP434J154	7p22.2
244139_s_at	1.00	0.95	P	84.50	0.27	0.01	P	22.70	PDCD6	5pter-p15.2
243553_x_at	1.00	0.97	P	179.70	0.27	0.01	P	48.37		
212203_x_at	1.00	0.96	P	1504.33	0.27	0.00	P	401.20	IFITM3	11p15.5
206770_s_at	0.99	0.94	P	741.00	0.27	0.00	P	197.40	SLC35A3	1p21
228698_at	1.00	0.95	P	91.30	0.27	0.01	P	24.70	SOX7	8p22
238022_at	1.00	0.95	P	1238.70	0.27	0.00	P	330.53		16q12.2
231183_s_at	1.00	0.95	P	90.53	0.27	0.00	A	24.17	JAG1	20p12.1-p11.23
239845_at	0.98	0.90	P	65.43	0.27	0.00	P,A	17.43		
203038_at	1.00	1.00	P	1161.00	0.27	0.00	P	309.20	PTPRK	6q22.2-23.1
229692_at	1.00	0.97	P	231.90	0.27	0.00	A	61.90		
244552_at	1.00	0.99	P	144.57	0.27	0.00	A	38.80		19p13.2
228812_at	0.99	0.95	P	137.57	0.27	0.01	P	36.90		
1554906_a_at	0.98	0.91	P	103.10	0.27	0.01	P	27.57	MPHOSPH6	16q23.3
226925_at	1.00	0.98	P	384.33	0.27	0.00	P	102.43	FLJ23751	3q23
1558486_at	1.00	0.98	P	77.10	0.27	0.00	A	20.70	LOC115648	19p13.11
1560647_at	0.97	0.88	P	34.47	0.27	0.03	P	9.77		
211986_at	0.99	0.94	P	1472.90	0.27	0.00	P	394.87	MGC5395	11q12.2

203002_at	0.99	0.94	P	1972.27	0.27	0.00	P	528.93	AMOTL2	3q21-q22
207616_s_at	0.97	0.89	P	930.00	0.27	0.00	P	250.23	TANK	2q24-q31
219406_at	0.99	0.92	P	148.20	0.27	0.00	A	39.70	MGC955	1p34.1
221669_s_at	0.98	0.90	P	573.60	0.27	0.00	P	154.47	ACAD8	11q25
206941_x_at	1.00	0.97	P,M	79.50	0.27	0.01	A	21.43	SEMA3E	7q21.11
200862_at	0.98	0.91	P	2654.03	0.27	0.00	P	708.47	DHCR24	1p33-p31.1
210205_at	0.99	0.94	P	114.77	0.27	0.02	A	31.67	B3GALT4	6p21.3
239891_x_at	0.98	0.91	P	1535.97	0.27	0.00	P	415.13		18p11.22
231644_at	0.99	0.93	P	45.50	0.27	0.01	A	12.60		
202748_at	1.00	0.98	P	280.73	0.27	0.01	P	75.70	GBP2	1p22.2
204862_s_at	1.00	0.95	P	267.97	0.27	0.00	A	71.93	NME3	16q13
215626_at	0.99	0.95	P	68.83	0.27	0.01	P	18.73		
205307_s_at	1.00	0.98	P	35.53	0.27	0.02	A	9.97	KMO	1q42-q44
201422_at	1.00	0.96	P	823.60	0.27	0.00	P	222.30	IFI30	19p13.1
229016_s_at	0.99	0.94	P	52.50	0.27	0.01	A	14.37	TRERF1	6p21.1-p12.1
225703_at	1.00	0.96	P	385.10	0.27	0.00	P	103.73	KIAA1545	12q24.33
1555137_a_at	0.98	0.89	P,A	82.63	0.27	0.01	A	22.77	FGD6	12q23.1
222237_s_at	1.00	0.97	P	83.63	0.27	0.00	P	22.67	ZNF228	19q13.2
211006_s_at	1.00	0.96	P,M	96.97	0.27	0.01	A	26.63	KCNB1	20q13.2
230469_at	1.00	0.98	P	106.87	0.28	0.01	P	29.13	PLEKHK1	10q21.3
215732_s_at	0.99	0.92	P	116.07	0.28	0.02	P,A	32.57	DTX2	7q11.23
242923_at	1.00	0.98	P	124.93	0.28	0.01	P	34.60	MGC15634	1q42.13
235190_at	0.99	0.94	P	543.23	0.28	0.00	P	146.93	CALM2	2p21
225699_at	1.00	0.99	P	1288.93	0.28	0.00	P	349.77	LOC285958	7p13
218263_s_at	1.00	0.99	P	1741.73	0.28	0.00	P	473.07	LOC58486	11p15.3
220760_x_at	0.98	0.89	P	442.47	0.28	0.01	P,M	121.93	FLJ14345	19q13.42
211965_at	0.99	0.93	P	390.47	0.28	0.01	P	107.70	ZFP36L1	14q22-q24
226206_at	1.00	0.99	P	926.83	0.28	0.00	P	252.13	MAFK	7p22.3
217419_x_at	1.00	0.99	P	1285.33	0.28	0.00	P	350.33	AGRN	1p36.33
1565162_s_at	0.97	0.88	P	1909.73	0.28	0.00	P	524.77	MGST1	12p12.3-p12.1
209101_at	0.97	0.87	P	498.73	0.28	0.00	P	136.73	CTGF	6q23.1
205103_at	0.99	0.95	P	326.73	0.28	0.00	M,A	89.13	CROC4	1q23.1
201868_s_at	0.99	0.95	P,A	64.27	0.28	0.02	A	17.97	TBL1X	xp22.3
226501_at	1.00	0.99	P	654.17	0.28	0.00	P	178.67	LOC63929	22q13.31-q13.33
232138_at	0.99	0.94	P	294.20	0.28	0.00	P	80.53	MBNL2	13q32.2
202558_s_at	0.99	0.94	P	544.73	0.28	0.00	P	149.90	STCH	21q11
225502_at	0.97	0.88	P	53.77	0.28	0.01	P,A	15.13	DOCK8	9p24.3
1552633_at	1.00	0.98	P	146.17	0.28	0.00	P	40.13	ZNF101	19p13.11
229385_s_at	1.00	0.99	P	130.80	0.28	0.00	A	35.97	PLAC2	19p13.3
204872_at	1.00	0.97	P	344.40	0.28	0.00	P	94.77	TLE4	9q21.32
232803_at	1.00	0.96	P	54.50	0.28	0.00	P,A	15.03		
227934_at	0.98	0.90	P	265.10	0.28	0.00	P	73.27		
212642_s_at	0.99	0.93	P	341.60	0.28	0.01	P	95.07	HIVEP2	6q23-q24
229660_at	1.00	0.97	P	96.80	0.28	0.01	A	26.77	FLJ31606	16q24.3
238576_at	1.00	1.00	P	118.67	0.28	0.00	P,M,A	33.00		
226127_at	1.00	0.96	P	704.70	0.28	0.00	P	194.37	DEPC-1	11p11.2
218276_s_at	0.99	0.92	P	730.80	0.28	0.00	P	202.40	SAV1	14q13-q23
231853_at	1.00	0.98	P	204.80	0.28	0.00	P	56.53	TUBD1	17q23.2
226360_at	1.00	0.97	P	309.33	0.28	0.00	P	85.70	FLJ12747	22q12.1
223628_at	0.99	0.94	P,A	114.23	0.28	0.01	A	32.37	DKFZp434N035	22q11.21
224952_at	1.00	0.97	P	143.73	0.28	0.01	A	40.03	DKFZP564D166	17q24.1
202554_s_at	1.00	0.98	P	1043.37	0.28	0.00	P	289.47	GSTM3	1p13.3
228661_s_at	1.00	0.98	P	217.30	0.28	0.00	P,M,A	60.20		
225448_at	1.00	0.98	P	758.10	0.28	0.01	P	212.37	NAPG	18p11.21
236862_at	1.00	0.97	P	83.47	0.28	0.02	M,A	23.63	GOPC	6q21
204090_at	1.00	0.95	P	431.50	0.28	0.02	P,A	121.73	STK19	6p21.3
233112_at	0.98	0.91	P	49.90	0.28	0.03	A	14.33		
218913_s_at	0.99	0.94	P	334.00	0.28	0.00	A	93.47	GMIP	19p11-p12
241869_at	1.00	0.98	P	103.83	0.28	0.01	P,M,A	29.20	APOL6	22q13.1
203431_s_at	0.99	0.92	P	177.40	0.28	0.01	P,M,A	49.87	RICS	11q24-q25
213945_s_at	0.98	0.89	P,M,A	52.43	0.28	0.03	A	15.13	NUP210	3p25.2-p25.1
207714_s_at	0.96	0.87	P	505.20	0.28	0.01	A	143.33	SERPINH1	11q13.5
219696_at	0.98	0.89	P	365.23	0.28	0.00	P	101.93	FLJ20054	1q31.3
218242_s_at	0.99	0.95	P	543.57	0.28	0.00	P	151.93	CGI-85	11q13.2
1557103_a_at	1.00	0.97	P	51.50	0.28	0.00	A	14.37	LMTK3	19q13.33
228457_at	1.00	0.96	P	58.50	0.28	0.01	A	16.70		
218706_s_at	0.98	0.90	P	767.50	0.28	0.00	P	215.13	NS3TP2	5q23.3
224495_at	1.00	0.97	P,M,A	105.27	0.28	0.00	A	29.40	MGC10744	17p13.1
219123_at	1.00	0.98	P	424.43	0.28	0.00	P	118.63	ZNF232	17p13-p12
228095_at	1.00	0.97	P	1026.03	0.28	0.01	P	287.03	PHF14	7p21.3
236471_at	1.00	0.96	P	85.07	0.28	0.01	P	23.93	NFE2L3	7p15-p14
225786_at	0.99	0.94	P	421.60	0.28	0.01	P	118.70	FAM36A	1q44
226231_at	1.00	0.96	P	101.17	0.28	0.00	P	28.50	PAWR	12q21
201200_at	1.00	0.99	P	1591.90	0.28	0.00	P	444.90	CREG	1q24
211458_s_at	0.97	0.87	P	358.83	0.28	0.01	P	100.93	GABARAPL1	12p13.31
203394_s_at	0.98	0.91	P	490.07	0.28	0.00	P	138.10	HES1	3q28-q29
231830_x_at	0.99	0.92	P	118.43	0.28	0.00	P,M	33.50	RCP	8p11.22
211502_s_at	1.00	0.97	P,A	63.27	0.28	0.00	A	17.83	PFTK1	7q21-q22
202339_at	0.99	0.94	P	59.23	0.28	0.02	A	17.20	SYMPK	19q13.3
225812_at	0.99	0.93	P	215.70	0.29	0.01	A	61.47		
1558700_s_at	1.00	1.00	P	338.87	0.29	0.01	P	96.47	LOC339324	19q13.13
230708_at	1.00	0.97	P	57.83	0.29	0.01	A	16.53	PRICKLE1	12q12
225867_at	1.00	0.95	P	121.50	0.29	0.00	A	34.57	LOC114990	16p13.3
226576_at	0.99	0.94	P	80.13	0.29	0.01	P	22.97	ARHGAP26	5q31
202429_s_at	0.99	0.94	P	957.63	0.29	0.00	P	271.30	PPP3CA	4q21-q24
238885_at	1.00	0.97	P	172.23	0.29	0.00	A	49.00		
225602_at	0.98	0.90	P	101.27	0.29	0.01	P,M,A	29.07	C9orf19	9p13-p12
232874_at	0.99	0.94	P,M	120.67	0.29	0.01	A	34.73	DOCK9	13q32.3
215359_x_at	1.00	0.97	P	523.87	0.29	0.00	P	148.13	ZNF44	19p13.2
225396_at	1.00	0.99	P	299.83	0.29	0.00	P	85.43	SYNCOILIN	1p34.3-p33
220242_x_at	1.00	0.98	P	99.63	0.29	0.01	P	28.50	FLJ12985	19q13.42
212669_at	0.99	0.94	P	391.63	0.29	0.00	P	111.47	CAMK2G	10q22
201746_at	1.00	0.98	P	1227.20	0.29	0.00	P	349.07	TP53	17p13.1
205781_at	1.00	0.98	P	245.20	0.29	0.00	P	69.83	C16orf7	16q24
226287_at	1.00	0.96	P	1608.73	0.29	0.00	P	458.53	LOC81023	11p14
214016_s_at	1.00	0.97	P	1100.20	0.29	0.01	P	316.57	SFPQ	1p34.3

209205_s_at	0.97	0.88	P	659.67	0.29	0.01	P	191.27	LMO4	1p22.3
1564200_at	1.00	0.96	P	57.17	0.29	0.01	A	16.50		
233907_s_at	1.00	0.96	P	79.13	0.29	0.01	M,A	22.73	LOC199827	1q32.2
202082_s_at	0.99	0.95	P	418.20	0.29	0.01	P	120.03	SEC14L1	17q25.1-17q25.2
235542_at	0.99	0.92	P	416.07	0.29	0.00	P	118.93	MGC22014	2p13.1
216350_s_at	0.99	0.94	P,M,A	56.53	0.29	0.02	A	16.43	ZNF10	12q24.33
239433_at	1.00	0.95	P	166.87	0.29	0.01	P,A	48.33	FLJ23420	19p13.3
226609_at	0.99	0.94	P	522.43	0.29	0.00	P	149.80	DCBLD1	6q22.31
205543_at	0.96	0.86	P,M,A	48.40	0.29	0.01	A	14.13	APG-1	4q28
227034_at	1.00	0.99	P	594.93	0.29	0.00	P	169.87	10-Sep	2q13
229317_at	1.00	0.97	P	141.70	0.29	0.01	P	40.87		
228280_at	0.99	0.95	P	606.23	0.29	0.00	P	174.33	MGC14289	7q34
204462_s_at	0.99	0.93	P	261.50	0.29	0.01	A	76.23	SLC16A2	xq13.2
224917_at	0.99	0.93	P	1580.47	0.29	0.01	P	460.47	VMP1	17q23.2
225079_at	0.99	0.92	P	749.27	0.29	0.00	P	215.33	EMP2	16p13.2
227718_at	1.00	0.99	P	544.30	0.29	0.01	P	156.57	PURB	7p13
211593_s_at	1.00	0.99	P	587.67	0.29	0.00	P	170.13	MAST2	1p34.1
227367_at	0.99	0.95	P	159.03	0.29	0.00	P,A	45.67	SLCO3A1	15q26
219635_at	0.99	0.94	P	129.57	0.29	0.01	P	37.40	ZNF606	19q13.4
203395_s_at	1.00	0.96	P	538.13	0.29	0.00	P	154.70	HES1	3q28-q29
218757_s_at	1.00	0.99	P	2621.93	0.29	0.00	P	752.13	UPF3B	xq25-q26
226040_at	1.00	0.98	P	159.80	0.29	0.01	P	46.23		
225988_at	1.00	0.97	P	1355.37	0.29	0.00	P	389.63	HERC4	10q22.1
228330_at	0.99	0.94	P	335.30	0.29	0.00	P	96.73	C6orf113	6q22.31
205306_x_at	1.00	0.96	P	153.40	0.29	0.00	A	44.40	KMO	1q42-q44
206687_s_at	1.00	0.97	P	164.20	0.29	0.00	P,M,A	47.47	PTPN6	12p13
205294_at	1.00	0.98	P,A	212.70	0.29	0.00	A	61.23	BAIAP2	17q25
214808_at	0.99	0.94	P	190.67	0.29	0.01	P,A	55.07		
215243_s_at	0.99	0.92	P	188.37	0.29	0.03	A	56.97	GJB3	1p34
225211_at	0.99	0.92	P,A	351.33	0.29	0.02	A	102.63	MGC16207	11q23.3
220945_x_at	1.00	0.99	P	950.30	0.29	0.01	P	276.30	MANSC1	12p13.2
226403_at	0.98	0.89	P	156.17	0.29	0.00	A	44.77	TMC4	19q13.42
211962_s_at	0.99	0.94	P	2012.80	0.29	0.00	P	582.83	ZFP36L1	14q22-q24
1556346_at	0.99	0.93	P	233.40	0.29	0.00	P,A	67.23		
221701_s_at	0.99	0.93	P	246.97	0.29	0.00	P,A	71.63	FLJ12541	15q22.33
238859_at	0.99	0.94	P	109.93	0.29	0.00	P	31.97	KIAA0974	10q22.2
201160_s_at	1.00	0.98	P	3131.77	0.29	0.00	P	907.87	CSDA	12p13.1
205807_s_at	0.99	0.95	P	802.30	0.29	0.00	P	231.67	TUFT1	1q21
230026_at	1.00	0.95	P	356.20	0.29	0.00	P	103.43	MRPL43	10q24.31
225541_at	1.00	0.99	P	3319.03	0.29	0.01	P	970.07	LOC200916	3q26.31
55065_at	1.00	0.98	P	415.30	0.30	0.00	P	120.77	MARK4	19q13.3
219001_s_at	1.00	0.98	P	651.87	0.30	0.01	P	190.03	MGC10765	9p13.1
242766_at	1.00	0.99	P	74.57	0.30	0.00	P,A	21.70		
234921_at	1.00	0.96	P	56.57	0.30	0.00	A	16.47		19q13.43
202845_s_at	0.99	0.94	P	2113.93	0.30	0.01	P	617.73	RALBP1	18p11.3
1559361_at	0.99	0.92	P,M	69.63	0.30	0.02	A	21.07		
207417_s_at	0.99	0.94	P	90.17	0.30	0.02	A	26.80	ZNF177	19p13.2
1568868_at	1.00	0.95	P	34.20	0.30	0.02	P,A	10.23		2q21.1
222744_s_at	1.00	0.99	P	265.53	0.30	0.00	P,A	77.37	TMLHE	xq28
225410_at	1.00	0.96	P	935.23	0.30	0.01	A	274.23	MGAT4A	2q12
240221_at	1.00	0.99	P	207.77	0.30	0.00	P	60.63		
236653_at	0.98	0.90	P	63.80	0.30	0.02	P,A	19.33		
231296_at	0.97	0.88	P,M,A	67.87	0.30	0.03	A	20.40		7
227425_at	0.99	0.93	P	95.90	0.30	0.02	P	29.00		
208078_s_at	1.00	0.99	P	334.03	0.30	0.00	P,A	97.57	TCF8	10p11.2
238401_at	0.98	0.91	P,A	113.17	0.30	0.03	A	35.27	FLJ35220	17q25.3
224452_s_at	0.99	0.94	P	643.53	0.30	0.00	P	189.10	MGC12966	7p22.2
226957_x_at	1.00	0.96	P	210.03	0.30	0.00	P,A	62.23	RALBP1	18p11.3
38269_at	0.99	0.94	P	568.60	0.30	0.00	P	166.87	PRKD2	19q13.3
203499_at	1.00	0.98	P	191.67	0.30	0.01	A	56.80	EPHA2	1p36
208807_s_at	0.97	0.89	P	424.77	0.30	0.00	A	124.50	CHD3	17p13.1
212776_s_at	0.98	0.90	P	686.93	0.30	0.00	P	202.63	KIAA0657	2q36.1
1555929_s_at	1.00	0.97	P	73.63	0.30	0.00	A	21.63		
225078_at	1.00	0.96	P	960.10	0.30	0.00	P	281.77	EMP2	16p13.2
200859_x_at	1.00	0.98	P	4023.77	0.30	0.00	P	1184.60	FLNA	xq28
228155_at	1.00	0.99	P	1576.87	0.30	0.00	P	463.70	C10orf58	10q23.1
213485_s_at	1.00	0.99	P	589.30	0.30	0.00	P	173.70	ABCC10	6p21.1
227135_at	0.97	0.89	P	104.13	0.30	0.02	A	31.07	ASAHL	4q21.1
243791_at	0.99	0.94	P	102.80	0.30	0.00	A	30.37		
220174_at	1.00	0.97	P	219.57	0.30	0.01	P	65.60	FLJ23420	19p13.3
214792_x_at	0.99	0.94	P	193.07	0.30	0.00	P	56.83	VAMP2	17p13.1
202738_s_at	1.00	0.97	P	596.27	0.30	0.01	P	178.27	PHKB	16q12-q13
205805_s_at	0.99	0.93	P,A	91.63	0.30	0.01	A	27.53	ROR1	1p32-p31
223519_at	0.99	0.93	P	991.23	0.30	0.01	P	294.90	ZAK	2q24.2
204613_at	0.96	0.86	P	263.53	0.30	0.02	P	79.67	PLCG2	16q24.1
205107_s_at	1.00	0.98	P	135.67	0.30	0.00	P,A	40.20	EFNA4	1q21-q22
205245_at	1.00	0.97	P	81.70	0.30	0.00	P,M	24.50	PARD6A	16q22.1
211732_x_at	0.99	0.95	P	105.67	0.30	0.01	A	31.67	HNMT	2q22.1
226313_at	0.99	0.93	P	295.97	0.30	0.01	P,A	88.10	C10orf35	10q22.2
219439_at	0.99	0.94	P	536.33	0.30	0.00	P	160.60	C1GALT1	7p14-p13
233599_at	0.97	0.87	P	80.07	0.30	0.01	M,A	23.97		
230820_at	0.99	0.91	P	197.50	0.30	0.00	P	59.07	SMURF2	17q22-q23
229404_at	0.99	0.95	P	138.40	0.30	0.00	M,A	41.00	TWIST2	2q37.3
213657_s_at	1.00	0.96	P	235.90	0.30	0.00	P,A	70.60	DKFZp547K1113	15q26.1
238049_at	0.99	0.93	P	83.67	0.30	0.03	P,A	25.63		
207732_s_at	0.99	0.93	P	158.03	0.30	0.00	A	47.23	DLG3	xq13.1
214519_s_at	1.00	0.96	P	114.40	0.30	0.01	A	34.73	RLN2	9p24.1
226485_at	0.99	0.92	P	110.87	0.30	0.01	A	33.63	FLJ20674	12q24.23
220127_s_at	1.00	0.99	P	1218.97	0.30	0.00	P	363.13	FBXL12	19p13.2
204766_s_at	0.99	0.95	P	321.47	0.30	0.01	P	96.70	NUDT1	7p22
208997_s_at	0.98	0.90	P	1284.27	0.30	0.00	P	385.37	UCP2	11q13
213196_at	1.00	0.97	P,M	201.27	0.30	0.02	A	61.80	ZNF629	16p11.2
224618_at	1.00	0.96	P	511.47	0.30	0.00	P	153.03	ROD1	9q33.1
52285_f_at	0.99	0.93	P	441.23	0.30	0.00	P	132.50	C18orf9	18p11.21
213572_s_at	1.00	0.97	P	474.33	0.30	0.00	P	142.63	SERPINB1	6p25
226511_at	0.99	0.93	P	659.83	0.30	0.00	P	198.37		

206558_at	1.00	0.99	P,A	101.70	0.30	0.02	A	31.27	SIM2	21q22.13
1557938_s_at	1.00	0.97	P	393.73	0.30	0.00	P,A	118.07		
211200_s_at	0.99	0.94	P	87.47	0.31	0.02	P	26.87		
54970_at	0.97	0.87	P	1143.40	0.31	0.00	P	340.97	DKFZp761I2123	7p13
207159_x_at	1.00	0.98	P	164.27	0.31	0.00	M,A	49.53	MECT1	19p13.11
203067_at	1.00	0.95	P	1780.20	0.31	0.00	P	536.63	PDHX	11p13
231399_at	1.00	0.98	P	55.73	0.31	0.01	A	17.07		
205917_at	1.00	0.97	P	384.73	0.31	0.00	P	115.67	ZNF264	19q13.4
225807_at	1.00	0.98	P	81.37	0.31	0.00	P	24.50	JUB	14q11.2
219241_x_at	1.00	0.99	P,A	355.60	0.31	0.00	A	107.97	SSH-3	11q13.1
236451_at	0.98	0.89	P	18.63	0.31	0.01	A	5.77		
205037_at	1.00	0.96	P	325.23	0.31	0.00	P	97.90	RABL4	22q13.1
218387_s_at	1.00	0.98	P	1563.67	0.31	0.00	P	472.40	PGLS	19p13.2
1553023_a_at	0.99	0.92	P	102.13	0.31	0.00	P,M	31.07	NOX5	15q22.31
211695_x_at	0.99	0.91	P	147.57	0.31	0.01	A	45.40	MUC1	1q21
223591_at	1.00	0.96	P	265.00	0.31	0.00	P	80.37	RNF135	17q11.2
209305_s_at	0.98	0.91	P	166.97	0.31	0.00	P,A	50.77	GADD45B	19p13.3
231136_at	1.00	0.97	P	378.80	0.31	0.00	P	114.73		
211033_s_at	0.99	0.92	P	486.50	0.31	0.00	P	147.40	PEX7	6q21-q22.2
1565595_at	0.98	0.91	P	66.80	0.31	0.01	A	20.63		
236685_at	0.99	0.94	P	129.10	0.31	0.00	P,A	39.23		
227352_at	1.00	0.97	P	250.40	0.31	0.00	P	75.93	FLJ35119	19p13.2
204264_at	0.98	0.91	P	279.93	0.31	0.02	P	86.53	CPT2	1p32
243179_at	1.00	0.96	P	106.60	0.31	0.01	M,A	32.73		
202331_at	0.99	0.95	P	378.13	0.31	0.00	M,A	114.33	BCKDHA	19q13.1-q13.2
228920_at	1.00	0.96	P	431.13	0.31	0.00	P	130.73	LOC339324	19q13.13
223167_s_at	1.00	0.96	P	90.23	0.31	0.01	P	27.80	USP25	21q11.2
204922_at	0.97	0.88	P	111.87	0.31	0.01	A	34.33	FLJ22531	11q13.1
244664_at	1.00	0.99	P	145.83	0.31	0.00	A	44.73		
206833_s_at	1.00	0.95	P	381.60	0.31	0.01	P	117.13	ACYP2	2p16.2
203956_at	1.00	0.99	P	837.87	0.31	0.01	P	258.27	ZCWCC1	22q12.2
209865_at	1.00	0.99	P	293.27	0.31	0.00	P	89.80	SLC35A3	1p21
220150_s_at	1.00	0.97	P	169.80	0.31	0.02	A	53.40	C6orf60	6q22.31
1569850_at	0.99	0.94	P,A	19.80	0.31	0.03	A	6.23		
223764_x_at	0.99	0.93	P	48.67	0.31	0.02	A	15.37	NIPSNAP3B	9q31.3
223411_at	0.99	0.94	P	431.70	0.31	0.00	P	131.27	AD023	17q25.2
34726_at	0.99	0.92	P	131.53	0.31	0.00	P	40.27	CACNB3	12q13
40850_at	1.00	0.96	P	360.80	0.31	0.01	A	111.23	FKBP8	19p12
1552628_a_at	1.00	0.96	P	824.63	0.31	0.00	P	253.13	FLJ22313	7p14.2
211202_s_at	0.99	0.95	P	498.63	0.31	0.00	P	152.93	JARID1B	1q32.1
204139_x_at	0.99	0.92	P,A	181.00	0.31	0.02	A	56.07	ZNF42	19q13.2-q13.4
239186_at	0.99	0.92	P	123.63	0.31	0.02	A	38.87	MGC39372	6p25.2
1554097_a_at	0.99	0.95	P	208.40	0.31	0.00	M,A	64.13		
224808_s_at	1.00	0.98	P	422.57	0.31	0.00	A	129.37	C7orf20	7p22.3
215001_s_at	1.00	0.98	P	1385.90	0.31	0.00	P	424.97	GLUL	1q31
203430_at	1.00	0.97	P	1770.93	0.31	0.00	P	546.17	HEBP2	6q24
202856_s_at	1.00	0.99	P	68.40	0.31	0.00	A	20.97	SLC16A3	17q25
227582_at	0.98	0.91	P,M,A	109.63	0.31	0.00	A	33.53	MGC33338	1q23.1
206414_s_at	0.99	0.93	P	794.77	0.31	0.00	P	244.87	DDEF2	2p25
218735_s_at	1.00	0.96	P	786.57	0.31	0.01	P	243.40	ZNF544	19q13.43
239164_at	1.00	0.95	P	141.07	0.31	0.00	P	43.43		
1598_g_at	0.99	0.95	P	825.73	0.31	0.00	A	253.57	GAS6	13q34
226981_at	1.00	0.97	P	531.73	0.31	0.01	P	164.23	MLL	11q23
243521_at	1.00	0.99	P	67.70	0.31	0.01	P	21.37		
216526_x_at	1.00	1.00	P	2958.73	0.31	0.00	P	910.73	HLA-C	6p21.3
203620_s_at	0.99	0.95	P,M	211.43	0.31	0.01	A	66.07	FCHSD2	11q13.3
210502_s_at	1.00	0.97	P	1133.80	0.31	0.00	P	350.10	PPIE	1p32
205896_at	1.00	0.98	P,A	159.17	0.31	0.00	A	49.13	SLC22A4	5q31.1
239336_at	0.99	0.94	P	38.87	0.31	0.00	A	12.03		
1557193_at	1.00	0.97	P	154.60	0.31	0.01	P,A	47.83		
226514_at	0.97	0.88	P	117.93	0.31	0.01	A	37.53	ZNF71	19q13.4
217436_x_at	0.98	0.91	P	394.50	0.31	0.01	P	124.03		
224443_at	1.00	0.97	P	146.17	0.31	0.00	P,A	45.57	MGC14801	1q32.3
238714_at	0.99	0.95	P	241.40	0.31	0.01	P,A	75.47		
207326_at	1.00	0.97	P,A	20.90	0.31	0.01	P,A	6.53	BTC	4q13-q21
228528_at	0.99	0.93	P	111.57	0.32	0.01	P	35.00		
222273_at	0.99	0.95	P	125.57	0.32	0.01	P	39.10		
223361_at	1.00	0.97	P	2661.60	0.32	0.00	P	828.87	C6orf115	6q23.3
238802_at	1.00	0.96	P	98.27	0.32	0.02	A	30.97	TYSND1	10q22.2
242053_at	0.99	0.92	P	103.20	0.32	0.01	P,A	32.20	TSGA10	2q11.2
206219_s_at	1.00	0.99	P	107.10	0.32	0.01	A	33.60	VAV1	19p13.2
223796_at	1.00	0.97	P	379.07	0.32	0.00	P	117.63	CNTNAP3	9p12
1559993_at	0.97	0.88	P	138.83	0.32	0.02	P	44.17	BA108L7.2	10q24.32
213137_s_at	1.00	0.96	P	455.47	0.32	0.00	P	142.00	PTPN2	18p11.3-p11.2
238738_at	1.00	0.96	P	87.67	0.32	0.00	P,A	27.20	PSMD7	16q23-q24
214086_s_at	1.00	0.95	P	884.07	0.32	0.01	P	277.17	ADPRTL2	14q11.2-q12
235349_at	1.00	0.97	P	108.70	0.32	0.02	P,M	34.43	FLJ32954	2p22.3
202193_at	1.00	0.97	P	353.20	0.32	0.00	P	109.90	LIMK2	22q12.2
203615_x_at	1.00	0.97	P	636.83	0.32	0.00	P	200.27	SULT1A1	16p12.1
65438_at	1.00	0.97	P	113.53	0.32	0.00	P	35.50	KIAA1609	16q24.1
209432_s_at	1.00	0.99	P	1001.83	0.32	0.00	P	312.97	CREB3	9pter-p22.1
220987_s_at	0.99	0.95	P	223.13	0.32	0.00	P	69.67	SNARK	1q32.1
210791_s_at	0.99	0.92	P	150.80	0.32	0.01	A	48.27	RICS	11q24-q25
1556638_at	0.99	0.92	P,M	149.90	0.32	0.00	A	47.10	LOC284530	1p36.13
213853_at	1.00	0.97	P	826.50	0.32	0.00	P	260.57	LOC120526	11p14.1
243016_at	1.00	0.97	P	166.80	0.32	0.01	P	52.53	TYMS	18p11.32
238402_s_at	0.99	0.93	P,M	150.60	0.32	0.00	A	47.00	FLJ35220	17q25.3
235398_at	0.99	0.91	P	112.17	0.32	0.01	P	35.33		
235363_at	1.00	0.98	P	110.57	0.32	0.01	P	35.40		
218412_s_at	1.00	0.96	P	666.90	0.32	0.00	P	209.70	GTF2IRD1	7q11.23
219078_at	1.00	0.99	P	280.27	0.32	0.00	P,M,A	88.13	GPATC2	1q41
202321_at	0.99	0.93	P	167.37	0.32	0.01	P	52.93	GGPS1	1q43
213136_at	1.00	0.95	P	1117.23	0.32	0.00	P	350.50	PTPN2	18p11.3-p11.2
225840_at	1.00	0.95	P	271.10	0.32	0.01	P,M	85.57	TEF	22q13.2
226787_at	0.99	0.92	P	78.83	0.32	0.01	A	24.93	ZNF18	17p11.2
51192_at	0.99	0.95	P	336.73	0.32	0.00	P	106.47	SSH-3	11q13.1

218371_s_at	1.00	0.99	P	531.17	0.32	0.00	P	167.27	PSPC1	13q12.11
238881_at	0.99	0.94	P	56.27	0.32	0.02	P,M,A	18.17		
202138_x_at	1.00	0.99	P	2079.73	0.32	0.01	P	659.17	JTV1	7p22
223608_at	1.00	0.96	P	253.50	0.32	0.00	P	80.17	MGC12458	1q44
238462_at	1.00	0.96	P	596.33	0.32	0.00	P	188.40	KIAA1959	11q24.1
207128_s_at	0.98	0.91	P	108.13	0.32	0.01	P	34.10	ZNF223	19q13.2
205632_s_at	1.00	0.98	P	146.83	0.32	0.00	A	46.67	PIP5K1B	9q13
232422_at	0.99	0.92	P	118.67	0.32	0.00	A	37.93	LOC87769	13q32.3
204291_at	1.00	0.98	P	154.00	0.32	0.01	P	48.93	ZNF518	10q24.1
210705_s_at	1.00	0.97	P	266.27	0.32	0.01	P	84.27	TRIM5	11p15
204542_at	1.00	0.97	P	436.27	0.32	0.01	P,M	139.27	SIAT7B	17q25.3
230874_at	0.98	0.89	P	42.73	0.32	0.01	P	13.70		
225774_at	0.98	0.91	P	536.40	0.32	0.00	P	171.07	KIAA1972	16q13
225311_at	1.00	0.97	P	283.27	0.32	0.01	P,M,A	90.13	IVD	15q14-q15
217200_x_at	1.00	0.96	P	784.90	0.32	0.00	P	249.80	CYB561	17q11-qter
238712_at	1.00	0.97	P	74.73	0.32	0.01	P,A	23.93		
213622_at	1.00	0.95	P	184.23	0.32	0.00	P	58.90	COL9A2	1p33-p32
228652_at	1.00	0.98	P	419.20	0.32	0.00	P	133.60	FLJ38288	19q13.43
222691_at	1.00	0.97	P	550.60	0.32	0.00	P	175.33	SLC35B3	6p24.3
225654_at	0.99	0.93	P	207.90	0.32	0.01	P,A	66.83	LOC202347	5q35.3
228208_x_at	0.99	0.94	P	181.60	0.32	0.00	A	57.90	ZNF354C	5q35
202457_s_at	0.98	0.91	P	755.67	0.32	0.01	P	243.83	PPP3CA	4q21-q24
202432_at	1.00	0.99	P	467.87	0.32	0.00	P	149.03	PPP3CB	10q21-q22
203777_s_at	0.99	0.94	P	409.63	0.32	0.00	P	130.30	RPS6KB2	11q13.1
219735_s_at	0.99	0.93	P,M	218.90	0.32	0.00	A	70.47	TFCP2L1	2q14
208936_x_at	0.97	0.88	P,A	136.50	0.32	0.01	A	44.33	LGALS8	1q42-q43
228289_at	1.00	0.98	P	302.67	0.32	0.00	P	96.93	ADCY7	16q12-q13
223425_at	1.00	0.98	P	893.33	0.32	0.00	P	285.93	RAVER1	19p13.2
224992_s_at	0.99	0.92	P	425.40	0.32	0.01	P	136.77	CMIP	16q23
230733_at	0.99	0.93	P	332.73	0.32	0.00	P	106.63		
222073_at	1.00	0.96	P	66.47	0.32	0.01	P	21.73	COL4A3	2q36-q37
222737_s_at	1.00	0.97	P	1340.40	0.32	0.00	P	429.27	BRD7	16q12
219603_s_at	1.00	0.96	P	241.30	0.33	0.00	P	77.10	ZNF226	19q13.2
216060_s_at	1.00	0.96	P	582.90	0.33	0.00	P	187.07	DAAM1	14q23.1
228654_at	1.00	0.97	P	2195.40	0.33	0.00	P	704.23	LOC139886	xq11.2
220076_at	1.00	0.96	P	73.03	0.33	0.01	A	23.50	ANKH	5p15.1
226962_at	1.00	0.98	P	611.87	0.33	0.00	P	196.47	FRBZ1	1q31.3
209129_at	1.00	0.99	P	718.07	0.33	0.00	P	230.17	TRIP6	7q22
232228_at	0.99	0.93	P	73.43	0.33	0.00	A	23.63	ZNF530	19q13.43
227255_at	1.00	0.98	P	258.70	0.33	0.00	P	83.00	LOC149420	1p35.3
241933_at	1.00	0.95	P	174.47	0.33	0.01	P	56.77	QRSL1	6q21
218377_s_at	0.99	0.95	P	679.63	0.33	0.01	A	218.80	C21orf6	21q22.11
212630_at	0.99	0.94	P	514.83	0.33	0.01	P	165.33	SEC6L1	5p15.33
241410_at	0.98	0.91	P	77.37	0.33	0.02	A	25.13		
214733_s_at	1.00	0.99	P	496.70	0.33	0.01	P,M,A	161.07	DJ167A19.1	1p33-p32.1
203306_s_at	1.00	0.98	P	1321.53	0.33	0.00	P	425.23	SLC35A1	6q15
1558626_at	1.00	0.95	P	40.30	0.33	0.01	A	13.03		
230643_at	1.00	0.97	P	44.57	0.33	0.00	M,A	14.33	WNT9A	1q42
209061_at	1.00	0.99	P	299.10	0.33	0.01	P	96.37	NCOA3	20q12
201012_at	0.99	0.92	P	3235.33	0.33	0.00	P	1044.30	ANXA1	9q12-q21.2
236476_at	1.00	0.98	P	314.03	0.33	0.02	P	102.40		
225066_at	1.00	0.99	P	162.87	0.33	0.00	P	52.37	PPP2R2D	10q26.3
226065_at	1.00	0.96	P	185.77	0.33	0.01	A	61.17	PRICKLE1	12q12
229014_at	1.00	0.99	P,M	318.30	0.33	0.01	A	103.80		5q15
224415_s_at	1.00	0.98	P	2824.43	0.33	0.00	P	907.57	HINT2	9p13.1
203771_s_at	0.99	0.93	P	178.03	0.33	0.01	A	58.07	BLVRA	7p14-cen
227616_at	0.97	0.88	P	480.90	0.33	0.00	P,M,A	154.27	BCL9L	11q23.3
235538_at	1.00	0.96	P	141.87	0.33	0.00	A	45.77		
1561720_at	0.99	0.93	P	22.23	0.33	0.02	P,A	7.33		
201387_s_at	1.00	0.98	P,M	106.77	0.33	0.02	A	35.50	UCHL1	4p14
1557046_x_at	1.00	0.95	P,A	28.43	0.33	0.02	A	9.37		
223311_s_at	1.00	0.98	P	344.77	0.33	0.01	M,A	112.57	MTA3	2p22.1
228868_x_at	0.99	0.93	P	975.83	0.33	0.02	P	320.50	CDT1	16q24.3
1562259_at	1.00	0.96	P	64.30	0.33	0.00	P	20.83	LOC161577	15q21.2
242343_x_at	0.98	0.91	P	127.30	0.33	0.03	P	42.33		
225009_at	0.99	0.92	P	658.13	0.33	0.00	P	212.10	CKLFSF4	16q22.1
235559_at	0.98	0.91	P	143.83	0.33	0.01	P,M,A	46.93	FLJ22374	7p15.1
225421_at	1.00	0.98	P	1095.47	0.33	0.01	P	358.73	ACY1L2	6q16.1
238773_at	1.00	0.98	P	569.83	0.33	0.00	P	184.87	FLJ33979	11p14.1
202620_s_at	1.00	0.96	P	1526.13	0.33	0.00	P	496.10	PLOD2	3q23-q24
223811_s_at	1.00	0.99	P	354.97	0.33	0.01	P	115.77	C7orf20	7p22.3
238884_at	1.00	0.97	P	155.10	0.33	0.02	P,A	51.17		
1553613_s_at	1.00	0.97	P	128.40	0.33	0.00	P	41.67	FOXC1	6p25
202636_at	1.00	0.97	P	619.97	0.33	0.01	P	202.30	RNF103	2p11.2
219311_at	0.99	0.94	P	259.03	0.33	0.01	A	84.73	C18orf9	18p11.21
225901_at	0.99	0.95	P	394.17	0.33	0.01	P	129.03	LOC114971	11p11.2
214910_s_at	1.00	0.95	P	208.77	0.33	0.01	A	68.70	APOM	6p21.31
204523_at	0.99	0.93	P	229.90	0.33	0.00	P	75.27	ZNF140	12q24.32-q24.33
219027_s_at	1.00	0.99	P,A	190.83	0.33	0.01	A	62.67	MYO9A	15q22-q23
229596_at	0.99	0.95	P	42.60	0.33	0.02	A	14.17	MGC35366	12q23.1
210166_at	0.99	0.92	P,A	111.43	0.33	0.00	A	36.43	TLR5	1q41-q42
202053_s_at	0.99	0.94	P	945.27	0.33	0.00	P	308.70	ALDH3A2	17p11.2
227148_at	0.99	0.92	P	71.83	0.33	0.01	P	23.80	KIAA2028	2p22.1
225133_at	0.98	0.89	P	511.23	0.33	0.00	P	167.57	KLF3	4p14
203274_at	0.99	0.95	P	1678.50	0.33	0.00	P	548.60	F8A	xq28
238025_at	1.00	0.98	P	341.27	0.33	0.00	P	111.40	FLJ34389	16q22.3
201791_s_at	1.00	0.99	P	1665.93	0.33	0.00	P	543.10	DHCR7	11q13.2-q13.5
223551_at	0.99	0.93	P	41.20	0.33	0.02	P,A	13.87	PKIB	6q22.32
214459_x_at	1.00	0.97	P	2556.13	0.33	0.00	P	836.83	HLA-C	6p21.3
36920_at	1.00	0.96	P	303.87	0.33	0.01	P	99.87	MTM1	xq28
225770_at	0.99	0.92	P	436.40	0.33	0.01	P	145.00	KIAA1972	16q13
230556_at	1.00	0.95	P	358.53	0.33	0.00	P	117.83	FLJ25059	11p14.1
205406_s_at	1.00	0.96	P	133.57	0.33	0.00	P	43.67	SPA17	11q24.2
214719_at	1.00	0.95	P	164.57	0.33	0.01	A	54.20	LOC283537	13q12.3
215903_s_at	1.00	0.95	P	465.33	0.33	0.01	P	152.63	MAST2	1p34.1
209716_at	0.99	0.92	P,A	169.40	0.33	0.00	P,A	55.67	CSF1	1p21-p13

201287_s_at	1.00	0.97	P	1583.37	0.33	0.00	P	519.43	SDC1	2p24.1
204334_at	1.00	0.96	P	187.53	0.33	0.01	P	62.20	KLF7	2q32
202869_at	0.99	0.92	P	237.13	0.33	0.00	A	77.47	OAS1	12q24.1
201326_at	1.00	0.99	P	3269.73	0.33	0.01	P	1079.13	CCT6A	7p11.2
231736_x_at	0.98	0.91	P	2497.90	0.33	0.00	P	829.93	MGST1	12p12.3-p12.1
222640_at	1.00	0.96	P	549.80	0.33	0.00	A	180.33	DNMT3A	2p23
230083_at	0.99	0.94	P	187.63	0.33	0.01	P	61.90	USP53	4q27
202758_s_at	1.00	0.95	P	564.60	0.33	0.00	P	185.50	RFXANK	19p12
242562_at	1.00	0.95	P	88.00	0.33	0.01	P	29.07	LOC120526	11p14.1
204453_at	1.00	0.96	P	165.90	0.33	0.01	P	55.30	ZNF84	12q24.33
222804_x_at	0.99	0.94	P	908.53	0.33	0.00	P	300.20	MGC10765	9p13.1
230002_at	1.00	0.98	P	419.93	0.33	0.00	P	138.43	MCLC	1p13.3
221215_s_at	1.00	0.97	P	1231.37	0.34	0.00	P	405.97	ANKRD3	21q22.3
227542_at	1.00	0.98	P	177.10	0.34	0.01	P	58.67		
207986_x_at	1.00	0.97	P	400.93	0.34	0.00	P	132.53	CYB561	17q11-qter
219916_s_at	0.99	0.95	P	296.60	0.34	0.00	A	98.27	RNF39	6p21.3
219705_at	1.00	0.98	P	253.37	0.34	0.00	P	83.67	FLJ21924	11p13
218634_at	1.00	0.99	P	212.30	0.34	0.00	A	70.23	PHLDA3	1q31
204255_s_at	0.99	0.94	P	104.07	0.34	0.01	P	34.80	VDR	12q12-q14
224747_at	1.00	0.98	P	721.63	0.34	0.00	P	239.40	LOC92912	15q23
202712_s_at	1.00	0.96	P	612.67	0.34	0.01	P	203.20	CKMT1	15q15
233142_at	0.98	0.90	P,A	126.10	0.34	0.01	A	42.40		
229532_at	1.00	0.98	P	64.53	0.34	0.01	A	21.80	ZNF502	3p21.32
233759_s_at	1.00	0.96	P	752.50	0.34	0.01	P	250.80	KIAA1387	2p16.2
229143_at	0.99	0.92	P	210.80	0.34	0.01	P	69.93	CNOT3	19q13.4
1570165_at	0.99	0.94	P	74.20	0.34	0.02	P	25.40		
224569_s_at	1.00	0.97	P	2986.30	0.34	0.00	P	994.07	IRF2BP2	1q42.3
212415_at	1.00	0.98	P	267.37	0.34	0.00	A	89.13	6-Sep	xq25
202084_s_at	0.99	0.95	P	965.73	0.34	0.00	P	322.10	SEC14L1	17q25.1-17q25.2
211721_s_at	1.00	0.96	P	265.63	0.34	0.01	P	89.33	ZNF551	19q13.43
1556160_a_at	1.00	0.95	P	75.40	0.34	0.01	A	25.63		
202449_s_at	0.99	0.92	P	443.23	0.34	0.00	P	147.47	RXRA	9q34.3
211924_s_at	0.99	0.93	P	199.63	0.34	0.01	A	67.87	PLAUR	19q13
1555136_at	0.99	0.94	P	35.47	0.34	0.01	A	11.93	FGD6	12q23.1
205251_at	1.00	0.96	P	299.03	0.34	0.01	P	100.63	PER2	2q37.3
225698_at	1.00	0.96	P	924.83	0.34	0.00	P	309.00	TIGA1	5q21-q22
214639_s_at	0.99	0.93	P,A	130.77	0.34	0.00	A	44.17	HOXA1	7p15.3
227234_at	0.99	0.94	P	92.67	0.34	0.02	A	31.53		
222408_s_at	1.00	0.97	P	504.80	0.34	0.00	P	169.37	CGI-127	2p23.3
223575_at	0.99	0.93	P	182.93	0.34	0.01	P	61.70	KIAA1549	7q34
209210_s_at	1.00	0.98	P	976.97	0.34	0.00	P	327.50	PLEKHC1	14q22.1
216836_s_at	1.00	0.97	P	221.90	0.34	0.01	M,A	75.37	ERBB2	17q21.1
235581_at	1.00	0.96	P	106.23	0.34	0.01	P	36.37		
202927_at	1.00	0.98	P	993.87	0.34	0.01	P	335.50	PIN1	19p13
229272_at	0.98	0.90	P	233.87	0.34	0.00	M,A	78.77	FNBP4	11p11.2-p11.12
201333_s_at	1.00	0.98	P	238.43	0.34	0.01	A	81.07	ARHGEF12	11q23.3
213593_s_at	0.99	0.92	P	194.93	0.34	0.02	P,A	66.23	TRA2A	7p15.3
239679_at	0.99	0.94	P	97.07	0.34	0.00	P	32.60		
225662_at	1.00	0.97	P	818.57	0.34	0.00	P	275.53	ZAK	2q24.2
212074_at	1.00	0.95	P	1391.23	0.34	0.00	P	469.90	UNC84A	7p22.3
239848_at	1.00	0.97	P	144.33	0.34	0.02	P,A	49.20		
221599_at	0.99	0.94	P	208.77	0.34	0.02	P,M,A	71.23	PTD015	11q13.4
1560089_at	0.98	0.91	P	131.97	0.34	0.03	A	45.27	LOC286208	9q34.13
225002_s_at	1.00	0.99	P	625.47	0.34	0.00	P	210.90	SUMF2	7q11.1
225773_at	1.00	0.99	P	607.57	0.34	0.00	P	205.27	KIAA1972	16q13
214239_x_at	1.00	0.96	P	512.70	0.34	0.01	P	173.17	RNF110	17q21.2
221931_s_at	1.00	0.98	P	2372.07	0.34	0.00	P	801.73	SEC13L	18p11.21
224570_s_at	1.00	0.99	P	1255.03	0.34	0.00	P	424.90	IRF2BP2	1q42.3
221493_at	1.00	0.99	P	1877.50	0.34	0.00	P	633.90	TSPYL1	6q22-q23
227979_at	0.99	0.95	P	519.70	0.34	0.01	P	175.83	MGC10871	11q13
240910_at	0.99	0.95	P,M	102.23	0.34	0.01	A	35.03		
227465_at	1.00	0.99	P	410.60	0.34	0.00	P	138.97	KIAA0892	19p13.11
233461_x_at	0.99	0.94	P	139.53	0.34	0.00	P	47.20	ZNF226	19q13.2
209632_at	0.97	0.87	P	298.67	0.34	0.01	P	102.47	PPP2R3A	3q22.1
219076_s_at	1.00	0.99	P	367.37	0.34	0.01	A	126.63	PXMP2	12q24.33
204034_at	1.00	0.98	P	1187.53	0.34	0.00	P	407.17	ETHE1	19q13.32
224839_s_at	0.99	0.92	P	617.53	0.35	0.03	P	214.43	GPT2	16q12.1
32094_at	1.00	0.97	P	266.23	0.35	0.01	P	90.70	CHST3	10q22.2
236078_at	0.99	0.92	P	67.40	0.35	0.02	M,A	23.13		
229537_at	0.99	0.95	P,A	69.40	0.35	0.00	A	23.67	LMO4	1p22.3
203740_at	0.99	0.94	P	1614.53	0.35	0.00	P	550.97	MPHOSPH6	16q23.3
212099_at	0.99	0.95	P	1230.10	0.35	0.00	P	420.13	RHOB	2p24
204290_s_at	0.99	0.94	P	173.00	0.35	0.01	A	59.53	ALDH6A1	14q24.3
211729_x_at	1.00	0.99	P	1113.43	0.35	0.00	P	379.57	BLVRA	7p14-cen
208107_s_at	0.99	0.95	P	287.67	0.35	0.01	P,A	98.10	LOC81691	16p13.11
212778_at	1.00	0.96	P,M	270.33	0.35	0.01	A	92.43	KIAA0602	14q32.33
219127_at	1.00	0.99	P	114.47	0.35	0.02	A	40.13	MGC11242	17q21.32
201327_s_at	1.00	0.96	P	4824.57	0.35	0.00	P	1650.47	CCT6A	7p11.2
235896_s_at	1.00	0.96	P	128.07	0.35	0.00	P,A	43.63	SMCR7	17p11.2
228466_at	1.00	0.96	P	263.40	0.35	0.01	A	91.30		
202494_at	1.00	0.97	P	481.37	0.35	0.00	P	164.10	PPIE	1p32
226451_at	1.00	0.97	P	994.70	0.35	0.00	P	341.40	MGC19604	19p13.2
210346_s_at	1.00	1.00	P	543.67	0.35	0.01	P	186.73	CLK1	2q33
236150_at	1.00	0.99	P	144.30	0.35	0.00	A	49.50		15q24.1
208581_x_at	0.99	0.95	P	3121.77	0.35	0.00	P	1072.87	MT1X	16q13
220968_s_at	0.99	0.95	P	217.10	0.35	0.00	P,A	74.57	PP1057	12p13.3
232231_at	1.00	0.99	P	178.57	0.35	0.00	P	61.27		
1555762_s_at	1.00	0.97	P	403.87	0.35	0.01	P	139.50	RBM15	1p13
213281_at	0.97	0.89	P	121.73	0.35	0.00	P	41.73	JUN	1p32-p31
218011_at	1.00	0.97	P	3256.83	0.35	0.00	P	1119.03	UBL5	19p13.3
1557128_at	0.99	0.94	P	233.33	0.35	0.01	M,A	80.63	CANP	11q12.2
227776_at	1.00	0.96	P	892.03	0.35	0.01	P	307.50		
229654_at	0.99	0.95	P,A	188.27	0.35	0.00	A	64.93	ZNF44	19p13.2
211423_s_at	0.99	0.95	P	1934.77	0.35	0.00	P	667.63	SC5DL	11q23.3
222810_s_at	1.00	0.97	P,A	507.23	0.35	0.00	A	174.67	RASAL2	1q24
231872_at	0.99	0.93	P	91.07	0.35	0.00	P,M	31.37	KIAA1764	8q21.13-q21.2

212080_at	1.00	0.96	P	479.80	0.35	0.00	M,A	165.03	MLL	11q23
202597_at	1.00	0.96	P	268.80	0.35	0.02	A	94.90	IRF6	1q32.3-q41
238021_s_at	1.00	0.98	P	3098.93	0.35	0.00	P	1068.67		16q12.2
1561749_at	1.00	0.97	P	185.77	0.35	0.02	P	65.87		
217824_at	0.98	0.91	P	203.17	0.35	0.01	P	70.97	UBE2J1	6q16.1
219848_s_at	1.00	0.95	P	191.03	0.35	0.01	P	66.07	ZNF432	19q13.41
229130_at	0.99	0.93	P,M	86.33	0.35	0.03	A	30.53	LOC285535	4p16.1
214093_s_at	1.00	0.97	P	371.07	0.35	0.00	P	128.37	NEXN	1p31.1
229732_at	1.00	0.98	P	169.47	0.35	0.01	P	58.90	HSZFP36	19p13.2
239726_at	1.00	0.99	P	91.77	0.35	0.00	P	31.73	ANK3	10q21
202387_at	1.00	0.98	P	983.03	0.35	0.00	P	339.63	BAG1	9p12
209431_s_at	0.99	0.95	P	124.03	0.35	0.02	A	43.43	ZNF278	22q12.2
243309_at	1.00	0.97	P	144.33	0.35	0.00	P	49.93		15q21.1
226265_at	1.00	0.99	P	2622.33	0.35	0.00	P	906.77	FLJ21924	11p13
229202_at	0.99	0.94	P	156.17	0.35	0.00	P,M	54.23	FLJ11383	1q42.2
211846_s_at	1.00	1.00	P	90.17	0.35	0.01	M,A	31.73	PVRL1	11q23
212332_at	0.99	0.92	P,M	213.40	0.35	0.00	P,A	74.20	RBL2	16q12.2
228006_at	1.00	0.95	P	229.30	0.35	0.00	P	79.50	PTEN	10q23.3
221710_x_at	0.99	0.93	P,A	61.00	0.35	0.03	A	21.87	FLJ10647	1p34.3
230660_at	0.99	0.93	P	346.27	0.35	0.00	P,A	119.83	SERTAD4	1q32.1-q41
229574_at	0.99	0.92	P	93.20	0.35	0.03	A	33.30	TRA2A	7p15.3
239449_at	0.99	0.93	P,A	39.90	0.35	0.01	A	14.03		
219174_at	0.99	0.92	P	91.37	0.35	0.02	P	32.17	CCDC2	9p21.1
210609_s_at	1.00	0.96	P	414.10	0.35	0.00	A	144.53	TP53I3	2p24.1
209194_at	1.00	0.97	P	1348.27	0.35	0.00	P	468.87	CETN2	xq28
210224_at	0.99	0.93	P,M,A	114.27	0.35	0.00	A	39.97	MR1	1q25.3
226408_at	1.00	0.96	P	291.07	0.35	0.00	P,M	101.40	TEAD2	19q13.3
237942_at	0.99	0.94	P	51.40	0.35	0.01	A	17.93	SNRK	3p21.32
206307_s_at	1.00	0.97	P	249.43	0.35	0.00	P	86.90	FOXD1	5q12-q13
231960_at	1.00	0.97	P	113.00	0.35	0.01	P	39.63	C21orf107	21q22.2
238510_at	0.99	0.94	P	195.17	0.35	0.01	P,M,A	68.00	LOC124411	16p11.2
203347_s_at	0.98	0.91	P	256.90	0.35	0.00	P	89.90	M96	1p22.1
224082_at	0.99	0.93	P,A	55.00	0.35	0.01	A	19.33		
213746_s_at	0.99	0.95	P	2357.97	0.35	0.01	P	827.83	FLNA	xq28
219058_x_at	0.98	0.89	P,A	105.17	0.35	0.02	A	37.77	LCN7	1p35.1
240421_x_at	1.00	0.96	P	156.60	0.35	0.01	P	54.73		4q31.1
201064_s_at	0.99	0.94	P	2815.53	0.35	0.00	P	985.67	PABPC4	1p32-p36
224918_x_at	0.99	0.94	P	2722.33	0.36	0.00	P	960.23	MGST1	12p12.3-p12.1
242196_at	0.99	0.95	P	109.77	0.36	0.00	P,M,A	38.43	RICS	11q24-q25
210172_at	1.00	0.96	P	522.60	0.36	0.01	P	184.07	SF1	11q13
201830_s_at	0.99	0.92	P	869.13	0.36	0.01	P	307.63	NET1	10p15
223599_at	1.00	0.97	P	224.67	0.36	0.01	P	79.13	TRIM6	11p15.4
231727_s_at	0.99	0.93	P	340.90	0.36	0.00	P	120.60	AD023	17q25.2
211168_s_at	1.00	0.98	P	695.43	0.36	0.00	P	243.90	RENT1	19p13.2-p13.11
235612_at	1.00	1.00	P	209.00	0.36	0.00	P	73.33		
241827_at	1.00	0.97	P	143.30	0.36	0.02	P,A	51.43	ZNF615	19q13.41
219495_s_at	1.00	0.96	P	179.73	0.36	0.01	P,A	63.47	ZNF180	19q13.2
232696_at	1.00	0.98	P	67.63	0.36	0.02	P	24.13		
224912_at	0.99	0.94	P	126.87	0.36	0.01	P,A	44.63	TTC7	2p21
225023_at	0.97	0.88	P	339.50	0.36	0.01	P	121.03	GOPC	6q21
226473_at	0.99	0.92	P	481.20	0.36	0.00	P	168.90	CBX2	17q25.3
218435_at	1.00	0.97	P	1588.10	0.36	0.00	P	559.27	DNAJD1	13q14.1
223455_at	0.99	0.94	P	659.50	0.36	0.00	P	232.33	MGC10854	12q24.12
224467_s_at	1.00	0.97	P	604.53	0.36	0.00	P	213.13	MGC13096	19q13.12
227208_at	1.00	0.98	P	394.77	0.36	0.00	P	139.07	DLNB14	11q23.3
213396_s_at	0.99	0.94	P	203.40	0.36	0.01	P	72.73	AKAP10	17p11.1
238597_at	1.00	0.97	P	106.43	0.36	0.01	P	37.67	DKFZP566D1346	1p32.3-p31.3
1557684_at	1.00	0.96	P	116.77	0.36	0.02	P	42.27	ZNF286	17p11.2
232156_at	1.00	0.98	P	127.40	0.36	0.01	P	45.13	CMYA5	5q14.1
204806_x_at	0.99	0.95	P	767.27	0.36	0.00	P	271.27	HLA-F	6p21.3
221875_x_at	1.00	0.97	P	1089.10	0.36	0.00	P	384.17	HLA-F	6p21.3
226994_at	1.00	0.99	P	934.37	0.36	0.00	P	330.07	DNAJA2	16q11.1-q11.2
228739_at	1.00	0.96	P	39.50	0.36	0.02	A	14.23		
221776_s_at	1.00	0.96	P	1195.80	0.36	0.00	P	423.77	BRD7	16q12
234937_x_at	0.98	0.90	P	95.20	0.36	0.00	A	33.87	ZFP28	19q13.43
213718_at	0.99	0.93	P	102.40	0.36	0.02	P,A	36.70	RBM4	11q13
207153_s_at	1.00	0.98	P	316.50	0.36	0.00	P	112.27	GLMN	1p22.1
212221_x_at	1.00	0.99	P	1245.57	0.36	0.00	P	441.30	IDS	xq28
227083_at	1.00	0.98	P	152.73	0.36	0.01	P	54.63	B3GTL	13q12.3
234192_s_at	0.98	0.90	P	93.23	0.36	0.03	P	33.97	GKAP1	9q22.1
208988_at	0.99	0.95	P	665.53	0.36	0.02	P	238.40	FBXL11	11q13.1
1557116_at	1.00	0.98	P	244.47	0.36	0.01	P	88.20		
224361_s_at	0.98	0.91	P	106.53	0.36	0.00	A	38.00	IL17RB	3p21.1
203439_s_at	1.00	0.97	P	120.87	0.36	0.01	P	43.17	STC2	5q35.2
212463_at	1.00	0.97	P	1228.50	0.36	0.01	P	437.90	CD59	11p13
230516_at	1.00	0.96	P	51.40	0.36	0.01	P,M,A	18.57	C7orf30	7p15.3
205380_at	1.00	0.96	P,A	39.63	0.36	0.00	A	14.07	PDZK1	1q21
214752_x_at	1.00	0.98	P	2613.63	0.36	0.00	P	930.23	FLNA	xq28
211998_at	0.99	0.95	P	608.80	0.36	0.00	P	216.27	H3F3B	17q25
204282_s_at	0.98	0.90	P	338.40	0.36	0.01	P	120.53	FARS1	6p25.1
211138_s_at	1.00	0.96	P	187.73	0.36	0.01	M,A	67.60	KMO	1q42-q44
1557270_at	1.00	0.96	P	279.17	0.36	0.02	P	101.23		
205682_x_at	1.00	0.95	P	268.17	0.36	0.00	P	95.40	APOM	6p21.31
204800_s_at	0.99	0.94	P	133.23	0.36	0.00	M,A	47.77	FLJ13639	13q14.2
1552643_at	0.99	0.94	P,M	118.70	0.36	0.01	A	43.07	ZNF626	19p13.11
37590_g_at	1.00	0.98	P	158.30	0.36	0.01	A	57.07	DKFZp547K1113	15q26.1
202049_s_at	1.00	0.96	P	365.10	0.36	0.01	P	131.33	ZNF262	1p32-p34
206959_s_at	0.98	0.90	P	188.43	0.36	0.01	P	67.70	UPF3A	13q34
227726_at	0.99	0.94	P	255.50	0.36	0.00	P	91.40	MGC2647	16q24.3
219860_at	0.99	0.92	P	95.97	0.36	0.00	M,A	34.13	LY6G5C	6p21.31
235716_at	0.99	0.93	P	259.27	0.36	0.02	P	94.57		
1563549_a_at	1.00	0.97	P,A	310.83	0.36	0.00	A	110.97	KIAA1623	19p13.12
235414_at	0.98	0.91	P,A	63.63	0.36	0.03	A	23.67	ZNF383	19q13.13
1555559_s_at	0.99	0.92	P,A	37.43	0.36	0.02	P,A	13.67	USP25	21q11.2
205797_s_at	0.98	0.89	P,A	85.90	0.36	0.03	A	31.57	FLJ11336	11p13
226990_at	1.00	0.96	P	1122.37	0.36	0.00	P	401.80	M11S1	11p13

202085_at	1.00	1.00	P	911.97	0.36	0.00	P	327.07	TJP2	9q13-q21
209751_s_at	1.00	0.97	P	593.27	0.36	0.00	P	212.53	SEDLP	19q13.4
205443_at	0.99	0.93	P	385.93	0.36	0.02	P	140.67	SNAPC1	14q22
226021_at	0.98	0.91	P	582.17	0.36	0.00	P	209.17	RDH10	8q13.3
242708_at	0.98	0.91	P	32.80	0.36	0.01	M,A	11.87	PEX1	7q21-q22
219680_at	1.00	0.96	P,A	278.00	0.36	0.00	A	99.57	NOD9	11q23.3
244677_at	0.98	0.90	P	74.00	0.36	0.02	P,A	26.87	PER1	17p13.1-17p12
229741_at	1.00	0.99	P	177.47	0.36	0.00	P	63.50		
226346_at	1.00	0.97	P	150.67	0.36	0.00	P	54.03		
205290_s_at	0.98	0.91	P,A	59.73	0.36	0.00	A	21.50	BMP2	20p12
205323_s_at	1.00	0.97	P	286.47	0.36	0.01	P	103.63	MTF1	1p33
232968_at	0.99	0.92	P,M	67.90	0.36	0.01	A	24.57	FANK1	10q26.2
202093_s_at	0.98	0.90	P	405.13	0.37	0.01	P	145.90	PD2	19q13.1
1564190_x_at	1.00	0.97	P	112.13	0.37	0.02	A	41.03	ZNF519	18p11.21
227179_at	1.00	0.99	P	144.10	0.37	0.02	P,M	52.30	STAU2	8q13-q21.1
212609_s_at	1.00	0.96	P	251.17	0.37	0.00	P	90.40	SDCCAG8	1q43-q44
222028_at	1.00	0.99	P	332.13	0.37	0.00	P	119.70	ZNF45	19q13.2
213927_at	1.00	1.00	P	341.70	0.37	0.00	P	123.23		
238593_at	1.00	0.98	P	153.60	0.37	0.00	P	55.40	FLJ22531	11q13.1
39650_s_at	1.00	0.97	P	101.93	0.37	0.01	P	36.90	FLJ11383	1q42.2
1557224_at	1.00	0.96	P	123.40	0.37	0.02	P	44.93		
226793_at	0.99	0.93	P,A	580.30	0.37	0.01	A	210.77	LOC283267	11p13
242028_at	0.99	0.94	P	72.93	0.37	0.02	P,A	26.87	FLJ38281	19p13.2
231055_at	1.00	0.98	P	94.93	0.37	0.01	P	34.77		
212197_x_at	1.00	0.95	P	1106.33	0.37	0.00	P	399.27	M-RIP	17p11.2
1558529_s_at	1.00	0.95	P	143.00	0.37	0.00	P,A	51.70		8p23.3
202883_s_at	1.00	1.00	P	644.67	0.37	0.00	P	234.10	PPP2R1B	11q23.2
205248_at	1.00	0.98	P	246.97	0.37	0.01	P	90.13	C21orf5	21q22.2
226696_at	1.00	0.97	P	471.70	0.37	0.00	P	170.40	RBBP9	20p11.2
1438_at	0.99	0.92	P	200.87	0.37	0.00	A	73.13	EPHB3	3q21-qter
226506_at	1.00	0.99	P	160.63	0.37	0.00	A	58.57	FLJ13710	15q22.33
208783_s_at	1.00	0.96	P	1524.33	0.37	0.00	P	553.13	MCP	1q32
1556136_at	0.99	0.93	P,M,A	71.20	0.37	0.02	A	26.70	LOC340156	6p25.2
221992_at	1.00	0.97	P	174.37	0.37	0.00	P	63.13	KIAA0220	16p12.3
1568623_a_at	1.00	0.98	P	118.47	0.37	0.01	P	43.10	SLC35E4	22q12.2
1555461_at	1.00	0.99	P	94.00	0.37	0.01	P,A	34.23		
219854_at	1.00	0.96	P	97.60	0.37	0.01	M,A	35.47	ZNF14	19p13.3-p13.2
240314_at	0.98	0.90	P	93.90	0.37	0.03	P,A	34.87		
216179_x_at	0.98	0.90	P	48.40	0.37	0.00	A	17.73		
1555148_a_at	0.97	0.88	P,A	43.67	0.37	0.02	A	16.40	LOC130951	2p13.1
201757_at	1.00	0.99	P	5784.67	0.37	0.00	P	2104.53	NDUFS5	1p34.2-p33
42361_g_at	0.99	0.93	P	350.57	0.37	0.01	P	127.30	C6orf18	6p21.3
237159_x_at	1.00	0.96	P	118.03	0.37	0.01	P	43.03		
219453_at	1.00	0.99	P	155.23	0.37	0.00	P,A	56.60	C16orf44	16q24.1
210664_s_at	0.99	0.92	P	221.77	0.37	0.00	P	81.23	TFPI	2q31-q32.1
203739_at	1.00	0.96	P	804.37	0.37	0.00	P	294.00	ZNF217	20q13.2
201854_s_at	0.99	0.95	P	971.77	0.37	0.00	P	354.03	KIAA0431	16q23.2
1553612_at	0.99	0.94	P	94.97	0.37	0.01	P	34.87	ZNF354B	5q35.3
236665_at	0.99	0.92	P	103.93	0.37	0.02	P	38.43	NY-SAR-41	1p22.1
229499_at	0.98	0.90	P	187.07	0.37	0.02	P,M,A	69.17	CAPN13	2p22-p21
1565804_at	1.00	0.97	P	79.53	0.37	0.01	A	29.23		
200633_at	1.00	0.99	P	5669.90	0.37	0.00	P	2071.97	UBB	17p12-p11.2
224763_at	0.99	0.93	P	275.73	0.37	0.02	P	102.00		
226195_at	0.99	0.92	P,A	156.10	0.37	0.00	A	57.00	MGC16028	14q24.3
236172_at	1.00	0.96	P,A	91.80	0.37	0.01	A	33.73	LTB4R	14q11.2-q12
215314_at	0.99	0.93	P	75.70	0.37	0.02	P,A	28.27	ANK3	10q21
227508_at	0.99	0.92	P	166.23	0.37	0.00	P,A	61.13		
1553227_s_at	1.00	0.95	P	115.93	0.37	0.01	P	42.87	C21orf107	21q22.2
203266_s_at	1.00	0.96	P	246.33	0.37	0.00	P	90.47	MAP2K4	17p11.2
225177_at	1.00	1.00	P	556.17	0.37	0.00	P	204.17	RCP	8p11.22
239797_at	0.99	0.92	P	63.20	0.37	0.00	A	23.27		
213135_at	1.00	0.95	P	705.93	0.37	0.00	P	259.70	TIAM1	21q22.1
214798_at	1.00	0.96	P	55.37	0.37	0.01	P	20.53	KIAA0703	16q24.1
244026_at	1.00	0.97	P	91.80	0.37	0.01	P,A	33.80		
1569257_at	1.00	0.97	P,A	11.27	0.37	0.00	A	4.13	FMNL1	17q21
227946_at	1.00	0.96	P,A	159.70	0.37	0.00	A	58.80	OSBPL7	17q21
1554824_at	1.00	0.98	P,A	31.70	0.37	0.02	A	11.77	ZNF585A	19q13.13
212847_at	0.99	0.94	P	459.77	0.37	0.00	P	169.73	NEXN	1p31.1
225666_at	1.00	1.00	P	438.23	0.37	0.00	P	161.07	FLJ14624	13q32.3
214440_at	0.99	0.95	P	415.43	0.37	0.00	P	153.50	NAT1	8p23.1-p21.3
200752_s_at	0.98	0.91	P	703.37	0.37	0.01	P	262.50	CAPN1	11q13
201816_s_at	0.99	0.93	P	2763.57	0.37	0.00	P	1021.57	GBAS	7p12
223391_at	1.00	0.95	P	586.47	0.37	0.01	P	217.33	SGPP1	14q23.2
225989_at	1.00	0.98	P	412.80	0.37	0.01	P	152.67	HERC4	10q22.1
236267_at	1.00	0.95	P	153.20	0.38	0.00	P	56.37	ZNF346	5q35.3
202012_s_at	1.00	0.96	P	1039.57	0.38	0.00	P	385.07	EXT2	11p12-p11
37831_at	0.98	0.91	P	364.67	0.38	0.01	P,A	134.57	SIPA1L3	19q13.13
218522_s_at	1.00	0.95	P	360.43	0.38	0.00	P	133.40	BPY2IP1	19p13.12
226951_at	1.00	0.95	P	206.83	0.38	0.00	P	76.57	MGC5509	2q12.2
227191_at	0.99	0.95	P	117.57	0.38	0.00	P	43.57	CDA08	16q11.2
232860_x_at	1.00	0.96	P	155.13	0.38	0.01	P	57.70	FLJ11016	xq22.1-24
215773_x_at	1.00	0.98	P	1250.77	0.38	0.00	P	463.87	ADPRTL2	14q11.2-q12
232331_at	0.99	0.94	P	130.63	0.38	0.00	M	48.33		
203104_at	1.00	0.96	P,M,A	84.63	0.38	0.01	A	31.60	CSF1R	5q33-q35
206510_at	0.99	0.93	P	85.40	0.38	0.01	A	31.77	SIX2	2p16-p15
227911_at	0.99	0.93	P	149.13	0.38	0.01	A	55.73	FLJ10312	18p11.23
204517_at	1.00	0.99	P	1526.57	0.38	0.00	P	566.37	PPIC	5q23.2
46323_at	1.00	0.98	P	1308.50	0.38	0.00	P	484.53	ENTPD8	17q25.3
204005_s_at	0.99	0.93	P	255.33	0.38	0.00	P	95.40	PAWR	12q21
218923_at	1.00	0.96	P	232.57	0.38	0.00	P	86.30	CTBS	1p22
204798_at	0.99	0.95	P	526.37	0.38	0.01	P	197.33	MYB	6q22-q23
201548_s_at	1.00	0.98	P	292.73	0.38	0.00	P	108.83	JARID1B	1q32.1
235033_at	1.00	0.95	P	61.03	0.38	0.02	P,M	23.27	NPEPL1	20q13.32
221989_at	1.00	0.95	P	880.63	0.38	0.00	P	328.10	RPL10	xq28
222835_at	0.99	0.95	P	155.87	0.38	0.01	P	58.93	FLJ13710	15q22.33
1559057_at	0.98	0.89	P	46.13	0.38	0.03	A	17.43	FLJ23018	Xq23

224974_at	1.00	0.96	P	392.50	0.38	0.02	P	147.17	SDS3	12q24.23
210461_s_at	0.98	0.91	P	141.77	0.38	0.02	P	54.30	ABLIM1	10q25
1556678_a_at	0.99	0.92	P	35.73	0.38	0.02	A	13.40	LOC284371	19q13.41
204745_x_at	0.99	0.94	P	1098.13	0.38	0.00	P	410.20	MT1G	16q13
213258_at	1.00	0.97	P	248.27	0.38	0.01	P	93.13	TFPI	2q31-q32.1
225606_at	1.00	0.98	P	242.30	0.38	0.00	A	90.63	BCL2L11	2q13
215397_x_at	0.99	0.92	P	142.20	0.38	0.02	P,A	54.03		
211529_x_at	0.98	0.91	P	1195.27	0.38	0.00	P	448.13	HLA-G	6p21.3
204991_s_at	1.00	0.98	P	95.27	0.38	0.01	P	35.87	NF2	22q12.2
222787_s_at	0.99	0.93	P	586.10	0.38	0.01	P	221.10	FLJ11273	7p21.3
204326_x_at	1.00	0.99	P	2695.57	0.38	0.01	P	1009.97	MT1X	16q13
1557432_at	0.99	0.93	P	70.40	0.38	0.01	A	26.33	RASAL2	1q24
238613_at	0.99	0.92	P	329.87	0.38	0.01	A	123.87	ZAK	2q24.2
235848_x_at	0.99	0.93	P	292.63	0.38	0.00	P	109.63		
212079_s_at	1.00	0.95	P	163.40	0.38	0.02	P	62.70	MLL	11q23
216215_s_at	0.99	0.92	P	901.10	0.38	0.00	P	336.53	RBM9	22q13.1
222751_at	1.00	0.96	P	580.70	0.38	0.01	P	218.60	FLJ22313	7p14.2
238686_at	0.98	0.91	P	293.97	0.38	0.00	P	110.83	FBXO3	11p13
226493_at	0.99	0.94	P	583.80	0.38	0.00	P	219.57	FLJ31322	2q33.2
219297_at	0.99	0.94	P	424.23	0.38	0.01	P	160.13	DKFZp686L20145	xq24
209989_at	0.99	0.92	P	75.13	0.38	0.01	P,A	28.40	ZNF268	12q24.33
226181_at	0.99	0.94	P	495.63	0.38	0.00	P	186.60	TUBE1	6q21
230179_at	1.00	0.97	P	682.73	0.38	0.00	P	256.63	LOC285812	6p23
202414_at	1.00	0.98	P	331.00	0.38	0.00	P	124.20	ERCC5	13q22
227697_at	0.99	0.94	P,A	18.97	0.38	0.01	A	7.20	SOCS3	17q25.3
235305_s_at	1.00	0.96	P	82.43	0.38	0.00	A	31.00	FLJ10948	1p32.3
220235_s_at	0.99	0.92	P	316.07	0.38	0.00	P	119.23	RIF1	1p13.2
1555105_a_at	1.00	0.98	P	137.30	0.38	0.00	P	51.53	MI-ER1	1p31.3
204067_at	0.99	0.94	P,A	224.57	0.38	0.03	A	85.93	SUOX	12q13.13
212285_s_at	1.00	0.98	P	1300.10	0.38	0.01	P	489.07	AGRN	1p36.33
1556344_at	0.99	0.94	P	122.30	0.38	0.00	A	46.47		21q22.11
212554_at	0.99	0.93	P	719.43	0.38	0.00	P	272.37	CAP2	6p22.3
229615_at	0.99	0.94	P,A	163.83	0.38	0.03	P	62.70		
241815_at	0.99	0.92	P	38.17	0.38	0.03	P	14.73		
223276_at	1.00	0.97	P	297.63	0.38	0.00	P	112.77	NID67	5q33.1
225675_at	0.99	0.93	P	132.60	0.38	0.03	P	51.20	C14orf101	14q22.3
201579_at	1.00	0.97	P	1084.33	0.38	0.01	P	410.63	FAT	4q34-q35
210389_x_at	1.00	0.98	P	248.30	0.38	0.00	P	94.13	TUBD1	17q23.2
212131_at	1.00	0.96	P	1027.33	0.38	0.00	P	388.47	C19orf13	19q13.12
226316_at	0.99	0.93	P	194.33	0.38	0.03	P	74.83	C13orf10	13q22.2
226705_at	0.99	0.94	P	228.17	0.38	0.02	A	87.83	FGFR1	8p11.2-p11.1
210070_s_at	0.99	0.95	P	186.90	0.38	0.00	P	70.50	CPT1B	22q13.33
202487_s_at	0.99	0.93	P	2994.43	0.38	0.00	P	1135.57	H2AFV	7p13
1563881_at	0.98	0.89	P	23.43	0.38	0.03	A	9.13		
207733_x_at	1.00	0.98	P	174.13	0.38	0.01	A	67.00	PSG9	19q13.2
1555372_at	1.00	0.97	P	245.87	0.38	0.00	P,M	93.33	BCL2L11	2q13
224492_s_at	0.99	0.93	P	429.50	0.38	0.01	P	165.27	ZNF627	19p13.2
202771_at	1.00	0.97	P	1195.73	0.38	0.00	P	454.70	FAM38A	16q24.3
204765_at	1.00	0.97	P	308.47	0.38	0.02	A	118.83	ARHGEF5	7q33-q35
227279_at	1.00	0.96	P	1288.90	0.39	0.01	P	489.47	MGC15737	xq22.2
221059_s_at	1.00	1.00	P	2278.67	0.39	0.00	P	863.63	CHST6	16q22
217995_at	0.99	0.95	P	898.97	0.39	0.01	P	342.17	SQRDL	15q15
40569_at	1.00	0.98	P	329.50	0.39	0.00	P	124.80	ZNF42	19q13.2-q13.4
201417_at	0.99	0.94	P	1096.63	0.39	0.00	P	417.00	SOX4	6p22.3
223605_at	0.99	0.92	P	80.33	0.39	0.03	A	31.33	SLC25A18	22q11.2
231828_at	1.00	0.98	P	212.47	0.39	0.01	P	81.50		
218798_at	0.98	0.91	P	301.97	0.39	0.01	P	115.23	FLJ12949	19p13.2
206860_s_at	1.00	0.97	P	536.80	0.39	0.01	P	205.17	FLJ20323	7p22-p21
219185_at	1.00	0.96	P	251.70	0.39	0.01	P	96.20	SIRT5	6p23
218072_at	0.98	0.90	P	784.77	0.39	0.00	P	296.47	COMMD9	11p13
219209_at	0.98	0.91	P	132.57	0.39	0.02	P	51.70	MDA5	2p24.3-q24.3
204295_at	0.99	0.92	P	750.27	0.39	0.01	P,M,A	286.40	SURF1	9q34.2
232373_at	1.00	0.96	P,A	47.07	0.39	0.01	A	18.00	NOXA1	
230933_at	1.00	0.97	P	76.13	0.39	0.01	P,A	29.43	DSTN	20p11.23
229119_s_at	1.00	0.96	P	819.40	0.39	0.00	P	312.47	ACTB	7p15-p12
212729_at	0.99	0.93	P	195.00	0.39	0.00	P	74.67	DLG3	xq13.1
202602_s_at	1.00	0.99	P	3092.67	0.39	0.00	P	1178.93	HTATSF1	xq26.1-q27.2
221568_s_at	1.00	0.98	P	1846.63	0.39	0.00	P	705.33	LIN7C	11p14
221230_s_at	1.00	0.97	P	576.57	0.39	0.00	P	220.13	ARID4B	1q42.1-q43
235245_at	0.99	0.92	P,A	112.03	0.39	0.03	A	44.03	FLJ33318	17q21.33
209704_at	0.99	0.92	P	267.57	0.39	0.01	P	103.00	M96	1p22.1
235110_at	1.00	0.98	P	196.03	0.39	0.00	P	75.17	HRASLS3	11q13.1
229099_at	0.99	0.93	P	416.13	0.39	0.03	P	161.27		
244699_at	0.98	0.89	P	136.27	0.39	0.03	A	52.63	AHI1	6q23.2
226246_at	0.98	0.91	P	465.03	0.39	0.00	A	178.83	KCTD1	18q12.1
1553175_s_at	1.00	0.98	P,A	48.50	0.39	0.00	A	18.57	PDE5A	4q25-q27
234974_at	0.99	0.92	P,A	82.33	0.39	0.01	A	31.90	GALM	2p22.3
213034_at	1.00	0.98	P	379.23	0.39	0.00	P	145.43	KIAA0999	11q23.3
227319_at	1.00	0.98	P	713.27	0.39	0.00	P	273.50	C16orf44	16q24.1
226499_at	0.99	0.94	P	121.60	0.39	0.00	A	46.57	TUBB2	
209706_at	1.00	0.96	P	179.60	0.39	0.02	P	69.87	NKX3-1	8p21
218486_at	0.99	0.94	P	429.43	0.39	0.00	P	165.50	TIEG2	2p25
44563_at	0.99	0.92	P	667.60	0.39	0.00	P	255.37	FLJ10385	17p13.2
240231_at	0.99	0.93	P	116.63	0.39	0.01	P	45.30		
1565868_at	0.98	0.89	P	246.50	0.39	0.01	P	95.37		
218532_s_at	1.00	0.99	P	50.27	0.39	0.01	P,A	19.47	FLJ20152	5p15.1
209822_s_at	0.99	0.94	P	157.73	0.39	0.00	P	61.00	VLDLR	9p24
241397_at	0.99	0.94	P	70.80	0.39	0.03	A	27.97	EHF	11p12
217596_at	0.98	0.90	P,A	87.57	0.39	0.02	A	34.27	UPF3A	13q34
226669_at	0.99	0.95	P	278.73	0.39	0.00	P	107.03	USP42	7p22.2
204545_at	0.99	0.93	P	192.40	0.39	0.02	P,A	74.90	PEX6	6p21.1
235071_at	1.00	0.99	P	294.00	0.39	0.01	P	114.17	LOC116143	2p13.3
202736_s_at	0.99	0.93	P	1231.90	0.39	0.00	P	476.90	LSM4	19p13.11
242920_at	0.99	0.94	P	125.97	0.39	0.02	P	49.10		
219928_s_at	0.98	0.91	P	145.10	0.39	0.01	P,A	56.03	CABYR	18q11.2
227840_at	1.00	0.96	P	176.10	0.39	0.01	P	68.30	LOC130355	2q14.2

1565863_at	0.99	0.91	P	49.57	0.39	0.01	P,A	19.23		
221626_at	0.98	0.90	P	105.93	0.39	0.01	P	41.27	ZNF506	19p13.11
241853_at	0.98	0.91	P	15.50	0.39	0.00	P,A	6.00		
228932_at	0.99	0.94	P	180.30	0.39	0.02	P	70.63		
225235_at	1.00	0.99	P	1072.83	0.39	0.01	P	416.00	FBXO23	5q35.3
1554743_x_at	0.98	0.90	P,A	59.60	0.39	0.01	A	23.23	PMS1	2q31.1
228347_at	0.99	0.92	P	178.27	0.39	0.00	P	69.27	SIX1	14q23.1
226259_at	0.99	0.91	P	228.77	0.39	0.00	P	88.47	SEC15L1	10q23.33
233078_at	0.99	0.91	P	336.17	0.39	0.01	P	130.83		
207865_s_at	0.99	0.94	P,M	26.93	0.39	0.01	A	10.47	BMP8B	1p35-p32
224991_at	1.00	0.95	P	520.20	0.39	0.01	P,M,A	203.03	CMIP	16q23
203114_at	1.00	0.96	P	540.40	0.39	0.02	P	211.43	SSSCA1	11q13.1
1554890_a_at	0.99	0.94	P	194.33	0.39	0.02	P	76.40	TIA1	2p13
219503_s_at	1.00	0.98	P	69.20	0.39	0.00	P,A	26.97	FLJ11036	3p25.2
214355_x_at	1.00	0.99	P	512.43	0.39	0.00	A	198.60	MGEA6	14q13.3
202932_at	0.99	0.94	P	2062.87	0.39	0.00	P	803.20	YES1	18p11.31-p11.21
228297_at	1.00	0.96	P	2167.30	0.39	0.00	P	842.90	CNN3	1p22-p21
201933_at	1.00	0.98	P	817.90	0.39	0.01	P	318.57	PCOLN3	16q24.3
218395_at	1.00	0.99	P	829.80	0.39	0.00	P	322.70	ACTR6	12q23.3
201549_x_at	1.00	0.98	P	424.63	0.39	0.00	P	165.77	JARID1B	1q32.1
35156_at	0.99	0.94	P	724.53	0.40	0.00	P	280.80	LOC203069	8p21.2
224715_at	1.00	0.96	P	1568.97	0.40	0.00	P	608.77	WDR34	9q34.13
202859_x_at	0.99	0.93	P	156.70	0.40	0.00	P,A	61.13	IL8	4q13-q21
215313_x_at	1.00	0.99	P	4370.70	0.40	0.00	P	1702.17	HLA-A	6p21.3
226085_at	0.99	0.94	P	1420.20	0.40	0.00	P	554.67		
225144_at	0.99	0.94	P	541.33	0.40	0.00	P	211.33	BMPR2	2q33-q34
229582_at	1.00	0.96	P	651.13	0.40	0.00	P	254.13	LOC125476	18q12.2
201275_at	1.00	0.98	P	2312.53	0.40	0.01	P	903.57	FDPS	1q22
220092_s_at	0.98	0.91	P,A	52.83	0.40	0.01	A	20.73	ANTXR1	2p13.1
242488_at	1.00	0.97	P	107.67	0.40	0.00	P	42.07		
1558345_a_at	0.99	0.92	P	199.90	0.40	0.00	M,A	77.70	LOC283031	10q11.21
1553768_a_at	0.99	0.94	P	168.63	0.40	0.00	P,A	65.97	DCBLD1	6q22.31
202933_s_at	0.99	0.94	P	2843.87	0.40	0.02	P	1128.03	YES1	18p11.31-p11.21
213526_s_at	1.00	0.95	P	187.10	0.40	0.01	P	73.10	F25965	19q13.1
218467_at	1.00	0.96	P	4131.77	0.40	0.00	P	1616.60	TNFSF5IP1	18p11.21
211979_at	1.00	0.95	P	274.17	0.40	0.02	P,A	108.43	GPR107	9q34.2
205641_s_at	1.00	0.99	P,A	248.37	0.40	0.01	A	98.03	TRADD	16q22
235659_at	0.99	0.93	P,A	171.10	0.40	0.00	A	66.93		
229436_x_at	1.00	0.99	P	716.37	0.40	0.01	P	281.50	C6.1A	Xq28
212104_s_at	0.99	0.94	P	804.97	0.40	0.01	P	317.93	RBM9	22q13.1
240155_x_at	0.98	0.89	P	103.97	0.40	0.02	P,M	41.00	LOC115648	19p13.11
229648_at	0.99	0.92	P	116.63	0.40	0.01	P	46.37	RICS	11q24-q25
203758_at	0.99	0.95	P	262.03	0.40	0.00	P	103.17	CTSO	4q31-q32
208934_s_at	1.00	0.97	P	199.50	0.40	0.00	M,A	78.43	LGALS8	1q42-q43
209275_s_at	0.99	0.92	P	655.10	0.40	0.01	P	258.03	CLN3	16p12.1
225101_s_at	1.00	0.96	P	697.37	0.40	0.00	P	274.00	SNX14	6q15
1554406_a_at	1.00	0.97	P,M,A	142.10	0.40	0.01	A	56.53	CLECSF12	12p13.2-p12.3
217144_at	1.00	0.97	P	1584.20	0.40	0.01	P	624.00	UBB	17p12-p11.2
219336_s_at	1.00	0.98	P	453.83	0.40	0.02	P	181.33	ASCC1	10pter-q25.3
235086_at	1.00	0.95	P,A	163.67	0.40	0.00	A	64.60	THBS1	15q15
243444_at	0.98	0.91	P,M,A	87.83	0.40	0.02	A	35.43		
232749_at	0.99	0.94	P	93.60	0.40	0.01	P	37.13		
238967_at	0.99	0.95	P	70.33	0.40	0.01	P,A	28.10		
219754_at	1.00	0.99	P	268.60	0.40	0.01	P,A	106.63	FLJ11016	xq22.1-24
227467_at	0.99	0.94	P	371.73	0.40	0.00	P	146.73	RDH10	8q13.3
206385_s_at	1.00	0.98	P	601.77	0.40	0.01	P	238.30	ANK3	10q21
229943_at	1.00	0.99	P	305.70	0.40	0.01	P,A	122.33	RFP2	13q14
236251_at	1.00	0.97	P	427.07	0.40	0.02	P	169.80		
41387_r_at	0.99	0.94	P	365.13	0.40	0.02	P	145.03	JMJD3	17p13.1
213946_s_at	1.00	0.98	P	93.43	0.40	0.01	P,A	37.10	KIAA0657	2q36.1
227804_at	1.00	0.99	P	224.83	0.40	0.00	P	88.77	LOC116238	17q11.2
228639_at	1.00	0.96	P	164.23	0.40	0.01	P,A	65.37	FGD1	Xp11.21
213287_s_at	1.00	0.97	P	3185.63	0.40	0.00	P	1258.60	KRT10	17q21
228559_at	1.00	0.98	P	1186.70	0.40	0.01	P	470.07		
243894_at	1.00	0.99	P	111.00	0.40	0.00	P	44.10	SLC41A2	12q24.11
221754_s_at	0.99	0.92	P	388.63	0.40	0.01	P	154.00	CORO1B	11q13.1
212368_at	1.00	0.96	P	507.73	0.40	0.01	P	201.83	ZNF292	6q15
224852_at	0.98	0.90	P	280.60	0.40	0.00	P	112.03	TTC17	11p11.2
212641_at	1.00	0.96	P	123.93	0.40	0.02	P,A	49.80	HIVEP2	6q23-q24
235099_at	0.99	0.94	P	405.43	0.40	0.01	P	161.10	CKLFSF8	3p22.3
218217_at	0.99	0.92	P	396.60	0.40	0.00	P	158.23	SCPEP1	17q23.2
228919_at	1.00	0.96	P	252.50	0.40	0.00	P	100.43		
210651_s_at	0.99	0.93	P	126.33	0.40	0.00	A	50.20	EPHB2	1p36.1-p35
210045_at	1.00	0.99	P,M	216.43	0.40	0.01	A	86.43	IDH2	15q26.1
225289_at	0.99	0.94	P	560.47	0.40	0.00	P	224.17	STAT3	17q21.31
1553719_s_at	1.00	0.97	P	81.33	0.41	0.00	P	32.47	ZNF548	19q13.43
202083_s_at	1.00	0.98	P	127.03	0.41	0.01	P	50.63	SEC14L1	17q25.1-17q25.2
235191_at	0.99	0.92	P	220.73	0.41	0.02	P	89.43	LOC148189	19q12
205871_at	0.98	0.91	P	53.93	0.41	0.01	P	21.73	PLGL	2p11-q11
215068_s_at	1.00	0.98	P	226.50	0.41	0.00	P	90.50	FLJ11467	7p22.2
221768_at	0.99	0.94	P	673.00	0.41	0.01	P	269.93	SFPQ	1p34.3
201409_s_at	0.98	0.91	P	828.93	0.41	0.01	P	333.10	PPP1CB	2p23
213116_at	0.98	0.90	P	124.00	0.41	0.01	P,A	49.50	NEK3	13q14.13
212321_at	1.00	0.97	P	451.23	0.41	0.00	P	180.00	SGPL1	10q21
208614_s_at	0.98	0.91	P	229.27	0.41	0.01	M,A	92.20	FLNB	3p14.3
217896_s_at	1.00	0.96	P	313.10	0.41	0.01	A	126.00	NIP30	16q13
205546_s_at	1.00	0.97	P	1186.97	0.41	0.00	P	473.17	TYK2	19p13.2
212815_at	0.99	0.92	P	1011.77	0.41	0.00	P	405.80	HELIC1	6q16
227036_at	0.99	0.94	P	274.63	0.41	0.00	P	110.40		
218192_at	0.97	0.88	P	254.17	0.41	0.01	P	102.43	IHPK2	3p21.31
203722_at	1.00	0.98	P	201.47	0.41	0.01	M,A	80.83	ALDH4A1	1p36
202511_s_at	1.00	0.96	P	1200.23	0.41	0.00	P	482.03	APG5L	6q21
233770_at	1.00	0.97	P	73.53	0.41	0.00	P	29.50		
215380_s_at	1.00	1.00	P	3972.20	0.41	0.00	P	1589.63	C7orf24	7p15-p14
209916_at	1.00	0.96	P	259.03	0.41	0.00	P	103.67	DHTKD1	10p14
219543_at	1.00	0.97	P	113.23	0.41	0.00	P	45.43	MAWBP	10pter-q25.3

225105_at	0.99	0.95	P	217.27	0.41	0.00	P	87.40	LOC387882	12q24.11
235467_s_at	1.00	0.96	P	110.83	0.41	0.01	P	44.57	KCNC4	1p21
228642_at	1.00	0.97	P	419.20	0.41	0.00	P	168.47		
223093_at	0.99	0.93	P	124.03	0.41	0.00	A	49.90	ANKH	5p15.1
235015_at	0.98	0.90	P,A	150.20	0.41	0.01	A	60.23	ZDHHC9	9
209790_s_at	0.99	0.94	P	358.33	0.41	0.01	P	144.47	CASP6	4q25
209971_x_at	1.00	0.98	P	2411.90	0.41	0.00	P	969.03	JTV1	7p22
222221_x_at	0.99	0.95	P	421.67	0.41	0.01	P	169.60	EHD1	11q13
205322_s_at	0.99	0.94	P	233.73	0.41	0.00	A	93.87	MTF1	1p33
1554627_a_at	0.99	0.93	P	660.53	0.41	0.01	P	267.97	ASCC1	10pter-q25.3
201948_at	1.00	0.97	P	1252.87	0.41	0.00	P	504.50	HUMAUANTIG	1p34.2
209733_at	0.99	0.94	P	91.17	0.41	0.00	P,A	36.77	LOC286440	xq22.3
202880_s_at	1.00	0.97	P	234.90	0.41	0.00	P	94.40	PSCD1	17q25
230815_at	1.00	0.97	P	66.53	0.41	0.01	P,A	27.00		9q22.1
201867_s_at	0.99	0.94	P	134.13	0.41	0.00	A	54.13	TBL1X	xp22.3
223152_at	0.99	0.94	P	161.00	0.41	0.00	P	65.10	PPP1R12C	19q13.42
213852_at	1.00	0.96	P	904.67	0.41	0.01	P	366.20	RBM8A	1q12
204404_at	1.00	0.98	P	1186.20	0.41	0.00	P	479.27	SLC12A2	5q23.3
242056_at	1.00	0.98	P	226.20	0.41	0.00	P	91.20	TRIM45	1p12
202981_x_at	1.00	0.99	P	826.43	0.41	0.00	P	333.37	SIAH1	16q12
36019_at	1.00	0.99	P	611.80	0.41	0.00	P	246.97	STK19	6p21.3
202326_at	0.99	0.95	P	261.63	0.41	0.01	P	106.80	BAT8	6p21.31
219596_at	1.00	0.99	P	284.30	0.41	0.01	P	115.40	THAP10	15q22.32
224366_s_at	1.00	0.96	P	621.30	0.41	0.00	P	251.83	REPS1	6q23.1-q24.1
240478_at	0.99	0.92	P	269.20	0.41	0.00	P	108.43		
206088_at	0.99	0.93	P	92.80	0.41	0.01	A	37.87	KIAA0563	17q21.32
238504_at	1.00	0.95	P	251.43	0.41	0.01	P	103.37	C6orf57	6q13
216092_s_at	1.00	0.96	P	126.93	0.41	0.00	P	51.27	SLC7A8	14q11.2
217784_at	1.00	0.99	P	630.43	0.41	0.02	P	258.37	YKT6	7p15.1
207020_at	0.99	0.95	P,M	127.60	0.41	0.01	A	52.17	HSF2BP	21q22.3
242423_x_at	0.99	0.94	P	159.43	0.41	0.01	P	65.13		
1569335_a_at	1.00	0.98	P	77.47	0.41	0.01	P,A	31.73	FLJ12541	15q22.33
204285_s_at	0.99	0.95	P	481.77	0.41	0.01	P	195.90	PMAIP1	18q21.32
223530_at	0.99	0.93	P	229.27	0.41	0.03	P	94.57	TDRKH	1q21
212848_s_at	1.00	0.97	P	239.57	0.41	0.00	A	97.17	C9orf3	9q22.33
243470_at	0.99	0.92	P	38.57	0.41	0.02	P,A	15.83		
224156_x_at	1.00	0.97	P,M	145.53	0.41	0.02	A	59.87	IL17RB	3p21.1
222688_at	1.00	0.99	P	721.00	0.41	0.01	P	294.53	PHCA	11q13.3
212184_s_at	1.00	0.97	P	335.80	0.41	0.01	P	137.03	MAP3K7IP2	6q25.1-q25.3
204333_s_at	1.00	0.99	P	223.33	0.41	0.00	P	90.73	AGA	4q32-q33
215696_s_at	1.00	0.98	P	1116.87	0.41	0.00	P	453.23	KIAA0310	9q34.3
1557433_at	1.00	0.98	P	146.47	0.41	0.00	P	59.50		
209575_at	1.00	0.97	P	523.67	0.41	0.01	P	212.63	IL10RB	21q22.11
210128_s_at	0.99	0.95	P,M,A	95.67	0.41	0.03	A	39.77	LTB4R	14q11.2-q12
218795_at	0.99	0.95	P	727.87	0.41	0.01	P	295.43	ACP6	1q21
225234_at	1.00	0.98	P	622.10	0.41	0.00	P	253.57	CBL	11q23.3
235119_at	0.99	0.94	P,M	77.47	0.41	0.01	A	31.47	TAF3	10p15.1
207700_s_at	0.98	0.91	P	390.47	0.41	0.01	P	160.53	NCOA3	20q12
202870_s_at	1.00	0.99	P	1667.47	0.41	0.00	P	678.00	CDC20	1p34.1
1556414_at	0.99	0.94	P	34.33	0.41	0.00	A	14.10		
1559249_at	0.99	0.93	P	132.73	0.41	0.01	P	54.57	SCA1	6p23
226702_at	1.00	0.98	P	105.97	0.41	0.01	P,A	43.27	LOC129607	2p25.2
212014_x_at	0.99	0.93	P	3124.70	0.41	0.00	P	1280.47	CD44	11p13
226726_at	0.98	0.91	P	529.83	0.41	0.00	P	217.07	LOC129642	2p25.2
213671_s_at	1.00	0.99	P	1516.47	0.41	0.01	P	618.77	MARS	12q13.2
225102_at	1.00	1.00	P	874.00	0.41	0.00	P	356.17	MGLL	3q21.3
229700_at	0.98	0.91	P	296.73	0.41	0.00	P	121.53	LOC148203	19p13.11
229551_x_at	1.00	0.97	P	1215.73	0.41	0.00	P	496.13	ZNF367	9q22
211555_s_at	0.99	0.93	P	98.97	0.41	0.01	A	40.63	GUCY1B3	4q31.3-q33
204565_at	1.00	0.96	P	1159.97	0.41	0.00	P	474.03	THEM2	6p22.1
218067_s_at	1.00	0.99	P	1717.20	0.41	0.01	P	702.33	FLJ10154	13q33.2
204238_s_at	0.99	0.93	P	424.93	0.42	0.03	P,A	176.80	C6orf108	6p21.1
229983_at	1.00	0.95	P	308.53	0.42	0.01	P	126.57	TIGD2	4q22.1
218927_s_at	1.00	0.96	P	235.80	0.42	0.01	P,M,A	96.83	CHST12	7p22
212032_s_at	1.00	0.98	P	950.40	0.42	0.01	P	390.17	PTOV1	19q13.33
209908_s_at	0.98	0.91	P	98.03	0.42	0.00	P,M	40.27	TGFB2	1q41
243543_at	0.99	0.93	P	80.47	0.42	0.03	P	33.43		
219751_at	1.00	0.98	P	407.07	0.42	0.02	P	167.57	FLJ21148	16q13
244561_at	1.00	0.96	P,A	122.90	0.42	0.00	A	50.23		
233506_at	0.99	0.95	P,M,A	136.37	0.42	0.01	A	55.87		
204752_x_at	1.00	0.99	P	1246.77	0.42	0.00	P	512.17	ADPRTL2	14q11.2-q12
226046_at	1.00	0.97	P	253.60	0.42	0.00	P	103.97	MAPK8	10q11.23
47560_at	1.00	0.95	P	729.37	0.42	0.00	P	298.03	LPHN1	19p13.2
226756_at	1.00	0.96	P	274.00	0.42	0.00	P	112.73	FLJ36031	7q22.2
221208_s_at	0.99	0.95	P	366.57	0.42	0.01	P	151.00	FLJ23342	11q24.2
225344_at	0.99	0.95	P	1763.03	0.42	0.00	P	724.10	NCOA7	6q22.33
219689_at	1.00	0.95	P,A	90.50	0.42	0.02	A	37.53	LOC56920	3p21.31
223097_at	1.00	0.96	P	929.73	0.42	0.00	P	380.63	ADPRHL2	1p34.3
238601_at	0.99	0.94	P	173.93	0.42	0.00	P	71.20	PHKB	16q12-q13
227605_at	1.00	0.97	P	228.87	0.42	0.00	P	94.07	SCYE1	4q25
213785_at	0.99	0.92	P,M	161.40	0.42	0.03	A	66.93	IPO9	1q32.1
236976_at	0.98	0.90	P,M	155.70	0.42	0.01	A	63.83	FANCA	16q24.3
1563497_at	0.99	0.94	P	67.13	0.42	0.01	P,A	27.93	USP25	21q11.2
200685_at	1.00	0.99	P	335.97	0.42	0.02	P	139.20	SFRS11	1p31
213518_at	0.99	0.92	P	757.60	0.42	0.00	P	312.70	PRKCI	3q26.3
225487_at	0.98	0.90	P	114.00	0.42	0.01	P	47.17	TMEM18	2p25.3
205263_at	1.00	0.97	P	836.80	0.42	0.00	P	344.50	BCL10	1p22
1557513_a_at	1.00	0.96	P	33.23	0.42	0.01	M,A	13.80		
238482_at	0.99	0.92	P	92.10	0.42	0.00	P	37.97	KLF7	2q32
223655_at	0.99	0.94	P	288.30	0.42	0.00	P	119.63	M160	12p13.31
237043_at	0.99	0.92	P	247.23	0.42	0.02	A	103.20		
238761_at	0.98	0.90	P	420.90	0.42	0.00	P	174.07		
221211_s_at	1.00	0.96	P,A	25.43	0.42	0.02	A	10.67	C21orf7	21q22.3
240869_at	0.99	0.93	P	127.87	0.42	0.00	P	52.87		
218712_at	0.99	0.93	P	769.27	0.42	0.01	P	319.07	FLJ20508	1p34.2
204365_s_at	1.00	0.95	P	167.90	0.42	0.00	A	69.03	C2orf23	2p11.2

232489_at	1.00	0.98	P	132.83	0.42	0.01	P	54.97	FLJ10287	1pter-q31.3
233952_s_at	0.99	0.92	P	620.87	0.42	0.01	P	257.90	ZNF295	21q22.3
207023_x_at	0.99	0.92	P	2815.17	0.42	0.00	P	1154.73	KRT10	17q21
219165_at	0.99	0.94	P	194.43	0.42	0.01	P	80.73	PDLIM2	8p21.2
218379_at	0.99	0.95	P	738.77	0.42	0.00	P	305.50	RBM7	11q23.1-q23.2
219286_s_at	1.00	0.95	P	768.53	0.42	0.01	P	317.80	RBM15	1p13
229312_s_at	0.99	0.93	P	102.37	0.42	0.01	P,M	42.60	GKAP1	9q22.1
204063_s_at	0.99	0.94	P,M	59.50	0.42	0.03	A	25.23	ULK2	17p11.2
235381_at	1.00	0.99	P	467.80	0.42	0.01	P	193.53	HBXAP	11q13.4
230421_at	1.00	0.96	P	118.83	0.42	0.02	P,A	50.03		5q35.3
202417_at	1.00	0.97	P	1660.53	0.42	0.00	P	685.27	KEAP1	19p13.2
209098_s_at	0.98	0.90	P	87.30	0.42	0.01	P	36.53	JAG1	20p12.1-p11.23
1555977_at	0.99	0.95	P	203.33	0.42	0.01	P	84.70	MRCL3	18p11.31
225112_at	1.00	0.99	P	873.60	0.42	0.01	P	362.63	ABI2	2q33
225446_at	0.99	0.93	P	169.63	0.42	0.01	P	70.93	C21orf107	21q22.2
1552562_at	1.00	0.96	P	72.13	0.42	0.02	A	30.23	ZNF570	19q13.13
37425_g_at	1.00	0.95	P	343.37	0.42	0.00	P	142.03	C6orf18	6p21.3
225198_at	1.00	0.95	P	611.10	0.42	0.00	P	254.97	VAPA	18p11.22
207039_at	1.00	0.96	P	618.23	0.42	0.03	P	259.50	CDKN2A	9p21
211580_s_at	1.00	0.96	P	104.57	0.42	0.00	A	43.57	PIK3R3	1p34.1
215235_at	0.99	0.95	P	879.47	0.42	0.01	P	365.80	SPTAN1	9q33-q34
231569_at	0.99	0.94	P	112.87	0.42	0.03	A	48.10	MGC39655	xq22.2
209835_x_at	0.99	0.94	P	3465.00	0.42	0.00	P	1447.17	CD44	11p13
215287_at	0.99	0.93	P	141.53	0.42	0.00	P	59.10		
220941_s_at	0.99	0.92	P	313.23	0.42	0.01	P	129.83	C21orf91	21q21.1
204203_at	1.00	0.97	P	371.37	0.42	0.00	P	154.87	CEBPG	19q13.12
222360_at	1.00	0.96	P	123.97	0.42	0.00	P	51.63	CGI-30	1p21.2
205167_s_at	0.99	0.92	P	160.83	0.42	0.03	P	68.50	CDC25C	5q31
204686_at	1.00	0.98	P	166.80	0.42	0.02	P	70.23	IRS1	2q36
203154_s_at	1.00	0.98	P,A	470.63	0.42	0.01	A	197.00	PAK4	19q13.2
200843_s_at	1.00	0.98	P	1057.90	0.42	0.01	P	441.83	EPRS	1q41-q42
218314_s_at	1.00	0.98	P	322.30	0.42	0.01	P	136.00	FLJ10726	11q23.2
207621_s_at	1.00	0.99	P	268.03	0.42	0.00	P	111.73	PEMT	17p11.2
204794_at	1.00	0.96	P,M,A	153.37	0.42	0.02	A	65.27	DUSP2	2q11
225192_at	0.99	0.94	P	503.70	0.42	0.00	P	209.40	C10orf46	10q26.13
227689_at	1.00	0.96	P	183.97	0.42	0.00	P	76.97	ZNF227	19q13.32
1566403_at	0.99	0.93	P	68.93	0.42	0.01	P,A	28.80	RNU68	19p13
201000_at	1.00	0.99	P	866.33	0.42	0.00	P,A	361.53	AARS	16q22
203818_s_at	1.00	0.97	P	790.70	0.42	0.00	P	329.60	SF3A3	1p34.2
203218_at	1.00	0.97	P	822.80	0.42	0.00	P	344.07	MAPK9	5q35
203345_s_at	1.00	0.98	P	635.20	0.42	0.00	P	265.33	M96	1p22.1
229464_at	0.99	0.94	P	62.60	0.42	0.01	P,A	26.30	MYEF2	15q15.2
218502_s_at	1.00	0.98	P	339.13	0.42	0.00	P	141.70	TRPS1	8q24.12
229134_at	1.00	0.98	P	172.93	0.42	0.02	P,A	72.87		
244354_at	1.00	0.96	P	185.93	0.42	0.01	P	78.13		
224646_x_at	1.00	0.99	P	570.43	0.42	0.01	P	239.43	H19	11p15.5
231025_at	0.98	0.89	P	67.23	0.42	0.02	M,A	28.20		
225785_at	1.00	0.99	P	672.73	0.43	0.00	P	281.27		
206261_at	1.00	0.97	P	169.70	0.43	0.02	P	72.30	ZNF239	10q11.22-q11.23
204547_at	1.00	0.99	P	306.90	0.43	0.00	P	128.73	RAB40B	17q25.3
218187_s_at	1.00	0.96	P	819.63	0.43	0.00	P	344.30	FLJ20989	8q24.3
213294_at	1.00	0.95	P	794.00	0.43	0.00	P	333.20	FLJ38348	2p22.3
226380_at	1.00	0.96	P	255.43	0.43	0.00	P	106.77	PTPN21	14q31.3
230141_at	1.00	0.97	P	72.30	0.43	0.00	P	30.30	ARID4A	14q22.3-q23.1
202737_s_at	1.00	0.98	P	2769.13	0.43	0.00	P	1160.93	LSM4	19p13.11
212482_at	0.99	0.92	P	463.97	0.43	0.01	P	196.23	FLJ13910	2p11.2
219866_at	1.00	0.98	P	99.10	0.43	0.00	A	41.60	CLIC5	6p12.1-21.1
210365_at	0.99	0.95	P	89.47	0.43	0.00	P,A	37.63	RUNX1	21q22.3
228751_at	1.00	0.96	P	195.03	0.43	0.03	P	82.73	CLK4	5q35
213421_x_at	1.00	0.97	P	139.27	0.43	0.00	A	58.47	PRSS3	9p11.2
1558896_at	0.99	0.92	P,A	135.63	0.43	0.02	M,A	57.43		
202315_s_at	1.00	0.99	P	408.20	0.43	0.01	P	171.97	BCR	22q11.23
207605_x_at	1.00	0.95	P,A	174.60	0.43	0.02	A	73.80	ZNF117	7q11.2
210046_s_at	0.99	0.95	P	980.70	0.43	0.00	P	413.73	IDH2	15q26.1
201324_at	0.99	0.93	P	1084.67	0.43	0.02	P	463.03	EMP1	12p12.3
235837_at	1.00	0.96	P	202.10	0.43	0.00	P,M,A	85.57		
203752_s_at	1.00	0.98	P	2373.27	0.43	0.00	P	999.07	JUND	19p13.2
211711_s_at	1.00	0.98	P	244.00	0.43	0.00	P	102.87	PTEN	10q23.3
203825_at	0.99	0.94	P	571.40	0.43	0.02	P	243.60	BRD3	9q34
204505_s_at	1.00	0.99	P	130.40	0.43	0.01	A	55.57	EPB49	8p21.1
1556026_at	0.99	0.92	P	27.90	0.43	0.02	P,A	11.83	IDS	Xq28
1554093_a_at	1.00	0.98	P	567.93	0.43	0.00	P	239.63	SNAPC5	15q22.2
227630_at	1.00	0.95	P	275.67	0.43	0.03	P	117.53	PPP2R5E	14q23.1
227443_at	1.00	0.97	P,A	152.70	0.43	0.01	A	65.07	C9orf150	9p22.3
1558809_s_at	0.98	0.91	P	77.67	0.43	0.02	P,A	33.50	LOC284408	19q13.13
232014_at	1.00	0.97	P	104.47	0.43	0.01	P	44.33	ZNF30	19q13.13
235660_at	1.00	0.97	P	100.27	0.43	0.00	A	42.40		
206659_at	0.98	0.90	P	69.27	0.43	0.03	A	29.93	FLJ14082	2q11.2
236525_at	1.00	0.95	P,M,A	45.63	0.43	0.02	A	19.77	FBXO36	2q37.1
226649_at	1.00	0.97	P	311.30	0.43	0.01	P	131.83	PANK1	10q23.31
1560028_at	0.98	0.90	P	83.97	0.43	0.01	P,M,A	35.90	FLJ10726	11q23.2
227920_at	1.00	0.96	P	340.50	0.43	0.01	P	145.03	KIAA1553	6q21
204165_at	0.99	0.95	P	528.27	0.43	0.00	P	224.47	WASF1	6q21-q22
1564651_at	1.00	0.96	P	144.83	0.43	0.00	P	61.73	LOC221710	6p24.1
208634_s_at	1.00	0.99	P	1621.07	0.43	0.00	P	687.77	MACF1	1p32-p31
230647_at	0.99	0.95	P,A	96.00	0.43	0.00	A	40.63	FLJ10597	1p34.1
226568_at	1.00	0.96	P	203.47	0.43	0.00	P	86.60	LOC284611	1p13.3
209678_s_at	1.00	0.96	P	2050.80	0.43	0.00	P	872.60	PRKCI	3q26.3
230588_s_at	0.99	0.94	P	238.53	0.43	0.01	P	101.57	LOC285074	2p11.1
230433_at	0.99	0.93	P	112.50	0.43	0.00	P,M,A	47.77		1p22.1
204306_s_at	1.00	0.96	P	580.37	0.43	0.01	P	246.87	CD151	11p15.5
217604_at	0.99	0.92	P	227.90	0.43	0.03	P	97.77		
222612_at	1.00	0.95	P	240.57	0.43	0.00	P	102.27	PSPC1	13q12.11
227706_at	0.99	0.92	P,M,A	101.83	0.43	0.00	A	43.70	SPIRE2	16q24
209153_s_at	1.00	0.98	P	484.20	0.43	0.02	P	207.73	TCF3	19p13.3
229955_at	1.00	0.96	P	263.70	0.43	0.00	P	113.13	FBXO3	11p13

229982_at	0.99	0.93	P	1935.67	0.43	0.00	P	831.20	FLJ21924	11p13
202289_s_at	1.00	0.97	P	281.60	0.43	0.01	P	120.20	TACC2	10q26
243515_at	1.00	0.97	P	97.63	0.43	0.00	P	41.63	C22orf23	22q13.1
214092_x_at	1.00	0.98	P	368.97	0.43	0.00	P	157.37	SFRS14	19p12
218060_s_at	1.00	0.97	P	547.80	0.43	0.01	P	235.37	FLJ13154	16q13
221219_s_at	1.00	0.96	P	273.77	0.43	0.00	P	116.87	DKFZp434G0522	16q24.3
220933_s_at	1.00	0.98	P	241.00	0.43	0.01	P	103.03	ZCCHC6	9q21
206240_s_at	0.99	0.94	P	161.63	0.43	0.01	P	69.33	ZNF136	19p13.2-p13.12
208407_s_at	1.00	0.99	P	875.97	0.43	0.01	P	375.50	CTNND1	11q11
208809_s_at	1.00	0.99	P	1754.23	0.43	0.02	P	754.00	C6orf62	6p22.1
219966_x_at	1.00	0.96	P	486.80	0.43	0.02	P	209.03	BANP	16q24
225673_at	1.00	0.98	P	1577.30	0.43	0.01	P	673.80	MYADM	19q13.42
222472_at	1.00	0.97	P	687.10	0.43	0.00	P	292.80	AFTIPHILIN	2p15
225441_x_at	1.00	0.97	P	690.03	0.43	0.00	P	294.50	MGC14151	17p13.2
227246_at	0.99	0.93	P	211.77	0.43	0.01	P	91.00	PLRG1	4q31.2-q32.1
203162_s_at	1.00	0.97	P	929.33	0.43	0.00	P	396.37	KATNB1	16q13
206600_s_at	1.00	0.98	P	223.20	0.43	0.01	A	95.60	SLC16A5	17q25.2
204512_at	0.99	0.93	P	237.83	0.44	0.01	P	102.40	HIVEP1	6p24-p22.3
230127_at	1.00	0.98	P	85.83	0.44	0.01	P	36.80		
202207_at	0.99	0.94	P	167.03	0.44	0.01	M,A	72.47	ARL7	2q37.2
226261_at	1.00	0.96	P	131.80	0.44	0.01	P	56.80	ZNRF2	7p15.1
204142_at	1.00	0.96	P	1013.77	0.44	0.02	P	436.07	HSRTSBETA	18p11.32
204710_s_at	1.00	0.98	P	1245.33	0.44	0.00	P	533.33	DKFZP434J154	7p22.2
226151_x_at	1.00	0.97	P	307.77	0.44	0.01	P	132.20	CRYZL1	21q21.3
219147_s_at	1.00	0.99	P	354.33	0.44	0.00	P	151.97	C9orf95	9q21.31
225679_at	1.00	0.97	P	678.10	0.44	0.00	P	291.00	C14orf35	14q22.3
214775_at	0.99	0.93	P	82.40	0.44	0.03	P,M	36.33	N4BP3	5q35.3
228498_at	0.98	0.90	P	837.60	0.44	0.02	P	363.40	B4GALT1	9p13
208713_at	1.00	0.97	P	1738.87	0.44	0.00	P	745.70	HNRPUL1	19q13.31
202847_at	1.00	0.98	P	416.60	0.44	0.01	P	178.87	PCK2	14q11.2
226850_at	1.00	0.99	P	403.53	0.44	0.00	P	173.17	SUMF1	3p26.2
233609_at	0.99	0.95	P,A	32.37	0.44	0.03	A	14.17	PTPRK	6q22.2-23.1
203243_s_at	0.99	0.93	P	1289.30	0.44	0.00	P	555.80	LIM	4q22
218751_s_at	1.00	0.97	P	69.90	0.44	0.02	P,M	30.27	FBXW7	4q31.3
213407_at	1.00	0.96	P	306.97	0.44	0.01	P,M	132.03	KIAA0931	16q22.2
223162_s_at	1.00	0.97	P	514.03	0.44	0.00	P	220.67	LCHN	7q34
238587_at	1.00	0.97	P	274.30	0.44	0.01	P	118.40	KIAA1959	11q24.1
223350_x_at	0.98	0.90	P	1893.97	0.44	0.01	P	814.77	LIN7C	11p14
217369_at	0.99	0.95	M,A	124.93	0.44	0.02	P	54.53		
231864_at	0.98	0.90	P	319.00	0.44	0.02	P	138.03	ZNF33A	10p11.2
50376_at	0.99	0.94	P	375.53	0.44	0.00	P	161.07	ZNF444	19q13.43
212955_s_at	1.00	0.99	P	1482.67	0.44	0.00	P	637.60	POLR2I	19q12
225835_at	1.00	0.99	P	1300.03	0.44	0.02	P	562.40	SLC12A2	5q23.3
225781_at	1.00	0.97	P	296.70	0.44	0.01	P	129.03	MAPK9	5q35
226099_at	1.00	0.98	P	428.67	0.44	0.00	P	184.77	ELL2	5q15
226604_at	1.00	0.99	P	371.87	0.44	0.01	P	160.30	SMILE	12q21.33
202801_at	0.99	0.93	P	1058.77	0.44	0.00	P	454.67	PRKACA	19p13.1
210800_at	0.98	0.90	P	37.67	0.44	0.01	P	16.33	TIMM8A	xq22.1
225638_at	1.00	0.97	P	1147.60	0.44	0.02	M,A	498.67		1q42.3
229063_s_at	0.99	0.92	P	250.67	0.44	0.02	P	108.47	MGC31967	9p13.2
231981_at	1.00	0.98	P	85.47	0.44	0.00	P	36.93	PRLR	5p14-p13
225589_at	1.00	0.97	P	407.73	0.44	0.02	P	177.83	SH3MD2	4q32.3
213243_at	0.99	0.95	P	338.33	0.44	0.02	P	147.07	COH1	8q22.2
201319_at	1.00	0.98	P	1860.83	0.44	0.00	P	803.27	MRCL3	18p11.31
209007_s_at	1.00	0.96	P	718.80	0.44	0.00	P	310.13	NPD014	1p36.13-p35.1
213203_at	1.00	0.97	P	579.23	0.44	0.01	P	251.00	SNAPC5	15q22.2
225325_at	1.00	0.99	P	834.13	0.44	0.01	P	361.67	FLJ20160	2q32.3
242143_at	0.98	0.90	P	209.30	0.44	0.01	P	90.30		
223090_x_at	1.00	0.99	P	819.57	0.44	0.01	P	355.67	VEZATIN	12q23.1
243736_at	1.00	0.95	P,A	100.00	0.44	0.02	A	43.97		
229535_at	1.00	0.99	P	296.33	0.44	0.00	A	128.73	LOC114971	11p11.2
232323_s_at	0.99	0.95	P	302.50	0.44	0.00	P	131.37	TTC17	11p11.2
210580_x_at	1.00	0.97	P	633.30	0.44	0.01	P	274.63	SULT1A3	16p11.2
226058_at	0.99	0.95	P	67.93	0.44	0.00	A	29.50	MGC4655	16q22.1
203787_at	1.00	0.99	P	252.20	0.44	0.00	P	109.33	SSBP2	5q14.1
218224_at	1.00	0.98	P	269.77	0.44	0.00	P	116.87	PNMA1	14q24.2
218528_s_at	1.00	0.97	P	1090.97	0.44	0.00	P	473.20	RNF38	9p13-p12
212856_at	1.00	0.95	P	316.17	0.44	0.00	P	136.67	KIAA0767	22q13.31
232589_at	0.99	0.94	P,A	108.23	0.44	0.02	A	47.83		
201620_at	1.00	0.95	P	580.93	0.44	0.00	P	252.60	MBTPS1	16q24
230256_at	0.99	0.94	P	392.03	0.44	0.03	P	172.10	FLJ35976	1q22
228282_at	0.99	0.95	P	166.13	0.44	0.01	P	72.57		
212132_at	1.00	0.96	P	839.77	0.44	0.02	P	368.83	C19orf13	19q13.12
215808_at	1.00	0.95	P	107.80	0.44	0.02	A	47.50	KLK10	19q13.3-q13.4
205112_at	0.99	0.94	P	87.80	0.44	0.00	P,M,A	38.33	PLCE1	10q23
202054_s_at	1.00	0.98	P	244.87	0.44	0.02	P	107.27	ALDH3A2	17p11.2
1552767_a_at	0.99	0.93	P,A	30.20	0.44	0.01	A	13.37	HS6ST2	xq26.2
220056_at	0.99	0.94	P	64.53	0.44	0.03	P,A	28.77	IL22RA1	1p36.11
228029_at	0.99	0.94	P	333.13	0.44	0.00	P	145.07	KIAA1982	4p16.3
227754_at	0.99	0.94	P	227.63	0.44	0.02	P	100.13	USP54	10q22.3
221988_at	1.00	0.97	P	237.50	0.44	0.01	P	103.47		
204658_at	1.00	0.97	P	615.93	0.44	0.00	P	268.80	TRA2A	7p15.3
228650_at	0.99	0.93	P	364.10	0.44	0.00	P	157.87		
209507_at	1.00	0.98	P	1689.57	0.44	0.00	P	737.47	RPA3	7p22
224923_at	0.99	0.91	P	181.47	0.44	0.01	P	79.00	TTC7	2p21
224849_at	0.99	0.94	P	779.47	0.44	0.00	P	341.80	TTC17	11p11.2
225649_s_at	1.00	0.96	P	673.67	0.44	0.01	P	293.77	STK35	20p13
219826_at	1.00	0.98	P,M,A	294.87	0.44	0.00	A	128.60	FLJ23233	19q13.43
223808_s_at	1.00	0.99	P	620.43	0.44	0.00	P	270.77	LOC114971	11p11.2
217728_at	1.00	0.97	P	6847.63	0.44	0.00	P	2994.27	S100A6	1q21
200860_s_at	1.00	0.98	P	2120.60	0.44	0.00	P	926.23	KIAA1007	16q13
207160_at	0.99	0.92	P,A	20.43	0.44	0.02	A	9.07	IL12A	3p12-q13.2
207125_at	0.99	0.94	P	36.10	0.44	0.01	P,A	15.90	ZNF225	19q13.2
201955_at	1.00	0.96	P	2512.83	0.44	0.00	P	1099.90	CCNC	6q21
235457_at	1.00	0.98	P	712.03	0.44	0.01	P	312.00	MAML2	11q21
229711_s_at	1.00	0.99	P	325.80	0.44	0.01	P	142.70	MGC5370	12q14.3

204327_s_at	1.00	0.99	P	225.67	0.44	0.00	P	98.90	ZNF202	11q23.3
218165_at	0.99	0.92	P	1295.83	0.44	0.00	P	567.90	FLJ11730	1p35.3-p33
1554724_at	1.00	0.95	P,A	28.70	0.44	0.00	A	12.63	SLC6A11	3p25.3
242826_at	0.99	0.94	P	148.57	0.45	0.03	P,A	65.97		
216863_s_at	0.99	0.93	P	365.57	0.45	0.00	P	161.57	ZCWCC1	22q12.2
202524_s_at	0.99	0.95	P	95.80	0.45	0.01	P,A	42.27	SPOCK2	10pter-q25.3
200975_at	1.00	0.98	P	3509.20	0.45	0.00	P	1538.93	PPT1	1p32
225534_at	1.00	0.97	P	672.87	0.45	0.00	P	295.60	LOC114926	8p11.21
225474_at	0.99	0.95	P,A	133.40	0.45	0.01	A	59.00	BAIAP1	3p14.1
202094_at	1.00	0.96	P	657.10	0.45	0.01	P	289.30	BIRC5	17q25
202275_at	0.99	0.94	P	1081.23	0.45	0.01	P	476.67	G6PD	xq28
225120_at	1.00	0.97	P	968.17	0.45	0.01	P	426.17	PURB	7p13
225716_at	1.00	0.95	P	1205.17	0.45	0.01	P	531.87		
226236_at	1.00	0.96	P	1615.77	0.45	0.02	P	716.73		20p11.23
209179_s_at	1.00	0.96	P	717.47	0.45	0.01	P	317.33	LENG4	19q13.4
227624_at	1.00	0.95	P	267.53	0.45	0.01	P	118.17	KIAA1546	4q24
202266_at	1.00	0.97	P	1899.17	0.45	0.00	P	836.43	TTRAP	6p22.3-p22.1
214960_at	1.00	0.95	P	298.70	0.45	0.03	P	132.53	API5	11p12-q12
226336_at	1.00	0.97	P	1456.47	0.45	0.00	P	642.73	PPIA	7p13-p11.2
209094_at	1.00	0.98	P	1082.07	0.45	0.00	P	476.40	DDAH1	1p22
228027_at	1.00	0.98	P,M	251.27	0.45	0.00	A	110.53	LOC114928	xq22.1
202772_at	1.00	0.98	P,A	228.67	0.45	0.00	A	100.77	HMGCL	1p36.1-p35
1558755_x_at	0.99	0.95	P	69.87	0.45	0.01	P,M	31.03	LOC284390	19p13.2
1556725_a_at	1.00	0.95	P	102.47	0.45	0.01	P,A	45.40		
216733_s_at	0.99	0.95	P,A	62.93	0.45	0.03	A	28.47	GATM	15q15.1
232466_at	0.99	0.94	P	118.67	0.45	0.03	P	52.90	CUL4A	13q34
218446_s_at	1.00	0.97	P	313.37	0.45	0.01	P	139.07	FAM18B	17p11.2
215291_at	0.99	0.92	P,A	32.10	0.45	0.01	A	14.20	MKL1	22q13
201250_s_at	1.00	1.00	P	558.90	0.45	0.00	P	246.50	SLC2A1	1p35-p31.3
1553704_x_at	1.00	0.97	P	490.60	0.45	0.01	P	216.43	FLJ90396	19p13.2
235679_at	1.00	0.96	P	294.20	0.45	0.01	P	131.17		
238681_at	1.00	0.95	P	141.27	0.45	0.01	P	62.53	FLJ37451	17q23.2
218931_at	0.99	0.95	P	246.77	0.45	0.02	A	109.20	RAB17	2q37.3
226318_at	1.00	0.97	P	252.80	0.45	0.00	P,A	111.67	TBRG1	11q24.2
242663_at	0.99	0.95	P	105.80	0.45	0.00	P	46.63		
217223_s_at	1.00	0.99	P	144.67	0.45	0.00	A	63.93	BCR	22q11.23
203744_at	1.00	0.97	P	1740.60	0.45	0.00	P	770.47	HMGB3	xq28
222457_s_at	1.00	0.96	P	307.30	0.45	0.02	P	136.80	EPLIN	12q13
218797_s_at	1.00	0.96	P	379.43	0.45	0.02	P	168.73	SIRT7	17q25
200797_s_at	1.00	0.96	P	2722.87	0.45	0.00	P	1200.90	MCL1	1q21
208632_at	1.00	0.96	P	305.67	0.45	0.01	P	136.07	RNF10	12q24.31
211475_s_at	0.99	0.95	P	1194.50	0.45	0.00	P	529.37	BAG1	9p12
224897_at	1.00	0.95	P	294.67	0.45	0.01	P	130.80	WDR26	1q42.12-q42.13
232336_at	1.00	0.96	P	129.97	0.45	0.00	A	57.43	ZSWIM5	1p34.1
203015_s_at	0.99	0.95	P	293.07	0.45	0.01	P	130.50	SSX2IP	1p22.3
219549_s_at	0.99	0.92	P	1374.53	0.45	0.00	P	611.63	RTN3	11q13
217892_s_at	1.00	0.95	P	1158.20	0.45	0.00	P	514.80	EPLIN	12q13
200710_at	1.00	0.97	P	1147.67	0.45	0.00	P	507.53	ACADVL	17p13-p11
210959_s_at	0.99	0.93	P	603.50	0.45	0.00	P	268.80	SRD5A1	5p15
225412_at	1.00	0.96	P	428.00	0.45	0.00	P	190.23	FLJ14681	2q13
208940_at	0.99	0.92	P	231.87	0.45	0.00	M,A	102.63	SEPHS1	10p14
202439_s_at	1.00	0.98	P	259.33	0.45	0.00	P	115.30	IDS	xq28
218986_s_at	0.99	0.95	P	97.80	0.45	0.01	P	43.67	FLJ20035	4q32.3
218584_at	0.98	0.91	P,A	233.80	0.45	0.01	A	103.63	FLJ21127	12q24.13
226810_at	1.00	0.99	P	562.40	0.45	0.00	P	249.93		
229057_at	0.99	0.94	P	13.70	0.45	0.01	A	6.17		
32069_at	1.00	0.97	P	479.93	0.45	0.00	P	213.20	N4BP1	16q12.1
224129_s_at	1.00	0.97	P	1591.53	0.45	0.00	P	708.30	LOC84661	2p23.2
200779_at	1.00	0.99	P	4872.90	0.45	0.00	P	2168.00	ATF4	22q13.1
213546_at	0.99	0.92	P	285.97	0.45	0.00	P	127.17	DKFZp586I1420	7p15.1
204937_s_at	1.00	0.96	P	757.13	0.45	0.00	P	336.67	ZNF274	19qter
212479_s_at	0.99	0.92	P	306.30	0.45	0.01	P	137.17	FLJ13910	2p11.2
202365_at	1.00	0.97	P	510.87	0.45	0.00	P	227.33	MGC5139	12q24.31
238653_at	1.00	0.98	P	291.97	0.45	0.00	P	130.10	LRIG2	1p13.1
217940_s_at	1.00	0.95	P	1006.63	0.45	0.01	P	449.43	FLJ10769	13q34
213204_at	0.99	0.93	P	175.10	0.45	0.00	P	77.70	PARC	6p21.1
230339_at	0.99	0.92	P	113.10	0.45	0.00	P	50.23	FLJ32745	2q12.3
221621_at	1.00	0.96	P	175.20	0.45	0.01	P	78.37		
218720_x_at	0.99	0.94	P	349.40	0.45	0.00	M,A	155.77	PSK-1	16p12.1
211528_x_at	1.00	0.97	P	1477.00	0.45	0.01	P	657.90	HLA-C	6p21.3
224478_s_at	1.00	0.98	P	464.37	0.45	0.00	P	206.53	MGC11257	7p22.3
223287_s_at	1.00	0.96	P	175.30	0.45	0.00	A	78.37	FOXP1	3p14.1
1563051_at	0.99	0.94	P	47.37	0.45	0.01	P,A	21.10	OSBP	11q12-q13
213221_s_at	1.00	0.97	P	217.80	0.45	0.01	P	97.30	SIK2	11q23.2
205062_x_at	0.99	0.93	P	178.13	0.45	0.02	P	80.43	ARID4A	14q22.3-q23.1
226838_at	0.99	0.93	P	245.23	0.45	0.02	P	109.53		2p24.3
219409_at	1.00	0.99	P	260.93	0.45	0.00	P	116.63	SNIP1	1p34.2
215128_at	1.00	0.96	P	258.77	0.45	0.00	P	115.43		
202901_x_at	0.99	0.94	P	25.00	0.45	0.02	P,A	11.40	CTSS	1q21
225898_at	0.99	0.92	P	238.87	0.45	0.01	P	106.50	FLJ12953	2p13.1
229606_at	1.00	0.95	P	1543.33	0.45	0.00	P	690.33	PPP3CA	4q21-q24
213260_at	0.99	0.92	P	254.57	0.45	0.01	P	114.17	FOXC1	6p25
210252_s_at	0.99	0.94	P,M	281.73	0.45	0.03	A	127.50	MADD	11p11.2
220956_s_at	0.99	0.92	P	569.13	0.45	0.03	P	257.33	EGLN2	19q13.2
208771_s_at	1.00	0.99	P	809.83	0.45	0.00	P	361.97	LTA4H	12q22
218669_at	0.99	0.95	P	1165.00	0.45	0.01	P	522.80	RAP2C	xq25
219481_at	1.00	0.98	P	326.20	0.45	0.00	P,A	145.93	TTC13	1q42.2
236545_at	1.00	0.98	P	122.37	0.45	0.01	P	54.77		
223389_s_at	1.00	0.96	P	270.03	0.45	0.02	P,M,A	121.63	ZNF581	19q13.43
203762_s_at	1.00	0.98	P,M	108.00	0.45	0.01	A	48.60	D2LIC	2p25.1-p24.1
225775_at	1.00	0.98	P	110.73	0.45	0.00	A	49.67	MGC50844	7q32.3
226015_at	1.00	0.98	P	360.40	0.45	0.00	P	161.33	ZNF325	7p22.2
244753_at	1.00	0.95	P	181.87	0.45	0.02	P	81.70		
1552287_s_at	0.99	0.93	P	316.30	0.46	0.01	P	142.53	AFG3L1	16q24
205171_at	1.00	0.98	P	262.87	0.46	0.00	A	118.03	PTPN4	2q14.2
238483_at	0.99	0.94	P	20.20	0.46	0.00	A	9.07		

224802_at	0.99	0.93	P	1340.17	0.46	0.01	P	603.00	NDFIP2	13q22.2
1556389_at	1.00	0.97	P	92.07	0.46	0.01	P,A	41.30	TNRC5	6pter-p12.1
235507_at	1.00	0.96	P	108.70	0.46	0.00	P	48.80	LOC115294	8q11.22
89476_r_at	1.00	0.95	P	49.73	0.46	0.02	A	22.70	NPEPL1	20q13.32
208617_s_at	1.00	0.98	P	1621.20	0.46	0.00	P	726.87	PTP4A2	1p35
226230_at	1.00	0.98	P	363.07	0.46	0.00	P	163.33	KIAA1387	2p16.2
1554889_at	0.99	0.94	P	68.17	0.46	0.01	P	30.97	TIA1	2p13
214965_at	1.00	0.95	P	180.93	0.46	0.02	P	82.20	MGC26885	16q24.3
238590_x_at	1.00	0.98	P	277.33	0.46	0.00	P	124.77	MGC10744	17p13.1
224685_at	0.99	0.94	P	595.10	0.46	0.01	P	268.53	MLLT4	6q27
223394_at	1.00	0.99	P	503.93	0.46	0.01	P	227.43	SERTAD1	19q13.1-q13.2
222200_s_at	0.99	0.95	P	230.87	0.46	0.00	P	104.43	FLJ10276	1p34.3
231810_at	1.00	0.96	P	604.17	0.46	0.01	P	272.87	BRI3BP	12q24.31
216220_s_at	0.99	0.95	P	97.97	0.46	0.01	M,A	44.40	ADORA1	1q32.1
212872_s_at	0.99	0.94	P	331.10	0.46	0.01	P	148.80	USP49	6p21.1
209442_x_at	1.00	0.96	P	164.43	0.46	0.03	P,M	75.10	ANK3	10q21
225060_at	1.00	0.97	P	1039.47	0.46	0.01	P	470.23	LRP11	6q24.3
218174_s_at	0.99	0.93	P	134.40	0.46	0.01	P	60.83	C10orf57	10q23.1
218641_at	1.00	0.98	P	281.60	0.46	0.01	P	127.17	MGC3032	11q13
219307_at	0.99	0.94	P	332.07	0.46	0.01	P	150.33	C6orf210	6q21
206745_at	1.00	0.98	P,M,A	162.57	0.46	0.00	A	73.47	HOXC11	12q13.3
228239_at	1.00	0.96	P	153.67	0.46	0.01	P	69.73	C21orf51	21q22.12
1561266_at	0.99	0.94	P	63.73	0.46	0.02	A	29.27		3q29
224504_s_at	1.00	0.98	P	329.90	0.46	0.00	P	148.90	MGC13125	11q23.3
208818_s_at	1.00	0.97	P	2636.37	0.46	0.00	P	1194.93	COMT	22q11.21
211724_x_at	1.00	0.98	P	409.70	0.46	0.00	P	185.40	FLJ20323	7p22-p21
1564630_at	1.00	0.96	P	88.03	0.46	0.01	M,A	40.13	EDN1	6p24.1
211596_s_at	0.99	0.95	P,A	434.93	0.46	0.01	P,M	197.33	LRIG1	3p14
202051_s_at	0.99	0.94	P	422.43	0.46	0.00	P	191.43	ZNF262	1p32-p34
223798_at	0.99	0.92	P	79.97	0.46	0.00	A	36.40	SLC41A2	12q24.11
212541_at	1.00	0.97	P	607.23	0.46	0.01	P	275.27	PP591	1q22
203763_at	1.00	0.96	P	171.93	0.46	0.02	P	78.00	D2LIC	2p25.1-p24.1
211975_at	0.99	0.94	P	879.70	0.46	0.01	P	398.53	ZNF289	11p11.2-p11.12
235866_at	0.99	0.94	P	52.73	0.46	0.01	P	24.23	C9orf85	9q21.2
225209_s_at	1.00	0.97	P	1044.37	0.46	0.01	P	473.40	UBE2J2	1p36.33
212551_at	0.99	0.94	P	741.70	0.46	0.01	P	337.30	CAP2	6p22.3
222424_s_at	1.00	0.97	P	762.23	0.46	0.01	P	345.67	NUCKS	1q32.1
223157_at	1.00	0.99	P	991.43	0.46	0.00	A	449.93	C4orf14	4q12
226139_at	1.00	0.96	P	99.33	0.46	0.00	A	45.33	DKFZp761B107	4p15.31
201350_at	1.00	0.99	P	659.10	0.46	0.00	P	299.23	FLOT2	17q11-q12
235812_at	1.00	0.98	P	674.83	0.46	0.00	P	306.50	FLJ38101	16q12.1
238523_at	1.00	0.95	P	207.63	0.46	0.03	P,M	95.27	C16orf44	16q24.1
225310_at	1.00	0.97	P	2737.17	0.46	0.00	P	1244.30	KAT3	1p22.2
226976_at	1.00	0.97	P	551.30	0.46	0.01	P	250.97	KPNA6	1p35.1-p34.3
223272_s_at	0.99	0.93	P	524.77	0.46	0.03	A	240.13	MGC13186	1q42.2
218274_s_at	1.00	0.96	P	104.50	0.46	0.03	A	47.80	FLJ10415	2q36.1
237395_at	1.00	0.96	P	38.53	0.46	0.02	M,A	17.90	CYP4Z1	1p33
1562416_at	0.99	0.94	P	108.10	0.46	0.01	P,A	49.37		
229689_s_at	1.00	0.96	P	149.27	0.46	0.01	P	67.80	DLG5	10q23
224653_at	1.00	0.97	P	527.60	0.46	0.01	P	241.23	EIF4EBP2	10q21-q22
239539_at	1.00	0.96	P	52.73	0.46	0.01	P	24.13	NEK3	13q14.13
225748_at	0.98	0.90	P	642.13	0.46	0.02	P	292.83	C6orf93	6q24.1
226386_at	1.00	0.99	P	2189.50	0.46	0.01	P	996.43	C7orf30	7p15.3
224436_s_at	0.99	0.94	P	752.27	0.46	0.01	P	343.40	NIPSNAP3A	9q31.3
203656_at	0.99	0.93	P	605.40	0.46	0.00	P	274.23	KIAA0274	6q21
228852_at	1.00	0.97	P	328.17	0.46	0.01	P	149.30	ENSA	1q21.3
226566_at	1.00	0.97	P	371.33	0.46	0.00	P	168.77	TRIM11	1q42.13
225409_at	1.00	0.97	P	243.00	0.46	0.00	P	110.97	MGAT4A	2q12
201973_s_at	1.00	1.00	P	2817.00	0.46	0.01	P	1284.83	C7orf28B	7p22.2
213932_x_at	1.00	0.98	P	4347.50	0.46	0.00	P	1982.53	HLA-A	6p21.3
209505_at	1.00	0.98	P	183.80	0.46	0.01	P	84.23	NR2F1	5q14
202531_at	1.00	0.96	P	235.53	0.46	0.00	P,M	107.90	IRF1	5q31.1
39891_at	0.99	0.93	P	199.47	0.46	0.01	P	91.27	DKFZp547K1113	15q26.1
201687_s_at	1.00	0.96	P	1799.97	0.46	0.01	P	824.37	API5	11p12-q12
203016_s_at	0.99	0.95	P	491.67	0.46	0.01	P	226.13	SSX2IP	1p22.3
201125_s_at	1.00	0.95	P	1826.57	0.46	0.01	P	834.07	ITGB5	3q21.2
212753_at	1.00	0.96	P	307.07	0.46	0.00	P	140.57	RNF3	4p16.3
225230_at	0.99	0.93	P	620.37	0.46	0.00	P	284.90	MGC54289	1p13.2
202601_s_at	0.99	0.95	P,A	182.03	0.46	0.00	P	83.50	HTATSF1	xq26.1-q27.2
222193_at	1.00	0.96	P	147.43	0.46	0.00	P,M,A	67.27	FLJ21820	2p24.2
202197_at	1.00	0.99	P	325.90	0.46	0.00	P	149.13	MTMR3	22q12.2
1556935_at	1.00	0.98	P	188.23	0.47	0.01	P	86.67		
212866_at	1.00	0.97	P	465.07	0.47	0.00	P,M	213.30	LOC203069	8p21.2
229285_at	1.00	0.97	P	62.83	0.47	0.00	M,A	28.70	RNASEL	1q25
209445_x_at	1.00	0.99	P	765.23	0.47	0.00	P	350.47	FLJ10803	7p13
230449_x_at	1.00	0.95	P	175.03	0.47	0.03	P	81.30		
225877_at	0.98	0.91	P	218.93	0.47	0.00	P	101.27	TYSND1	10q22.2
1555978_s_at	0.99	0.93	P	132.53	0.47	0.00	P	61.03	MRCL3	18p11.31
227489_at	0.98	0.91	P	162.40	0.47	0.01	P	75.53	SMURF2	17q22-q23
229268_at	1.00	0.96	P	383.10	0.47	0.01	P	176.00	RABEP1	17p13.3
201041_s_at	0.99	0.93	P	536.27	0.47	0.00	P	247.20	DUSP1	5q34
204283_at	1.00	0.95	P	586.80	0.47	0.00	P	268.80	FARS1	6p25.1
227112_at	1.00	0.97	P	312.77	0.47	0.01	P	144.63	KIAA0779	3q21.3
205750_at	1.00	0.99	P	275.07	0.47	0.01	P	126.53	BPHL	6p25
227772_at	1.00	0.95	P	314.07	0.47	0.01	P	144.73	LATS1	6q24-q25.1
203567_s_at	0.99	0.93	P	660.33	0.47	0.00	P	304.20	TRIM38	6p21.3
219394_at	1.00	0.99	P	270.47	0.47	0.01	P	124.80	PGS1	17q25.3
214603_at	0.99	0.95	P	2760.83	0.47	0.00	P	1272.57	MAGEA3	xq28
220643_s_at	1.00	0.97	P	537.30	0.47	0.02	P	249.40	FAIM	3q22.3
218637_at	1.00	0.96	P	137.57	0.47	0.01	P	63.70	IMPACT	18q11.2-q12.1
201455_s_at	1.00	0.96	P	339.10	0.47	0.01	P	156.73	NPEPPS	17q21
1556942_at	1.00	0.97	P,M,A	29.70	0.47	0.00	P,A	13.67	KIAA1126	8q24.3
219332_at	1.00	0.97	P	270.73	0.47	0.01	M,A	124.73	FLJ23471	7p22.3
1556054_at	0.99	0.92	P,A	36.97	0.47	0.01	P,A	17.17	TBC1D8	2q12.1
223742_at	1.00	0.96	P	280.53	0.47	0.01	P	129.47	MRPL4	19p13.2
222401_s_at	0.99	0.95	P	1619.60	0.47	0.00	P	747.47	SMP1	1p36.11

206109_at	1.00	0.97	P	111.50	0.47	0.01	A	52.00	FUT1	19q13.3
231092_s_at	1.00	0.99	P,A	45.10	0.47	0.00	A	20.77	ZNRF1	16q22.3
226896_at	1.00	0.96	P	1313.87	0.47	0.01	P	607.93	CHCHD1	10q22.3
235534_at	1.00	0.96	P	96.73	0.47	0.00	A	44.60		
209252_at	1.00	0.96	P	417.87	0.47	0.01	P	193.70	HARSL	5q31.3
239504_at	0.99	0.92	P	69.53	0.47	0.01	A	32.30		
207713_s_at	0.99	0.93	P	214.43	0.47	0.02	P	99.50	C20orf18	20p13
211809_x_at	0.99	0.92	P,A	64.87	0.47	0.02	A	30.23	COL13A1	10q22
203488_at	0.99	0.93	P	249.93	0.47	0.00	M,A	115.70	LPHN1	19p13.2
211657_at	1.00	0.98	P	149.93	0.47	0.02	P	69.67	CEACAM6	19q13.2
234978_at	1.00	0.96	P	237.73	0.47	0.02	A	110.70	SLC36A4	11q14.3
204784_s_at	0.99	0.93	P	517.43	0.47	0.01	P	239.77	MLF1	3q25.1
223491_at	0.99	0.93	P	654.33	0.47	0.01	P	304.17	COMMD2	3q25.1
225604_s_at	0.99	0.95	P	68.20	0.47	0.01	P,A	31.87	C9orf19	9p13-p12
201325_s_at	0.99	0.93	P	532.57	0.47	0.00	P	247.37	EMP1	12p12.3
244687_at	1.00	0.96	P	152.67	0.47	0.01	A	71.17		
228721_at	1.00	0.97	P	89.57	0.47	0.00	P	41.47	LOC339903	3p21.33
202100_at	0.99	0.94	P	961.87	0.47	0.00	P	444.27	RALB	2cen-q13
244474_at	0.99	0.94	P	185.80	0.47	0.00	P,M	85.87		
1568873_at	1.00	0.97	P	134.93	0.47	0.00	P	62.60	ZNF519	18p11.21
223195_s_at	1.00	0.95	P,A	163.57	0.47	0.00	P,M	75.97	SESN2	1p35.2
222720_x_at	0.99	0.94	P,A	31.17	0.47	0.03	A	14.83	C1orf27	1q25
242012_at	1.00	0.96	P	100.77	0.47	0.02	A	47.03		
227665_at	0.99	0.93	P	276.13	0.47	0.03	P	130.27	MCART1	9p13.3-p12
222557_at	1.00	0.96	P	93.57	0.47	0.01	A	43.83	STMN3	20q13.3
244201_at	1.00	0.95	P	113.17	0.47	0.00	P,A	52.40		
217802_s_at	1.00	0.98	P	2352.27	0.47	0.01	P	1095.17	NUCKS	1q32.1
232319_at	0.99	0.95	P	29.57	0.47	0.03	P,A	13.90		
202646_s_at	1.00	0.97	P	2414.80	0.47	0.01	P	1123.10	D1S155E	1p22
235640_at	0.99	0.95	P	115.00	0.47	0.02	P,A	53.77		
38398_at	0.99	0.94	P	289.47	0.47	0.01	P	134.37	MADD	11p11.2
243315_at	0.99	0.95	P	116.07	0.47	0.02	P,M,A	54.33		
242989_at	0.99	0.95	P	71.43	0.47	0.00	P	33.23		
227085_at	1.00	0.98	P	1095.17	0.47	0.00	P	510.53	H2AFV	7p13
202180_s_at	0.99	0.93	P	292.83	0.47	0.01	P	136.07	MVP	16p13.1-p11.2
201368_at	1.00	0.96	P	1327.00	0.47	0.02	P	622.07	ZFP36L2	2p22.3-p21
204319_s_at	1.00	0.99	P	508.83	0.47	0.00	P	236.40	RGS10	10q25
204949_at	1.00	0.97	P	859.00	0.47	0.01	P	399.40	ICAM3	19p13.3-p13.2
203823_at	1.00	0.98	P,A	223.67	0.47	0.00	A	104.13	RGS3	9q32
218961_s_at	1.00	0.99	P	437.70	0.47	0.00	P	203.57	PNKP	19q13.3-q13.4
209472_at	1.00	1.00	P	733.50	0.47	0.01	P	341.53	KAT3	1p22.2
226808_at	0.99	0.95	P	44.33	0.47	0.01	P	20.83	KIAA0543	7q36.1
200842_s_at	0.99	0.95	P	1111.20	0.47	0.02	P	521.73	EPRS	1q41-q42
201214_s_at	1.00	0.99	P	552.37	0.47	0.00	P	257.30	PPP1R7	2q37.3
242514_at	1.00	0.95	P,A	51.93	0.47	0.01	A	24.13		
228292_at	1.00	0.96	P	206.00	0.47	0.01	P	96.47	GBA2	9p13.2
229838_at	0.99	0.94	P,M,A	133.47	0.47	0.00	P,A	61.97	NUCB2	11p15.1-p14
230099_at	1.00	0.96	P	208.00	0.47	0.01	A	97.37		
208780_x_at	1.00	0.99	P	4455.73	0.47	0.00	P	2080.33	VAPA	18p11.22
225953_at	1.00	0.98	P	629.67	0.47	0.01	P	293.73	P15RS	18q12.2
209594_x_at	1.00	0.99	P	224.87	0.47	0.00	A	104.80	PSG9	19q13.2
1555487_a_at	0.99	0.94	P	316.10	0.47	0.03	A	149.03	ARP3BETA	7q32-q36
223675_s_at	0.99	0.95	P	453.40	0.47	0.02	P	213.27	VEZATIN	12q23.1
239381_at	1.00	0.96	P	164.97	0.47	0.00	P	77.27	KLK7	19q13.41
235768_at	1.00	0.98	P	85.80	0.47	0.01	A	40.43	SH3RF2	5q32
222870_s_at	0.99	0.93	P	653.40	0.47	0.00	P	306.43	B3GNT1	2p15
202215_s_at	1.00	1.00	P	899.00	0.47	0.01	P	420.33	NFYC	1p32
207122_x_at	1.00	0.97	P	385.23	0.47	0.00	A	179.73	SULT1A1	16p12.1
1557252_at	0.99	0.95	P	59.60	0.47	0.01	P	27.80		
210695_s_at	1.00	0.96	P	369.90	0.48	0.02	P	175.60	WWOX	16q23.3-q24.1
1553167_a_at	0.99	0.94	P	47.77	0.48	0.01	P	22.47	SLA/LP	4p15.31
234950_s_at	1.00	0.99	P	544.30	0.48	0.01	P	254.90	COP1	1q24.2-q24.3
216129_at	0.99	0.93	P,A	28.23	0.48	0.01	A	13.30	ATP9A	20q13.11-q13.2
235117_at	1.00	0.99	P	825.80	0.48	0.00	P	388.07		
207513_s_at	1.00	0.98	P	274.57	0.48	0.02	P	129.50	ZNF189	9q22-q31
213215_at	0.99	0.95	P	379.43	0.48	0.01	P	177.27		
202734_at	1.00	0.96	P	667.60	0.48	0.00	P	313.23	TRIP10	19p13.3
228480_at	1.00	0.95	P	124.87	0.48	0.00	P	58.40	VAPA	18p11.22
225480_at	1.00	0.96	P	1551.53	0.48	0.00	P	725.23	FLJ45459	1p34.2
201063_at	1.00	0.98	P	2471.80	0.48	0.01	P	1157.93	RCN1	11p13
200670_at	1.00	0.96	P	1433.03	0.48	0.00	P	673.20	XBP1	22q12.1
208021_s_at	1.00	0.96	P	292.87	0.48	0.02	P	138.60	RFC1	4p14-p13
235159_at	0.99	0.94	P	154.77	0.48	0.02	P	73.03		
239538_at	0.99	0.92	P	91.43	0.48	0.01	P,A	43.07	DKFZP434B1727	2q21.3
207358_x_at	1.00	0.96	P	1128.90	0.48	0.01	P	532.87	MACF1	1p32-p31
209608_s_at	1.00	0.97	P	1467.17	0.48	0.00	P	690.13	ACAT2	6q25.3-q26
219296_at	1.00	0.99	P	334.03	0.48	0.01	P	157.40	ZDHHC13	11p15.1
212748_at	1.00	0.96	P	290.80	0.48	0.00	P	137.13	MKL1	22q13
225745_at	1.00	0.98	P	316.73	0.48	0.01	P	149.33	LRP6	12p11-p13
218578_at	1.00	0.98	P	563.27	0.48	0.01	P	265.67	HRPT2	1q25
201724_s_at	1.00	0.97	P	341.20	0.48	0.00	P	160.87	GALNT1	18q12.1
218018_at	0.99	0.94	P	2057.17	0.48	0.02	P	973.67	PDXK	21q22.3
209204_at	0.99	0.95	P	83.53	0.48	0.01	P	39.73	LMO4	1p22.3
201695_s_at	1.00	0.95	P	807.70	0.48	0.00	P	382.27	NP	14q13.1
203322_at	0.99	0.92	P	228.20	0.48	0.02	P	108.43	KIAA0863	18q23
209146_at	1.00	0.97	P	1953.00	0.48	0.00	P	921.60	SC4MOL	4q32-q34
241379_at	1.00	0.98	P	20.77	0.48	0.01	P,A	9.83	C2orf13	2p13.3
243490_at	0.99	0.94	P	71.93	0.48	0.02	P	34.17		
219313_at	1.00	0.98	P	88.90	0.48	0.02	M,A	42.20	DKFZp434C0328	3q13.31
213373_s_at	0.99	0.94	P	685.00	0.48	0.01	P	324.57	CASP8	2q33-q34
228697_at	1.00	0.99	P	761.60	0.48	0.00	P	359.70	HINT3	6q22.33
221588_x_at	1.00	0.97	P	235.93	0.48	0.00	M,A	111.53	ALDH6A1	14q24.3
204120_s_at	1.00	0.99	P	899.33	0.48	0.01	P	425.70	ADK	10q22
223637_s_at	0.99	0.95	P	227.27	0.48	0.00	P	107.27	DKFZP566M1046	11p15.4
51158_at	1.00	0.97	P	391.73	0.48	0.02	P	186.17	LOC400451	15q26.1
220073_s_at	1.00	0.96	P	121.47	0.48	0.02	P,A	57.83	FLJ10665	12p13.32

214773_x_at	1.00	0.99	P	642.03	0.48	0.00	P	304.53	MGC3794	1q23.2
200076_s_at	1.00	0.98	P	1045.03	0.48	0.01	P	497.37	MGC2749	19p13.11
235492_at	1.00	0.95	P,M	128.13	0.48	0.00	A	61.03	IBRDC1	6q22.33
226050_at	1.00	0.96	P	1000.67	0.48	0.00	P	477.53	C13orf11	13q34
220741_s_at	1.00	0.95	P	794.13	0.48	0.01	P	378.07	PPA2	4q25
211385_x_at	1.00	0.99	P	304.80	0.48	0.01	A	145.27	SULT1A1	16p12.1
208384_s_at	1.00	0.96	P	201.83	0.48	0.01	P,A	96.07	MID2	xq22
202378_s_at	0.99	0.94	P	999.57	0.48	0.01	P	475.17	OBRGRP	1p31.3
202980_s_at	0.99	0.92	P	147.77	0.48	0.00	P	70.57	SIAH1	16q12
212760_at	1.00	0.96	P	511.60	0.48	0.00	P	243.77	C6orf133	6p21.1
214585_s_at	0.99	0.94	P	924.07	0.48	0.00	P	439.53	VPS52	6p21.3
206949_s_at	1.00	0.98	P	1156.23	0.48	0.00	P	551.87	RUSC1	1q21-q22
212286_at	0.99	0.93	P	241.60	0.48	0.01	P	115.43	ANKRD12	18p11.22
90265_at	0.99	0.94	P	224.57	0.48	0.01	A	107.23	CENTA1	7p22.3
225912_at	0.99	0.94	P	151.90	0.48	0.00	P,M,A	72.07	TP53INP1	8q22
224509_s_at	0.99	0.93	P	304.83	0.48	0.02	P	145.90	RTN4IP1	6q21
203610_s_at	1.00	0.96	P	426.73	0.48	0.00	P	204.20	TRIM38	6p21.3
210570_x_at	0.99	0.94	P,A	362.33	0.48	0.01	A	174.70	MAPK9	5q35
208092_s_at	0.99	0.94	P	104.60	0.48	0.02	A	50.07	DKFZP566A1524	2p24.3
243947_s_at	0.99	0.93	P	99.13	0.48	0.02	P	47.80		
207416_s_at	0.99	0.92	P	113.40	0.48	0.02	P	54.67	NFATC3	16q22.2
203606_at	0.99	0.93	P	3763.33	0.48	0.02	P	1799.97	NDUFS6	5p15.33
208690_s_at	1.00	0.99	P	1567.10	0.48	0.01	P	749.63	PDLIM1	10q22-q26.3
1553274_a_at	1.00	0.95	P	239.03	0.49	0.03	P	114.97	C6orf151	6p24.3
227785_at	1.00	0.97	P,A	95.43	0.49	0.01	A	45.63	SDCCAG8	1q43-q44
204064_at	1.00	0.97	P	796.53	0.49	0.00	P	379.73	THOC1	18p11.32
217125_at	0.99	0.93	P,M,A	86.47	0.49	0.01	A	41.77	UBB	17p12-p11.2
217798_at	1.00	0.98	P	506.63	0.49	0.02	P	243.13	CNOT2	12q14.3-q15
1557965_at	1.00	0.98	P	315.27	0.49	0.01	P	151.70	MGC61716	2q37.3
201315_x_at	0.99	0.93	P	709.97	0.49	0.02	P	341.13	IFITM3	11p15.5
223305_at	1.00	0.96	P	222.67	0.49	0.00	M,A	107.10	MGC:13379	11q13.1
40359_at	1.00	0.99	P	621.17	0.49	0.00	P	297.17	C11orf13	11p15.5
212983_at	1.00	0.97	P	405.17	0.49	0.01	P	194.23	HRAS	11p15.5
204028_s_at	1.00	0.96	P	624.33	0.49	0.00	P	298.27	RABGAP1	9q34.11
227701_at	0.99	0.94	P	314.80	0.49	0.02	P	151.97	C10orf118	10q26.11
203450_at	1.00	0.96	P,A	341.80	0.49	0.01	A	164.67	C22orf2	22q12
235145_at	1.00	0.95	P,A	171.43	0.49	0.00	A	82.37	ZFP67	1q22
1553703_at	1.00	0.96	P	634.13	0.49	0.02	P	304.97	FLJ90396	19p13.2
1560049_at	0.99	0.93	P	191.80	0.49	0.03	P	93.07		
218668_s_at	1.00	0.98	P	1194.80	0.49	0.01	P	572.40	RAP2C	xq25
224782_at	1.00	0.97	P	1193.20	0.49	0.00	P	571.73	FLJ31121	5q31.3
201697_s_at	1.00	0.97	P	2595.70	0.49	0.00	P	1245.67	DNMT1	19p13.2
238889_at	1.00	0.95	P,M,A	42.67	0.49	0.03	A	20.73	FLJ21839	2p23.3
238327_at	1.00	0.95	P	65.87	0.49	0.01	A	31.73	ECGF1	22q13.33
53968_at	0.99	0.92	P	691.60	0.49	0.01	P	331.27	KIAA1698	11q12.3
222732_at	1.00	0.99	P	413.53	0.49	0.00	P	199.00	TRIM39	6p21.3
213590_at	1.00	0.99	P	187.00	0.49	0.00	P	90.07	SLC16A5	17q25.2
214934_at	1.00	0.97	P	97.20	0.49	0.00	P,A	46.57	ATP9B	18q23
201407_s_at	1.00	1.00	P	1236.27	0.49	0.02	P	596.60	PPP1CB	2p23
227996_at	1.00	0.96	P	103.33	0.49	0.00	A	49.80	FARP1	13q32.2-q32.3
212991_at	0.99	0.94	P	87.47	0.49	0.02	A	42.33	FBXO9	6p12.3-p11.2
223849_s_at	1.00	0.97	P	188.43	0.49	0.00	P	90.43	MOV10	1p13.1
229854_at	0.99	0.94	P	82.77	0.49	0.00	A	39.73	OBSCN	1q42
225203_at	1.00	0.98	P	216.17	0.49	0.02	P,A	104.87	PPP1R16A	8q24.3
212289_at	1.00	0.97	P	228.73	0.49	0.01	P	110.20	ANKRD12	18p11.22
1553325_at	0.99	0.94	P,M,A	58.33	0.49	0.03	M,A	28.77	FLJ25680	5q15
213311_s_at	0.99	0.94	P	622.03	0.49	0.03	P	301.40	KIAA1049	16q24.3
215071_s_at	1.00	0.97	P	334.73	0.49	0.02	P	161.77	HIST1H2AC	6p21.3
202671_s_at	1.00	0.98	P	1828.33	0.49	0.00	P	880.90	PDXK	21q22.3
202754_at	1.00	0.96	P	1118.53	0.49	0.01	P	539.27	R3HDM	2q21.3
226143_at	1.00	0.98	P	1158.17	0.49	0.01	P	559.53	RAI1	17p11.2
226640_at	1.00	0.95	P	197.30	0.49	0.01	P	95.20	LOC221955	7p22.2
207196_s_at	1.00	0.99	P	540.33	0.49	0.01	P	261.17	TNIP1	5q32-q33.1
214352_s_at	1.00	0.96	P	1213.30	0.49	0.00	P	584.37	KRAS2	12p12.1
222689_at	0.98	0.91	P	401.07	0.49	0.01	P	195.13	PHCA	11q13.3
230375_at	1.00	0.97	P	106.20	0.49	0.02	P	51.50	C6orf111	6q16.3
62212_at	1.00	0.98	P	279.43	0.49	0.00	P	134.93	MGC955	1p34.1
203465_at	0.99	0.92	P	816.43	0.49	0.01	P	396.17	MRPL19	2q11.1-q11.2
239196_at	1.00	0.96	P	189.70	0.49	0.02	A	92.27	ANKRD22	10q23.31-q23.32
200789_at	1.00	0.99	P	884.03	0.49	0.00	P	427.40	ECH1	19q13.1
223596_at	0.99	0.95	P,M	54.97	0.49	0.01	P,A	26.53	SLC12A6	15q13-q15
243876_at	0.99	0.95	P	21.87	0.49	0.03	P,A	10.73		
202068_s_at	1.00	0.97	P	1144.17	0.49	0.02	P	554.83	LDLR	19p13.3
227876_at	0.99	0.93	P	147.83	0.49	0.02	P	72.03	KIAA1688	8q24.3
201475_x_at	1.00	0.97	P	1581.67	0.49	0.00	P	765.70	MARS	12q13.2
214220_s_at	1.00	0.97	P	190.37	0.49	0.01	P	92.07	ALMS1	2p13
201899_s_at	1.00	0.98	P	2716.43	0.49	0.00	P	1314.23	UBE2A	xq24-q25
232457_at	0.98	0.91	P	36.10	0.49	0.01	P,A	17.50	KIAA1102	4p14
212780_at	1.00	0.98	P	244.23	0.49	0.01	P	118.57	SOS1	2p22-p21
40446_at	0.99	0.92	P	1152.57	0.49	0.03	P	560.13	PHF1	6p21.3
202893_at	1.00	0.96	P	531.30	0.49	0.01	P	259.10	UNC13B	9p12-p11
219289_at	1.00	0.99	P	455.00	0.49	0.00	P	220.47	FLJ20718	16q12.1
204480_s_at	1.00	0.96	P	729.83	0.49	0.00	P	354.33	C9orf16	9q34.1
209849_s_at	0.99	0.94	P	295.10	0.49	0.02	P	146.03	RAD51C	17q22-q23
201460_at	1.00	0.99	P	666.90	0.49	0.00	P	323.03	MAPKAPK2	1q32
218846_at	1.00	0.98	P	411.00	0.49	0.01	P	199.70	CRSP3	6q22.33-q24.1
1556879_at	0.99	0.93	P	69.00	0.49	0.02	P	34.03		
201543_s_at	1.00	0.97	P	1386.27	0.49	0.00	P	672.57	SARA1	10q22.2
201833_at	1.00	0.99	P	2586.80	0.49	0.00	P	1254.93	HDAC2	6q21
229035_s_at	1.00	0.96	P	221.90	0.49	0.00	P	107.90	DKFZp434G0522	16q24.3
208816_x_at	0.99	0.93	P	1495.67	0.49	0.01	P	723.47	ANXA2P1	4q21-q31
244398_x_at	0.99	0.94	P	89.10	0.49	0.02	P	43.63	MGC27466	1p34.2
227369_at	1.00	0.95	P	454.13	0.49	0.02	P	222.50	PAI-RBP1	1p31-p22
226109_at	1.00	1.00	P	1054.97	0.49	0.01	P	513.50	C21orf91	21q21.1
203017_s_at	0.99	0.95	P	262.77	0.49	0.02	P	129.03	SSX2IP	1p22.3
233595_at	1.00	0.97	P	267.80	0.49	0.02	P	130.83		

226070_at	1.00	0.97	P,A	226.87	0.49	0.03	M,A	111.40	LOC286257	9q34.3
226177_at	1.00	0.97	P	989.27	0.49	0.00	P	482.00	GLTP	12q24.12
227568_at	0.98	0.91	P	159.87	0.49	0.02	P	78.70	HECTD2	10q23.32
229862_x_at	1.00	0.97	P	195.43	0.49	0.00	P	94.93	ZNF499	19q13.43
218570_at	0.99	0.94	P	388.10	0.49	0.02	P	188.83	KBTBD4	11p11.2
222266_at	1.00	0.97	P	397.03	0.49	0.02	P	194.43	C19orf2	19q12
213684_s_at	0.99	0.94	P	89.17	0.49	0.00	P	43.40	LIM	4q22
210582_s_at	0.99	0.94	P	369.80	0.49	0.00	P	181.13	LIMK2	22q12.2
218837_s_at	1.00	0.97	P	290.93	0.49	0.00	M,A	141.53	LOC51619	7p13
242592_at	1.00	0.99	P	68.93	0.50	0.00	P	33.73	DKFZp762F0713	14q22.1
202814_s_at	1.00	0.97	P	639.53	0.50	0.01	P	311.17	HIS1	17q21.31
228328_at	0.99	0.93	P	130.70	0.50	0.02	P	64.13		
228785_at	1.00	0.96	P	748.90	0.50	0.01	P	364.50	ZNF281	1q32.1
212103_at	1.00	0.99	P,M	298.13	0.50	0.01	A	145.40	KPNA6	1p35.1-p34.3
239134_at	0.99	0.93	P	193.00	0.50	0.00	P	93.73		
225053_at	1.00	0.99	P	1763.73	0.50	0.00	P	860.07	CNOT7	8p22-p21.3
226538_at	1.00	0.97	P	717.27	0.50	0.01	P	349.83	MAN2A1	5q21-q22
229337_at	0.99	0.95	P	135.20	0.50	0.00	A	65.83	USP2	11q23.3
213349_at	1.00	0.96	P	202.50	0.50	0.00	P	99.10	KIAA0779	3q21.3
220372_at	1.00	0.96	P,M	38.67	0.50	0.01	P,M	18.93	C21orf55	21q22.11
209911_x_at	0.99	0.93	P	361.17	0.50	0.00	P	177.47	HIST1H2BD	6p21.3
41329_at	0.99	0.93	P	158.77	0.50	0.02	P	77.67	PACE-1	1q23.3
226525_at	0.99	0.92	P	369.80	0.50	0.01	P	180.47		
1555976_s_at	0.99	0.94	P	163.33	0.50	0.00	A	80.17	MRCL3	18p11.31
1555852_at	0.99	0.92	P	56.97	0.50	0.02	M,A	27.87	PSMB8	6p21.3
234394_at	1.00	0.96	P	54.67	0.50	0.02	P	27.10		
222603_at	1.00	0.97	P	941.60	0.50	0.02	P	463.90	KIAA1815	9p24
224390_s_at	0.99	0.94	P,A	79.13	0.50	0.02	A	39.23	RGS8	1q25
225706_at	0.99	0.95	P	218.53	0.50	0.03	P	108.10	GLCCI1	7p22.1
235223_at	1.00	1.00	P	327.77	0.50	0.02	P	161.20	C6orf153	6p21.1
244622_at	1.00	0.97	P	59.53	0.50	0.02	M,A	29.53	C21orf107	21q22.2
221880_s_at	1.00	0.97	P	210.80	0.50	0.01	P	103.23	LOC400451	15q26.1
213658_at	1.00	0.97	P	105.40	0.50	0.02	P	52.00	DKFZp547K1113	15q26.1
212552_at	1.00	0.98	P	1207.87	0.50	0.00	P	593.80	HPCAL1	2p25.1
225853_at	1.00	0.98	P	1013.10	0.50	0.00	P	497.17	GNPNAT1	14q22.1
201502_s_at	0.99	0.94	P	499.20	0.50	0.01	P	245.77	NFKBIA	14q13
235610_at	1.00	0.98	P	88.63	0.50	0.00	P	43.60	LOC91801	11q23.1
215786_at	1.00	0.97	P	103.93	0.50	0.01	P	51.00		
229394_s_at	1.00	0.99	P	804.47	0.50	0.01	P	395.40	GRLF1	19q13.3
209607_x_at	1.00	1.00	P	511.13	0.50	0.00	P	250.83	SULT1A3	16p11.2
244669_at	1.00	0.96	P	321.17	0.50	0.03	P	158.63		6q15
243256_at	0.98	0.91	P	122.30	0.50	0.02	A	60.13	MKNK1	1p34.1
202205_at	0.99	0.93	P	655.50	0.50	0.00	P	323.10	VASP	19q13.2-q13.3
225900_at	1.00	0.99	P,A	166.93	0.50	0.00	A	82.20	SEC15L2	2p13.2
226223_at	0.99	0.94	P	607.23	0.50	0.01	P	299.40	PAWR	12q21
218526_s_at	1.00	0.98	P	395.03	0.50	0.00	P	194.57	RANGNRF	17p13
39582_at	1.00	0.96	P	120.10	0.50	0.03	P	59.57	CYLD	16q12-q13
221860_at	1.00	0.97	P	142.33	0.50	0.03	P,M,A	70.90	HNRPL	19q13.2
226602_s_at	1.00	0.95	P	273.30	0.50	0.01	P	134.37	BCR	22q11
218235_s_at	0.99	0.93	P	1250.10	0.50	0.01	P	617.40	CGI-94	1p34.2
240636_at	0.99	0.93	P	113.50	0.50	0.00	P	55.57		
226385_s_at	1.00	0.98	P	2989.47	0.50	0.01	P	1470.63	C7orf30	7p15.3
