# Supplementary material for: Multi-Platform Whole-Genome Microarray Analyses Refine the Epigenetic Signature of Breast Cancer Metastasis with Gene Expression and Copy Number
Source: PLoS One. 2010 Jan 13;5(1):e8665. doi: 10.1371/journal.pone.0008665 (PMC2801616; doi:10.1371/journal.pone.0008665)
Supplement: Table S6 — Genes Hypermethylated AND Decreased in expression, no change in copy number (Venn region 1). (0.05 MB PDF) [file pone.0008665.s007.pdf]

Supplemental Table 6: Genes Hypermethylated AND Decreased in expression, no change in copy number (Venn region 1)

Affy Probe ID	Expression fold change	Common	Map
215729_s_at	-100.00	VGLL1	xq26.3
223315_at	-60.33	NTN4	12q22-q23
224901_at	-55.92	SCD4	4q21.3
218804_at	-53.52	TMEM16A	11q13.2
205980_s_at	-48.11	ARHGAP8	22q13.31
201820_at	-45.17	KRT5	12q12-q13
223374_s_at	-36.31	B3GALT3	3q25
210059_s_at	-31.97	MAPK13	6p21.31
37117_at	-24.03	ARHGAP8	22q13.31
225664_at	-22.30	COL12A1	6q12-q13
223748_at	-21.78	SLC4A11	20p12
205916_at	-21.25	S100A7	1q21
202489_s_at	-20.77	FXYD3	19q13.13
218309_at	-17.67	CaMKIINalpha	1p36.13
205990_s_at	-15.95	WNT5A	3p21-p14
213308_at	-15.78	SHANK2	11q13.2
231766_s_at	-15.21	COL12A1	6q12-q13
229245_at	-13.73	PEPP3	1q32.1
209894_at	-12.37	LEPR	1p31
204664_at	-12.31	ALPP	2q37
203535_at	-12.16	S100A9	1q21
228249_at	-11.76	LOC119710	11p13
211812_s_at	-11.76	B3GALT3	3q25
223949_at	-11.45	TMPRSS3	21q22.3
33323_r_at	-11.45	SFN	1p35.3
202995_s_at	-10.74	FBLN1	22q13.31
205258_at	-10.60	INHBB	2cen-q13
202552_s_at	-10.42	CRIM1	2p21
203713_s_at	-10.16	LLGL2	17q24-q25
201596_x_at	-10.09	KRT18	12q13
205487_s_at	-9.88	VGLL1	xq26.3
209605_at	-9.75	TST	22q13.1
215125_s_at	-9.56	UGT1A10	2q37
201842_s_at	-9.46	EFEMP1	2p16
202551_s_at	-9.44	CRIM1	2p21
223895_s_at	-9.33	EPN3	17q21.33
235626_at	-9.25	CAMK1D	10p14
209260_at	-9.12	SFN	1p35.3
220177_s_at	-9.05	TMPRSS3	21q22.3
213603_s_at	-8.43	RAC2	22q13.1
229927_at	-8.37	LOC93273	1q32.1
212531_at	-8.35	LCN2	9q34
219580_s_at	-8.28	TMC5	16p13.11
210058_at	-8.02	MAPK13	6p21.31
219797_at	-7.97	MGAT4A	2q12
219532_at	-7.91	ELOVL4	6q14
227342_s_at	-7.79	MYEOV	11q13
222830_at	-7.69	TFCP2L2	2p25.1
208006_at	-7.23	FOXI1	5q34
224724_at	-7.19	SULF2	20q12-q13.2
210026_s_at	-6.96	CARD10	22q13.1
215189_at	-6.92	KRTHB6	12q13
201787_at	-6.91	FBLN1	22q13.31
228302_x_at	-6.60	CaMKIINalpha	1p36.13
218764_at	-6.59	PRKCH	14q22-q23
33322_i_at	-6.44	SFN	1p35.3
243681_at	-6.43	SHANK2	11q13.2
211538_s_at	-6.00		
202812_at	-6.00	GAA	7q25.2-q25.3
218086_at	-5.98	NPDC1	9q34.3
203571_s_at	-5.90	C10orf116	10q23.31
216268_s_at	-5.87	JAG1	0p12.1-p11.23
211725_s_at	-5.60	BID	22q11.1
218928_s_at	-5.55	SLC37A1	21q22.3
206125_s_at	-5.42	KLK8	9q13.3-q13.4
222582_at	-5.37	PRKAG2	7q35-q36
204908_s_at	-5.32	BCL3	9q13.1-q13.2
212706_at	-5.32	RASA4	7q22-q31.1
213425_at	-5.30	WNT5A	3p21-p14
208161_s_at	-5.28	ABCC3	17q22
1554006_a_at	-5.27	LLGL2	17q24-q25
203085_s_at	-5.26	TGFB1	19q13.1
218490_s_at	-5.23	ZNF302	19q13.12
203287_at	-5.21	LAD1	1q25.1-q32.3
208763_s_at	-5.16	DSIPI	xq22.3
223194_s_at	-5.15	C6orf85	6p25.2
219411_at	-5.02	ELMO3	16q22.1
224792_at	-4.87	TNKS1BP1	11q12.1
238017_at	-4.85	RDHE2	8q12.1
216641_s_at	-4.83	LAD1	1q25.1-q32.3
228176_at	-4.81	EDG3	9q22.1-q22.2
233555_s_at	-4.76	SULF2	20q12-q13.2
210261_at	-4.71	KCNK2	1q41
209641_s_at	-4.65	ABCC3	17q22
205093_at	-4.54	PEPP3	1q32.1
238149_at	-4.52		19q13.42
209099_x_at	-4.51	JAG1	0p12.1-p11.23
205593_s_at	-4.46	PDE9A	21q22.3
220959_s_at	-4.19	OBP2A	9q34
225373_at	-4.18	PP2135	10q22.3
225475_at	-4.17	MI-ER1	1p31.3
226246_at	-4.16	KCTD1	18q12.1
219010_at	-4.14	FLJ10901	1q32.1
1557129_a_at	-4.11	CANP	11q12.2
227053_at	-4.04	PACSIN1	6p21.3
201843_s_at	-4.03	EFEMP1	2p16
218019_s_at	-4.01	C21orf97	21q22.3
214240_at	-3.96	GAL	11q13.1
226245_at	-3.89	KCTD1	18q12.1

208596_s_at	-3.83	UGT1A10	2q37
218856_at	-3.82	TNFRSF21	6p21.1-12.2
223435_s_at	-3.75	PCDHA6	5q31
235729_at	-3.74	ZNF514	2q11.2
203149_at	-3.61	PVRL2	9q13.2-q13.4
220945_x_at	-3.60	MANSC1	12p13.2
234192_s_at	-3.57	GKAP1	9q22.1
39966_at	-3.56	CSPG5	3p21.3
224492_s_at	-3.51	ZNF627	19p13.2
214958_s_at	-3.50	EVER1	17q25.3
206276_at	-3.47	LY6D	8q24-qter
207419_s_at	-3.44	RAC2	22q13.1
229534_at	-3.43	PTE2B	14q24.2
1554179_s_at	-3.43	LYNX1	8q24.3
218263_s_at	-3.38	LOC58486	11p15.3
227429_at	-3.31	MGC45840	11p15.5
235165_at	-3.29	PARD6B	20q13.13
207802_at	-3.27	CRISP3	6p12.3
210674_s_at	-3.26	PCDHAC2	5q31
201681_s_at	-3.24	DLG5	10q23
206827_s_at	-3.21	TRPV6	7q33-q34
228393_s_at	-3.20	ZNF302	19q13.12
204328_at	-3.17	EVER1	17q25.3
210069_at	-3.16	CPT1B	22q13.33
220246_at	-3.13	CAMK1D	10p14
242871_at	-3.10	MPRG	15q22.31
225354_s_at	-3.08	SH3BGRL2	6q13-15
205265_s_at	-3.04	APEG1	2q36.1
209502_s_at	-3.03	BAIAP2	17q25
214697_s_at	-3.03	ROD1	9q33.1
221614_s_at	-2.98	RPH3AL	17p13.3
209751_s_at	-2.98	SEDLP	19q13.4
207126_x_at	-2.96	UGT1A10	2q37
215299_x_at	-2.95	SULT1A1	16p12.1
235515_at	-2.95	FLJ36445	19q13.13
204379_s_at	-2.93	FGFR3	4p16.3
205109_s_at	-2.92	ARHGEF4	2q22
223179_at	-2.91	MGC10500	16p12.1
225940_at	-2.90	MGC39820	3p14
215001_s_at	-2.88	GLUL	1q31
208078_s_at	-2.88	TCF8	10p11.2
213307_at	-2.84	SHANK2	11q13.2
202994_s_at	-2.83	FBLN1	22q13.31
220318_at	-2.81	EPN3	17q21.33
204579_at	-2.80	FGFR4	5q35.1-qter
201791_s_at	-2.79	DHCR7	1q13.2-q13.5
212131_at	-2.79	C19orf13	19q13.12
205807_s_at	-2.77	TUFT1	1q21
212443_at	-2.77	KIAA0540	3p21.31
210538_s_at	-2.77	BIRC3	11q22
218292_s_at	-2.76	PRKAG2	7q35-q36
224212_s_at	-2.72	PCDHA6	5q31
208373_s_at	-2.71	P2RY6	11q13.5
204532_x_at	-2.68	UGT1A6	2q37
202712_s_at	-2.68	CKMT1	15q15
216470_x_at	-2.66	PRSS3	9p11.2
232422_at	-2.64	LOC87769	13q32.3
1555420_a_at	-2.63	KLF7	2q32
204589_at	-2.62	ARK5	12q24.11
1555105_a_at	-2.61	MI-ER1	1p31.3
208998_at	-2.58	UCP2	11q13
204686_at	-2.58	IRS1	2q36
233748_x_at	-2.56	PRKAG2	7q35-q36
203615_x_at	-2.55	SULT1A1	16p12.1
220144_s_at	-2.54	ANKRD5	20pter-q11.23
207722_s_at	-2.54	BTBD2	19p13.3
212657_s_at	-2.52	IL1RN	2q14.2
225165_at	-2.47	PPP1R1B	17q21.2
218170_at	-2.44	CGI-111	5q22.1-q33.3
205632_s_at	-2.42	PIP5K1B	9q13
214733_s_at	-2.41	DJ167A19.1	1p33-p32.1
1553611_s_at	-2.41	FLJ33790	11q13.3
229404_at	-2.40	TWIST2	2q37.3
209459_s_at	-2.39	ABAT	16p13.2
239853_at	-2.39	KLC2L	19q13
213526_s_at	-2.38	F25965	19q13.1
204517_at	-2.36	PPIC	5q23.2
211975_at	-2.36	ZNF289	1p11.2-p11.12
203317_at	-2.35	TIC	2q13
208997_s_at	-2.30	UCP2	11q13
218644_at	-2.29	PLEK2	14q24.1
204862_s_at	-2.28	NME3	16q13
223411_at	-2.28	AD023	17q25.2
206094_x_at	-2.27	UGT1A6	2q37
203620_s_at	-2.26	FCHSD2	11q13.3
39650_s_at	-2.25	FLJ11383	1q42.2
34726_at	-2.25	CACNB3	12q13
222810_s_at	-2.24	RASAL2	1q24
202365_at	-2.24	MGC5139	12q24.31
46256_at	-2.23	SSB3	16p13.3
32402_s_at	-2.22	SYMPK	19q13.3
209125_at	-2.20	KRT6A	12q12-q13
230120_s_at	-2.19	PLGL	2p11-q11
233550_s_at	-2.19	SLC4A11	20p12
226408_at	-2.19	TEAD2	19q13.3
223948_s_at	-2.19	TMPRSS3	21q22.3
211237_s_at	-2.19	FGFR4	5q35.1-qter
215084_s_at	-2.18	MGC8974	1p33-p32.1
208009_s_at	-2.18	ARHGEF16	1p36.3
202357_s_at	-2.17	BF	6p21.3
204334_at	-2.17	KLF7	2q32
214580_x_at	-2.17	KRT6A	12q12-q13
223628_at	-2.14	DKFZp434N035	22q11.21

223665_at	-2.14	ARPM1	3q26.31
1555935_s_at	-2.13	HUNK	21q22.1
219076_s_at	-2.12	PXMP2	12q24.33
218166_s_at	-2.12	HBXAP	11q13.4
243256_at	-2.11	MKNK1	1p34.1
223500_at	-2.10	CPLX1	4p16.3
206516_at	-2.09	AMH	19p13.3
239730_at	-2.08		
221599_at	-2.08	PTD015	11q13.4
212556_at	-2.08	SCRIB	8q24.3
213540_at	-2.06	HSD17B8	6p21.3
1552319_a_at	-2.03	KLK8	9q13.3-q13.4
225898_at	-2.02	FLJ12953	2p13.1
222640_at	-2.01	DNMT3A	2p23
207517_at	-2.01	LAMC2	1q25-q31
227759_at	-2.01	PCSK9	1p32.3
1557128_at	-2.00	CANP	11q12.2
209110_s_at	-2.00	RAB2L	6p21.3
205871_at	-1.98	PLGL	2p11-q11
229312_s_at	-1.93	GKAP1	9q22.1
219749_at	-1.93	SH2D4A	8p21.2
210070_s_at	-1.86	CPT1B	22q13.33
202267_at	-1.84	LAMC2	1q25-q31
