# Supplementary material for: Multi-Platform Whole-Genome Microarray Analyses Refine the Epigenetic Signature of Breast Cancer Metastasis with Gene Expression and Copy Number
Source: PLoS One. 2010 Jan 13;5(1):e8665. doi: 10.1371/journal.pone.0008665 (PMC2801616; doi:10.1371/journal.pone.0008665)
Supplement: Table S7 — Genes Hypermethylated AND Decreased in expression, loss in copy number (Venn region 2). (0.07 MB PDF) [file pone.0008665.s008.pdf]

Supplemental Table 7: Genes Hypermethylated AND Decreased in expression, loss in copy number (Venn region 2)

Probe set ID	468GFP-LN/468GFP Fold Decrease	Common	Description
231856_at	-100.00	KIAA1244	KIAA1244
225175_s_at	-100.00	CTL2	CTL2 gene
224609_at	-100.00	CTL2	CTL2 gene
219850_s_at	-100.00	EHF	ets homologous factor
217764_s_at	-100.00	RAB31	RAB31, member RAS oncogene family
217763_s_at	-100.00	RAB31	RAB31, member RAS oncogene family
217762_s_at	-100.00	RAB31	RAB31, member RAS oncogene family
215172_at	-100.00	DKFZP566K0524	DKFZP566K0524 protein
210095_s_at	-100.00	IGFBP3	insulin-like growth factor binding protein 3
209602_s_at	-100.00	GATA3	GATA binding protein 3
208091_s_at	-100.00	DKFZP564K0822	hypothetical protein DKFZp564K0822
203028_s_at	-100.00	CYBA	cytochrome b-245, alpha polypeptide
201983_s_at	-100.00	EGFR	epidermal growth factor receptor (erythroblastic leukemia viral (v-erb-b) oncogene homolog, avian)
200606_at	-100.00	DSP	desmoplakin
206067_s_at	-95.54	WT1	Wilms tumor 1
216905_s_at	-94.77	ST14	Human SNC19 mRNA sequence.
221645_s_at	-84.87	ZNF83	zinc finger protein 83 (HPF1)
212143_s_at	-81.91	IGFBP3	insulin-like growth factor binding protein 3
220289_s_at	-74.94	AIM1L	absent in melanoma 1-like
206400_at	-72.74	LGALS7	lectin, galactoside-binding, soluble, 7 (galectin 7)
218162_at	-61.48	OLFML3	HNOEL-iso protein
208165_s_at	-58.98	PRSS16	protease, serine, 16 (thymus)
228600_x_at	-58.44	MGC72075	hypothetical protein MGC72075
237493_at	-57.47	IL22RA2	ht73g10.x1 NCI_CGAP_Lu24 Homo sapiens cDNA clone IMAGE:3152418 3', mRNA sequence.
225016_at	-56.31	APCDD1	adenomatosis polyposis coli down-regulated 1
206142_at	-54.34	ZNF135	zinc finger protein 135 (clone pHZ-17)
209173_at	-51.84	AGR2	anterior gradient 2 homolog (Xenopus laevis)
1560201_at	-51.56	FLJ39963	hypothetical protein FLJ39963
202177_at	-49.41	GAS6	growth arrest-specific 6
232361_s_at	-48.95	EHF	ets homologous factor
219429_at	-48.32	FA2H	fatty acid 2-hydroxylase
204730_at	-47.83	RIMS3	
201984_s_at	-46.93	EGFR	epidermal growth factor receptor (erythroblastic leukemia viral (v-erb-b) oncogene homolog, avian)
205547_s_at	-46.72	TAGLN	transgelin
211341_at	-44.44	POU4F1	POU domain, class 4, transcription factor 1
1554897_s_at	-44.44	RHBDL2	rhomboid, veinlet-like 2 (Drosophila)
215537_x_at	-43.89	DDAH2	
224189_x_at	-43.69	EHF	ets homologous factor
211607_x_at	-40.44	EGFR	epidermal growth factor receptor (erythroblastic leukemia viral (v-erb-b) oncogene homolog, avian)
218960_at	-40.07	TMPRSS4	
202005_at	-39.02	ST14	suppression of tumorigenicity 14 (colon carcinoma, matriptase, epithin)
204855_at	-37.53	SERPINB5	serine (or cysteine) proteinase inhibitor, clade B (ovalbumin), member 5
1555724_s_at	-36.16	TAGLN	transgelin
203421_at	-34.50	TP53I11	tumor protein p53 inducible protein 11
209270_at	-34.17	LAMB3	laminin, beta 3
214667_s_at	-33.35	TP53I11	tumor protein p53 inducible protein 11
204083_s_at	-30.66	TPM2	tropomyosin 2 (beta)
209538_at	-30.30	ZNF32	zinc finger protein 32 (KOX 30)
209691_s_at	-29.80	DOK4	docking protein 4
205048_s_at	-29.21	PSPH	
204179_at	-28.28	MB	myoglobin
1554544_a_at	-27.45	MBP	CDNA clone MGC:70813 IMAGE:6060520, complete cds
1554246_at	-27.37	MGC52423	hypothetical protein MGC52423
222240_s_at	-24.71	ISYNA1	myo-inositol 1-phosphate synthase A1
206382_s_at	-23.54	BDNF	brain-derived neurotrophic factor
236337_at	-22.88	LOC221711	hypothetical protein LOC221711
201510_at	-22.62	ELF3	E74-like factor 3 (ets domain transcription factor, epithelial-specific)
217627_at	-22.50	ZNF573	zinc finger protein 573
209603_at	-22.37	GATA3	GATA binding protein 3
1552427_at	-21.99	ZNF485	zinc finger protein 485
209604_s_at	-21.75	GATA3	GATA binding protein 3
1552502_s_at	-21.49	RHBDL2	rhomboid, veinlet-like 2 (Drosophila)
216052_x_at	-21.40	ARTN	
218921_at	-20.95	SIGIRR	single Ig IL-1R-related molecule
238805_at	-20.24	MGC14839	similar to RIKEN cDNA 2310030G06 gene
218810_at	-20.12	FLJ23231	hypothetical protein FLJ23231
216468_s_at	-19.66	LOC91120	
219522_at	-19.31	FJX1	four jointed box 1 (Drosophila)
222561_at	-19.14	LANCL2	LanC lantibiotic synthetase component C-like 2 (bacterial)
220027_s_at	-18.78	RASIP1	Ras-interacting protein
209873_s_at	-18.41	PKP3	plakophilin 3
205646_s_at	-18.00	PAX6	paired box gene 6 (aniridia, keratitis)
212654_at	-17.24	TPM2	tropomyosin 2 (beta)
216953_s_at	-16.92	WT1	Wilms tumor 1
204019_s_at	-16.84	SH3YL1	SH3 domain containing, Ysc84-like 1 (S. cerevisiae)
206723_s_at	-16.22	EDG4	endothelial differentiation, lysophosphatidic acid G-protein-coupled receptor, 4
218219_s_at	-16.09	LANCL2	LanC lantibiotic synthetase component C-like 2 (bacterial)
206876_at	-15.38		match: proteins: Sw:P81133 Sw:Q61045; Human DNA sequence from clone RP3-399E4 on chromosome 6.
207291_at	-15.37	TMG4	transmembrane gamma-carboxyglutamic acid protein 4
220362_at	-14.81	PSORS1C1	psoriasis susceptibility 1 candidate 1
214909_s_at	-14.48	DDAH2	dimethylarginine dimethylaminohydrolase 2
52940_at	-14.23	SIGIRR	single Ig IL-1R-related molecule
201631_s_at	-13.49	IER3	immediate early response 3
222774_s_at	-13.45	NETO2	neuropilin (NRP) and tolloid (TLL)-like 2
205514_at	-13.41	ZNF415	zinc finger protein 415
204401_at	-12.99	KCNN4	potassium intermediate/small conductance calcium-activated channel, subfamily N, member 4
221646_s_at	-12.96	ZNF399; FLJ13153	Homo sapiens GL013 mRNA, complete cds.
203687_at	-12.91	CX3CL1	chemokine (C-X3-C motif) ligand 1
206722_s_at	-12.41	EDG4	endothelial differentiation, lysophosphatidic acid G-protein-coupled receptor, 4
218888_s_at	-12.39	NETO2	neuropilin (NRP) and tolloid (TLL)-like 2
210827_s_at	-12.31	ELF3	E74-like factor 3 (ets domain transcription factor, epithelial-specific)
203126_at	-12.29	IMPA2	inositol(myo)-1(or 4)-monophosphatase 2
201242_s_at	-11.89	ATP1B1	ATPase, Na+/K+ transporting, beta 1 polypeptide
203365_s_at	-11.71	MMP15	matrix metalloproteinase 15 (membrane-inserted)
219567_s_at	-11.66	FLJ21144	hypothetical protein FLJ21144
203920_at	-11.20	NR1H3	nuclear receptor subfamily 1, group H, member 3

212859_x_at	-11.16	MT1E	metallothionein 2A
219266_at	-10.99	ZNF350	zinc finger protein 350
221081_s_at	-10.99	FLJ22457	hypothetical protein FLJ22457
210237_at	-10.69	ARTN	artemin
201462_at	-10.65	SCRN1	secernin 1
223659_at	-10.14	MSP	mosaic serine protease
223642_at	-10.09	ZIC2	Zic family member 2 (odd-paired homolog, Drosophila)
221655_x_at	-9.92	EPS8L1	EPS8-like 1
214734_at	-9.76	SLAC2-B	SLAC2-B
1552477_a_at	-9.57	IRF6	interferon regulatory factor 6
223432_at	-9.24	OSBP2	oxysterol binding protein 2
91826_at	-8.76	EPS8L1	EPS8-like 1
202262_x_at	-8.72	DDAH2	dimethylarginine dimethylaminohydrolase 2
225129_at	-8.70	CPNE2	copine II
203741_s_at	-8.68	ADCY7	adenylate cyclase 7
201243_s_at	-8.36	ATP1B1	ATPase, Na+/K+ transporting, beta 1 polypeptide
218779_x_at	-8.36	EPS8L1	EPS8-like 1
209357_at	-8.14	CITED2	Cbp/p300-interacting transactivator, with Glu/Asp-rich carboxy-terminal domain, 2
203585_at	-8.14	ZNF185	zinc finger protein 185 (LIM domain)
209872_s_at	-8.01	PKP3	plakophilin 3
221665_s_at	-7.83	EPS8L1	EPS8-like 1
226650_at	-7.82	LOC90637	hypothetical protein LOC90637
228517_at	-7.74	FLJ11730	hypothetical protein FLJ11730
207980_s_at	-7.69	CITED2	Cbp/p300-interacting transactivator, with Glu/Asp-rich carboxy-terminal domain, 2
204656_at	-7.68	MCART1	
222902_s_at	-7.49	FLJ21144	hypothetical protein FLJ21144
205194_at	-7.35	PSPH	phosphoserine phosphatase
1569107_s_at	-7.32	FLJ16030	FLJ16030 protein
203027_s_at	-7.06	MVD	mevalonate (diphospho) decarboxylase
204681_s_at	-6.98	RAPGEF5	Rap guanine nucleotide exchange factor (GEF) 5
218982_s_at	-6.96	MRPS17	mitochondrial ribosomal protein S17
235953_at	-6.88	ZNF610	hypothetical protein FLJ36040
225406_at	-6.69	TWSG1	twisted gastrulation homolog 1 (Drosophila)
210136_at	-6.56		myelin basic protein
205437_at	-6.29	ZNF211	zinc finger protein 211
210239_at	-6.28	IRX5	iroquois homeobox protein 5
219354_at	-6.22	FLJ11078	hypothetical protein FLJ11078
204928_s_at	-6.19	SLC10A3	solute carrier family 10 (sodium/bile acid cotransporter family), member 3
205020_s_at	-6.09	ARL4A	ADP-ribosylation factor-like 4
212056_at	-5.92	KIAA0182	KIAA0182 protein
227045_at	-5.75	ZNF614	FLJ21941 protein
212057_at	-5.71	KIAA0182	zq55f01.s1 Stratagene neuroepithelium (#937231) Homo sapiens cDNA clone IMAGE:645529 3', mRNA sequence.
203359_s_at	-5.67	MYCBP	c-myc binding protein
204106_at	-5.61	TESK1	testis-specific kinase 1
202743_at	-5.61	PIK3R3	601440792T1 NIH_MGC_72 Homo sapiens cDNA clone IMAGE:3915695 3', mRNA sequence.
219622_at	-5.51	RAB20	RAB20, member RAS oncogene family
201594_s_at	-5.48	PPP4R1	protein phosphatase 4, regulatory subunit 1
221843_s_at	-5.36	KIAA1609	KIAA1609 protein
204029_at	-5.26	CELSR2	cadherin, EGF LAG seven-pass G-type receptor 2 (flamingo homolog, Drosophila)
202727_s_at	-5.26	IFNGR1	interferon gamma receptor 1
220419_s_at	-5.26	USP25	ubiquitin specific protease 25
238054_at	-5.19	ADPRHL1	ADP-ribosylhydrolase like 1
204131_s_at	-5.16	FOXO3A	yx83c03.s1 Soares melanocyte 2NbHM Homo sapiens cDNA clone IMAGE:268324 3', mRNA sequence.
203564_at	-5.09	FANCG	Fanconi anemia, complementation group G
204773_at	-4.99	IL11RA	interleukin 11 receptor, alpha
1555486_a_at	-4.99	FLJ14213	hypothetical protein FLJ14213
36499_at	-4.98	CELSR2	cadherin, EGF LAG seven-pass G-type receptor 2 (flamingo homolog, Drosophila)
219201_s_at	-4.96	TWSG1	twisted gastrulation homolog 1 (Drosophila)
213358_at	-4.83	KIAA0802	KIAA0802 protein
222234_s_at	-4.83	MGC3101	hypothetical protein MGC3101
230281_at	-4.76	FLJ32702	hypothetical protein FLJ32702
216336_x_at	-4.74	MT1E	
1553695_a_at	-4.72	NOD9	NOD9 protein
219383_at	-4.72	FLJ14213	hypothetical protein FLJ14213
207675_x_at	-4.70	ARTN	artemin
218432_at	-4.62	FBXO3	F-box only protein 3
1552283_s_at	-4.60	ZDHHC11	zinc finger, DHHC domain containing 11
203509_at	-4.59	SORL1	sortilin-related receptor, L(DLR class) A repeats-containing
823_at	-4.59	CX3CL1	chemokine (C-X3-C motif) ligand 1
202844_s_at	-4.58	RALBP1	ralA binding protein 1
203484_at	-4.52	SEC61G	Sec61 gamma subunit
217759_at	-4.50	TRIM44	tripartite motif-containing 44
210652_s_at	-4.48	C1orf34	chromosome 1 open reading frame 34
228065_at	-4.47	BCL9L	B-cell CLL/lymphoma 9-like
239492_at	-4.45	SEC14L4	SEC14-like 4 (S. cerevisiae)
239007_at	-4.43	ZNF616	602323789F1 NIH_MGC_89 Homo sapiens cDNA clone IMAGE:4426679 5', mRNA sequence.
217165_x_at	-4.39	MT2A	human metallothionein-If; Human metallothionein-If gene (hMT-If).
204657_s_at	-4.35	SHB	SHB (Src homology 2 domain containing) adaptor protein B
213629_x_at	-4.32	MT1F	metallothionein 1F (functional)
234963_s_at	-4.22	FA2H	Homo sapiens Chromosome 16 BAC clone CIT987SK-A-233A8, complete sequence.
218854_at	-4.18	SART2	squamous cell carcinoma antigen recognized by T cells 2
222392_x_at	-4.05	PERP	PERP, TP53 apoptosis effector
227215_at	-4.02	GOPC	golgi associated PDZ and coiled-coil motif containing
205042_at	-3.94	GNE	glucosamine (UDP-N-acetyl)-2-epimerase/N-acetylmannosamine kinase
203360_s_at	-3.88	MYCBP	c-myc binding protein
218744_s_at	-3.85	PACSIN3	protein kinase C and casein kinase substrate in neurons 3
217760_at	-3.84	TRIM44	tripartite motif-containing 44
212268_at	-3.78	SERPINB1	serine (or cysteine) proteinase inhibitor, clade B (ovalbumin), member 1
202031_s_at	-3.72	DKFZP434J154	DKFZP434J154 protein
206770_s_at	-3.71	SLC35A3	solute carrier family 35 (UDP-N-acetylglucosamine (UDP-GlcNAc) transporter), member A3
226127_at	-3.57	DEPC-1	prostate cancer antigen-1
202554_s_at	-3.55	GSTM3	glutathione S-transferase M3 (brain)
241869_at	-3.54	APOL6	apolipoprotein L, 6
205781_at	-3.46	C16orf7	chromosome 16 open reading frame 7
226287_at	-3.46	LOC81023	NY-REN-41 antigen
227718_at	-3.44	PURB	purine-rich element binding protein B
211593_s_at	-3.43	MAST2	microtubule associated serine/threonine kinase 2
225988_at	-3.43	HERC4	DKFZP564G092 protein
219001_s_at	-3.39	MGC10765	hypothetical protein MGC10765
234921_at	-3.39		
202845_s_at	-3.38	RALBP1	ralA binding protein 1
226957_x_at	-3.36	RALBP1	ralA binding protein 1
202738_s_at	-3.33	PHKB	phosphorylase kinase, beta

204613_at	-3.33	PLCG2	phospholipase C, gamma 2 (phosphatidylinositol-specific)
220127_s_at	-3.30	FBXL12	F-box and leucine-rich repeat protein 12
52285_f_at	-3.29	C18orf9	chromosome 18 open reading frame 9
213572_s_at	-3.29	SERPINB1	serine (or cysteine) proteinase inhibitor, clade B (ovalbumin), member 1
203067_at	-3.27	PDHX	pyruvate dehydrogenase complex, component X
204264_at	-3.25	CPT2	carnitine palmitoyltransferase II
223167_s_at	-3.24	USP25	ubiquitin specific protease 25
209865_at	-3.23	SLC35A3	solute carrier family 35 (UDP-N-acetylglucosamine (UDP-GlcNAc) transporter), member A3
220150_s_at	-3.23	C6orf60	chromosome 6 open reading frame 60
1552628_a_at	-3.21	FLJ22313	hypothetical protein FLJ22313
218735_s_at	-3.20	ZNF544	zinc finger protein 544
1598_g_at	-3.20	GAS6	growth arrest-specific 6
210502_s_at	-3.19	PPIE	peptidylprolyl isomerase E (cyclophilin E)
213137_s_at	-3.17	PTPN2	protein tyrosine phosphatase, non-receptor type 2
235349_at	-3.16	FLJ32954	hypothetical protein FLJ32954
65438_at	-3.15	KIAA1609	KIAA1609 protein
209432_s_at	-3.15	CREB3	cAMP responsive element binding protein 3
206461_x_at	-3.14	MT1H	metallothionein 1H
213136_at	-3.13	PTPN2	protein tyrosine phosphatase, non-receptor type 2
228652_at	-3.10	FLJ38288	ql51f03.x1 Soares_NhHMPu_S1 Homo sapiens cDNA clone IMAGE:1875869 3' similar to contains element MER29 repetitive element
223425_at	-3.08	RAVER1	
224992_s_at	-3.08	CMIP	c-Maf-inducing protein
227255_at	-3.07	LOC149420	wf14h01.x1 Soares_NFL_T_GBC_S1 Homo sapiens cDNA clone IMAGE:2350609 3', mRNA sequence.
224415_s_at	-3.06	HINT2	histidine triad nucleotide binding protein 2
227616_at	-3.05	BCL9L	B-cell CLL/lymphoma 9-like
238773_at	-3.03	FLJ33979	hypothetical protein FLJ33979
219311_at	-3.03	C18orf9	chromosome 18 open reading frame 9
203274_at	-3.02	F8A	coagulation factor VIII-associated (intronic transcript)
215903_s_at	-3.00	MAST2	microtubule associated serine/threonine kinase 2
201326_at	-3.00	CCT6A	601304610F1 NIH_MGC_39 Homo sapiens cDNA clone IMAGE:3639098 5', mRNA sequence.
222804_x_at	-2.99	MGC10765	hypothetical protein MGC10765
211721_s_at	-2.96	ZNF551	zinc finger protein 551
202927_at	-2.93	PIN1	ubiquitin-like 5
225002_s_at	-2.92	SUMF2	ht48a11.x1 NCI_CGAP_Mel15 Homo sapiens cDNA clone IMAGE:3149948 3' similar to contains Alu;contains MER35 repetitive element
201327_s_at	-2.88	CCT6A	chaperonin containing TCP1, subunit 6A (zeta 1)
202494_at	-2.88	PPIE	peptidylprolyl isomerase E (cyclophilin E)
208581_x_at	-2.87	MT1X	metallothionein 1X
218011_at	-2.87	UBL5	ubiquitin-like 5
221776_s_at	-2.78	BRD7	bromodomain containing 7
212463_at	-2.77	CD59	CD59 antigen p18-20 (antigen identified by monoclonal antibodies 16.3A5, EJ16, EJ30, EL32 and G344)
204282_s_at	-2.76	FARS1	phenylalanine-tRNA synthetase 1 (mitochondrial)
202049_s_at	-2.76	ZNF262	zinc finger protein 262
219680_at	-2.75	NOD9	NOD9 protein
202883_s_at	-2.72	PPP2R1B	yd71a11.s1 Soares fetal liver spleen 1NFLS Homo sapiens cDNA clone IMAGE:113660 3', mRNA sequence.
219854_at	-2.71	ZNF14	zinc finger protein 14 (KOX 6)
42361_g_at	-2.70	C6orf18	chromosome 6 open reading frame 18
219453_at	-2.70	C16orf44	chromosome 16 open reading frame 44
203266_s_at	-2.68	MAP2K4	mitogen-activated protein kinase kinase 4
201816_s_at	-2.67	GBAS	glioblastoma amplified sequence
218522_s_at	-2.66	BPY2IP1	VCY2 interacting protein 1
204798_at	-2.65	MYB	v-myb myeloblastosis viral oncogene homolog (avian)
204326_x_at	-2.63	MT1X	
222751_at	-2.62	FLJ22313	hypothetical protein FLJ22313
202414_at	-2.62	ERCC5	excision repair cross-complementing rodent repair deficiency, comp. group 5 (xeroderma pigmentosum, compgroup G (Cockayne)
221059_s_at	-2.60	CHST6	carbohydrate (N-acetylglucosamine 6-O) sulfotransferase 6
40569_at	-2.60	ZNF42	Human zinc finger protein 42 (MZF-1) mRNA, complete cds.
213034_at	-2.57	KIAA0999	KIAA0999 protein
224991_at	-2.54	CMIP	c-Maf-inducing protein
202933_s_at	-2.52	YES1	v-yes-1 Yamaguchi sarcoma viral oncogene homolog 1
224852_at	-2.48	TTC17	tetratricopeptide repeat domain 17
212641_at	-2.48	HIVEP2	Human DNA sequence from clone RP1-67K17 on chromosome 6q24.1-24.3, complete sequence.
215068_s_at	-2.47	FLJ11467	hypothetical protein FLJ11467
205546_s_at	-2.46	TYK2	tyrosine kinase 2
202511_s_at	-2.46	APG5L	APG5 autophagy 5-like (S. cerevisiae)
219543_at	-2.45	MAWBP	MAWD binding protein
201948_at	-2.45	HUMAUANTIG	nucleolar GTPase
221208_s_at	-2.40	FLJ23342	hypothetical protein FLJ23342
1563497_at	-2.40	USP25	ubiquitin specific protease 25
205263_at	-2.39	BCL10	B-cell CLL/lymphoma 10
37425_g_at	-2.37	C6orf18	a-helix coiled-coil rod homologue; Homo sapiens HCR (a-helix coiled-coil rod homologue) gene, complete cds.
225198_at	-2.37	VAPA	VAMP (vesicle-associated membrane protein)-associated protein A, 33kDa
211580_s_at	-2.37	PIK3R3	phosphoinositide-3-kinase, regulatory subunit, polypeptide 3 (p55, gamma)
211711_s_at	-2.33	PTEN	phosphatase and tensin homolog (mutated in multiple advanced cancers 1)
226261_at	-2.30	ZNRF2	zinc finger/RING finger 2
204142_at	-2.30	HSRTSBETA	rTS beta protein
204710_s_at	-2.30	DKFZP434J154	DKFZP434J154 protein
232323_s_at	-2.27	TTC17	tetratricopeptide repeat domain 17
218528_s_at	-2.27	RNF38	ring finger protein 38
219826_at	-2.26	FLJ23233	hypothetical protein FLJ23233
218165_at	-2.25	FLJ11730	hypothetical protein FLJ11730
225120_at	-2.24	PURB	CDNA FLJ33107 fis, clone TRACH2000959
207753_at	-2.23	ZNF304	zinc finger protein 304
202439_s_at	-2.22	IDS	iduronate 2-sulfatase (Hunter syndrome)
204937_s_at	-2.21	ZNF274	zinc finger protein 274
212479_s_at	-2.21	FLJ13910	hypothetical protein FLJ13910
219409_at	-2.21	SNIP1	Smad nuclear interacting protein
219307_at	-2.18	C6orf210	chromosome 6 open reading frame 210
224504_s_at	-2.18	MGC13125	hypothetical protein MGC13125
211724_x_at	-2.18	FLJ20323	hypothetical protein FLJ20323
203656_at	-2.16	KIAA0274	KIAA0274
204157_s_at	-2.14	KIAA0999	KIAA0999 protein
204283_at	-2.14	FARS1	phenylalanine-tRNA synthetase 1 (mitochondrial)
204132_s_at	-2.11	FOXO3A	forkhead box O3A
222870_s_at	-2.11	B3GNT1	UDP-GlcNAc:betaGal beta-1,3-N-acetylglucosaminyltransferase 1
225480_at	-2.10	FLJ45459	FLJ45459 protein
1553274_a_at	-2.06	C6orf151	chromosome 6 open reading frame 151
218235_s_at	-2.00	CGI-94	comparative gene identification transcript 94
217804_s_at	-1.97	ILF3	interleukin enhancer binding factor 3, 90kDa
204935_at	-1.95	PTPN2	protein tyrosine phosphatase, non-receptor type 2
202739_s_at	-1.82	PHKB	phosphorylase kinase, beta
243439_at	-1.73		wq87e04.x1 NCI_CGAP_GC6 Homo sapiens cDNA clone IMAGE:2479038 3', mRNA sequence.
