# Supplementary material for: Multi-Platform Whole-Genome Microarray Analyses Refine the Epigenetic Signature of Breast Cancer Metastasis with Gene Expression and Copy Number
Source: PLoS One. 2010 Jan 13;5(1):e8665. doi: 10.1371/journal.pone.0008665 (PMC2801616; doi:10.1371/journal.pone.0008665)
Supplement: Table S8 — Genes Hypomethylated AND Increased in expression, no change in copy number (Venn region 3). (0.06 MB PDF) [file pone.0008665.s009.pdf]

Supplemental Table 8: Genes Hypomethylated AND Increased in expression, no change in copy number (Venn region 3)

Affy Probe ID	fold change	Common	Map	Description
206427_s_at	139.46	MLANA	9p24.1	melan-A
206426_at	84.19	MLANA	9p24.1	melan-A
220534_at	37.14	TRIM48	11q12.1	tripartite motif-containing 48
204973_at	18.10	GJB1	xq13.1	gap junction protein, beta 1, 32kDa (connexin 32, Charcot-Marie-Tooth neuropathy, X-linked)
207397_s_at	17.21	HOXD13	2q31.1	homeo box D13
210762_s_at	12.69	DLC1	8p22	deleted in liver cancer 1
235158_at	12.10	FLJ14803	7q32.3	hypothetical protein FLJ14803
210105_s_at	10.23	FYN	6q21	FYN oncogene related to SRC, FGR, YES
216033_s_at	7.77	FYN	6q21	FYN oncogene related to SRC, FGR, YES
207398_at	7.38	HOXD13	2q31.1	homeo box D13
215966_x_at	6.61	GK	xp21.3	glycerol kinase
219759_at	5.73	LRAP	16	leukocyte-derived arginine aminopeptidase
202784_s_at	5.60	NNT	5p13.1-5cen	nicotinamide nucleotide transhydrogenase
212486_s_at	5.46	FYN	6q21	FYN oncogene related to SRC, FGR, YES
202783_at	5.07	NNT	5p13.1-5cen	nicotinamide nucleotide transhydrogenase
235104_at	4.99	LRAP	16	leukocyte-derived arginine aminopeptidase
221962_s_at	4.91	UBE2H	7q32	ubiquitin-conjugating enzyme E2H (UBC8 homolog, yeast)
224822_at	4.66	DLC1	8p22	deleted in liver cancer 1
230158_at	4.01	FLJ32949	12q14.1	hypothetical protein FLJ32949
220494_s_at	3.72	C14orf43	14q24.2	
203561_at	3.61	FCGR2A	1q23	Fc fragment of IgG, low affinity IIa, receptor for (CD32)
215043_s_at	3.41	LOC153561	5q13.2	Similar to SMA3 protein (LOC391792), mRNA
222420_s_at	3.33	UBE2H	7q32	ubiquitin-conjugating enzyme E2H (UBC8 homolog, yeast)
213397_x_at	3.23	RNASE4	14q11.1	ribonuclease, RNase A family, 4
211791_s_at	3.18	KCNAB2	1p36.3	potassium voltage-gated channel, shaker-related subfamily, beta member 2
219405_at	3.07	RNF137	11p15.4	ring finger protein 137
1552519_at	2.96	ACVR1C	2q24.2	activin A receptor, type IC
238002_at	2.85	GOLPH4	3q26.2	602013186F1 NCI_CGAP_Brn64 Homo sapiens cDNA clone IMAGE:4148900 5', mRNA sequence.
1554273_a_at	2.80	LRAP	16	leukocyte-derived arginine aminopeptidase
217799_x_at	2.78	UBE2H	7q32	ubiquitin-conjugating enzyme E2H (UBC8 homolog, yeast)
1557290_at	2.78			hypothetical protein FLJ36166
215599_at	2.67	SMA4	5q13	SMA3
65585_at	2.65	MGC16279	8p23.1	CDNA FLJ42457 fis, clone BRACE2018700
222267_at	2.60	FLJ14803	7q32.3	601472975F1 NIH_MGC_68 Homo sapiens cDNA clone IMAGE:3875730 5', mRNA sequence.
211514_at	2.50	DustyPK	1q32.1	dusty protein kinase
211515_s_at	2.46	DustyPK	1q32.1	dusty protein kinase
238606_at	2.39	MGC2474	16p11.2	hypothetical protein MGC2474
201231_s_at	2.25	ENO1	1p36.3-p36.2	enolase 1, (alpha)
226043_at	2.22	GPSM1	9q34.3	G-protein signalling modulator 1 (AGS3-like, C. elegans)
221706_s_at	2.21	MDS032	19p13.12	uncharacterized hematopoietic stem/progenitor cells protein MDS032
238784_at	2.19	FLJ32949	12q14.1	hypothetical protein FLJ32949
209535_s_at	2.18	AKAP13	15q24-q25	normal LBC; an alternative 5' translation initiation start site exists upstream of the start site denoted in this sequence; alternative 5' spliced proto-Lbc mRNAs are commonly observed in human skeletal muscle, as well as in other human tissues; Homo sapiens non-ocogenic Rho GTPase-specific GTP exchange factor (proto-LBC) mRNA, complete cds.
202246_s_at	2.17	CDK4	12q14	cyclin-dependent kinase 4
208912_s_at	2.10	CNP	17q21	2',3'-cyclic nucleotide 3' phosphodiesterase
220353_at	2.09	MGC16279	8p23.1	hypothetical protein FLJ10661
202733_at	2.04	P4HA2	5q31	procollagen-proline, 2-oxoglutarate 4-dioxygenase (proline 4-hydroxylase), alpha polypeptide II
219262_at	2.03	SUV39H2	10p13	suppressor of variegation 3-9 homolog 2 (Drosophila)
203452_at	2.00	B3GAT3	11q12.3	beta-1,3-glucuronyltransferase 3 (glucuronosyltransferase I)
212242_at	1.95	TUBA1	2q36.1	tubulin, alpha 1 (testis specific)
1555630_a_at	1.92	RAB34	17q11.2	RAB34, member RAS oncogene family
207614_s_at	1.91	CUL1	7q36.1	cullin 1
207508_at	1.81	ATP5G3	2q31.2	ATP synthase, H+ transporting, mitochondrial F0 complex, subunit c (subunit 9) isoform 3
204579_at	#REF!	FGFR4	5q35.1-qter	fibroblast growth factor receptor 4
201791_s_at	#REF!	DHCR7	11q13.2-q13.5	7-dehydrocholesterol reductase
212131_at	#REF!	C19orf13	19q13.12	chromosome 19 open reading frame 13
205807_s_at	#REF!	TUFT1	1q21	tuftelin 1
212443_at	#REF!	KIAA0540	3p21.31	KIAA0540 protein
210538_s_at	#REF!	BIRC3	11q22	IAP homolog C; interacts with TRAF1 and TRAF2 in yeast two hybrid system; homolog of Baculovirus IAP genes; Mammalian IAP homolog C; Human IAP homolog C (MIHC) mRNA, complete cds.
218292_s_at	#REF!	PRKAG2	7q35-q36	protein kinase, AMP-activated, gamma 2 non-catalytic subunit
224212_s_at	#REF!	PCDHA6	5q31	protocadherin alpha 5
208373_s_at	#REF!	P2RY6	11q13.5	pyrimidinergic receptor P2Y, G-protein coupled, 6
204532_x_at	#REF!	UGT1A6	2q37	UDP glycosyltransferase 1 family, polypeptide A10
202712_s_at	#REF!	CKMT1	15q15	creatine kinase, mitochondrial 1 (ubiquitous)
216470_x_at	#REF!	PRSS3	9p11.2	
232422_at	#REF!	LOC87769	13q32.3	hypothetical protein BC004360
1555420_a_at	#REF!	KLF7	2q32	Kruppel-like factor 7 (ubiquitous)
204589_at	#REF!	ARK5	12q24.11	
1555105_a_at	#REF!	MI-ER1	1p31.3	mesoderm induction early response 1
208998_at	#REF!	UCP2	11q13	uncoupling protein 2 (mitochondrial, proton carrier)
204686_at	#REF!	IRS1	2q36	insulin receptor substrate 1
233748_x_at	#REF!	PRKAG2	7q35-q36	protein kinase, AMP-activated, gamma 2 non-catalytic subunit
203615_x_at	#REF!	SULT1A1	16p12.1	sulfotransferase family, cytosolic, 1A, phenol-preferring, member 1
220144_s_at	#REF!	ANKRD5	20pter-q11.23	ankyrin repeat domain 5
207722_s_at	#REF!	BTBD2	19p13.3	BTB (POZ) domain containing 2
212657_s_at	#REF!	IL1RN	2q14.2	
225165_at	#REF!	PPP1R1B	17q21.2	protein phosphatase 1, regulatory (inhibitor) subunit 1B (dopamine and cAMP regulated phosphoprotein, DARPP-32)
218170_at	#REF!	CGI-111	5q22.1-q33.3	CGI-111 protein
205632_s_at	#REF!	PIP5K1B	9q13	phosphatidylinositol-4-phosphate 5-kinase, type I, beta
214733_s_at	#REF!	DJ167A19.1	1p33-p32.1	Human DNA sequence from clone RP1-167A19 on chromosome 1p32.1-33, complete sequence.
1553611_s_at	#REF!	FLJ33790	11q13.3	hypothetical protein FLJ33790
229404_at	#REF!	TWIST2	2q37.3	twist homolog 2 (Drosophila)
209459_s_at	#REF!	ABAT	16p13.2	4-aminobutyrate aminotransferase
239853_at	#REF!	KLC2L	19q13	kinesin light chain 2-like
213526_s_at	#REF!	F25965	19q13.1	presenilin enhancer 2
204517_at	#REF!	PPIC	5q23.2	601656143R1 NIH_MGC_66 Homo sapiens cDNA clone IMAGE:3855754 3', mRNA sequence.
211975_at	#REF!	ZNF289	11p11.2-p11.12	600944342T1 NIH_MGC_17 Homo sapiens cDNA clone IMAGE:2960218 3', mRNA sequence.
203317_at	#REF!	TIC	2q13	SEC7 homolog
208997_s_at	#REF!	UCP2	11q13	uncoupling protein 2 (mitochondrial, proton carrier)
218644_at	#REF!	PLEK2	14q24.1	pleckstrin 2
204862_s_at	#REF!	NME3	16q13	non-metastatic cells 3, protein expressed in
223411_at	#REF!	AD023	17q25.2	Homo sapiens AD023 mRNA, complete cds.
206094_x_at	#REF!	UGT1A6	2q37	UDP glycosyltransferase 1 family, polypeptide A10
203620_s_at	#REF!	FCHSD2	11q13.3	
39650_s_at	#REF!	FLJ11383	1q42.2	hypothetical protein FLJ11383
34726_at	#REF!	CACNB3	12q13	calcium channel, voltage-dependent, beta 3 subunit
222810_s_at	#REF!	RASAL2	1q24	RAS protein activator like 2
202365_at	#REF!	MGC5139	12q24.31	acyl-Coenzyme A dehydrogenase, C-2 to C-3 short chain
46256_at	#REF!	SSB3	16p13.3	ni39a05.s1 NCI_CGAP_Lu1 Homo sapiens cDNA clone IMAGE:979184 3', mRNA sequence.
32402_s_at	#REF!	SYMPK	19q13.3	symplekin
209125_at	#REF!	KRT6A	12q12-q13	unnamed protein product; keratin; Human messenger fragment encoding cytoskeletal keratin (type II). mRNA from cultured epidermal cells from human foreskin.
230120_s_at	#REF!	PLGL	2p11-q11	Transcribed sequences
233550_s_at	#REF!	SLC4A11	20p12	
226408_at	#REF!	TEAD2	19q13.3	CD37 antigen
223948_s_at	#REF!	TMPRSS3	21q22.3	transmembrane protease, serine 3
211237_s_at	#REF!	FGFR4	5q35.1-qter	fibroblast growth factor receptor 4
215084_s_at	#REF!	MGC8974	1p33-p32.1	Human DNA sequence from clone RP1-167A19 on chromosome 1p32.1-33, complete sequence.
208009_s_at	#REF!	ARHGEF16	1p36.3	Rho guanine exchange factor (GEF) 16
202357_s_at	#REF!	BF	6p21.3	B-factor, properdin
204334_at	#REF!	KLF7	2q32	Kruppel-like factor 7 (ubiquitous)
214580_x_at	#REF!	KRT6A	12q12-q13	keratin 6A
223628_at	#REF!	DKFZp434N035	22q11.21	hypothetical protein DKFZp434N035
223665_at	#REF!	ARPM1	3q26.31	actin related protein M1
1555935_s_at	#REF!	HUNK	21q22.1	hormonally upregulated Neu-associated kinase
219076_s_at	#REF!	PXMP2	12q24.33	peroxisomal membrane protein 2, 22kDa
218166_s_at	#REF!	HBXAP	11q13.4	hepatitis B virus x associated protein
243256_at	#REF!	MKNK1	1p34.1	Transcribed sequence with weak similarity to protein ref:NP_060265.1 (H.sapiens) hypothetical protein FLJ20378 [Homo sapiens]
223500_at	#REF!	CPLX1	4p16.3	complexin 1
206516_at	#REF!	AMH	19p13.3	anti-Mullerian hormone
239730_at	#REF!			Full length insert cDNA clone ZD87H06
221599_at	#REF!	PTD015	11q13.4	PTD015 protein
212556_at	#REF!	SCRIB	8q24.3	scribble

213540_at	#REF!	HSD17B8	6p21.3		
1552319_a_at	#REF!	KLK8	19q13.3-q13.4	kallikrein 8 (neuropsin/ovasin)	
225898_at	#REF!	FLJ12953	2p13.1	hypothetical protein FLJ12953 similar to Mus musculus D3Mm3e	
222640_at	#REF!	DNMT3A	2p23	DNA (cytosine-5-)-methyltransferase 3 alpha	
207517_at	#REF!	LAMC2	1q25-q31	laminin, gamma 2	
227759_at	#REF!	PCSK9	1p32.3	proprotein convertase subtilisin/kexin type 9	
1557128_at	#REF!	CANP	11q12.2	cancer-associated nucleoprotein	
209110_s_at	#REF!	RAB2L	6p21.3	RAB2, member RAS oncogene family-like	
205871_at	#REF!	PLGL	2p11-q11	plasminogen-like	
229312_s_at	#REF!	GKAP1	9q22.1	protein kinase anchoring protein GKAP42	
219749_at	#REF!	SH2D4A	8p21.2	hypothetical protein FLJ20967	
210070_s_at	#REF!	CPT1B	22q13.33	carnitine palmitoyltransferase 1B (muscle)	
202267_at	#REF!	LAMC2	1q25-q31	laminin, gamma 2	
