# Supplementary material for: Multi-Platform Whole-Genome Microarray Analyses Refine the Epigenetic Signature of Breast Cancer Metastasis with Gene Expression and Copy Number
Source: PLoS One. 2010 Jan 13;5(1):e8665. doi: 10.1371/journal.pone.0008665 (PMC2801616; doi:10.1371/journal.pone.0008665)
Supplement: Table S9 — Genes Hypomethylated AND Increased in expression, gain in copy number (Venn region 4). (0.07 MB PDF) [file pone.0008665.s010.pdf]

Supplemental Table 9: Genes Hypomethylated AND Increased in expression, gain in copy number (Venn region 4)

Probe ID	468GFP-LN/468GFP fold increase	Common	Description
203603_s_at	615.83	ZFHX1B	zinc finger homeobox 1b
209170_s_at	595.83	GPM6B	Homo sapiens m6b1 mRNA, complete cds.
206070_s_at	471.97	EPHA3	EphA3
209167_at	270.99	GPM6B	glycoprotein M6B
208986_at	257.20	TCF12	transcription factor 12 (HTF4, helix-loop-helix transcription factors 4)
213139_at	207.57	SNAI2	snail homolog 2 (Drosophila)
206696_at	182.45	GPR143	G protein-coupled receptor 143
218474_s_at	181.35	KCTD5	potassium channel tetramerisation domain containing 5
209392_at	148.48	ENPP2	ectonucleotide pyrophosphatase/phosphodiesterase 2 (autotaxin)
218839_at	82.58	HEY1	hairy/enhancer-of-split related with YRPW motif 1
206071_s_at	77.99	EPHA3	EphA3
225945_at	76.33	VIK	vav-1 interacting Kruppel-like protein
209169_at	74.17	GPM6B	glycoprotein M6B
207253_s_at	62.87	UBN1	ubinuclein 1
1554726_at	50.22	VIK	vav-1 interacting Kruppel-like protein
207808_s_at	42.96	PROS1	protein S (alpha)
202527_s_at	40.09	SMAD4	MAD, mothers against decapentaplegic homolog 4 (Drosophila)
214971_s_at	37.61	SIAT1	sialyltransferase 1 (beta-galactoside alpha-2,6-sialyltransferase)
223723_at	37.47	MFI2	antigen p97 (melanoma associated) identified by monoclonal antibodies 133.2 and 96.5
220425_x_at	35.48	ROPN1	ropporin, rhophilin associated protein 1
210078_s_at	33.26	KCNAB1	potassium voltage-gated channel, shaker-related subfamily, beta member 1
210839_s_at	30.09	ENPP2	ectonucleotide pyrophosphatase/phosphodiesterase 2 (autotaxin)
1555527_at	26.55	COL9A1	collagen, type IX, alpha 1
210619_s_at	25.54	HYAL1	hyaluronoglucosaminidase 1
204860_s_at	24.90	BIRC1	Transcribed sequence with strong similarity to protein sp:Q13075 (H.sapiens) BIR1_HUMAN Baculoviral IAP repeat-containing protein 1
208454_s_at	23.58	PGCP	plasma glutamate carboxypeptidase
224477_s_at	22.53	SDOS	hypothetical protein MGC11275
212576_at	22.39	MGRN1	mahogunin, ring finger 1
206522_at	21.64	MGAM	maltase-glucoamylase (alpha-glucosidase)
221772_s_at	21.03	PPP2R2D	protein phosphatase 2, regulatory subunit B, delta isoform
235359_at	20.99	UNQ3030	ELLP3030
213355_at	20.75	SIAT10	sialyltransferase 10 (alpha-2,3-sialyltransferase VI)
229947_at	20.75		CDNA FLJ26876 fis, clone PRS09003
209168_at	20.65	GPM6B	glycoprotein M6B
210942_s_at	19.26	SIAT10	sialyltransferase 10 (alpha-2,3-sialyltransferase VI)
203348_s_at	19.19	ETV5	ets variant gene 5 (ets-related molecule)
222008_at	18.51	COL9A1	collagen, type IX, alpha 1
235238_at	18.28	RaLP	602086260F1 NIH_MGC_83 Homo sapiens cDNA clone IMAGE:4250534 5', mRNA sequence.
227007_at	16.79	LOC255104	hypothetical protein LOC255104
216375_s_at	15.94	ETV5	ets variant gene 5 (ets-related molecule)
1560292_a_at	15.93	LOC255104	hypothetical protein LOC255104
205227_at	15.46	IL1RAP	interleukin 1 receptor accessory protein
223302_s_at	15.38	VIK	vav-1 interacting Kruppel-like protein
226066_at	14.52	MITF	microphthalmia-associated transcription factor
210471_s_at	14.26	KCNAB1	potassium voltage-gated channel, shaker-related subfamily, beta member 1
225874_at	14.07	LOC124402	601297616F1 NIH_MGC_19 Homo sapiens cDNA clone IMAGE:3627891 5', mRNA sequence.
221628_s_at	13.71	N-PAC	cytokine-like nuclear factor n-pac
44783_s_at	13.30	HEY1	hairy/enhancer-of-split related with YRPW motif 1
220167_s_at	12.46	TP53TG3	TP53TG3 protein
200756_x_at	12.41	CALU	calumenin
222153_at	12.32	MYEF2	myelin expression factor 2
205439_at	12.23	GSTT2	glutathione S-transferase theta 2
204469_at	11.49	PTPRZ1	protein tyrosine phosphatase, receptor-type, Z polypeptide 1
238418_at	11.24	SLC35B4	solute carrier family 35, member B4
209515_s_at	11.23	RAB27A	RAB27A, member RAS oncogene family
209514_s_at	11.21	RAB27A	RAB27A, member RAS oncogene family
211737_x_at	11.19	PTN	pleiotrophin (heparin binding growth factor 8, neurite growth-promoting factor 1)
207233_s_at	11.18	MITF	microphthalmia-associated transcription factor
214845_s_at	10.94	CALU	calumenin
210233_at	10.88	IL1RAP	interleukin 1 receptor accessory protein
240770_at	10.72	LOC134285	hypothetical protein LOC134285
210951_x_at	10.68	RAB27A	RAB27A, member RAS oncogene family
210852_s_at	10.57	AASS	aminoadipate-semialdehyde synthase
203349_s_at	10.39	ETV5	ets variant gene 5 (ets-related molecule)
222652_s_at	9.89	N-PAC	cytokine-like nuclear factor n-pac
205383_s_at	9.85	ZNF288	zinc finger protein 288
212730_at	9.62	DMN	desmuslin
221643_s_at	9.48	RERE	arginine-glutamic acid dipeptide (RE) repeats
237215_s_at	9.28	TFRC	transferrin receptor (p90, CD71)
222851_at	9.15	FLJ10997	DKFZp761B158_r1 761 (synonym: hamy2) Homo sapiens cDNA clone DKFZp761B158 5', mRNA sequence.
208670_s_at	8.97	CRI1	CREBBP/EP300 inhibitory protein 1
209465_x_at	8.83	PTN	pleiotrophin (heparin binding growth factor 8, neurite growth-promoting factor 1)
1554874_at	8.51	MITF	synonym: WS2A; Homo sapiens microphthalmia-associated transcription factor, mRNA (cDNA clone IMAGE:4708731), complete cds.
222645_s_at	8.30	KCTD5	potassium channel tetramerisation domain containing 5
204105_s_at	7.94	NRCAM	neuronal cell adhesion molecule
201337_s_at	7.61	VAMP3	vesicle-associated membrane protein 3 (cellubrevin)
219260_s_at	7.19	DERP6	S-phase 2 protein
203501_at	7.11	PGCP	aminopeptidase; go_component: extracellular space [goid 0005615] [evidence E] [pmid 10206990]; go_component:
225881_at	7.06	SLC35B4	AL513639 Homo sapiens NEUROBLASTOMA Homo sapiens cDNA clone XCL0BB001ZF12 3-PRIME, mRNA sequence.
223865_at	7.04	SOX6	SRY (sex determining region Y)-box 6
1554717_a_at	6.82	PDE4D	phosphodiesterase 4D, cAMP-specific (phosphodiesterase E3 dunce homolog, Drosophila)
215728_s_at	6.67	BACH	
226084_at	6.50	MAP1B	microtubule-associated protein 1B
200755_s_at	6.17	CALU	calumenin
208669_s_at	6.02	CRI1	CREBBP/EP300 inhibitory protein 1
216316_x_at	5.47	GK	H.sapiens glycerol kinase pseudogene, chromosome 1.
219815_at	5.38	GAL3ST4	beta-galactose-3-O-sulfotransferase, 4
240528_s_at	5.32	SEC8L1	SEC8-like 1 (S. cerevisiae)
200939_s_at	5.30	RERE	arginine-glutamic acid dipeptide (RE) repeats
208213_s_at	5.25	KCNAB1	potassium voltage-gated channel, shaker-related subfamily, beta member 1
210647_x_at	5.23	PLA2G6	phospholipase A2, group VI (cytosolic, calcium-independent)
225484_at	5.03	TSGA14	testis specific, 14
208002_s_at	4.97	BACH	brain acyl-CoA hydrolase
200757_s_at	4.71	CALU	calumenin
202545_at	4.69	PRKCD	protein kinase C, delta

211749_s_at	4.45	VAMP3	vesicle-associated membrane protein 3 (cellubrevin)
201998_at	4.45	SIAT1	sialyltransferase 1 (beta-galactoside alpha-2,6-sialyltransferase)
217437_s_at	4.42	TACC1	transforming, acidic coiled-coil containing protein 1
202139_at	4.39	AKR7A2	aldo-keto reductase family 7, member A2 (aflatoxin aldehyde reductase)
1558249_s_at	4.39	STX16	syntaxin 16
223163_s_at	4.33	NIPA	nuclear interacting partner of anaplastic lymphoma kinase (ALK)
223107_s_at	4.33	PS1D	putative S1 RNA binding domain protein
215723_s_at	4.30	PLD1	phospholipase D1, phophatidylcholine-specific
204691_x_at	4.29	PLA2G6	phospholipase A2, group VI (cytosolic, calcium-independent)
202536_at	4.27	DKFZP564O123	DKFZP564O123 protein
217960_s_at	4.20	TOMM22	chromosome 22 open reading frame 2
219703_at	4.18	MNS1	meiosis-specific nuclear structural protein 1
229061_s_at	4.17	SLC25A13	solute carrier family 25, member 13 (citrin)
202570_s_at	4.11	DLGAP4	disks large-associated protein 4
207541_s_at	4.09	PMSCL2	polymyositis/scleroderma autoantigen 2, 100kDa
216205_s_at	4.08	MFN2	mitofusin 2
215938_s_at	4.06	PLA2G6	phospholipase A2, group VI (cytosolic, calcium-independent)
218981_at	4.04	ACN9	ACN9 homolog (S. cerevisiae)
1554449_at	3.96	FLJ35954	hypothetical protein FLJ35954
221935_s_at	3.96	MGC34132	unnamed protein product; Homo sapiens cDNA FLJ13078 fis, clone NT2RP3002002.
219033_at	3.90	FLJ21308	hypothetical protein FLJ21308
211698_at	3.83	CRI1	CREBBP/EP300 inhibitory protein 1
1554690_a_at	3.82	TACC1	transforming, acidic coiled-coil containing protein 1
206907_at	3.82	TNFSF9	tumor necrosis factor (ligand) superfamily, member 9
202675_at	3.82	SDHB	succinate dehydrogenase complex, subunit B, iron sulfur (Ip)
200973_s_at	3.79	TM4SF8	transmembrane 4 superfamily member 8
205084_at	3.76	BCAP29	B-cell receptor-associated protein 29
211599_x_at	3.76	MET	Human (tpr-met fusion) oncogene mRNA, complete cds.
221986_s_at	3.75	DRE1	DRE1 protein
233803_s_at	3.74	MYBBP1A	MYB binding protein (P160) 1a
213807_x_at	3.74	MET	met proto-oncogene (hepatocyte growth factor receptor)
212459_x_at	3.66	SUCLG2	succinate-CoA ligase, GDP-forming, beta subunit
223220_s_at	3.65	BAL	B aggressive lymphoma gene
222206_s_at	3.65	LOC56926	hypothetical protein from EUROIMAGE 2021883
225331_at	3.63	C3orf6	chromosome 3 open reading frame 6
215772_x_at	3.62	SUCLG2	succinate-CoA ligase, GDP-forming, beta subunit
221638_s_at	3.62	STX16	syntaxin 16
225882_at	3.61	SLC35B4	solute carrier family 35, member B4
63825_at	3.59	ABHD2	abhydrolase domain containing 2
217118_s_at	3.58	KIAA0930	KIAA0930 protein
218501_at	3.57	ARHGEF3	Rho guanine nucleotide exchange factor (GEF) 3
209147_s_at	3.55	PPAP2A	phosphatidic acid phosphatase type 2A
221815_at	3.54	ABHD2	7a47d01.x1 NCI_CGAP_GC6 Homo sapiens cDNA clone IMAGE:3221857 3' similar to gb:X12433 PROTEIN PHPS1-2 (HUMAN);, mRNA seq
225485_at	3.52	TSGA14	testis specific, 14
209737_at	3.52	AIP1	atrophin-1 interacting protein 1
220444_at	3.52	ZNF557	zinc finger protein 557
228759_at	3.49	CREB3L2	cAMP responsive element binding protein 3-like 2
205356_at	3.46	USP13	ubiquitin specific protease 13 (isopeptidase T-3)
234726_s_at	3.43	FLJ13576	hypothetical protein FLJ13576
208503_s_at	3.40	ODAG	ocular development-associated gene
204369_at	3.40	PIK3CA	phosphoinositide-3-kinase, catalytic, alpha polypeptide
221985_at	3.39	DRE1	DRE1 protein
226980_at	3.37	XTP1	HBxAg transactivated protein 1
225677_at	3.35	BCAP29	B-cell receptor-associated protein 29
238465_at	3.34	MGC33648	qq90e10.x1 Soares_total_fetus_Nb2HF8_9w Homo sapiens cDNA clone IMAGE:1938666 3', mRNA sequence.
223539_s_at	3.33	SERF1A	small EDRK-rich factor 1A (telomeric)
205071_x_at	3.32	XRCC4	X-ray repair complementing defective repair in Chinese hamster cells 4
223518_at	3.27	DFFA	DNA fragmentation factor, 45kDa, alpha polypeptide
63009_at	3.19	FLJ10539	hypothetical protein FLJ10539
212345_s_at	3.14	CREB3L2	cAMP responsive element binding protein 3-like 2
202566_s_at	3.12	SVIL	supervillin
225674_at	3.11	BCAP29	B-cell receptor-associated protein 29
222678_s_at	3.10	RP42	RP42 homolog
204324_s_at	3.06	GOLPH4	golgi phosphoprotein 4
219083_at	3.06	FLJ10539	hypothetical protein FLJ10539
213018_at	3.05	ODAG	ocular development-associated gene
217043_s_at	3.03	MFN1	mitofusin 1
224790_at	3.02	DDEF1	development and differentiation enhancing factor 1
202538_s_at	3.01	DKFZP564O123	DKFZP564O123 protein
201439_at	3.01	GBF1	golgi-specific brefeldin A resistance factor 1
1554465_s_at	2.99	FLJ20344	hypothetical protein FLJ20344
212178_s_at	2.99	POM121	POM121 membrane glycoprotein (rat)
223457_at	2.96	COPG2	coatomer protein complex, subunit gamma 2
209414_at	2.96	FZR1	Fzr1 protein
202929_s_at	2.93	DDT	D-dopachrome tautomerase
222841_s_at	2.91	TIMM22	translocase of inner mitochondrial membrane 22 homolog (yeast)
234926_s_at	2.91	C20orf43	chromosome 20 open reading frame 43
217737_x_at	2.91	C20orf43	chromosome 20 open reading frame 43
201521_s_at	2.90	NCBP2	nuclear cap binding protein subunit 2, 20kDa
208727_s_at	2.87	CDC42	cell division cycle 42 (GTP binding protein, 25kDa)
206918_s_at	2.87	CPNE1	copine I
214531_s_at	2.86	SNX1	sorting nexin 1
208674_x_at	2.85	DDOST	dolichyl-diphosphooligosaccharide-protein glycosyltransferase
211595_s_at	2.85	MRPS11	mitochondrial ribosomal protein S11
217915_s_at	2.85	C15orf15	chromosome 15 open reading frame 15
1553750_a_at	2.84	MGC33371	hypothetical protein MGC33371
214835_s_at	2.82	SUCLG2	succinate-CoA ligase, GDP-forming, beta subunit
233842_x_at	2.81	C20orf43	chromosome 20 open reading frame 43
200940_s_at	2.80	RERE	arginine-glutamic acid dipeptide (RE) repeats
214259_s_at	2.80	AKR7A2	aldo-keto reductase family 7, member A2 (aflatoxin aldehyde reductase)
201752_s_at	2.79	ADD3	adducin 3 (gamma)
205748_s_at	2.78	RNF126	ring finger protein 126
222572_at	2.78	PPM2C	protein phosphatase 2C, magnesium-dependent, catalytic subunit
201716_at	2.77	SNX1	sorting nexin 1
200911_s_at	2.73	TACC1	transforming, acidic coiled-coil containing protein 1
211801_x_at	2.73	MFN1	mitofusin 1
208728_s_at	2.73	CDC42	cell division cycle 42 (GTP binding protein, 25kDa)
226087_at	2.72	LZIC	leucine zipper and CTNNBIP1 domain containing
220688_s_at	2.72	C1orf33	chromosome 1 open reading frame 33
213816_s_at	2.71	MET	met proto-oncogene (hepatocyte growth factor receptor)
232048_at	2.70	MGC33371	hypothetical protein MGC33371
208675_s_at	2.67	DDOST	dolichyl-diphosphooligosaccharide-protein glycosyltransferase
223108_s_at	2.65	PS1D	putative S1 RNA binding domain protein

208677_s_at	2.62	BSG	basigin (OK blood group)
223286_at	2.62	DERP6	S-phase 2 protein
202537_s_at	2.61	DKFZP564O123	DKFZP564O123 protein
207098_s_at	2.61	MFN1	mitofusin 1
204172_at	2.60	CPOX	coproporphyrinogen oxidase
238609_at	2.59		ya86b01.r1 Stratagene fetal spleen (#937205) Homo sapiens cDNA clone IMAGE:68521 5' similar to contains Alu repetitive element, mRNA seq
209350_s_at	2.59	GPS2	G protein pathway suppressor 2
223538_at	2.58	SERF1A	small EDRK-rich factor 1A (telomeric)
210983_s_at	2.58	MCM7	MCM7 minichromosome maintenance deficient 7 (S. cerevisiae)
220043_s_at	2.57	MFI2	antigen p97 (melanoma associated) identified by monoclonal antibodies 133.2 and 96.5
219239_s_at	2.57	FLJ10997	hypothetical protein FLJ10997
33760_at	2.56	PEX14	peroxisomal biogenesis factor 14
1554456_a_at	2.55	WINS1	WINS1 protein with Drosophila Lines (Lin) homologous domain
202290_at	2.54	PDAP1	PDGFA associated protein 1
212873_at	2.54	HA-1	minor histocompatibility antigen HA-1
215905_s_at	2.54	HPRP8BP	U5 snRNP-specific 40 kDa protein (hPrp8-binding)
218273_s_at	2.54	PPM2C	protein phosphatase 2C, magnesium-dependent, catalytic subunit
203775_at	2.53	SLC25A13	solute carrier family 25, member 13 (citrin)
209811_at	2.53	CASP2	caspase 2, apoptosis-related cysteine protease (neural precursor cell expressed, developmentally down-regulated 2)
200972_at	2.52	TM4SF8	transmembrane 4 superfamily member 8
216088_s_at	2.51	PSMA7	
215931_s_at	2.49	ARFGEF2	ADP-ribosylation factor guanine nucleotide-exchange factor 2 (brefeldin A-inhibited)
203636_at	2.49	MID1	midline 1 (Opitz/BBB syndrome)
212721_at	2.48	SFRS12	splicing factor, arginine/serine-rich 12
209466_x_at	2.48	PTN	pleiotrophin (heparin binding growth factor 8, neurite growth-promoting factor 1)
221039_s_at	2.47	DDEF1	synonyms: PAP, PAG2, ASAP1, ZG14P, KIAA1249; Homo sapiens development and differentiation enhancing factor 1 (DDEF1), mRNA.
222474_s_at	2.47	TOMM22	chromosome 22 open reading frame 2
205882_x_at	2.45	ADD3	adducin 3 (gamma)
213194_at	2.45	ROBO1	roundabout, axon guidance receptor, homolog 1 (Drosophila)
226524_at	2.45	MGC26717	hypothetical protein MGC26717
209080_x_at	2.44	TXNL2	thioredoxin-like 2
242838_at	2.43	FLJ12748	zt16g02.r1 NCI_CGAP_GCB1 Homo sapiens cDNA clone IMAGE:713330 5', mRNA sequence.
203637_s_at	2.43	MID1	midline 1 (Opitz/BBB syndrome)
221041_s_at	2.42	SLC17A5	solute carrier family 17 (anion/sugar transporter), member 5
210813_s_at	2.41	XRCC4	X-ray repair complementing defective repair in Chinese hamster cells 4
222679_s_at	2.40	RP42	RP42 homolog
216267_s_at	2.40	PL6	placental protein 6
223203_at	2.40	PRO0659	PRO0659 protein
203621_at	2.38	NDUFB5	NADH dehydrogenase (ubiquinone) 1 beta subcomplex, 5, 16kDa
200883_at	2.37	UQCRC2	ubiquinol-cytochrome c reductase core protein II
202394_s_at	2.36	ABCF3	ATP-binding cassette, sub-family F (GCN20), member 3
204593_s_at	2.34	FLJ20232	hypothetical protein FLJ20232
201155_s_at	2.34	MFN2	mitofusin 2
202168_at	2.34	TAF9	TAF9 RNA polymerase II, TATA box binding protein (TBP)-associated factor, 32kDa
201336_at	2.33	VAMP3	vesicle-associated membrane protein 3 (cellubrevin)
200929_at	2.33	TMP21	transmembrane trafficking protein
217367_s_at	2.33	ZHX3	zinc fingers and homeoboxes 3
224836_at	2.30	C20orf110	
231912_s_at	2.30	DKFZP434B0335	DKFZP434B0335 protein
218580_x_at	2.30	AKIP	hypothetical protein MGC3047
225612_s_at	2.29	B3GNT5	7d27a05.x1 NCI_CGAP_Pr28 Homo sapiens cDNA clone IMAGE:3248432 3', mRNA sequence.
1553559_at	2.27	LOC134285	hypothetical protein LOC134285
1555609_a_at	2.27	WIG1	p53 target zinc finger protein
219819_s_at	2.26	MRPS28	mitochondrial ribosomal protein S28
219628_at	2.26	WIG1	p53 target zinc finger protein
227063_at	2.26	MGC40107	phospholipid scramblase 3
201248_s_at	2.24	SREBF2	sterol regulatory element binding transcription factor 2
222465_at	2.23	C15orf15	chromosome 15 open reading frame 15
211137_s_at	2.22	ATP2C1	ATPase, Ca++ transporting, type 2C, member 1
218593_at	2.21	FLJ10377	hypothetical protein FLJ10377
225552_x_at	2.21	AKIP	hypothetical protein MGC3047
224573_at	2.17	MGC49942	hypothetical protein MGC49942
209665_at	2.17	CYB561D2	putative tumor suppressor 101F6
218793_s_at	2.15	SCML1	sex comb on midleg-like 1 (Drosophila)
226359_at	2.14	GTPBP1	GTP binding protein 1
201034_at	2.13	HADHSC	adducin 3 (gamma)
208050_s_at	2.13	CASP2	caspase 2, apoptosis-related cysteine protease (neural precursor cell expressed, developmentally down-regulated 2)
202572_s_at	2.12	DLGAP4	disks large-associated protein 4
214718_at	2.11	ODAG	ocular development-associated gene
221203_s_at	2.10	FLJ10201	hypothetical protein FLJ10201
218898_at	2.10	CT120	membrane protein expressed in epithelial-like lung adenocarcinoma
218212_s_at	2.10	MOCS2	molybdenum cofactor synthesis 2
228201_at	2.09	DKFZp761H079	7g22a10.x1 NCI_CGAP_Brn23 Homo sapiens cDNA clone IMAGE:3307194 3', mRNA sequence.
201114_x_at	2.09	PSMA7	proteasome (prosome, macropain) subunit, alpha type, 7
207396_s_at	2.08	ALG3	asparagine-linked glycosylation 3 homolog (yeast, alpha-1,3-mannosyltransferase)
1554321_a_at	2.08	NFS1	NFS1 nitrogen fixation 1 (S. cerevisiae)
218472_s_at	2.08	PELO	pelota homolog (Drosophila)
201753_s_at	2.07	ADD3	adducin 3 (gamma)
212244_at	2.06	GRINL1A	glutamate receptor, ionotropic, N-methyl D-aspartate-like 1A
217918_at	2.05	DNCL2A	dynein, cytoplasmic, light polypeptide 2A
224332_s_at	2.04	MRPL43	mitochondrial ribosomal protein L43
217917_s_at	2.03	DNCL2A	dynein, cytoplasmic, light polypeptide 2A
203510_at	2.02	MET	met proto-oncogene (hepatocyte growth factor receptor)
218495_at	2.01	UXT	ubiquitously-expressed transcript
219098_at	2.00	MYBBP1A	MYB binding protein (P160) 1a
219244_s_at	1.98	MRPL46	mitochondrial ribosomal protein L46
225261_x_at	1.98	TH1L	TH1-like (Drosophila)
225359_at	1.97	TIM14	homolog of yeast TIM14
212422_at	1.96	PDCD11	programmed cell death 11
223441_at	1.96	SLC17A5	solute carrier family 17 (anion/sugar transporter), member 5
1555789_s_at	1.95	MGC2941	GABA(A) receptor-associated protein
203667_at	1.93	TBCA	tubulin-specific chaperone a
202027_at	1.91	C22orf5	chromosome 22 open reading frame 5
211704_s_at	1.90	SPIN2	spindlin family, member 2
217843_s_at	1.88	VDRIP	vitamin D receptor interacting protein
209254_at	1.86	KIAA0265	KIAA0265 protein
225865_x_at	1.86	TH1L	Homo sapiens mRNA for putative protein TH1, partial, clone IMAGE ID 785447.
210946_at	1.85	PPAP2A	phosphatidic acid phosphatase type 2A
224345_x_at	1.85	E2IG5	
206632_s_at	1.85	APOBEC3B	apolipoprotein B mRNA editing enzyme, catalytic polypeptide-like 3B
217972_at	1.84	CHCHD3	coiled-coil-helix-coiled-coil-helix domain containing 3
225006_x_at	1.80	TH1L	TH1-like (Drosophila)
205633_s_at	1.79	ALAS1	aminolevulinate, delta-, synthase 1

220607_x_at	1.77	TH1L	TH1-like (Drosophila)
221641_s_at	1.73	ACATE2	likely ortholog of mouse acyl-Coenzyme A thioesterase 2, mitochondrial
203277_at	1.71	DFFA	DNA fragmentation factor, 45kDa, alpha polypeptide
223193_x_at	1.71	E2IG5	growth and transformation-dependent protein
200830_at	1.63	PSMD2	proteasome (prosome, macropain) 26S subunit, non-ATPase, 2
212600_s_at	1.60	UQCRC2	AV727381 HTC Homo sapiens cDNA clone HTCCEF02 5', mRNA sequence.
201527_at	1.57	ATP6V1F	ATPase, H+ transporting, lysosomal 14kDa, V1 subunit F
202716_at	1.54	PTPN1	protein tyrosine phosphatase, non-receptor type 1
214205_x_at	1.38	TXNL2	thioredoxin-like 2
